# Supplementary material for: Iron-catalyzed three-component amino(radio)fluorination of alkenes to unprotected β-(radio)fluoroamines
Source: Nat Commun. 2025 Dec 5;16:10917. doi: 10.1038/s41467-025-65880-z (PMC12680689; doi:10.1038/s41467-025-65880-z)
Supplement: Supplementary file 1 — Supplementary Information [file 41467_2025_65880_MOESM1_ESM.pdf]

Supplementary Information

Iron-Catalyzed Three-Component Amino(radio)fluorination of Alkenes to Unprotected β-(radio)Fluoroamines

Yang Li,[1,2#] Yu Zhou,[3,#] Mark R. Bortolus,[4,5,#] Xiaoxuan Zhang,[2] Zhitong Wang,[2] Dezhi Liu,[2] Hannah Le,[4,5] Oaikhena Z. Esezobor,[4] Jie Ni,[3] Qian Zhang,[2] Neil Vasdev,[4,5] Gui-Juan Cheng,*[,3] Chao Zheng,*[,4,5] and Junkai Fu*[,1,2]

[1] National Engineering Laboratory for Druggable Gene and Protein Screening, College of Life Science, Northeast Normal University, Changchun 130024, P. R. China

[2] Jilin Province Key Laboratory of Organic Functional Molecular Design & Synthesis, Department of Chemistry, Northeast Normal University, Changchun 130024, China

[3] Warshel Institute for Computational Biology, School of Medicine, The Chinese University of Hong Kong, Shenzhen, Shenzhen 518172, China

[4] Azrieli Centre for Neuro-Radiochemistry, Brain Health Imaging Centre, Campbell Family Mental Health Research Institute, Centre for Addiction and Mental Health (CAMH), Toronto, ON M5T 1R8, Canada

[5] Department of Chemistry, Pharmacology and Toxicology, and/or Psychiatry, University of Toronto, Toronto, ON M5T-1R8, Canada

[#] These authors contributed equally to this work

* Correspondence: fujk109@nenu.edu.cn (J. F.);
chao.zheng@camh.ca (C. Z.);
chengguijuan@cuhk.edu.cn (G. C.)

Table of Contents

Part 1: General information ---S3

Part 2: Supplementary data --S4

Part 3: Synthesis of alkene substrates--S13

Part 4: General procedure and characteristic data for products **2a**-**2av**-----------------------------------S14

Part 5: Procedure and characteristic data for derivatization--S32

Part 6: Procedure and characteristic data for control experiments---------------------------------------S43

Part 7: Radiochemistry---S48

Part 8: NMR spectra--S71

Part 9: References---S181

Part 1: General information

Unless otherwise noted, all reactions were carried out under an argon atmosphere as well as anhydrous conditions, and all reagents were purchased from commercial suppliers without further purification. Anhydrous tetrahydrofuran (THF) and diethyl ether (Et_2O) were distilled from sodium-benzophenone. Anhydrous 1, 2-dichloroethane (DCE), dichloromethane (DCM), acetonitrile (CH_3CN) and dimethylformamide (DMF) were distilled from calcium hydride. Anhydrous toluene was distilled from sodium. Anhydrous chloroform ($CHCl_3$) was distilled from phosphorus pentoxide. $Et_3N{\cdot}3HF$ (98%) was purchased from Energy.

Reactions were monitored by Thin Layer Chromatography (TLC) on plates (GF254) supplied by Yantai Chemicals (China) visualized by UV or stained with ethanolic solution of phosphomolybdic acid and basic solution of $KMnO_4$. The products were purified by column chromatography over silica gel (300-400 size).

The NMR spectra were recorded on a Brüker Advance 600 (^{1}H: 600 MHz, ^{13}C:150 MHz), Brüker Advance 500 (^{1}H: 500 MHz, ^{13}C: 125 MHz), or Brüker Advance 400 (^{1}H: 400 MHz), with TMS as the internal standard. The following abbreviations were used to denote multiplicities: s = singlet, d = doublet, t = triplet, q = quartet, dd = doublet of doublets, m = multiplet, br = broad.

IR spectra were recorded on an IRPrestige-21 FTIR spectrometer. High resolution mass spectrometric (HRMS) data was recorded on Brüker Apex IV RTMS by using ESI method.Radioactivity was quantified using a Capintec Radioisotope Calibrator (CRC-712M) ion chamber. Radio-TLC analysis was employed to determine radiochemical identity, purity, and yield. Radiochemical reactions were monitored by radio-TLC on aluminum oxide plates coated on aluminum foil (Catalog No. 103016) supplied by Analtech. Radio-TLC was visualized by UV and analyzed on an Eckert & Ziegler AR-2000 TLC Scanner. For analytic radio-HPLC, either a Synergi Fusion column (250 x 4.6 mm, 5 μm) or Phenomenex Prodigy column (250 x 4.6 mm, 5 μm) were used. The mobile phase comprised varying ratios of CH_3CN and 0.1% aqueous formic acid or 50 mM Ammonium Formate (40:60, 50:50, or 54:46) at a flow rate of 1-2 mL/min, with injection volumes between 20-80 μL. The HPLC systems consisted of a Waters 1515 Isocratic HPLC Pump equipped with a Waters 2487 Dual λ Absorbance Detector, and a Bioscan Flow-Count equipped with a NaI crystal, controlled via Breeze software, or a Prominence LC-20AT HPLC Pump equipped with a Prominence SPD-20A Absorbance detector, a Bicron frisk-tech radiation detector, controlled via eDAQ Powerchrom software.

Analysis of crude reaction mixtures and isolated products **2a'**, [^{18}F]KP23 and calibration curve was recorded using an Advion-Avant Interchim Scientific HPLC and UHPLC equipped with a Luna C(18)2 column (250 x 4.6 mm, 10 μm), A-2041 UV-DAD (2 Hz, 200 – 400 nm), A-2045/A-2046 UV-Vis DAD (20 Hz, 200 – 800 nm) and Carroll & Ramsey Associates (Model 105-S) analogue gamma detector. Calibration curves using authentic standards for the two substrates was generated by preparing samples of varying concentrations via serial dilution, starting with a solution of 0.2 mg in CH_3CN (1.0 mL). These were injected onto the HPLC system in 20 μL injection volumes from a 1.0 mL stock. UV response was measured by integrating the peak of interest on 254 nm wavelength.

Part 2: Supplementary data

2.1 The explanation for selective control of acyclic and cyclic internal alkenes

The differences for diastereocontrol of acyclic and cyclic internal alkenes can be explained through a stepwise mechanism. As shown below, after the addition of *N*-centered radical onto the acyclic internal alkene, the rotatable C–C bond allows for the formation of two aziridinium ion isomers. An *anti*-addition of fluoride ion to the aziridinium ion species then produces the β-fluoroamine product **2ah** as a mixture containing two diastereomeric pairs in a ratio of 1:1.

On the other hand, for cyclic internal alkene substrates, the conformation limitation will prohibit the free rotation of the C–C bond for carbon radical intermediate. This leads to the formation of single aziridinium ion intermediate, which undergoes *syn*-addition by fluoride ion to finally produce the *cis*-β-fluoroamine **2ag** as the major product (*cis*:*trans* = 7:1).

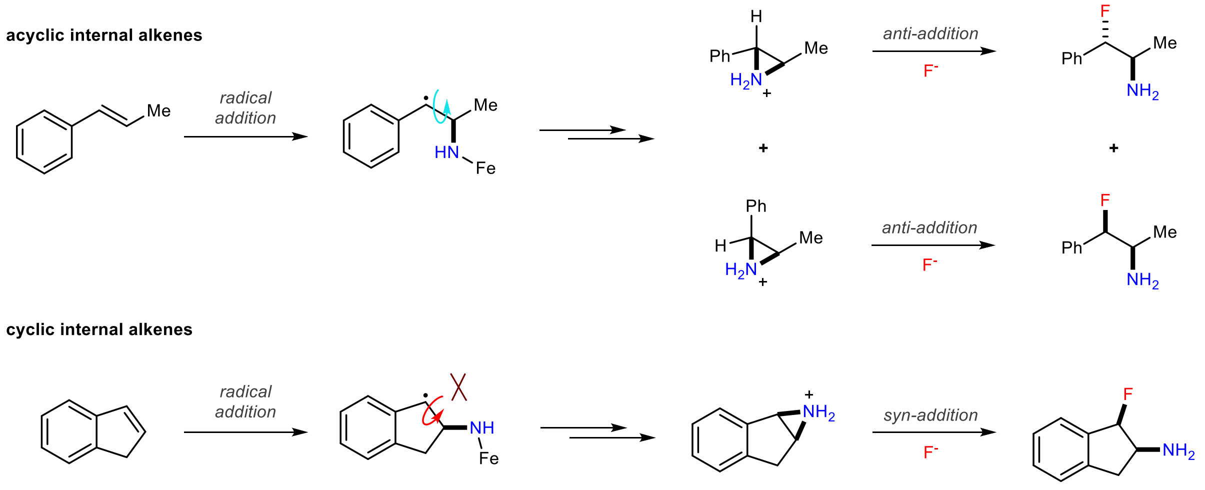

Supplementary Fig. 1. The explanation for selective control of acyclic and cyclic internal alkenes

2.2 ESI-MS experiment

We performed Mass spectrometry (MS) experiments by an AB SCIEX TripleTOF 6600 instrument equipped with an electrospray ion source. All substrates mentioned below were dissolved in DCM. FePc (5.7 mg, 10 µmol) was dissolved in the DCM (0.05 mL) and subsequently diluted 100-foldto give a 2 mM FePc solution. Similarly, an 8 mM solution of $Et_3N{\cdot}3HF$ was prepared. The FePc and $Et_3N{\cdot}3HF$ solutions were then mixed in a 1:1 ratio, yielding a solution containing 1mM FePc and 4 mM $Et_3N{\cdot}3HF$. After allowing the mixture to equilibrate at room temperature for approximately 5 minutes, it was injected into the instrument to record the source spectrum. In addition, we performed collision-induced dissociation (CID) experiments to detect fragment ions for the given *m*/*z* values under various CID voltages ranging from 10 V to 50 V. The CID spectrum was recorded under a CID voltage of 23 V.

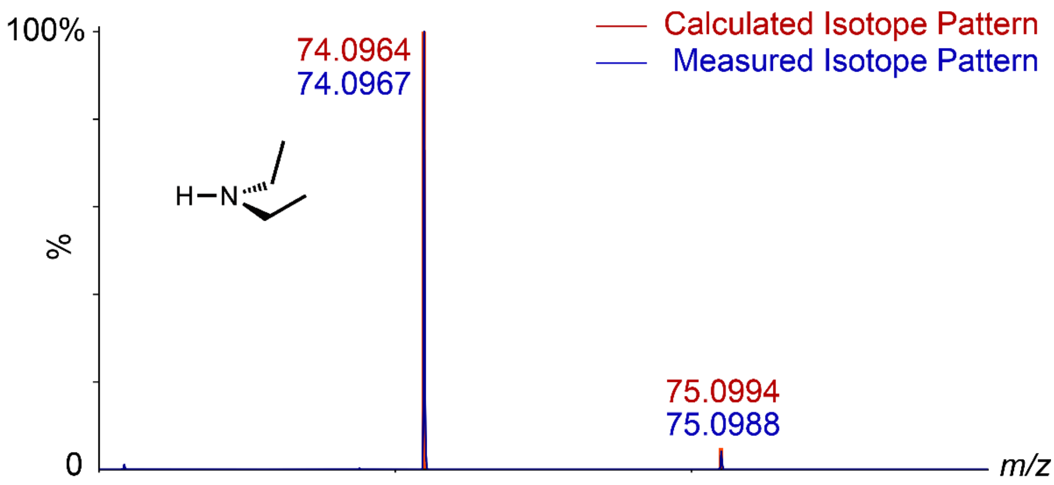

Supplementary Fig. 2. Characterization of $HNEt_2$ by mass spectrometry in the mixture of FePc and $Et_3N{\cdot}3HF$: calculated and measured isotope patterns are represented by red and blue line, respectively.

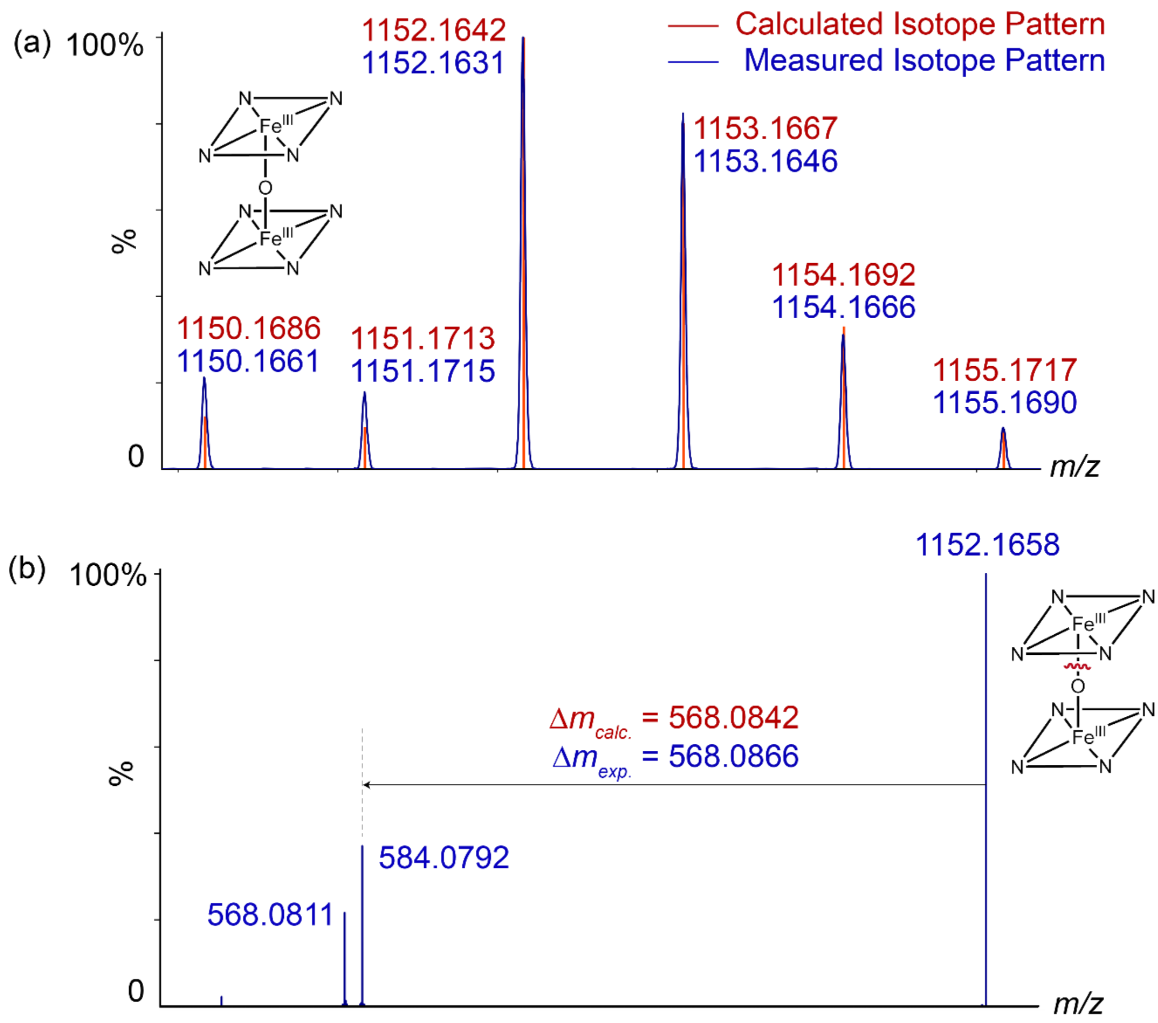

Supplementary Fig. 3. (a) Characterization of $(FePc)_2O$ by mass spectrometry in the DCM solvent of FePc: calculated and measured isotope patterns are shown in red and blue line, respectively. (b) Fragmentation analysis of *m*/*z* 1152.1631.

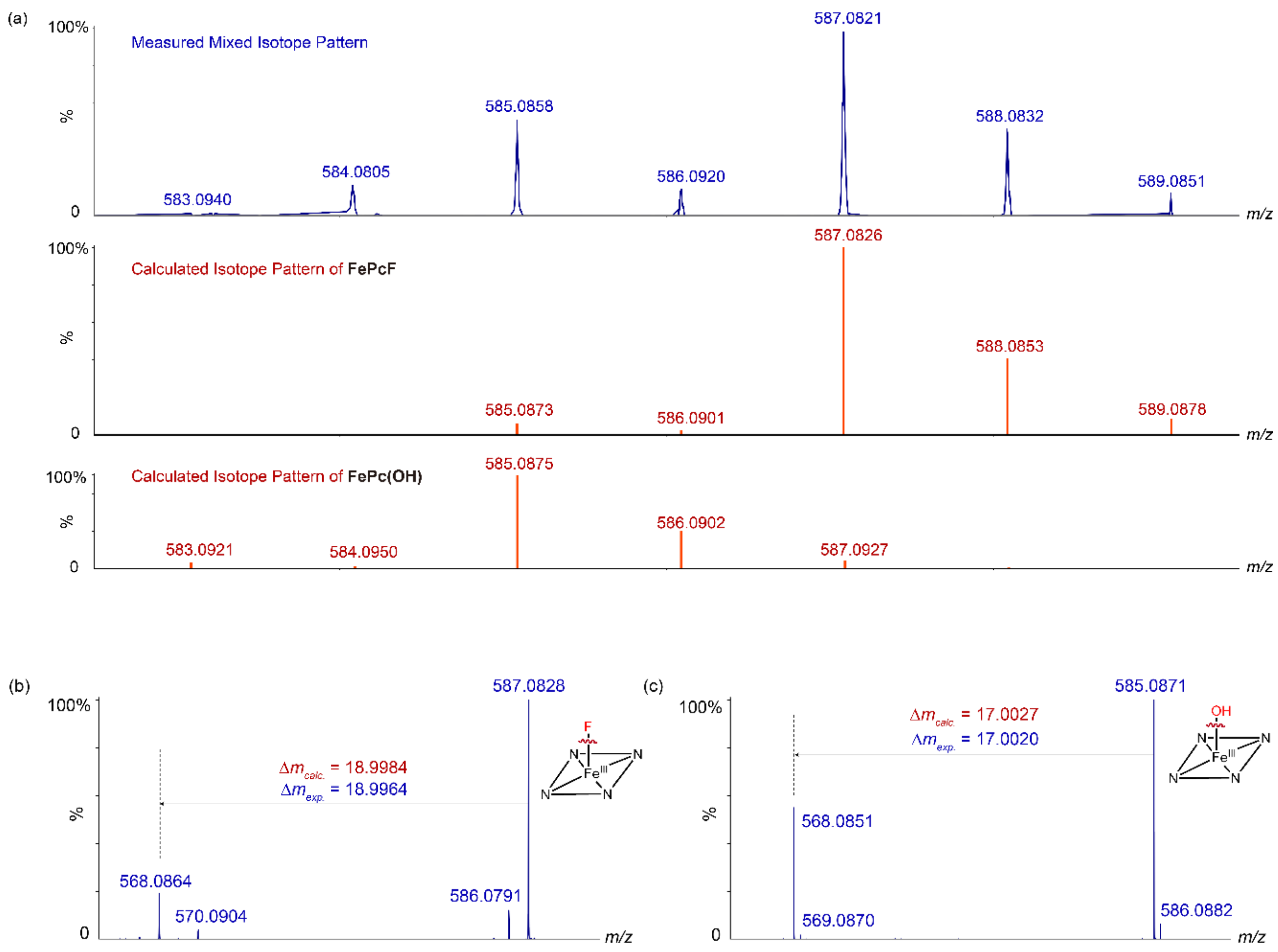

Supplementary Fig. 4. (a) Characterization of FePcF and FePc(OH) by mass spectrometry in the mixture of FePc and $Et_3N{\cdot}3HF$: calculated and measured isotope patterns are shown in red and blue line, respectively. (b) Fragmentation analysis of *m*/*z* 587.0828. (c) Fragmentation analysis of *m*/*z* 585.0871.

2.3 DFT calculations

Density functional theory (DFT) calculations were performed using the Gaussian 09 software[S1]. The B3LYP[S2-S4] functional, well-documented in benchmark studies for iron-oxo systems [S5-S6], has also demonstrated accuracy in related iron-nitrogen systems, as confirmed by recent electronic structure calculations[S7-S8] and theoretical Mössbauer spectroscopic parameter[S9]. Therefore, the B3LYP functional was selected for this study. Geometry optimization were carried out using the UB3LYP-D3/def2-SVP[S10] method. Frequency calculations were conducted to confirm that all intermediates had no imaginary frequencies, whiletransition states showed only one imaginary frequency. Additionally, intrinsic reaction coordinate (IRC) calculations were performed to verify that the transition states connected the correct forward and reverse intermediates. Spin populations were obtained through natural population analysis (NPA). Single point energies were calculated with the UB3LYP-D3/def2-TZVP method, and solvent effects were evaluated in dichloromethane within the SMD[S11] model.

We explored the reaction pathways across different spin states, presenting the most favorable spin surface in both the main text and supporting information. The spin state of the iron complex is denoted by the superscript in its label, while species without superscripts are in the triplet state.

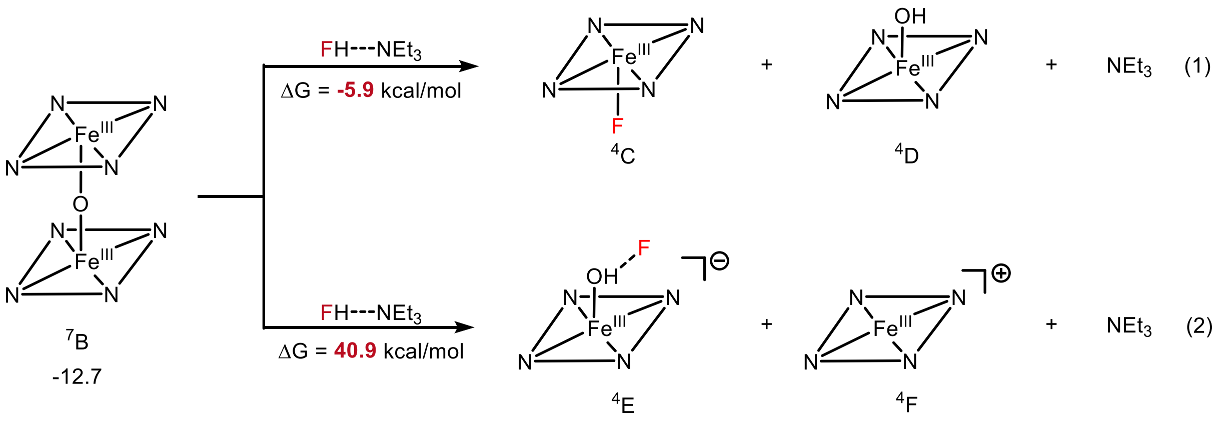

Supplementary Fig. 5. The decomposition of the diiron complex B into FePcF and FePc(OH) is thermodynamically favored in the presence of the fluorination reagent $Et_3N{\cdot}3HF$. The formation of FePcF and FePc(OH) was confirmed byESI-MS analysis. FePc (**A**) was set as the reference point.

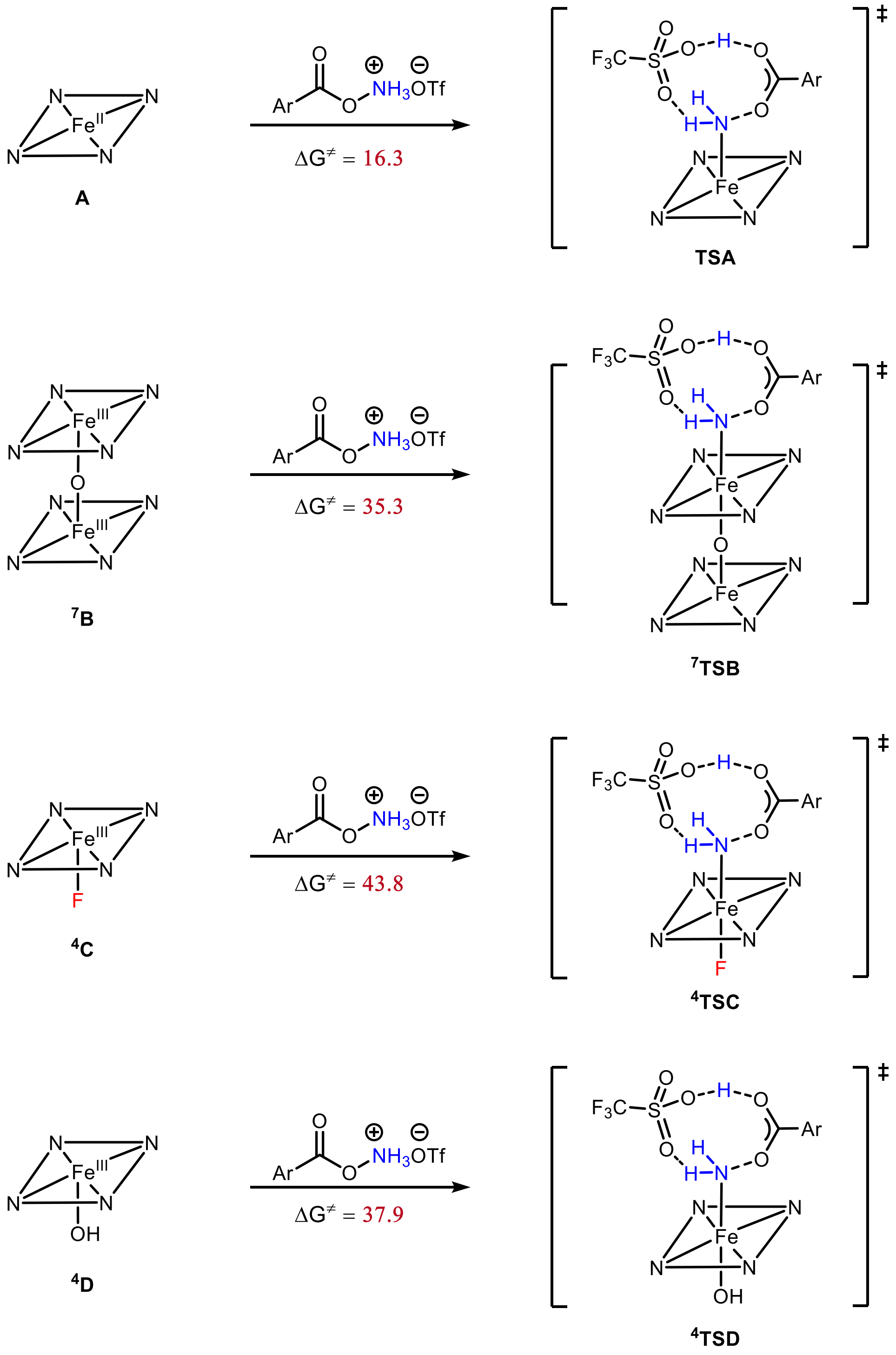

Supplementary Fig. 6. Activation of the hydroxylamine reagent by different iron complexes. FePc was quite facile to activate the hydroxylamine reagent, however, all of the ferric species (**B**, **C** and **D**) were not reactive toward the N–O bond cleavage. Free energies were given in kcal/mol. Ar = p-NO_2Ph.

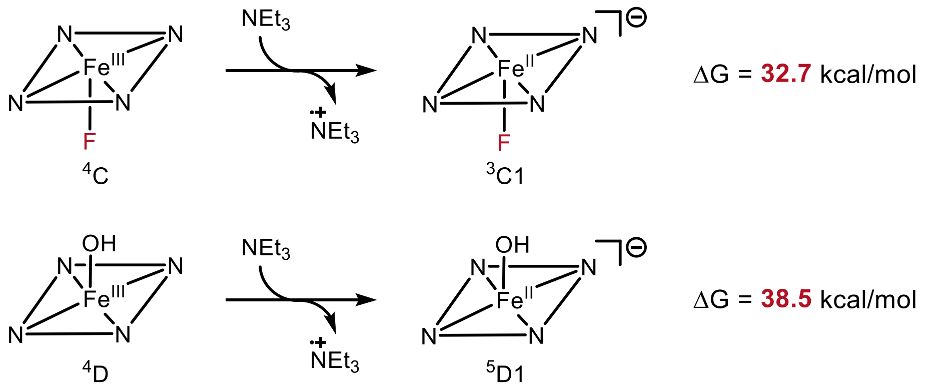

Supplementary Fig. 7. Reduction of ferric species via single electron transfer with Et_3N is not feasible.

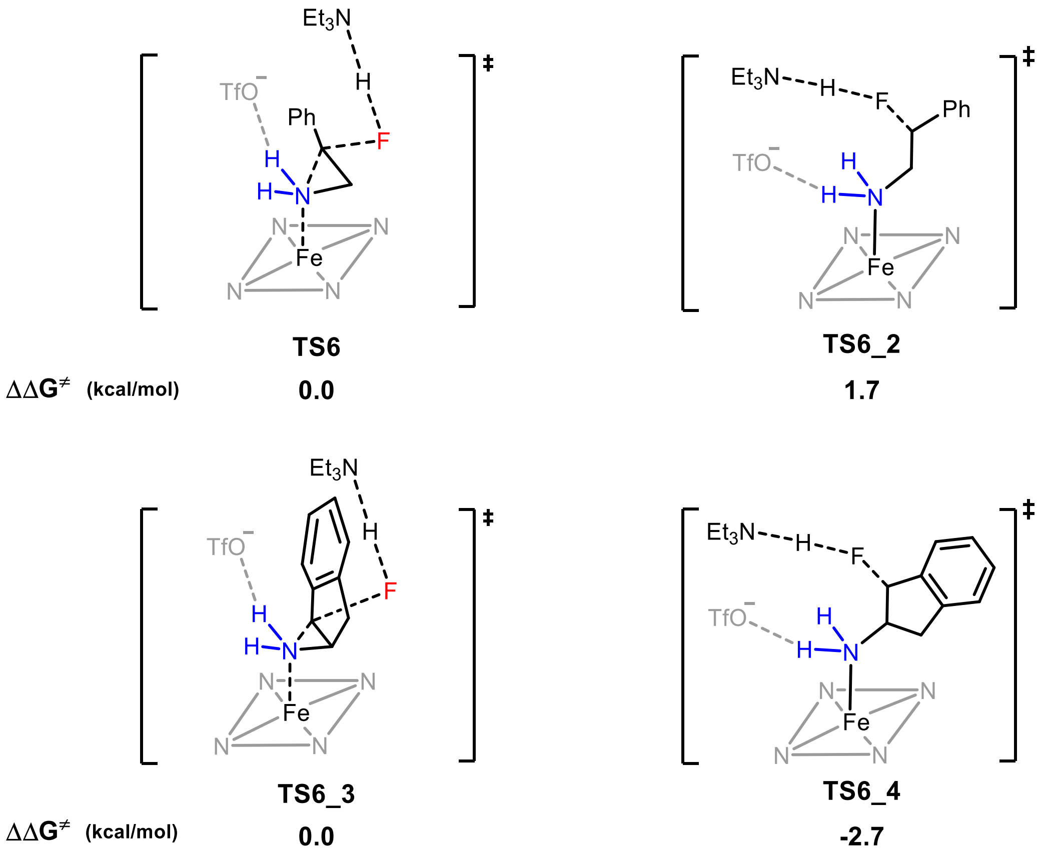

Supplementary Fig. 8. Calculated transition states of *syn*- and *anti*- addition for styrene and cyclic internal alkene (**1ag**) substrates.

Energy table

Supplementary Table 1. Zero-point energy (ZPE), thermal correction to Enthalpy (Hcorr), thermal correction to Gibbs Free Energy (Gcorr), singlet point energy in the solvent (Esol), and imaginary frequency (cm^{-1}) of transition states of optimized structures. Energies are given in Hartree.

Structures	ZPE	Hcorr	Gcorr	Esol	Imaginary frequency
**^{3}A**	0.41759	0.44557	0.36047	-2931.71287	\
**^{5}A**	0.41448	0.44332	0.35501	-2931.69529	\
**4-NO_2-BzONH_3-OTf**	0.17566	0.19658	0.12116	-1643.30777	\
**HF**	0.00929	0.01259	-0.00712	-100.49850	\
**Et_3N**	0.20473	0.21509	0.17074	-292.55231	\
**INT1**	0.19064	0.20145	0.15503	-291.89914	\
**Et_3N-HF**	0.21883	0.23102	0.18240	-393.07728	\
**Et_3N^+-HF**	0.20471	0.21518	0.16869	-292.36313	\
**H_2O**	0.02123	0.02501	0.00357	-76.47054	\
**Styrene**	0.13338	0.14104	0.10211	-309.78501	\
**4-NO_2-BzOH**	0.11881	0.12931	0.08246	-625.61007	\
**O_2**	0.00387	0.00717	-0.01609	-150.38998	\
**HNEt_2**	0.14886	0.15662	0.11867	-213.89811	\
**CH_3CHO**	0.05514	0.05998	0.03020	-153.90735	\
**TS1**	0.62052	0.66088	0.54709	-3324.14290	-881.98
**INT2**	0.20928	0.22081	0.17357	-367.81277	\
**TS2**	0.22189	0.23423	0.18505	-468.32833	-187.54
**^{1}B**	0.83858	0.89681	0.74923	-5938.66435	\
**^{3}B**	0.83939	0.89731	0.74975	-5938.66267	\
**^{5}B**	0.83827	0.89639	0.74871	-5938.66973	\
**^{7}B**	0.83823	0.89660	0.74694	-5938.67500	\
**^{9}B**	0.83503	0.89390	0.74348	-5938.63184	\
**^{2}C**	0.41991	0.44941	0.36128	-3031.59117	\
**^{4}C**	0.41941	0.44911	0.35986	-3031.61622	\
**^{6}C**	0.41782	0.44789	0.35715	-3031.60875	\
**^{2}D**	0.43104	0.46109	0.37191	-3007.55816	\

**^{4}D**	0.42924	0.45913	0.36975	-3007.56413	\
**^{6}D**	0.42766	0.45794	0.36703	-3007.56073	\
**^{2}E**	0.43053	0.46195	0.36972	-3107.56228	\
**^{4}E**	0.42970	0.46141	0.36728	-3107.57456	\
**^{6}E**	0.42949	0.46116	0.36776	-3107.54387	\
**^{2}F**	0.41721	0.44544	0.35993	-2931.52892	\
**^{4}F**	0.41835	0.44658	0.36036	-2931.52932	\
**^{6}F**	0.41430	0.44245	0.35602	-2931.49226	\
**^{3}C1**	0.41668	0.44648	0.35750	-3031.74894	\
**^{5}C1**	0.41585	0.44591	0.35574	-3031.73915	\
**^{3}D1**	0.42703	0.45750	0.36727	-3007.67596	\
**^{5}D1**	0.42629	0.45709	0.36568	-3007.68583	\
**TS3**	0.59096	0.64027	0.50500	-4575.01801	-633.66
**^{5}TS3**	0.58805	0.63784	0.50125	-4574.97765	-670.65
**^{7}TSB**	1.01086	1.08978	0.89490	-7581.95333	-1086.62
**^{4}TSC**	0.59097	0.64161	0.50401	-4674.87409	-1042.15
**^{4}TSD**	0.60343	0.65485	0.51594	-4650.83651	-679.82
**INT3**	0.47158	0.51067	0.39908	-3949.43363	\
**INT4**	0.47118	0.51006	0.39852	-3949.42347	\
**TS4**	0.60501	0.65207	0.52213	-4259.22332	-187.24
**INT5**	0.61063	0.65696	0.52600	-4259.27241	\
**TS5**	0.61040	0.65642	0.52944	-4259.25341	-238.41
**INT6**	0.61188	0.65774	0.53059	-4259.28743	\
**TS6**	0.82989	0.88865	0.73154	-4652.35534	-464.70
**P**	0.20902	0.22731	0.16209	-1428.08590	\
**TS6_2**	0.83369	0.89138	0.74106	-4652.36056	-419.0882
**TS6_3**	0.83725	0.89569	0.74145	-4690.47636	-396.41
**TS6_4**	0.84079	0.89826	0.74923	-4690.48856	-356.28

Part 3: Synthesis of alkene substrates

All substrates were used as received from commercial suppliers or prepared according to published procedures unless otherwise stated.

1j is a known alkene according to ref. S12. **1s**, **1t** are known alkenes according to ref. S13. **1u** is a known alkene according to ref. S14. **1v**, **1af**, **1ap** are known alkenes according to ref. S15. **1w** is a known alkene according to ref. S16. **1y** is a known alkene according to ref. S17. **1aa** is a known alkene according to ref. S18. **1ab** is a known alkene according to ref. S19. **1ac** is a known alkene according to ref. S20. **1aj** is a known alkene according to ref. S21. **1ak** is a known alkene according to ref. S22. **1al** is a known alkene according to ref. S23. **1am** is a known alkene according to ref. S24. **1an** is a known alkene according to ref. S25. **1ao** is a known alkene according to ref. S26. **1as** is a known alkene according to ref. S27. **1at** is a known alkene according to ref. S28. **1au** is a known alkene according to ref. S29.

Procedure A:

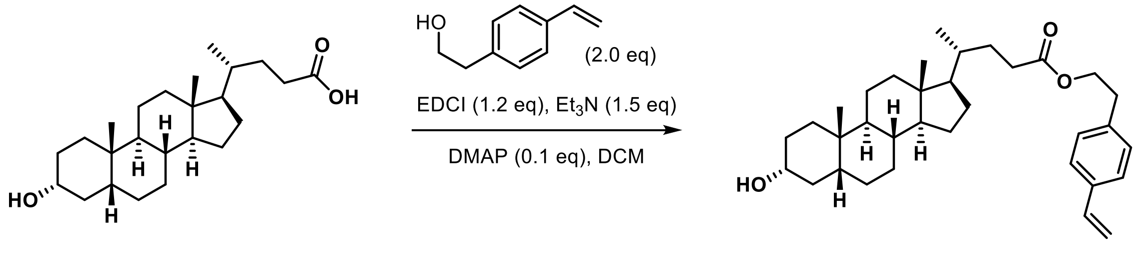

A 100-mL flask equipped with a stirring bar was charged with a solution of 4-ethenylbenzeneethanol (1.48 g, 10 mmol, 2.0 equiv), *N*-(3-dimethylaminopropyl)-*N'*-ethylcarbodiimide hydrochloride (EDCI, 1.15 g, 6.0 mmol, 1.2 equiv), triethylamine (1.0 mL, 7.5 mmol, 1.5 equiv), and 4-dimethylaminopyridine (DMAP, 61.1 mg, 0.50 mmol, 0.1 equiv) in dichloromethane (25 mL). Lithocholic acid (1.88 g, 5.0 mmol, 1.0 equiv) was then added to the reaction mixture at 0 °C. The mixture was stirred overnight at room temperature. The resulting mixture was diluted with DCM (50 mL) and washed sequentially with 1 M HCl, saturated aqueous $NaHCO_3$, and brine. The organic layer was dried over anhydrous Na_2SO_4, and the solvent was evaporated under reduced pressure. The crude product was purified by flash column chromatography using PE/EA = 4:1 as the eluent to afford the desired substrate **1aq** as a colorless oil (1.52 g, 3.0 mmol, 60%). **^{1}H NMR** (600 MHz, $CDCl_3$) δ 7.33 (d, *J* = 8.4 Hz, 2H), 7.16 (d, *J* = 8.4 Hz, 2H), 6.68 (dd, *J* = 17.4, 10.8 Hz, 1H), 5.70 (d, *J* = 17.4 Hz, 1H), 5.20 (d, *J* = 10.8 Hz, 1H), 4.26 (t, *J* = 7.2 Hz, 2H), 3.59 (m, 1H), 2.90 (t, *J* = 7.2 Hz, 2H), 2.53 – 2.42 (m, 1H), 2.31 (m, 1H), 2.18 (m, 1H), 1.93 (dt, *J* = 12.0, 3.0 Hz, 1H), 1.83 (m, 2H), 1.77 – 1.73 (m, 2H), 1.67 – 1.63 (m, 1H), 1.57 – 1.48 (m, 2H), 1.42 – 1.31 (m, 8H), 1.29 – 1.19 (m, 4H), 1.13 (m, 1H), 1.08 (m, 2H), 1.04 (m, 2H), 0.96 (dd, *J* = 14.4, 3.6 Hz, 1H), 0.91 (s, 3H), 0.88 (d, *J* = 6.6 Hz, 3H), 0.62 (s, 3H). **^{13}C NMR** (150 MHz, $CDCl_3$) δ 174.2, 137.5, 136.5, 135.9, 129.0, 126.3, 113.4, 71.6, 64.6, 56.4,

55.9, 42.7, 42.1, 40.4, 40.1, 36.3, 35.8, 35.4, 35.3, 34.8, 34.5, 31.2, 30.9, 30.4, 28.1, 27.2, 26.4, 24.2, 23.4, 20.8, 18.2, 12.0. **IR** ν_{max} (film): 3384, 2933, 2868, 1716, 1515, 1451, 1370, 1257, 1160, 1050, 843 cm^{-1}.

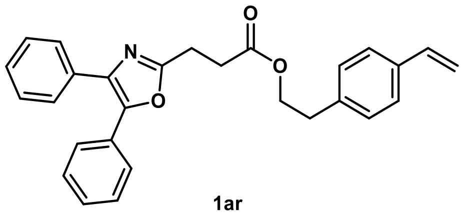

1ar

Substrate **1ar** was prepared following procedure A, except that oxaprozin (1.47 g, 5.0 mmol, 1.0 equiv) was used to replace lithocholic acid. Purification using column chromatography (PE/EA = 4:1) afforded **1an** as a yellow oil (1.36 g, 3.2 mmol, 64%).^{1}**H NMR** (600 MHz, $CDCl_3$) δ 7.67 – 7.61 (m, 2H), 7.58 – 7.52 (m, 2H), 7.40 – 7.26 (m, 8H), 7.14 (d, *J* = 7.8 Hz, 2H), 6.66 (dd, *J* = 17.4, 10.8 Hz, 1H), 5.69 (d, *J* = 17.4 Hz, 1H), 5.19 (d, *J* = 10.8 Hz , 1H), 4.32 (t, *J* = 7.2 Hz, 2H), 3.18 – 3.12 (m, 2H), 2.94 – 2.86 (m, 4H). ^{13}**C NMR** (150 MHz, $CDCl_3$) δ 171.9, 161.8, 145.5, 137.4, 136.5, 136.0, 135.2, 132.5, 129.1, 129.0, 128.7, 128.6, 128.5, 128.1, 128.0, 126.5, 126.4, 113.5, 65.2, 34.8, 31.2, 23.6. **IR** ν_{max} (film): 3432, 3023, 2956, 2116, 1717, 1645, 1499, 1370, 1276, 1159, 983, 854, 777 cm^{-1}.

Part 4: General procedure and characteristic data for products 2a-2av

The general procedure A:

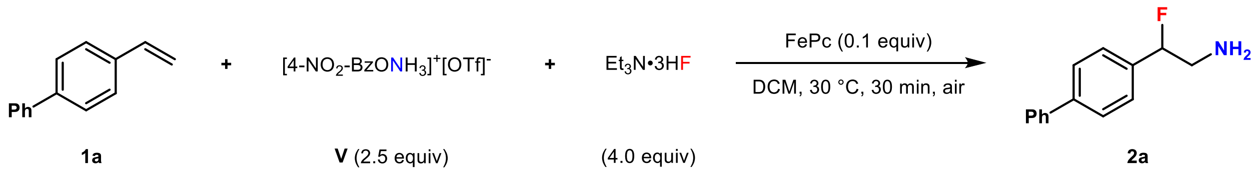

Hydroxylamine reagent 4-NO_2-BzONH_3OTf (**V**) (249 mg, 0.75 mmol, 2.5 equiv) was added to a plastic centrifuge tube charged with **1a** (54.1 mg, 0.30 mmol, 1.0 equiv), FePc (17.0 mg, 0.03 mmol, 0.1 equiv), $Et_3N \cdot 3HF$ (196 μL, 1.2 mmol, 4.0 equiv), and anhydrous CH_2Cl_2 (2.0 mL). The mixture was stirred in air at 30 °C (oil bath) for 30 min. Upon completion, the reaction was quenched with Et_3N (0.5 mL) at 0 °C. The mixture was direct purified by flash column chromatography (DCM/MeOH = 20:1 as eluent) to afford β-fluoroamine **2a** as a yellow oil (47.1 mg, 0.22 mmol, 73%). ^{1}**H NMR** (600 MHz, $CDCl_3$) δ 7.65 – 7.56 (m, 4H), 7.47 – 7.42 (m, 2H), 7.41 (d, *J* = 8.4 Hz, 2H), 7.39 – 7.34 (m, 1H), 5.58 (ddd, *J* = 48.0, 7.2, 3.6 Hz, 1H), 3.22 – 3.06 (m, 2H), 1.44 (s, 2H). ^{13}**C NMR** (150 MHz, $CDCl_3$) δ 141.6, 140.7, 137.1 (d, *J* = 19.8 Hz), 129.0, 127.6, 127.4, 127.3, 126.1 (d, *J* = 7.2 Hz), 95.8 (d, *J* = 170.6 Hz), 48.3 (d, *J* = 24.8 Hz). ^{19}**F NMR** (565 MHz, $CDCl_3$) δ -184.6 – -185.3 (m). **IR** ν_{max} (film): 3714, 3609, 3432, 3344, 2933, 2844, 2586, 1749, 1643, 1507 cm^{-1}. **HRMS** (ESI) m/z calcd for $C_{14}H_{14}FNNa$ $[M+Na]^+$: 238.1002; found: 238.1003.

The general procedure B:

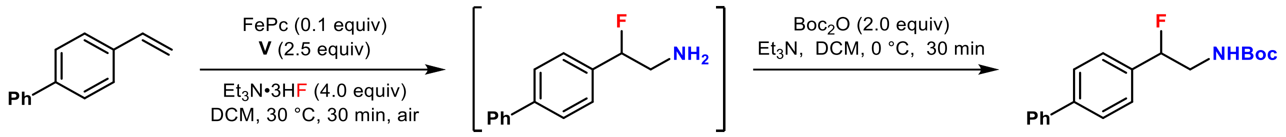

1a 2a 2a'

Hydroxylamine reagent 4-NO_2-$BzONH_3OTf$ (**V**) (249 mg, 0.75 mmol, 2.5 equiv) was added to a plastic centrifuge tube charged with **1a** (54.1 mg, 0.30 mmol, 1.0 equiv), FePc (17.0 mg, 0.03 mmol, 0.1 equiv), Et_3N·3HF (196 µL, 1.2 mmol, 4.0 equiv), and anhydrous CH_2Cl_2 (2.0 mL). The mixture was stirred under air at 30 °C (oil bath) for 30 min. Upon completion, the reaction was quenched with Et_3N (0.50 mL), followed by the dropwise addition of Boc_2O (138 µL, 0.60 mmol, 2.0 equiv) at 0 °C. The mixture was further stirred for 30 min at this temperature. Direct purification by flash column chromatography (PE/EA = 20:1 as eluent) afforded Boc-protected β-fluoroamine **2a'** as a white solid (67.2 mg, 0.21 mmol, 71%). **^{1}H NMR** (600 MHz, $CDCl_3$) δ 7.61 (d, *J* = 7.8 Hz, 2H), 7.58 (d, *J* = 7.2 Hz, 2H), 7.46 – 7.39 (m, 4H), 7.36 (t, *J* = 7.2 Hz, 1H), 5.58 (dd, *J* = 48.6, 6.0 Hz, 1H), 4.96 (s, 1H), 3.77 – 3.64 (m, 1H), 3.50 – 3.36 (m, 1H), 1.45 (s, 9H). **^{13}C NMR** (150 MHz, $CDCl_3$) δ 155.9, 141.8, 140.7, 136.4 (d, *J* = 19.8 Hz), 129.0, 127.7, 127.5, 127.3, 126.1 (d, *J* = 7.0 Hz), 93.2 (d, *J* = 172.2 Hz), 79.9, 46.5 (d, *J* = 24.3 Hz), 28.5. **^{19}F NMR** (565 MHz, $CDCl_3$) δ -183.6 (ddd, *J* = 48.5, 31.3, 17.7 Hz). **IR** ν_{max} (film): 3005, 2875, 1685, 1560, 1535, 1467, 1425, 1271, 1166, 846, 739 cm^{-1}. **HRMS** (ESI) m/z calcd for $C_{19}H_{22}FNO_2Na$ [M+Na]$^+$: 338.1527; found: 338.1536.

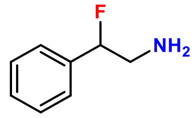

2b

Product **2b** was prepared following the general procedure A. Purification using column chromatography (DCM/MeOH = 20:1 as eluent) afforded **2b** as a yellow oil (30.1 mg, 0.22 mmol, 72%). **^{1}H NMR** (600 MHz, $CDCl_3$) δ 7.41 – 7.36 (m, 2H), 7.35 – 7.28 (m, 3H), 5.44 (ddd, *J* = 48.0, 7.2, 3.6 Hz, 1H), 3.16 – 3.00 (m, 2H), 1.76 (s, 2H). **^{13}C NMR** (150 MHz, $CDCl_3$) δ 138.1 (d, *J* = 19.3 Hz), 128.62, 128.56, 125.6 (d, *J* = 7.1 Hz), 95.7 (d, *J* = 171.4 Hz), 48.2 (d, *J* = 24.6 Hz). **^{19}F NMR** (565 MHz, $CDCl_3$) δ -175.2 – -190.7 (m). **IR** ν_{max} (film): 2920, 2841, 1675, 1548, 1455, 1348, 987, 758, 688 cm^{-1}. **HRMS** (ESI) m/z calcd for $C_8H_{11}FN$ [M+H]$^+$: 140.0870; found: 140.0875.

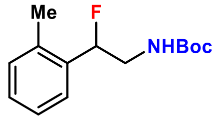

2c'

Product **2c'** was prepared following the general procedure B. Purification using column chromatography (PE/EA = 20:1 as eluent) afforded Boc-protected β-fluoroamine **2c'** as a yellow oil (46.3 mg, 0.18 mmol, 61%). **^{1}H NMR**

(600 MHz, $CDCl_3$) δ 7.33 – 7.28 (m, 1H), 7.18 – 7.17 (m, 1H), 7.17 – 7.15 (m, 1H), 7.12 – 7.07 (m, 1H), 5.66 (dd, J = 48.6, 8.4 Hz, 1H), 4.91 (s, 1H), 3.65 – 3.51 (m, 1H), 3.30 – 3.15 (m, 1H), 2.31 (s, 3H), 1.38 (s, 9H). **^{13}C NMR** (150 MHz, $CDCl_3$) δ 155.9, 135.6 (d, J = 18.6 Hz), 134.8, 130.7, 128.6, 126.3, 125.2 (d, J = 8.7 Hz), 91.2 (d, J = 170.7 Hz), 79.8, 45.7 (d, J = 23.7 Hz), 28.5, 19.0. **^{19}F NMR** (565 MHz, $CDCl_3$) δ -186.0 (ddd, J = 49.2, 33.1, 17.3 Hz). **IR** ν_{max} (film): 3470, 3347, 2918, 1672, 1514, 1459, 1365, 1253, 1246, 1173, 1041, 732 cm^{-1}. **HRMS** (ESI) m/z calcd for $C_{14}H_{21}FNO_2$ $[M+H]^+$: 254.1551; found: 254.1542.

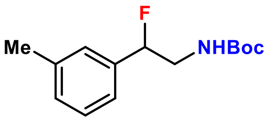

2d'

Product **2d'** was prepared following the general procedure B. Purification using column chromatography (PE/EA = 20:1) afforded **2d'** as a yellow oil (50.1 mg, 0.20 mmol, 66%). **^{1}H NMR** (600 MHz, $CDCl_3$) δ 7.21 – 7.17 (m, 1H), 7.11 – 7.03 (m, 3H), 5.42 (dd, J = 48.6, 8.4 Hz, 1H), 4.89 (s, 1H), 3.69 – 3.46 (m, 1H), 3.37 – 3.18 (m, 1H), 2.29 (s, 3H), 1.38 (s, 9H). **^{13}C NMR** (150 MHz, $CDCl_3$) δ 155.9, 138.4, 137.4 (d, J = 19.5 Hz), 129.5, 128.6, 126.3 (d, J = 6.9 Hz), 122.7 (d, J = 7.2 Hz), 93.5 (d, J = 171.9 Hz), 79.8, 46.6 (d, J = 24.1 Hz), 28.5, 21.5. **^{19}F NMR** (565 MHz, $CDCl_3$) δ -183.8 (ddd, J = 48.8, 32.3, 17.0 Hz). **IR** ν_{max} (film): 3440, 3332, 3041, 2908, 1701, 1509, 1491, 1368, 1256, 1172, 941, 795, 704 cm^{-1}. **HRMS** (ESI) m/z calcd for $C_{14}H_{20}FNO_2Na$ $[M+Na]^+$: 276.1370; found: 276.1379.

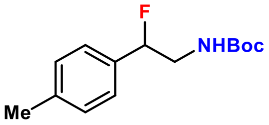

2e'

Product **2e'** was prepared following the general procedure B. Purification using column chromatography (PE/EA = 20:1) afforded **2e'** as a yellow oil (53.9 mg, 0.21 mmol, 71%). **^{1}H NMR** (600 MHz, $CDCl_3$) δ 7.25 – 7.21 (m, 2H), 7.19 (d, J = 7.8 Hz, 2H), 5.49 (dd, J = 48.6, 8.4 Hz, 1H), 4.94 (s, 1H), 3.73 – 3.53 (m, 1H), 3.43 – 3.28 (m, 1H), 2.35 (s, 3H), 1.45 (s, 9H). **^{13}C NMR** (150 MHz, $CDCl_3$) δ 155.9, 138.7, 134.5 (d, J = 19.7 Hz), 129.4, 125.7 (d, J = 6.6 Hz), 93.4 (d, J = 171.7 Hz), 79.8, 46.5 (d, J = 24.0 Hz), 28.5, 21.3. **^{19}F NMR** (565 MHz, $CDCl_3$) δ -182.3 (ddd, J = 48.7, 31.9, 17.0 Hz). **IR** ν_{max} (film): 3455, 3363, 3010, 2964, 1679, 1617, 1560, 1417, 1376, 1168, 869, 760, 724 cm^{-1}. **HRMS** (ESI) m/z calcd for $C_{14}H_{20}FNO_2Na$ $[M+Na]^+$: 276.1370; found: 276.1373.

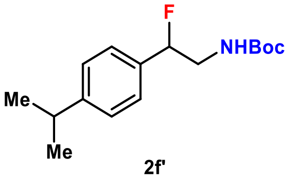

2f'

Product **2f'** was prepared following the general procedure B. Purification using column chromatography (PE/EA = 20:1) afforded **2f'** as a yellow oil (60.8 mg, 0.22 mmol, 72%). **^{1}H NMR** (600 MHz, $CDCl_3$) δ 7.22 – 7.18 (m, 2H),

7.18 – 7.15 (m, 2H), 5.43 (dd, J = 48.6, 8.4 Hz, 1H), 4.87 (s, 1H), 3.67 – 3.50 (m, 1H), 3.36 – 3.27 (m, 1H), 2.88 – 2.80 (m, 1H), 1.37 (s, 9H), 1.18 (d, J = 6.6 Hz, 6H). **^{13}C NMR** (150 MHz, $CDCl_3$) δ 155.9, 149.7, 134.8 (d, J = 19.6 Hz), 126.8, 125.8 (d, J = 6.7 Hz), 93.4 (d, J = 171.3 Hz), 79.8, 46.4 (d, J = 24.6 Hz), 34.0, 28.5, 24.1, 24.0. **^{19}F NMR** (565 MHz, $CDCl_3$) δ -182.2 (ddd, J = 48.5, 31.5, 17.0 Hz). **IR** ν_{max} (film): 3470, 3347, 3030, 2964, 1679, 1521, 1458, 1172, 843 cm^{-1}. **HRMS** (ESI) m/z calcd for $C_{16}H_{24}FNO_2Na$ $[M+Na]^+$: 304.1683; found: 304.1682.

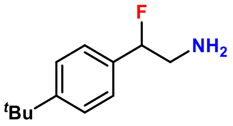

2g

Product **2g** was prepared following the general procedure A. Purification using column chromatography (DCM/MeOH = 20:1 as eluent) afforded **2g** as a yellow oil (43.9 mg, 0.23 mmol, 75%). **^{1}H NMR** (600 MHz, $CDCl_3$) δ 7.41 (d, J = 8.4 Hz, 2H), 7.26 (d, J = 8.4 Hz, 2H), 5.41 (ddd, J = 48.0, 7.2, 3.6 Hz, 1H), 3.13 (ddd, J = 19.2, 14.4, 7.2 Hz, 1H), 3.04 (ddd, J = 27.6, 14.4, 3.6 Hz, 1H), 1.52 (s, 2H), 1.32 (s, 9H). **^{13}C NMR** (150 MHz, $CDCl_3$) δ 151.7, 135.1 (d, J = 20.0 Hz), 125.6, 125.5 (d, J = 7.0 Hz), 95.8 (d, J = 170.4 Hz), 48.1 (d, J = 24.8 Hz), 34.7, 31.4. **^{19}F NMR** (565 MHz, $CDCl_3$) δ -183.7 (ddd, J = 47.0, 27.5, 18.8 Hz). **IR** ν_{max} (film): 2911, 2855, 1732, 1642, 1450, 1263, 1207, 1057, 1024, 874, 801 cm^{-1}. **HRMS** (ESI) m/z calcd for $C_{12}H_{18}FNNa$ $[M+Na]^+$: 218.1315; found: 218.1319.

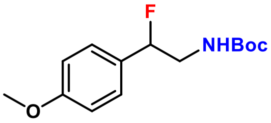

2h'

Product **2h'** was prepared following the general procedure B, except that the reaction was performed at 30 °C for 15 min. Purification using column chromatography (PE/EA = 20:1) afforded **2h'** as a yellow oil (60.6 mg, 0.23 mmol, 75%). **^{1}H NMR** (600 MHz, $CDCl_3$) δ 7.22 – 7.18 (m, 2H), 6.84 (d, J = 8.4 Hz, 2H), 5.39 (dd, J = 48.0, 8.4 Hz, 1H), 4.87 (s, 1H), 3.74 (s, 3H), 3.62 – 3.45 (m, 1H), 3.39 – 3.24 (m, 1H), 1.37 (s, 9H). **^{13}C NMR** (150 MHz, $CDCl_3$) δ 160.1, 155.9, 129.5 (d, J = 20.0 Hz), 127.3 (d, J = 6.3 Hz), 114.1, 93.2 (d, J = 171.0 Hz), 79.8, 55.4, 46.4 (d, J = 24.6 Hz), 28.5. **^{19}F NMR** (565 MHz, $CDCl_3$) δ -179.1 (ddd, J = 48.2, 31.5, 16.3 Hz). **IR** ν_{max} (film): 3416, 2933, 2844, 1641, 1515, 1411, 1168, 899, 769, 703 cm^{-1}. **HRMS** (ESI) m/z calcd for $C_{14}H_{20}FNO_3Na$ $[M+Na]^+$: 292.1319; found: 292.1320.

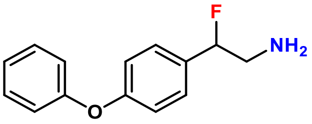

2i

Product **2i** was prepared following the general procedure A. Purification using column chromatography (DCM/MeOH = 20:1 as eluent) afforded **2i** as a yellow oil (49.3 mg, 0.21 mmol, 71%). **^{1}H NMR** (600 MHz, $CDCl_3$)

δ 7.37 – 7.32 (m, 2H), 7.29 (d, J = 8.4 Hz, 2H), 7.12 (t, J = 7.2 Hz, 1H), 7.04 – 6.96 (m, 4H), 5.42 (ddd, J = 48.0, 7.8, 3.6 Hz, 1H), 3.19 – 2.98 (m, 2H), 2.07 (s, 2H). **^{13}C NMR** (150 MHz, $CDCl_3$) δ 157.8, 156.9, 132.8 (d, J = 20.1 Hz), 130.0, 127.4 (d, J = 6.9 Hz), 123.7, 119.3, 118.7, 95.6 (d, J = 170.5 Hz), 48.2 (d, J = 24.8 Hz). **^{19}F NMR** (565 MHz, $CDCl_3$) δ -178.9 – -189.6 (m). **IR** ν_{max} (film): 2920, 2855, 1670, 1478, 1421, 1278, 1207, 993, 874, 787, 688 cm^{-1}. **HRMS** (ESI) m/z calcd for $C_{14}H_{14}FNNaO$ $[M+Na]^+$: 254.0952; found: 254.094

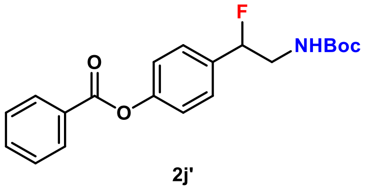

2j'

Product **2j'** was prepared following the general procedure B. Purification using column chromatography (PE/EA = 15:1) afforded **2j'** as a yellow oil (69.0 mg, 0.19 mmol, 64%). **^{1}H NMR** (600 MHz, $CDCl_3$) δ 8.13 (d, J = 6.6 Hz, 2H), 7.57 (t, J = 7.8 Hz, 1H), 7.44 (t, J = 7.8 Hz, 2H), 7.35 (d, J = 7.8 Hz, 2H), 7.18 (d, J = 7.8 Hz, 2H), 5.50 (dd, J = 48.6, 8.4 Hz, 1H), 4.90 (s, 1H), 3.71 – 3.48 (m, 1H), 3.37 – 3.20 (m, 1H), 1.39 (s, 9H). **^{13}C NMR** (150 MHz, $CDCl_3$) δ 165.2, 155.9, 151.3, 135.1 (d, J = 20.0 Hz), 133.9, 130.3, 129.5, 128.7, 126.9 (d, J = 7.1 Hz), 122.1, 92.9 (d, J = 173.1 Hz), 80.0, 46.5 (d, J = 24.0 Hz), 28.5. **^{19}F NMR** (565 MHz, $CDCl_3$) δ -183.4 (ddd, J = 48.5, 31.4, 17.1 Hz). **IR** ν_{max} (film): 3249, 3005, 2945, 1734, 1685, 1667, 1523, 1169, 965, 876, 701 cm^{-1}. **HRMS** (ESI) m/z calcd for $C_{20}H_{22}FNO_4Na$ $[M+Na]^+$: 382.1425; found: 382.1420.

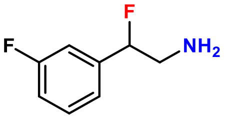

2k

Product **2k** was prepared following the general procedure A. Purification using column chromatography (DCM/MeOH = 20:1 as eluent) afforded **2k** as a yellow oil (30.6 mg, 0.20 mmol, 65%). **^{1}H NMR** (600 MHz, $CDCl_3$) δ 7.39 – 7.31 (m, 1H), 7.09 (d, J = 7.8 Hz, 1H), 7.08 – 6.99 (m, 2H), 5.44 (ddd, J = 48.0, 6.6, 4.2 Hz, 1H), 3.44 – 2.77 (m, 2H), 1.43 (s, 2H). **^{13}C NMR** (150 MHz, $CDCl_3$) δ 163.0 (d, J = 246.5 Hz), 140.8 (dd, J = 20.2, 7.3 Hz), 130.3 (d, J = 8.2 Hz), 121.1 (dd, J = 7.5, 2.9 Hz), 115.4 (d, J = 21.0 Hz), 112.7 (dd, J = 22.3, 8.2 Hz), 95.0 (d, J = 173.1 Hz), 48.2 (d, J = 24.5 Hz). **^{19}F NMR** (565 MHz, $CDCl_3$) δ -106.0 – -117.6 (m, 1H), -182.4 – -189.7 (m, 1H). **IR** ν_{max} (film): 2911, 2863, 1674, 1478, 1421, 1272, 1199, 993, 888, 787 cm^{-1}. **HRMS** (ESI) m/z calcd for $C_8H_{10}F_2N$ $[M+H]^+$: 158.0776; found: 158.0770.

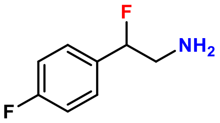

2l

Product **2l** was prepared following the general procedure A. Purification using column chromatography (DCM/MeOH = 20:1 as eluent) afforded **2l** as a yellow oil (32.5 mg, 0.21 mmol, 69%). **^{1}H NMR** (600 MHz, $CDCl_3$) δ 7.30 (dd, *J* = 8.4, 5.4 Hz, 2H), 7.07 (t, *J* = 8.4 Hz, 2H), 5.41 (ddd, *J* = 48.0, 7.8, 3.6 Hz, 1H), 3.14 – 2.95 (m, 2H), 1.67 (s, 2H). **^{13}C NMR** (150 MHz, $CDCl_3$) δ 162.9 (d, *J* = 246.7 Hz), 134.0 (dd, *J* = 20.8, 3.5 Hz), 130.2 – 125.7 (m), 115.6 (d, *J* = 21.2 Hz), 95.3 (d, *J* = 171.5 Hz), 48.3 (d, *J* = 24.8 Hz). **^{19}F NMR** (565 MHz, $CDCl_3$) δ -112.5 – -114.8 (m, 1H), -177.1 – -187.5 (m, 1H). **IR** ν_{max} (film): 2934, 2855, 1512, 1230, 1156, 993, 832 cm^{-1}. **HRMS** (ESI) m/z calcd for $C_8H_9F_2NNa$ $[M+Na]^+$: 180.0595; found: 180.0598.

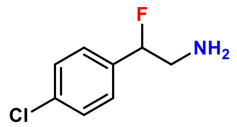

2m

Product **2m** was prepared following the general procedure A. Purification using column chromatography (DCM/MeOH = 20:1 as eluent) afforded **2m** as a yellow oil (26.6 mg, 0.15 mmol, 51%). **^{1}H NMR** (600 MHz, $CDCl_3$) δ 7.36 (d, *J* = 8.4 Hz, 2H), 7.26 (d, *J* = 8.4 Hz, 2H), 5.41 (ddd, *J* = 48.0, 7.2, 3.6 Hz, 1H), 3.17 – 2.78 (m, 2H), 1.34 (s, 2H). **^{13}C NMR** (150 MHz, $CDCl_3$) δ 136.7 (d, *J* = 20.9 Hz), 134.4, 128.8, 127.0 (d, *J* = 7.8 Hz), 95.2 (d, *J* = 172.5 Hz), 48.2 (d, *J* = 24.6 Hz). **^{19}F NMR** (565 MHz, $CDCl_3$) δ -185.5 (ddd, *J* = 47.8, 27.4, 20.3 Hz). **IR** ν_{max} (film): 2925, 2855, 1661, 1484, 1413, 1086, 1015, 874, 823 cm^{-1}. **HRMS** (ESI) m/z calcd for $C_8H_{10}FClN$ $[M+H]^+$: 174.0480; found: 174.0488.

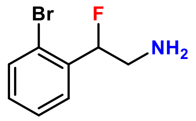

2n

Product **2n** was prepared following the general procedure A. Purification using column chromatography (DCM/MeOH = 20:1 as eluent) afforded **2n** as a yellow oil (29.4 mg, 0.14 mmol, 45%). **^{1}H NMR** (600 MHz, $CDCl_3$) δ 7.54 (d, *J* = 8.4 Hz, 1H), 7.47(dd, *J* = 7.8, 1.8 Hz, 1H), 7.37 (t, *J* = 7.8 Hz, 1H), 7.20 (td, *J* = 7.8, 1.8 Hz, 1H), 5.74 (ddd, *J* = 47.4, 7.2, 3.0 Hz, 1H), 3.17 (ddd, *J* = 29.4, 14.4, 3.0 Hz, 1H), 3.02 (ddd, *J* = 21.8, 14.4, 7.2 Hz, 1H) , 1.58 (s, 2H). **^{13}C NMR** (150 MHz, $CDCl_3$) δ 137.4 (d, *J* = 21.7 Hz), 132.8, 129.8, 127.7, 127.2 (d, *J* = 10.5 Hz), 120.7 (d, *J* = 5.9 Hz), 94.9 (d, *J* = 172.7 Hz), 46.8 (d, *J* = 24.1 Hz). **^{19}F NMR** (565 MHz, $CDCl_3$) δ -190.2 (ddd, *J* = 47.6, 31.0, 22.0 Hz). **IR** ν_{max} (film): 2911, 2863, 1610, 1455, 1263, 1024, 773, 730 cm^{-1}. **HRMS** (ESI) m/z calcd for $C_8H_9FBrNNa$ $[M+Na]^+$: 239.9795; found: 239.9798.

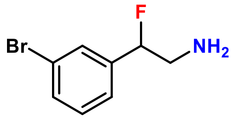

2o

Product **2o** was prepared following the general procedure A. Purification using column chromatography (DCM/MeOH = 20:1 as eluent) afforded **2o** as a yellow oil (31.4 mg, 0.14 mmol, 48%). **^{1}H NMR** (600 MHz, $CDCl_3$) δ 7.49 (s, 1H), 7.47 (d, *J* = 7.2 Hz, 1H), 7.27 – 7.23 (m, 2H), 5.40 (ddd, *J* = 48.0, 7.2, 3.6 Hz, 1H), 3.16 – 2.99 (m, 2H), 1.38 (s, 2H). **^{13}C NMR** (150 MHz, $CDCl_3$) δ 140.5 (d, *J* = 20.0 Hz), 131.6, 130.2, 128.7 (d, *J* = 8.1 Hz), 124.2 (d, *J* = 7.1 Hz), 122.8, 94.9 (d, *J* = 173.1 Hz), 48.2 (d, *J* = 24.6 Hz). **^{19}F NMR** (565 MHz, $CDCl_3$) δ -183.5 – -190.1 (m). **IR** ν_{max} (film): 2920, 2855, 2770, 1675, 1569, 1421, 1273, 1199, 987, 880, 787, 696 cm^{-1}. **HRMS** (ESI) m/z calcd for $C_8H_9FBrNNa$ [M+Na]$^+$: 239.9795; found: 239.9794.

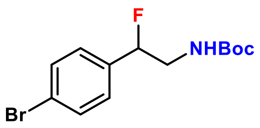

2p'

Product **2p'** was prepared following the general procedure B. Purification using column chromatography (PE/EA = 20:1) afforded **2p'** as a yellow oil (41.9 mg, 0.13 mmol, 44%). **^{1}H NMR** (500 MHz, $CDCl_3$) δ 7.51 (d, *J* = 8.0 Hz, 2H), 7.21 (d, *J* = 8.0 Hz, 2H), 5.49 (dd, *J* = 48.5, 8.5 Hz, 1H), 4.92 (s, 1H), 3.74 – 3.55 (m, 1H), 3.45 – 3.25 (m, 1H), 1.43 (s, 9H). **^{13}C NMR** (150 MHz, $CDCl_3$) δ 155.9, 136.5 (d, *J* = 20.1 Hz), 131.9, 127.3 (d, *J* = 7.1 Hz), 122.8 (d, *J* = 2.3 Hz), 92.8 (d, *J* = 173.3 Hz), 80.1, 46.4 (d, *J* = 24.0 Hz), 28.5. **^{19}F NMR** (565 MHz, $CDCl_3$) δ -184.8 (ddd, *J* = 48.9, 31.1, 18.1 Hz). **IR** ν_{max} (film): 3313, 3061, 2932, 1680, 1509, 1414, 1365, 1263, 1165, 838, 751, 690 cm^{-1}. **HRMS** (ESI) m/z calcd for $C_{13}H_{17}FBrNO_2Na$ [M+Na]$^+$: 340.0319; found: 340.0320.

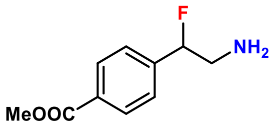

2q

Product **2q** was prepared following the general procedure A. Purification using column chromatography (DCM/MeOH = 20:1 as eluent) afforded **2q** as a yellow oil (24.8 mg, 0.13 mmol, 42%). **^{1}H NMR** (600 MHz, $CDCl_3$) δ 8.06 (d, *J* = 7.8 Hz, 2H), 7.40 (d, *J* = 7.8 Hz, 2H), 5.60 – 5.43 (m, 1H), 3.93 (s, 3H), 3.20 – 2.99 (m, 2H), 1.49 (s, 2H). **^{13}C NMR** (150 MHz, $CDCl_3$) δ 166.8, 143.1 (d, *J* = 19.9 Hz), 130.3, 129.9, 125.4 (d, *J* = 7.6 Hz), 95.3 (d, *J* = 173.5 Hz), 52.3, 48.2 (d, *J* = 23.7 Hz). **^{19}F NMR** (565 MHz, $CDCl_3$) δ -188.3 – -188.5 (m). **IR** ν_{max} (film): 2948, 2855, 1712, 1534, 1436,1263, 1193, 1114, 758, 716 cm^{-1}. **HRMS** (ESI) m/z calcd for $C_{10}H_{13}FNO_2$ [M+H]$^+$: 198.0925; found: 198.0929.

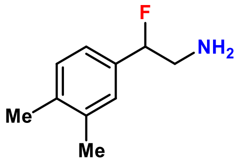

2s

Product **2s** was prepared following the general procedure A. Purification using column chromatography (DCM/MeOH = 20:1 as eluent) afforded **2s** as a yellow oil (34.6 mg, 0.21 mmol, 69%).**^{1}H NMR** (600 MHz, $CDCl_3$) δ 7.14 (d, *J* = 7.8 Hz, 1H), 7.09 (s, 1H), 7.05 (d, *J* = 7.8 Hz, 1H), 5.37 (ddd, *J* = 48.0, 7.2, 3.6 Hz, 1H), 3.11 (ddd, *J* = 19.2, 14.4, 7.2 Hz, 1H), 3.02 (ddd, *J* = 27.6, 14.4, 3.6 Hz, 1H), 2.28 (s, 3H), 2.26 (s, 3H), 1.46 (s, 2H). **^{13}C NMR** (150 MHz, $CDCl_3$) δ 137.1, 136.9, 135.6 (d, *J* = 19.9 Hz), 129.9, 127.0 (d, *J* = 7.0 Hz), 123.2 (d, *J* = 7.0 Hz), 96.0 (d, *J* = 170.4 Hz), 48.2 (d, *J* = 24.8 Hz), 19.9, 19.6. **^{19}F NMR** (565 MHz, $CDCl_3$) δ -183.3 (ddd, *J* = 47.4, 27.5, 18.6 Hz). **IR** ν_{max} (film): 2925, 2863, 1633, 1596, 1375, 1114, 1015, 880, 815, 773 cm^{-1}. **HRMS** (ESI) m/z calcd for $C_{10}H_{15}FN$ [M+H]$^+$: 168.1183; found: 168.1180.

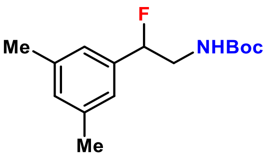

2t'

Product **2t'** was prepared following the general procedure B. Purification using column chromatography (PE/EA = 20:1) afforded **2t'** as a yellow oil (56.9 mg, 0.21 mmol, 71%). **^{1}H NMR** (600 MHz, $CDCl_3$) δ 6.91 – 6.84 (m, 3H), 5.38 (dd, *J* = 48.6, 8.4 Hz, 1H), 4.89 (s, 1H), 3.66 – 3.45 (m, 1H), 3.33 – 3.17 (m, 1H), 2.24 (s, 6H), 1.38 (s, 9H). **^{13}C NMR** (150 MHz, $CDCl_3$) δ 155.9, 138.3, 137.4 (d, *J* = 19.3 Hz), 130.4, 123.4 (d, *J* = 6.9 Hz), 93.5 (d, *J* = 172.0 Hz), 79.8, 46.7 (d, *J* = 24.0 Hz), 28.5, 21.4. **^{19}F NMR** (565 MHz, $CDCl_3$) δ -183.6 (ddd, *J* = 49.3, 32.8, 16.8 Hz). **IR** ν_{max} (film): 3470, 3363, 2918, 1674, 1537, 1521, 1261, 1173, 1059, 911, 852, 733 cm^{-1}. **HRMS** (ESI) m/z calcd for $C_{15}H_{22}FNO_2Na$ [M+Na]$^+$: 290.1527; found: 290.1526.

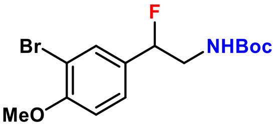

2u'

Product **2u'** was prepared following the general procedure B. Purification using column chromatography (PE/EA = 20:1) afforded **2u'** as a yellow oil (74.1 mg, 0.21 mmol, 71%). **^{1}H NMR** (600 MHz, $CDCl_3$) δ 7.48 (s, 1H), 7.20 – 7.16 (m, 1H), 6.83 (d, *J* = 9.0 Hz, 1H), 5.38 (dd, *J* = 48.0, 8.4 Hz, 1H), 4.86 (s, 1H), 3.83 (s, 3H), 3.60 – 3.46 (m, 1H), 3.37 – 3.22 (m, 1H), 1.37 (s, 9H). **^{13}C NMR** (150 MHz, $CDCl_3$) δ 156.3, 155.9, 131.0, 130.9 (d, *J* = 7.1 Hz), 126.2 (d, *J* = 6.6 Hz), 112.0, 111.9, 92.4 (d, *J* = 172.5 Hz), 80.0, 56.4, 46.4 (d, *J* = 24.8 Hz), 28.5. **^{19}F NMR** (565 MHz, $CDCl_3$) δ -180.9 – -181.3 (m). **IR** ν_{max} (film): 3332, 2964, 2850, 1690, 1659, 1521, 1461, 1168, 814, 736, 625 cm^{-1}. **HRMS** (ESI) m/z calcd for $C_{14}H_{19}FBrNO_3Na$ [M+Na]$^+$: 370.0425; found: 370.0426.

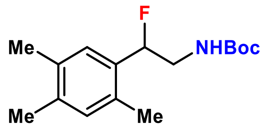

2v'

Product **2v'** was prepared following the general procedure B. Purification using column chromatography (PE/EA = 20:1) afforded **2v'** as a yellow oil (64.1 mg, 0.23 mmol, 76%). **^{1}H NMR** (600 MHz, $CDCl_3$) δ 7.05 (s, 1H), 6.86 (s, 1H), 5.59 (dd, J = 48.6, 8.4 Hz, 1H), 4.91 (s, 1H), 3.63 – 3.43 (m, 1H), 3.33 – 3.13 (m, 1H), 2.22 (s, 3H), 2.16 (s, 3H), 2.14 (s, 3H), 1.37 (s, 9H). **^{13}C NMR** (150 MHz, $CDCl_3$) δ 155.9, 137.0, 134.4, 132.9 (d, J = 18.5 Hz), 132.1, 132.0, 126.6 (d, J = 8.7 Hz), 91.2 (d, J = 170.1 Hz), 79.7, 45.8 (d, J = 24.5 Hz), 28.5, 27.5, 19.4 (d, J = 2.3 Hz), 18.4. **^{19}F NMR** (565 MHz, $CDCl_3$) δ -184.4 (ddd, J = 48.6, 33.0, 16.4 Hz). **IR** ν_{max} (film): 3448, 2917, 2836, 1688, 1552, 1539, 1411, 1168, 1040, 825 cm^{-1}. **HRMS** (ESI) m/z calcd for $C_{16}H_{24}FNO_2Na$ $[M+Na]^+$: 304.1683; found: 304.1683.

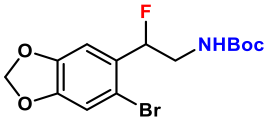

2w'

Product **2w'** was prepared following the general procedure B. Purification using column chromatography (PE/EA = 20:1) afforded **2w'** as a yellow oil (70.4 mg, 0.20 mmol, 65%). **^{1}H NMR** (500 MHz, $CDCl_3$) δ 6.98 (s, 1H), 6.91 (s, 1H), 5.98 (dd, J = 15.5, 1.5 Hz, 2H), 5.73 (dd, J = 48.0, 6.0 Hz, 1H), 4.86 (s, 1H), 3.75 – 3.55 (m, 1H), 3.49 – 3.24 (m, 1H), 1.42 (s, 9H). **^{13}C NMR** (150 MHz, $CDCl_3$) δ 155.7, 148.3, 147.3, 130.0 (d, J = 21.6 Hz), 112.9, 111.9, 107.2, 102.1, 92.4 (d, J = 174.1 Hz), 79.8, 44.9 (d, J = 24.2 Hz), 28.4. **^{19}F NMR** (565 MHz, $CDCl_3$) δ -185.3 (ddd, J = 47.9, 28.7, 19.8 Hz). **IR** ν_{max} (film): 3378, 3056, 2964, 1689, 1529, 1407, 1254, 1171, 1114, 867, 638 cm^{-1}. **HRMS** (ESI) m/z calcd for $C_{14}H_{18}FBrNO_4$ $[M+H]^+$: 362.0398; found: 362.0396.

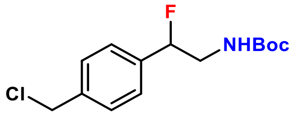

2x'

Product **2x'** was prepared following the general procedure B. Purification using column chromatography (PE/EA = 20:1) afforded **2x'** as a yellow oil (58.6 mg, 0.20 mmol, 68%). **^{1}H NMR** (600 MHz, $CDCl_3$) δ 7.34 (d, J = 7.8 Hz, 2H), 7.27 (d, J = 7.8 Hz, 2H), 5.47 (dd, J = 48.6, 8.4 Hz, 1H), 4.86 (s, 1H), 4.52 (s, 2H), 3.67 – 3.45 (m, 1H), 3.38 – 3.22 (m, 1H), 1.37 (s, 9H). **^{13}C NMR** (150 MHz, $CDCl_3$) δ 155.9, 138.1, 137.7 (d, J = 19.8 Hz), 128.9, 126.0 (d, J = 7.1 Hz), 93.0 (d, J = 172.8 Hz), 80.0, 46.5 (d, J = 23.5 Hz), 45.8, 28.5. **^{19}F NMR** (565 MHz, $CDCl_3$) δ -184.8 (ddd, J = 48.6, 31.4, 17.8 Hz). **IR** ν_{max} (film): 3378, 3010, 2933, 1690, 1674, 1521, 1364, 1250, 1166, 963, 854, 744 cm^{-1}. **HRMS** (ESI) m/z calcd for $C_{14}H_{19}FClNO_2Na$ $[M+Na]^+$: 310.0981; found: 310.0986.

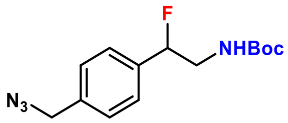

2y'

Product **2y'** was prepared following the general procedure B. Purification using column chromatography (PE/EA = 20:1) afforded **2y'** as a yellow oil (61.8 mg, 0.21 mmol, 70%). ^{1}**H NMR** (600 MHz, $CDCl_3$) δ 7.32 – 7.29 (m, 2H), 7.29 – 7.25 (m, 2H), 5.48 (dd, J = 48.6, 8.4 Hz, 1H), 4.88 (s, 1H), 4.28 (s, 2H), 3.67 – 3.50 (m, 1H), 3.37 – 3.19 (m, 1H), 1.37 (s, 9H). ^{13}**C NMR** (150 MHz, $CDCl_3$) δ 155.9, 137.6 (d, J = 19.7 Hz), 136.1, 128.5, 126.1 (d, J = 7.1 Hz), 93.1 (d, J = 172.6 Hz), 80.0, 54.5, 46.5 (d, J = 24.0 Hz), 28.5. ^{19}**F NMR** (565 MHz, $CDCl_3$) δ -184.6 (ddd, J = 48.9, 31.8, 17.8 Hz). **IR** ν_{max} (film): 3332, 3021, 2912, 2100, 1688, 1509, 1407, 1364, 1161, 1059, 963, 809, 710 cm^{-1}. **HRMS** (ESI) m/z calcd for $C_{14}H_{19}FN_4O_2Na$ $[M+Na]^+$: 317.1384; found: 317.1379.

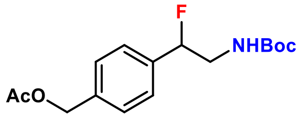

2z'

Product **2z'** was prepared following the general procedure B. Purification using column chromatography (PE/EA = 15:1) afforded **2z'** as a yellow oil (60.7 mg, 0.20 mmol, 65%). ^{1}**H NMR** (600 MHz, $CDCl_3$) δ 7.33 – 7.29 (m, 2H), 7.29 – 7.25 (m, 2H), 5.47 (dd, J = 48.6, 7.8 Hz, 1H), 5.04 (s, 2H), 4.88 (s, 1H), 3.75 – 3.45 (m, 1H), 3.38 – 3.22 (m, 1H), 2.04 (s, 3H), 1.37 (s, 9H). ^{13}**C NMR** (150 MHz, $CDCl_3$) δ 171.0, 155.9, 137.5 (d, J = 19.6 Hz), 136.6, 128.6, 125.8 (d, J = 7.3 Hz), 93.1 (d, J = 173.2 Hz), 79.9, 66.0, 46.5 (d, J = 24.3 Hz), 28.5, 21.1. ^{19}**F NMR** (565 MHz, $CDCl_3$) δ -184.5 (ddd, J = 48.8, 31.6, 17.4 Hz). **IR** ν_{max} (film): 3440, 3316, 2949, 2366, 1706, 1507, 1368, 1251, 1163, 963, 838, 740 cm^{-1}. **HRMS** (ESI) m/z calcd for $C_{16}H_{22}FNO_4Na$ $[M+Na]^+$: 334.1425; found: 334.1433.

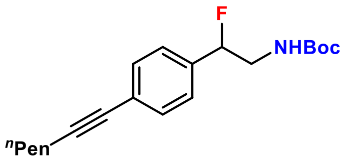

2aa'

Product **2aa'** was prepared following the general procedure B. Purification using column chromatography (PE/EA = 20:1) afforded **2aa'** as a yellow oil (60.0 mg, 0.18 mmol, 60%). ^{1}**H NMR** (600 MHz, $CDCl_3$) δ 7.33 (d, J = 7.8 Hz, 2H), 7.21 – 7.16 (m, 2H), 5.44 (dd, J = 48.6, 8.4 Hz, 1H), 4.85 (s, 1H), 3.74 – 3.44 (m, 1H), 3.36 – 3.16 (m, 1H), 2.32 (t, J = 7.2 Hz, 2H), 1.56 – 1.51 (m, 2H), 1.37 (s, 9H), 1.36 – 1.32 (m, 2H), 1.32 – 1.26 (m, 2H), 0.85 (t, J = 7.2 Hz, 3H). ^{13}**C NMR** (150 MHz, $CDCl_3$) δ 155.9, 136.6 (d, J = 19.7 Hz), 131.9, 125.5 (d, J = 7.2 Hz), 124.8, 93.1 (d, J = 172.3 Hz), 91.4, 80.2, 79.9, 46.4 (d, J = 23.1 Hz), 31.3, 28.5, 28.5, 22.4, 19.5, 14.1. ^{19}**F NMR** (565 MHz, $CDCl_3$) δ -184.5 (ddd, J = 48.6, 31.3, 17.5 Hz). **IR** ν_{max} (film): 3363, 2949, 2841, 2223, 1674, 1521, 1470, 1267, 1168, 1159, 964, 833, 734 cm^{-1}. **HRMS** (ESI) m/z calcd for $C_{20}H_{28}FNO_2Na$ $[M+Na]^+$: 356.1996; found: 356.1991.

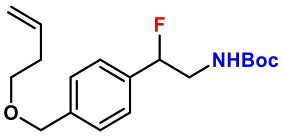

2ab'

Product **2ab'** was prepared following the general procedure B. Purification using column chromatography (PE/EA = 20:1) afforded **2ab'** as a yellow oil (53.3 mg, 0.17 mmol, 55%). **^{1}H NMR** (500 MHz, $CDCl_3$) δ 7.40 – 7.34 (m, 2H), 7.34 – 7.30 (m, 2H), 5.91 – 5.78 (m, 1H), 5.52 (dd, *J* = 49.0, 8.5 Hz, 1H), 5.10 (dd, *J* = 17.0, 1.5 Hz, 1H), 5.05 (d, *J* = 10.5 Hz, 1H), 4.96 (s, 1H), 4.52 (s, 2H), 3.73 – 3.58 (m, 1H), 3.53 (t, *J* = 7.0 Hz, 2H), 3.44 – 3.31 (m, 1H), 2.43 – 2.33 (m, 2H), 1.44 (s, 9H). **^{13}C NMR** (150 MHz, $CDCl_3$) δ 155.9, 139.3, 136.7 (d, *J* = 19.7 Hz), 135.3, 127.9, 125.7 (d, *J* = 7.0 Hz), 116.6, 93.2 (d, *J* = 172.4 Hz), 79.9, 72.6, 69.9, 46.5 (d, *J* = 24.4 Hz), 34.3, 28.5. **^{19}F NMR** (565 MHz, $CDCl_3$) δ -183.6 (ddd, *J* = 48.6, 31.3, 17.1 Hz). **IR** ν_{max} (film): 3455, 3332, 2933, 1695, 1644, 1509, 1424, 1365, 1254, 1161, 1076, 917, 865, 747 cm^{-1}. **HRMS** (ESI) m/z calcd for $C_{18}H_{27}FNO_3$ $[M+H]^+$: 324.1969; found: 324.1965.

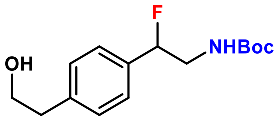

2ac'

Product **2ac'** was prepared following the general procedure B. Purification using column chromatography (PE/EA = 5:1) afforded **2ac'** as a yellow oil (51.0 mg, 0.18 mmol, 60%). **^{1}H NMR** (500 MHz, $CDCl_3$) δ 7.31 – 7.26 (m, 2H), 7.26 – 7.22 (m, 2H), 5.49 (dd, *J* = 49.0, 8.5 Hz, 1H), 4.97 (s, 1H), 3.84 (t, *J* = 6.5 Hz, 2H), 3.71 – 3.55 (m, 1H), 3.45 – 3.27 (m, 1H), 2.86 (t, *J* = 6.5 Hz, 2H), 1.43 (s, 9H). **^{13}C NMR** (150 MHz, $CDCl_3$) δ 155.9, 139.4, 135.6 (d, *J* = 19.8 Hz), 129.4, 125.9 (d, *J* = 6.9 Hz), 93.2 (d, *J* = 172.2 Hz), 79.9, 63.6, 46.5 (d, *J* = 24.3 Hz), 39.0, 28.5. **^{19}F NMR** (565 MHz, $CDCl_3$) δ -183.0 (ddd, *J* = 48.4, 31.3, 17.4 Hz). **IR** ν_{max} (film): 3455, 3332, 2918, 2821, 1705, 1689, 1516, 1464, 1367, 1267, 1162, 1050, 911, 848, 738 cm^{-1}. **HRMS** (ESI) m/z calcd for $C_{15}H_{22}FNO_3Na$ $[M+Na]^+$: 306.1476; found: 306.1475.

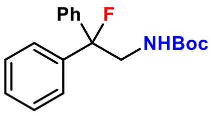

2ad'

Product **2ad'** was prepared following the general procedure B. Purification using column chromatography (PE/EA = 20:1) afforded **2ad'** as a yellow oil (65.2 mg, 0.21 mmol, 69%). **^{1}H NMR** (600 MHz, $CDCl_3$) δ 7.32 (d, *J* = 7.2 Hz, 4H), 7.28 (t, *J* = 7.8 Hz, 4H), 7.25 – 7.20 (m, 2H), 4.78 (s, 1H), 3.98 (dd, *J* = 24.6, 6.0 Hz, 2H), 1.27 (s, 9H). **^{13}C NMR** (150 MHz, $CDCl_3$) δ 155.8, 140.9 (d, *J* = 23.1 Hz), 128.5, 128.2, 125.8 (d, *J* = 7.9 Hz), 99.7 (d, *J* = 189.8 Hz), 79.7, 48.2 (d, *J* = 22.1 Hz), 28.4. **^{19}F NMR** (565 MHz, $CDCl_3$) δ -151.8 (t, *J* = 24.5 Hz). **IR** ν_{max} (film): 3455, 3332, 3041, 2964, 1706, 1631, 1507, 1411, 1365, 1251, 1164, 741, 701 cm^{-1}. **HRMS** (ESI) m/z calcd for $C_{19}H_{23}FNO_2$

[M+H]$^+$: 316.1707; found: 316.1710.

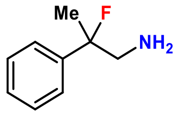

2ae

Product **2ae** was prepared following the general procedure A, except that the reaction was performed at 30 °C for 15 min. Purification using column chromatography (DCM/MeOH = 20:1 as eluent) afforded **2ae** as a yellow oil (33.5 mg, 0.22 mmol, 73%). **^{1}H NMR** (600 MHz, $CDCl_3$) δ 7.39 (t, J = 7.8 Hz, 2H), 7.35 – 7.29 (m, 3H), 3.05 (s, 1H), 3.02 (d, J = 3.0 Hz, 1H), 1.64 (d, J = 22.2 Hz, 3H), 1.32 (s, 2H). **^{13}C NMR** (150 MHz, $CDCl_3$) δ 142.7 (d, J = 22.2 Hz), 128.6, 127.6, 124.5 (d, J = 10.4 Hz), 98.2 (d, J = 172.6 Hz), 52.4 (d, J = 24.9 Hz), 25.1 (d, J = 24.7 Hz). **^{19}F NMR** (565 MHz, $CDCl_3$) δ -155.2 – -159.4 (m). **IR** ν_{max} (film): 2923, 2869, 1670, 1478, 1427, 1263, 1213, 987, 880, 787, 688 cm^{-1}. **HRMS** (ESI) m/z calcd for $C_9H_{13}FN$ [M+H]$^+$: 154.1027; found: 154.1025.

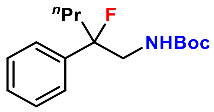

2af'

Product **2af'** was prepared following the general procedure B, except that the reaction was performed at 30 °C for 15 min. Purification using column chromatography (PE/EA = 20:1) afforded **2af'** as a yellow oil (53.1 mg, 0.19 mmol, 63%). **^{1}H NMR** (600 MHz, $CDCl_3$) δ 7.32 – 7.26 (m, 2H), 7.25 – 7.20 (m, 3H), 4.64 (s, 1H), 3.64 – 3.55 (m, 1H), 3.52 – 3.44 (m, 1H), 1.91 – 1.84 (m, 1H), 1.82 – 1.72 (m, 1H), 1.30 (s, 9H), 1.26 – 1.21 (m, 1H), 1.07 – 1.00 (m, 1H), 0.77 (t, J = 7.8 Hz, 3H). **^{13}C NMR** (150 MHz, $CDCl_3$) δ 156.1, 140.7 (d, J = 22.0 Hz), 128.4, 127.6, 124.9 (d, J = 10.0 Hz), 99.9 (d, J = 175.9 Hz), 79.6, 49.0 (d, J = 22.2 Hz), 39.8 (d, J = 22.7 Hz), 28.4, 16.5 (d, J = 4.1 Hz), 14.3. **^{19}F NMR** (565 MHz, $CDCl_3$) δ -163.5 – -164.1 (m). **IR** ν_{max} (film): 3301, 3071, 2879, 1659, 1513, 1401, 1367, 1287, 1240, 1164, 1034, 740 cm^{-1}. **HRMS** (ESI) m/z calcd for $C_{16}H_{25}FNO_2$ [M+H]$^+$: 282.1864; found: 282.1870.

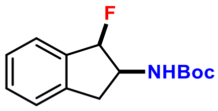

2ag', *cis*:*trans* = 7:1

Product **2ag'** was prepared following the general procedure B. Purification using column chromatography (PE/EA = 20:1) afforded **2ag'** as a yellow oil (*cis*:*trans* = 7:1, 46.7 mg, 0.19 mmol, 62%).

Major *cis*-diastereomeric pair (**2ag'-I**): **^{1}H NMR** (600 MHz, $CDCl_3$) δ 7.45 – 7.39 (m, 1H), 7.34 – 7.29 (m, 1H), 7.24 – 7.19 (m, 2H), 5.60 (dd, J = 58.2, 4.8 Hz, 1H), 5.09 (d, J = 9.6 Hz, 1H), 4.58 – 4.24 (m, 1H), 3.22 (dd, J = 15.0, 7.2 Hz, 1H), 2.89– 2.79 (m, 1H), 1.42 (s, 9H). **^{13}C NMR** (150 MHz, $CDCl_3$) δ 155.7, 143.1, 137.8 (d, J = 15.3 Hz), 131.0 (d, J = 4.5 Hz), 127.5 (d, J = 3.7 Hz), 126.6 (d, J = 2.7 Hz), 125.3 (d, J = 2.9 Hz), 94.6 (d, J = 176.8 Hz), 79.9, 53.7 (d, J = 19.1 Hz), 36.8, 28.5. **^{19}F NMR** (565 MHz, $CDCl_3$) δ -179.2 (dd, J = 58.3, 25.7 Hz). **IR** ν_{max} (film): 3440,

3332, 2979, 1705, 1688, 1508, 1303, 1252, 1165, 1054, 753 cm^{-1}. **HRMS** (ESI) m/z calcd for $C_{14}H_{18}FNO_2Na$ $[M+Na]^+$: 274.1214; found: 274.1210.

Minor *trans*-diastereomeric pair (**2ag'-II**) is a known compound. [1]

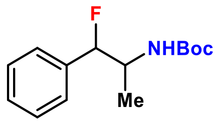

2ah'
two diastereomeric pairs in a 1:1 ratio

Product **2ah'** was prepared following the general procedure B. Purification using column chromatography (PE/EA = 20:1) afforded **2ah'** as a yellow oil (a mixture containing two diastereomeric pairs in a ratio of 1:1, 32.7 mg, 0.13 mmol, 43%).

One diastereomeric pair (**2ah'-I**): **^{1}H NMR** (500 MHz, $CDCl_3$) δ 7.41 – 7.34 (m, 2H), 7.35 – 7.27 (m, 3H), 5.63 (d, *J* = 48.0 Hz, 1H), 4.85 (s, 1H), 4.14 – 3.87 (m, 1H), 1.46 (s, 9H), 1.02 (d, *J* = 7.0 Hz, 3H). **^{13}C NMR** (150 MHz, $CDCl_3$) δ 155.3, 137.6 (d, *J* = 20.3 Hz), 128.5, 128.1, 125.1 (d, *J* = 8.8 Hz), 95.5 (d, *J* = 177.8 Hz), 79.7, 51.1 (d, *J* = 21.8 Hz), 28.5, 13.3. **^{19}F NMR** (565 MHz, $CDCl_3$) δ -202.4 (dd, *J* = 48.0, 27.7 Hz).

Another diastereomeric pair (**2ah'-II**): **^{1}H NMR** (500 MHz, $CDCl_3$) δ 7.39 – 7.34 (m, 2H), 7.34 – 7.27 (m, 3H), 5.40 (dd, *J* = 46.0, 5.5 Hz, 1H), 4.56 (s, 1H), 4.24 – 3.96 (m, 1H), 1.35 (s, 9H), 1.19 (d, *J* = 7.0 Hz, 3H). **^{13}C NMR** (150 MHz, $CDCl_3$) δ 155.3, 137.2 (d, *J* = 20.5 Hz), 128.5, 128.4, 126.0 (d, *J* = 7.7 Hz), 95.5 (d, *J* = 176.9 Hz), 79.6, 50.1 (d, *J* = 23.3 Hz), 28.4, 17.2. **^{19}F NMR** (565 MHz, $CDCl_3$) δ 190.6 (dd, *J* = 46.2, 19.5 Hz). **IR** ν_{max} (film): 3440, 3335, 2973, 2924, 1680, 1512, 1490, 1361, 1241, 1168, 1056, 785 cm^{-1}. **HRMS** (ESI) m/z calcd for $C_{14}H_{21}FNO_2$ $[M+H]^+$: 254.1551; found: 254.1549.

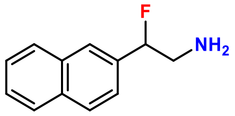

2ai

Product **2ai** was prepared following the general procedure A. Purification using column chromatography (DCM/MeOH = 20:1 as eluent) afforded **2ai** as a yellow oil (39.2 mg, 0.21 mmol, 69%). **^{1}H NMR** (600 MHz, $CDCl_3$) δ 7.89 – 7.83 (m, 3H), 7.81 (s, 1H), 7.55 – 7.47 (m, 2H), 7.42 (dd, *J* = 8.4, 1.8 Hz, 1H), 5.60 (ddd, *J* = 48.0, 7.2, 3.8 Hz, 1H), 3.26 – 3.08 (m, 2H), 1.40 (s, 2H). **^{13}C NMR** (150 MHz, $CDCl_3$) δ 135.5 (d, *J* = 19.9 Hz), 133.4, 133.1, 128.6, 128.2, 127.9, 126.6, 126.5, 125.0 (d, *J* = 8.2 Hz), 123.2 (d, *J* = 6.9 Hz), 96.0 (d, *J* = 172.4 Hz), 48.3 (d, *J* = 24.7 Hz). **^{19}F NMR** (565 MHz, $CDCl_3$) δ -185.2 (ddd, *J* = 46.8, 26.7, 19.8 Hz). **IR** ν_{max} (film): 2905, 2863, 1661, 1512, 1235, 950, 832, 739 cm^{-1}. **HRMS** (ESI) m/z calcd for $C_{12}H_{13}FN$ $[M+H]^+$: 190.1027; found: 190.1020.

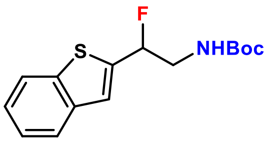

2aj'

Product **2aj'** was prepared following the general procedure B. Purification using column chromatography (PE/EA = 15:1) afforded **2aj'** as a yellow oil (51.3 mg, 0.17 mmol, 58%). ^{1}**H NMR** (600 MHz, $CDCl_3$) δ 7.79 – 7.74 (m, 1H), 7.72 – 7.68 (m, 1H), 7.33 – 7.27 (m, 2H), 7.27 – 7.24 (m, 1H), 5.75 (dd, *J* = 48.6, 4.8 Hz, 1H), 4.90 (s, 1H), 3.80 – 3.65 (m, 1H), 3.59 – 3.49 (m, 1H), 1.38 (s, 9H). ^{13}**C NMR** (150 MHz, $CDCl_3$) δ 155.9, 139.9, 139.8 (d, *J* = 21.4 Hz), 139.1, 125.1, 124.7, 124.2, 123.0 (d, *J* = 6.8 Hz), 122.6, 89.6 (d, *J* = 172.3 Hz), 80.1, 46.0 (d, *J* = 24.8 Hz), 28.5. ^{19}**F NMR** (565 MHz, $CDCl_3$) δ -170.1 (ddd, *J* = 46.4, 28.0, 16.8 Hz). **IR** ν_{max} (film): 3440, 3363, 2918, 2841, 1691, 1507, 1459, 1367, 1265, 1161, 1053, 740 cm^{-1}. **HRMS** (ESI) m/z calcd for $C_{15}H_{19}FSNO_2$ $[M+H]^+$: 296.1115; found: 296.1116.

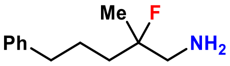

2ak

Product **2ak** was prepared following the general procedure A, except that the reaction was performed in DCE at 80 °C for 1 h. Purification using column chromatography (DCM/MeOH = 20:1 as eluent) afforded **2ak** as a yellow oil (31.6 mg, 0.16 mmol, 54%) with 15% of the starting materials **1ak** recovered (7.2 mg, 0.05 mmol). ^{1}**H NMR** (600 MHz, $CDCl_3$) δ 7.28 (dd, *J* = 8.4, 6.6, 2H), 7.20 – 7.17 (m, 3H), 2.82 – 2.66 (m, 2H), 2.66 – 2.60 (m, 2H), 1.76 – 1.58 (m, 4H), 1.28 (d, *J* = 21.8 Hz, 3H), 1.19 – 1.05 (m, 2H). ^{13}**C NMR** (150 MHz, $CDCl_3$) δ 142.1, 128.5, 126.0, 97.6 (d, *J* = 167.9 Hz), 50.1 (d, *J* = 24.2 Hz), 36.8 (d, *J* = 22.7 Hz), 36.2, 25.5 (d, *J* = 5.7 Hz), 21.8 (d, *J* = 24.7 Hz). ^{19}**F NMR** (565 MHz, $CDCl_3$) δ -152.5 – -155.5 (m). **IR** ν_{max} (film): 2939, 2863, 1498, 1455, 1199, 1094, 908, 832, 753, 688 cm^{-1}. **HRMS** (ESI) m/z calcd for $C_{12}H_{19}FN$ $[M+H]^+$: 196.1496; found: 196.1492.

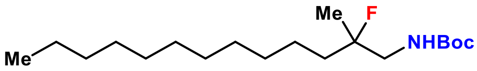

2al'

Product **2al'** was prepared following the general procedure B, except that the aminofluorination reaction was performed in DCE at 80 °C for 1 h. Purification using column chromatography (PE/EA = 20:1) afforded **2al'** as a yellow oil (48.7 mg, 0.15 mmol, 49%) with 20% of the starting materials **1al** recovered (11.8 mg, 0.06 mmol). ^{1}**H NMR** (600 MHz, $CDCl_3$) δ 4.81 (t, *J* = 6.6 Hz, 1H), 3.26 (dt, *J* = 21.6, 6.6 Hz, 2H), 1.65 – 1.50 (m, 2H), 1.43 (s, 9H), 1.38 – 1.34 (m, 2H), 1.32 – 1.22 (m, 19H), 0.86 (t, *J* = 6.6 Hz, 3H). ^{13}**C NMR** (150 MHz, $CDCl_3$) δ 156.3, 97.5 (d, *J* = 167.7 Hz), 79.5, 48.1 (d, *J* = 21.8 Hz), 37.5 (d, *J* = 21.9 Hz), 32.1, 30.1, 29.8, 29.77, 29.75, 29.6, 29.5, 23.6 (d, *J* = 6.2 Hz), 22.8, 21.7 (d, *J* = 24.3 Hz), 14.2. ^{19}**F NMR** (565 MHz, $CDCl_3$) δ -149.5 – -150.2 (m). **IR** ν_{max} (film):

3560, 3378, 3083, 2966, 2767, 1874, 1632, 1513, 1404, 1342, 1243, 1198, 1162, 1010 cm^{-1}. **HRMS** (ESI) m/z calcd for $C_{19}H_{39}FNO_2$ $[M+H]^+$: 332.2959; found: 332.2957.

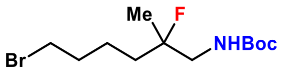

2am'

Product **2am'** was prepared following the general procedure B, except that the aminofluorination reaction was performed in DCE at 80 °C for 1 h. Purification using column chromatography (PE/EA = 20:1) afforded **2am'** as a yellow oil (51.5 mg, 0.17 mmol, 55%) with 17% of the starting materials **1am** recovered (9.0 mg, 0.05 mmol). **^{1}H NMR** (600 MHz, $CDCl_3$) δ 4.84 (t, *J* = 6.6 Hz, 1H), 3.41 (t, *J* = 6.6 Hz, 2H), 3.30 (dd, *J* = 21.6, 6.6 Hz, 2H), 1.92 – 1.84 (m, 2H), 1.70 – 1.62 (m, 1H), 1.61 – 1.50 (m, 3H), 1.45 (s, 9H), 1.31 (d, *J* = 21.6 Hz, 3H). **^{13}C NMR** (150 MHz, $CDCl_3$) δ 156.2, 97.1 (d, *J* = 168.9 Hz), 79.6, 48.0 (d, *J* = 22.0 Hz), 36.3 (d, *J* = 22.2 Hz), 33.4, 33.0, 28.5, 22.2 (d, *J* = 6.0 Hz), 21.7 (d, *J* = 24.2 Hz). **^{19}F NMR** (565 MHz, $CDCl_3$) δ -150.5 – -150.8 (m). **IR** ν_{max} (film): 2981, 2928, 2868, 1696, 1636, 1517, 1461, 1397, 1359, 1252, 1166, 754 cm^{-1}. **HRMS** (ESI) m/z calcd for $C_{12}H_{23}BrFNO_2Na$ $[M+Na]^+$: 334.0788; found: 334.0794.

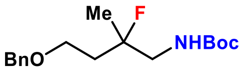

2an'

Product **2an'** was prepared following the general procedure B, except that the aminofluorination reaction was performed in DCE at 80 °C for 1 h. Purification using column chromatography (PE/EA = 20:1) afforded **2an'** as a yellow oil (54.1 mg, 0.17 mmol, 58%) with 10% of the starting materials **1an** recovered (5.3 mg, 0.03 mmol). **^{1}H NMR** (600 MHz, $CDCl_3$) δ 7.29 – 7.16 (m, 5H), 4.92 – 4.86 (m, 1H), 4.42 (s, 2H), 3.58 – 3.48 (m, 2H), 3.25 (dd, *J* = 19.8, 6.6 Hz, 2H), 1.98 – 1.81 (m, 2H), 1.37 (s, 9H), 1.27 (d, *J* = 22.2 Hz, 3H). **^{13}C NMR** (150 MHz, $CDCl_3$) δ 156.2, 138.2, 128.5, 127.8, 127.7, 96.4 (d, *J* = 169.2 Hz), 79.5, 73.3, 65.6 (d, *J* = 7.0 Hz), 48.0 (d, *J* = 24.2 Hz), 37.4 (d, *J* = 22.4 Hz), 28.5, 22.7 (d, *J* = 23.8 Hz). **^{19}F NMR** (565 MHz, $CDCl_3$) δ -149.5 – -149.8 (m). **IR** ν_{max} (film): 3245, 3030, 2981, 2784, 1749, 1690, 1507, 1403, 1377, 1366, 1163, 927, 854, 742, 701 cm^{-1}. **HRMS** (ESI) m/z calcd for $C_{17}H_{26}FNNaO_3$ $[M+Na]^+$: 334.1789; found: 334.1791.

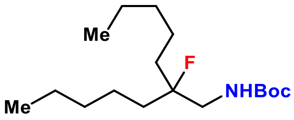

2ao'

Product **2ao'** was prepared following the general procedure B, except that the aminofluorination reaction was performed in DCE at 80 °C for 1 h. Purification using column chromatography (PE/EA = 20:1) afforded **2ao'** as a yellow oil (42.8 mg, 0.14 mmol, 47%) with 27% of the starting materials **1ao** recovered (13.6 mg, 0.08 mmol). **^{1}H**

NMR (600 MHz, $CDCl_3$) δ 4.75 (t, *J* = 6.6 Hz, 1H), 3.30 (dd, *J* = 22.2, 6.6 Hz, 2H), 1.62 – 1.55 (m, 4H), 1.45 (s, 9H), 1.35 – 1.24 (m, 12H), 0.89 (t, *J* = 6.6 Hz, 6H). **^{13}C NMR** (150 MHz, $CDCl_3$) δ 156.2, 99.4 (d, *J* = 169.5 Hz), 79.5, 46.5 (d, *J* = 21.7 Hz), 34.5 (d, *J* = 22.1 Hz), 32.3, 28.5, 23.0 (d, *J* = 6.5 Hz), 22.6, 14.1. **^{19}F NMR** (565 MHz, $CDCl_3$) δ -154.4 – -154.7 (m). **IR** ν_{max} (film): 3443, 2917, 2852, 1716, 1669, 1515, 1458, 1370, 1241, 1168 cm^{-1}. **HRMS** (ESI) m/z calcd for $C_{17}H_{35}FNO_2$ $[M+H]^+$: 304.2646; found: 304.2647.

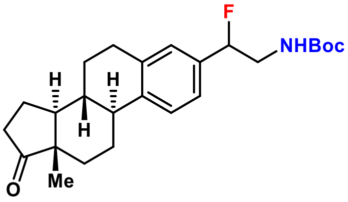

2ap', *dr* = 1:1

Product **2ap'** was prepared following the general procedure B. Purification using column chromatography (PE/EA = 4:1) afforded **2ap'** as a yellow oil (*dr* = 1:1, 82.6 mg, 0.20 mmol, 66%). **^{1}H NMR** (600 MHz, $CDCl_3$) δ 7.24 (d, *J* = 8.4 Hz, 1H), 7.06 (d, *J* = 8.4 Hz, 1H), 7.01 (s, 1H), 5.40 (dd, *J* = 48.0, 8.4 Hz, 1H), 4.90 (s, 1H), 3.66 – 3.49 (m, 1H), 3.35 – 3.20 (m, 1H), 2.88 – 2.81 (m, 2H), 2.43 (dd, *J* = 19.2, 9.0 Hz, 1H), 2.39 – 2.33 (m, 1H), 2.27 – 2.19 (m, 1H), 2.12 – 2.03 (m, 1H), 2.03 – 1.94 (m, 2H), 1.92 – 1.87 (m, 1H), 1.75 – 1.58 (m, 1H), 1.58 – 1.52 (m, 2H), 1.52 – 1.47 (m, 1H), 1.47 – 1.43 (m, 1H), 1.43– 1.41 (m, 1H), 1.38 (s, 9H), 0.84 (s, 3H). **^{13}C NMR** (150 MHz, $CDCl_3$) δ 220.8, 155.9, 140.5, 137.0, 134.9 (d, *J* = 19.5 Hz), 126.3 (d, *J* = 6.6 Hz), 126.3 (d, *J* = 6.8 Hz), 125.7, 123.1 (d, *J* = 5.8 Hz), 123.1 (d, *J* = 5.9 Hz), 93.4 (d, *J* = 172.5 Hz), 93.3 (d, *J* = 171.7 Hz), 79.8, 50.6, 48.1, 46.5 (d, *J* = 24.9 Hz), 44.5, 38.2 (d, *J* = 2.2 Hz), 35.9, 31.7, 29.5 (d, *J* = 7.6 Hz), 28.5, 26.5 (d, *J* = 3.2 Hz), 25.8 (d, *J* = 3.9 Hz), 21.7, 13.9. **^{19}F NMR** (565 MHz, $CDCl_3$) δ -182.8 (ddd, *J* = 48.6, 32.4, 16.8 Hz), -183.1 (ddd, *J* = 48.8, 32.5, 16.9 Hz). **IR** ν_{max} (film): 3425, 3316, 3017, 2918, 2350, 1715, 1510, 1456, 1365, 1272, 1249, 1161, 1000, 913, 740 cm^{-1}. **HRMS** (ESI) m/z calcd for $C_{25}H_{35}FNO_3$ $[M+H]^+$: 416.2595; found: 416.2596.

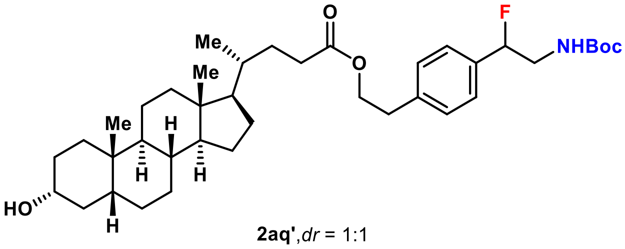

2aq',*dr* = 1:1

Product **2aq'** was prepared following the general procedure B. Purification using column chromatography (PE/EA = 2:1) afforded **2aq'** as a yellow oil (*dr* = 1:1, 123 mg, 0.19 mmol, 64%). **^{1}H NMR** (600 MHz, $CDCl_3$) δ 7.30 – 7.25 (m, 2H), 7.24 – 7.20 (m, 2H), 5.50 (dd, *J* = 48.0, 8.4 Hz, 1H), 5.11 (s, 1H), 4.28 – 4.24 (m, 2H), 3.72 – 3.54 (m, 2H), 3.41 – 3.28 (m, 1H), 2.92 (t, *J* = 7.2 Hz, 2H), 2.35 – 2.27 (m, 1H), 2.22 – 2.12 (m, 2H), 1.96 – 1.90 (m, 1H), 1.86 – 1.81 (m, 2H), 1.81 – 1.76 (m, 2H), 1.76 – 1.71 (m, 2H), 1.66 – 1.62 (m, 1H), 1.57 – 1.53 (m, 1H), 1.52 – 1.46 (m, 1H), 1.43 (s, 9H), 1.37 – 1.33 (m, 2H), 1.32 – 1.27 (m, 2H), 1.26 – 1.23 (m, 2H), 1.23 – 1.17(m, 2H), 1.17 – 1.11 (m, 2H), 1.08 – 1.05 (m, 2H), 1.05 – 1.00 (m, 2H),0.99 – 0.93 (m, 1H), 0.90 (s, 3H), 0.88 (d, *J* = 6.6 Hz, 3H), 0.62 (s,

3H). **^{13}C NMR** (150 MHz, $CDCl_3$) δ 174.2, 155.9, 138.5, 135.6 (d, J = 19.7 Hz), 129.1, 125.7 (d, J = 6.9 Hz), 93.1 (d, J = 172.3 Hz), 79.7, 71.7, 64.5, 56.5, 56.0, 46.4 (d, J = 24.1 Hz), 42.7, 42.1, 40.4, 40.2, 36.4, 35.9, 35.4, 35.3, 34.8, 34.6, 31.2, 31.0, 30.5, 28.4, 28.2, 27.2, 26.5, 24.2, 23.4, 20.8, 18.3, 12.1. **^{19}F NMR** (565 MHz, $CDCl_3$) δ -182.8 – -183.3 (m). **IR** ν_{max} (film): 3384, 3025, 2933, 2868, 2641, 2458, 2018, 1716, 1515, 1451, 1370, 1160, 1050, 800 cm^{-1}. **HRMS** (ESI) m/z calcd for $C_{39}H_{61}FNO_5$ $[M+H]^+$: 642.4528; found: 642.4520.

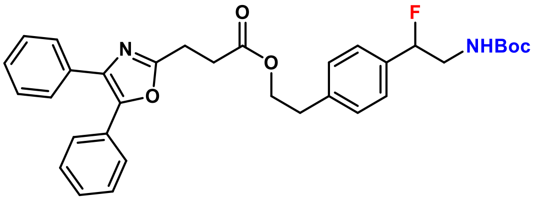

2ar'

Product **2ar'** was prepared following the general procedure B. Purification using column chromatography (PE/EA = 2:1) afforded **2ar'** as a yellow oil (105 mg, 0.19 mmol, 63%). **^{1}H NMR** (600 MHz, $CDCl_3$) δ 7.66 (d, J = 8.4 Hz, 2H), 7.59 (d, J = 8.4 Hz, 2H), 7.39 – 7.35 (m, 3H), 7.36 – 7.31 (m, 3H), 7.31 – 7.26 (m, 2H), 7.24 (d, J = 7.8 Hz, 2H), 5.51 (dd, J = 48.0, 8.4 Hz, 1H), 5.04 (s, 1H), 4.36 (t, J = 6.6 Hz, 2H), 3.75 – 3.57 (m, 1H), 3.43 – 3.33 (m, 1H), 3.19 (t, J = 7.2 Hz, 2H), 2.97 (t, J = 7.2 Hz, 2H), 2.92 (t, J = 7.8 Hz, 2H), 1.47 (s, 9H). **^{13}C NMR** (150 MHz, $CDCl_3$) δ 171.9, 161.8, 155.8, 145.5, 138.4, 135.7 (d, J = 19.8 Hz), 135.2, 132.5, 129.1, 129.0, 128.7, 125.8 (d, J = 6.9 Hz), 128.1, 128.0, 126.5, 125.8, 125.8, 93.1 (d, J = 172.3 Hz), 79.7, 65.1, 46.4 (d, J = 24.1 Hz), 34.8, 31.2, 28.4, 23.5. **^{19}F NMR** (565 MHz, $CDCl_3$) δ -183.1 (ddd, J = 48.2, 31.2, 16.9 Hz). **IR** ν_{max} (film): 3440, 3240, 2979, 2366, 1736, 1651, 1573, 1417, 1374, 1214, 1160, 898, 811, 736 cm^{-1}. **HRMS** (ESI) m/z calcd for $C_{33}H_{36}FN_2O_5$ $[M+H]^+$: 559.2603; found: 559.2601.

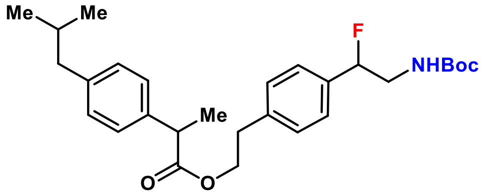

2as', two diastereomeric pairs in a 1:1 ratio

Product **2as'** was prepared following the general procedure B. Purification using column chromatography (PE/EA = 10:1) afforded **2as'** as a yellow oil (a mixture containing two diastereomeric pairs in a ratio of 1:1, 99.0 mg, 0.21 mmol, 70%). **^{1}H NMR** (600 MHz, $CDCl_3$) δ 7.25 (d, J = 7.8 Hz, 2H), 7.18 (d, J = 7.8 Hz, 2H), 7.14 (d, J = 7.8 Hz, 2H), 7.11 (d, J = 7.8 Hz, 2H), 5.52 (dd, J = 48.0, 8.4 Hz, 1H), 4.98 (s, 1H), 4.35 – 4.29 (m, 1H), 4.29 – 4.23 (m, 1H), 3.74 – 3.61 (m, 2H), 3.44 – 3.32 (m, 1H), 2.95 – 2.82 (m, 2H), 2.48 (d, J = 7.2 Hz, 2H), 1.92 – 1.84 (m, 1H), 1.49 (s, 3H), 1.47 (s, 9H), 0.93 (d, J = 6.6 Hz, 6H). **^{13}C NMR** (150 MHz, $CDCl_3$) δ 174.7, 155.9, 140.7, 138.7, 137.8, 135.6 (d, J = 19.7 Hz), 129.4, 129.2, 127.3, 125.8 (d, J = 6.9 Hz), 93.2 (d, J = 172.2 Hz), 79.8, 65.0, 46.5 (d, J = 23.9 Hz), 45.3, 45.2, 34.9, 30.3, 28.5, 22.5, 18.5. **^{19}F NMR** (565 MHz, $CDCl_3$) δ -183.1 (ddd, J = 49.1, 31.6, 17.2 Hz). **IR** ν_{max} (film): 3316, 3010, 2918, 2826, 1706, 1660, 1509, 1459, 1366, 1269, 1249, 1162, 901, 808, 737 cm^{-1}. **HRMS** (ESI)

m/z calcd for $C_{28}H_{39}FNO_4$ $[M+H]^+$: 472.2858; found: 472.2859.

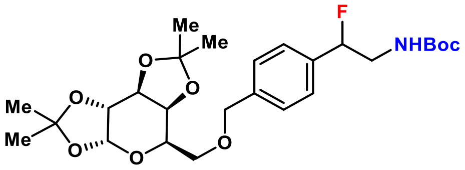

2at', *dr* = 1:1

Product **2at'** was prepared following the general procedure B. Purification using column chromatography (PE/EA = 4:1) afforded **2at'** as a yellow oil (*dr* = 1:1, 92.0 mg, 0.18 mmol, 60%). ^{1}**H NMR** (600 MHz, $CDCl_3$) δ 7.30 (d, *J* = 7.8 Hz, 2H), 7.27 – 7.21 (m, 2H), 5.55 – 5.33 (m, 2H), 4.90 (s, 1H), 4.58 – 4.50 (m, 2H), 4.48 (d, *J* = 12.6 Hz, 1H), 4.27 – 4.21 (m, 1H), 4.20 (d, *J* = 7.8 Hz, 1H), 3.94 (t, *J* = 6.0 Hz, 1H), 3.68 – 3.50 (m, 3H), 3.35 – 3.23 (m, 1H), 1.47 (s, 3H), 1.38 (s, 9H), 1.37 (s, 3H), 1.26 (s, 6H). ^{13}**C NMR** (150 MHz, $CDCl_3$) δ 155.9, 139.1, 136.7 (d, *J* = 19.7 Hz), 128.0, 125.6 (d, *J* = 7.0 Hz), 109.4, 108.7, 96.5, 93.2 (d, *J* = 172.4 Hz), 79.8, 73.0, 71.3, 70.8, 70.7, 69.2, 67.1, 46.5 (d, *J* = 24.0 Hz), 28.5, 28.4, 27.9, 26.2, 26.1, 25.0, 24.6. ^{19}**F NMR** (565 MHz, $CDCl_3$) δ -183.4 – -183.8 (m). **IR** ν_{max} (film): 3290, 3095, 2936, 2832, 1701, 1571, 1500, 1412, 1366, 1254, 1163, 1061, 910, 764, 733 cm^{-1}. **HRMS** (ESI) m/z calcd for $C_{26}H_{39}FNO_8$ $[M+H]^+$: 512.2654; found: 512.2657.

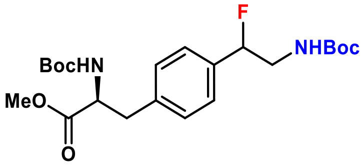

2au', *dr* = 1:1

Product **2au'** was prepared following the general procedure B. Purification using column chromatography (PE/EA = 4:1) afforded **2au'** as a yellow oil (*dr* = 1:1, 81.9 mg, 0.19 mmol, 62%). ^{1}**H NMR** (500 MHz, $CDCl_3$) δ 7.30 – 7.23 (m, 2H), 7.14 (d, *J* = 7.0 Hz, 2H), 5.49 (dd, *J* = 48.5, 8.5 Hz, 1H), 4.95 (s, 2H), 4.64 – 4.50 (m, 1H), 3.70 (s, 3H), 3.68 – 3.54 (m, 1H), 3.41 – 3.28 (m, 1H), 3.16 – 3.07 (m, 1H), 3.07 – 2.99 (m, 1H), 1.44 (s, 9H), 1.40 (s, 9H). ^{13}**C NMR** (150 MHz, $CDCl_3$) δ 172.3, 155.9, 155.2, 136.9, 136.2 (d, *J* = 20.2 Hz), 129.7, 125.9 (d, *J* = 6.9 Hz), 93.2 (d, *J* = 171.9 Hz), 80.2, 79.9, 54.5, 52.4, 46.5 (d, *J* = 24.2 Hz), 38.2, 28.5, 28.4. ^{19}**F NMR** (565 MHz, $CDCl_3$) δ -183.3 – -184.1 (m). **IR** ν_{max} (film): 3363, 3233, 3041, 2918, 2841, 1690, 1509, 1457, 1414, 1307, 1254, 1168, 1052, 966, 867, 741 cm^{-1}. **HRMS** (ESI) m/z calcd for $C_{22}H_{34}FN_2O_6$ $[M+H]^+$: 441.2395; found: 441.2401.

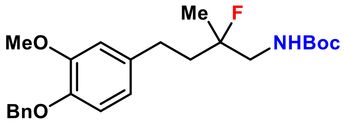

2av'

Product **2av'** was prepared following the general procedure B, except that the aminofluorination reaction was performed in DCE at 80 °C for 1 h. Purification using column chromatography (PE/EA = 10:1) afforded **2av'** as a

yellow oil (56.4 mg, 0.14 mmol, 45%). ^{1}**H NMR** (600 MHz, $CDCl_3$) δ 7.45 – 7.41 (m, 2H), 7.37 – 7.34 (m, 2H), 7.31 – 7.27 (m, 1H), 6.80 (d, *J* = 7.8 Hz, 1H), 6.73 (d, *J* = 2.4 Hz, 1H), 6.66 (dd, *J* = 7.8, 1.8 Hz, 1H), 5.12 (s, 2H), 4.82 (t, *J* = 6.6 Hz, 1H), 3.88 (s, 3H), 3.35 (dd, *J* = 22.2, 6.6 Hz, 2H), 2.64 (m, 2H), 1.97 – 1.82 (m, 2H), 1.45 (s, 9H), 1.36 (d, *J* = 22.2 Hz, 3H). ^{13}**C NMR** (150 MHz, $CDCl_3$) δ 156.3, 149.8, 146.6, 137.5, 135.0, 128.6, 127.9, 127.4, 120.2, 114.5, 112.4, 97.1 (d, *J* = 168.8 Hz), 79.7, 71.4, 56.1, 48.0 (d, *J* = 22.2 Hz), 39.4 (d, *J* = 22.2 Hz), 29.5 (d, *J* = 6.6 Hz), 28.5, 21.9 (d, *J* = 24.2 Hz). ^{19}**F NMR** (565 MHz, $CDCl_3$) δ -150.7 – -151.0 (m). **IR** ν_{max} (film): 3456, 3214, 3010, 2850, 1716, 1630, 1517, 1458, 1383, 1362, 1292, 1249, 1165, 1119, 917, 844 cm^{-1}. **HRMS** (ESI) m/z calcd for $C_{24}H_{33}FNO_4$ $[M+H]^+$: 418.2388; found: 418.2387.

Part 5: Procedure and characteristic data for derivatization

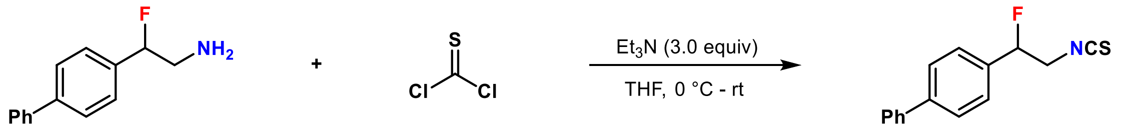

2a (1.0 equiv) **3**

To a solution of **2a** (43.1 mg, 0.20 mmol, 1.0 equiv) in dry THF (20 mL), carbon chlorosulfide (15 µL, 0.20 mmol, 1.0 equiv) and Et_3N (83 µL, 0.60 mmol, 3.0 equiv) were added at 0 °C. The mixture was stirred for 30 min at 0 °C, then allowed to warm to room temperature and stirred for another 30 min. Water (10 mL) was added to quench the reaction, followed by extraction with diethyl ether (20 mL × 3). The combined organic phases were dried over anhydrous Na_2SO_4, and the solvents were removed under reduced pressure. Purification of the crude product by flash column chromatography using PE as the eluent afforded the desired product **3** as a colorless oil (31.9 mg, 0.12 mmol, 62%). ^{1}**H NMR** (600 MHz, $CDCl_3$) δ 7.64 (d, *J* = 7.8 Hz, 2H), 7.59 (d, *J* = 7.2 Hz, 2H), 7.45 (t, *J* = 7.8 Hz, 2H), 7.42 (d, *J* = 7.8 Hz, 2H), 7.37 (t, *J* = 7.2 Hz, 1H), 5.67 (ddd, *J* = 46.8, 7.2, 3.6 Hz, 1H), 4.04 – 3.74 (m, 2H). ^{13}**C NMR** (150 MHz, $CDCl_3$) δ 142.5, 140.2, 134.8, 134.5 (d, *J* = 19.9 Hz), 128.9, 127.8, 127.6, 127.2, 126.1 (d, *J* = 7.0 Hz), 91.3 (d, *J* = 180.8 Hz), 50.4 (d, *J* = 27.1 Hz). ^{19}**F NMR** (565 MHz, $CDCl_3$) δ -178.9 – -184.3 (m). **IR** ν_{max} (film): 1627, 1586, 1489, 1381, 1354, 1267, 1220, 1046, 911, 745 cm^{-1}. **HRMS** (ESI) m/z calcd for $C_{15}H_{13}FNS$ $[M+H]^+$: 258.0747; found: 258.0743.

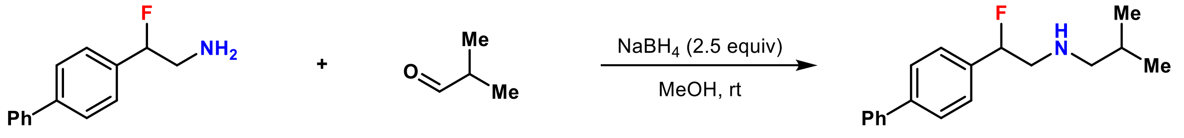

2a (1.2 equiv) **4**

In an oven-dried round bottomed flask, **2a** (43.1 mg, 0.20 mmol, 1.0 equiv) and 2-formylpropane (22 µL, 0.24

mmol, 1.2 equiv) were dissolved in anhydrous MeOH (2.0 mL) and the mixture was stirred for 12 h. Subsequently, $NaBH_4$ (18.9 mg, 0.50 mmol, 2.5 equiv) was added. After stirred for an additional 2 h, water (10 mL) was added to the mixture, which was then extracted with DCM (15 mL × 3). The combined organic layers were washed with brine (20 mL), dried over Na_2SO_4, filtered, and concentrated under reduced pressure. Purification by flash column chromatography using PE/EA = 1:2 as the eluent afforded product **4** as a colorless oil (45.0 mg, 0.17 mmol, 83%). **^{1}H NMR** (600 MHz, $CDCl_3$) δ 7.56 – 7.47 (m, 4H), 7.40 – 7.32 (m, 4H), 7.27 (t, *J* = 7.2 Hz, 1H), 5.57 (ddd, *J* = 48.0, 9.0, 3.0 Hz, 1H), 3.07 (ddd, *J* = 16.2, 13.2, 9.0 Hz, 1H), 2.84 (ddd, *J* = 32.4, 13.2, 3.0 Hz, 1H), 2.53 – 2.31 (m, 2H), 1.70 (dq, *J* = 13.2, 6.6 Hz, 1H), 0.85 (d, *J* = 6.6 Hz, 6H). **^{13}C NMR** (150 MHz, $CDCl_3$) δ 141.5, 140.6, 137.5 (d, *J* = 19.0 Hz), 128.8, 127.5, 127.3, 127.1, 126.1 (d, *J* = 7.0 Hz), 93.7 (d, *J* = 171.2 Hz), 57.8, 55.9 (d, *J* = 24.0 Hz), 28.4, 20.6. **^{19}F NMR** (565 MHz, $CDCl_3$) δ -178.3 – -184.5 (m). **IR** ν_{max} (film): 1629, 1592, 1488, 1384, 1357, 1285, 1097, 1000, 763, 726 cm^{-1}. **HRMS** (ESI) m/z calcd for $C_{18}H_{23}FN$ $[M+H]^+$: 272.1809; found: 272.1810.

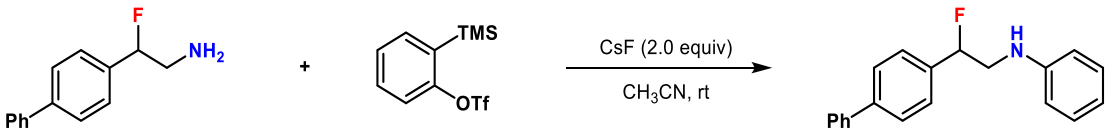

2a (1.5 equiv) **5**

To a solution of **2a** (64.6 mg, 0.30 mmol, 1.5 equiv) and silylaryl triflate (49 µL, 0.20 mmol, 1.0 equiv) in MeCN (4.0 mL) was added CsF (60.8 mg, 0.40 mmol, 2.0 equiv). The reaction mixture was allowed to stir at room temperature and monitored by TLC. Once the reaction was completed, the resulting solution was washed with brine (10 mL) and extracted with diethyl ether (15 mL × 3). The combined organic layers were dried over Na_2SO_4 and filtered. The solvent was removed under reduced pressure and the residue was purified by flash chromatography using eluents (PE/EA = 15:1) to afford the desired product **5** as a yellow oil (41.4 mg, 0.14 mmol, 71%). **^{1}H NMR** (500 MHz, $CDCl_3$) δ 7.63 (d, *J* = 8.0 Hz, 2H), 7.59 (d, *J* = 7.0 Hz, 2H), 7.49 – 7.42 (m, 4H), 7.37 (t, *J* = 7.5 Hz, 1H), 7.24 – 7.18 (m, 2H), 6.76 (t, *J* = 7.5 Hz, 1H), 6.68 (d, *J* = 8.0 Hz, 2H), 5.77 – 5.61 (m, 1H), 4.07 (s, 1H), 3.67 – 3.51 (m, 2H). **^{13}C NMR** (125 MHz, $CDCl_3$) δ 147.5, 142.0, 140.6, 136.8 (d, *J* = 19.6 Hz), 129.6, 129.0, 127.7, 127.6, 127.3, 126.3 (d, *J* = 6.4 Hz), 118.3, 113.4, 92.7 (d, *J* = 172.6 Hz), 50.1 (d, *J* = 25.0 Hz). **^{19}F NMR** (470 MHz, $CDCl_3$) δ -168.4 – -193.3 (m). **IR** ν_{max} (film): 2382, 1599, 1485, 1386, 1351, 1297, 1108, 1034, 766, 727 cm^{-1}. **HRMS** (ESI) m/z calcd for $C_{20}H_{19}FN$ $[M+H]^+$: 292.1496; found: 292.1493.

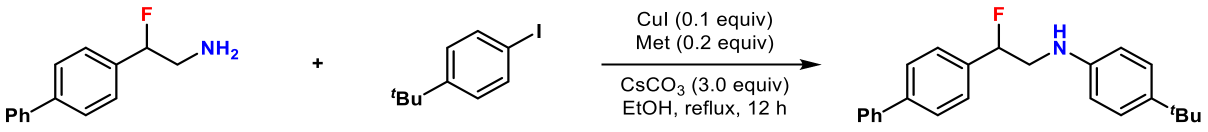

2a (3.0 equiv) **6**

A solution of the **2a** (43.1 mg, 0.20 mmol, 1.0 equiv), 1-tert-butyl-4-iodobenzene (106 µL, 0.60 mmol, 3.0 equiv), CuI (3.8 mg, 0.02 mmol, 0.1 equiv), metformin hydrochloride (6.6 mg, 0.04 mmol, 0.2 equiv) and Cs_2CO_3 (195 mg, 0.60 mmol, 3.0 equiv) in EtOH (2.0 mL) was heated to reflux under N_2 for12 h. After completion of the reaction, the mixture was cooled to room temperature, and the solid was removed by filter. The filtrate was concentrated under

reduced pressure and the crude product was purified by column chromatography using eluents (PE/EA = 15:1) to afford the corresponding product **6** as a yellow oil (43.0 mg, 0.12 mmol, 62%). **^{1}H NMR** (600 MHz, $CDCl_3$) δ 7.56 (d, J = 7.8 Hz, 2H), 7.54 – 7.51 (m, 2H), 7.40 – 7.35 (m, 4H), 7.32 – 7.28 (m, 1H), 7.17 (d, J = 8.4 Hz, 2H), 6.57 (d, J = 8.4 Hz, 2H), 5.70 – 5.53 (m, 1H), 3.52 (d, J = 6.0 Hz, 1H), 3.48 (dd, J = 6.0, 3.6 Hz, 1H), 1.22 (s, 9H). **^{13}C NMR** (150 MHz, $CDCl_3$) δ 145.0, 141.8, 141.1, 140.6, 136.8 (d, J = 19.9 Hz), 128.9, 127.6, 127.4, 127.2, 126.2, 126.1 (d, J = 6.1 Hz), 113.1, 92.6 (d, J = 172.5 Hz), 50.3 (d, J = 25.4 Hz), 33.9, 31.5. **^{19}F NMR** (565 MHz, $CDCl_3$) δ -182.4 (dd, J = 46.4, 24.1 Hz). **IR** ν_{max} (film): 1601, 1486, 1384, 1354, 1295, 1209, 1112, 997, 767, 734 cm^{-1}. **HRMS** (ESI) m/z calcd for $C_{24}H_{27}FN$ $[M+H]^+$: 348.2122; found: 348.2125.

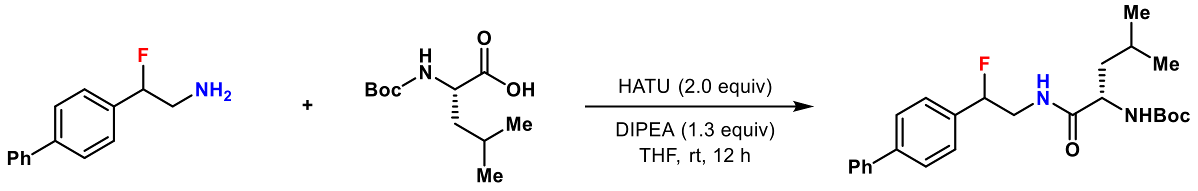

2a (1.2 equiv) **7**, *dr* = 1:1

To a solution of the Boc-*L*-leucine (55.5 mg, 0.24 mmol, 1.2 equiv) and *N*, *N*-diisopropylethylamine (DIPEA, 45 µL, 0.26 mmol, 1.3 equiv) in anhydrous THF (2.0 mL) was added **2a** (43.1 mg, 0.20 mmol, 1.0 equiv). Then, *O*-(7-azabenzotriazol-1-yl)-*N*, *N*, *N*', *N*'-tetramethyluronium hexafluorophosphate (HATU, 152 mg, 0.40 mmol, 2.0 equiv) was added to the reaction. The resulting mixture was stirred at room temperature overnight. Upon TLC showed complete consumption of the starting material, the mixture was filtered through celite. The filtrate was washed with saturated $NaHCO_3$ solution, dried over anhydrous Na_2SO_4, and concentrated in *vacuo*. Further purification by flash column chromatography using PE/EA = 8:1 as the eluent afforded the desired product **7** as a colorless oil (*dr* = 1:1, 68.6 mg, 0.16 mmol, 80%). **^{1}H NMR** (500 MHz, $CDCl_3$) δ 7.62 – 7.55 (m, 4H), 7.46 – 7.39 (m, 4H), 7.38 – 7.33 (m, 1H), 6.64 (s,1H), 5.66 – 5.48 (m, 1H), 4.90 (d, J = 8.5 Hz, 1H), 4.14 (s, 1H), 4.06 – 3.85 (m, 1H), 3.62 – 3.37 (m, 1H), 1.71 – 1.59 (m, 2H), 1.44 (s, 10H), 0.96 – 0.89 (m, 6H). **^{13}C NMR** (125 MHz, $CDCl_3$) δ 173.1, 155.8, 141.8, 141.7, 140.5, 136.1 (d, J = 19.3 Hz), 128.8, 127.6, 127.3, 127.1, 126.0 (d, J = 6.9), 125.9 (d, J = 6.9), 92.7 (d, J = 173.1 Hz), 93.3 (d, J = 173.1 Hz), 80.2, 53.2, 45.0 (d, J = 24.0 Hz), 41.4, 41.3, 28.3, 24.8, 24.7, 23.0, 22.0. **^{19}F NMR** (470 MHz, $CDCl_3$) δ -169.8 – -193.1 (m). **IR** ν_{max} (film): 1627, 1596, 1382, 1351, 1265, 1169, 11083, 847, 743, 720 cm^{-1}. **HRMS** (ESI) m/z calcd for $C_{25}H_{33}FN_2O_3Na$ $[M+Na]^+$: 451.2367; found: 451.2359.

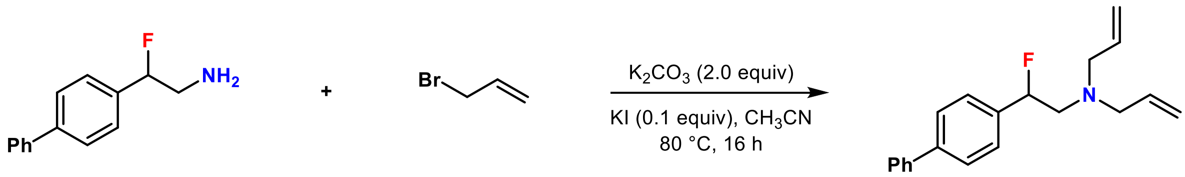

2a (3.0 equiv) **8**

To a mixture of K_2CO_3 (55.2 mg, 0.40 mmol, 2.0 equiv), KI (3.3 mg, 0.02 mmol, 0.1 equiv) and **2a** (43.1 mg, 0.20 mmol, 1.0 equiv) were added allyl bromide (52 µL, 0.60 mmol, 3.0 equiv) and CH_3CN (2.0 mL) sequentially. After being stirred at 80 °C (oil bath) for 16 h, the reaction was quenched with H_2O (10 mL) and extracted with ether (15

mL × 3). The combined organic layers were washed with brine, dried over anhydrous Na_2SO_4, and concentrated under reduced pressure. Purification of the crude product by flash column chromatography using PE/EA = 10:1 as the eluent afforded the desired product **8** as a colorless oil (46.0 mg, 0.16 mmol, 78%). **^{1}H NMR** (600 MHz, $CDCl_3$) δ 7.51 (dd, *J* = 7.8, 5.4 Hz, 4H), 7.36 (t, *J* = 7.2 Hz, 2H), 7.31 (d, *J* = 7.8 Hz, 2H), 7.28 (t, *J* = 7.2 Hz, 1H), 5.82 (ddd, *J* = 17.2, 10.2, 5.4 Hz, 2H), 5.61 (dd, *J* = 48.0, 7.8 Hz, 1H), 5.15 (d, *J* = 17.2 Hz, 2H), 5.11 (d, *J* = 10.2 Hz, 2H), 3.22 (d, *J* = 6.6 Hz, 4H), 2.97 (ddd, *J* = 16.2, 14.4, 8.4 Hz, 1H), 2.76 (ddd, *J* = 32.6, 14.4, 3.0 Hz, 1H). **^{13}C NMR** (150 MHz, $CDCl_3$) δ 141.3, 140.7, 137.8 (d, *J* = 20.4 Hz), 135.1, 128.8, 127.5, 127.2, 127.1, 126.0 (d, *J* = 7.0 Hz), 118.3, 93.0 (d, *J* = 172.9 Hz), 59.0 (d, *J* = 23.6 Hz), 57.6. **^{19}F NMR** (565 MHz, $CDCl_3$) δ -173.5 – -182.7 (m). **IR** ν_{max} (film): 1602, 1515, 1383, 1357, 1286, 1098, 996, 858, 782, 701cm^{-1}. **HRMS** (ESI) m/z calcd for $C_{20}H_{23}FN$ [M+H]$^+$: 296.1809; found: 296.1815.

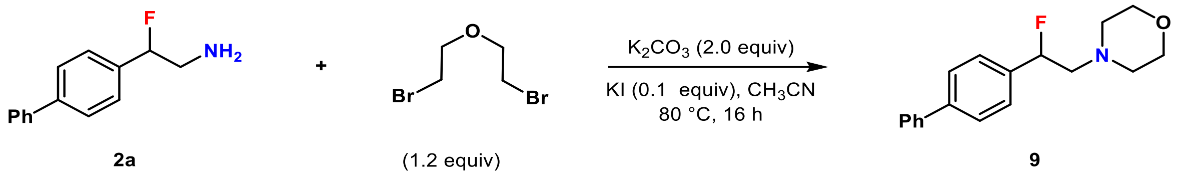

To a mixture of K_2CO_3 (55.2 mg, 0.40 mmol, 2.0 equiv), KI (3.3 mg, 0.02 mmol, 0.1 equiv) and **2a** (43.1 mg, 0.20 mmol, 1.0 equiv) were added 2,2'-dibromodiethyl ether (30 μL, 0.24 mmol, 1.2 equiv) and CH_3CN (2.0 mL) sequentially. After being stirred at 80 °C (oil bath) for 16 h, the reaction was quenched with H_2O (10 mL) and extracted with ether (15 mL × 3). The combined organic layers were washed with brine, dried over anhydrous Na_2SO_4, and concentrated under reduced pressure. Purification of the crude product by flash column chromatography using PE/EA = 4:1 as the eluent afforded the desired product **9** as a colorless oil (47.9 mg, 0.17 mmol, 84%). **^{1}H NMR** (500 MHz, $CDCl_3$) δ 7.63 – 7.55 (m, 4H), 7.48 – 7.39 (m, 4H), 7.36 (t, *J* = 7.5 Hz, 1H), 5.71 (ddd, *J* = 49.5, 8.5, 2.5 Hz, 1H), 3.77 (t, *J* = 4.5 Hz, 4H), 2.95 (ddd, *J* = 17.5, 14.5, 8.5 Hz, 1H), 2.75 – 2.65 (m, 1H), 2.68 – 2.56 (m, 4H). **^{13}C NMR** (125 MHz, $CDCl_3$) δ 141.6, 140.7, 137.8 (d, *J* = 19.9 Hz), 129.0, 127.7, 127.4, 127.2, 126.2 (d, *J* = 6.9 Hz), 92.6 (d, *J* = 173.6 Hz), 67.1, 65.0 (d, *J* = 23.1 Hz), 54.3. **^{19}F NMR** (470 MHz, $CDCl_3$) δ -176.5 (ddd, *J* = 49.5, 33.9, 17.1 Hz). **IR** ν_{max} (film): 1596, 1385, 1352, 1303, 1116, 1025, 1013, 872, 764, 702 cm^{-1}. **HRMS** (ESI) m/z calcd for $C_{18}H_{21}FNO$ [M+H]$^+$: 286.1602; found: 286.1610.

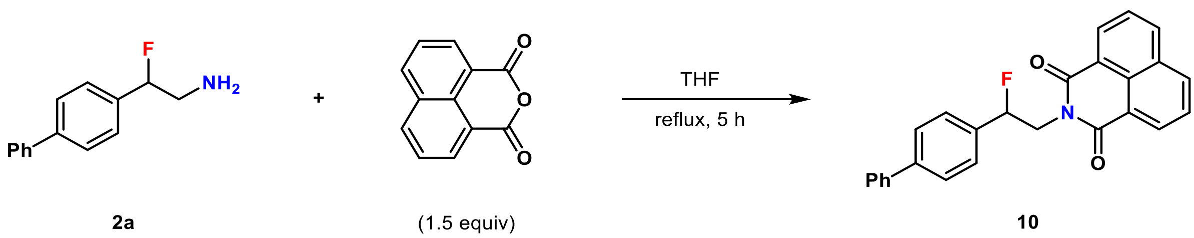

1,8-Naphthalic anhydride (59.5 mg, 0.30 mmol, 1.5 equiv) was added to a solution of **2a** (43.1 mg, 0.20 mmol, 1.0 equiv) in THF (2.0 mL). After being refluxed for 5 h, the reaction was quenched by water (10 mL) and extracted with EtOAc (15 mL × 3). The combined organic layers were washed with brine, dried over anhydrous Na_2SO_4, filtered, and concentrated in *vacuo*. Purification of the crude product by flash column chromatography using PE/EA

= 6:1 as the eluent afforded the desired product **10** as a colorless oil (44.3 mg, 0.11 mmol, 56%). **^{1}H NMR** (600 MHz, $CDCl_3$) δ 8.59 (d, *J* = 7.2 Hz, 2H), 8.18 (d, *J* = 8.4 Hz, 2H), 7.72 (t, *J* = 7.8 Hz, 2H), 7.58 (dd, *J* = 15.6, 7.8 Hz, 4H), 7.54 (d, *J* = 7.2 Hz, 2H), 7.38 (t, *J* = 7.8 Hz, 2H), 7.29 (t, *J* = 7.2 Hz, 1H), 5.90 (ddd, *J* = 49.2, 9.6, 3.0 Hz, 1H), 4.95 (ddd, *J* = 14.4, 12.0, 9.6 Hz, 1H), 4.25 (ddd, *J* = 34.2, 14.4, 3.0 Hz, 1H). **^{13}C NMR** (150 MHz, $CDCl_3$) δ 164.4, 141.8, 140.6, 136.5 (d, *J* = 19.1 Hz), 134.2, 131.7, 131.6, 128.8, 128.4, 127.5, 127.4, 127.2, 127.0, 126.3 (d, *J* = 6.9 Hz), 122.5, 91.6 (d, *J* = 175.9 Hz), 45.5 (d, *J* = 24.8 Hz). **^{19}F NMR** (565 MHz, $CDCl_3$) δ -179.3 – -190.1 (m). **IR** ν_{max} (film): 1657, 1599, 1519, 1386, 1355, 1292, 1039, 901, 834, 763 cm^{-1}. **HRMS** (ESI) m/z calcd for $C_{26}H_{19}FNO_2$ $[M+H]^+$: 396.1394; found: 396.1398.

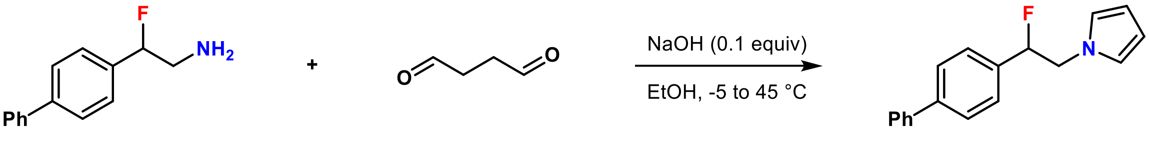

2a (1.1 equiv) **11**

NaOH (0.8 mg, 0.02 mmol, 0.1 equiv) was added to a solution of **2a** (43.1 mg, 0.20 mmol, 1.0 equiv) in EtOH (2.0 mL) at -5 °C. The mixture was stirred for 5 min and then butanedial (18 μL, 0.22 mmol 1.1 equiv) was added. The mixture was allowed to warm to ambient temperature slowly and the temperature was further raised to 45 °C. After 12 h, the reaction mixture was quenched by water (10 mL) and extracted with EtOAc (20 mL × 3). Combined organic layers were washed with brine, dried over anhydrous Na_2SO_4, filtered, and concentrated in *vacuo*. Purification of the crude product by flash column chromatography using PE/EA = 9:1 as the eluent afforded the desired product **11** as a colorless oil (43.5 mg, 0.16 mmol, 82%). **^{1}H NMR** (600 MHz, $CDCl_3$) δ 7.56 – 7.48 (m, 4H), 7.37 (t, *J* = 7.8 Hz, 2H), 7.29 (t, *J* = 7.2 Hz, 1H), 7.26 (d, *J* = 7.8 Hz, 2H), 6.60 (s, 2H), 6.10 (s, 2H), 5.59 (ddd, *J* = 47.4, 7.2, 3.6 Hz, 1H), 4.24 (ddd, *J* = 18.6, 15.0, 7.2 Hz, 1H), 4.16 (ddd, *J* = 26.2, 15.0, 3.6 Hz, 1H). **^{13}C NMR** (150 MHz, $CDCl_3$) δ 142.1, 140.5, 135.9 (d, *J* = 20.0 Hz), 129.0, 127.8, 127.5, 127.3, 126.2 (d, *J* = 7.0 Hz), 121.5, 108.8, 93.5 (d, *J* = 177.2 Hz), 55.4 (d, *J* = 25.9 Hz). **^{19}F NMR** (565 MHz, $CDCl_3$) δ -178.3 (ddd, *J* = 46.1, 26.4, 18.6 Hz). **IR** ν_{max} (film): 1686, 1592, 1384, 1356, 1280, 1118, 1107, 992, 766, 760 cm^{-1}. **HRMS** (ESI) m/z calcd for $C_{18}H_{17}FN$ $[M+H]^+$: 266.1340; found: 266.1339.

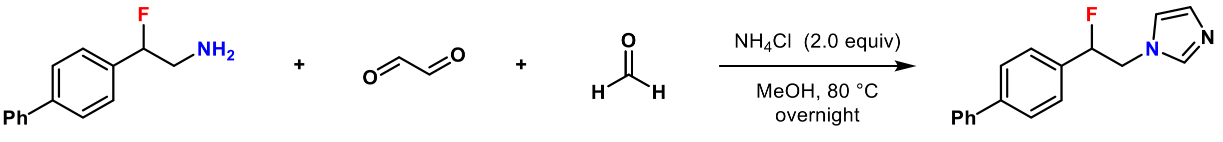

2a (2.0 equiv) (2.0 equiv) **12**

2a (43.1 mg, 0.20 mmol, 1.0 equiv), glyoxal (40% in water, 45 μL, 0.40 mmol, 2.0 equiv), formaldehyde (37% in water, 30 μL, 0.40 mmol, 2.0 equiv) and ammonium chloride (21.4 mg, 0.40 mmol, 2.0 equiv) was dissolved in MeOH (1.0 mL). The reaction was heated at 80 ºC in Radley tube overnight. After cooling to room temperature, 1 M NaOH (5.0 mL) was added and the product was extracted with DCM (3 × 10 mL). The combined organic layers were concentrated under reduced pressure. Purification of the crude product by flash column chromatography using PE/EA = 2:1 as the eluent afforded the desired product **12** as a colorless oil (39.4 mg, 0.15 mmol, 74%). **^{1}H NMR**

(600 MHz, $CDCl_3$) δ 7.63 – 7.56 (m, 4H), 7.50 – 7.41 (m, 3H), 7.37 (t, J = 7.8 Hz, 1H), 7.31 (d, J = 7.8 Hz, 2H), 7.07 (s, 1H), 6.94 (s, 1H), 5.68 (dt, J = 46.8, 5.4 Hz, 1H), 4.34 (dd, J = 22.8, 5.4 Hz, 2H). **^{13}C NMR** (150 MHz, $CDCl_3$) δ 142.4, 140.3, 137.9, 134.9 (d, J = 20.0 Hz), 129.7, 129.0, 127.9, 127.6, 127.2, 126.0 (d, J = 6.9 Hz), 119.9, 92.6 (d, J = 178.1 Hz), 52.5 (d, J = 26.5 Hz). **^{19}F NMR** (565 MHz, $CDCl_3$) δ -179.3 (dt, J = 46.8, 22.8 Hz). **IR** ν_{max} (film): 1659, 1487, 1393, 1248, 1206, 1141, 1126, 1046, 763, 698 cm^{-1}. **HRMS** (ESI) m/z calcd for $C_{17}H_{16}FN_2$ $[M+H]^+$: 267.1292; found: 267.1287.

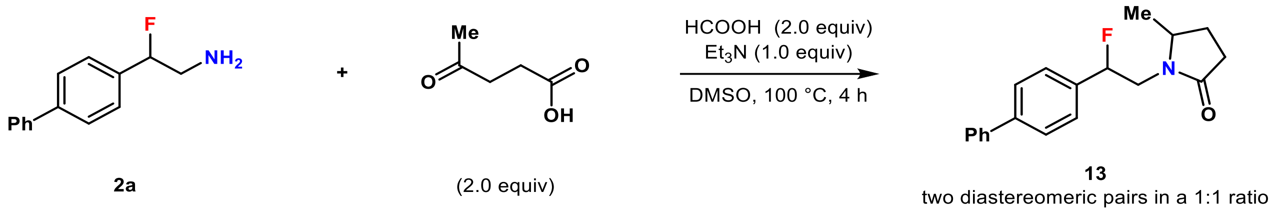

2a (2.0 equiv) 13
two diastereomeric pairs in a 1:1 ratio

2a (43.1 mg, 0.20 mmol, 1.0 equiv) and levulinic acid (41 μL, 0.40 mmol, 2.0 equiv) was dissolved in DMSO (1.0 mL). The mixture was degassed three times with argon, then formic acid (15 μL, 0.40 mmol, 2.0 equiv) and Et_3N (28 μL, 0.20 mmol, 1.0 equiv) were added into the reaction mixture. The mixture was allowed to stir for a further 4h at 100 °C and then cooled to ambient temperature. After that, the reaction mixture was quenched by 1 M NaOH (10 mL) and extracted with EtOAc (20 mL × 3). The combined organic layers were washed with brine, dried over anhydrous Na_2SO_4, filtered, and concentrated in *vacuo*. Purification of the crude product by flash column chromatography using PE/EA = 7:1 as the eluent afforded the desired product **13** as a colorless oil (a mixture containing two diastereomeric pairs in a ratio of 1:1, 42.2 mg, 0.14 mmol, 71%). **^{1}H NMR** (600 MHz, $CDCl_3$) δ 7.56 – 7.46 (m, 4H), 7.41 – 7.31 (m, 4H), 7.29 – 7.23 (m, 1H), 5.72 – 5.51 (m, 1H), 3.99 – 3.83 (m, 1.5H), 3.54 – 3.46 (m, 0.5H), 3.38 – 3.27 (m, 0.5H), 3.17 (td, J = 14.4, 9.6 Hz, 0.5H), 2.37 – 2.27 (m, 1.5H), 2.25 – 2.13 (m, 1H), 2.08 – 2.00 (m, 0.5H), 1.56 – 1.45 (m, 1H), 1.18 – 1.37 (m, 3H). **^{13}C NMR** (150 MHz, $CDCl_3$) δ 175.7 , 175.3 , 141.7 , 141.6 , 140.5 , 140.4 , 136.5 (d, J = 19.9 Hz), 136.4 (d, J = 19.4 Hz), 128.9 , 127.6 , 127.3 , 127.3 , 127.1 , 127.1 , 126.1 (d, J = 7.0 Hz), 125.9 (d, J = 7.1 Hz), 94.1 (d, J = 173.9 Hz), 90.9 (d, J = 176.2 Hz), 54.9 , 54.3 , 46.3 (d, J = 26.8 Hz), 46.1 (d, J = 22.7 Hz), 30.1 , 30.0 , 27.09 , 27.07, 19.9, 19.5. **^{19}F NMR** (565 MHz, $CDCl_3$) δ -181.0 (ddd, J = 46.2, 26.6, 17.9 Hz), -181.8 (ddd, J = 50.7, 38.1, 13.7 Hz). **IR** ν_{max} (film): 1718, 1666, 1546, 1472, 1286, 1183, 1112, 1094, 985, 763 cm^{-1}. **HRMS** (ESI) m/z calcd for $C_{19}H_{20}FNONa$ $[M+Na]^+$: 320.1421; found: 320.1419.

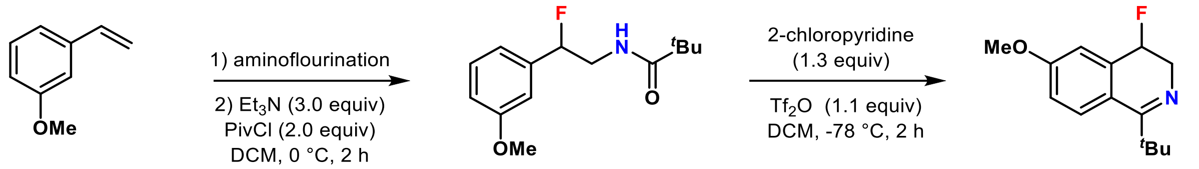

14-1 14

Hydroxylamine reagent 4-NO_2-BzONH$_3$OTf **V** (249 mg, 0.75 mmol, 2.5 equiv) was added to a plastic centrifuge tube charged with 3-methoxystyrene (42 μL, 0.30 mmol, 1.0 equiv), FePc (17.0 mg, 0.03 mmol, 0.1 equiv), $Et_3N·3HF$ (196 μL, 1.2 mmol, 4.0 equiv), and anhydrous CH_2Cl_2 (2.0 mL). The mixture was stirred in air at 30 °C (oil bath) for 30 min. Upon completion, the reaction was quenched with Et_3N (0.5 mL) at 0 °C. The reaction mixture was

washed through a short column chromatography (EtOAc) to afford the crude β-fluoroamine as yellow oil.

The β-fluoroamine was charged into a 10 mL RB flask containing DCM (2.0 mL) and Et_3N (125 µL, 0.90 mmol, 3.0 equiv). Pivaloyl chloride (74 µL, 0.60 mmol, 2.0 equiv) was added slowly at 0 °C, and the reaction was stirred for 2 h. After that, the reaction was quenched by 1M HCl (10 mL) and extracted with EtOAc (10 mL × 3). The combined organic layers were washed with brine, dried over anhydrous Na_2SO_4, filtered, and concentrated in *vacuo*. Purification of the crude product by flash column chromatography using PE/EA = 9:1 as the eluent afforded the desired product **14-1** as white solid (47.9 mg, 0.19 mmol, 63% over two steps). **^{1}H NMR** (600 MHz, $CDCl_3$) δ 7.3 (t, *J* = 7.8 Hz, 1H), 7.0 – 6.8 (m, 3H), 6.1 (s, 1H), 5.5 (dd, *J* = 48.6, 7.8 Hz, 1H), 3.9 – 3.8 (m, 1H), 3.8 (s, 3H), 3.5 – 3.3 (m, 1H), 1.2 (s, 9H). **^{13}C NMR** (150 MHz, $CDCl_3$) δ 178.8, 159.9, 139.0 (d, *J* = 19.7 Hz), 129.8, 117.7 (d, *J* = 7.2 Hz), 114.4, 110.9 (d, *J* = 7.9 Hz), 92.9 (d, *J* = 172.6 Hz), 55.4, 45.2 (d, *J* = 23.7 Hz), 38.8, 27.6. **^{19}F NMR** (565 MHz, $CDCl_3$) δ -184.2 (ddd, *J* = 48.6, 31.8, 17.9 Hz). **IR** ν_{max} (film): 2853, 1775, 1516, 1459, 1377, 1178, 1119, 1035, 834 cm^{-1}. cm^{-1}. **HRMS** (ESI) m/z calcd for $C_{14}H_{21}FNO_2$ $[M+H]^+$: 254.1551; found: 254.1546.

To a solution of product **14-1** (50.7 mg, 0.20 mmol, 1.0 equiv) in dry DCM (0.5 mL) was added 2-chloropyridine (24 µL, 0.26 mmol, 1.3 equiv) dropwise at -78 °C and stirred for 10 min. Then Tf_2O (37 µL, 0.22 mmol, 1.1 equiv) was added slowly, and the mixture was stirred at the same temperature for 5 min. The resulting mixture was allowed slowly warm up to room temperature and stirred for 2 h. Once completion, the mixture was treated with 1 M NaOH (3.0 mL) and extracted with DCM (10 mL × 3). The combined organic layers were washed with brine (10 mL), dried over Na_2SO_4 and concentrated *in vacuo*. Purification of the crude product by flash column chromatography using PE/EA = 20:1 as the eluent afforded the desired product **14** as a colorless oil (37.2 mg, 0.16 mmol, 79%). **^{1}H NMR** (600 MHz, $CDCl_3$) δ 7.85 (d, *J* = 8.4 Hz, 1H), 7.04 – 6.90 (m, 2H), 5.36 (dt, *J* = 51.0, 4.8 Hz, 1H), 4.25 (td, *J* = 16.2, 5.4 Hz, 1H), 3.87 (s, 3H), 3.68 (ddd, *J* = 37.2, 16.8, 4.2 Hz, 1H), 1.40 (s, 9H). **^{13}C NMR** (150 MHz, $CDCl_3$) δ 171.9, 160.6, 137.0 (d, *J* = 16.5 Hz), 129.2, 120.2, 114.6 (d, *J* = 3.4 Hz), 112.7 (d, *J* = 5.9 Hz), 86.0 (d, *J* = 173.7 Hz), 55.6, 51.9 (d, *J* = 22.3 Hz), 39.8, 30.2. **^{19}F NMR** (565 MHz, $CDCl_3$) δ -177.5 (ddd, *J* = 51.7, 37.2, 15.3 Hz). **IR** ν_{max} (film): 1666, 1592, 1382, 1351, 1285, 1224, 1110, 1097, 764, 712 cm^{-1}. **HRMS** (ESI) m/z calcd for $C_{14}H_{19}FNO$ $[M+H]^+$: 236.1445; found: 236.1449 .

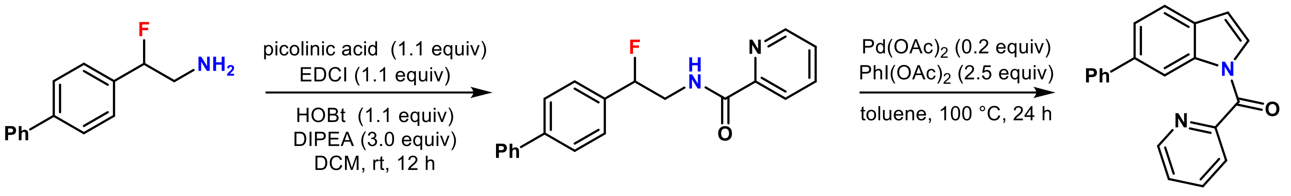

2a **15-1** **15**

A 10-mL flask fitted with a stirring bar was charged with a solution of **2a** (43.1 mg, 0.20 mmol, 1.0 equiv), *N*-(3-dimethylaminopropyl)-*N'*-ethylcarbodiimide hydrochloride (EDCI, 42.2 mg, 0.22 mmol, 1.1 equiv), 1-hydroxybenzotriazole (HOBt, 29.7 mg, 0.22 mmol, 1.1 equiv) and *N*, *N*-diisopropylethylamine (DIPEA, 105 µL, 0.60 mmol, 3.0 equiv) in dichloromethane (2.0 mL). Picolinic acid (21 µL, 0.22 mmol, 1.1 equiv) was then added at 0 °C, and the reaction mixture was stirred overnight at room temperature for 12 h. The resulting mixture was diluted with DCM (20 mL), and washed successively by 1 M HCl, saturated aqueous $NaHCO_3$, and brine. The organic layer

was dried over anhydrous Na_2SO_4 and evaporated in *vacuo*. Purification of the crude product by flash column chromatography using PE/EA = 2:1 as the eluent afforded the desired substrate **15-1** as a colorless oil (53.2 mg, 0.17 mmol, 83%). **^{1}H NMR** (600 MHz, $CDCl_3$) δ 8.58 (d, J = 4.8 Hz, 1H), 8.49 (t, J = 6.0 Hz, 1H), 8.21 (d, J = 7.8 Hz, 1H), 7.86 (td, J = 7.8, 1.8 Hz, 1H), 7.63 (d, J = 7.8 Hz, 2H), 7.61 – 7.57 (m, 2H), 7.49 (d, J = 7.8 Hz, 2H), 7.45 (dd, J = 8.4, 7.2 Hz, 3H), 7.36 (t, J = 7.2 Hz, 1H), 5.71 (ddd, J = 48.6, 8.4, 3.0 Hz, 1H), 4.15 (dddd, J = 31.8, 14.4, 7.8, 3.0 Hz, 1H), 3.73 (dddd, J = 16.8, 14.4, 9.0, 4.8 Hz, 1H). **^{13}C NMR** (150 MHz, $CDCl_3$) δ 164.6, 149.6, 148.2, 141.8, 140.5, 137.4, 136.3 (d, J = 19.8 Hz), 128.8, 127.6, 127.4, 127.2, 126.4, 126.1 (d, J = 6.2 Hz), 122.3, 92.9 (d, J = 172.8 Hz), 45.2 (d, J = 24.7 Hz). **^{19}F NMR** (565 MHz, $CDCl_3$) δ -182.5 (ddd, J = 48.3, 32.2, 16.6 Hz). **IR** ν_{max} (film): 2928, 1739, 1598, 1455, 1345, 1291, 1246, 1163, 1157, 1118, 868, 814 cm^{-1}. **HRMS** (ESI) m/z calcd for $C_{20}H_{18}FN_2O$ $[M+H]^+$: 321.1398; found: 321.1394.

To a Schlenk tube equipped with magnetic stir bar were added $Pd(OAc)_2$ (9.0 mg, 0.04 mmol, 0.2 equiv) and $PhI(OAc)_2$ (161 mg, 0.50 mmol, 2.5 equiv). After that, **15-1** (64.1 mg, 0.20 mmol, 1.0 equiv) in toluene (2.0 mL) was added into the mixture. The mixture was degass three times with argon and then stirred at 100 °C for 24 h. After cooling to room temperature, the reaction mixture was direct purified by flash column chromatography (PE/EA = 3:1 as eluent) to afford β-fluoroamine **15** as a colorless oil (32.2 mg, 0.11 mmol, 54%). **^{1}H NMR** (600 MHz, $CDCl_3$) δ 8.83 (s, 1H), 8.71 (d, J = 4.8 Hz, 1H), 8.07 (d, J = 7.8 Hz, 1H), 8.02 (d, J = 3.6 Hz, 1H), 7.88 (t, J = 7.8 Hz, 1H), 7.71 (d, J = 7.2 Hz, 2H), 7.62 (d, J = 7.8 Hz, 1H), 7.57 (dd, J = 7.8, 1.8 Hz, 1H), 7.50 – 7.42 (m, 3H), 7.34 (t, J = 7.2 Hz, 1H), 6.63 (d, J = 3.6 Hz, 1H). **^{13}C NMR** (150 MHz, $CDCl_3$) δ 165.8, 152.4, 148.6, 141.8, 138.5, 137.5, 137.2, 130.0, 129.1, 128.8, 127.6, 127.1, 126.2, 125.9, 123.8, 121.0, 115.7, 108.9. **IR** ν_{max} (film): 1593, 1538, 1381, 1354, 1253, 1104, 1006, 999, 866, 776 cm^{-1}. **HRMS** (ESI) m/z calcd for $C_{20}H_{15}N_2O$ $[M+H]^+$: 299.1179; found: 299.1173.

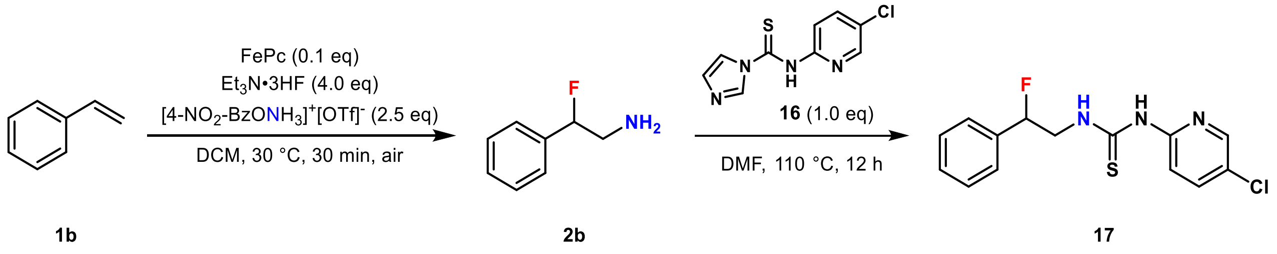

Hydroxylamine reagent 4-NO_2-BzONH_3OTf (**V**) (249 mg, 0.75 mmol, 2.5 equiv) was added to a plastic centrifuge tube charged with **1b** (23 μL, 0.30 mmol, 1.0 equiv), FePc (17.0 mg, 0.03 mmol, 0.1 equiv), $Et_3N\cdot 3HF$ (196 μL, 1.2 mmol, 4.0 equiv), and anhydrous CH_2Cl_2 (2.0 mL). The mixture was stirred in air at 30 °C (oil bath) for 30 min. Upon completion, the reaction was quenched with Et_3N (0.5 mL) at 0 °C. The reaction mixture was washed through a short column chromatography (EtOAc) to afford the crude β-fluoroamine **2b**.

A 10-mL flask fitted with a stirring bar was charged with crude β-fluoroamine **2b**, **16** (71.6 mg, 0.30 mmol, 1.0 equiv), and DMF (2.0 mL). The mixture was then stirred at 110 °C for 12 h. After which time, the reaction was quenched by addition of water (10 mL) and extracted with EtOAc (15 mL × 3). The combined organic layers were washed with brine (10 mL), dried over Na_2SO_4 and concentrated *in vacuo*. Purification of the crude product by flash column chromatography using PE/EA = 4:1 as the eluent afforded the desired product **17** as a white solid (61.3 mg,

0.20 mmol, 66% over two steps). **^{1}H NMR** (600 MHz, $CDCl_3$) δ 11.69 (t, J = 5.4 Hz, 1H), 9.30 (s, 1H), 8.11 (d, J = 2.4 Hz, 1H), 7.61 (dd, J = 8.4, 2.4 Hz, 1H), 7.48 – 7.40 (m, 4H), 7.40 – 7.36 (m, 1H), 6.88 (d, J = 9.0 Hz, 1H), 5.84 (ddd, J = 48.6, 8.4, 3.0 Hz, 1H), 4.48 (dddd, J = 31.8, 14.4, 6.6, 3.0 Hz, 1H), 3.93 (dddd, J = 18.6, 14.4, 8.4, 4.2 Hz, 1H). **^{13}C NMR** (150 MHz, $CDCl_3$) δ 180.3, 151.4, 144.6, 138.9, 137.2 (d, J = 18.9 Hz), 129.0, 128.8, 125.7, 125.6 (d, J = 7.1 Hz), 113.2, 92.2 (d, J = 174.1 Hz), 51.4 (d, J = 23.1 Hz). **^{19}F NMR** (565 MHz, $CDCl_3$) δ -181.5 – -186.4 (m). **IR** ν_{max} (film): 1678, 1616, 1416, 1350, 1277, 1185, 1039, 962, 846, 765 cm^{-1}. **HRMS** (ESI) m/z calcd for $C_{14}H_{14}ClFN_3S$ $[M+H]^+$: 310.0576; found: 310.0573.

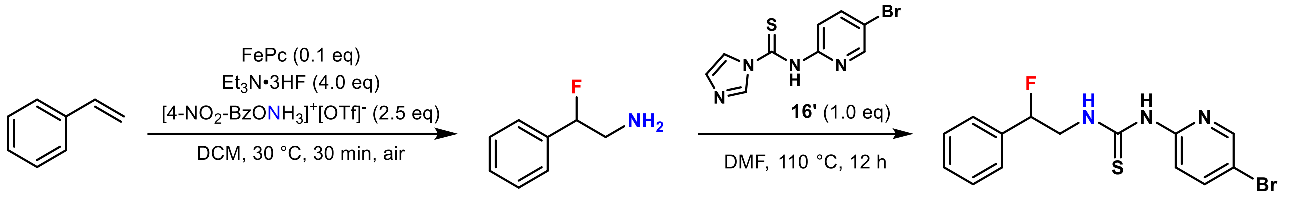

1b **2b** **18**

Product **18** was prepared following above method for the synthesis of compound **17**, except that **16'** (84.9 mg, 0.30 mmol, 1.0 equiv) was used to replace **16**. Purification using column chromatography (PE/EA = 4:1) afforded **18** as a yellow oil (71.2 mg, 0.20 mmol, 67% over two steps). **^{1}H NMR** (600 MHz, $CDCl_3$) δ 11.69 (t, J = 5.4 Hz, 1H), 9.34 (s, 1H), 8.20 (d, J = 2.4 Hz, 1H), 7.73 (dd, J = 9.0, 2.4 Hz, 1H), 7.55 – 7.40 (m, 4H), 7.40 – 7.35 (m, 1H), 6.84 (d, J = 9.0 Hz, 1H), 5.84 (ddd, J = 48.6, 8.4, 3.0 Hz, 1H), 4.48 (dddd, J = 31.8, 14.4, 6.6, 3.0 Hz, 1H), 3.92 (dddd, J = 18.6, 14.4, 8.4, 4.2 Hz, 1H). **^{13}C NMR** (150 MHz, $CDCl_3$) δ 180.2, 151.8, 146.8, 141.5, 137.3 (d, J = 18.9 Hz), 129.0, 128.8, 125.6 (d, J = 7.1 Hz), 113.7, 113.3, 92.2 (d, J = 173.8 Hz), 51.4 (d, J = 23.4 Hz). **^{19}F NMR** (565 MHz, $CDCl_3$) δ 183.8 – -184.0 (m). **IR** ν_{max} (film): 1700, 1685, 1532, 1467, 1254, 1197, 1128, 1085, 975, 842 cm^{-1}. **HRMS** (ESI) m/z calcd for $C_{14}H_{14}BrFN_3S$ $[M+H]^+$: 354.0070; found: 354.0076.

The procedure and characteristic data for the synthesis of LY503430

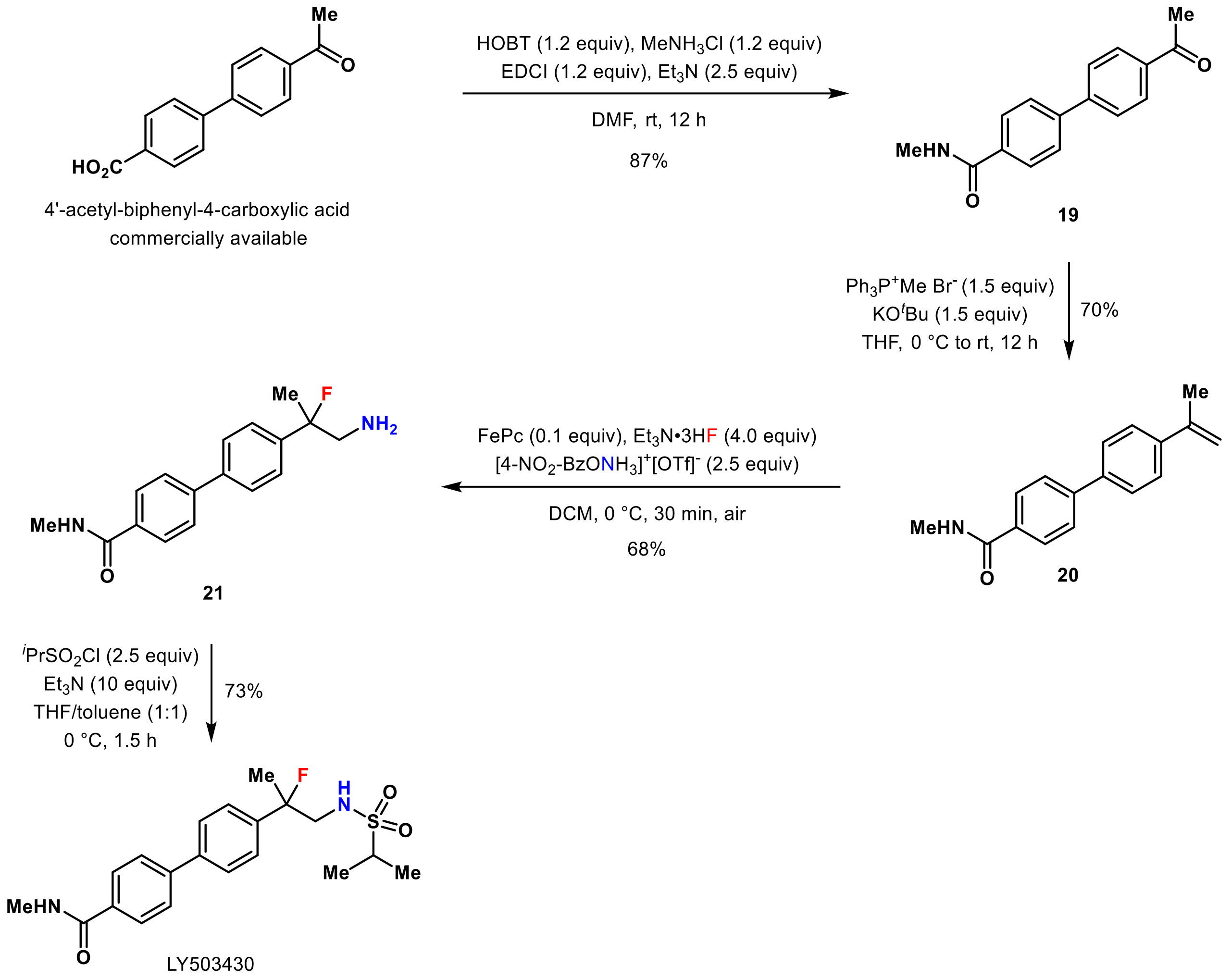

The commercially available acid (481 mg, 2.0 mmol, 1.0 equiv) was dissolved in DMF (20 mL). HOBt (324 mg, 2.4 mmol, 1.2 equiv), EDCI (460 mg, 2.4 mmol, 1.2 equiv), $MeNH_3Cl$ (162 mg, 2.4 mmol, 1.2 equiv), and Et_3N (695 µL, 5.0 mmol, 2.5 equiv) were added. After 12 h at room temperature, a saturated solution of NH_4Cl (15 mL) was added. The mixture was extracted with CH_2Cl_2 (30 mL × 3). The combined organic layers were washed with brine, dried over $MgSO_4$, filtered and concentrated under reduced pressure. Purification of the residue by flash chromatography on silica gel (PE/EA = 1:1 as eluent) afforded compound **19** (440 mg, 1.7 mmol, 87%) as a white solid. **^{1}H NMR** (600 MHz, $CDCl_3$) δ 8.05 (d, J = 8.4 Hz, 2H), 7.87 (d, J = 8.4 Hz, 2H), 7.72 – 7.67 (m, 4H), 6.26 (d, J = 4.8 Hz, 1H), 3.05 (d, J = 4.8 Hz, 3H), 2.65 (s, 3H). **^{13}C NMR** (150 MHz, $CDCl_3$) δ 197.8, 167.8, 144.7, 142.9, 136.5, 134.3, 129.1, 127.7, 127.6, 127.5, 27.0, 26.8. **IR** ν_{max} (film): 3316, 3034, 2876, 1740, 1716, 1643, 1490, 1248, 1160, 1040, 967, 830, 765 cm^{-1}. **HRMS** (ESI) m/z calcd for $C_{16}H_{15}NO_2Na$ $[M+Na]^+$: 276.0995; found: 276.0999.

To a suspension of methyltriphenylphosphonium bromide (932 mg, 2.6 mmol, 1.5 equiv) in THF (17 mL) was added KO^{t}Bu (293 mg, 2.6 mmol, 1.5 equiv) at 0 °C. After 1 h, compound **19** (440 mg, 1.7 mmol, 1.0 equiv) was added to the mixture at 0 °C. The mixture was allowed to slowly warm up to ambient temperature and stirred for 12 h. The reaction was quenched with a saturated aqueous solution of $NaHCO_3$. The layers were separated and the

aqueous phase was extracted with EtOAc (30 mL × 3). The combined organic phases were dried over anhydrous Na_2SO_4 and the solvents were removed under reduced pressure. Purification of the crude product by flash column chromatography (PE/EA = 2:1 as eluent) afforded the desired alkene compound **20** as a white solid (306 mg, 1.22 mmol, 70%). **^{1}H NMR** (600 MHz, $CDCl_3$) δ 7.83 (d, *J* = 7.8 Hz, 2H), 7.65 (d, *J* = 7.8 Hz, 2H), 7.60 – 7.53 (m, 4H), 6.23 (s, 1H), 5.44 (s, 1H), 5.14 (s, 1H), 3.04 (d, *J* = 4.8 Hz, 3H), 2.19 (s, 3H). **^{13}C NMR** (150 MHz, $CDCl_3$) δ 168.1, 143.8, 142.8, 141.0, 139.1, 133.4, 127.5, 127.2, 127.1, 126.2, 113.0, 27.0, 21.9. **IR** ν_{max} (film): 3311, 3053, 2917, 1719, 1643, 1555, 1426, 1321, 1168, 1112, 964, 831, 726 cm^{-1}. **HRMS** (ESI) m/z calcd for $C_{17}H_{17}NONa$ $[M+Na]^+$: 274.1202; found: 274.1207.

Hydroxylamine reagent 4-NO_2-$BzONH_3OTf$ (**V**) (249 mg, 0.75 mmol, 2.5 equiv) was added to a plastic centrifuge tube charged with alkene compound **20** (75.3 mg, 0.30 mmol, 1.0 equiv), FePc (17.0 mg, 0.03 mmol, 0.1 equiv), $Et_3N\cdot 3HF$ (196 μL, 1.2 mmol, 4.0 equiv), and anhydrous CH_2Cl_2 (2.0 mL). The mixture was stirred under air at 30 °C (oil bath) for 30 min. Upon completion, the reaction was quenched with Et_3N (0.5 mL) at 0 °C. The mixture was direct purified by flash column chromatography (PE/EA/Et_3N = 50:100:1) to afford β-fluoroamine **21** as a white solid (58.4 mg, 0.20 mmol, 68%). **^{1}H NMR** (600 MHz, $CDCl_3$) δ 7.84 (d, *J* = 8.4 Hz, 2H), 7.66 – 7.59 (m, 4H), 7.42 (d, *J* = 8.4 Hz, 2H), 6.39 (d, *J* = 5.4 Hz, 1H), 3.09 (s, 1H), 3.06 (d, *J* = 4.2 Hz, 1H), 3.04 (d, *J* = 5.4 Hz, 3H), 1.68 (d, *J* = 22.2 Hz, 3H). **^{13}C NMR** (150 MHz, $CDCl_3$) δ 168.0, 143.6, 142.6 (d, *J* = 22.3 Hz), 139.3, 133.6, 127.6, 127.3, 127.2, 125.2 (d, *J* = 9.7 Hz), 98.2 (d, *J* = 172.8 Hz), 52.3 (d, *J* = 24.8 Hz), 27.0, 25.1 (d, *J* = 24.8 Hz). **^{19}F NMR** (565 MHz, $CDCl_3$) δ -156.4 – -157.9 (m). **IR** ν_{max} (film): 3335, 3056, 2949, 2924, 2844, 1716, 1643, 1547, 1402, 1289, 1160, 864, 754, 693 cm^{-1}. **HRMS** (ESI) m/z calcd for $C_{17}H_{19}FN_2ONa$ $[M+Na]^+$: 309.1374; found: 309.1379.

To a solution of **21** (143 mg, 0.50 mmol, 1.0 equiv) in mixed toluene (2.5 mL) and THF (2.5 mL) were added Et_3N (695 μL, 5.0 mmol, 10 equiv) and ^{i}PrSO_2Cl (140 μL, 1.3 mmol, 2.5 equiv). After 1.5 h at 0 °C, the reaction was quenched with 1 M HCl and the mixture was extracted with CH_2Cl_2 (10 mL × 3). The combined organic phases dried over anhydrous Na_2SO_4 and evaporated in *vacuo*. Purification of the crude product by flash column chromatography (PE/EA = 1:2 as eluent) afforded the desired product LY503430 (107 mg, 0.37 mmol, 73%) as a white solid. **^{1}H NMR** (600 MHz, $CDCl_3$) δ 7.85 (d, *J* = 8.4 Hz, 2H), 7.67 – 7.60 (m, 4H), 7.43 (d, *J* = 8.4 Hz, 2H), 6.34 – 6.22 (m, 1H), 4.38 (t, *J* = 6.0 Hz, 1H), 3.66 – 3.52 (m, 2H), 3.09 – 3.01 (m, 4H), 1.76 (d, *J* = 22.8 Hz, 3H), 1.31 (d, *J* = 6.6 Hz, 3H), 1.28 (d, *J* = 6.6 Hz, 3H). **^{13}C NMR** (150 MHz, $CDCl_3$) δ 168.0, 143.3, 141.3 (d, *J* = 21.8 Hz), 140.0, 133.7, 127.6, 127.5, 127.3, 125.0 (d, *J* = 9.4 Hz), 97.0 (d, *J* = 175.5 Hz), 54.1, 52.7 (d, *J* = 23.6 Hz), 27.0, 24.9 (d, *J* = 24.3 Hz), 16.7, 16.6. **^{19}F NMR** (565 MHz, $CDCl_3$) δ -153.7 – -154.0 (m). **IR** ν_{max} (film): 3532, 3311, 3053, 2917, 2841, 1643, 1555, 1426, 1321, 1168, 1112, 908, 846, 726 cm^{-1}. **HRMS** (ESI) m/z calcd for $C_{20}H_{25}FN_2O_3NaS$ $[M+Na]^+$: 415.1462; found: 415.1462.

Part 6: Procedure and characterististic data for control experiments

Radical trapping experiment:

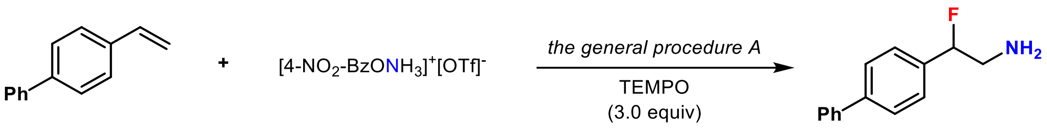

1a **V** **2a**, 0%

When 2,2,6,6-tetramethylpiperidinooxy (TMPEO, 141 mg, 0.90 mmol, 3.0 equiv) was added into the reaction of **1a** and **V** following the general procedure A, the aminofluorination of alkene was completely suppressed and the desired product **2a** was not detected.

Radical clock experiment:

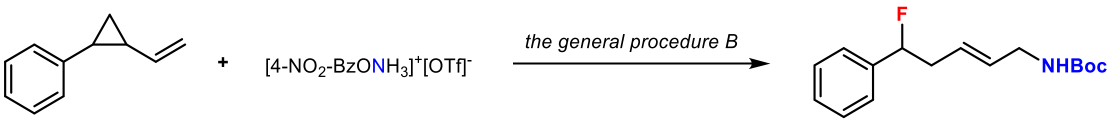

22 **V** **23'**, 65%

When cyclopropylvinyl compound **22** (43.3 mg, 0.30 mmol, 1.0 equiv) was treated with hydroxylamine reagent **V** following the general procedure B, compound **23'** was obtained by column chromatography (PE/EA = 20:1) as a yellow oil (54.5 mg, 0.20 mmol, 65%, *E:Z* > 20:1). **^{1}H NMR** (600 MHz, $CDCl_3$) δ 7.40 – 7.34 (m, 2H), 7.35 – 7.29 (m, 3H), 5.63 – 5.53 (m, 2H), 5.50 – 5.36 (m, 1H), 4.55 (s, 1H), 3.73 – 3.64 (m, 2H), 2.75 – 2.50 (m, 1H), 2.75 – 2.50 (m, 1H), 1.44 (s, 9H). **^{13}C NMR** (150 MHz, $CDCl_3$) δ 155.8, 139.8 (d, J = 19.8 Hz), 130.6, 128.6, 128.5 (d, J = 2.0 Hz), 126.6 (d, J = 5.1 Hz), 125.7 (d, J = 6.8 Hz), 93.9 (d, J = 172.9 Hz), 79.4, 42.4, 40.1 (d, J = 24.3 Hz), 28.5. **^{19}F NMR** (565 MHz, $CDCl_3$) δ -172.2 – -175.8 (m). **IR** ν_{max} (film): 3119, 3023, 2867, 1901, 1742, 1679, 1357, 1219, 1164, 978, 745 cm^{-1}. **HRMS** (ESI) m/z calcd for $C_{16}H_{22}FNO_2Na$ [M+Na]$^+$: 302.1527; found: 302.1525.

Competing reactions:

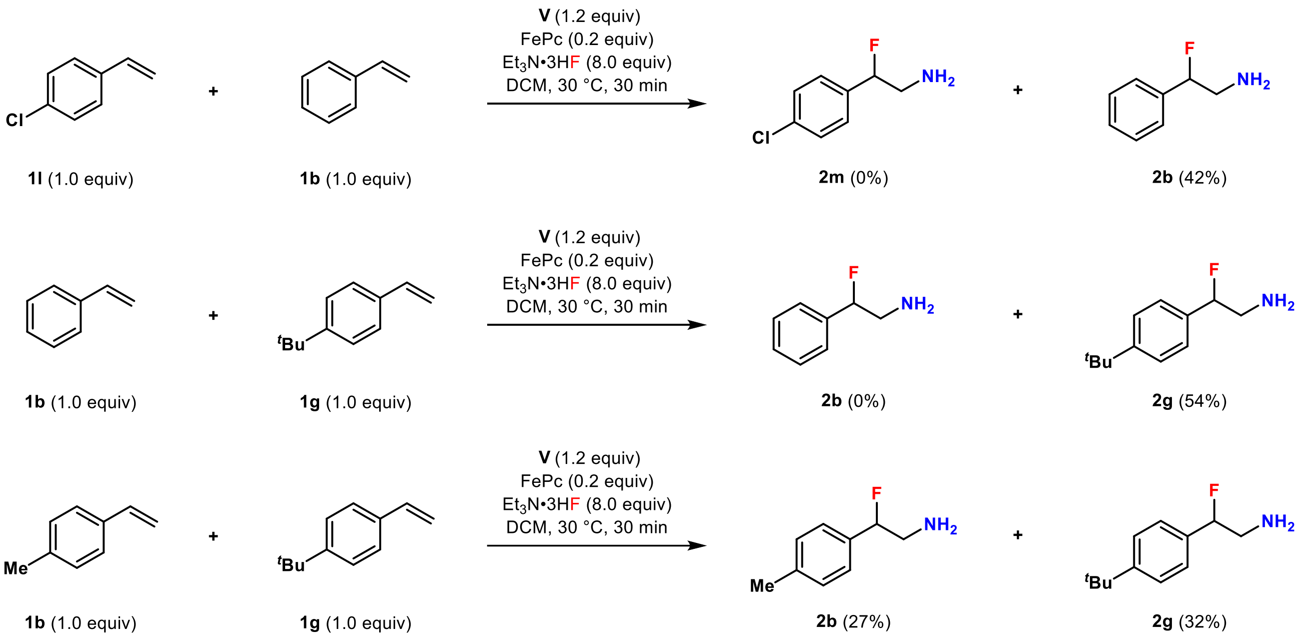

Hydroxylamine reagent 4-NO$_2$-BzONH$_3$OTf (**V**) (59.7 mg, 0.18 mmol, 1.2 equiv) was added to a plastic centrifuge tube charged with **alkene 1** (0.15 mmol, 1.0 equiv), **alkene 2** (0.15 mmol, 1.0 equiv), FePc (17.0 mg, 0.03 mmol, 0.2 equiv), Et$_3$N·3HF (196 μL, 1.2 mmol, 8.0 equiv), and anhydrous CH$_2$Cl$_2$ (2.0 mL). The mixture was stirred under air at 30 °C (oil bath) for 30 min. Upon completion, direct purification by flash column chromatography (PE/EA = 20:1 as eluent) afforded above results.

Trapping the aziridinium intermediate:

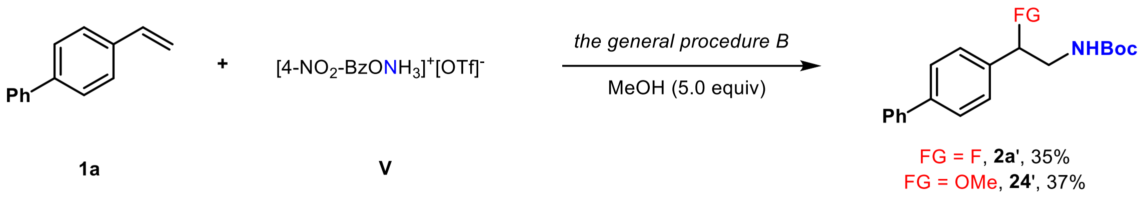

MeOH (61 μL, 1.5 mmol, 5.0 equiv) was added into the reaction of **1a** (54.1 mg, 0.30 mmol, 1.0 equiv) and 4-NO$_2$-BzONH$_3$OTf (**V**) (249 mg, 0.75 mmol, 2.5 equiv) following the general procedure B. Purification using column chromatography (PE/EA = 15:1) afforded **2a'** (33.1 mg, 0.11 mmol, 35%) and **24'** as a yellow oil (36.3 mg, 0.11 mmol, 37%). **11'**: **^{1}H NMR** (600 MHz, CDCl$_3$) δ 7.51 (d, J = 7.8 Hz, 4H), 7.36 (t, J = 7.8 Hz, 2H), 7.30 (d, J = 7.8 Hz, 2H), 7.26 (t, J = 7.8 Hz, 1H), 4.91 (s, 1H), 4.28 – 4.15 (m, 1H), 3.46 – 3.34 (m, 1H), 3.22 (s, 3H), 3.15 (ddd, J = 13.8, 8.4, 4.2 Hz, 1H), 1.37 (s, 9H). **^{13}C NMR** (150 MHz, CDCl$_3$) δ 156.0, 141.1, 140.9, 138.4, 128.9, 127.5, 127.4,

127.3, 127.2, 82.6, 79.4, 57.1, 47.0, 28.6. **IR** ν_{max} (film): 3349, 3214, 3011, 2867, 1722, 1667, 1512, 1421, 1164, 978, 867 cm^{-1}. **HRMS** (ESI) m/z calcd for $C_{20}H_{25}NO_3Na$ $[M+Na]^+$: 350.1727; found: 350.1725.

Direct detection of aziridinium intermediate:

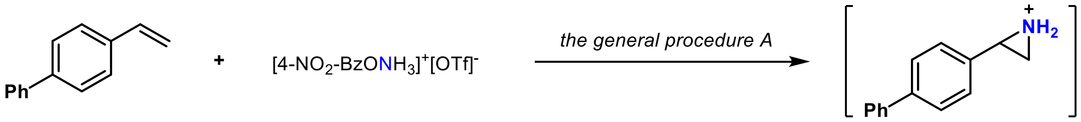

1a **V** **9**

calcd. for M^+: 196.1121
found for M^+: 196.1119

The aziridium ion **9** could be detected by ESI-MS analysis when the aminofluorination reaction was preformed after 3 min.

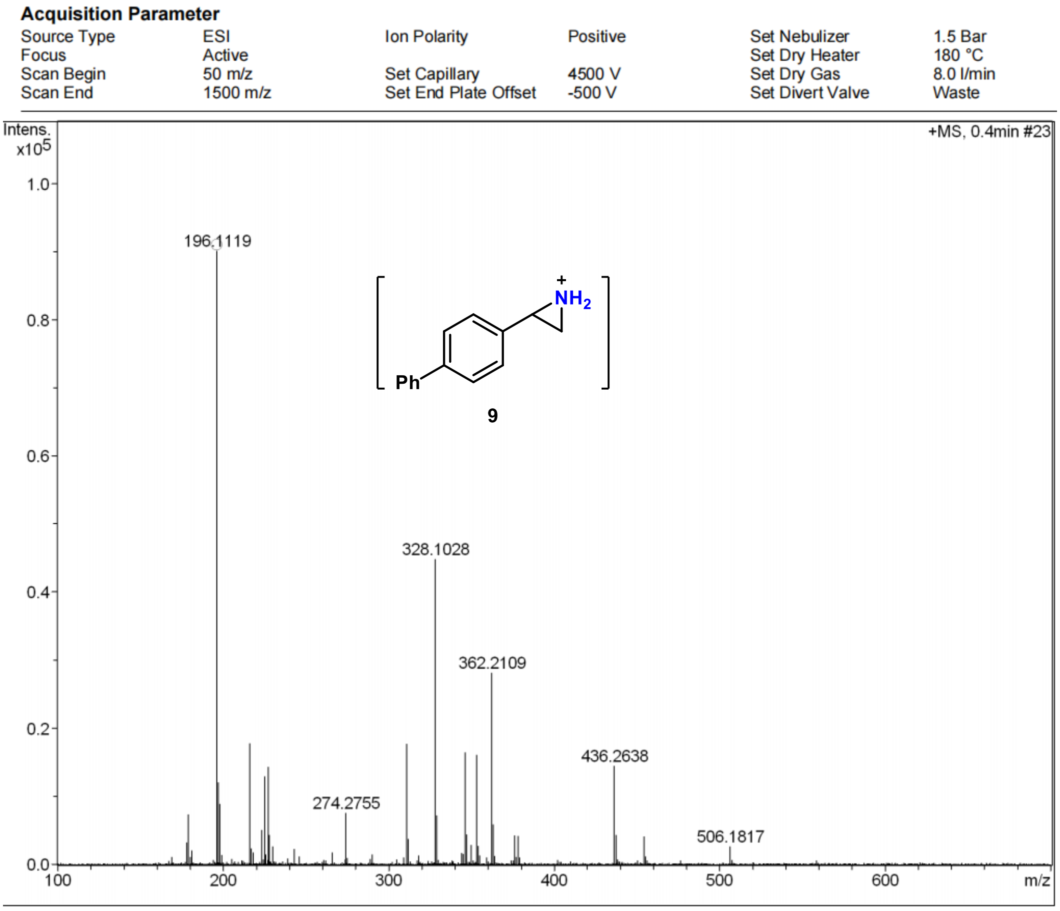

Meas. m/z	#	Ion Formula	m/z	err [ppm]	mSigma	# Sigma	Score	rdb	e⁻ Conf	N-Rule
196.1119	1	C14H14N	196.1121	0.7	52.5	1	100.00	8.5	even	ok

Supplementary Fig. 9. Characterization of **9** by mass spectrometry

The procedure for deprotection of Boc group:

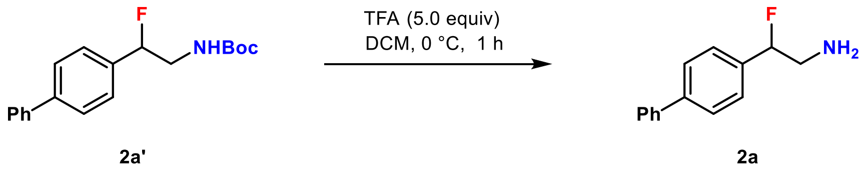

To a solution of **2a'** (63.1 mg, 0.20 mmol, 1.0 equiv) in DCM (1.0 mL) was added TFA (77 µL, 1.0 mmol, 5.0 equiv) at 0 °C, and the mixture was stirred for 1 h. The reaction was diluted with DCM (2.0 mL) and quenched with Et_3N (0.5 mL). The mixture was direct purified by flash column chromatography (DCM/MeOH = 20:1 as eluent) to afford β-fluoroamine **2a** as a yellow oil (38.3 mg, 0.18 mmol, 89%).

The procedure and characteristic data for the synthesis of KP23

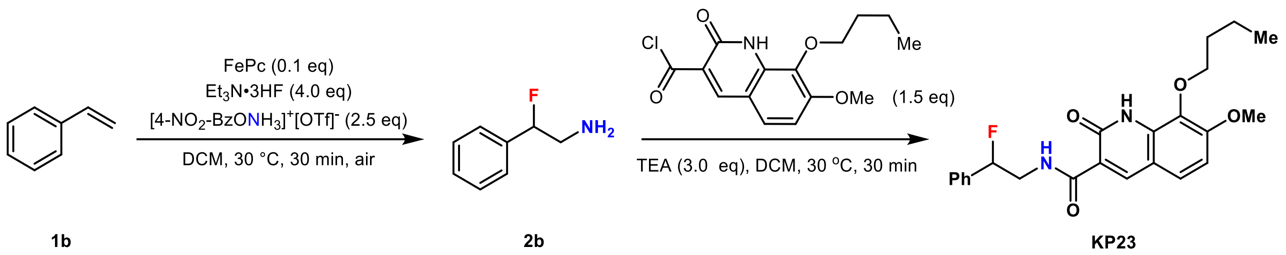

Hydroxylamine reagent 4-NO_2-BzONH_3OTf (**V**) (249 mg, 0.75 mmol, 2.5 equiv) was added to a plastic centrifuge tube charged with **1b** (23 µL, 0.30 mmol, 1.0 equiv), FePc (17.0 mg, 0.03 mmol, 0.1 equiv), $Et_3N\cdot 3HF$ (196 µL, 1.2 mmol, 4.0 equiv), and anhydrous CH_2Cl_2 (2.0 mL). The mixture was stirred in air at 30 °C (oil bath) for 30 min. Upon completion, the reaction was quenched with Et_3N (0.5 mL) at 0 °C. The reaction mixture was washed through a short column chromatography (EtOAc) to afford the crude β-fluoroamine **2b**.

The crude β-fluoroamine **2b** was charged into a 10 mL RB flask containing DCM (1.0 mL) and Et_3N (125 µL, 0.90 mmol, 3.0 equiv). Acyl chloride (139 mg, 0.45 mmol, 1.5 equiv in 1.0 mL DCM) was added slowly at 0 °C, and the reaction was stirred at 30 °C for 30 min. After that, the reaction was quenched by 1 M HCl (10 mL) and extracted with EtOAc (15 mL × 3). The combined organic layers were washed with brine, dried over anhydrous Na_2SO_4, filtered, and concentrated in *vacuo*. Purification of the crude product by flash column chromatography using PE/EA = 3:1 as the eluent afforded the desired product **KP23** as a white solid (81.7 mg, 0.20 mmol, 66% over two steps).[S30] **^{1}H NMR** (600 MHz, $CDCl_3$) δ 10.00 (t, J = 6.0 Hz, 1H), 9.26 (s, 1H), 8.85 (s, 1H), 7.47 – 7.33 (m, 6H), 6.93 (d, J = 9.0 Hz, 1H), 5.69 (ddd, J = 48.0, 8.4, 3.6 Hz, 1H), 4.15 (td, J = 6.6, 1.8 Hz, 2H), 4.07 (dddd, J = 30.6, 14.4, 6.6, 3.6 Hz, 1H), 3.97 (s, 3H), 3.81 – 3.66 (m, 1H), 1.87 – 1.70 (m, 2H), 1.57 – 1.46 (m, 2H), 0.99 (t, J = 7.2 Hz, 3H). **^{13}C NMR** (150 MHz, $CDCl_3$) δ 164.1, 162.2, 154.5, 145.1, 137.8 (d, J = 19.8 Hz), 133.7, 132.5, 128.8, 128.6, 125.7 (d, J = 7.0 Hz), 125.4, 119.4, 109.2, 92.8 (d, J = 173.8 Hz), 73.6, 56.4, 45.5 (d, J = 24.6 Hz), 32.3, 19.2, 13.9. **^{19}F NMR** (565 MHz, $CDCl_3$) δ -180.6 – -185.2 (m). **HRMS** (ESI) m/z calcd for $C_{23}H_{26}FN_2O_4$ $[M+H]^+$: 413.1871; found:

413.1876. **IR** ν_{max} (film): 2401, 1629, 1597, 1496, 1389, 1352, 1240, 1112, 1027, 771 cm^{-1}.

Supplementary Table 2. A comparative NMR study of compound KP23.

**^{1}H NMR** Literature[S30]	**^{1}H NMR** synthesized	**^{13}C NMR** literature	**^{13}C NMR** synthesized	**^{19}F NMR** literature	**^{19}F NMR** synthesized
10.00 (t, *J* = 6 Hz, 1H)	10.00 (t, *J* = 6.0 Hz, 1H)	164.0	164.1	183.1 (dt, J = 47.6, 30.8, 14.0 Hz).	-180.6 – -185.2 (m)
9.17 (s, 1H)	9.26 (s, 1H)	162.0	162.2		
8.88 (s, 1H)	8.85 (s, 1H)	154.5	154.5		
7.49 – 7.28 (m, 6H)	7.47 – 7.33 (m, 6H)	145.1	145.1		
6.95 (d, *J* = 9.0 Hz, 1H)	6.93 (d, *J* = 9.0 Hz, 1H)	137.8	137.8 (d, *J* = 19.8 Hz)		
5.70 (dm, $J_{(HF)}$ = 48 Hz, 1H)	5.69 (ddd, *J* = 48.0, 8.4, 3.6 Hz, 1H)	137.6			
4.19 – 4.14 (m, 3H)	4.15 (td, *J* = 6.6, 1.8 Hz, 2H)	133.6	133.7		
	4.07 (dddd, *J* = 30.6, 14.4, 6.6, 3.6 Hz, 1H)	132.4	132.5		
3.99 (s, 3H)	3.97 (s, 3H)	128.7	128.8		
3.85 – 3.65 (m, 1H)	3.81 – 3.66 (m, 1H)	128.4	128.6		
1.84 – 1.76 (m, 2H)	1.87 – 1.70 (m, 2H)	125.64	125.7 (d, *J* = 7.0 Hz)		
1.57 – 1.50 (m, 2H),	1.57 – 1.46 (m, 2H)	125.58			
1.02 (t, *J* = 7.2 Hz, 3H).	0.99 (t, *J* = 7.2 Hz, 3H)	125.4	125.4		
		119.4	119.4		
		109.1	109.2		
		92.71 (d, = 172.5 Hz)	92.8 (d, *J* = 173.8 Hz)		
		73.6	73.6		
		56.3	56.4		
		45.40 (d, J = 24.0 Hz)	45.5 (d, *J* = 24.6 Hz)		
		32.3	32.3		
		19.2	19.2		
		13.8	13.9		

Part 7: Radiochemistry

Radioisotope production and preparation

Anhydrous TMAF is highly hygroscopic, and any moisture incorporation results in significantly diminishes fluoride nucleophilicity. However, TMAF and other quaternary ammonium fluoride salts are known to form adducts with methanol, ethanol, isopropanol, tert-butanol, and tert-amyl alcohol that are highly moisture resistant and excellent fluoride-ion sources.[S31] In this study, [^{18}F]TMAF was azeotropically dried with a 1:1 mixture of CH_3CN and hexafluoroisopropanol (HFIP) to give the proposed [^{18}F]TMAF·HFIP adduct as a fluoride-ion transfer reagent.

A Scanditronix MC17 cyclotron was used for [^{18}F]fluoride production via the ^{18}O(p,n)^{18}F nuclear reaction by irradiating ^{18}O-enriched water. [^{18}F]fluoride was delivered to a lead-shielded hot cell in ^{18}O-enriched water by nitrogen gas pressure. It was then prepared for radiofluorination by passing the target water through a Waters Sep-Pak Accell Plus QMA Plus Light Cartridge (130 mg) that had been preconditioned with 5 mL EtOH, 5 mL TMACl $_{(aq)}$ (90 mg/mL), and 10 mL H_2O, and subsequently flushed with air. The captured [^{18}F]fluoride was eluted with a solution of TMACl (~7.5 mg) in 1 mL of 1:1 CH_3CN/HFIP to afford [^{18}F]TMAF·HFIP, which was dried on an automated radiosynthesis platform in two steps: first, the solution was evaporated to dryness by heating under vacuum at 85 ^{o}C for 5 min. Next, 1 mL of 1:1 CH_3CN/HFIP was added to the reaction vial, and the vessel was placed under vacuum and heated to 85 ^{o}C for 5 min, followed by 115 ^{o}C for 5 min. Alternatively, [^{18}F]Fluoride could be eluted with TMAOTf (~7.5 mg) in 1 mL of 1:1 CH_3CN/HFIP and dried as previously described to afford [^{18}F]TMAF·HFIP.

Anhydrous [^{18}F]TBAF was prepared by passing [^{18}F]fluoride in [^{18}O]H_2O target water through a Waters PS HCO_3^- anion exchange cartridge which had been preconditioned using 5 mL EtOH, 5 mL KOTf$_{(aq)}$ (90 mg/mL), and 10 mL H_2O, and eluted using TBAOTf (~9 mg) in 1 mL MeOH. The wet, methanolic [^{18}F]TBAF was then azeotropically dried on an automated radiosynthesis platform in two steps: first, the solution was evaporated to dryness by heating under vacuum at 85 ^{o}C for 5 min. Next, 1 mL of anhydrous CH_3CN was added to the reaction vial, and the vessel was placed under vacuum and heated to 85 ^{o}C for 5 min, followed by 115 ^{o}C for another 5 min to afford anhydrous [^{18}F]TBAF.

Anhydrous [^{18}F]AgF was prepared by capturing [^{18}F]fluoride from [^{18}O]H_2O target water on a Waters PS HCO_3^- anion exchange cartridge preconditioned using 5 mL EtOH, 5 mL KOTf$_{(aq)}$ (90 mg/mL), and 10 mL of H_2O. The [^{18}F]fluoride was eluted with AgOTf (~8 mg) dissolved in 1 mL of CH_3CN/H_2O (9:1), followed by drying with 1 mL of anhydrous CH_3CN as previously described.

Analysis of radiofluorination reactions

Radioactivity was quantified using a Capintec Radioisotope Calibrator (CRC-712M) ion chamber. Radio-TLC analysis was employed to determine radiochemical identity, purity, and yield. After the reaction, crude mixtures were

spotted onto alumina TLC plates and developed with mixtures of hexanes:ethyl acetate or toluene:ethyl acetate in a glass TLC chamber. Product identification was confirmed by comparing the retention factors (Rf) of radio-TLC spots with those of the fluorinated standards under identical TLC conditions. Radiochemical conversion (RCC) was calculated by integrating the area under the curve for the radio-TLC product peak and dividing by the total counts on the TLC plate after subtracting background signal from a region which had no activity. Decay-corrected radiochemical yield (RCY) for [^{18}F]**2a** and [^{18}F]KP23 was calculated as the product of release efficiency, resolubilization efficiency, RCC, and isolation efficiency, as described by Herth et al.,[S32] and decay-corrected to end of beam. Due to the limitations of performing manual radiosynthesis, trapping efficiency could not be measured due to potential high radioactivity exposure, and was thus omitted from the calculation. Starting activity was defined as the radioactivity measured in the reactor after the addition of DCM/HFIP (8:1) containing dissolved [^{18}F]TMAF·HFIP. End-of-reaction activity was defined as the activity measured after Et_3N/DCM evaporation and CH_3CN redissolution, before filtration (vide infra). For analytic radio-HPLC, either a Synergi Fusion column (250 x 4.6 mm, 5 µm) or Phenomenex Prodigy column (250 x 4.6 mm, 10 µm) were used. The mobile phase comprised varying ratios of CH_3CN and 0.1% aqueous formic acid or ammonium formate (50mM) at a flow rate of 1-2 mL/min, with injection volumes between 20-80 µL. The HPLC systems consisted of a Waters 1515 Isocratic HPLC Pump equipped with a Waters 2487 Dual λ Absorbance Detector, and a Bioscan Flow-Count equipped with a NaI crystal, controlled via Breeze software, and a Prominence LC-20AT HPLC Pump equipped with a Prominence SPD-20A Absorbance detector, a Bicron frisk-tech radiation detector, and eDAQ Powerchrom software. For isolation and analysis of [^{18}F]**2a** and [^{18}F]KP23, analytic radio-HPLC was carried out using an Advion-Avant Interchim Scientific HPLC and UHPLC equipped with a Luna C(18)2 column (250 x 4.6 mm, 10 µm), A-2041 UV-DAD (2 Hz, 200 – 400 nm), A-2045/A-2046 UV-Vis DAD (20 Hz, 200 – 800 nm) and Carroll & Ramsey Associates (Model 105-S) analogue gamma detector. In certain cases, unprotected and Boc-protected aminofluorination products showed retention on the column and required ethanol washing for elution. Analytical HPLC chromatograms are provided for products that were successfully eluted under standard conditions, strictly for product identification purposes.

Manual ^{19}F-aminofluorination reaction screening - replacement of $Et_3N·3HF$.

Before carrying out radiochemical reactions, various cold reaction conditions were screened to determine an optimal combination of reagents as a replacement for $Et_3N·3HF$. The screening of cold aminofluorination reactions was performed using **1a** as the model substrate on a 0.10 mmol scale, as outlined below:

The fluoride source (0.5 equiv) and Et_3N (0.2 equiv) were dissolved in the reaction solvent (1 mL) and added to a mixture of alkene (1.0 equiv), FePc (0.1 equiv), additives, and hydroxylamine reagent **V** (2.5 equiv). The mixture was stirred under air at 30 °C (oil bath) for 30 min. Upon completion, the reaction was quenched with Et_3N (0.3 mL), followed by the addition of Boc_2O (10 equiv). The mixture was then stirred for an additional 10 min.

Supplementary Table 3. Aminofluorination reaction optimization using nucleophilic fluoride sources.

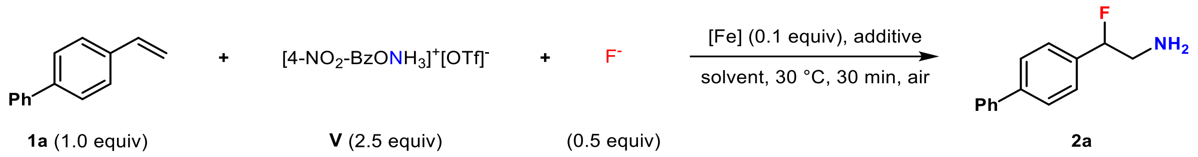

Entry	[Fe]	Fluoride source	Additive (equiv)	Solvent	Yield of **2a**[a]
1	FePc	KF	Et_3N (0.2)	DCM	0%
2	FePc	AgF	Et_3N (0.2)	DCM	Trace
3	FePc	TMAF	Et_3N (0.2)	DCM	10%
4	FePc	TBAF	Et_3N (0.2)	DCM	6%
5	FePc	TMAF	None	DCM	0%
6	FePc	TMAF	*N*-methyl-benzamide (0.2)	DCM	0%
7	FePc	TMAF	*N*, *N*-dimethyl-benzamide (0.2)	DCM	0%
8	FePc	TMAF	Et_3N (0.2) + HOTf (0.2)	DCM	0%
9	FePc	TMAF	Et_3N (0.2) + TFA (0.2)	DCM	0%
10	FePc	TMAF	Et_3N (0.2) + TsOH (0.2)	DCM	0%
11	FePc	TMAF	Et_3N (0.2) + $Et_2NH·HCl$ (0.2)	DCM	16%
12	FePc	TMAF	Et_3N (0.2) + $Et_2NH·HCl$ (0.2)	CH_3CN	Trace
13	FePc	TMAF	Et_3N (0.2) + $Et_2NH·HCl$ (0.2)	α,α,α-trifluorotoluene	0%
14	FePc	TMAF	Et_3N (0.2) + $Et_2NH·HCl$ (0.2)	dioxane	0%
15	FePc	TMAF	Et_3N (0.2) + $Et_2NH·HCl$ (0.2)	THF	0%
16	FePc	TMAF	Et_3N (0.2) + $Et_2NH·HCl$ (0.2)	DCM/CH_3CN (8:1)	Trace
17	FePc	TMAF	Et_3N (0.2) + $Et_2NH·HCl$ (0.2)	DCM/THF (8:1)	Trace
18	FePc	TMAF	Et_3N (0.2) + $Et_2NH·HCl$ (0.2)	DCM/dioxane (8:1)	Trace
**19**	**FePc**	**TMAF**	**Et_3N (0.2) + $Et_2NH·HCl$ (0.2)**	**DCM/HFIP** (8:1)	**25%**
20	$FeSO_4·7H_2O$	TMAF	Et_3N (0.2) + $Et_2NH·HCl$ (0.2)	DCM/HFIP (8:1)	0%
21	$Fe(OTf)_2$	TMAF	Et_3N (0.2) + $Et_2NH·HCl$ (0.2)	DCM/HFIP (8:1)	0%
22	$FeSO_4·7H_2O$ + 1,10-phenanthroline	TMAF	Et_3N (0.2) + $Et_2NH·HCl$ (0.2)	DCM/HFIP (8:1)	0%

[a]Yields of isolated products after Boc protection.

Screening results are summarized in **Supplementary Table 3.** Initial attempts to replace $Et_3N·3HF$ focused on the use of Et_3N and several nucleophilic fluoride sources without the addition of an external proton source. The reaction of **1a** in DCM solvent under the general conditions described above with Et_3N and KF showed no formation of **2a**, whereas the use of AgF as the fluoride source showed trace product formation. This indicated that more

nucleophilic fluoride-ion sources were required for the reaction. Using quaternary ammonium salts, TBAF and TMAF afforded **2a** in 6% and 10% yield, respectively. Notably, the reaction with TMAF in the absence of Et_3N yielded no product, confirming that a nitrogen-based reductant is required for the reaction to proceed. Replacement of Et_3N with *N*-methyl-benzamide or *N*, *N*-dimethyl benzamide gave no product. Furthermore, the addition of proton sources such as HOTf, TFA, or TsOH proved detrimental, completely suppressing the formation of **2a**. The addition of $Et_2NH{\cdot}HCl$ improved the yield of **2a** to 16%. Using a fixed combination of Et_3N, $Et_2NH{\cdot}HCl$, and TMAF, we investigated the influence of solvent and iron sources. Reactions carried out in CH_3CN and in 8:1 mixtures of DCM/CH_3CN, DCM/THF, and DCM/dioxane resulted in only trace amounts of **2a**, consistent with the observation that *O*- and *N*-coordinating solvents suppress product formation in reactions with $Et_3N{\cdot}3HF$. Reactions in neat α,α,α-trifluorotoluene, dioxane, and THF showed no detectable **2a**, where α,α,α-trifluorotoluene was explored as a "green" alternative to DCM. Notably, the use of an 8:1 mixture of DCM/HFIP improved the yield of **2a** to 25%. This is attributed to the ability of HFIP to act as a labile proton source. Finally, the use of alternative iron sources was examined but showed no desired product formation.

Manual ^{18}F-aminoradiofluorination reaction optimization.

Four reactions were carried out simultaneously during the manual radiosyntheses process. Optimization of the aminoradiofluorination reaction was performed using **1a** as the model substrate under the following conditions:

<u>Et_3N as an additive:</u> The substrate (0.025-0.05 mmol), FePc (0.1 equiv), and hydroxylamine reagent **V** (2.5 equiv) were combined in air in a screw-cap borosilicate vial outfitted with a Teflon cap, a vent needle, and a stir bar. The [^{18}F]fluoride source was prepared as previously described and manually dissolved in 1.6 mL of solvent containing 0.8 equivalents of Et_3N. Approximately 0.4 mL of the DCM:HFIP solution containing [^{18}F]fluoride (ca. 3-10 mCi) and Et_3N (0.2 equiv) was then syringed into each of the four reaction vessels. Following this addition, the activity in each reaction vial was measured, and the mixture were heated to 30 °C for 30 min with stirring. Subsequently, Boc_2O (0.13 mL) and Et_3N (0.2 mL) were added, and the reaction was stirred for an additional 10 min.

<u>A combination of Et_3N and $Et_2NH{\cdot}HCl$ as the additive</u>: The substrate (0.025-0.05 mmol), FePc (0.1 equiv), hydroxylamine reagent **V** (2.5 equiv), and $Et_2NH{\cdot}HCl$ (0.1-1.0 equiv) were combined in air in a screw-cap vial outfitted with a Teflon cap, a vent needle, and a stir bar. The [^{18}F]fluoride source was prepared as previously described and manually dissolved in 1.6 mL of solvent containing 0.8 equivalents of Et_3N. Approximately 0.4 mL of the solvent solution containing the [^{18}F]fluoride source (ca. 3-10 mCi) and Et_3N (0.2 equiv) was then syringed into each of the four reaction vessels. Following solvent addition, the radioactivity in the reaction vial was measured. The reaction mixture was heated to 30 °C for 30 min with stirring. Subsequently, Boc_2O (0.13 mL) and Et_3N (0.2 mL) were added, and the reaction was stirred for an additional 10 min.

The radioactivity in the reaction vial was measured after Boc-protection. To facilitate future application in automated radiotracer production, the reaction mixture must be soluble in aqueous media to enable injection onto a

reverse-phase semi-preparative radio-HPLC column. Therefore, DCM and excess Et_3N were removed by placing the reaction vessels under a continuous flow of nitrogen at 40 °C for 45 min. Afterward, the residues were resolubilized in 1 mL of anhydrous CH_3CN, and the activity of each solution was recorded. Radio-TLC and/or radio-HPLC were used to characterize the CH_3CN solutions. The samples were filtered through either a Waters Silica Plus or Alumina Plus Sep-Pak to neutralize excess Et_3N and were subsequently eluted with 4 mL of anhydrous CH_3CN. The filtered solutions demonstrated improved stability against moisture. The activities of the filtered solutions were recorded after filtration, and radio-TLC confirmed the presence of unreacted [^{18}F]TMAF, the desired ^{18}F-labeled β-fluoroamine products, and a minor ^{18}F-labeled side-product. Filtration proved to be an effective method for processing samples for purification and analysis by HPLC. Alternatively, approximately 0.2-0.5 mL of ethanol could be added to the CH_3CN solutions instead of filtering for radio-TLC analysis. A control reaction was carried out in the absence of alkene substrate which showed only the formation of the side-product.

Supplementary Table 4. Optimization of manual radiochemical reactions.

Entry	Alkene (mmol)	Fluoride source	Additive (equiv)	Solvent	RCC[a]
1	0.05	[^{18}F]AgF	Et_3N (0.2)	DCM/HFIP (8:1, 0.4 mL)	N.D.
2	0.05	[^{18}F]TBAF	Et_3N (0.2)	DCM/HFIP (8:1, 0.4 mL)	N.D.
3	0.05	[^{18}F]TBAF	Et_3N (0.2)	CH_3CN (0.4 mL)	N.D.
4	0.05	[^{18}F]TMAF·HFIP	Et_3N (0.2)	DCM/HFIP (8:1, 0.4 mL)	3%[b]
5	0.05	[^{18}F]TMAF·HFIP	$Et_2NH·HCl$ (1.0)	DCM/HFIP (8:1, 0.4 mL)	10%
6	0.05	[^{18}F]TMAF·HFIP	Et_3N (0.2) + $Et_2NH·HCl$ (0.5)	DCM/HFIP (8:1, 0.4 mL)	16%
7	0.05	[^{18}F]TMAF·HFIP	Et_3N (0.2) + $Et_2NH·HCl$ (0.25)	DCM/HFIP (8:1, 0.4 mL)	15%
8	0.05	[^{18}F]TMAF·HFIP	Et_3N (0.2) + $Et_2NH·HCl$ (0.1)	DCM/HFIP (8:1, 0.4 mL)	13%
9	0.025	[^{18}F]TMAF·HFIP	Et_3N (0.2) + $Et_2NH·HCl$ (0.5)	DCM/HFIP (8:1, 0.3 mL)	14%
10	0.025	[^{18}F]TMAF·HFIP	Et_3N (0.2) + $Et_2NH·HCl$ (0.5)	DCM/HFIP (8:1, 0.4 mL)	16%
11	0.025	[^{18}F]TMAF·HFIP	Et_3N (0.2) + $Et_2NH·HCl$ (0.5)	DCM/HFIP (8:1, 0.5 mL)	13%

[a] Radiochemical conversion was determined using radio-TLC. [b] Radiochemical conversion was determined by radio-HPLC.

A summary of the optimization results is provided in **Supplementary Table 4**. Using Et_3N (0.2 equiv) as the reductant, no product was observed when [^{18}F]AgF or [^{18}F]TBAF was employed as the fluoride source, whereas the use of [^{18}F]TMAF·HFIP showed the formation of [^{18}F]**2a'** by analytic radio-HPLC (3% RCC). Using a combination of Et_3N and $Et_2NH·HCl$ (0.1-1.0 equiv) significantly improved the RCCs, yielding [^{18}F]**2a'** in 10-16% RCCs. Reactions at both 0.05 mmol and 0.025 mmol scales afforded comparable conversions. Accurately weighing 0.1 equiv of $Et_2NH·HCl$ at 0.025 mmol scale proved challenging due to the tacky crystalline nature of the compound,

leading to the use of 0.25-0.5 equiv instead. Altering the reaction volume from 0.3-0.5 mL did not significantly affect the RCC, but 0.4 mL was selected as the optimal volume as it provided superior solubility and ease of mixing compared to 0.3 mL.

General Optimized Procedure for Manual Aminoradiofluorination.

Up to four reactions were carried out simultaneously during manual radiosyntheses. In a typical reaction, the substrate (0.025-0.05 mmol, 1 equiv) was combined in air with FePc (0.1 equiv), hydroxylamine reagent **V** (2.5 equiv), and $Et_2NH \cdot HCl$ (0.5 equiv) in a screw-cap vial outfitted with a Teflon cap, a vent needle, and a stir bar. The [^{18}F]fluoride source was prepared as previously described and manually dissolved in 1.6 mL of 8:1 DCM/HFIP containing Et_3N (0.8 equiv). Approximately 0.4 mL of the DCM/HFIP solution containing [^{18}F]fluoride (ca. 3-10 mCi) and Et_3N (0.2 equiv) was then syringed into each of the four reaction vessels. After measuring the initial activity, the samples were heated to 30 ^{o}C for 30 min with stirring. Subsequently, Boc_2O (10 equiv) and Et_3N (0.2 mL) were added, and the mixture was stirred for an additional 10 min. Alternatively in the case of **[^{18}F]KP23**, Et_3N (0.2 mL) and then DCM (0.3 mL) containing an acyl chloride coupling partner (1.5 equiv) were added and the mixture was stirred for an additional 10 min. The activity was measured post-coupling, and the samples were placed under a continuous flow of nitrogen at 40 ^{o}C for 45 min to evaporate DCM and excess Et_3N. The resulting solid mixtures were dissolved in 1 mL anhydrous CH_3CN, followed by a measurement of the activity, and the products were characterized by radio-TLC and/or analytic radio-HPLC. Finally, the solutions were filtered through a Waters Silica Plus or Alumina Plus Sep-Pak using 4 mL anhydrous CH_3CN as the eluent. The activity was measured again, and the Boc-protected products were characterized once more using radio-TLC and/or analytic radio-HPLC.

Radiosynthesis and Isolation of [^{18}F]2a' for RCY and A_m Determination.

Biphenyl substrate **1a** (0.025 mmol) was combined in air with FePc (0.1 equiv), hydroxylamine reagent **V** (2.5 equiv), and $Et_2NH \cdot HCl$ (0.5 equiv) in a screw-cap vial outfitted with a Teflon cap, a vent needle, and a stir bar. The [^{18}F]fluoride source was prepared as previously described and manually dissolved in 0.6 mL of 8:1 DCM/HFIP containing Et_3N (0.8 equiv). Approximately 0.4 mL of the DCM/HFIP solution containing [^{18}F]fluoride (ca. 30 mCi) and Et_3N (0.2 equiv) was then syringed into a vial containing the substrate. The DCM/HFIP/[^{18}F]fluoride/Et_3N/ substrate mixture was then syringed the reaction vessel. After measuring the initial activity, the sample was heated to 40 ^{o}C for 30 min with stirring. Subsequently, Boc_2O (10 equiv) and Et_3N (0.2 mL) were added, and the mixture was stirred for an additional 10 min at 30 ^{o}C. The activity was measured pre-coupling, and the samples were placed under a continuous flow of nitrogen at 30 ^{o}C for 10 min to evaporate DCM and excess Et_3N. After drying, 1 mL of hexane was added to extract the product from the crude and syringed into a separate vial before being dried under a flow of nitrogen for 5 min to evaporate the hexane. The product was diluted with 1 mL of CH_3CN, and the 20 µL (ca. 54 µCi) of the mixture was injected into an analytical HPLC column and manually purified (Luna C(18)2, 250 × 4.6 mm, 10 µm) using a gradient (Solvent A: 100 mM ammonium formate; Solvent B: CH_3CN, 5-90% Solvent B

over 5 min at a flow rate of 1 mL/min). The retention time for **[^{18}F]2a'** was 8.57 min (UV peak) and 8.69 min (radio peak). The product peak was manually collected and the activity in the vial was measured. RCY and A_m were determined through reverse-phase HPLC. Radiochemical identity was confirmed via a co-injection of an authentic standard with the aliquot.

Radiosynthesis and Isolation of [^{18}F]KP23 for RCY and A_m Determination

[^{18}F]KP23 was synthesized following the same method as **[^{18}F]2a'.** The styrene (**1b**) was fluoroaminated followed by coupling with acyl chloride. The product was similarly confirmed and isolated by HPLC.

Aminoradiofluorination Substrate Scope.

Calculation of Retention Factor (Rf): Rf is calculated as the distance traveled by the labeled compound or authentic standard divided by the distance traveled by the solvent front, measured from the origin. The origin is indicated by the horizontal line drawn across the bottom of the TLC plate where the sample was spotted. The solvent front is indicated by the second horizontal line drawn at the top of the TLC plate. On radio-TLC the x-axis begins at 0 mm, which corresponds to the bottom of the TLC plate, not the origin. Rf values for radio-TLC are therefore calculated as follows:

$$Rf_{\text{radio-TLC}} = \frac{(\text{distance of gamma peak from bottom of TLC plate} - \text{distance of origin from bottom of TLC plate})}{(\text{distance of solvent front from bottom of TLC plate} - \text{distance of origin from bottom of TLC plate})}$$

Note: *Product gamma peaks are indicated with a black circle overlay on crude reaction TLCs.*

Aminoradiofluorination Substrate Scope.

Calculation of Retention Factor (Rf): Rf is calculated as the distance traveled by the labeled compound or authentic standard divided by the distance traveled by the solvent front, measured from the origin. The origin is indicated by the horizontal line drawn across the bottom of the TLC plate where the sample was spotted. The solvent front is indicated by the second horizontal line drawn at the top of the TLC plate. On radio-TLC the x-axis begins at 0 mm, which corresponds to the bottom of the TLC plate, not the origin. Rf values for radio-TLC are therefore calculated as follows:

$$Rf_{\text{radio-TLC}} = \frac{\text{(distance of gamma peak from bottom of TLC plate – distance of origin from bottom of TLC plate)}}{\text{(distance of solvent front from bottom of TLC plate – distance of origin from bottom of TLC plate)}}$$

Note: *Product gamma peaks are indicated with a black circle overlay on crude reaction TLCs.*

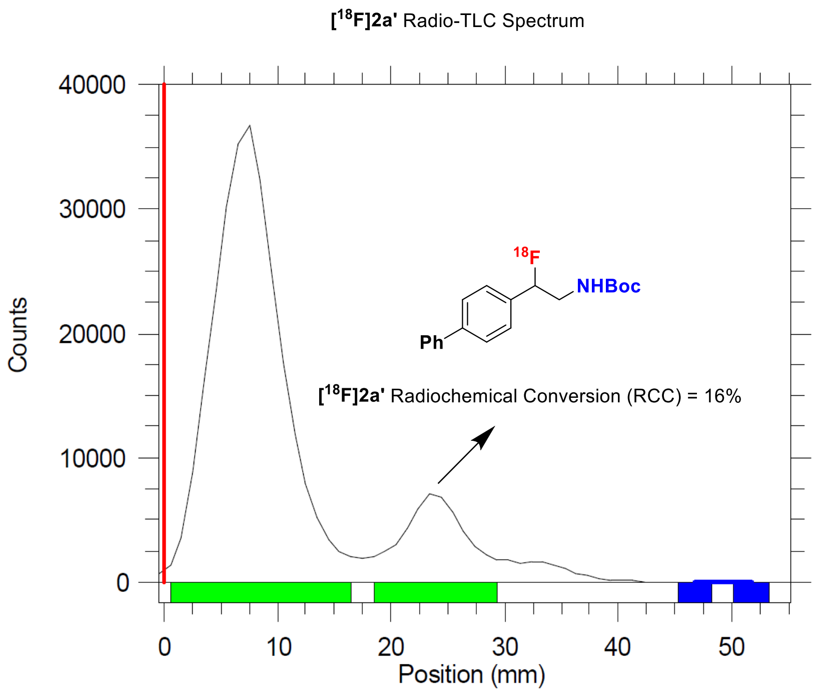

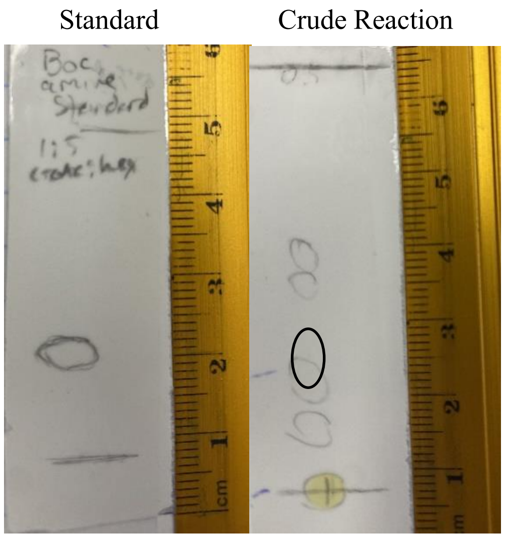

$Rf_{(standard)} = (2.0 - 0.8)/(4.9 - 0.8) = 0.30$

$Rf_{([^{18}F]2a' \text{ in crude})} = (2.4 - 0.7)/(6.4 - 0.7) = 0.30$

Supplementary Fig. 10. Radio-TLC spectrum of **[^{18}F]2a'** (1:5 EtOAc:Hexanes).

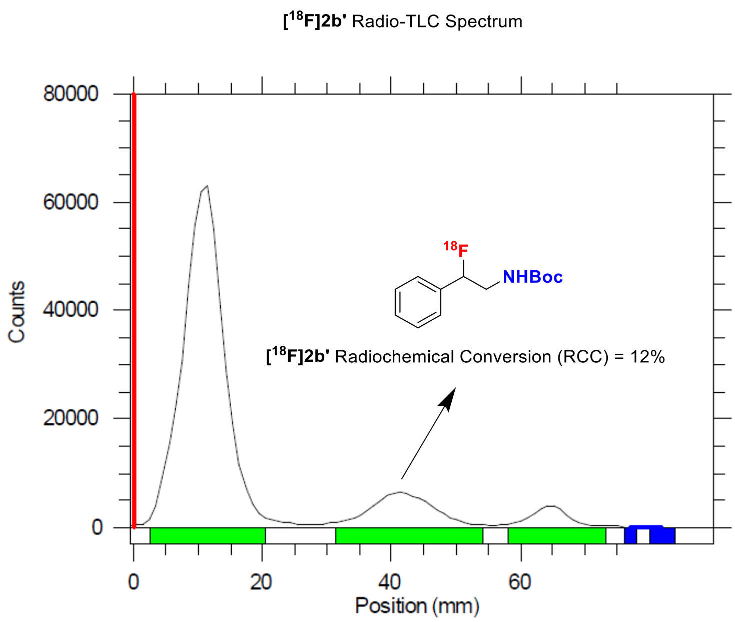

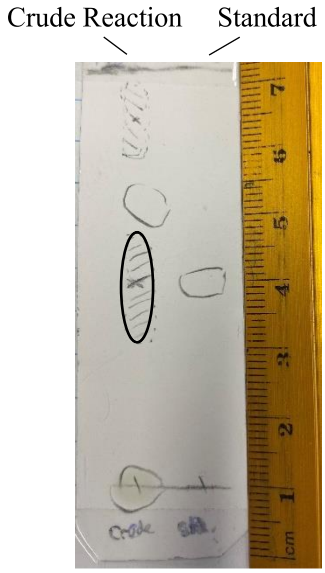

$Rf_{(standard)} = (4.1 – 1.0)/(7.2 – 1.0) = 0.5$

$Rf_{([^{18}F]2b' \text{ in crude})} = (4.1 – 1.0)/(7.2 – 1.0) = 0.5$

Supplementary Fig. 11. Radio-TLC spectrum of **[^{18}F]2b'** (1:12 EtOAc:Hexanes).

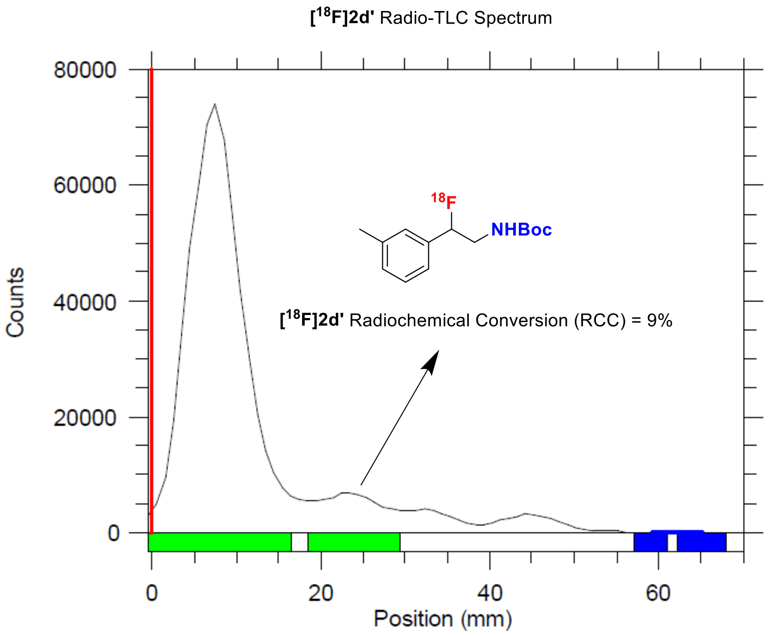

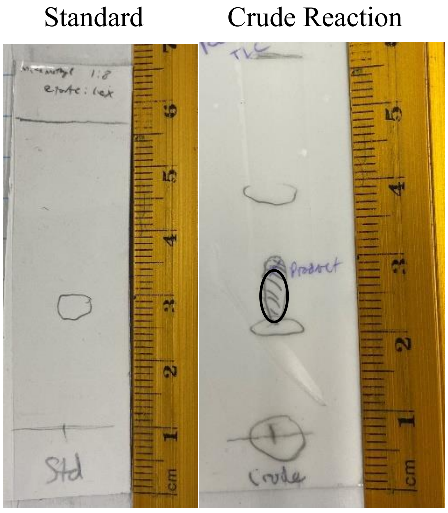

$Rf_{(standard)} = (2.8 – 1.0)/(5.7 – 1.0) = 0.38$

$Rf_{([^{18}F]2d' \text{ in crude})} = (2.4 – 0.6)/(5.6 – 0.7) = 0.37$

Supplementary Fig. 12. Radio-TLC spectrum of **[^{18}F]2d'** (1:8 EtOAc:Hexanes).

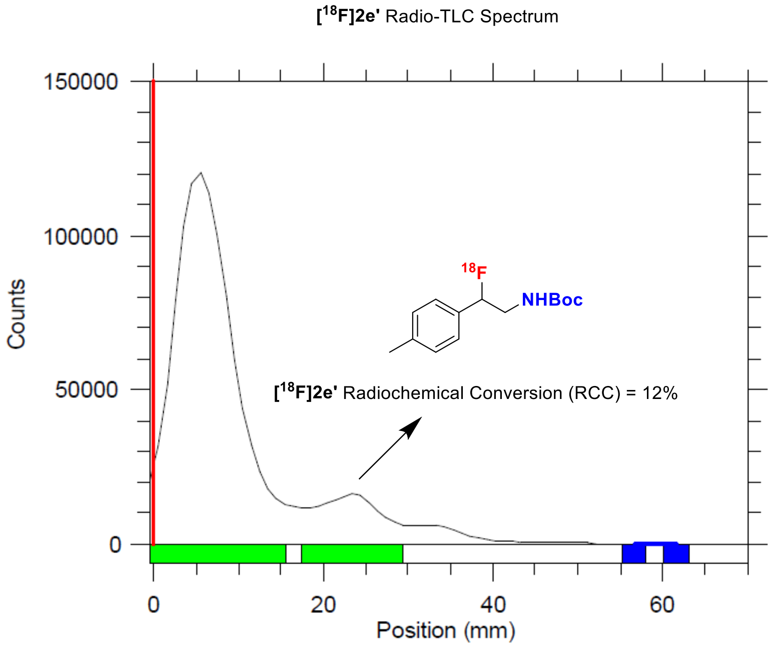

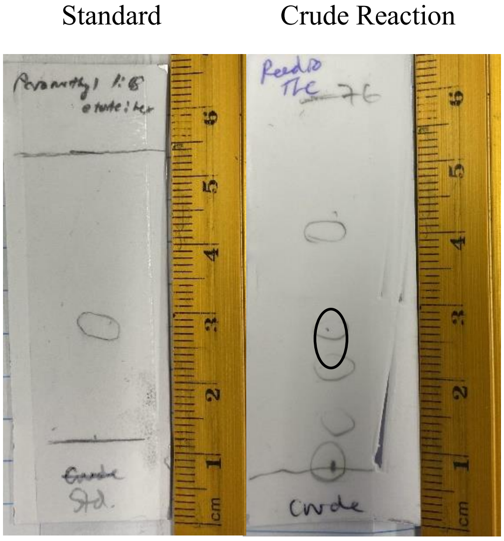

$Rf_{(standard)} = (2.7 – 1.2)/(5.4 – 1.2) = 0.36$

$Rf_{([^{18}F]2e' \text{ in crude})} = (2.5 – 0.6)/(5.9 – 0.6) = 0.36$

Supplementary Fig. 13. Radio-TLC spectrum of **[^{18}F]2e'** (1:8 EtOAc:Hexanes).

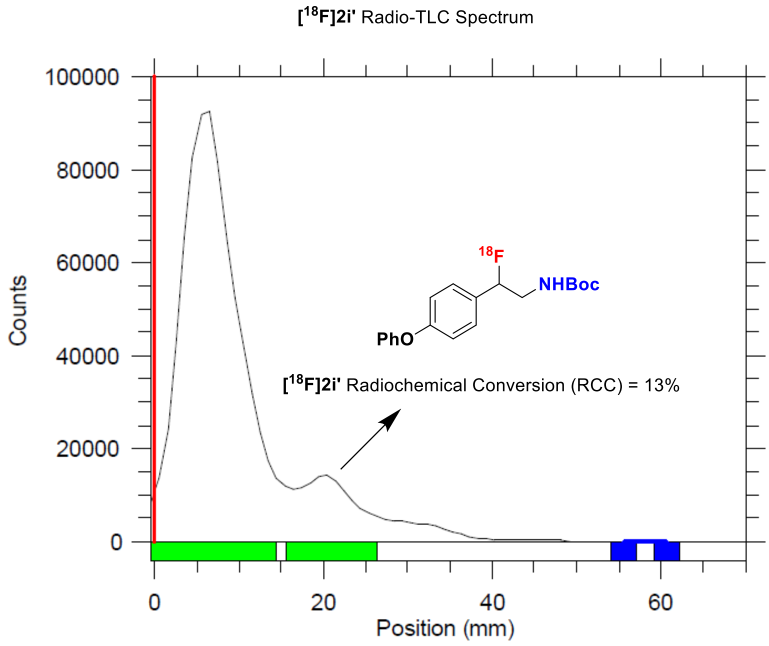

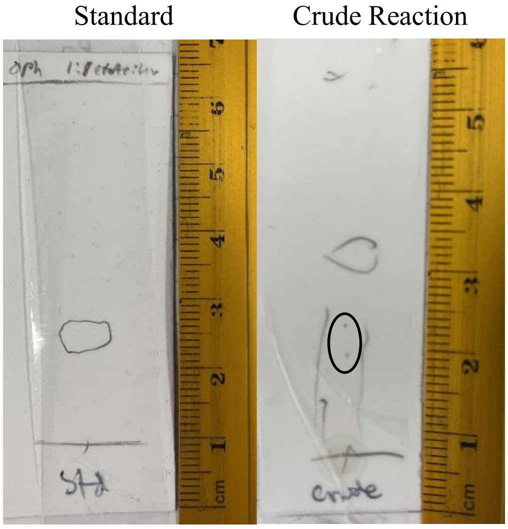

$Rf_{(standard)} = (2.5 – 0.9)/(6.2 – 0.9) = 0.30$

$Rf_{([^{18}F]2i' \text{ in crude})} = (2.1 – 0.6)/(5.5 – 0.6) = 0.31$

Supplementary Fig. 14. Radio-TLC spectrum of **[^{18}F]2i'** (1:8 EtOAc:Hexanes).

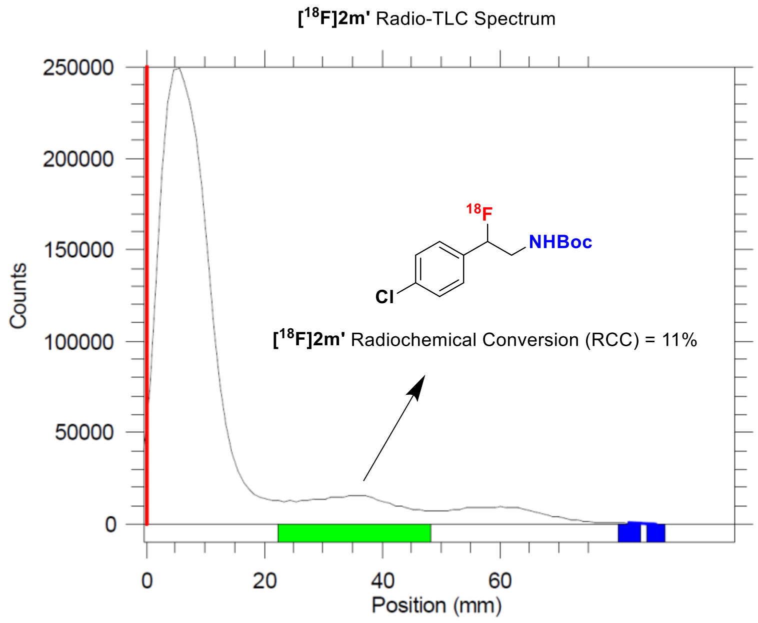

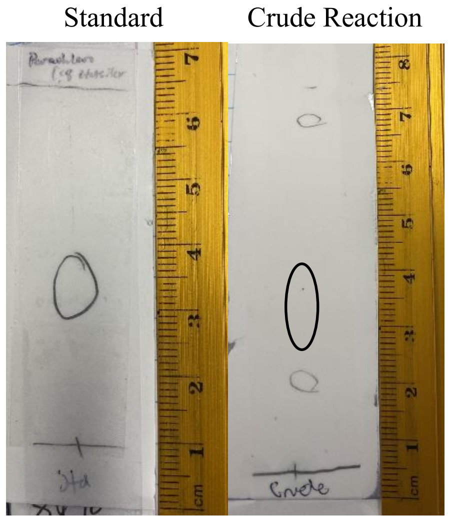

$Rf_{(standard)} = (3.4 – 0.9)/(6.4 – 0.9) = 0.45$

$Rf_{([^{18}F]2m' \text{ in crude})} = (3.6 – 0.4)/(7.4 – 0.4) = 0.46$

Supplementary Fig. 15. Radio-TLC spectrum of **[^{18}F]2m'** (1:8 EtOAc:Hexanes).

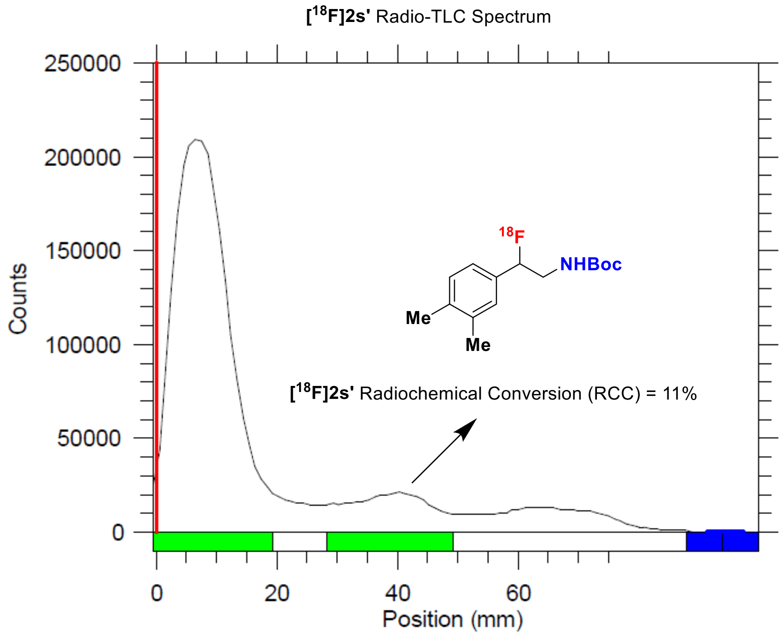

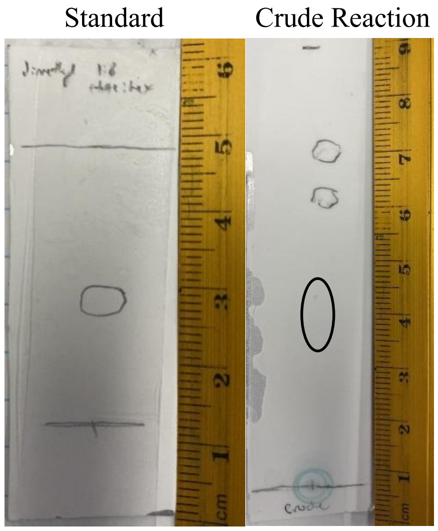

$Rf_{(standard)} = (2.8 – 1.2)/(4.9 – 1.2) = 0.43$

$Rf_{([^{18}F]2s' \text{ in crude})} = (4.1 – 0.5)/(9.0 – 0.5) = 0.42$

Supplementary Fig. 16. Radio-TLC spectrum of **[^{18}F]2s'** (1:8 EtOAc:Hexanes).

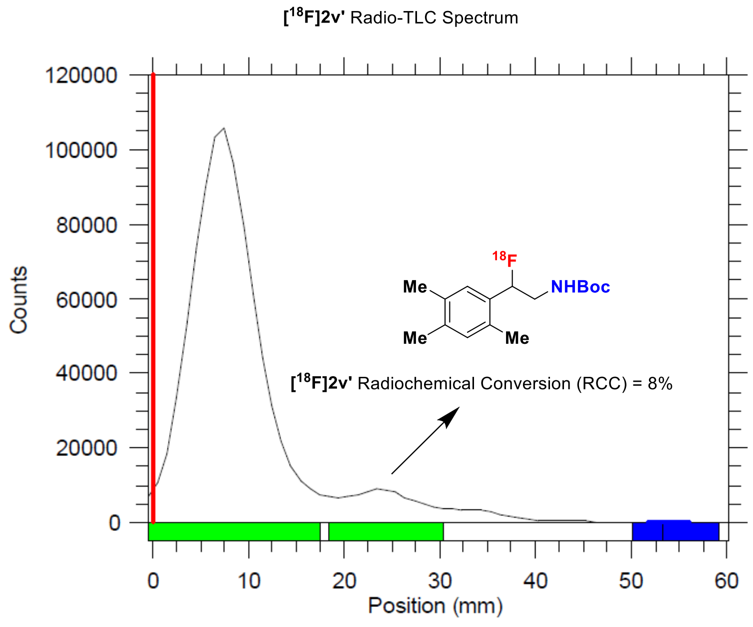

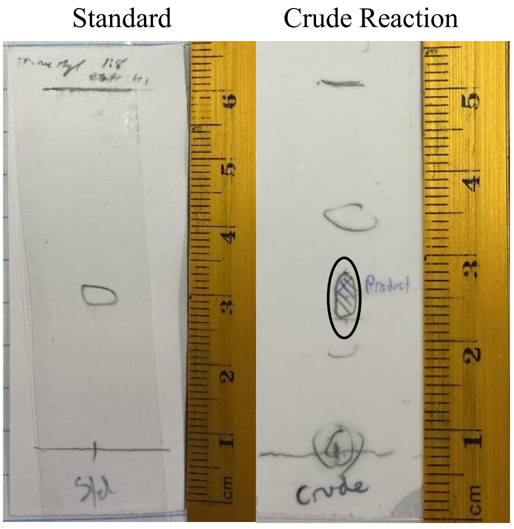

$Rf_{(standard)} = (2.9 – 0.8)/(6.0 – 0.8) = 0.40$

$Rf_{([^{18}F]2v' \text{ in crude})} = (2.4 – 0.7)/(5.1 – 0.7) = 0.39$

Supplementary Fig. 17. Radio-TLC spectrum of **[18F]2v'** (1:8 EtOAc:Hexanes).

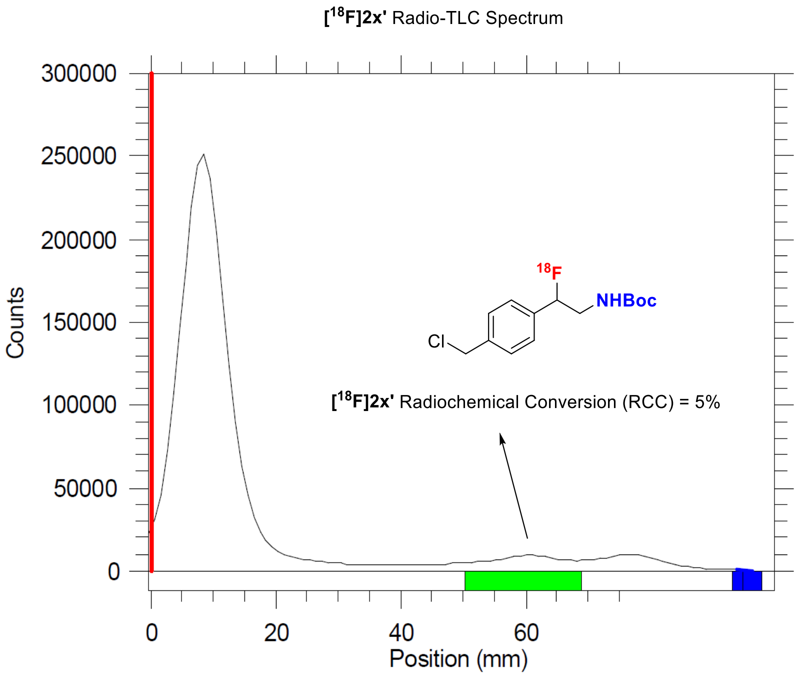

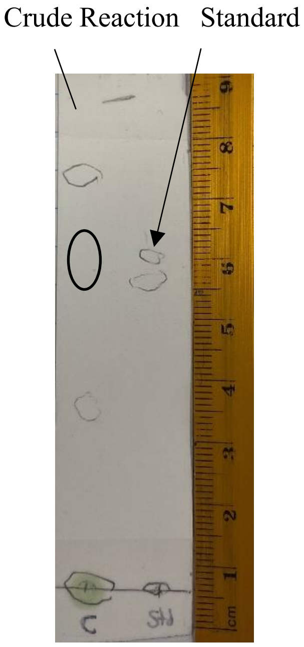

$Rf_{(standard)} = (6.0 – 0.6)/(8.7 – 0.6) = 0.67$

$Rf_{([^{18}F]2x' \text{ in crude})} = (6.0 – 0.6)/(8.7 – 0.6) = 0.67$

Supplementary Fig. 18. Radio-TLC spectrum of **[18F]2x'** (1:12 EtOAc:Toluene).

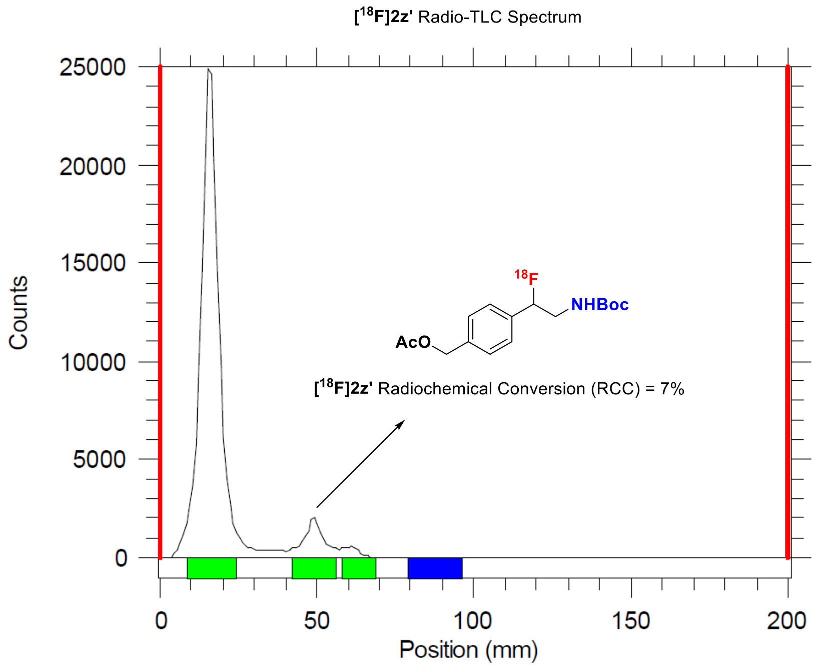

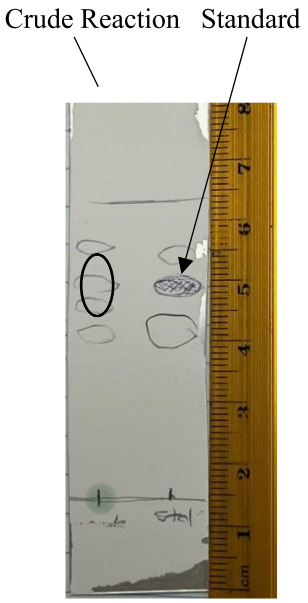

$Rf_{(standard)} = (4.9 – 0.9)/(6.7 – 0.9) = 0.69$

$Rf_{([^{18}F]2z' \text{ in crude})} = (4.9 – 0.9)/(6.7 – 0.9) = 0.69$

Supplementary Fig. 19. Radio-TLC spectrum of **[^{18}F]2z'** (3:4 EtOAc:Hexanes).

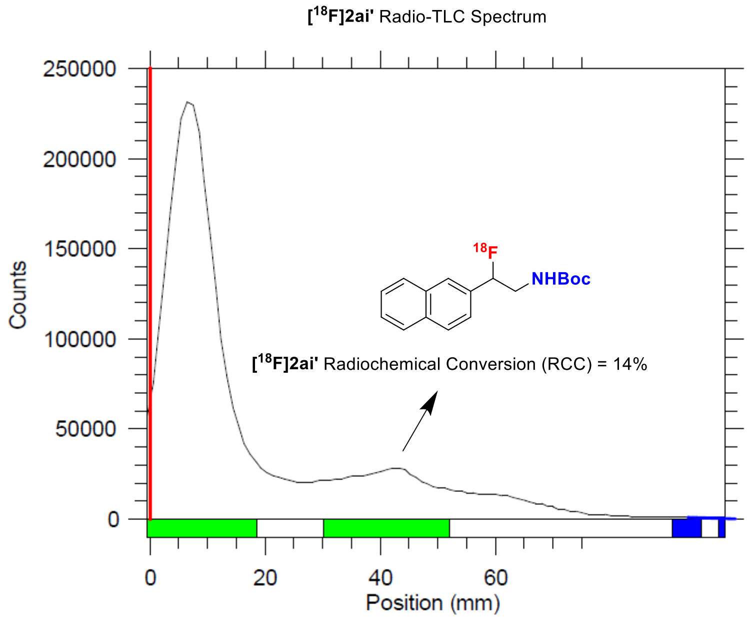

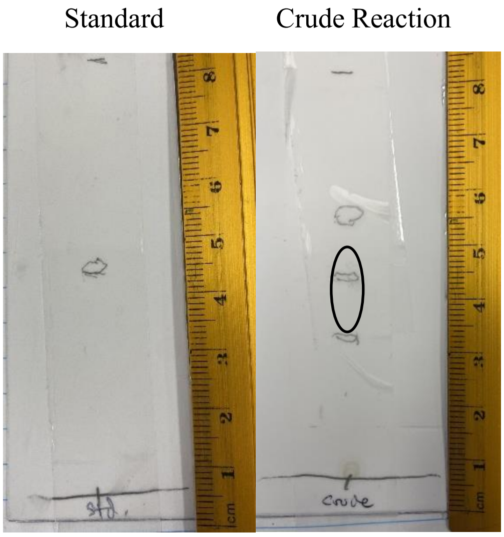

$Rf_{(standard)} = (4.4 – 0.6)/(8.3 – 0.6) = 0.49$

$Rf_{([^{18}F]2ai' \text{ in crude})} = (4.3 – 0.6)/(8.2 – 0.6) = 0.49$

Supplementary Fig. 20. Radio-TLC spectrum of **[^{18}F]2ai'** (1:12 EtOAc:Toluene).

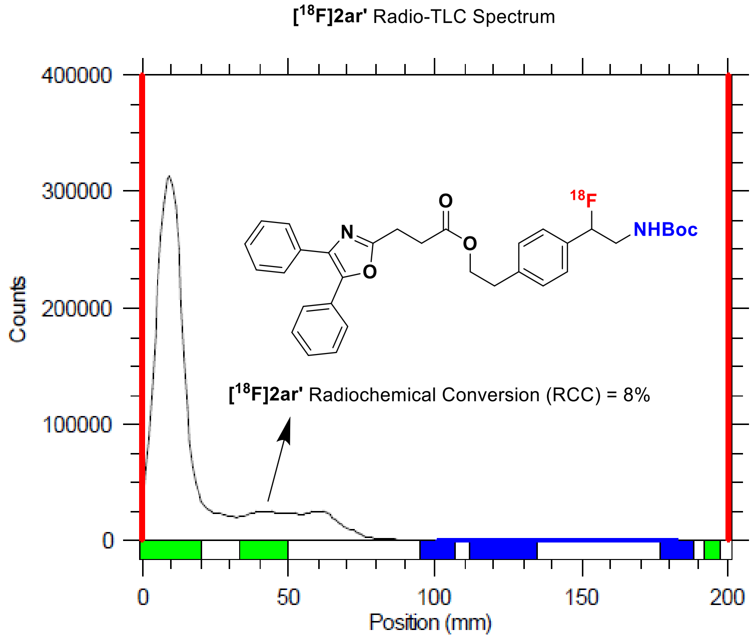

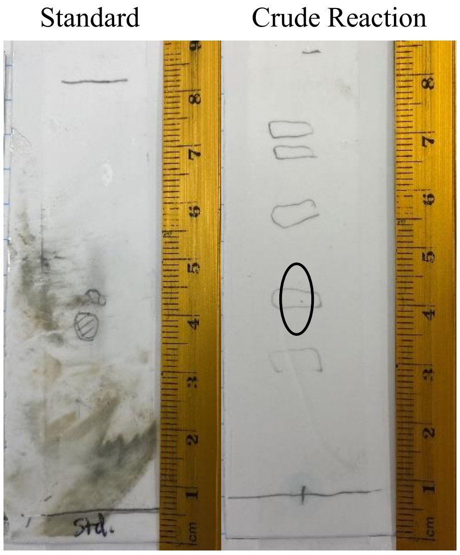

$Rf_{(standard)} = (3.9 – 0.5)/(8.2 – 0.5) = 0.44$

$Rf_{([^{18}F]2ar' \text{ in crude})} = (4.1 – 0.7)/(8.4 – 0.7) = 0.44$

Supplementary Fig. 21. Radio-TLC spectrum of **[^{18}F]2ar'** (1:6 EtOAc:Toluene).

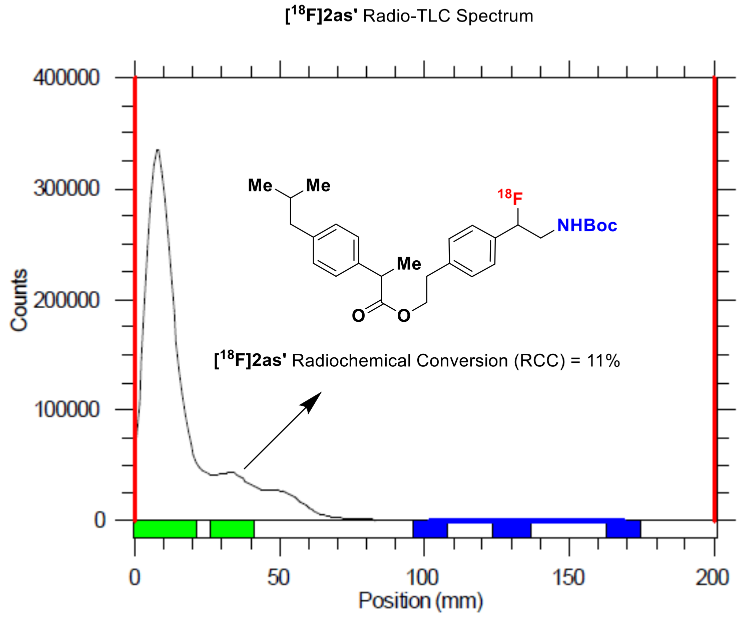

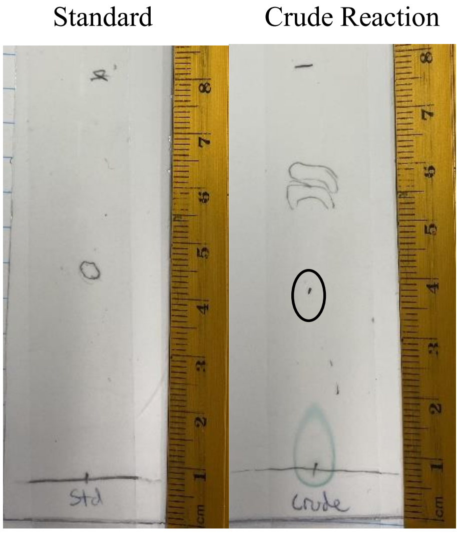

$Rf_{(standard)} = (4.3 – 0.8)/(8.2 – 0.8) = 0.47$

$Rf_{([^{18}F]2as' \text{ in crude})} = (3.8 – 0.6)/(7.7 – 0.6) = 0.45$

Supplementary Fig. 22. Radio-TLC spectrum of **[^{18}F]2as'** (1:12 EtOAc:Toluene).

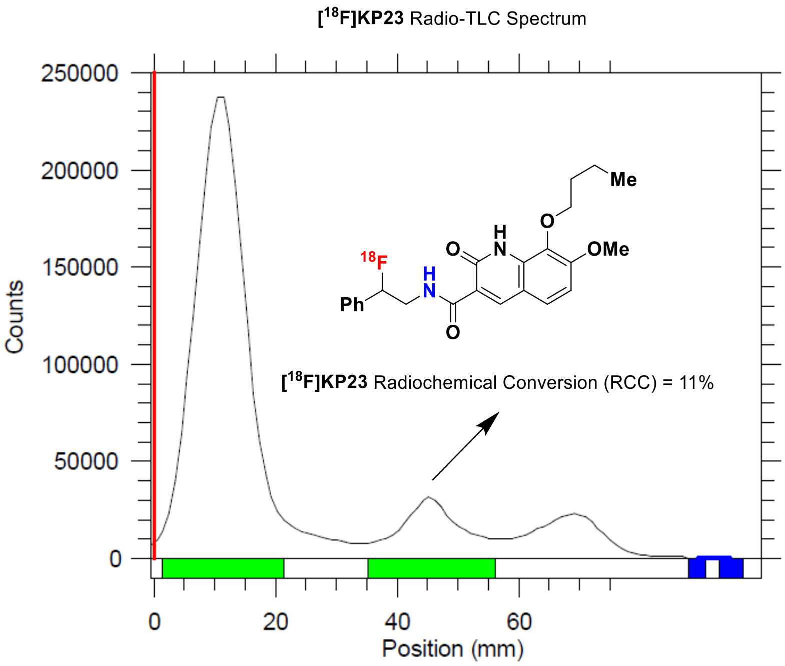

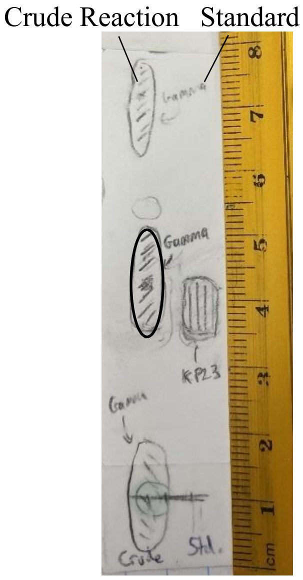

$Rf_{(standard)} = (4.1 – 1.0)/(7.9 – 1.0) = 0.45$

$Rf_{([^{18}F]KP23\ in\ crude)} = (4.2 – 1.0)/(7.9 – 1.0) = 0.46$

Supplementary Fig. 23. Radio-TLC spectrum of **[^{18}F]KP23** (1:12 EtOAc:Toluene).

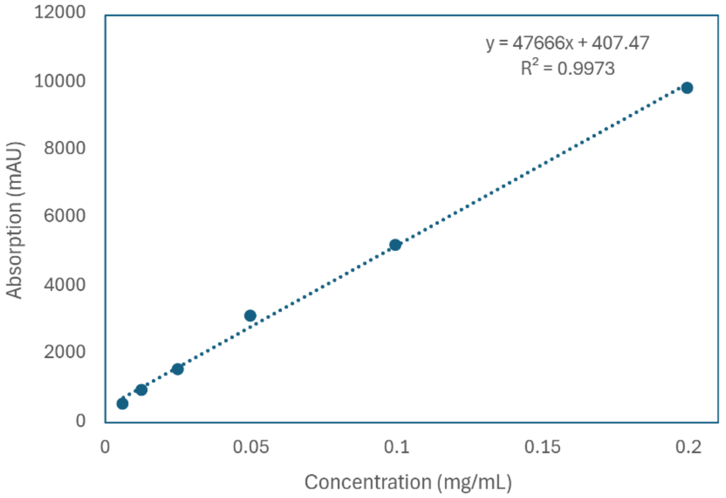

Supplementary Fig. 24. HPLC calibration curve for authentic reference **2a'**.

Supplementary Table 5. Molar activity of **[^{18}F]2a'** (n = 3).

Measurement	Activity Injected (MBq, d.c.)	Peak Area (mAu*s)	Activity Collected (MBq, d. c.)	A_m (GBq/nmol)
1	38.79	1.10×10^4	6.60	46.9
2	29.14	9.20×10^3	1.56	13.3
3	19.51	4.20×10^3	3.345	66.2

Average A_m = 42 ± 27 GBq/nmol

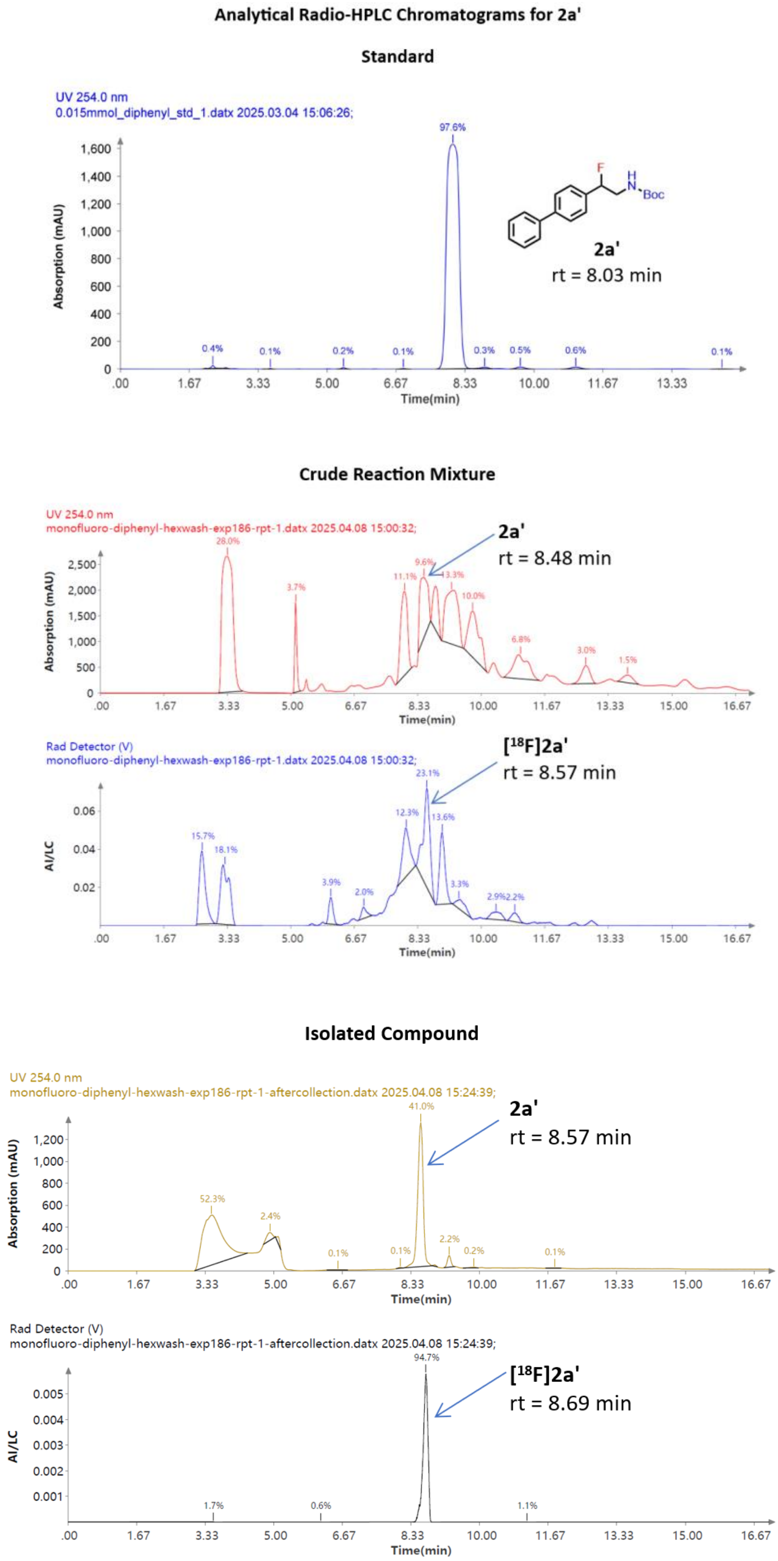

Supplementary Fig. 25. Analytical radio-HPLC chromatograms of **[^{18}F]2a'**. The chromatograms were recorded using a Advion-Avant Interchim Scientific HPLC and UHPLC equipped with a Luna C(18)2 column (250 x 4.6 mm, 10 µm), A-2041 UV-DAD (2 Hz, 200 – 400 nm), A-2045/A-2046 UV-Vis DAD (20 Hz, 200 – 800 nm) and Carroll & Ramsey Associates (Model 105-S) analogue gamma detector. Mobile phase: gradient (5:95 (5 mins) then 95:5 (15 mins) CH_3CN: 0.1 M ammonium formate$_{(aq)}$, 1mL/min flow, 30-50 µL injection).

[^{18}F]**2d'** Analytic Radio-HPLC Chromatograms

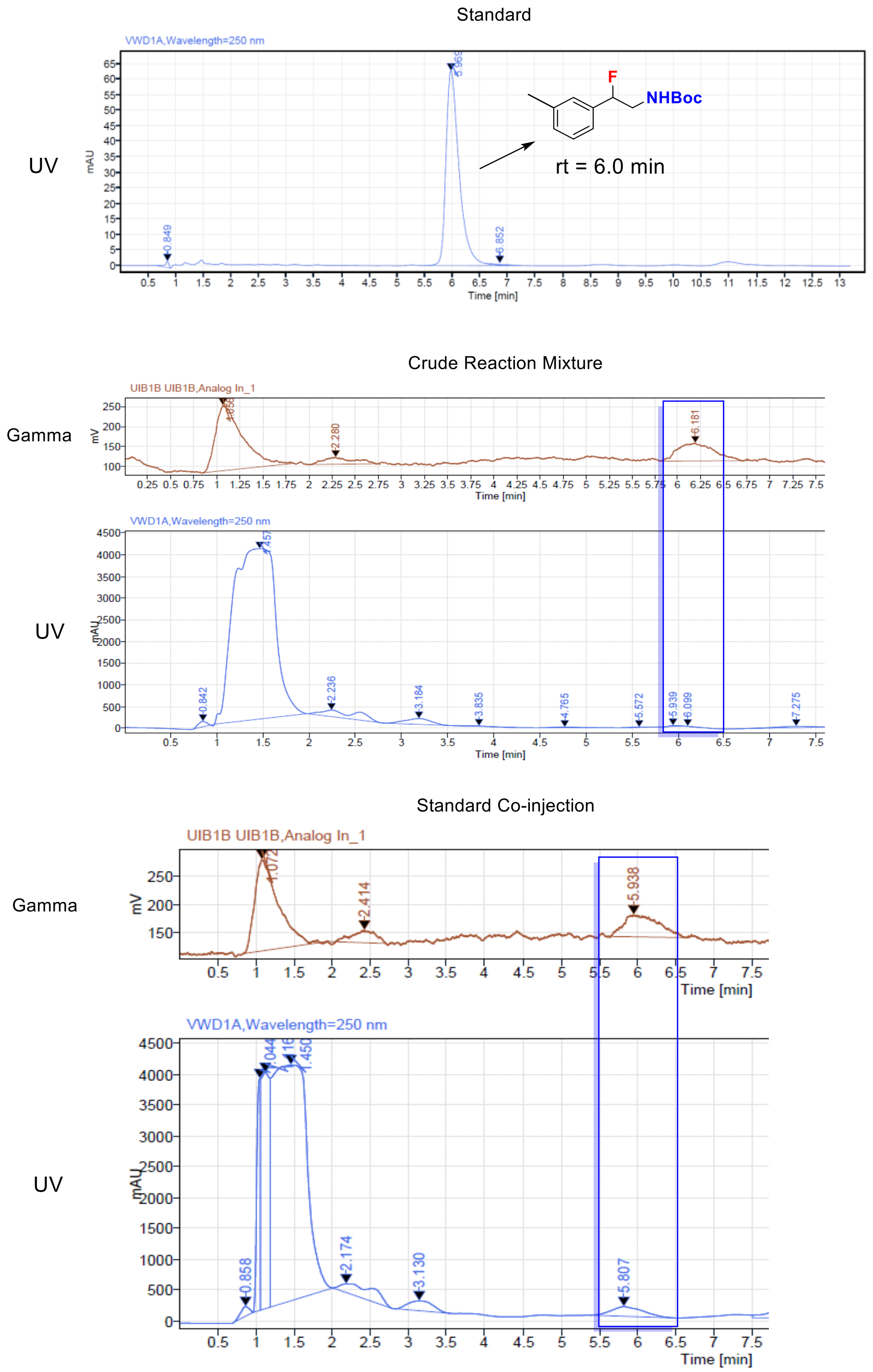

Supplementary Fig. 26. Analytical radio-HPLC spectra of **[^{18}F]2d'**. A Synergi Fusion column (250 x 4.6 mm, 5 µm, Mobile phase, 40:60 CH_3CN:0.1% Formic Acid$_{(aq)}$, 2mL/min flow, 20-80 µL injection) was used with a Waters 1515 Isocratic HPLC Pump equipped with a Waters 2487 Dual λ Absorbance Detector, a Bioscan Flow-Count equipped with a NaI crystal, and Breeze software.

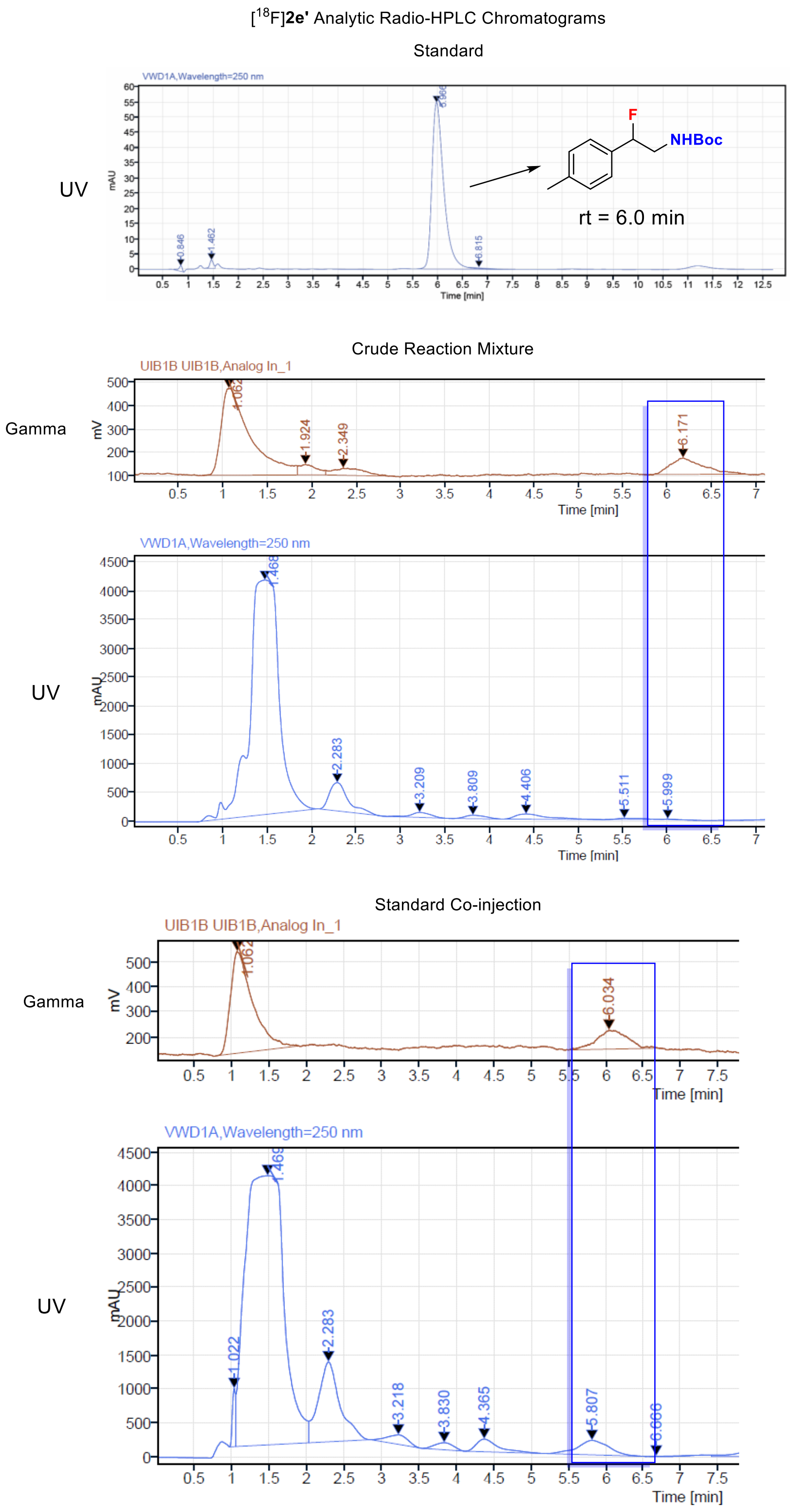

Supplementary Fig. 27. Analytical radio-HPLC spectra of **[^{18}F]2e'**. A Synergi Fusion column (250 x 4.6 mm, 5 µm, Mobile phase, 40:60 CH_3CN:0.1% Formic Acid$_{(aq)}$, 2mL/min flow, 20-80 µL injection) was used with a Waters 1515 Isocratic HPLC Pump equipped with a Waters 2487 Dual λ Absorbance Detector, a Bioscan Flow-Count equipped with a NaI crystal, and Breeze software.

[^{18}F]**2ai'** Analytic Radio-HPLC Chromatograms

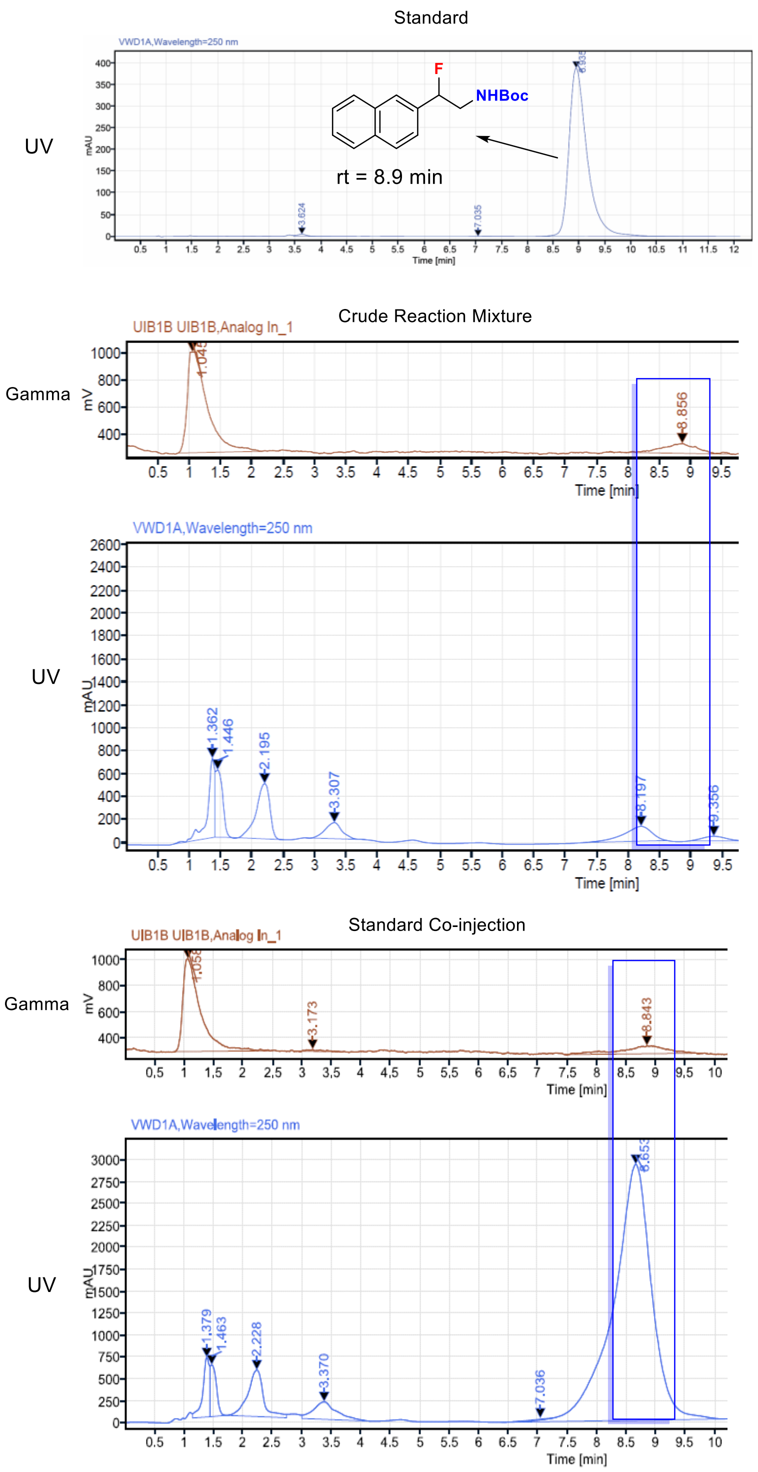

Supplementary Fig. 28. Analytical radio-HPLC spectra of **[^{18}F]2ai'.** Synergi Fusion column (250 x 4.6 mm, 5 µm, Mobile phase, 40:60 CH_3CN:0.1% Formic Acid$_{(aq)}$, 2 mL/min flow, 20-80 µL injection) was used with a Waters 1515 Isocratic HPLC Pump equipped with a Waters 2487 Dual λ Absorbance Detector, a Bioscan Flow-Count equipped with a NaI crystal, and Breeze software.

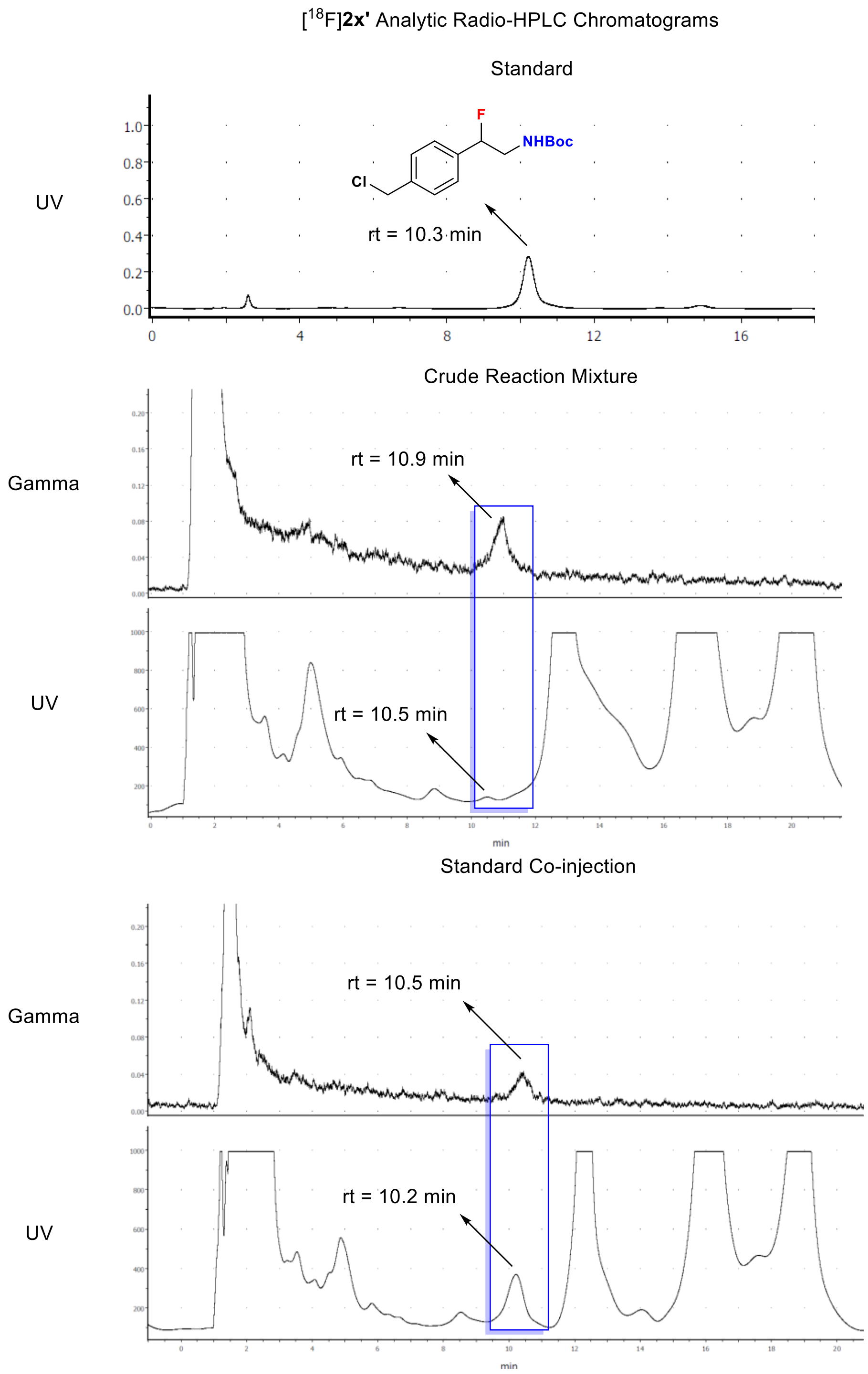

Supplementary Fig. 29. Analytical radio-HPLC spectra of **[^{18}F]2x'**. A Phenomenex Prodigy column (250 x 4.6 mm, 10 µm, Mobile phase, 54:46 CH_3CN:0.1% Formic Acid$_{(aq)}$, 1.35 mL/min flow, 20-80 µL injection) was used with a Prominence LC-20AT HPLC Pump equipped with a Prominence SPD-20A Absorbance detector, a Bicron frisk-tech radiation detector, and eDAQ Powerchrom software.

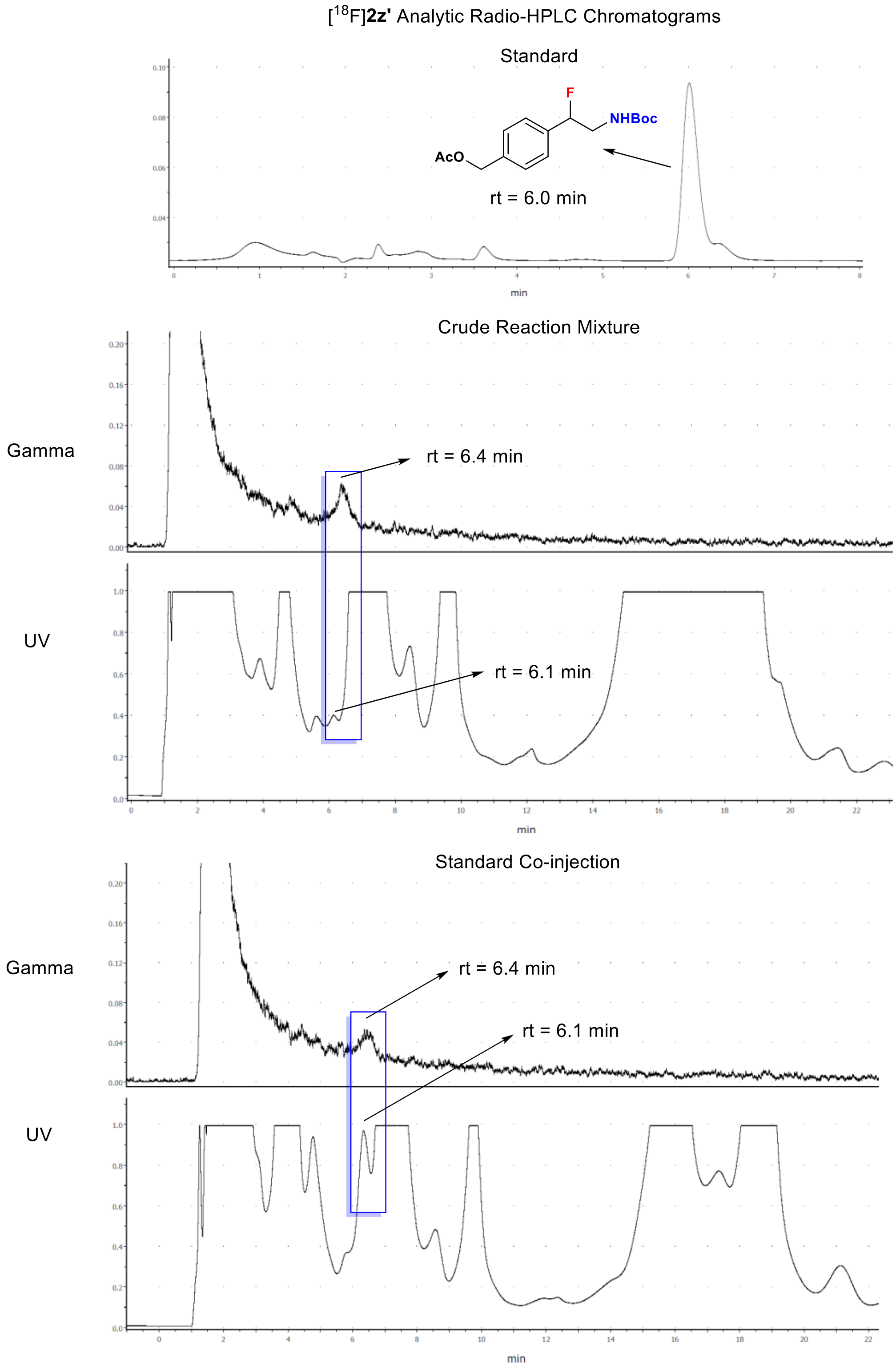

Supplementary Fig. 30. Analytical radio-HPLC spectra of **[^18F]2z'**. A Phenomenex Prodigy column (250 x 4.6 mm, 10 µm, Mobile phase, 54:46 CH_3CN:0.1% Formic Acid$_{(aq)}$, 1.35 mL/min flow, 20-80 µL injection) was used with a Prominence LC-20AT HPLC Pump equipped with a Prominence SPD-20A Absorbance detector, a Bicron frisk-tech radiation detector, and eDAQ Powerchrom software.

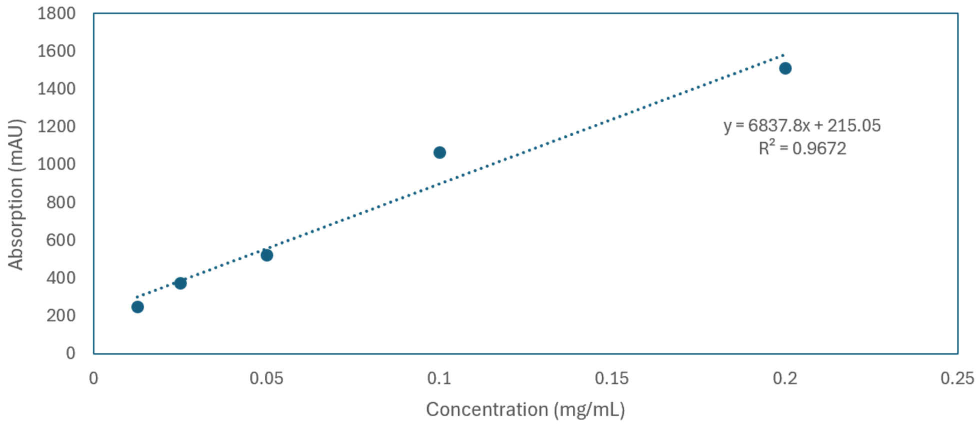

Supplementary Fig. 31. HPLC calibration curve for authentic reference **KP23**.

Supplementary Table 6. Molar activity of **[^{18}F]KP23** (n = 1).

Measurement	Activity Injected (MBq, d.c.)	Peak Area (mAu*s)	Activity Collected (MBq, d. c.)	A_m (GBq/nmol)
1	13.36	1.45×10^3	0.15	1.68

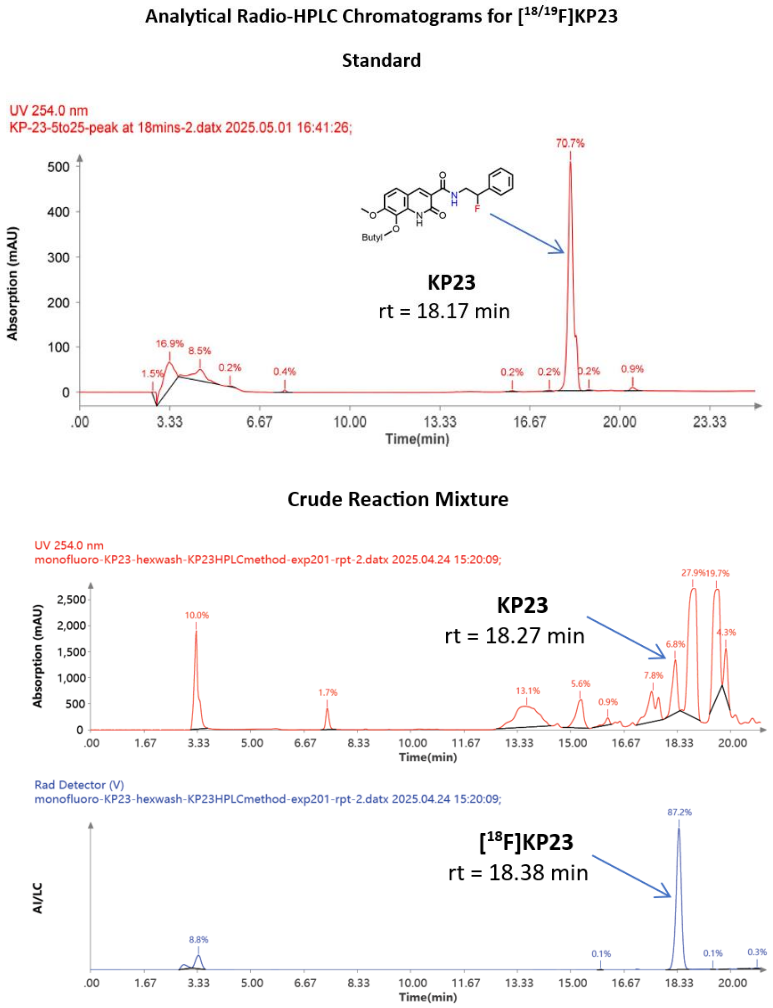

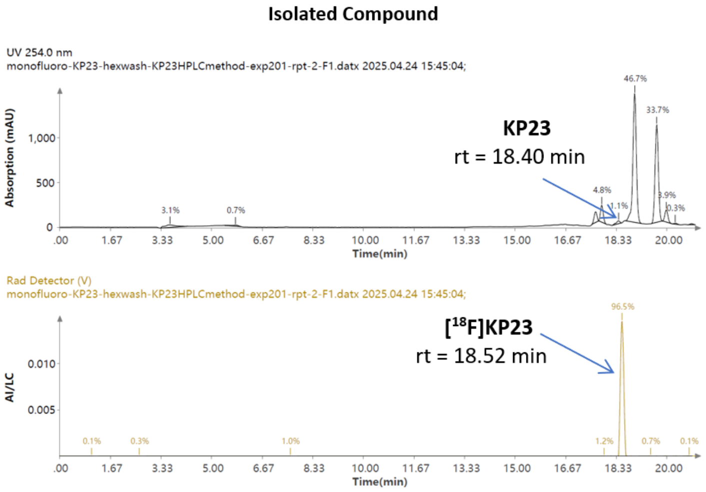

Supplementary Fig. 32. Analytical radio-HPLC spectra of **[18F]KP23**. The chromatograms were recorded using a Advion-Avant Interchim Scientific HPLC and UHPLC equipped with a Luna C(18)2 column (250 x 4.6 mm, 10 µm), A-2041 UV-DAD (2 Hz, 200 – 400 nm), A-2045/A-2046 UV-Vis DAD (20 Hz, 200 – 800 nm) and Carroll & Ramsey Associates (Model 105-S) analogue gamma detector. Mobile phase: gradient (5:95 (5 mins) then 25:75 (10 mins) and 95:5 (5 mins) CH_3CN: 0.1 M ammonium formate$_{(aq)}$, 1mL/min flow, 30-50 µL injection).

Part 8: NMR spectra

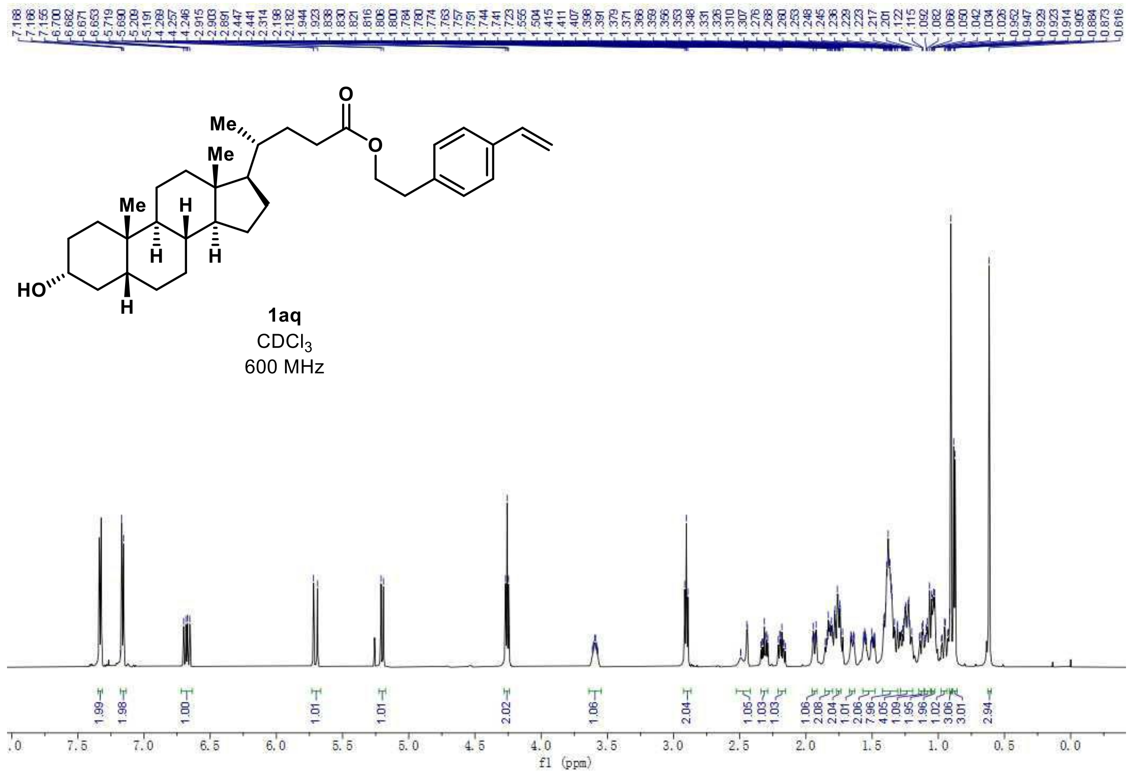

Supplementary Fig. 33. ^{1}H NMR Spectra of **1aq**

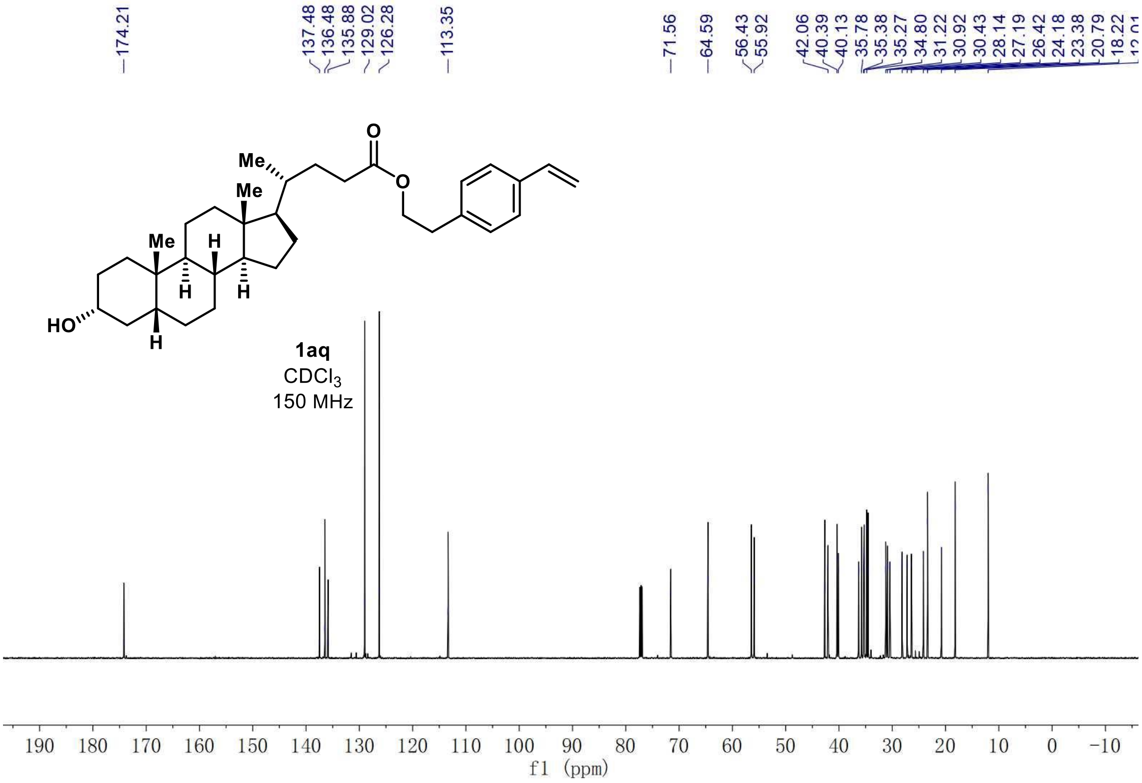

Supplementary Fig. 34. ^{13}C NMR Spectra of **1aq**

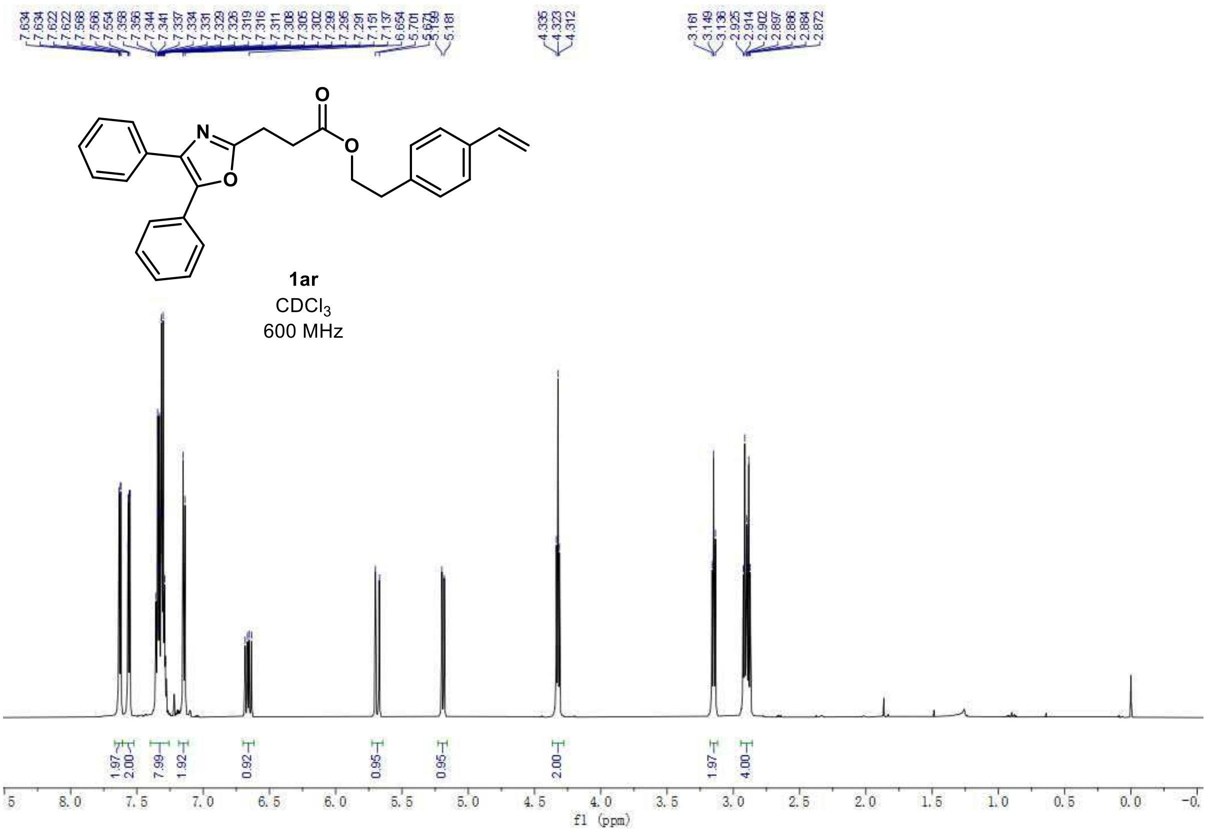

Supplementary Fig. 35. ¹H NMR Spectra of **1ar**

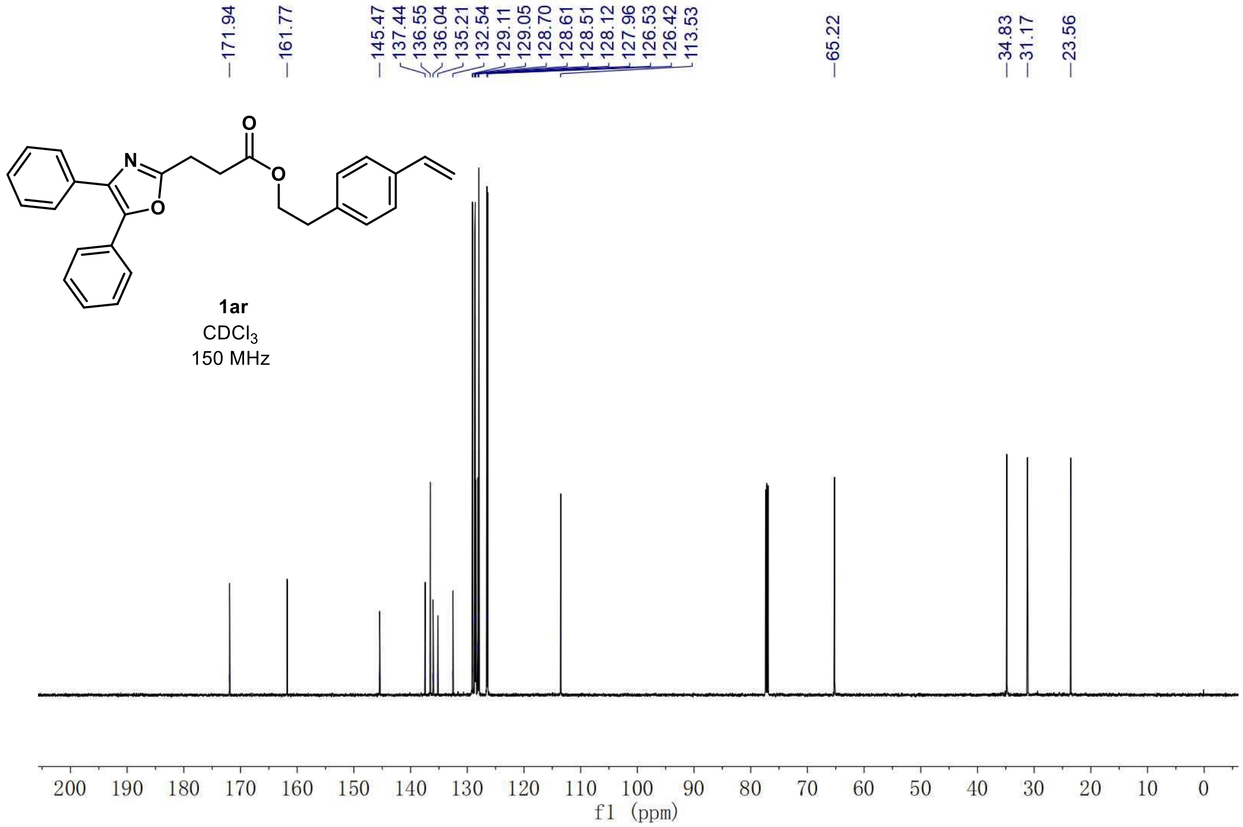

Supplementary Fig. 36. ¹³C NMR Spectra of **1ar**

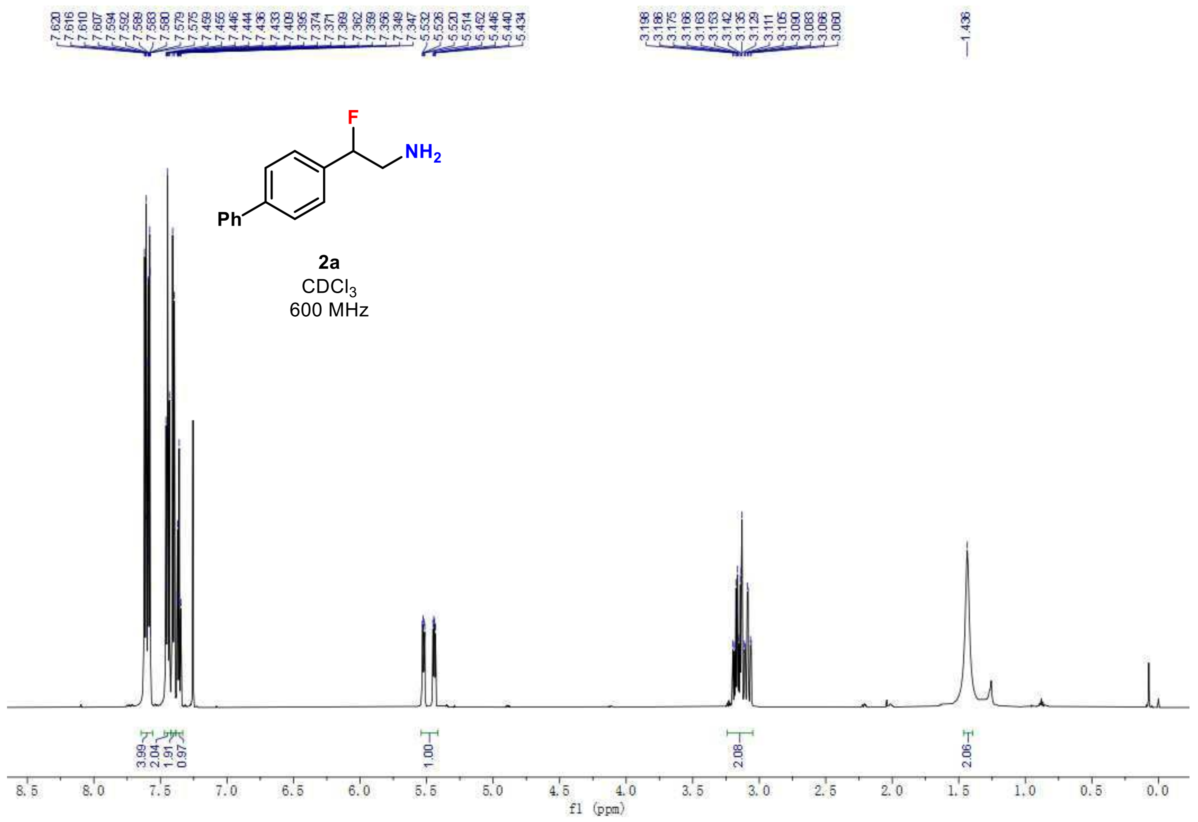

Supplementary Fig. 37. **^{1}H NMR** Spectra of **2a**

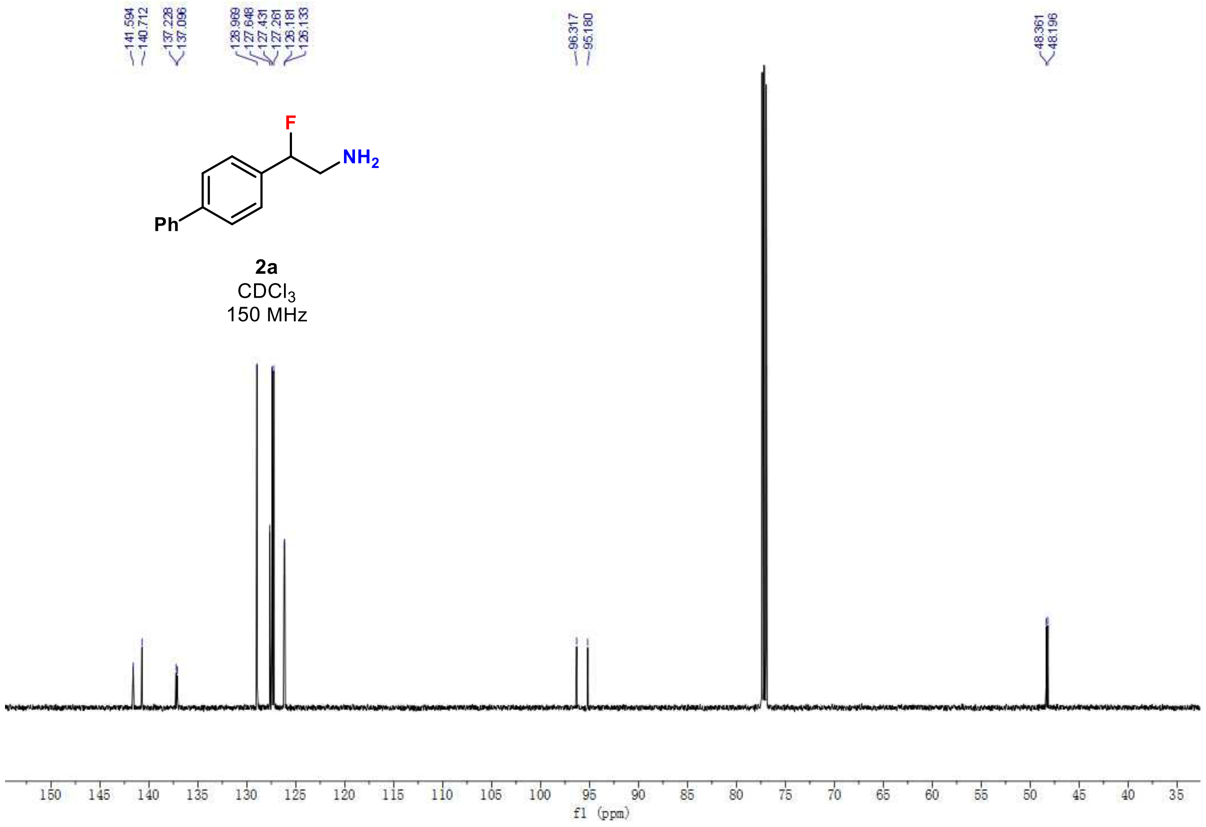

Supplementary Fig. 38. **^{13}C NMR** Spectra of **2a**

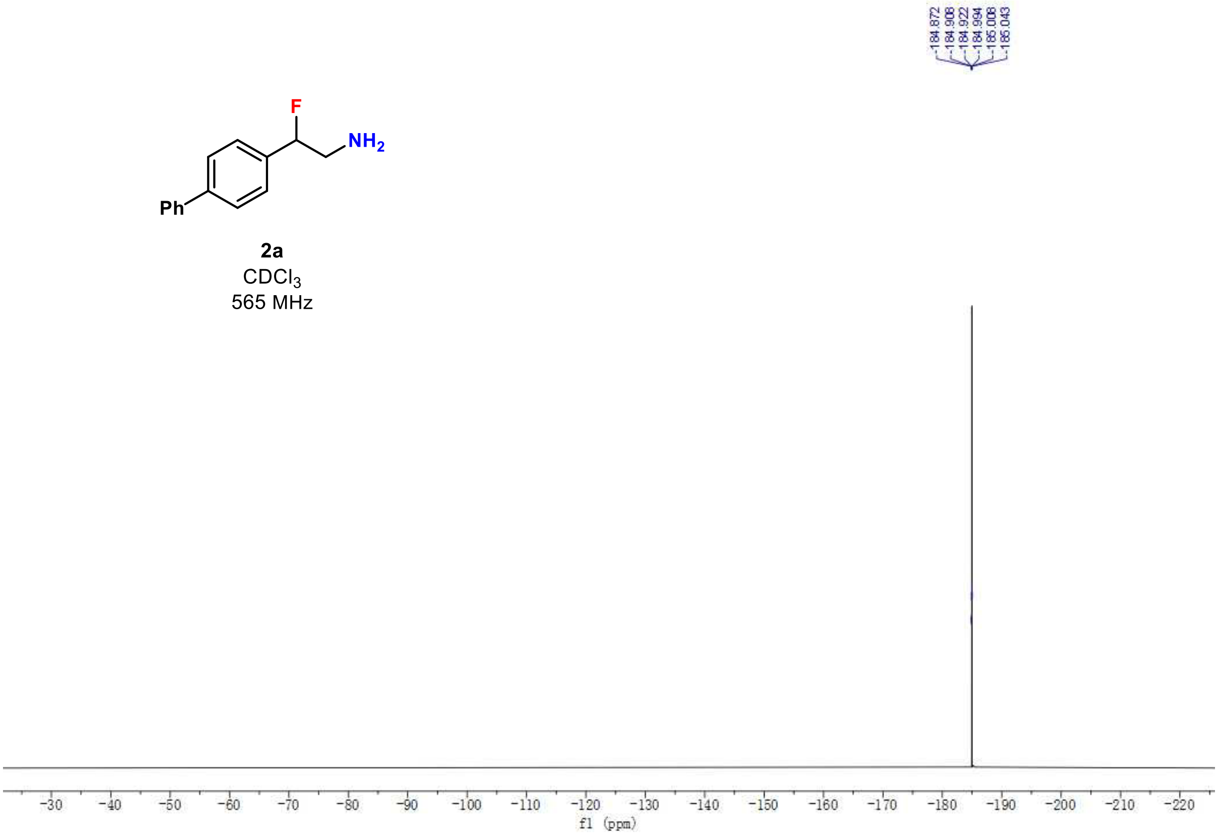

Supplementary Fig. 39. ^{19}**F NMR** Spectra of **2a**

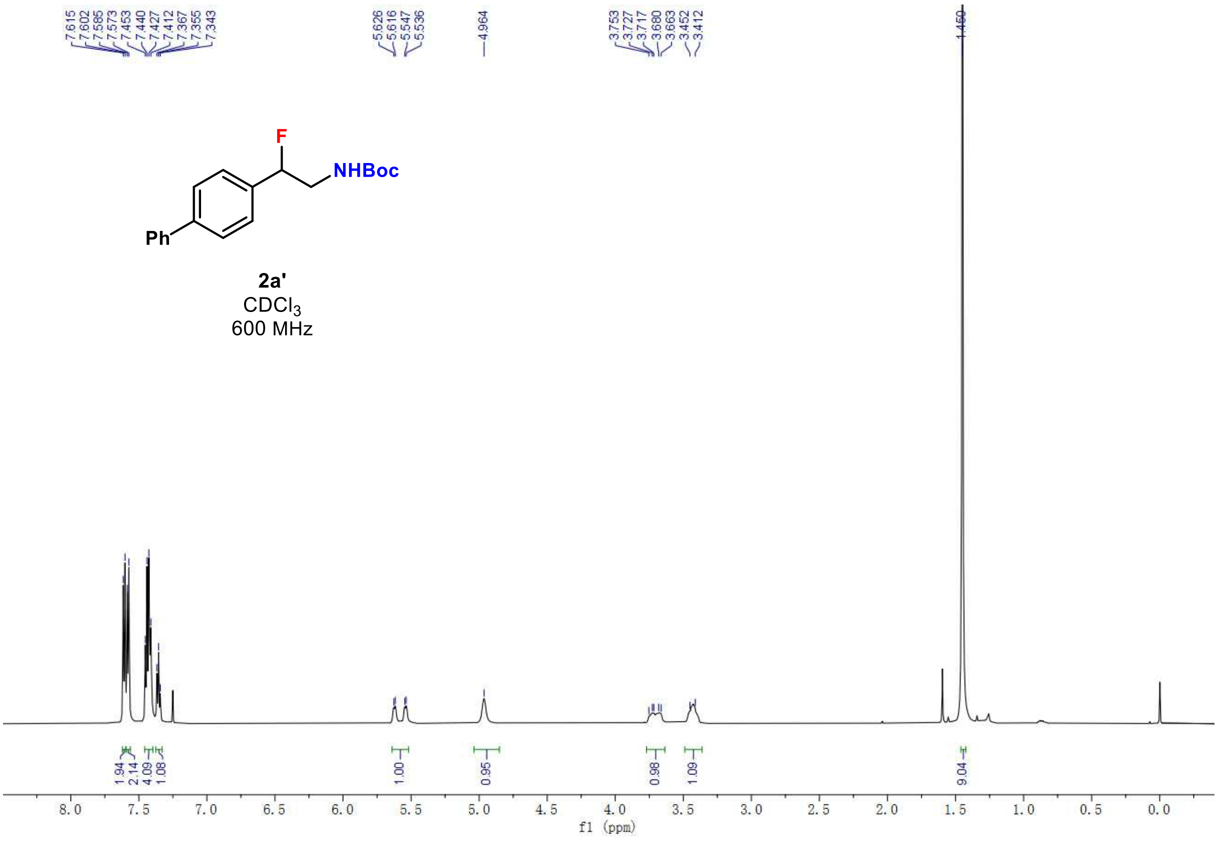

Supplementary Fig. 40. ^{1}**H NMR** Spectra of **2a'**

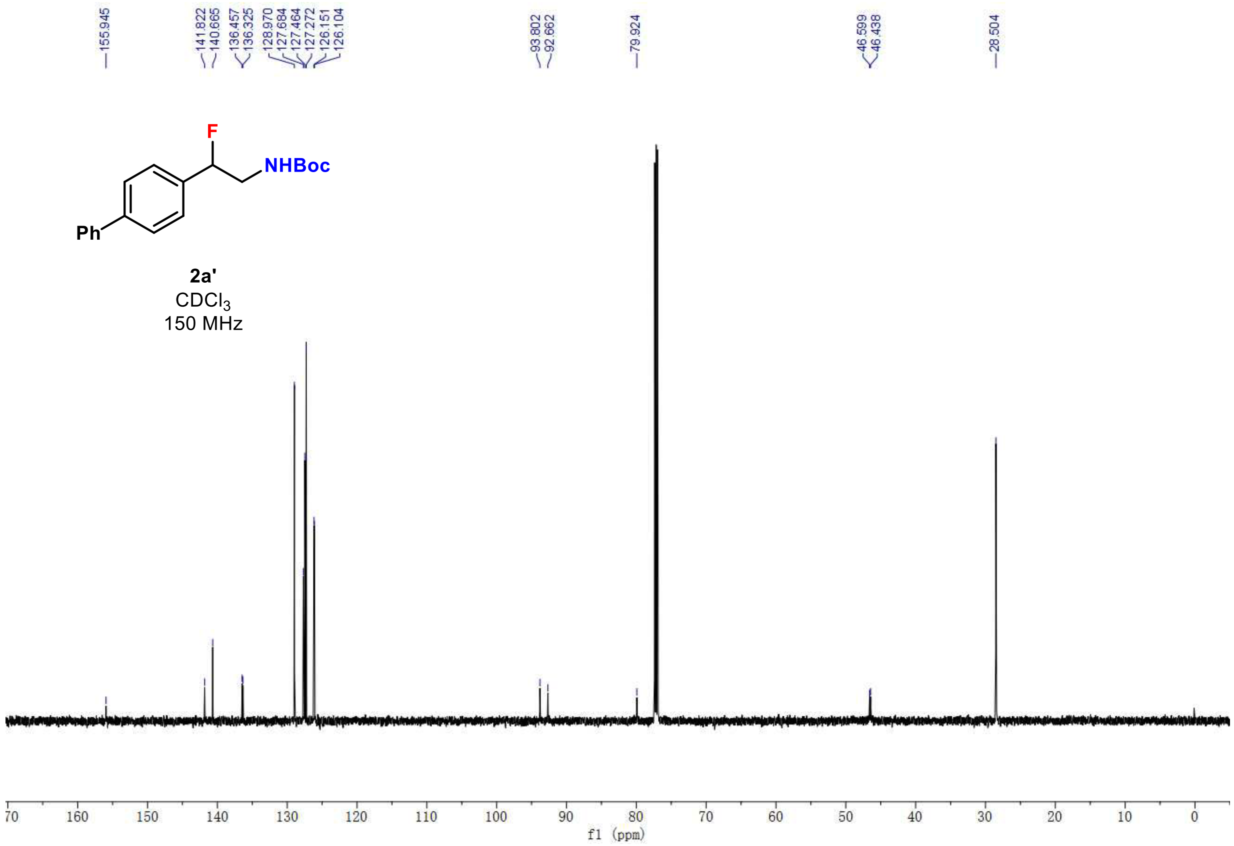

Supplementary Fig. 41. ^{13}**C NMR** Spectra of **2a'**

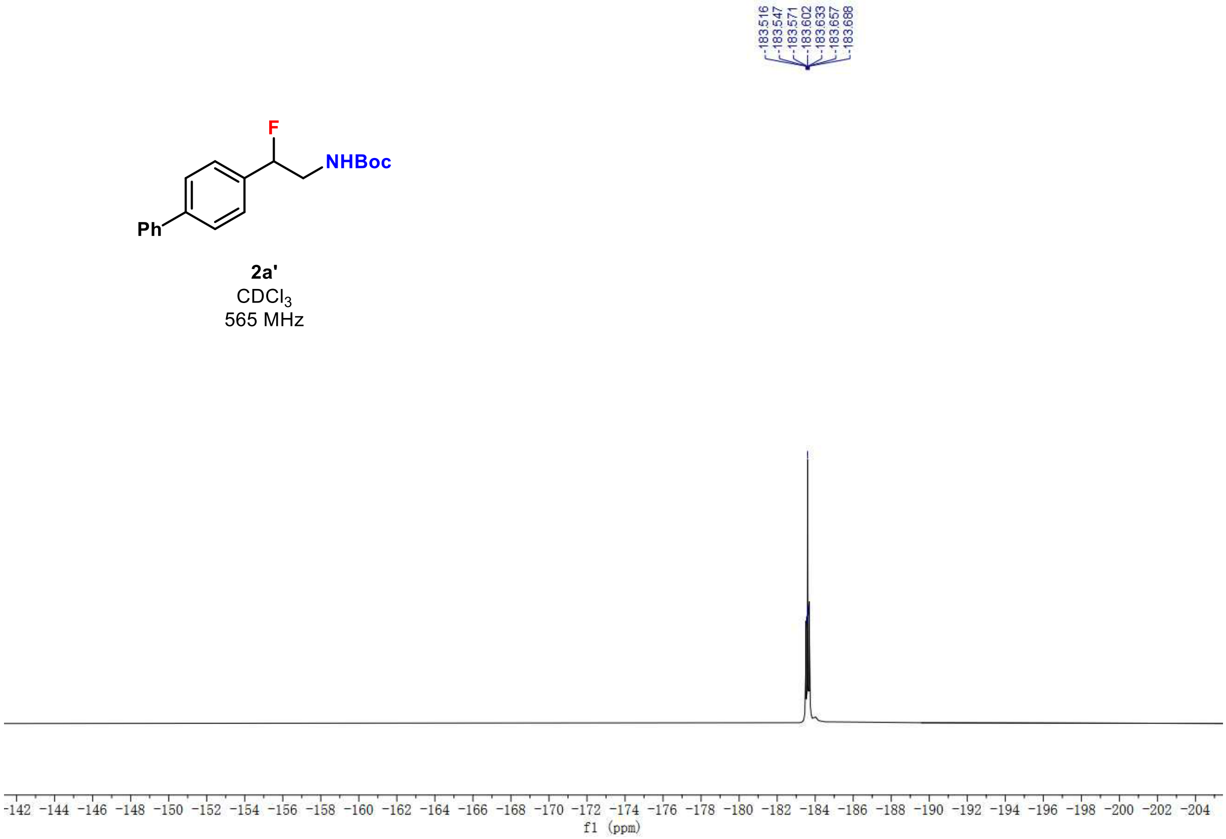

Supplementary Fig. 42. ^{19}**F NMR** Spectra of **2a'**

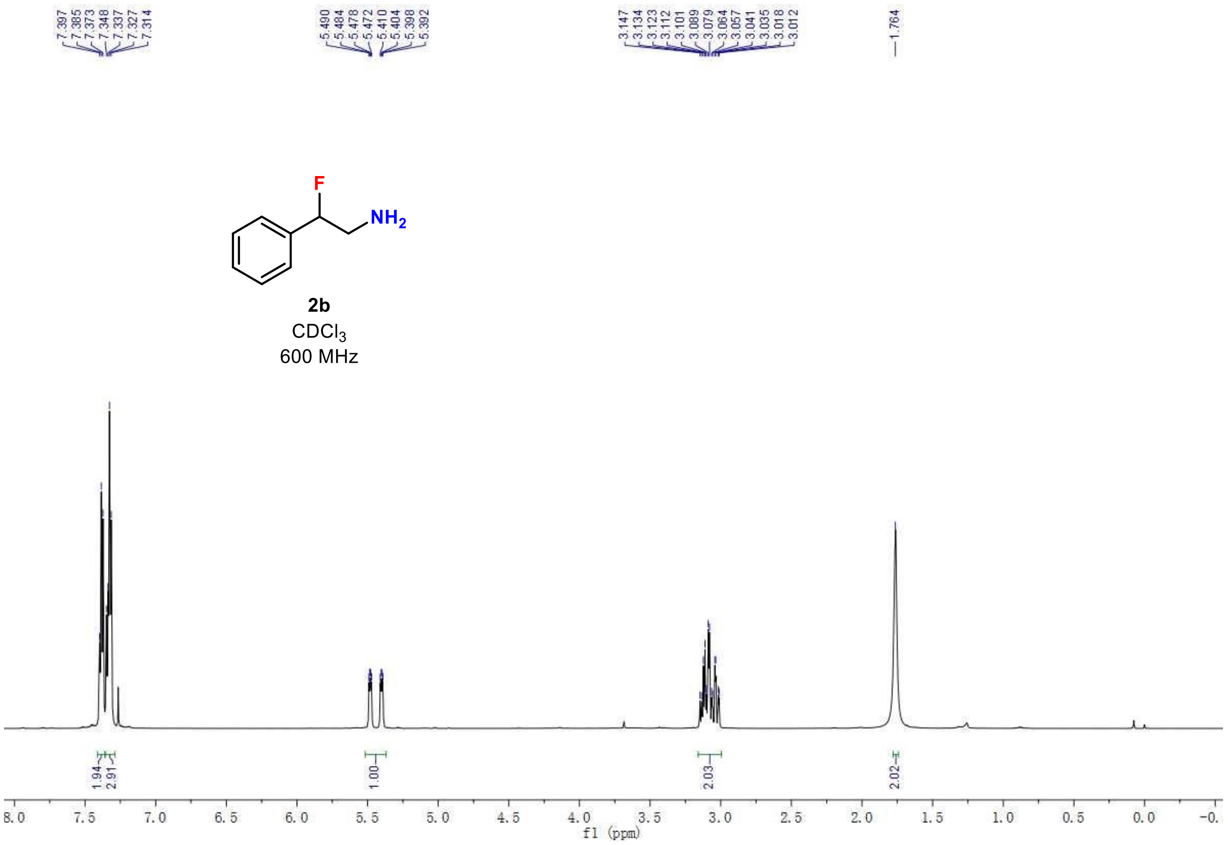

Supplementary Fig. 43. ^{1}**H NMR** Spectra of **2b**

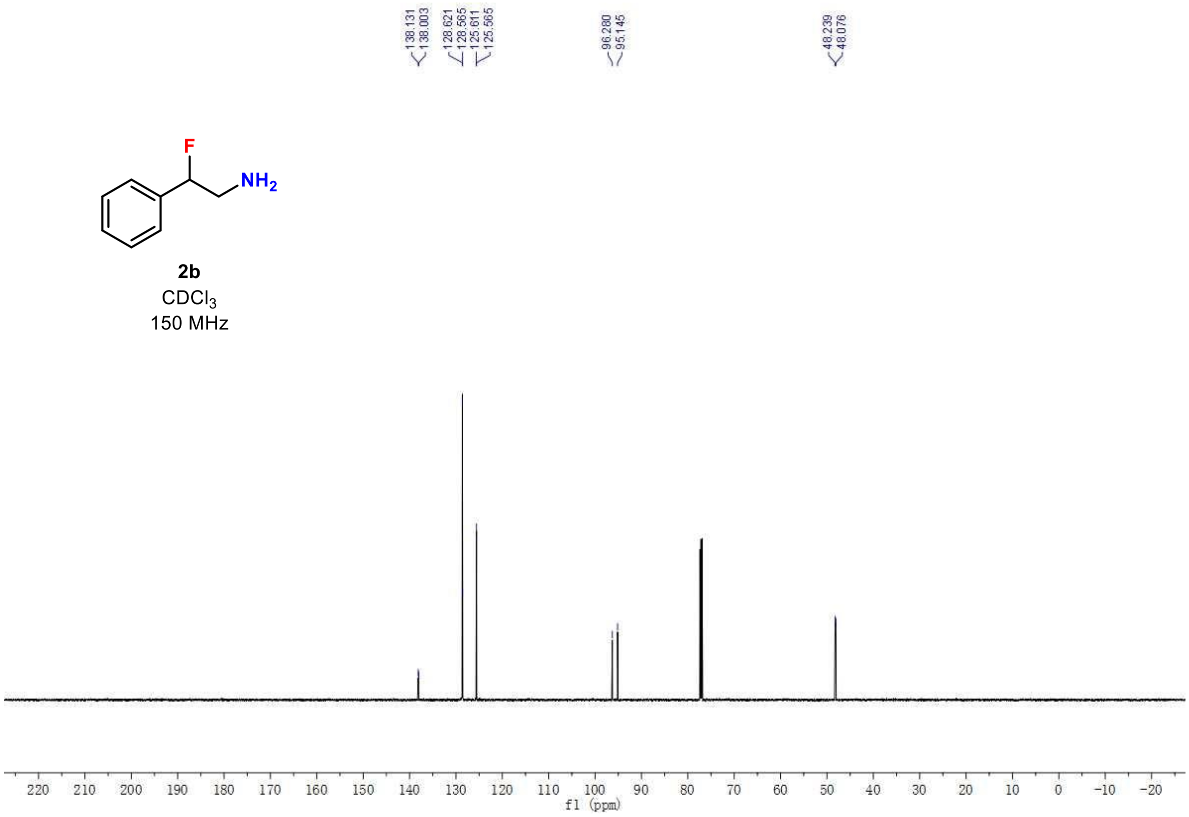

Supplementary Fig. 44. ^{13}**C NMR** Spectra of **2b**

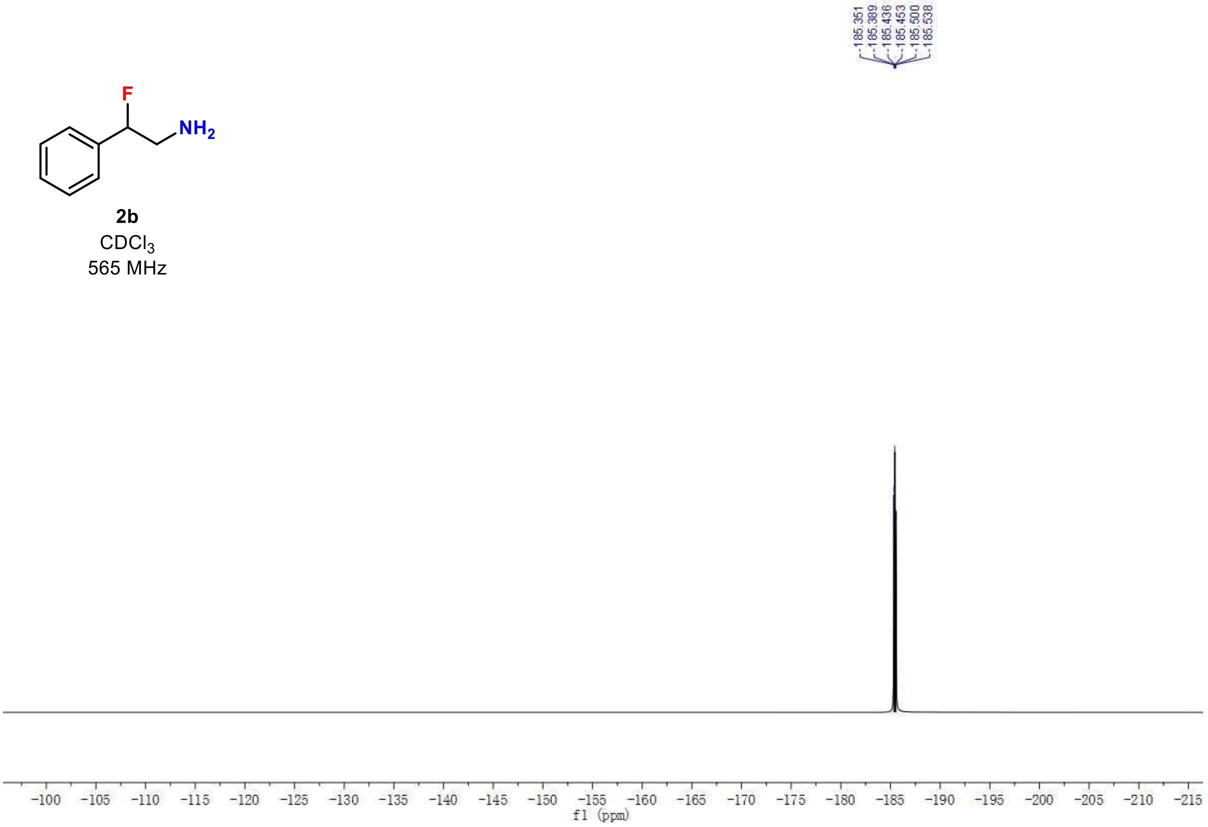

Supplementary Fig. 45. ^{19}**F NMR** Spectra of **2b**

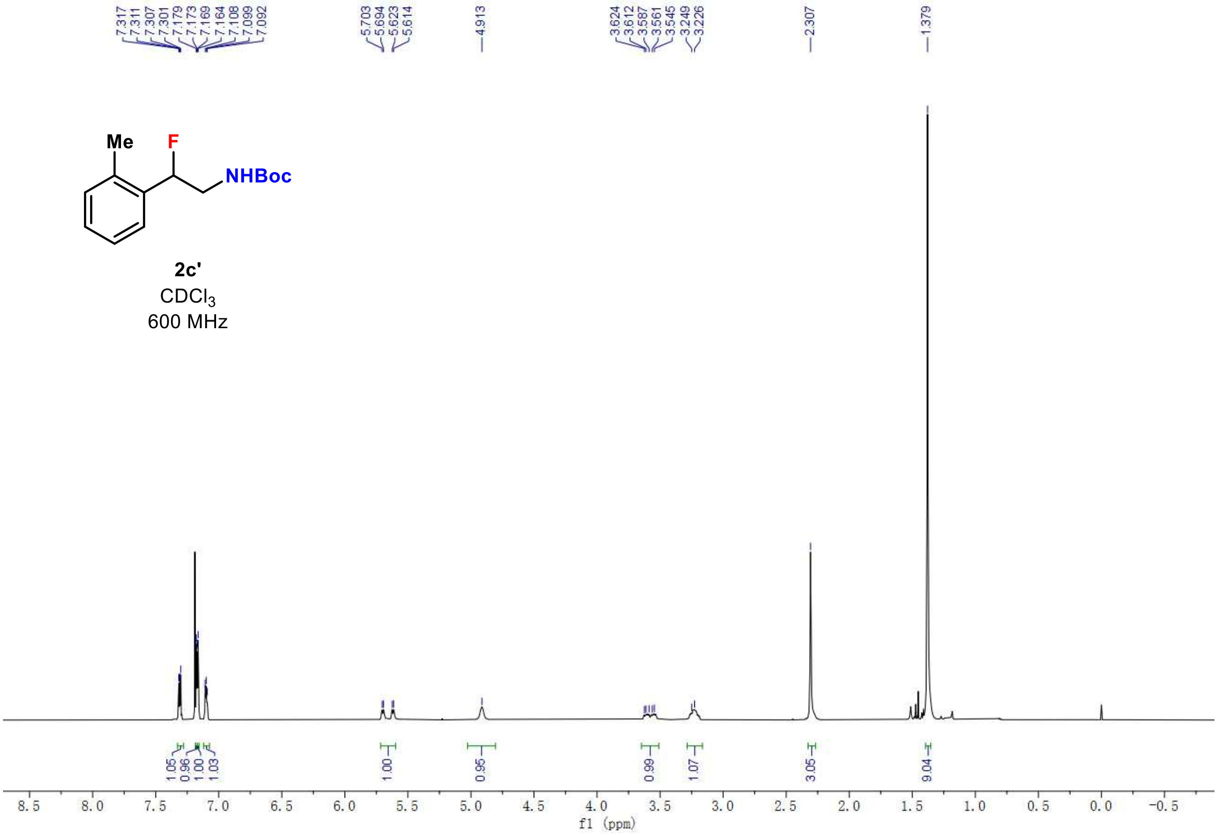

Supplementary Fig. 46. ^{1}**H NMR** Spectra of **2c'**

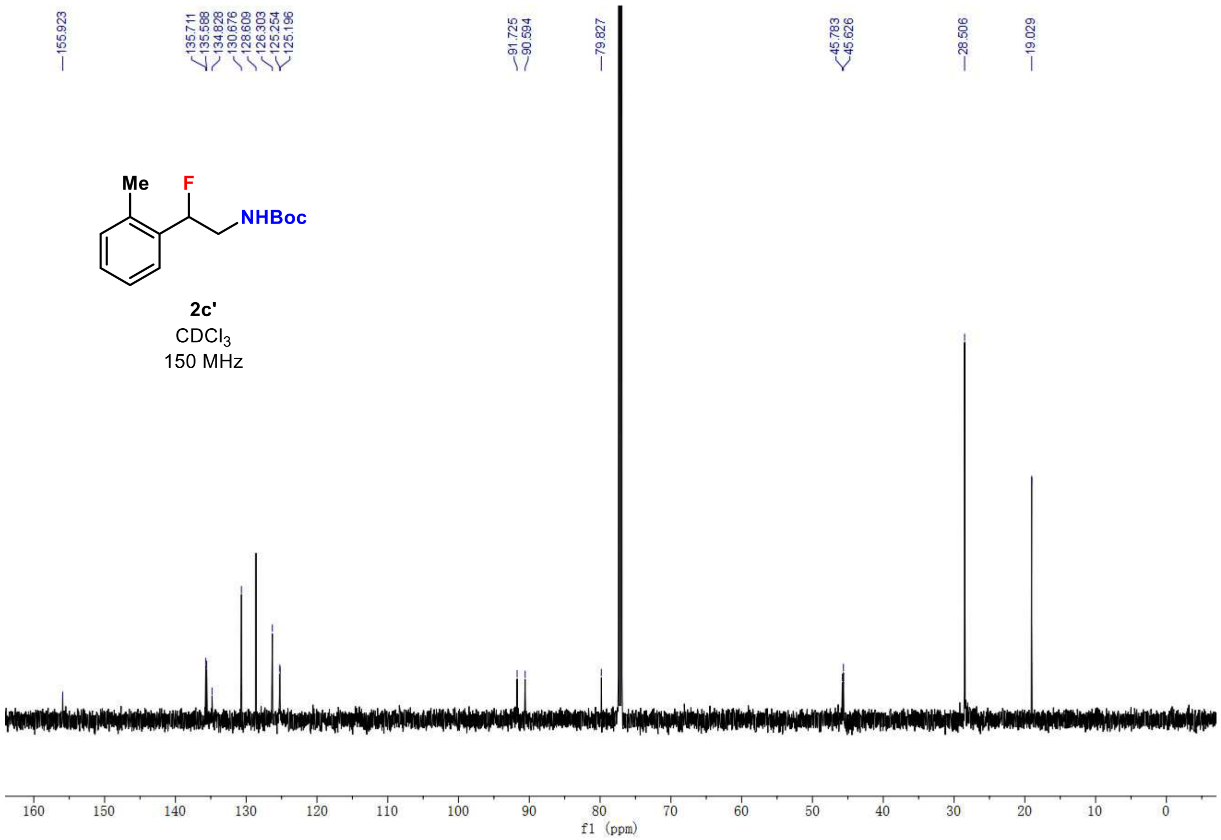

Supplementary Fig. 47. ^{13}**C NMR** Spectra of **2c'**

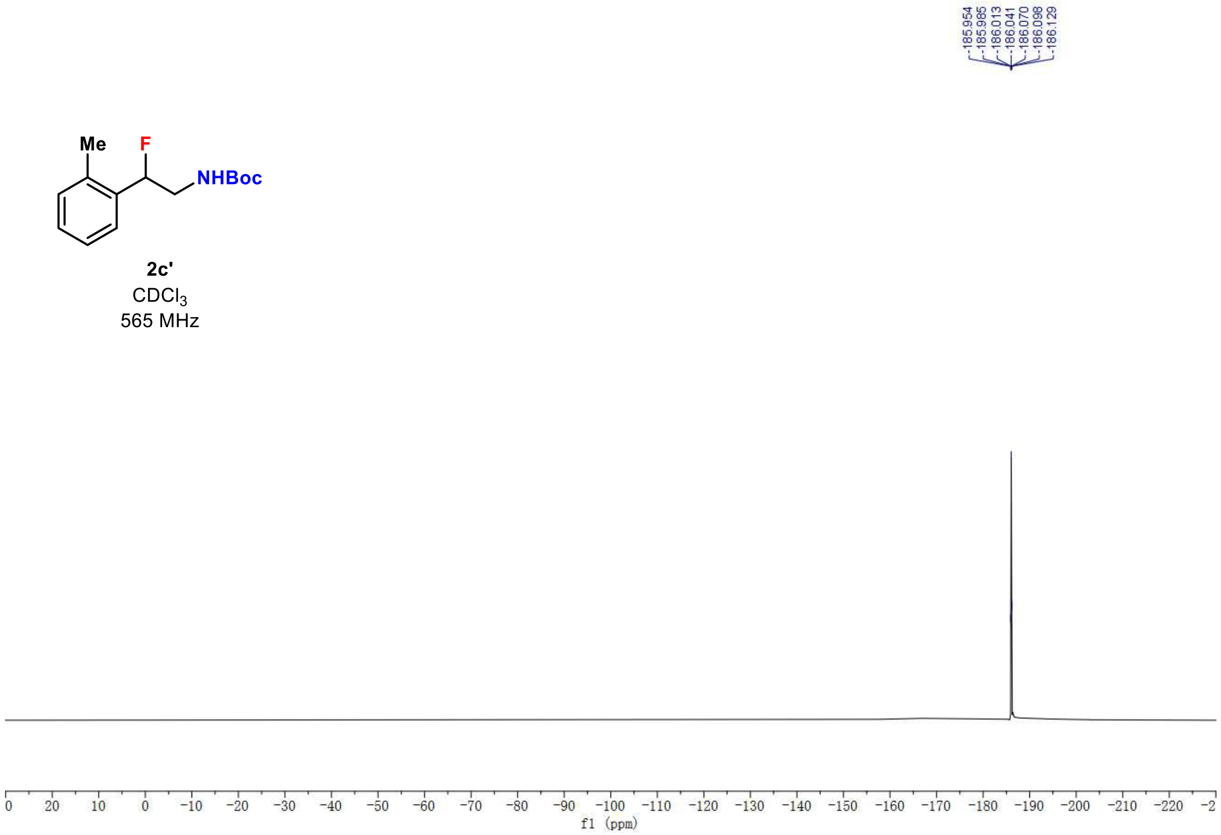

Supplementary Fig. 48. ^{19}**F NMR** Spectra of **2c'**

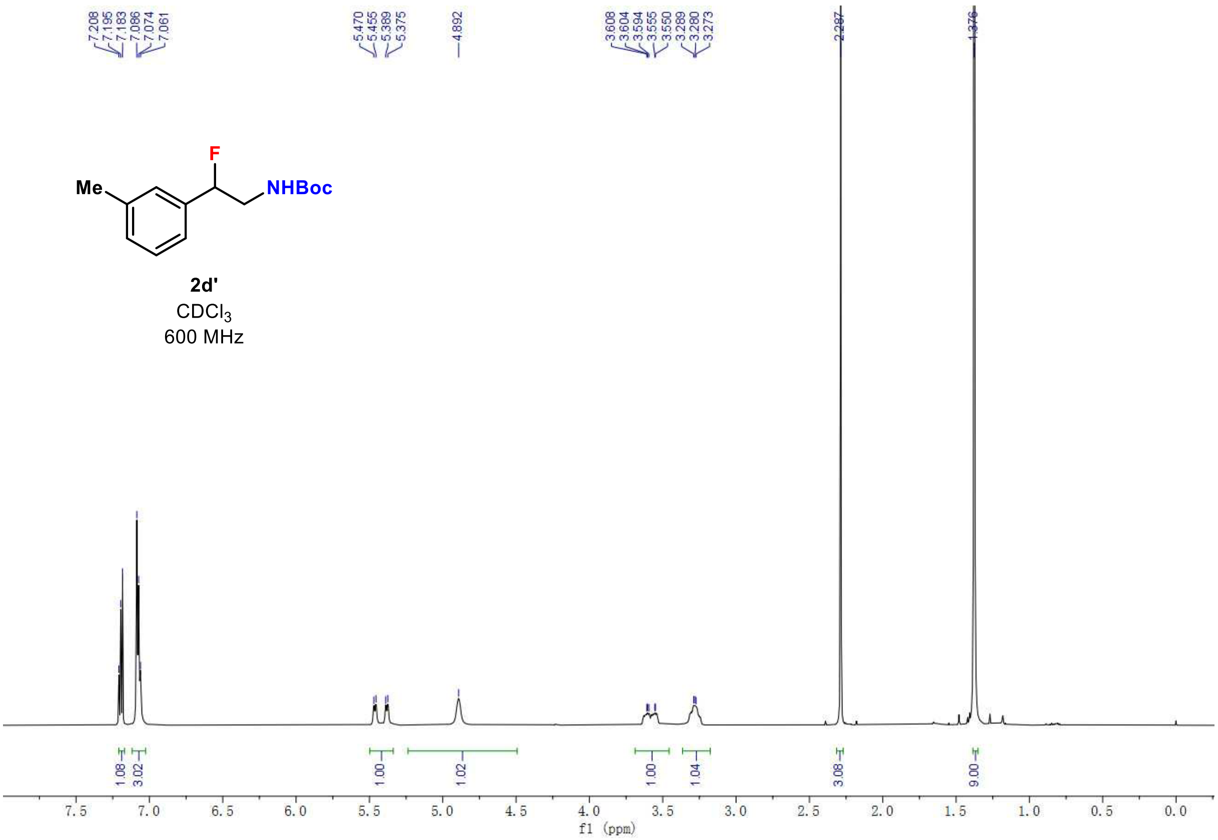

Supplementary Fig. 49. ^{1}**H NMR** Spectra of **2d'**

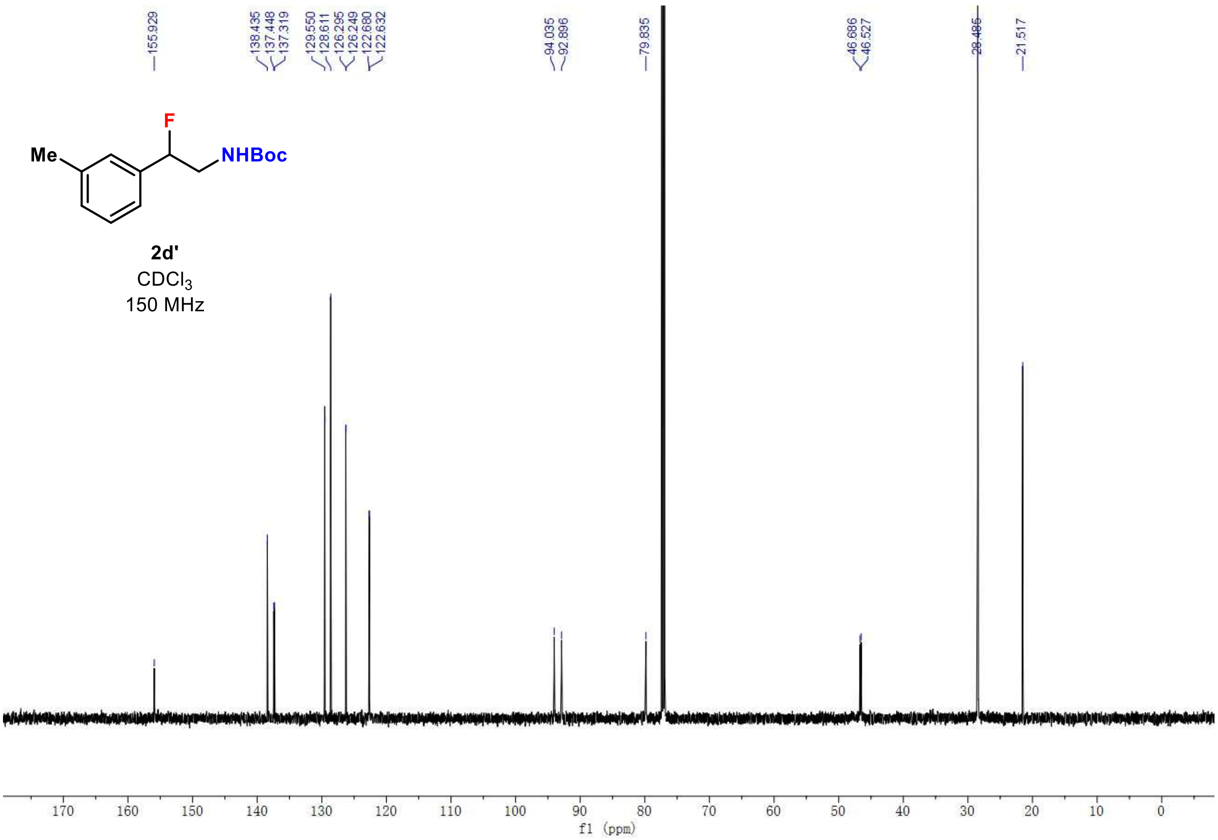

Supplementary Fig. 50. ^{13}**C NMR** Spectra of **2d'**

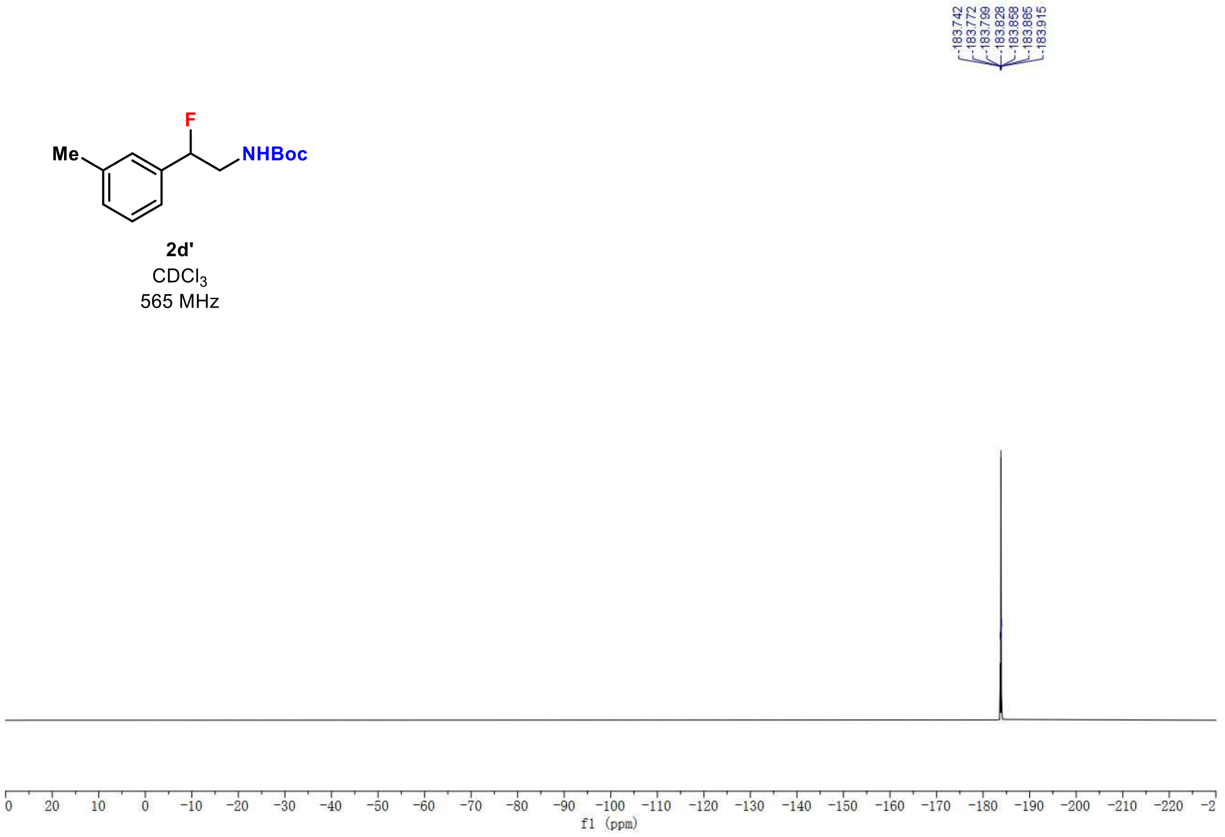

Supplementary Fig. 51. **^{19}F NMR** Spectra of **2d'**

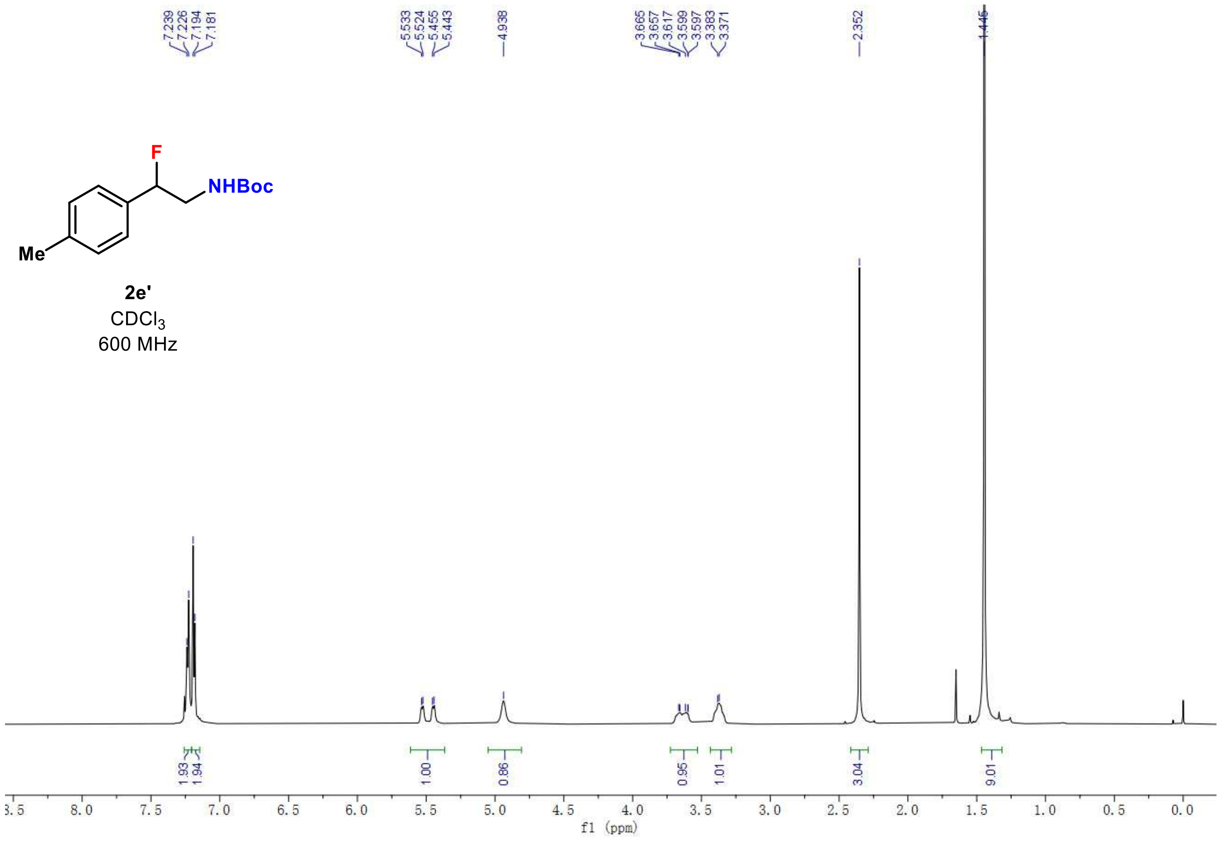

Supplementary Fig. 52. **^{1}H NMR** Spectra of **2e'**

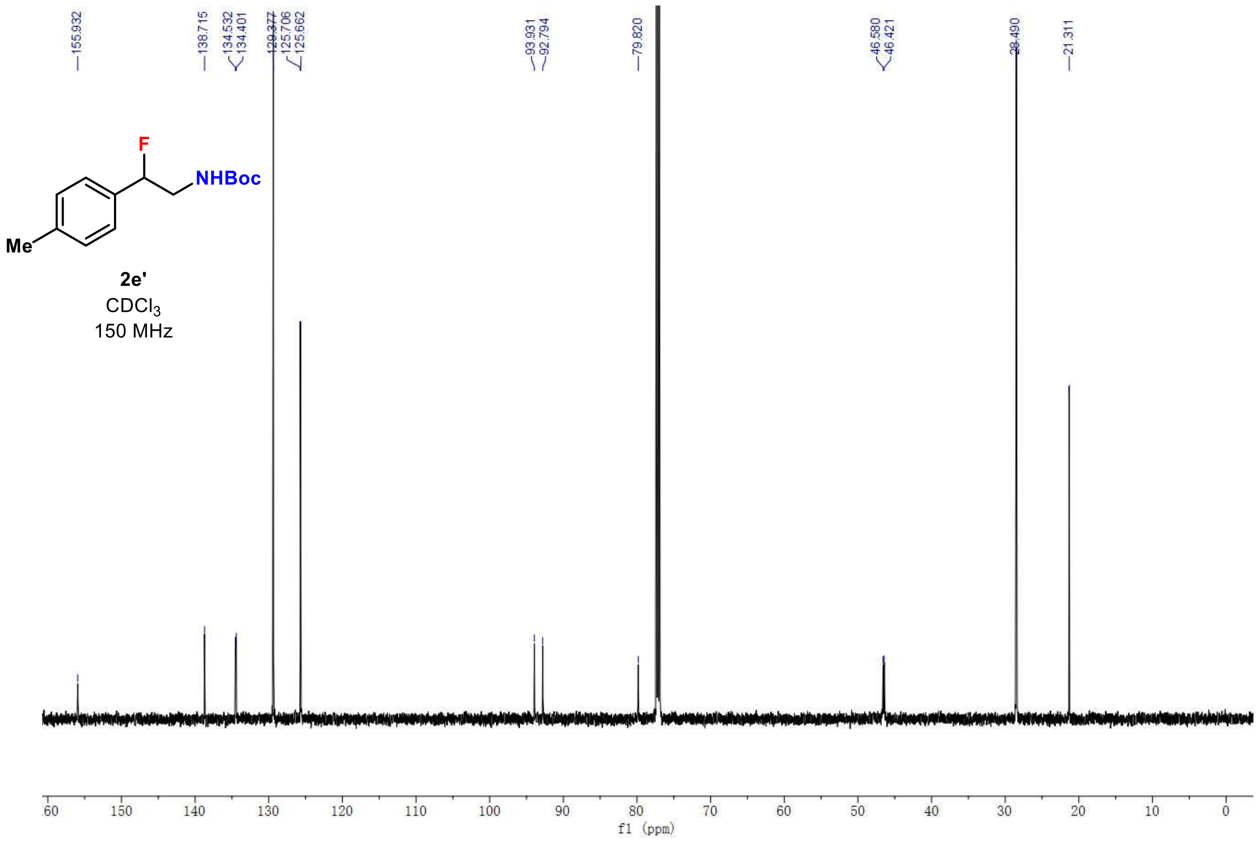

Supplementary Fig. 53. ^{13}**C NMR** Spectra of **2e'**

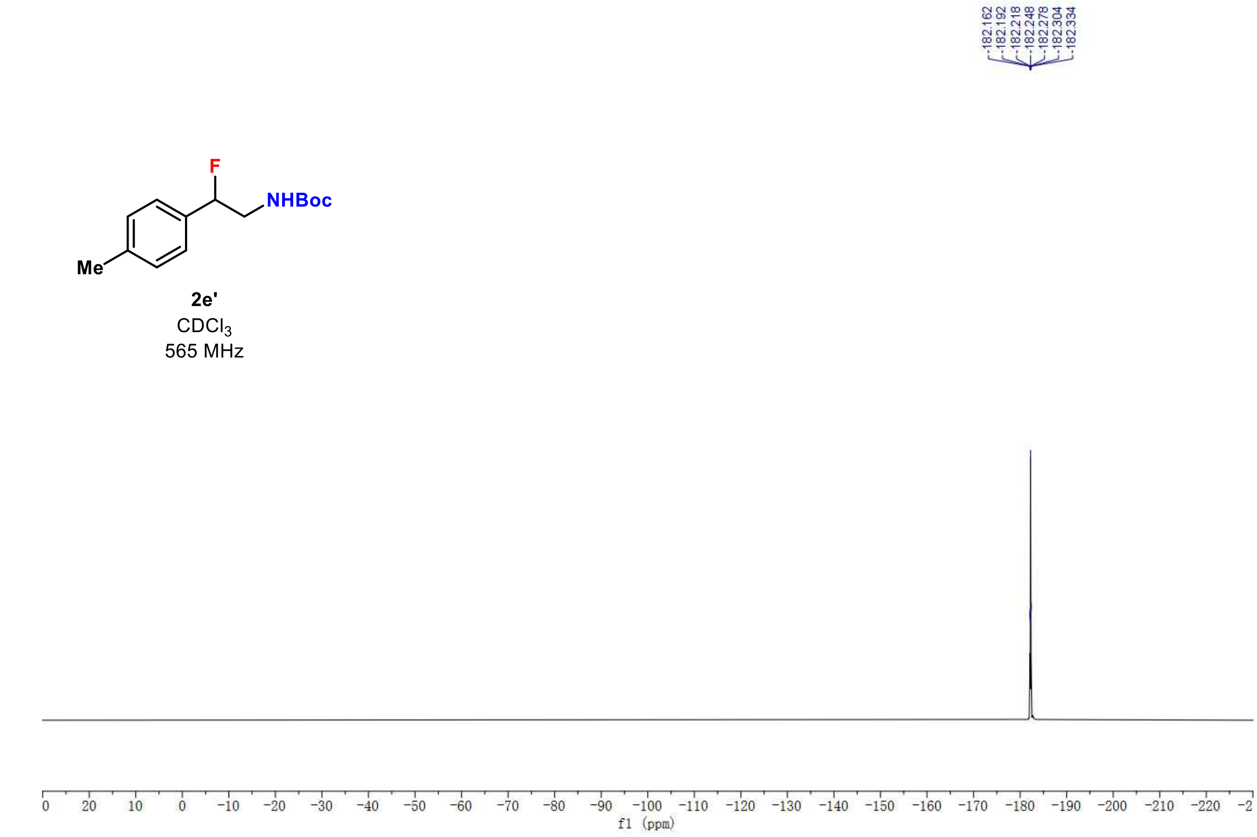

Supplementary Fig. 54. ^{19}**F NMR** Spectra of **2e'**

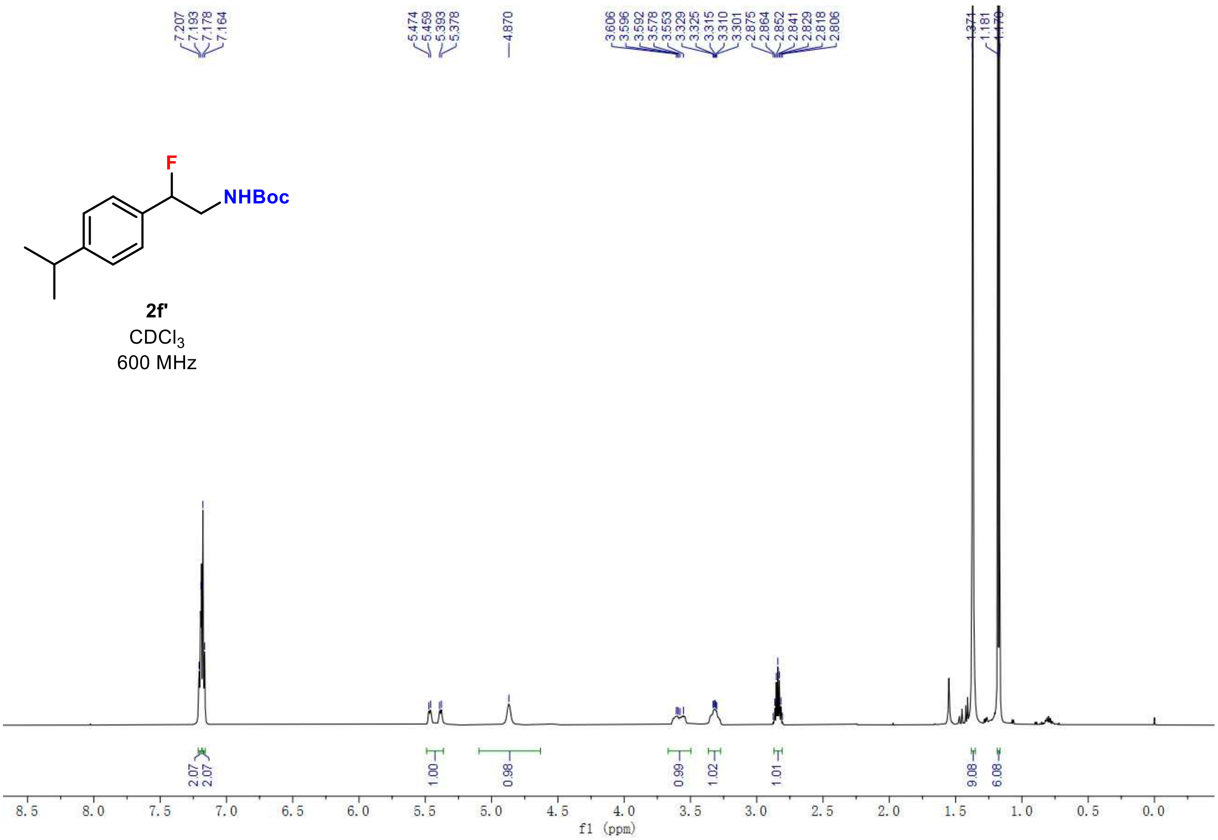

Supplementary Fig. 55. ^{1}H NMR Spectra of 2f'

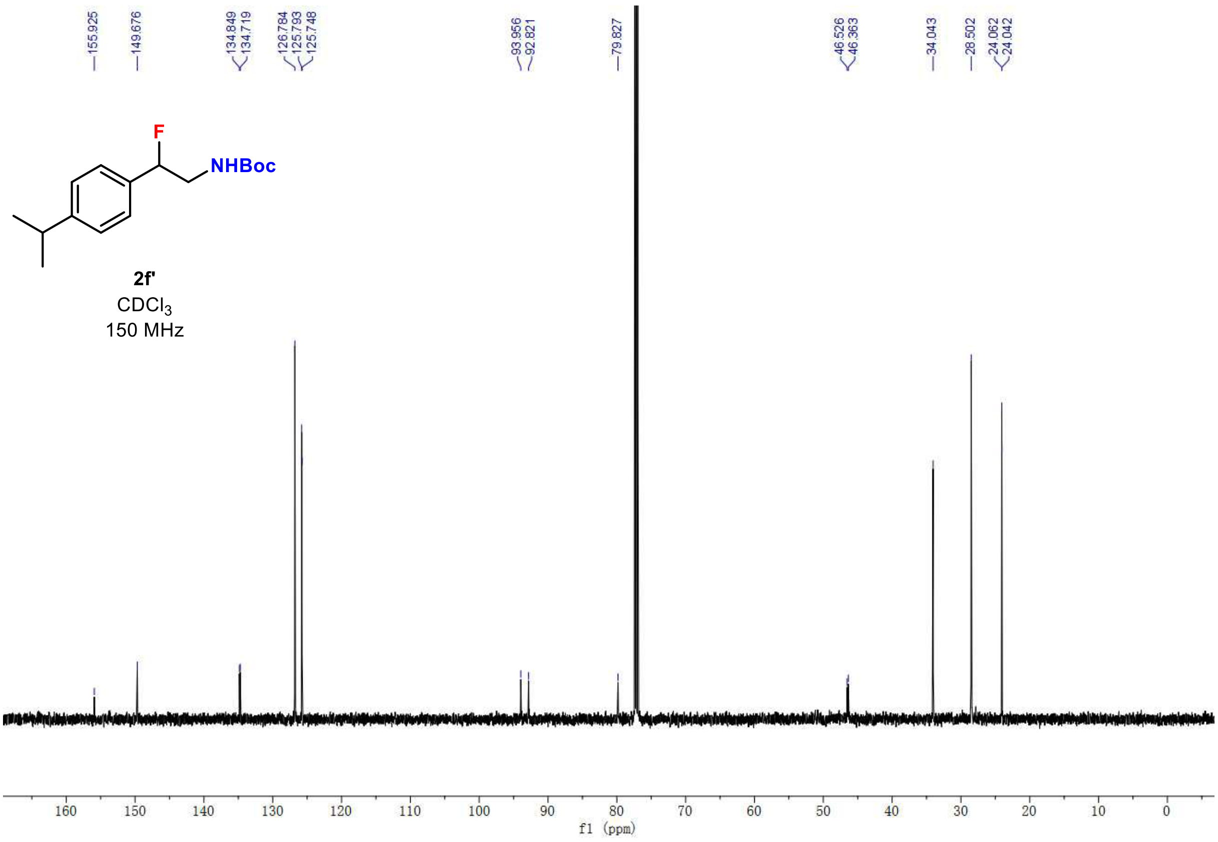

Supplementary Fig. 56. ^{13}C NMR Spectra of 2f'

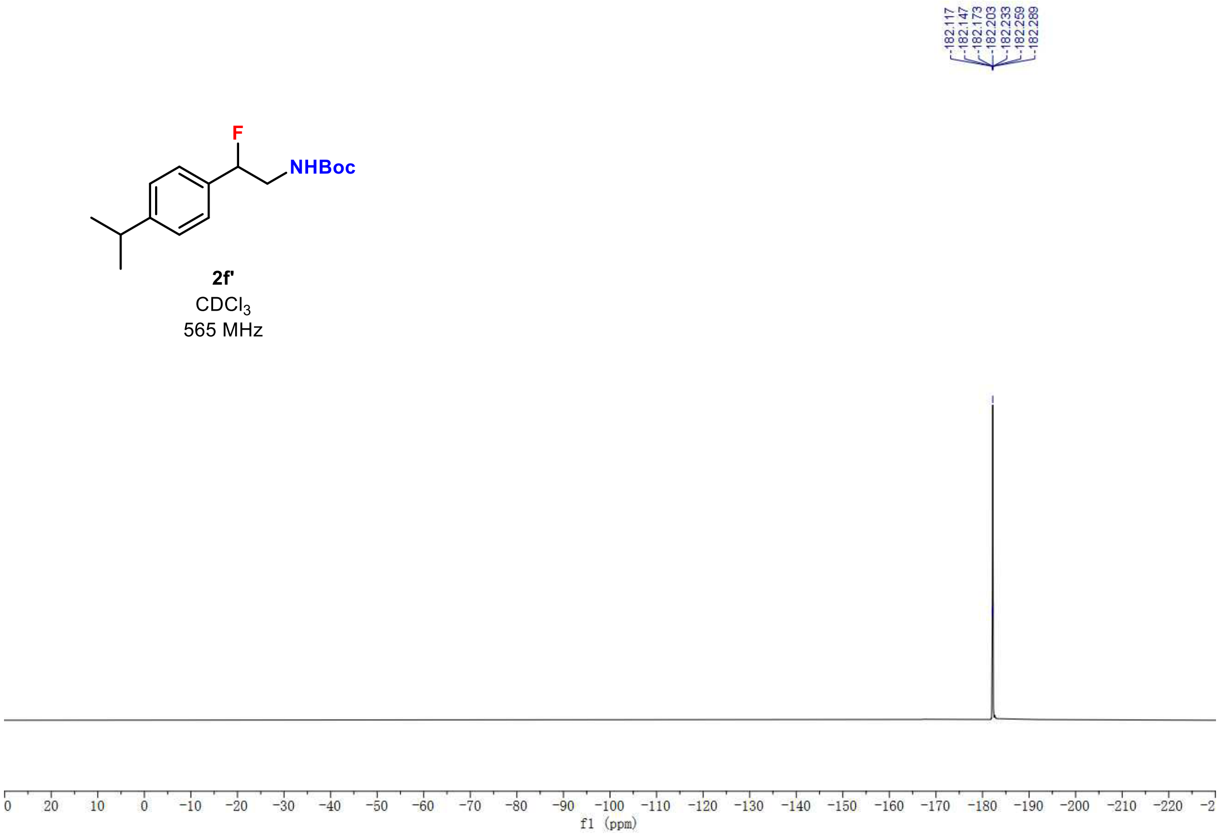

Supplementary Fig. 57. **^{19}F NMR** Spectra of **2f'**

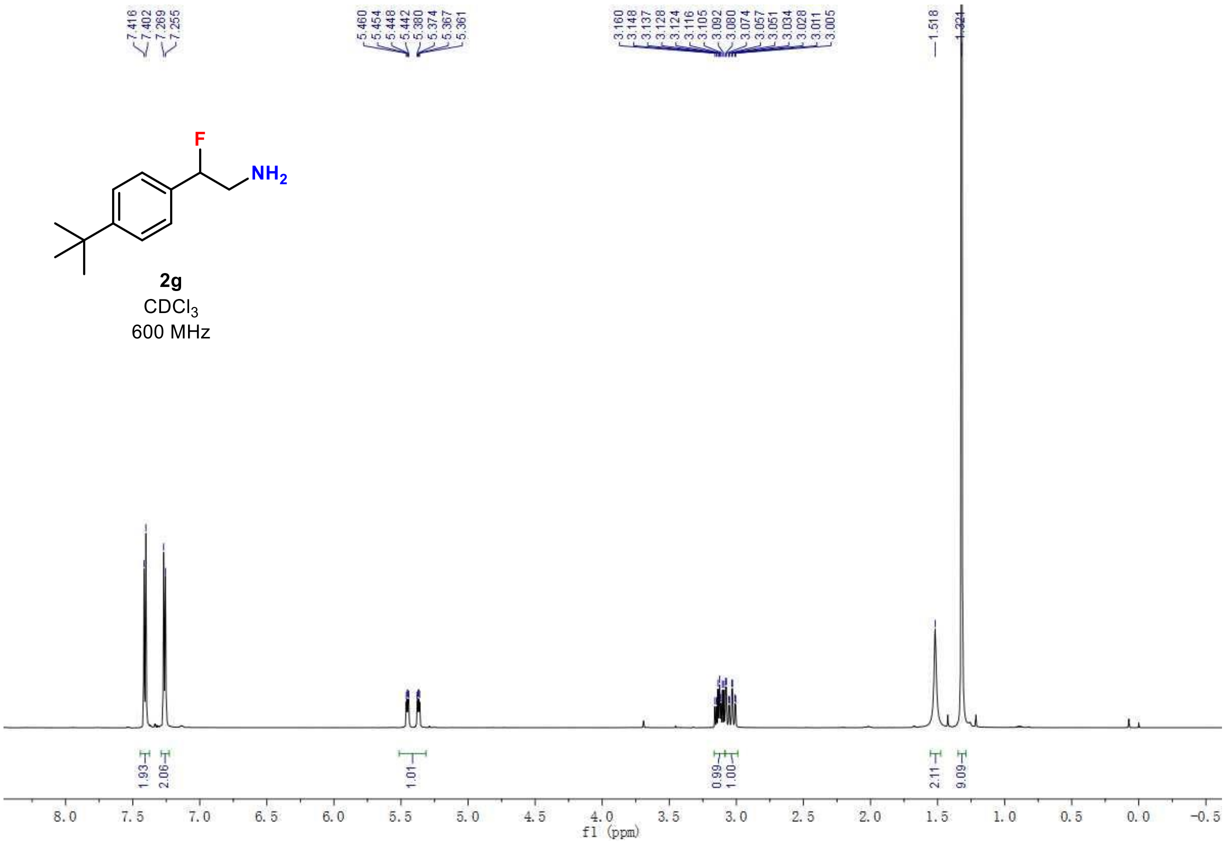

Supplementary Fig. 58. **^{1}H NMR** Spectra of **2g**

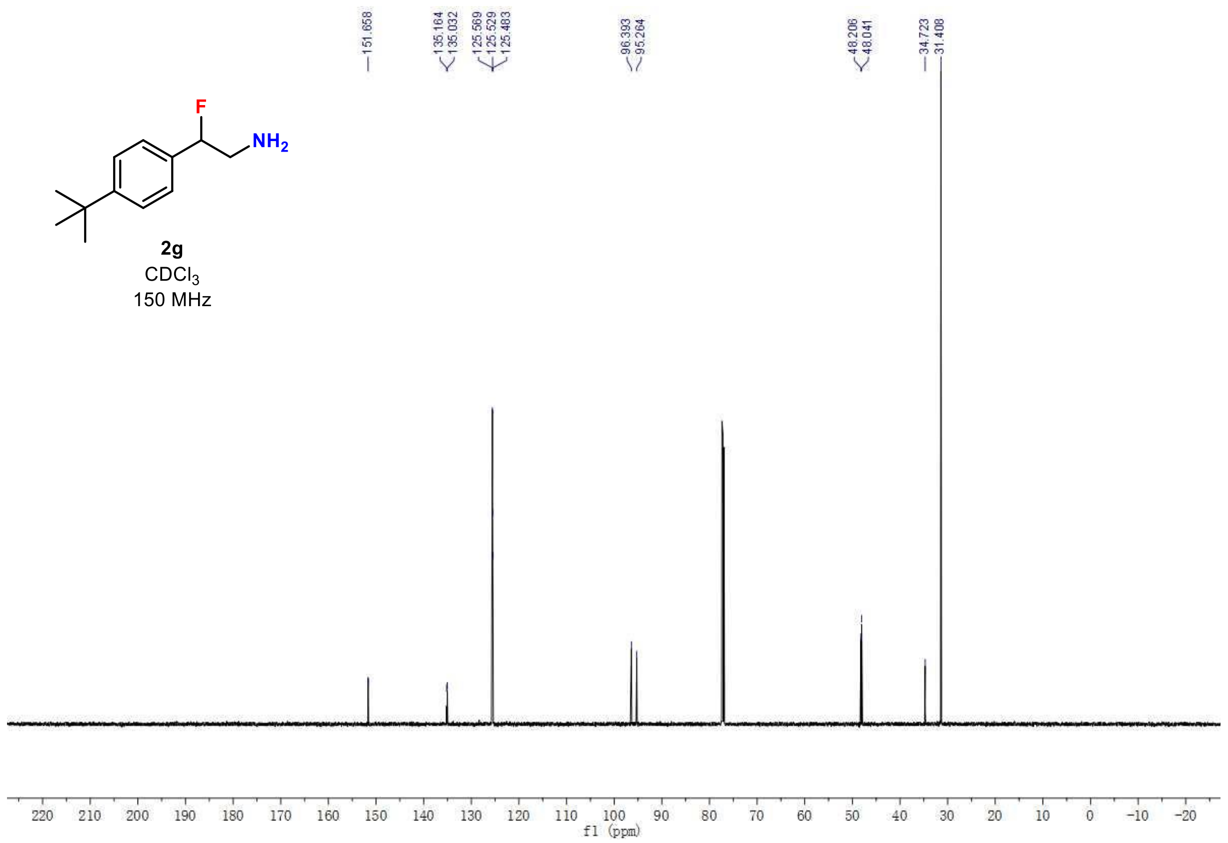

Supplementary Fig. 59. ^{13}**C NMR** Spectra of **2g**

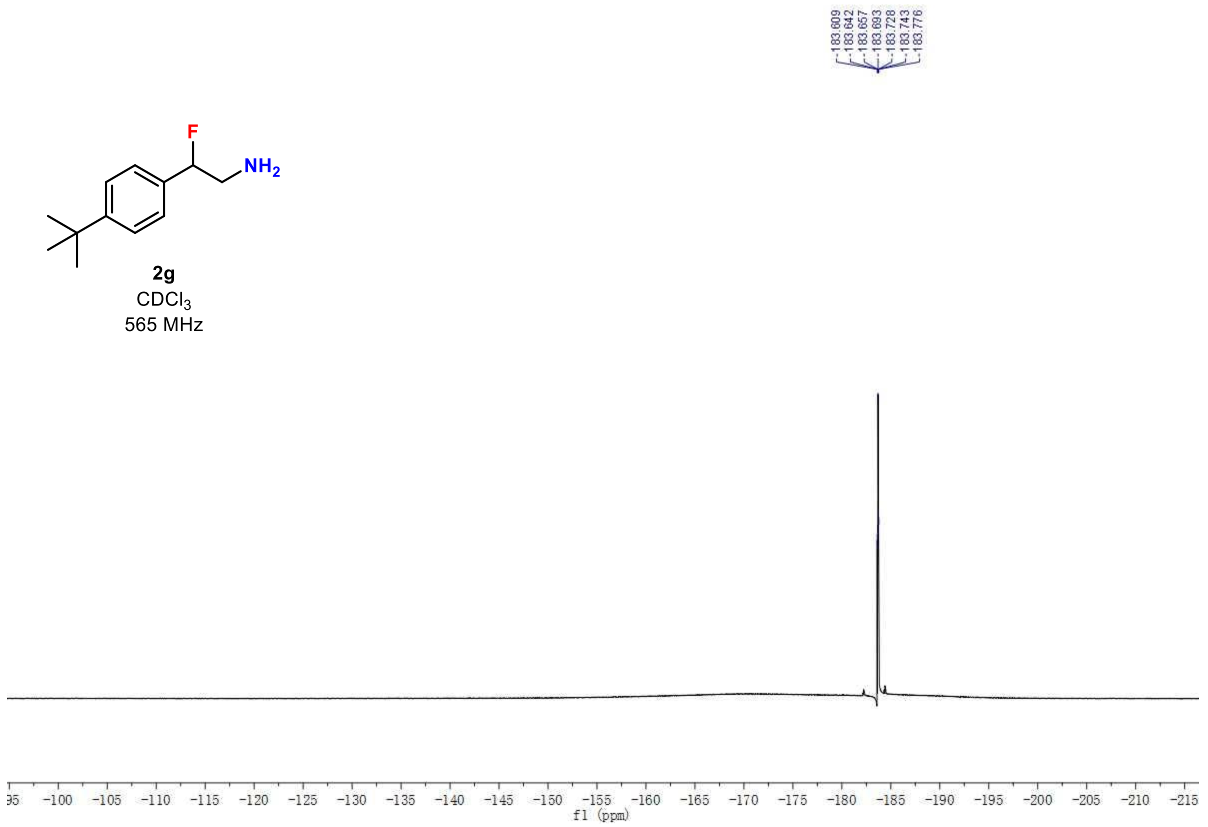

Supplementary Fig. 60. ^{19}**F NMR** Spectra of **2g**

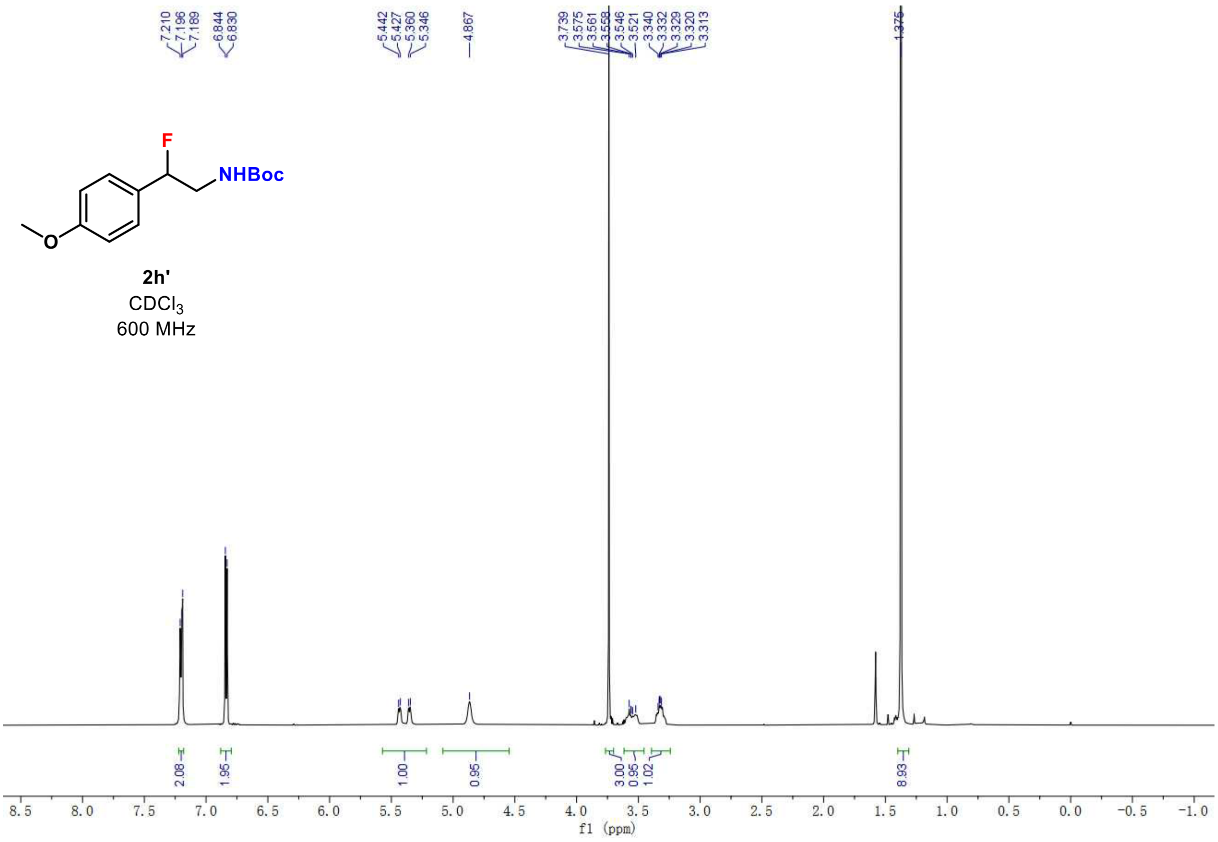

Supplementary Fig. 61. **^{1}H NMR** Spectra of **2h'**

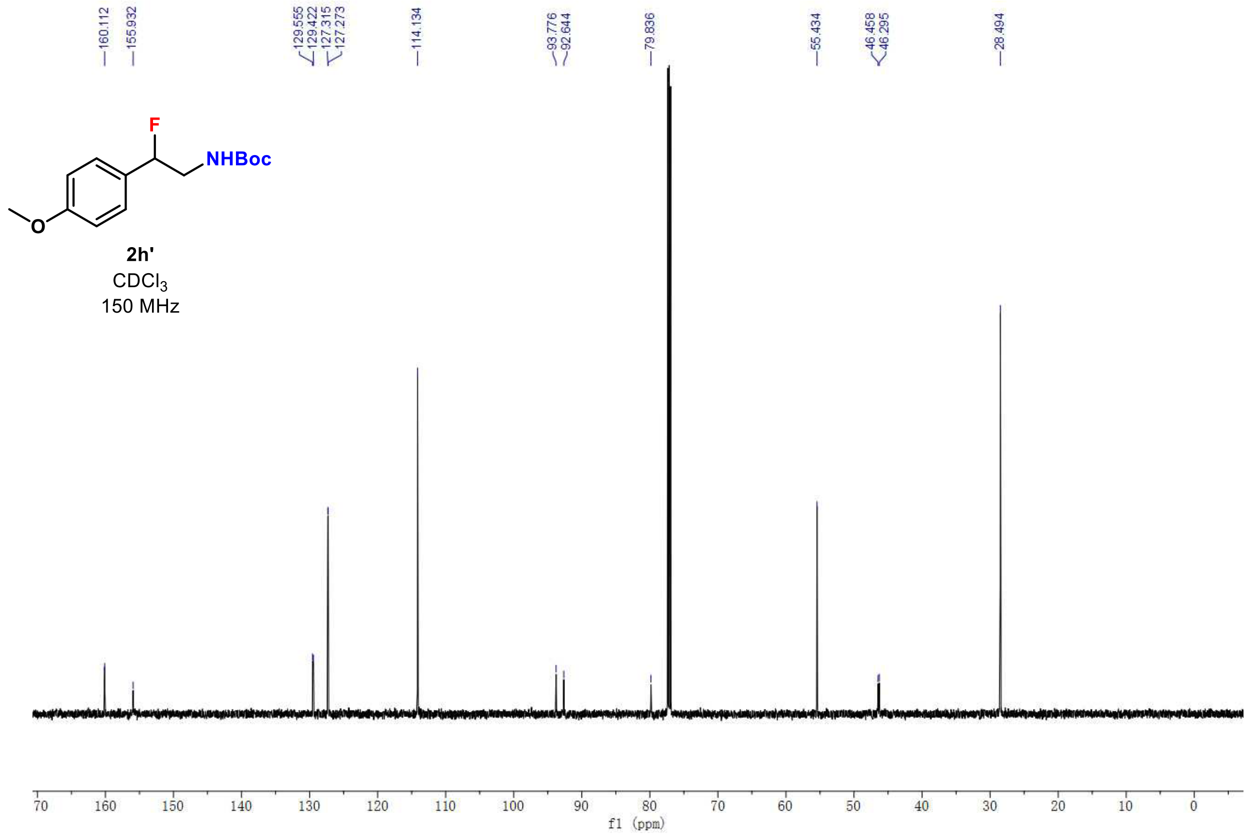

Supplementary Fig. 62. **^{13}C NMR** Spectra of **2h'**

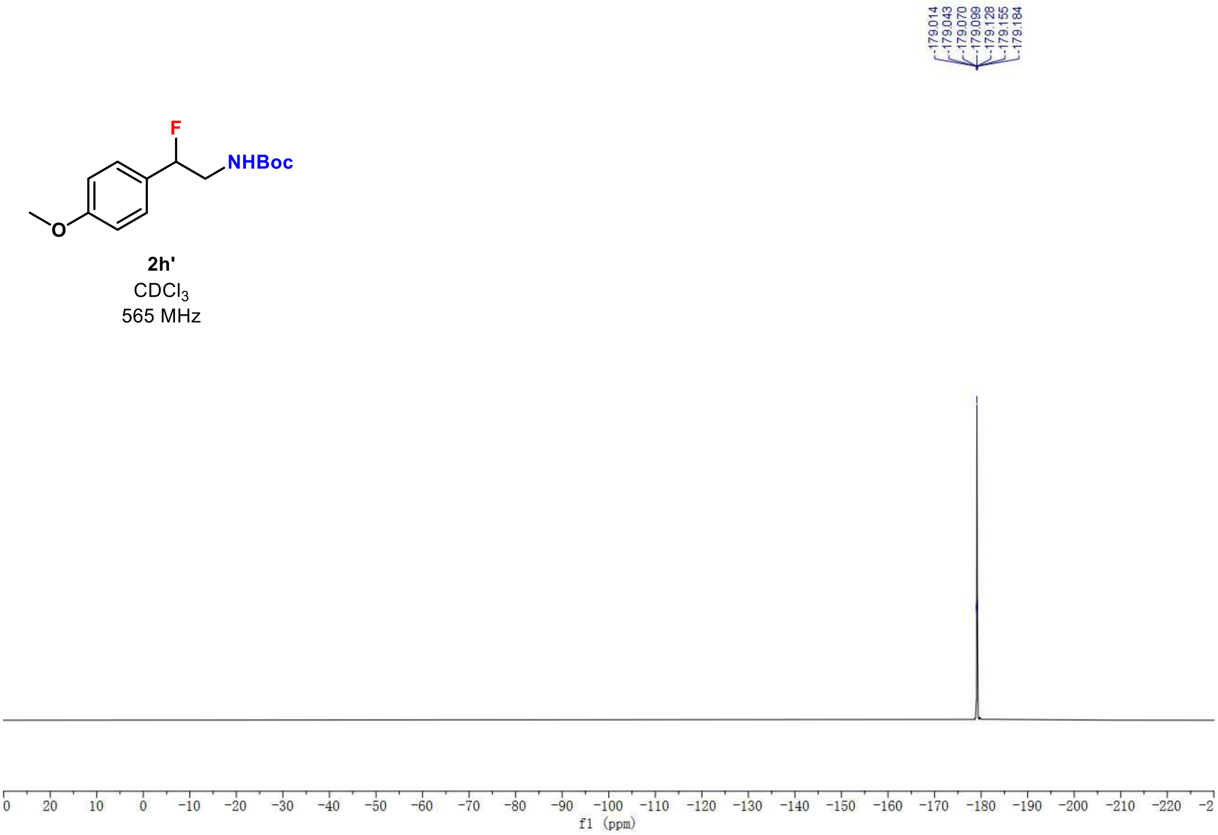

Supplementary Fig. 63. ^{19}**F NMR** Spectra of **2h'**

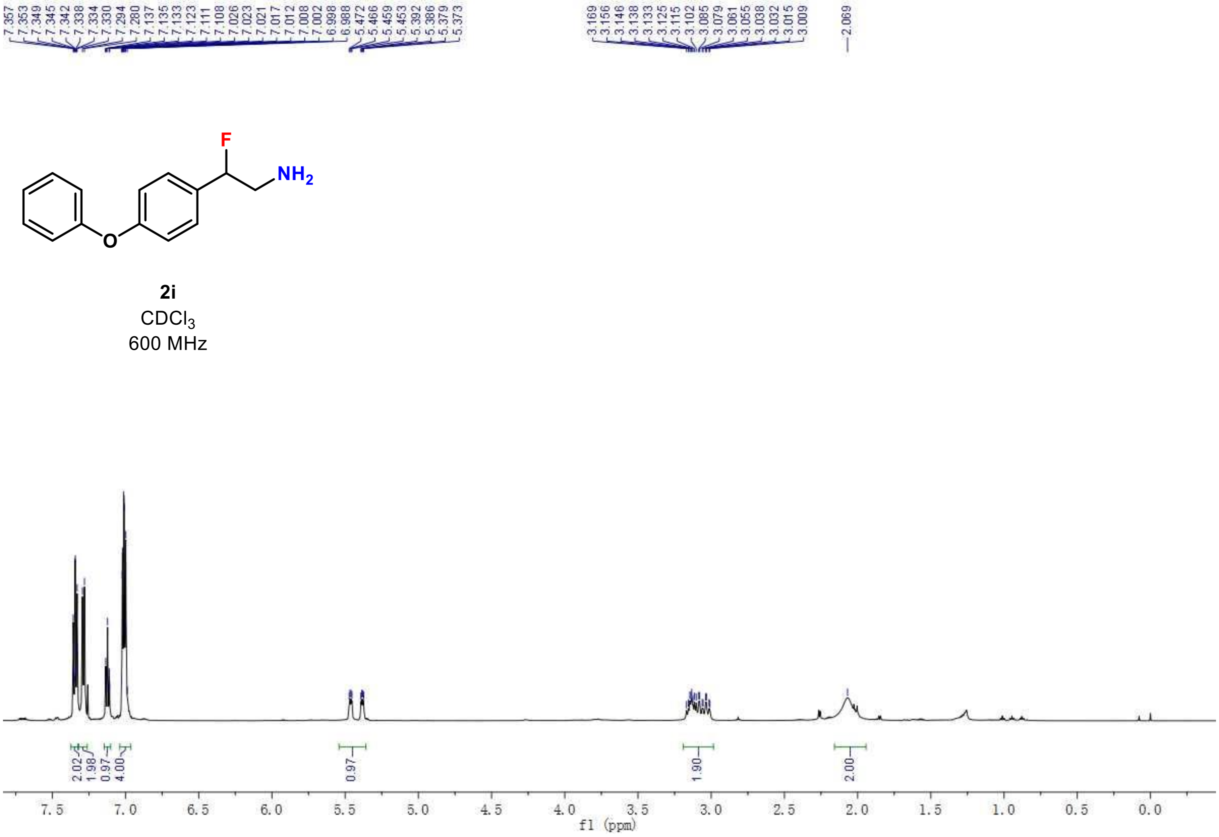

Supplementary Fig. 64. ^{1}**H NMR** Spectra of **2i**

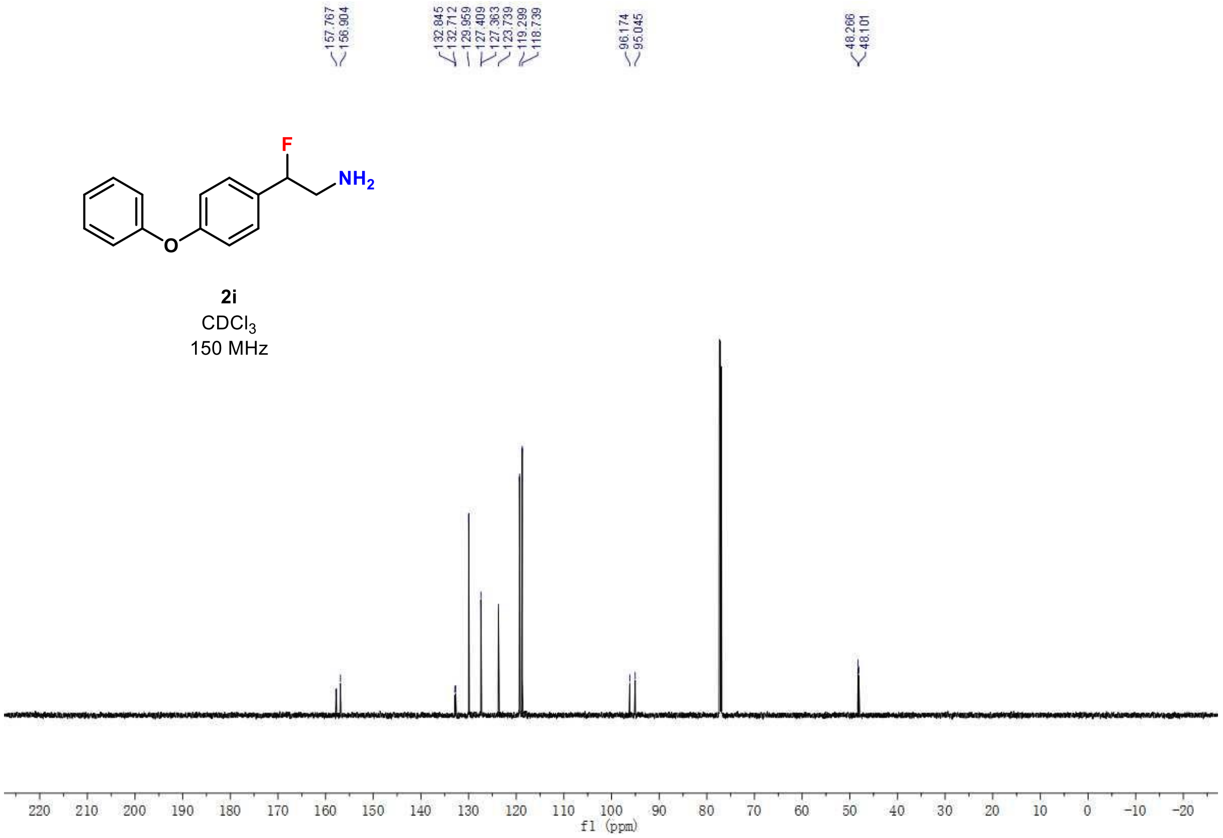

Supplementary Fig. 65. ^{13}C NMR Spectra of 2i

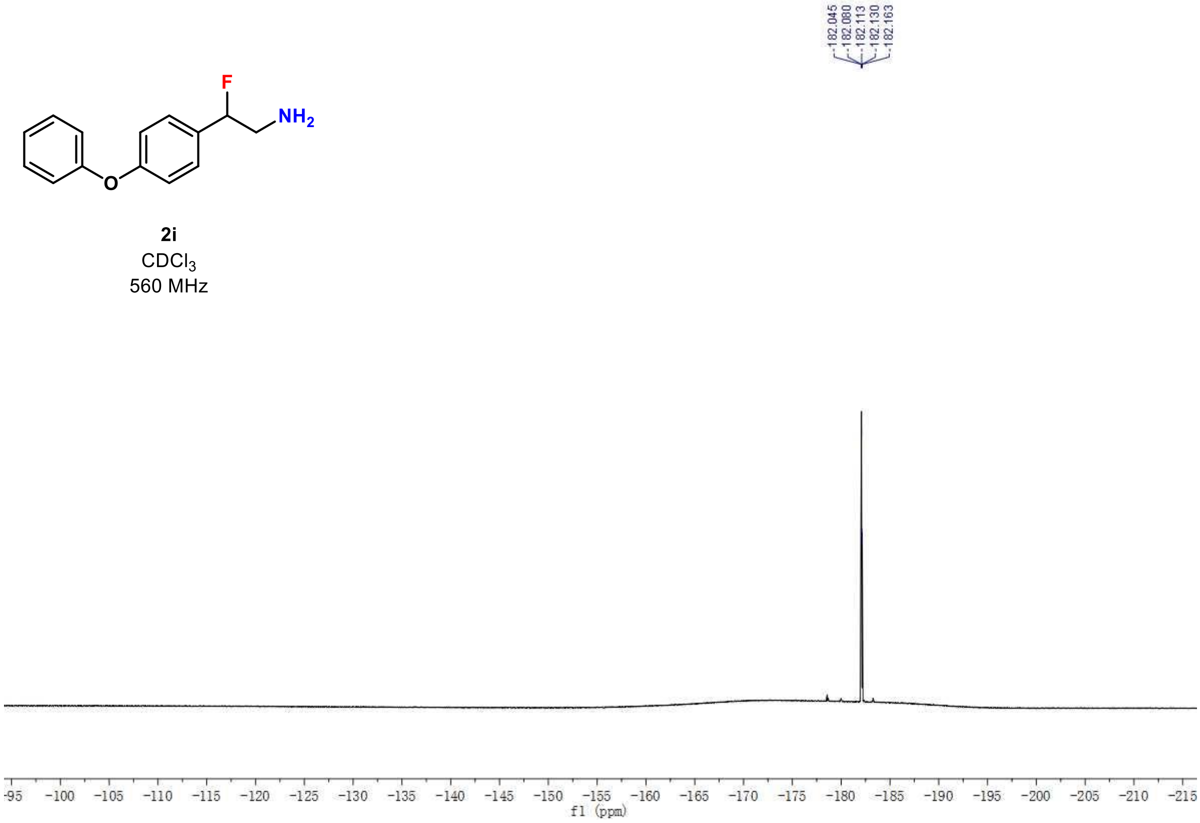

Supplementary Fig. 66. ^{19}F NMR Spectra of 2i

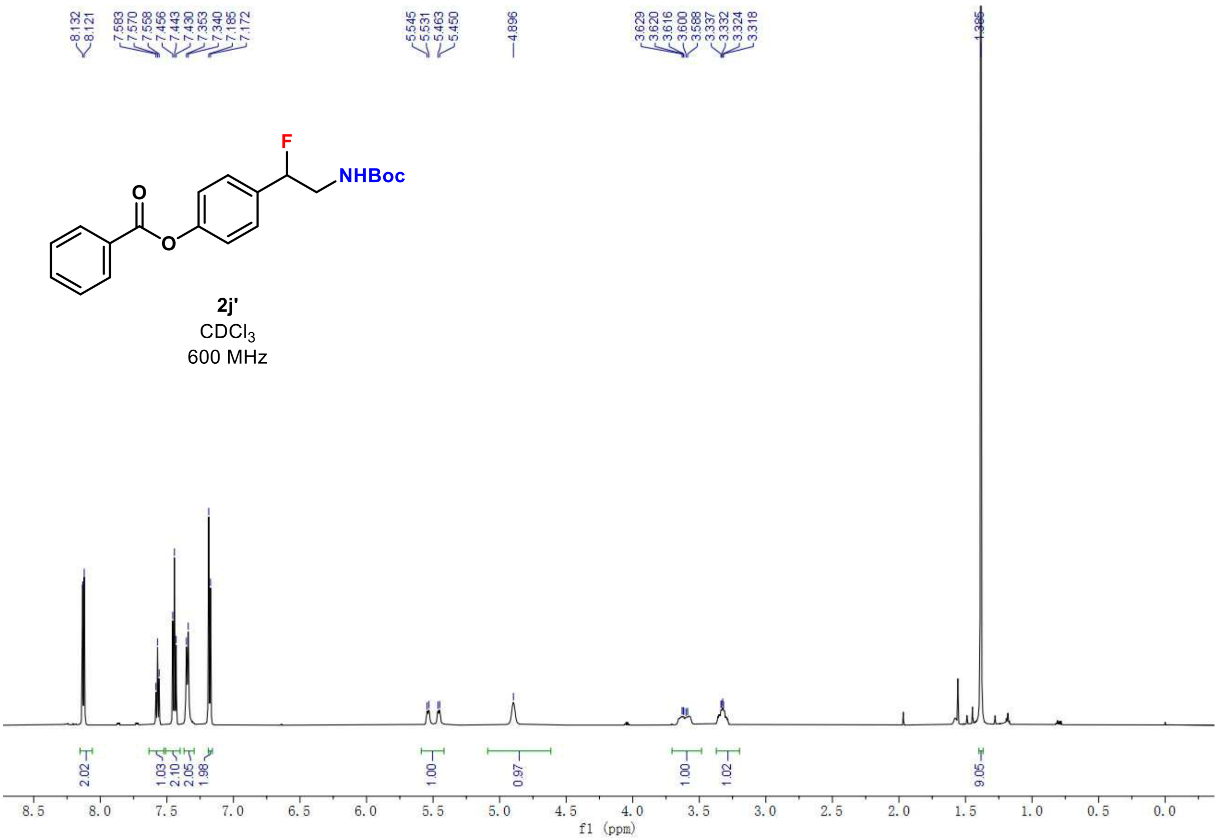

Supplementary Fig. 67. ^{1}**H NMR** Spectra of **2j'**

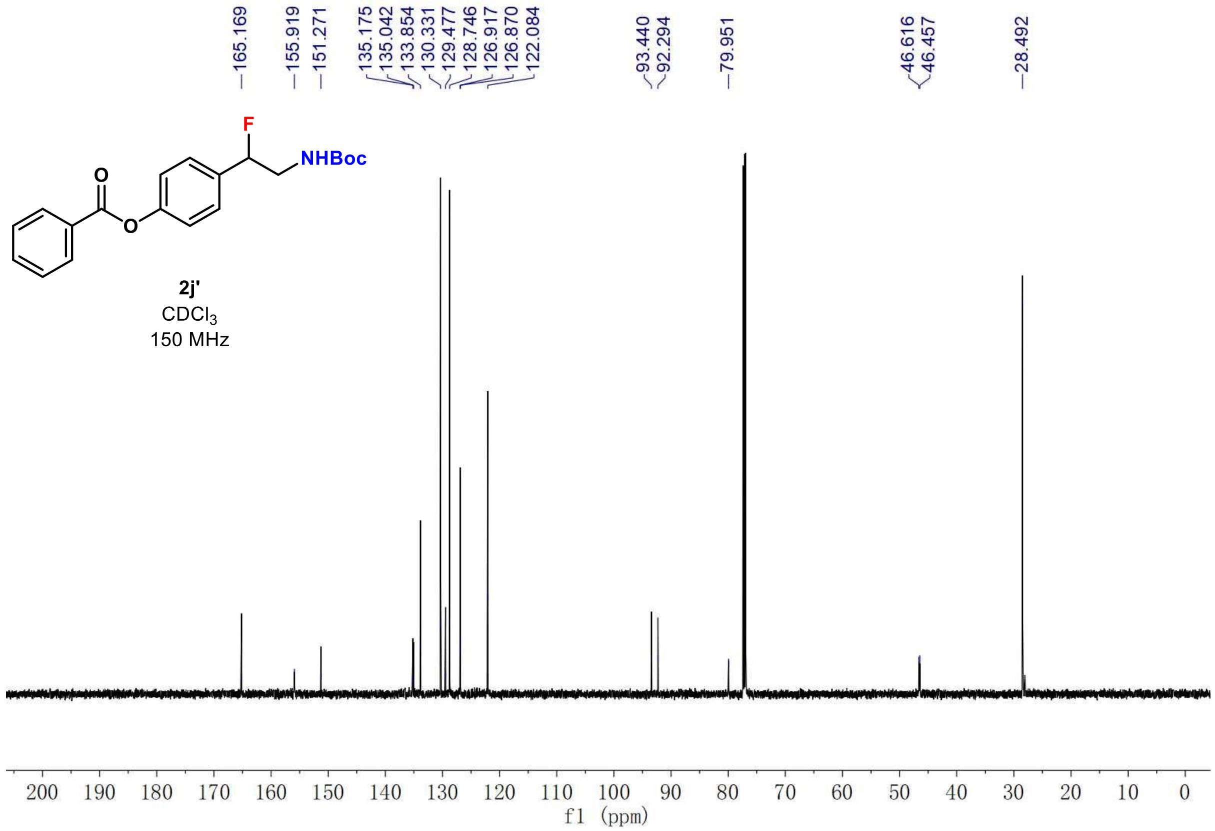

Supplementary Fig. 68. ^{13}**C NMR** Spectra of **2j'**

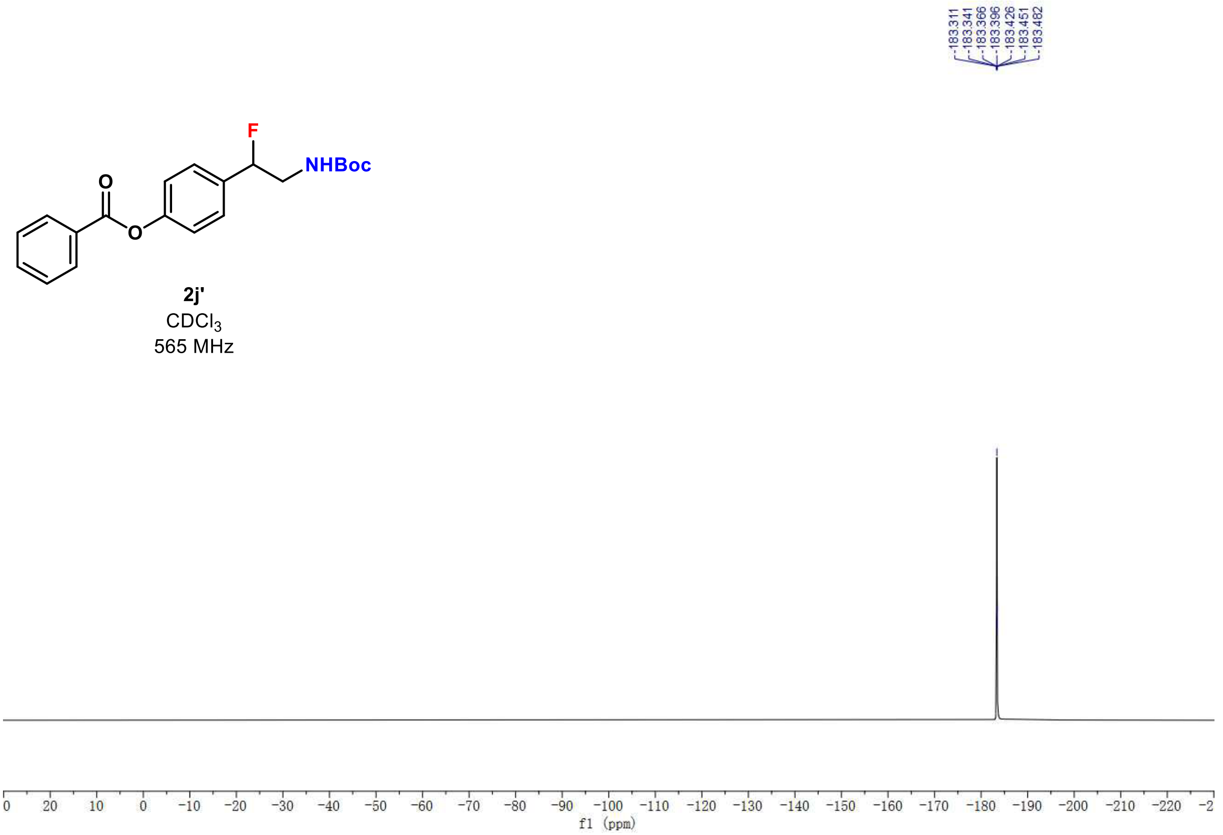

Supplementary Fig. 69. ^{19}**F NMR** Spectra of **2j'**

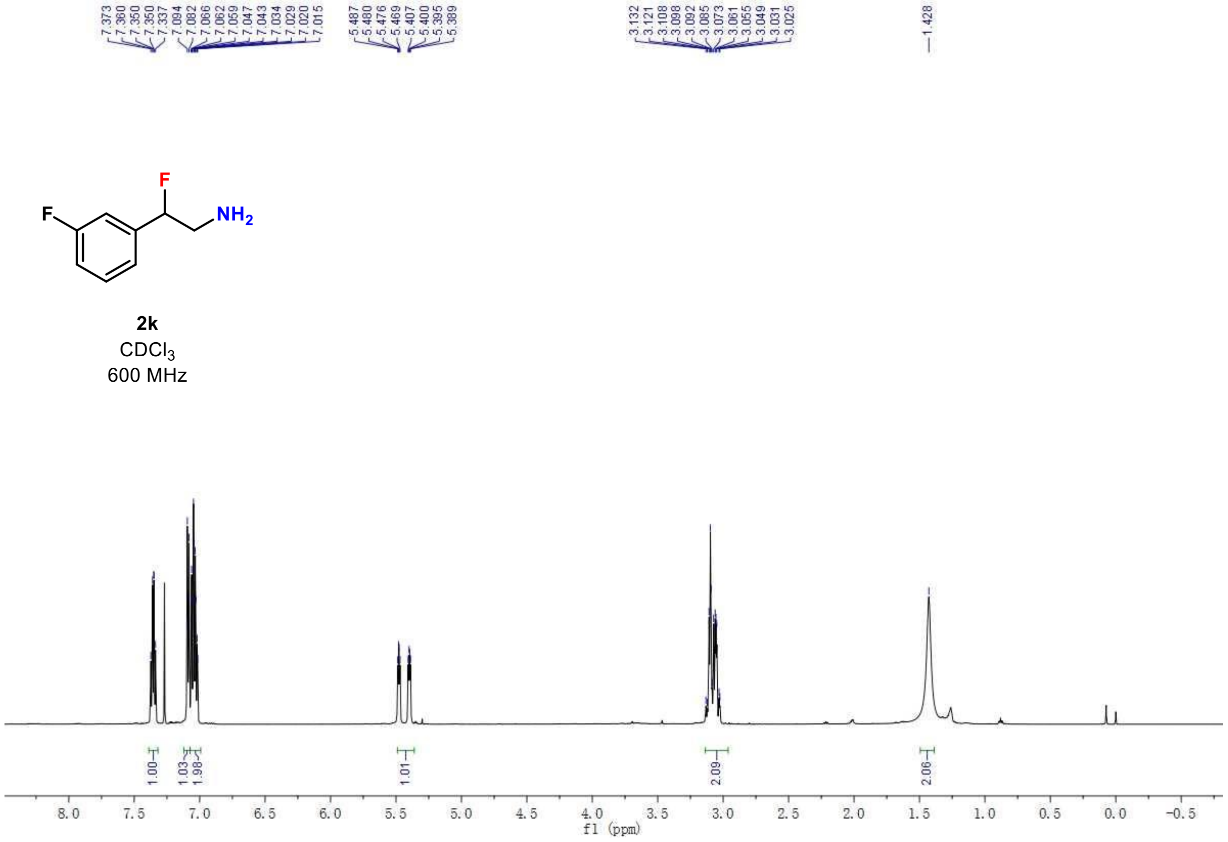

Supplementary Fig. 70. ^{1}**H NMR** Spectra of **2k**

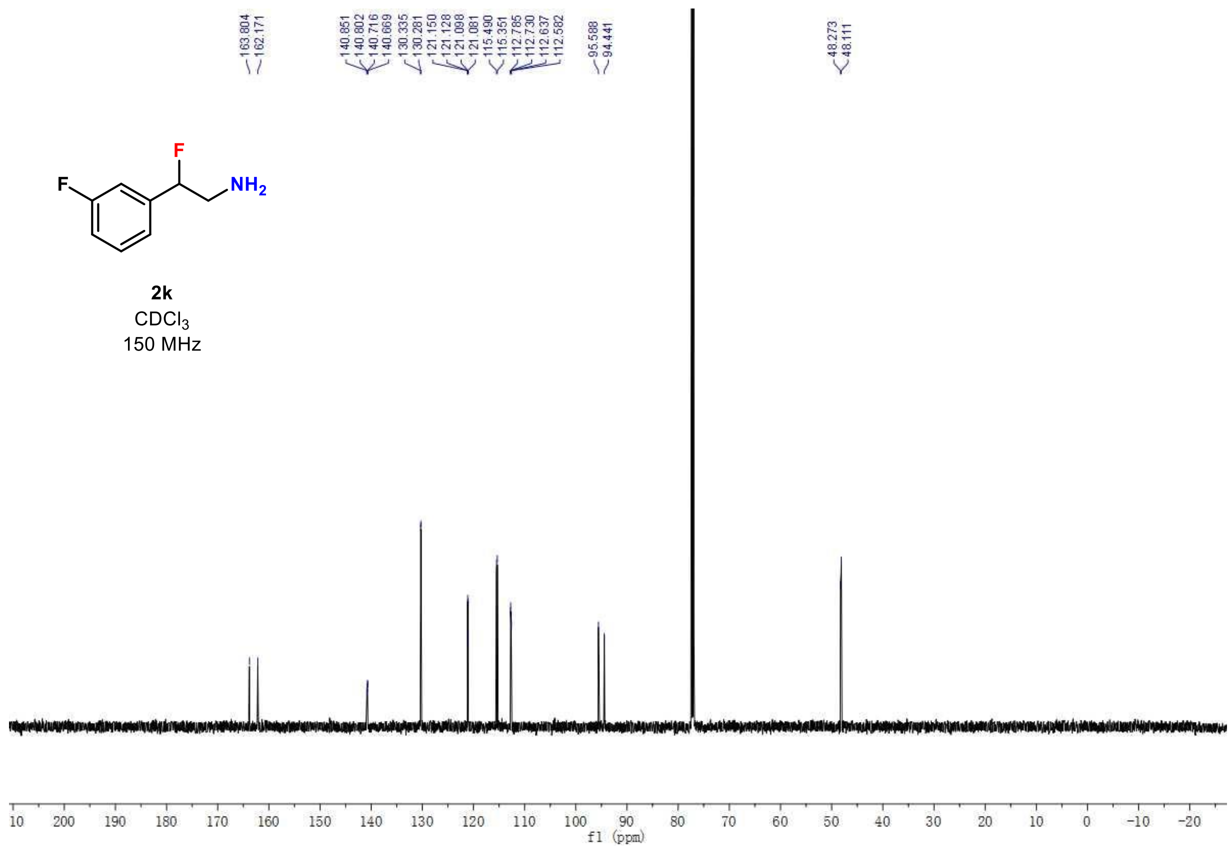

Supplementary Fig. 71. ^{13}**C NMR** Spectra of **2k**

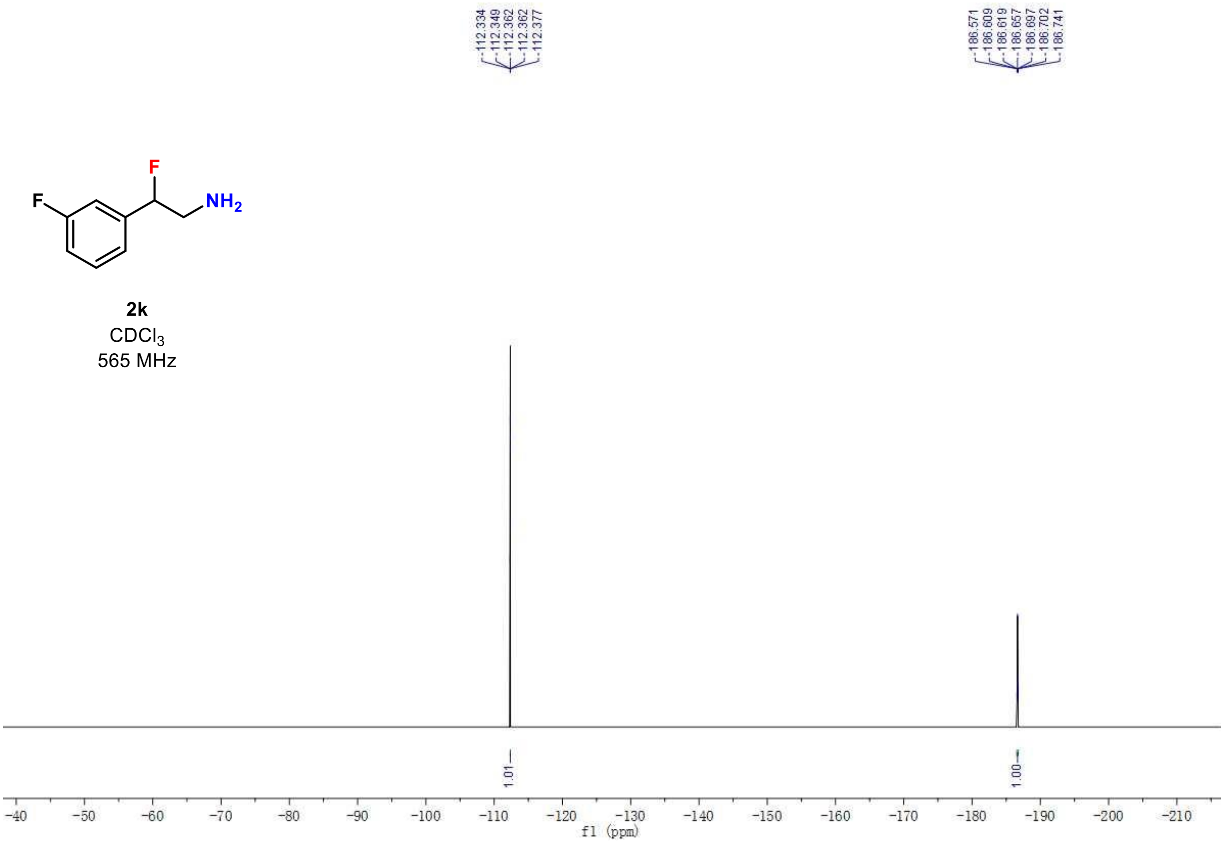

Supplementary Fig. 72. ^{19}**F NMR** Spectra of **2k**

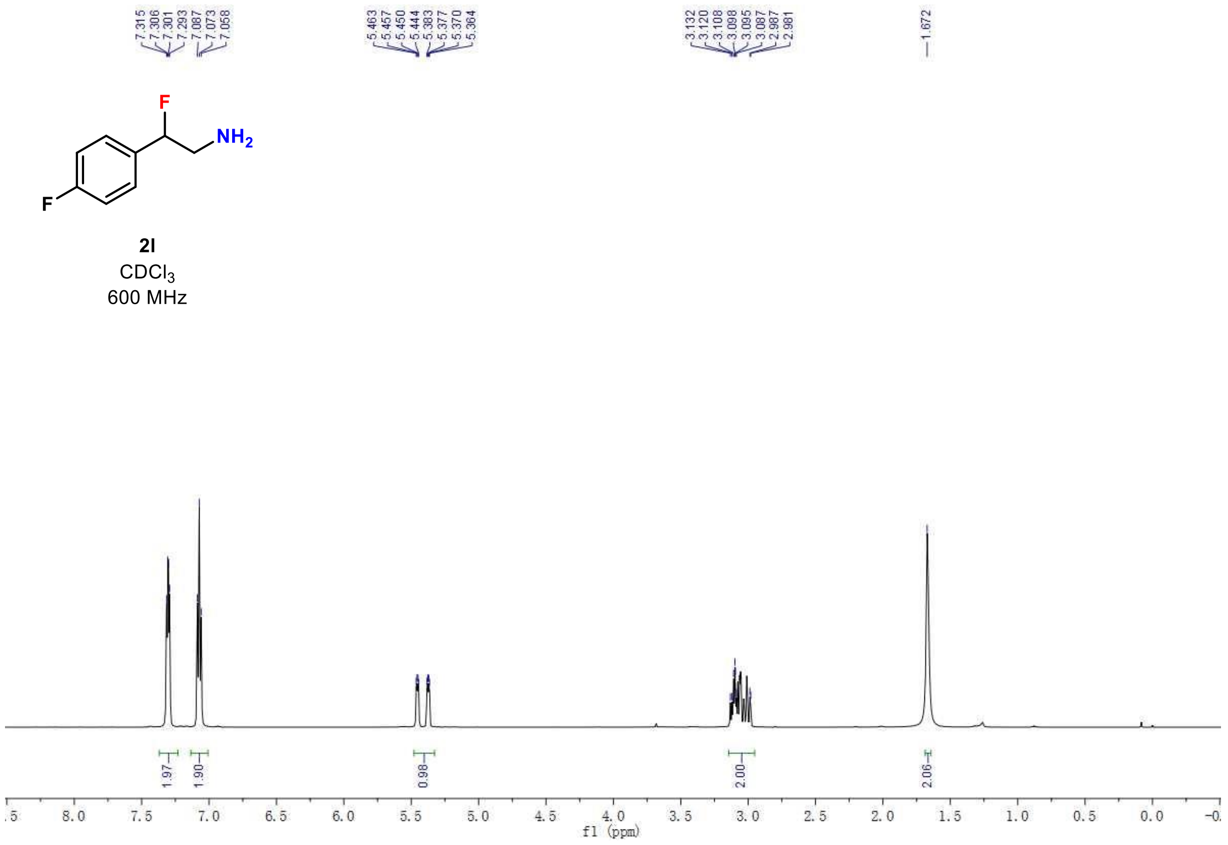

Supplementary Fig. 73. ^{1}**H NMR** Spectra of **2l**

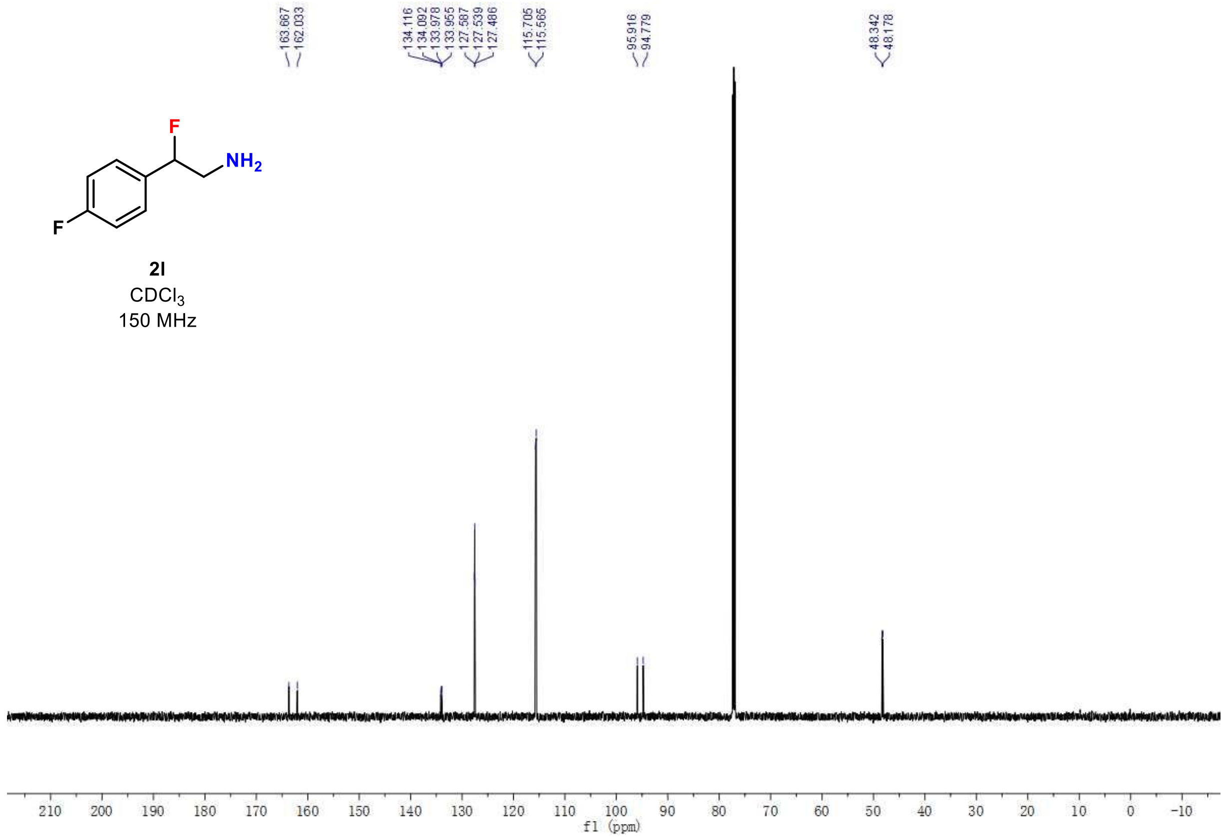

Supplementary Fig. 74. ^{13}**C NMR** Spectra of **2l**

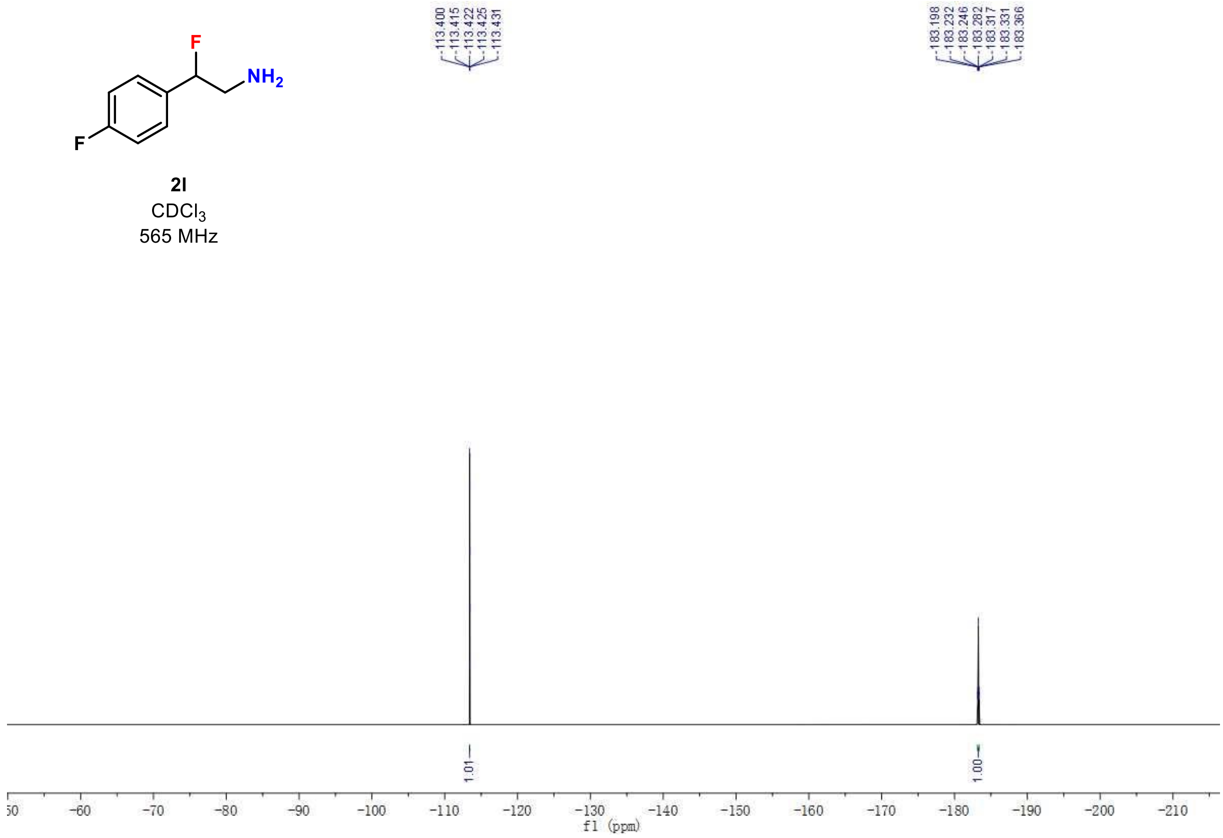

Supplementary Fig. 75. **^{19}F NMR** Spectra of **2l**

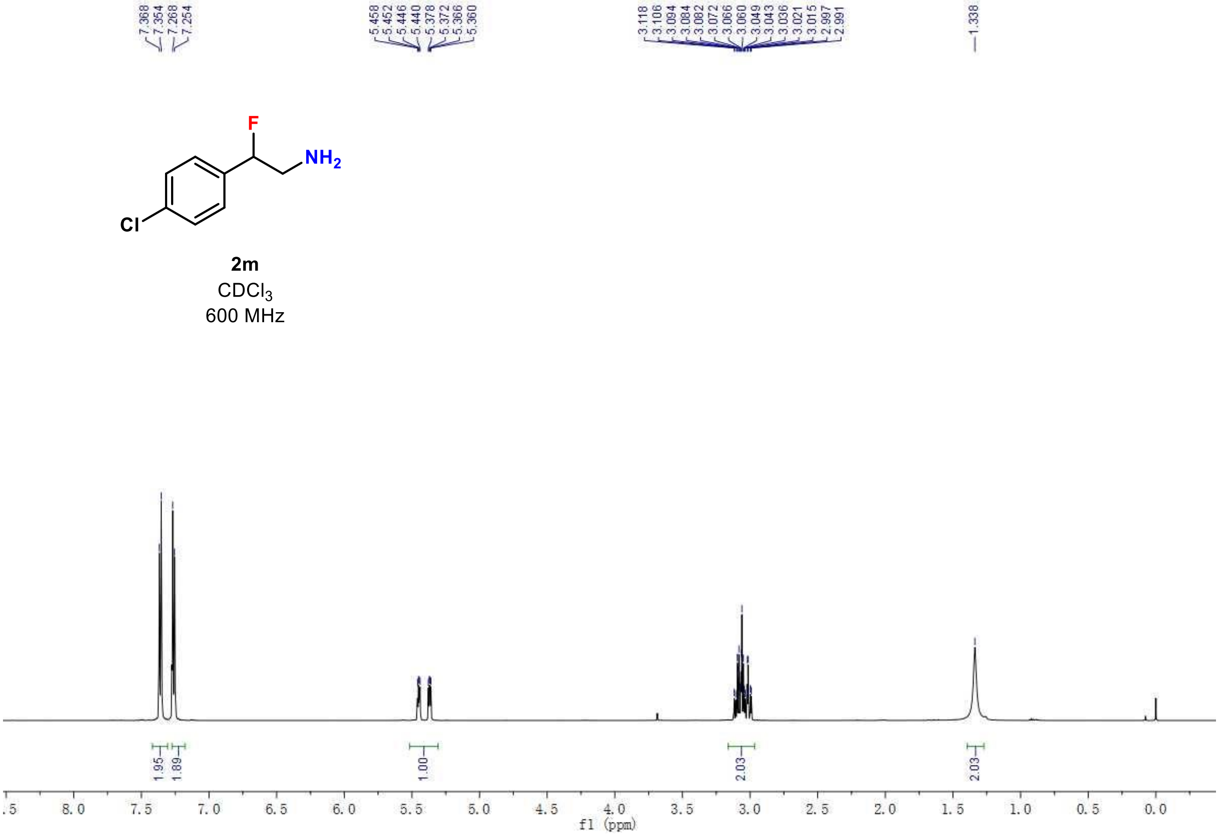

Supplementary Fig. 76. **^{1}H NMR** Spectra of **2m**

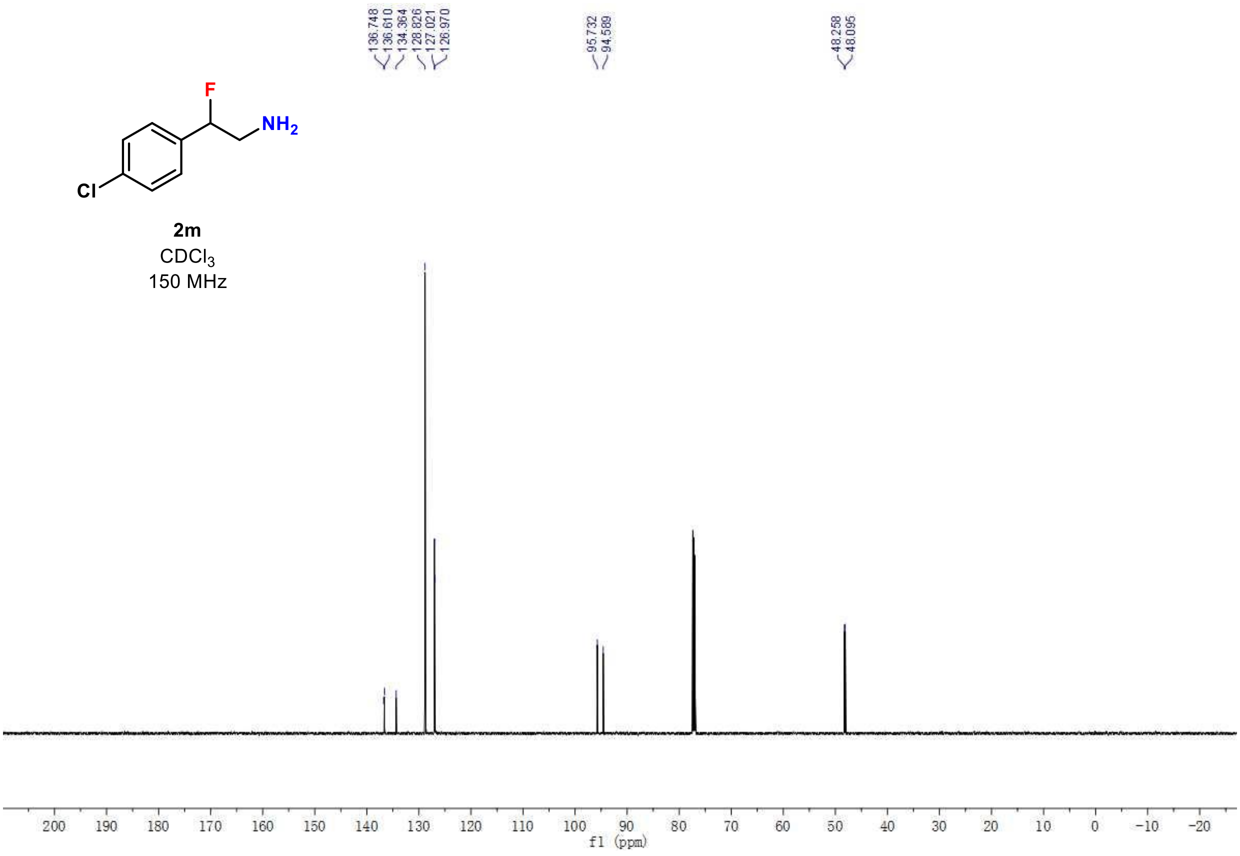

Supplementary Fig. 77. ^{13}**C NMR** Spectra of **2m**

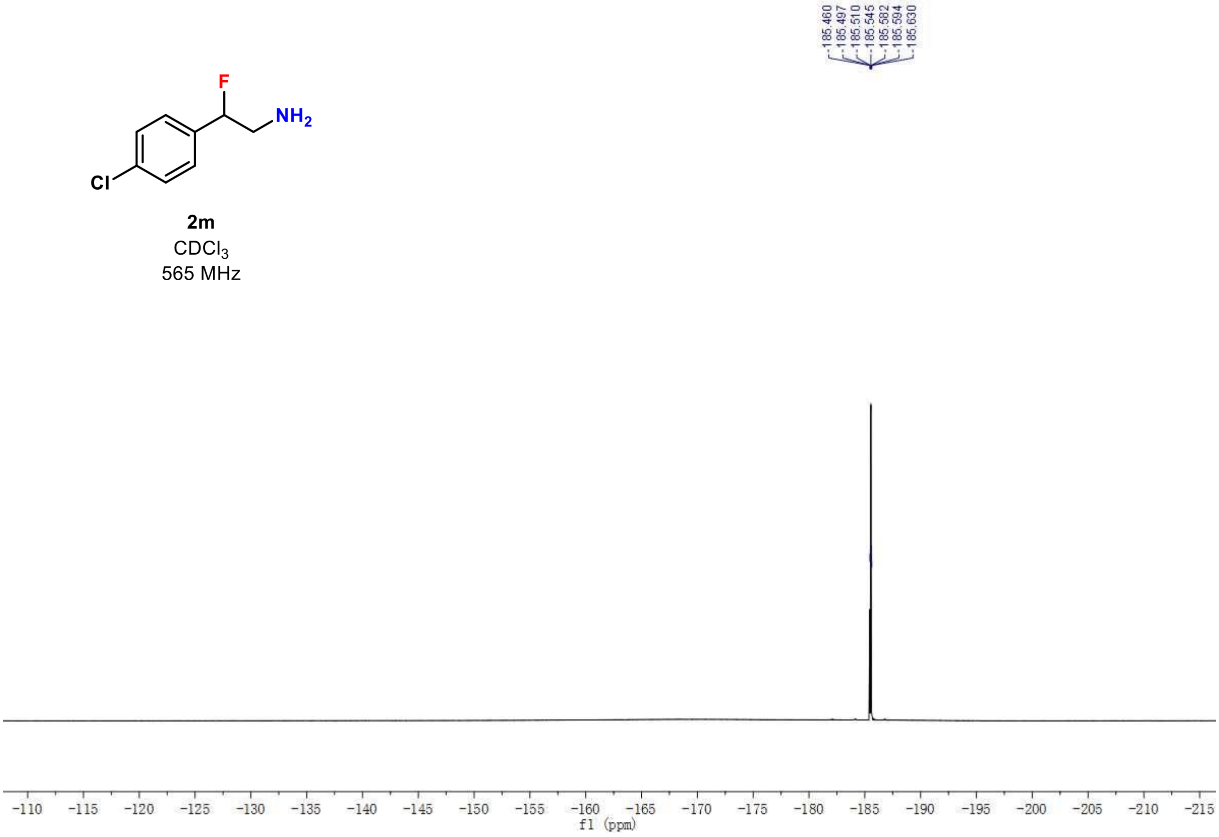

Supplementary Fig. 78. ^{19}**F NMR** Spectra of **2m**

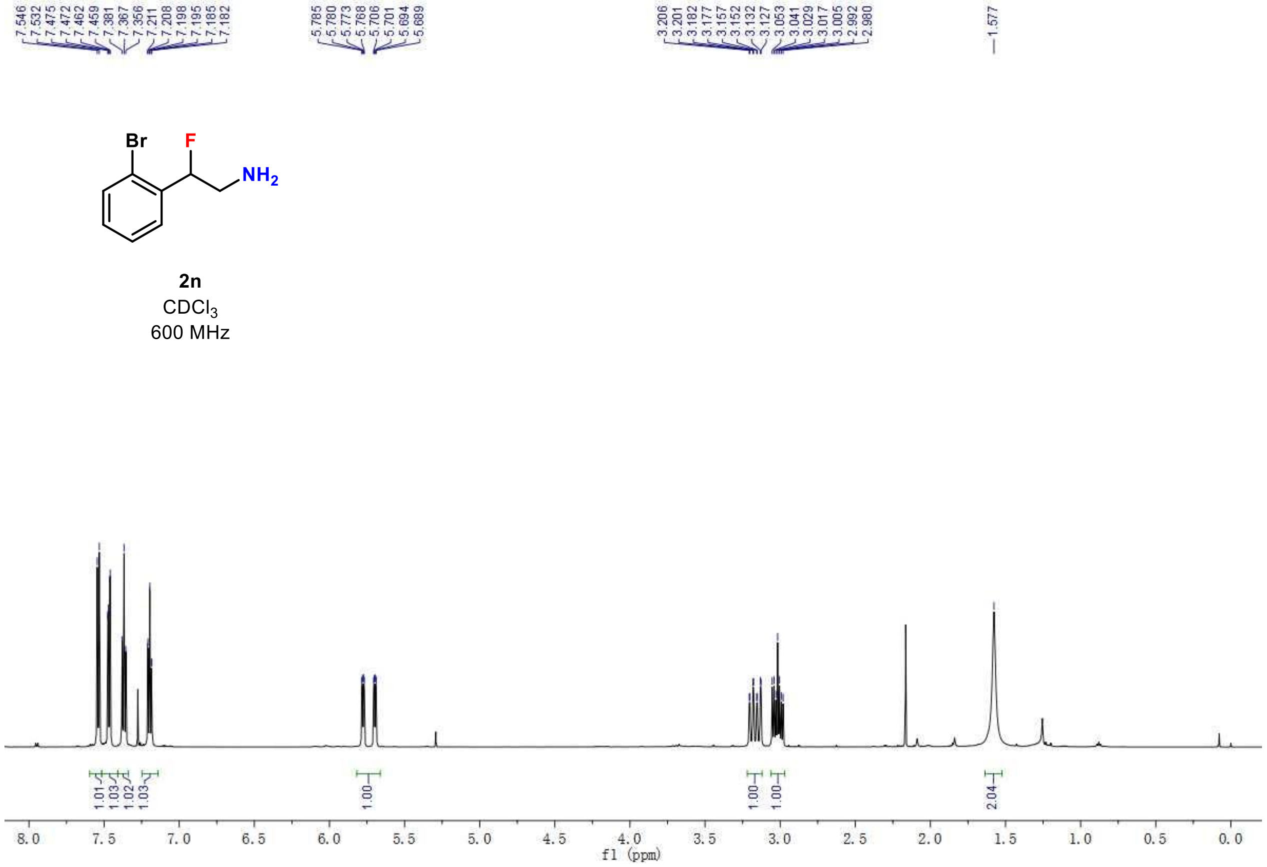

Supplementary Fig. 79. ^{1}**H NMR** Spectra of **2n**

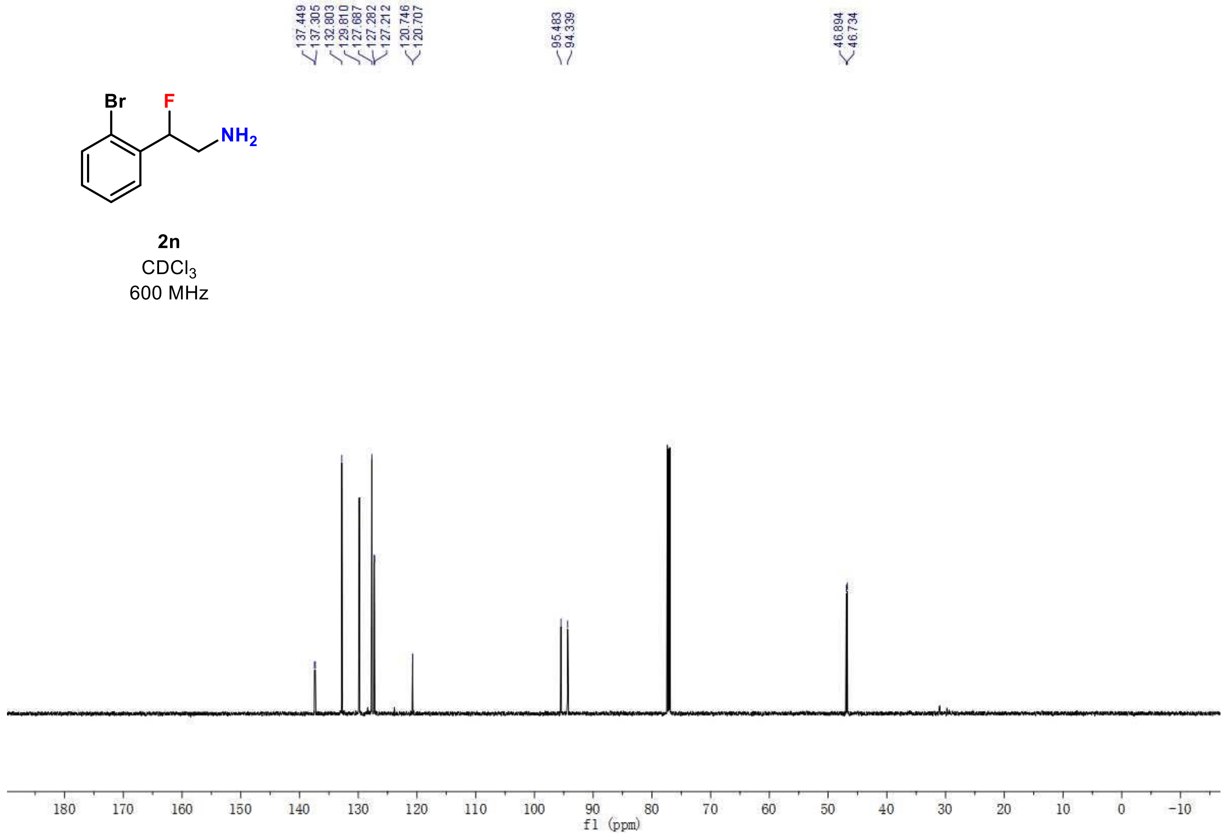

Supplementary Fig. 80. ^{13}**C NMR** Spectra of **2n**

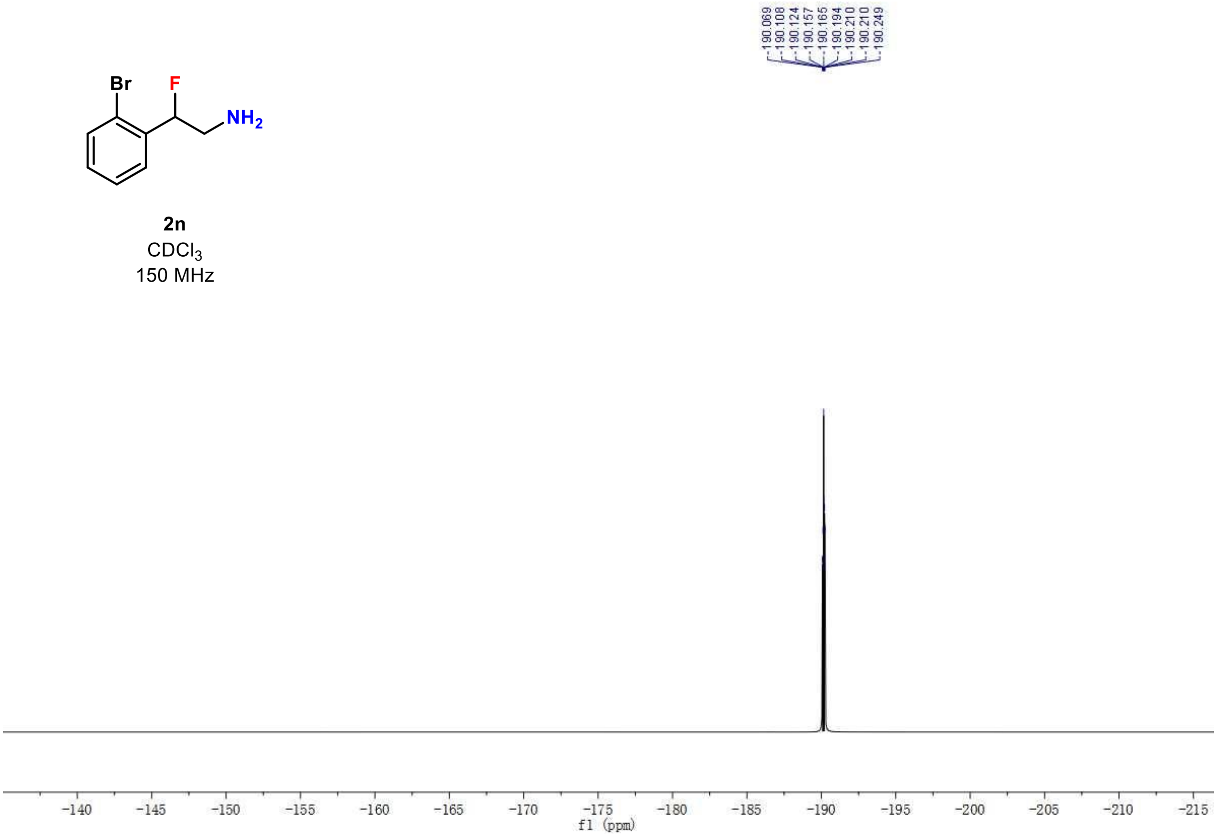

Supplementary Fig. 81. ^{19}**F NMR** Spectra of **2n**

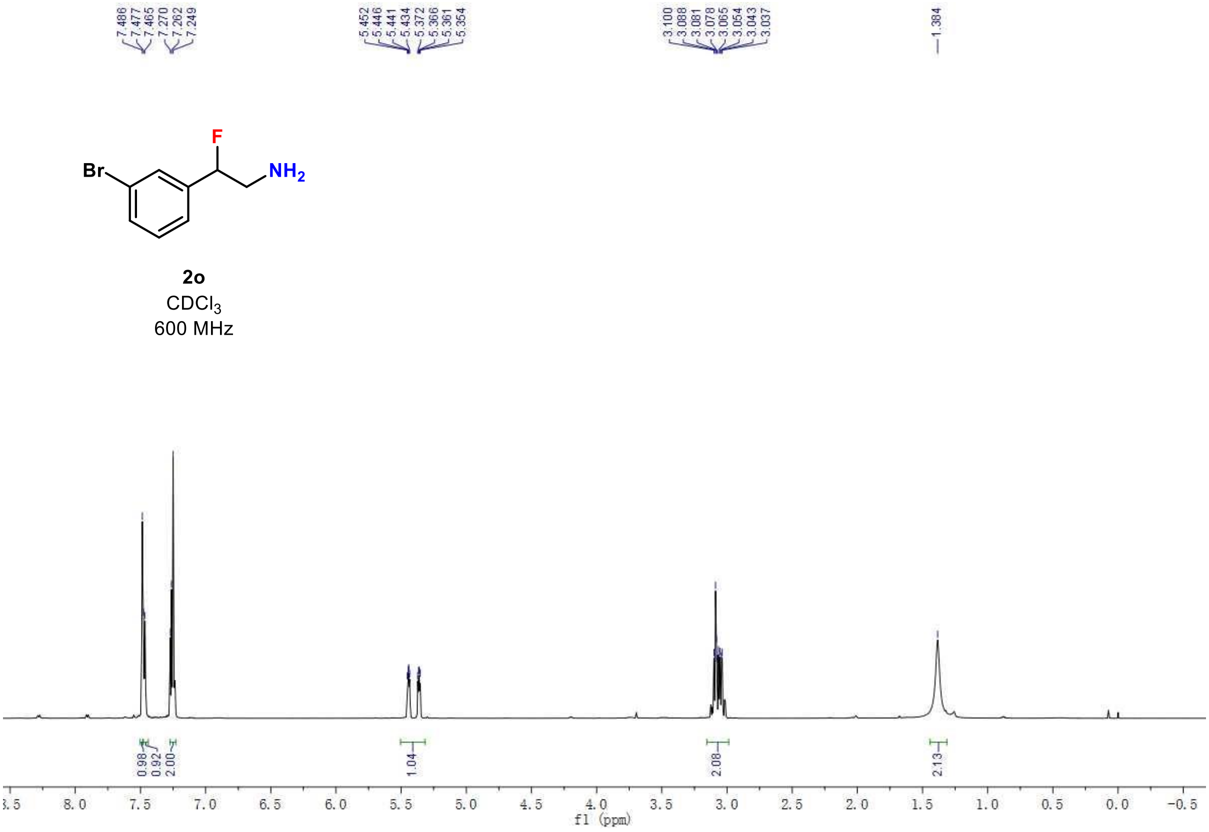

Supplementary Fig. 82. ^{1}**H NMR** Spectra of **2o**

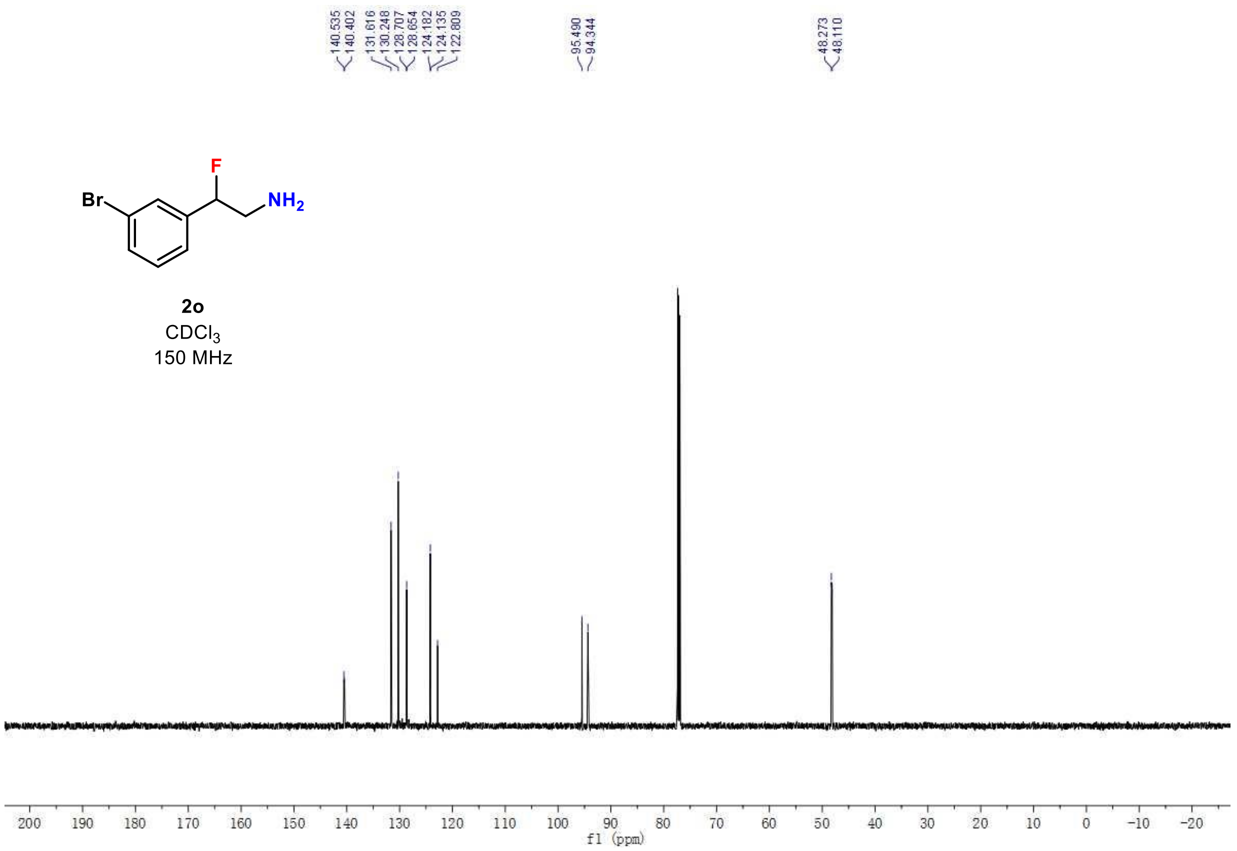

Supplementary Fig. 83. ^{13}**C NMR** Spectra of **2o**

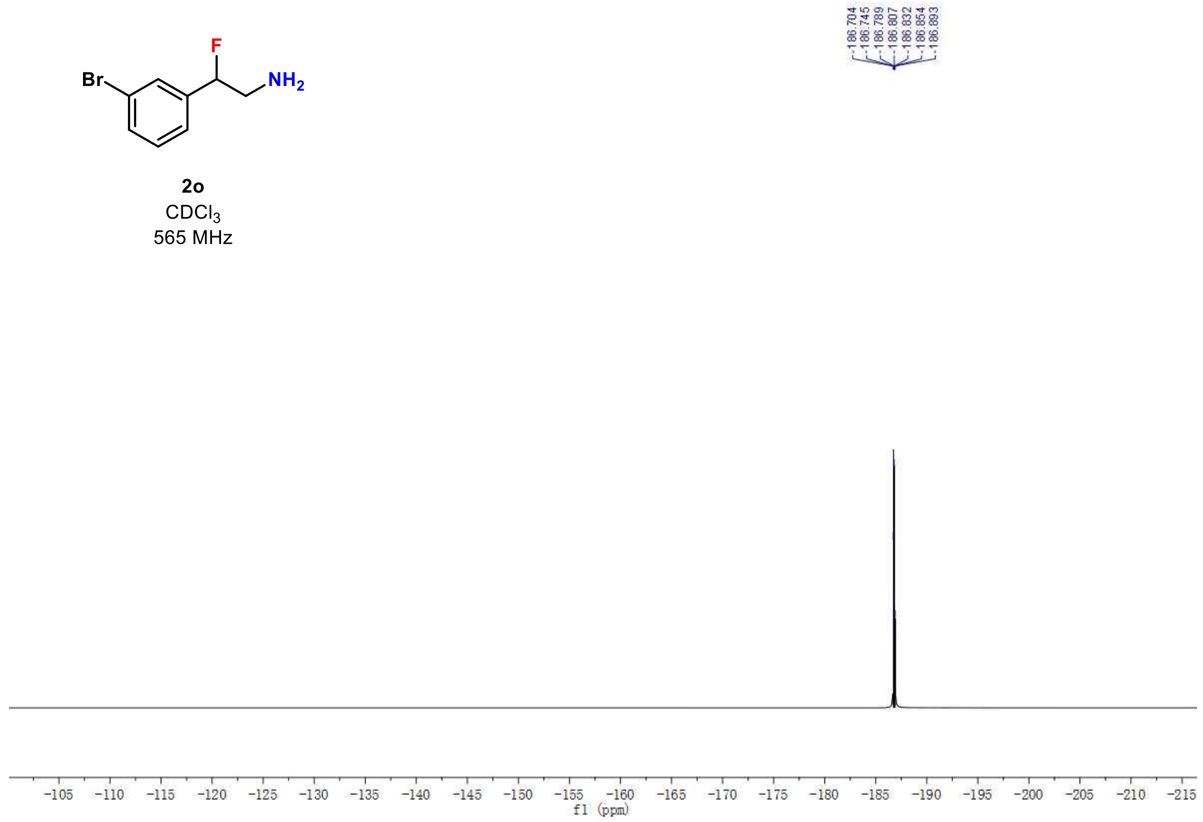

Supplementary Fig. 84. ^{19}**F NMR** Spectra of **2o**

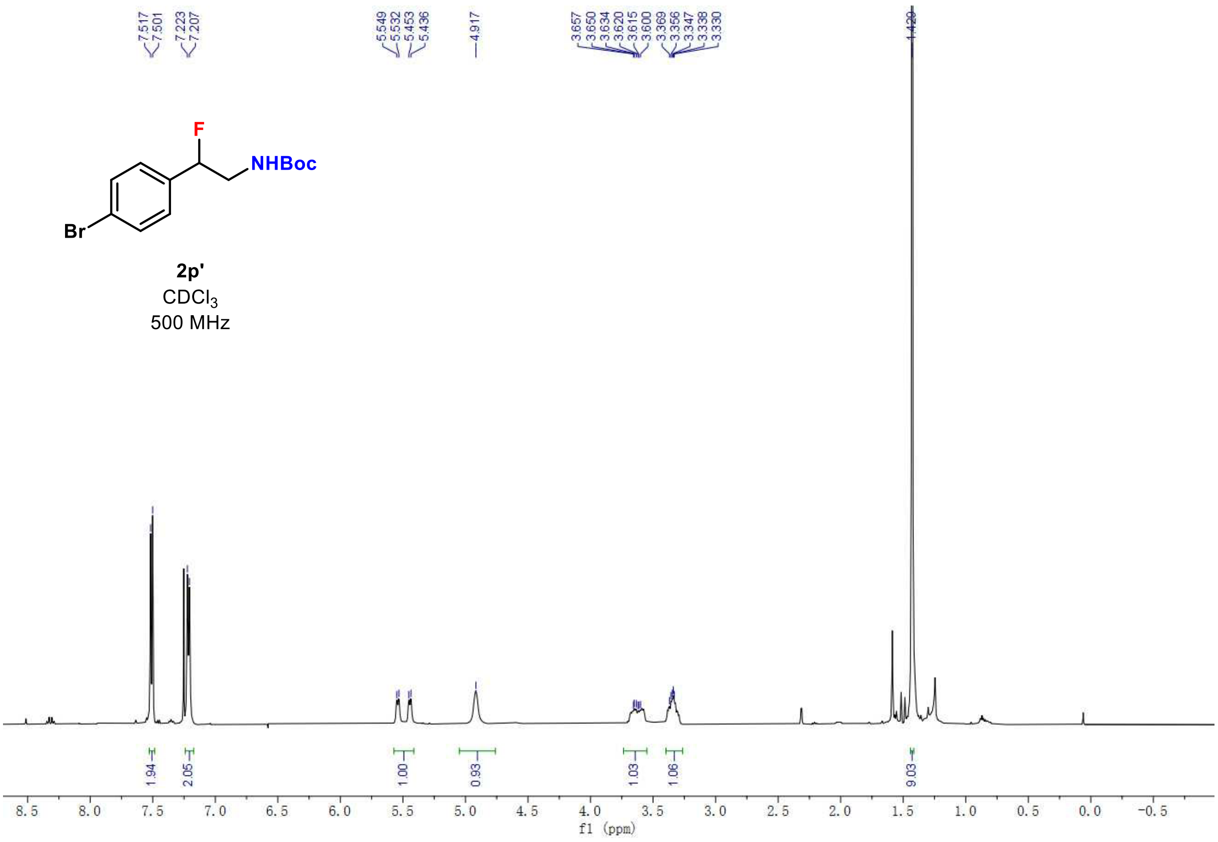

Supplementary Fig. 85. ¹H NMR Spectra of **2p'**

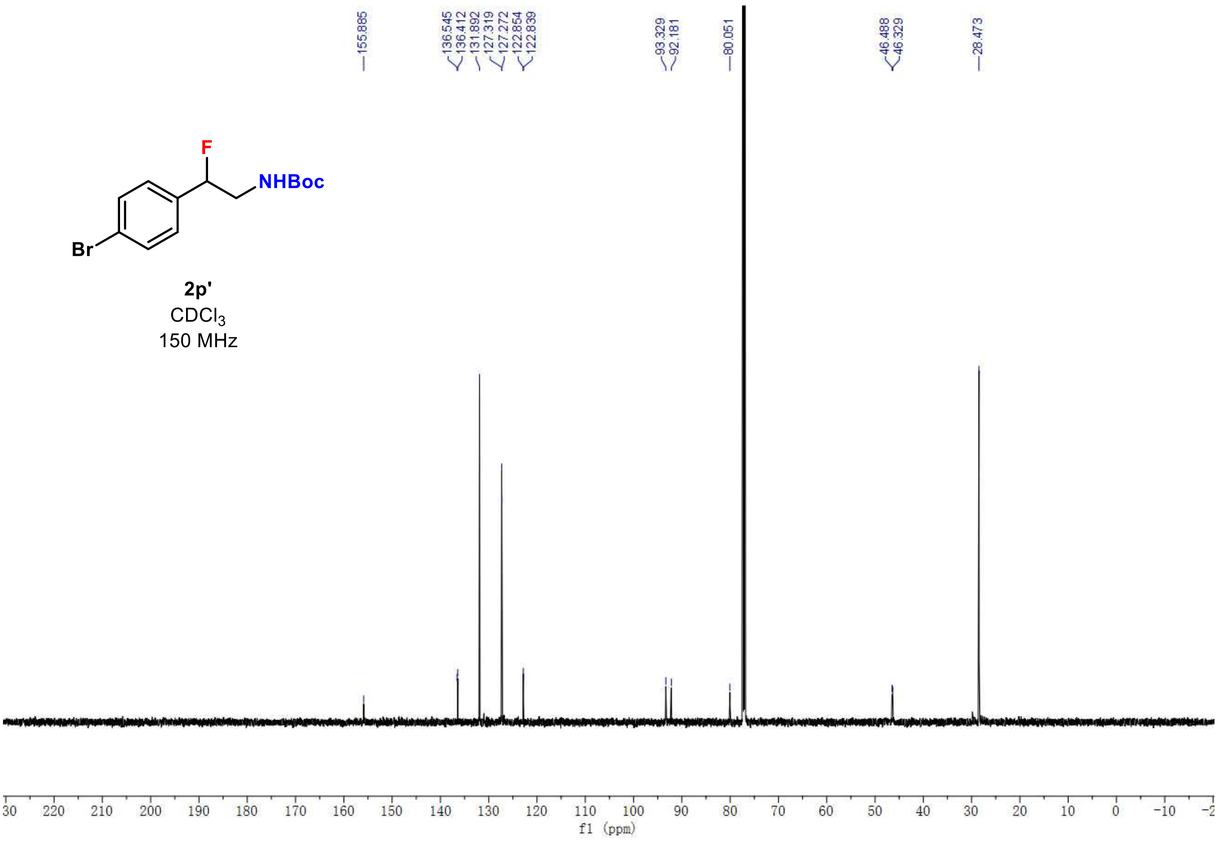

Supplementary Fig. 86. ¹³C NMR Spectra of **2p'**

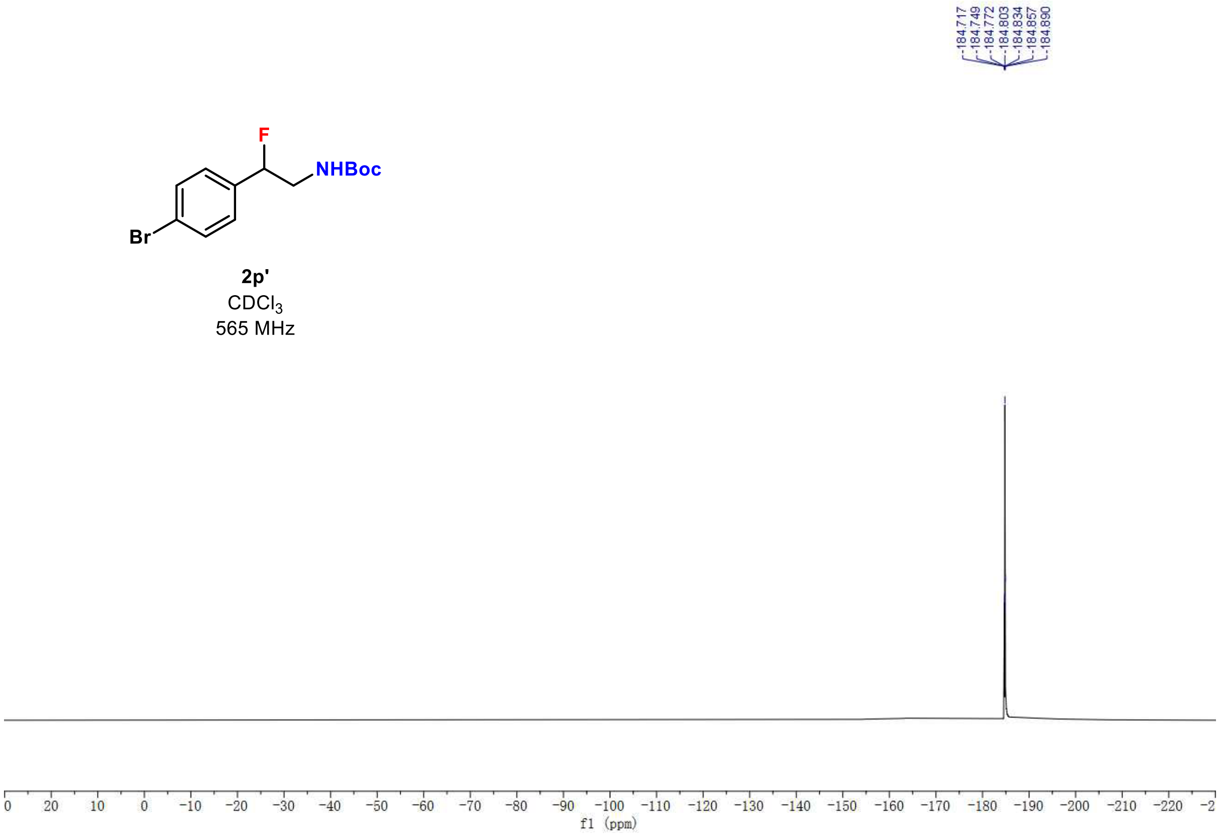

Supplementary Fig. 87. ^{19}F NMR Spectra of **2p'**

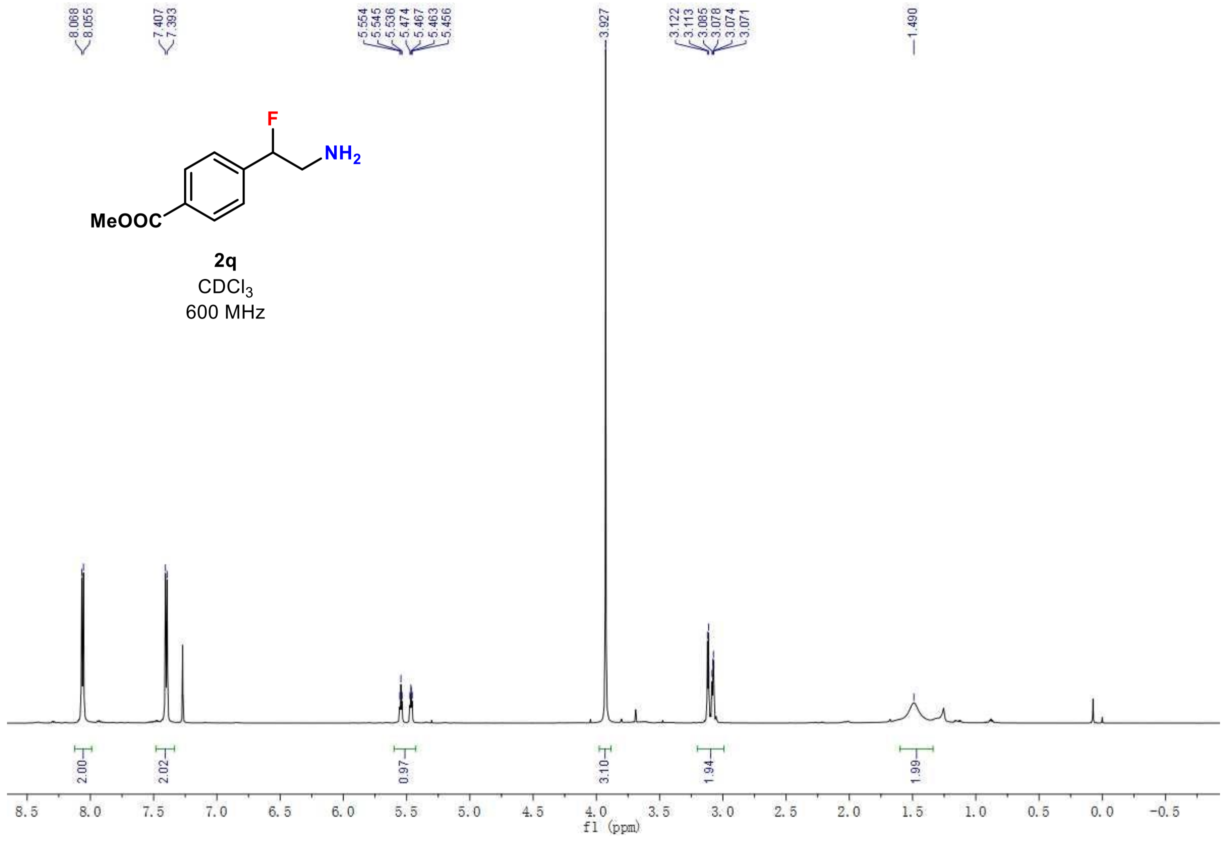

Supplementary Fig. 88. ^{1}H NMR Spectra of **2q**

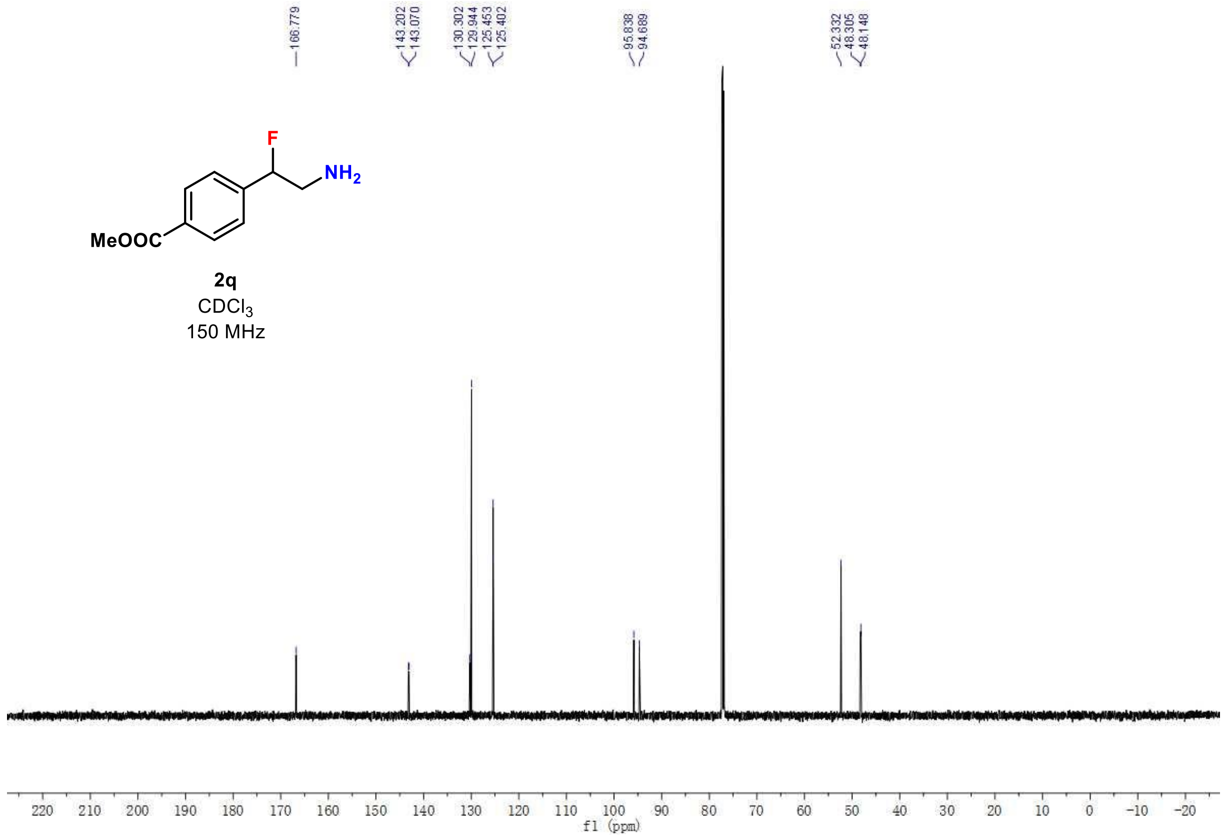

Supplementary Fig. 89. ^{13}**C NMR** Spectra of **2q**

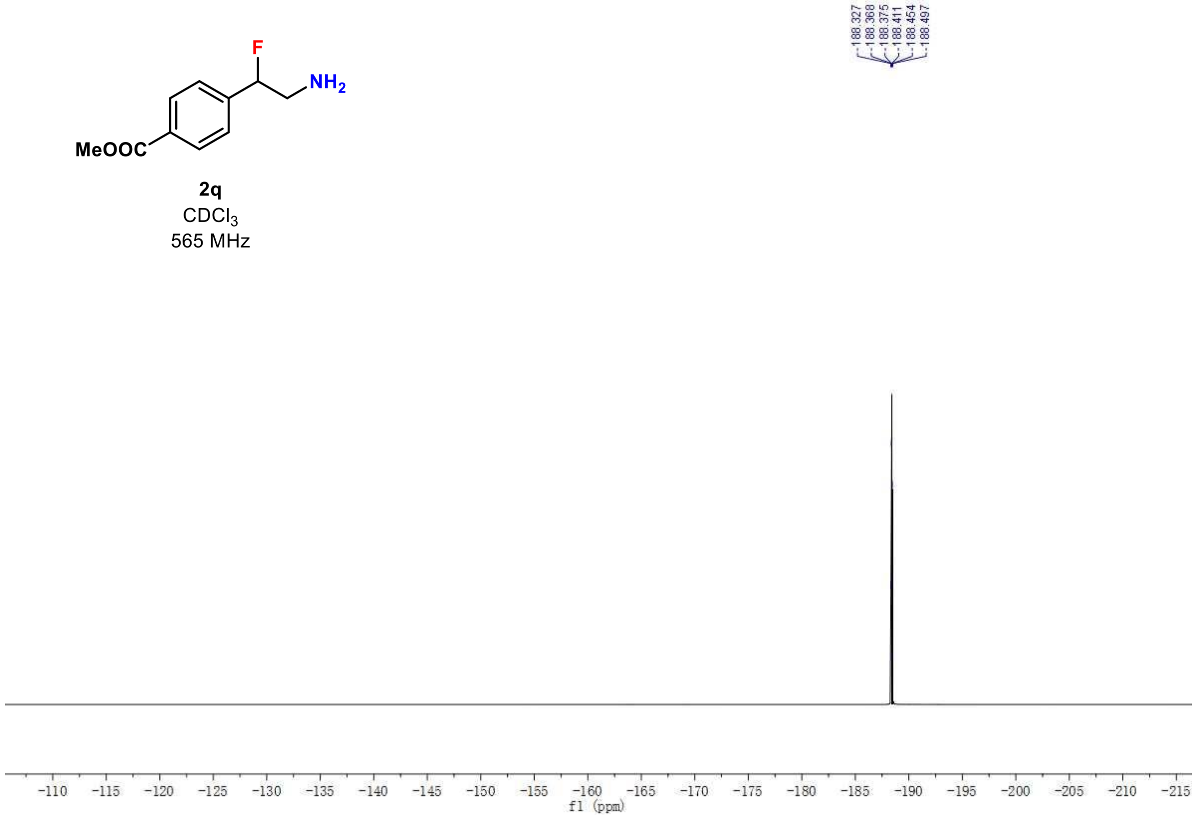

Supplementary Fig. 90. ^{19}**F NMR** Spectra of **2q**

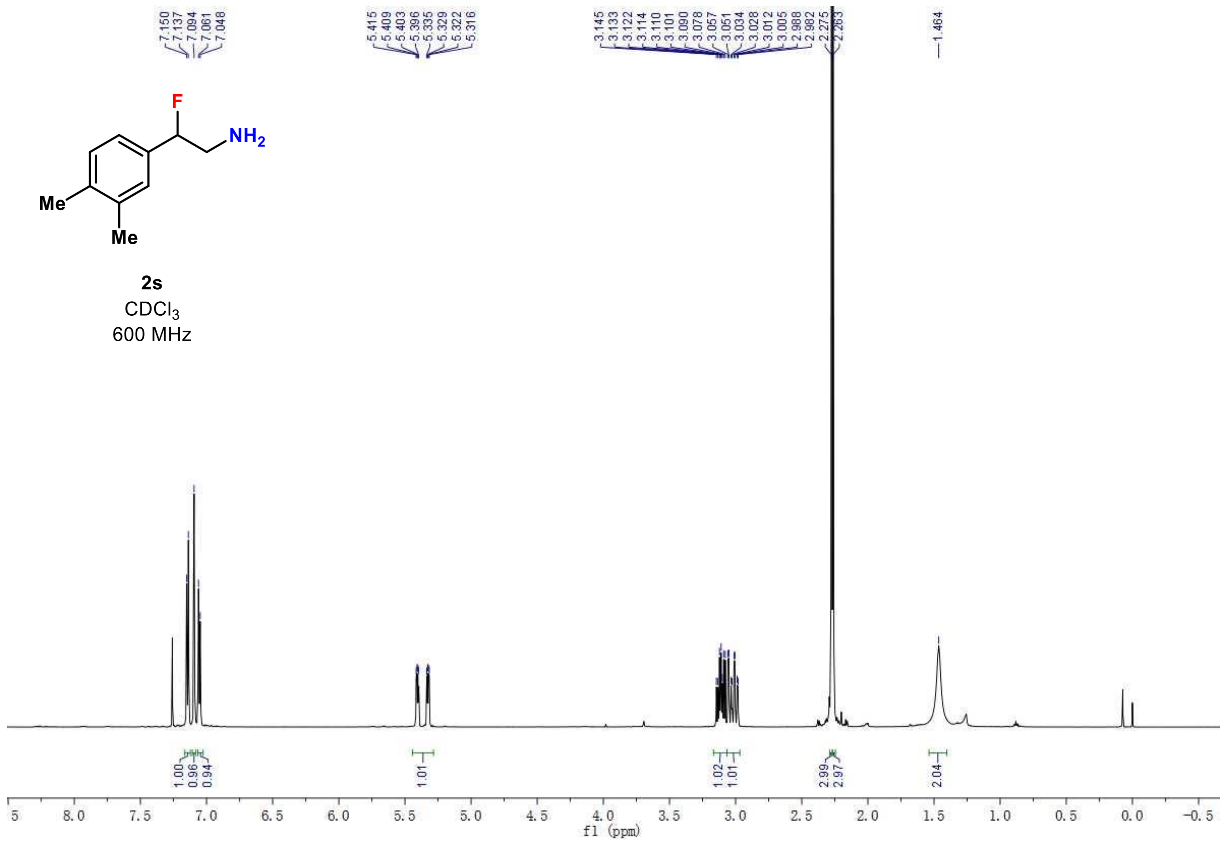

Supplementary Fig. 91. **^{1}H NMR** Spectra of **2s**

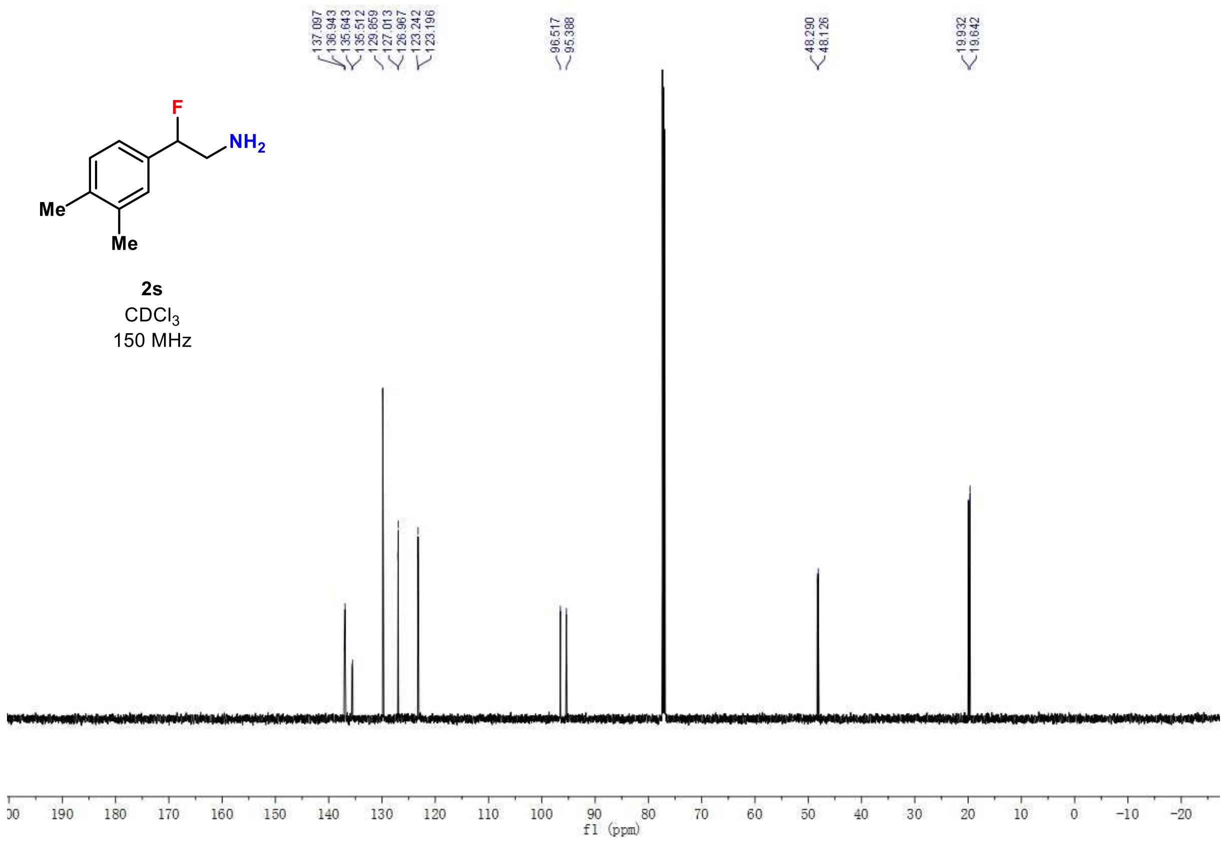

Supplementary Fig. 92. **^{13}C NMR** Spectra of **2s**

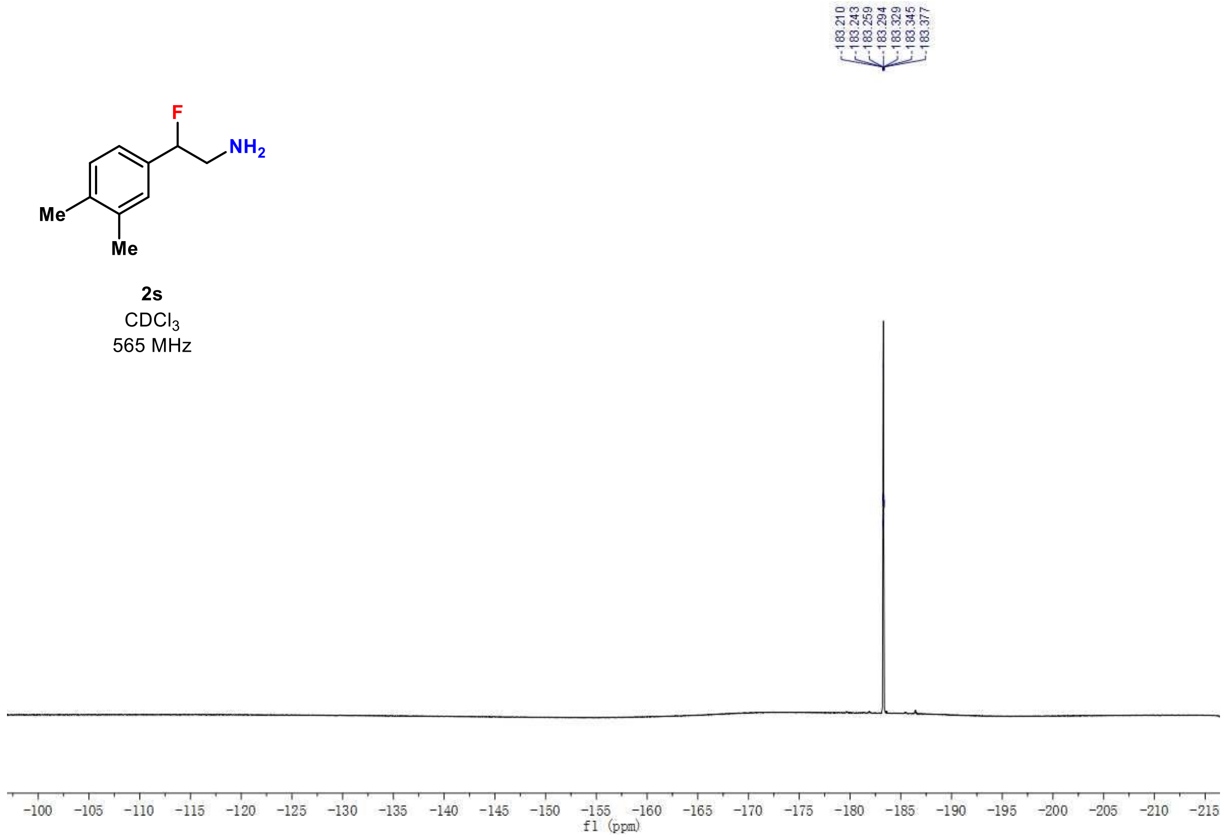

Supplementary Fig. 93. **^{19}F NMR** Spectra of **2s**

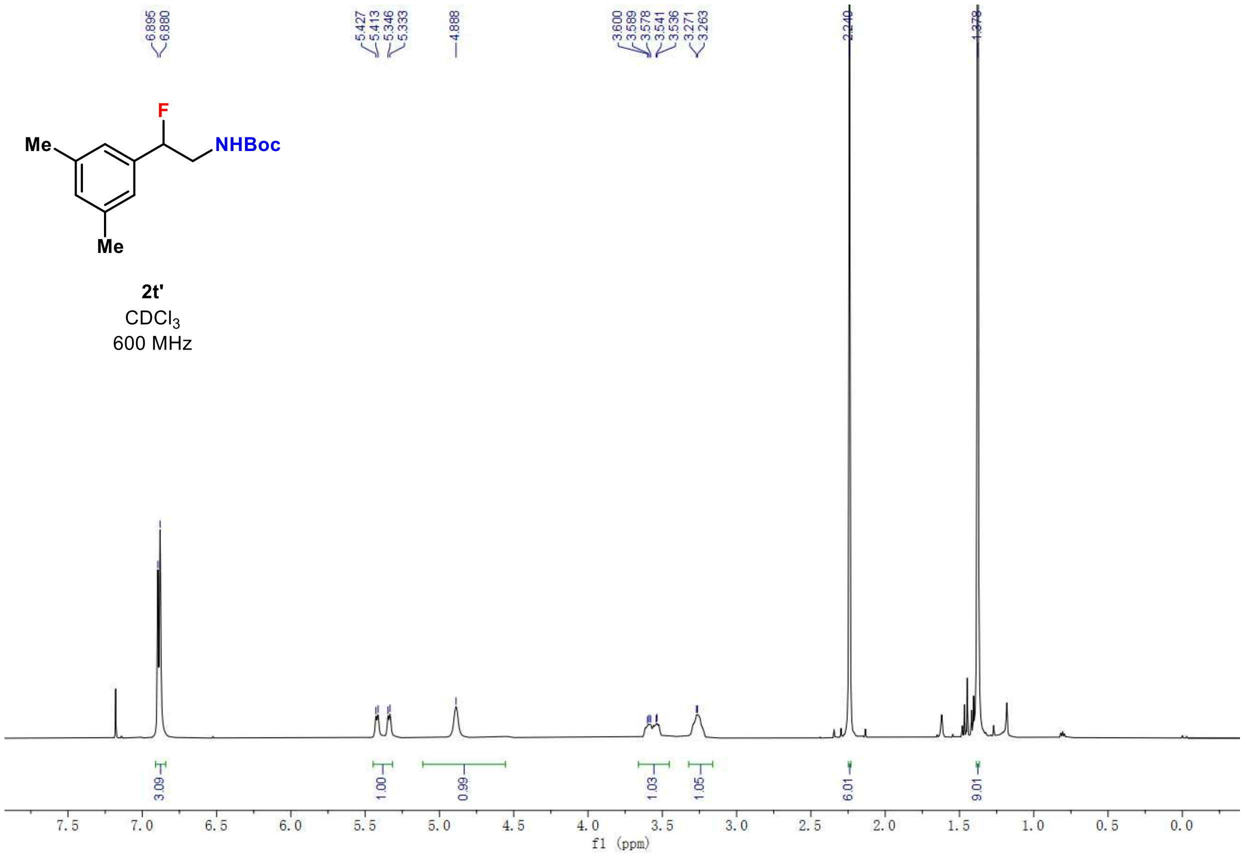

Supplementary Fig. 94. **^{1}H NMR** Spectra of **2t'**

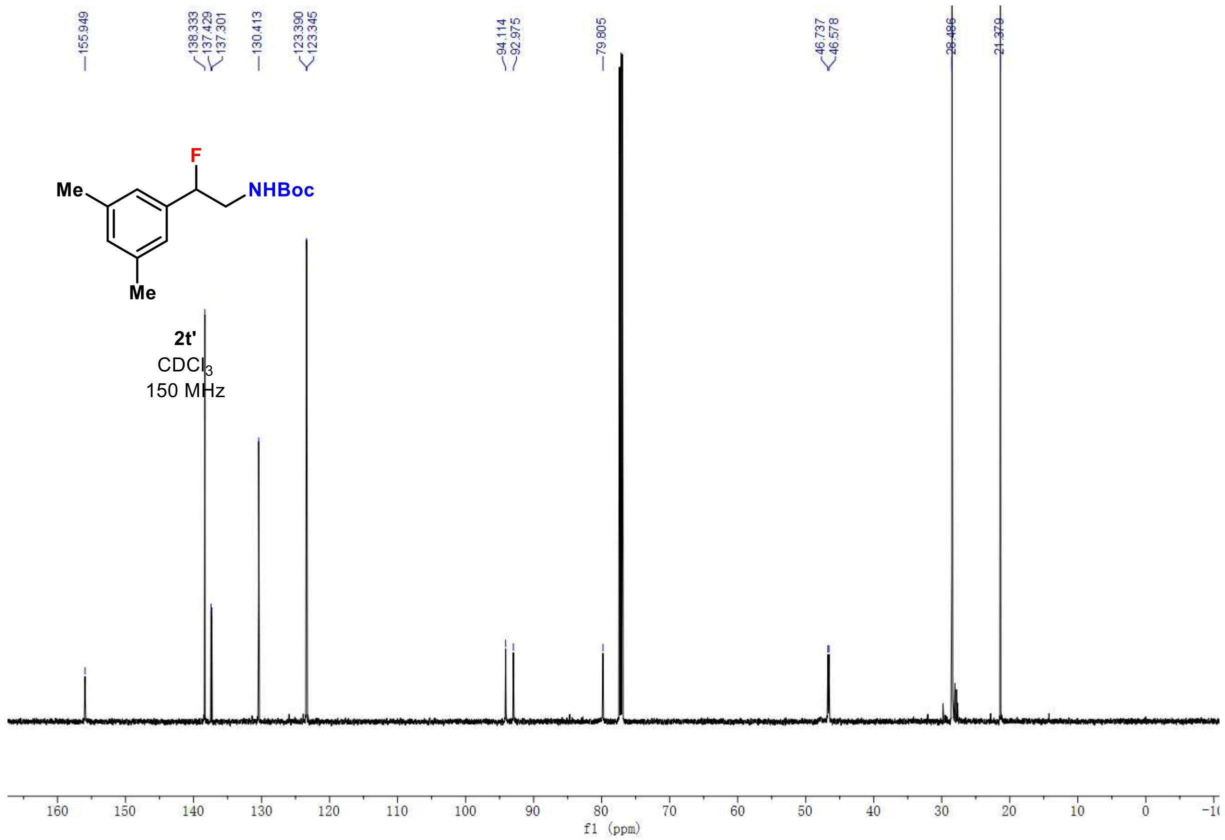

Supplementary Fig. 95. ^{13}**C NMR** Spectra of **2t'**

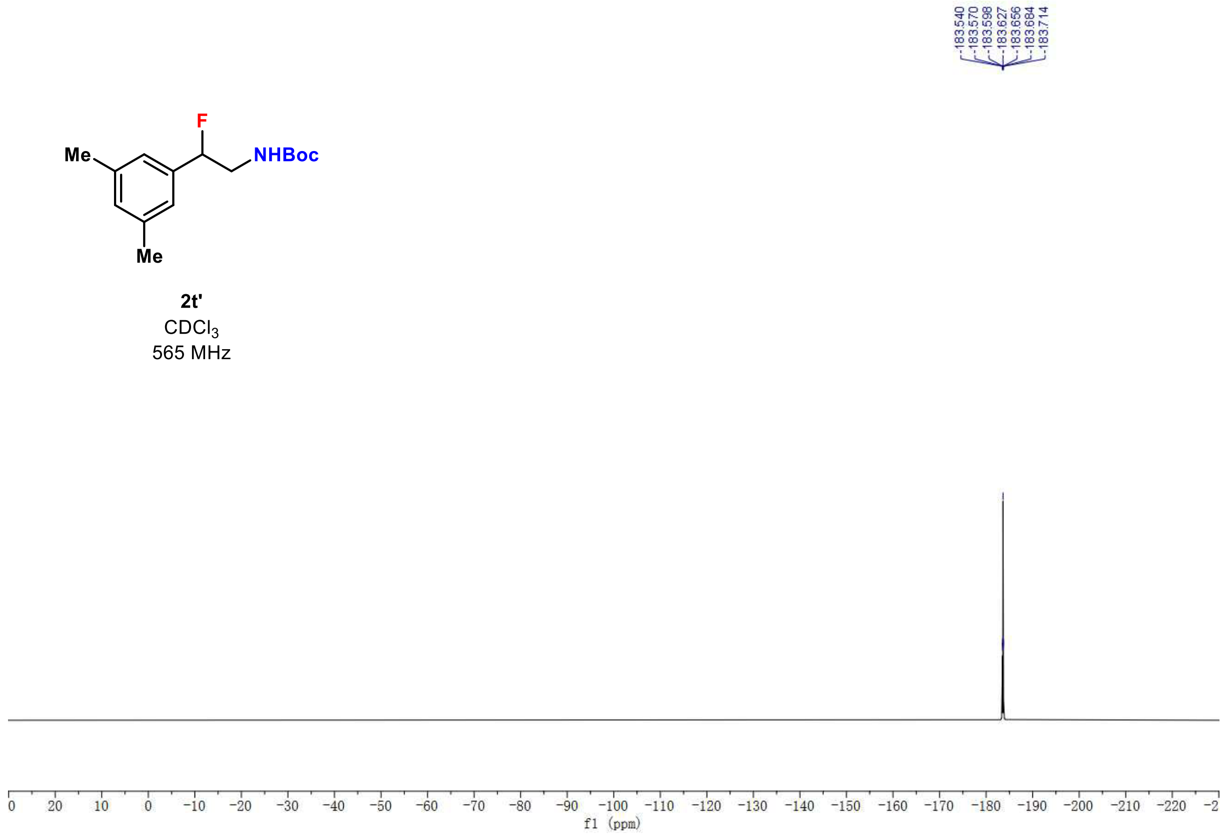

Supplementary Fig. 96. ^{19}**F NMR** Spectra of **2t'**

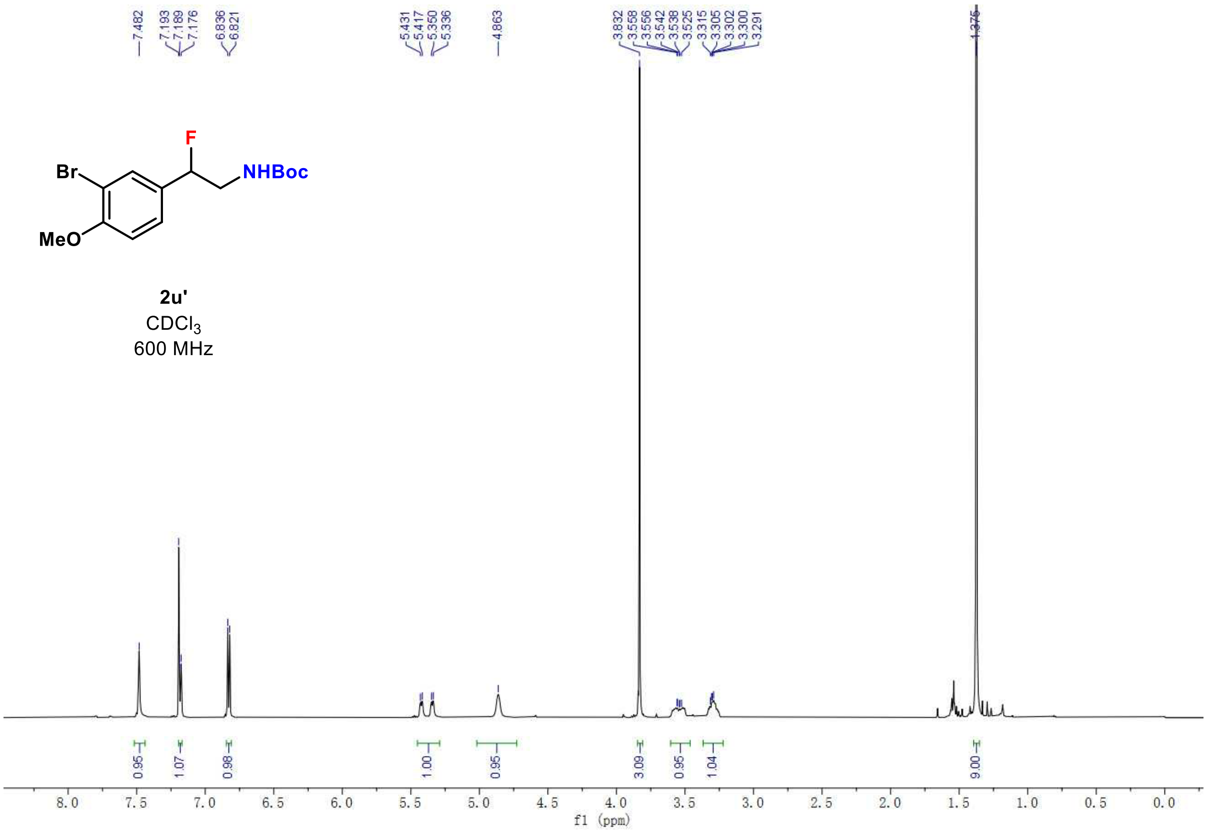

Supplementary Fig. 97. **^{1}H NMR** Spectra of **2u'**

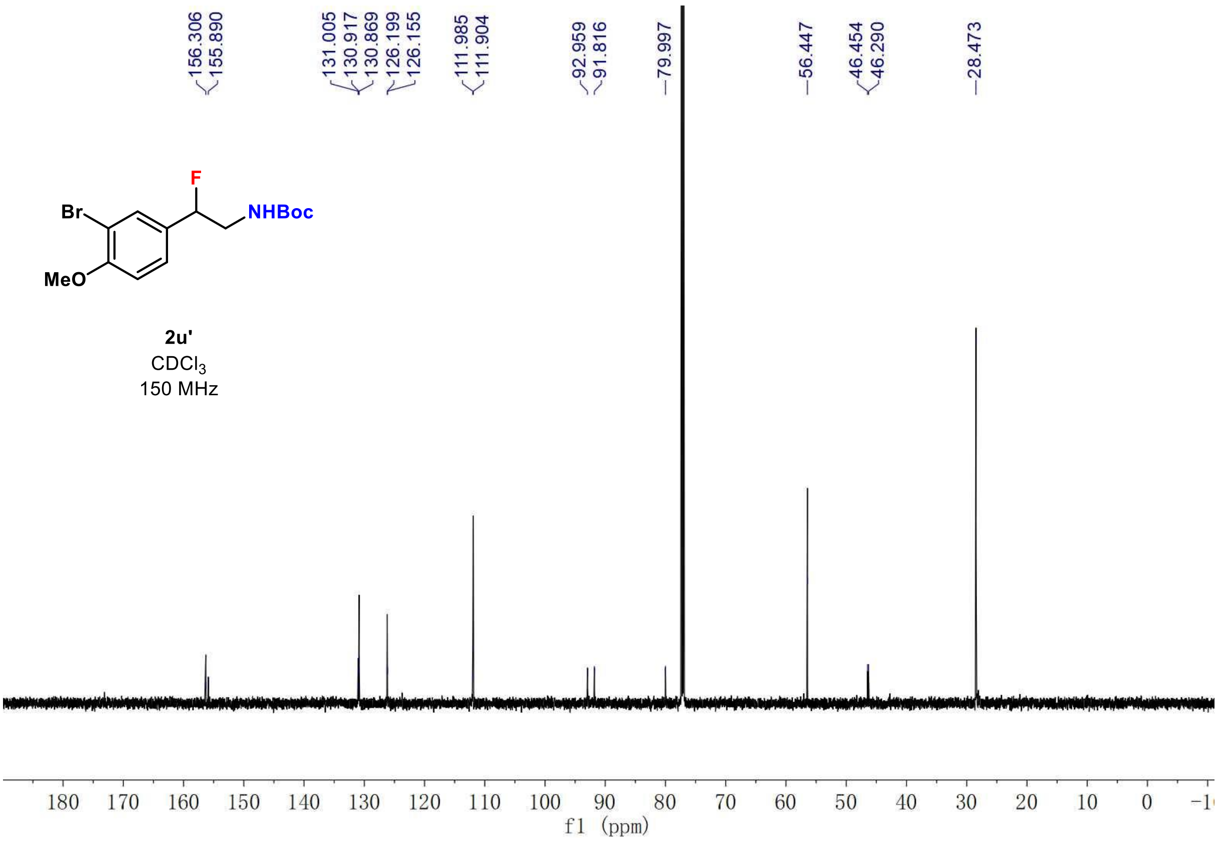

Supplementary Fig. 98. **^{13}C NMR** Spectra of **2u'**

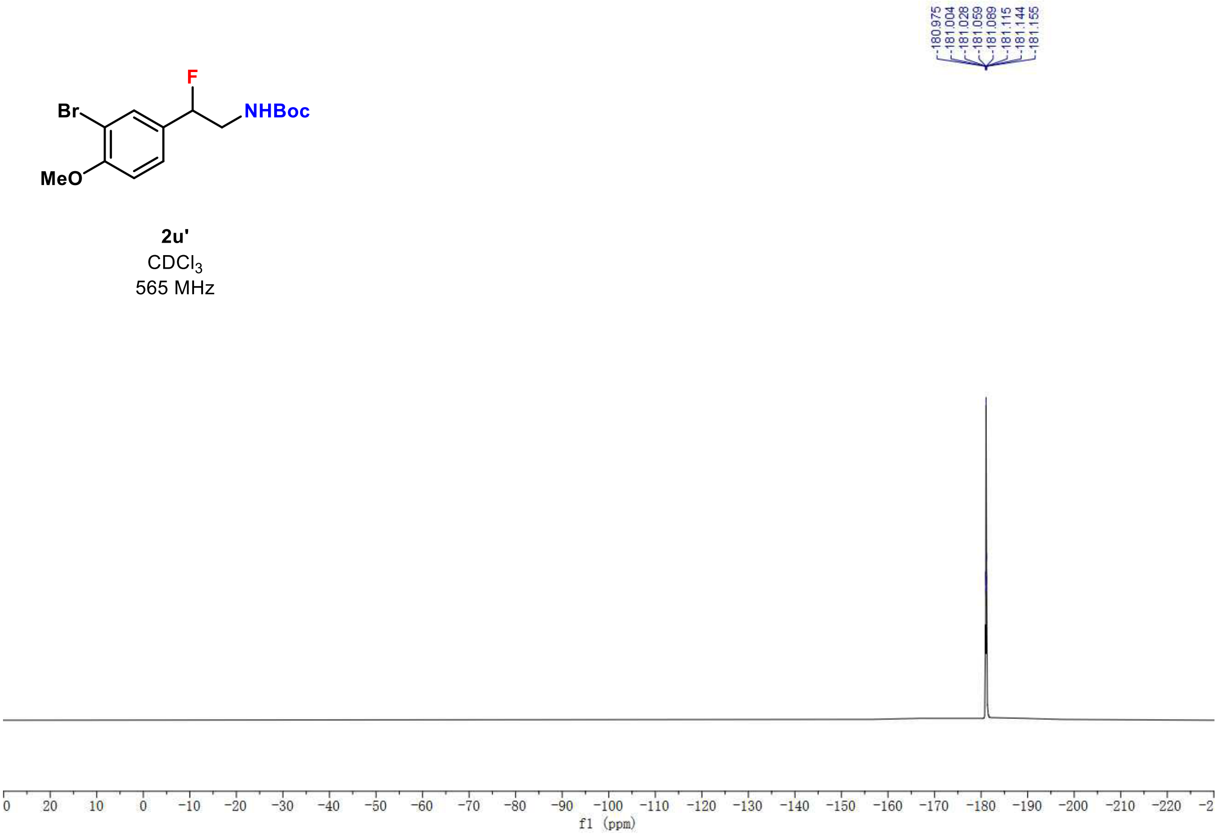

Supplementary Fig. 99. ^{19}**F NMR** Spectra of **2u'**

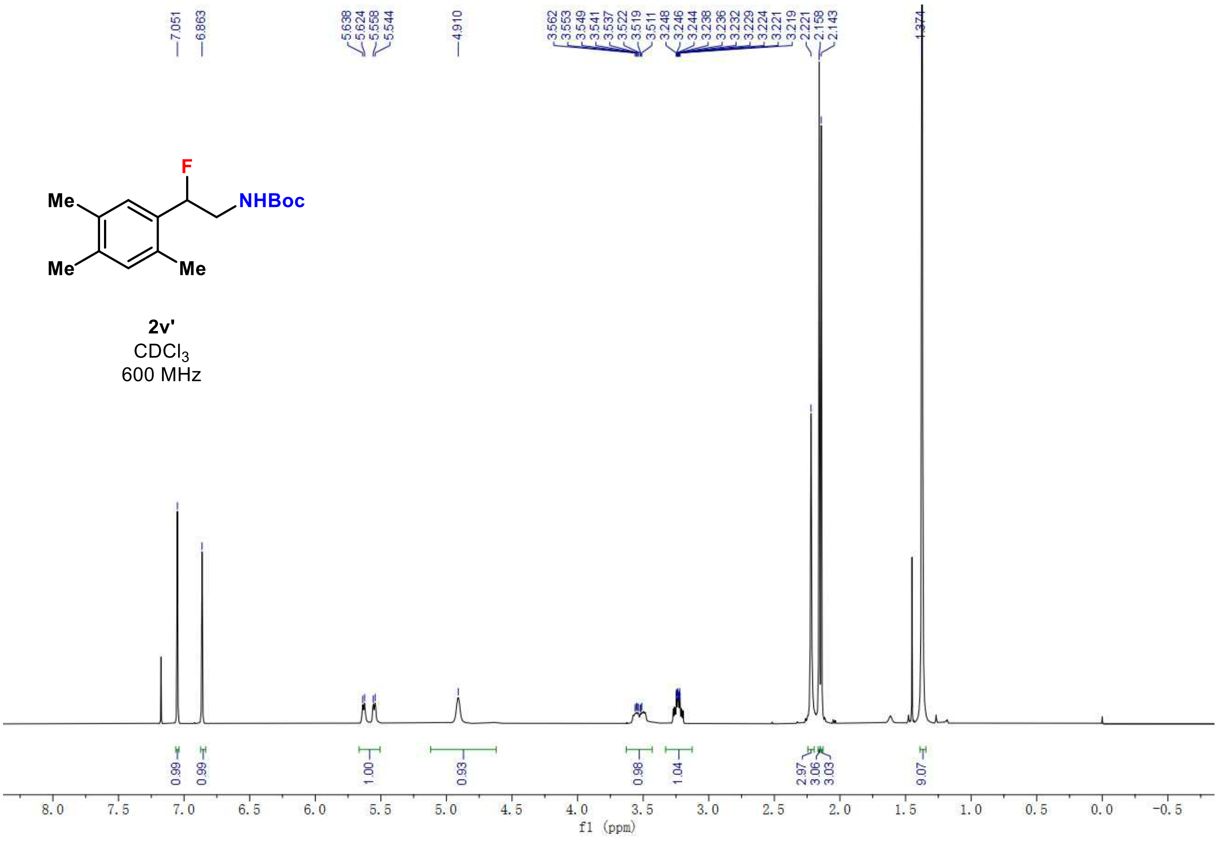

Supplementary Fig. 100. ^{1}**H NMR** Spectra of **2v'**

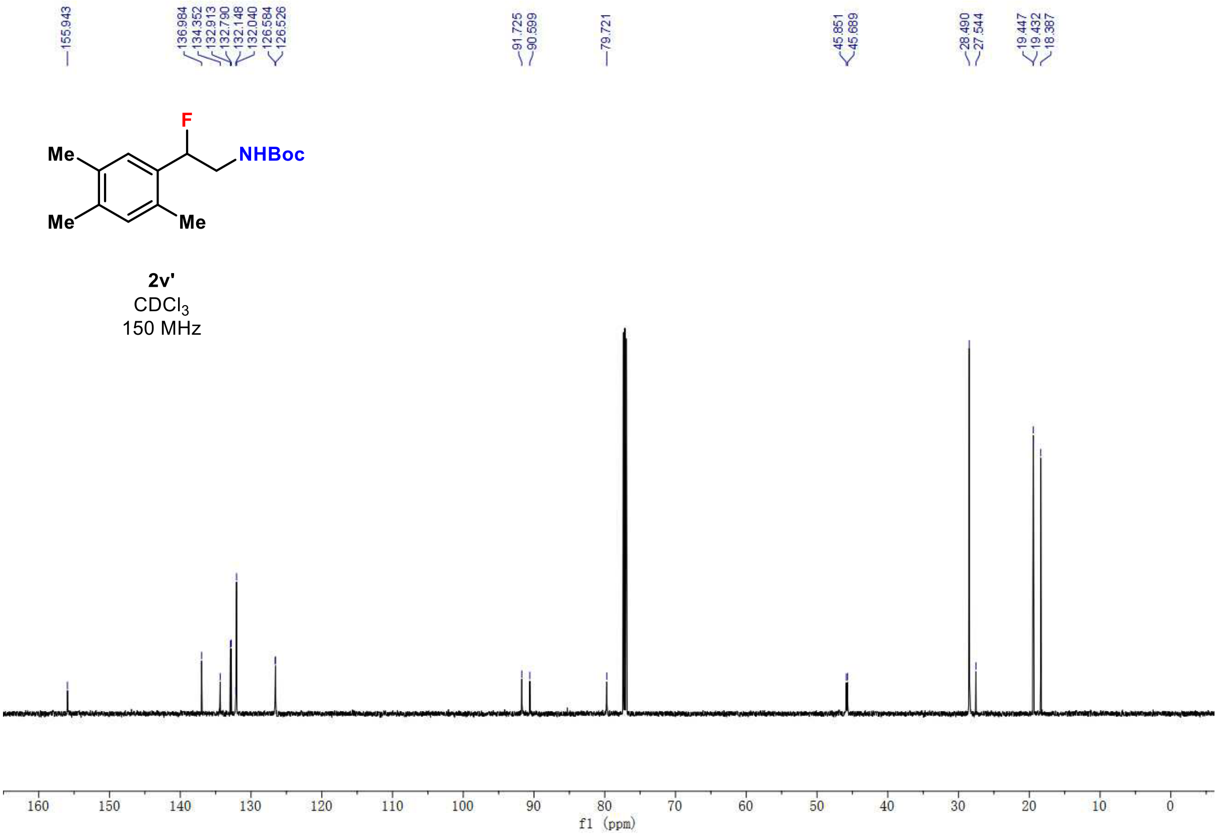

Supplementary Fig. 101. ^{13}C NMR Spectra of **2v'**

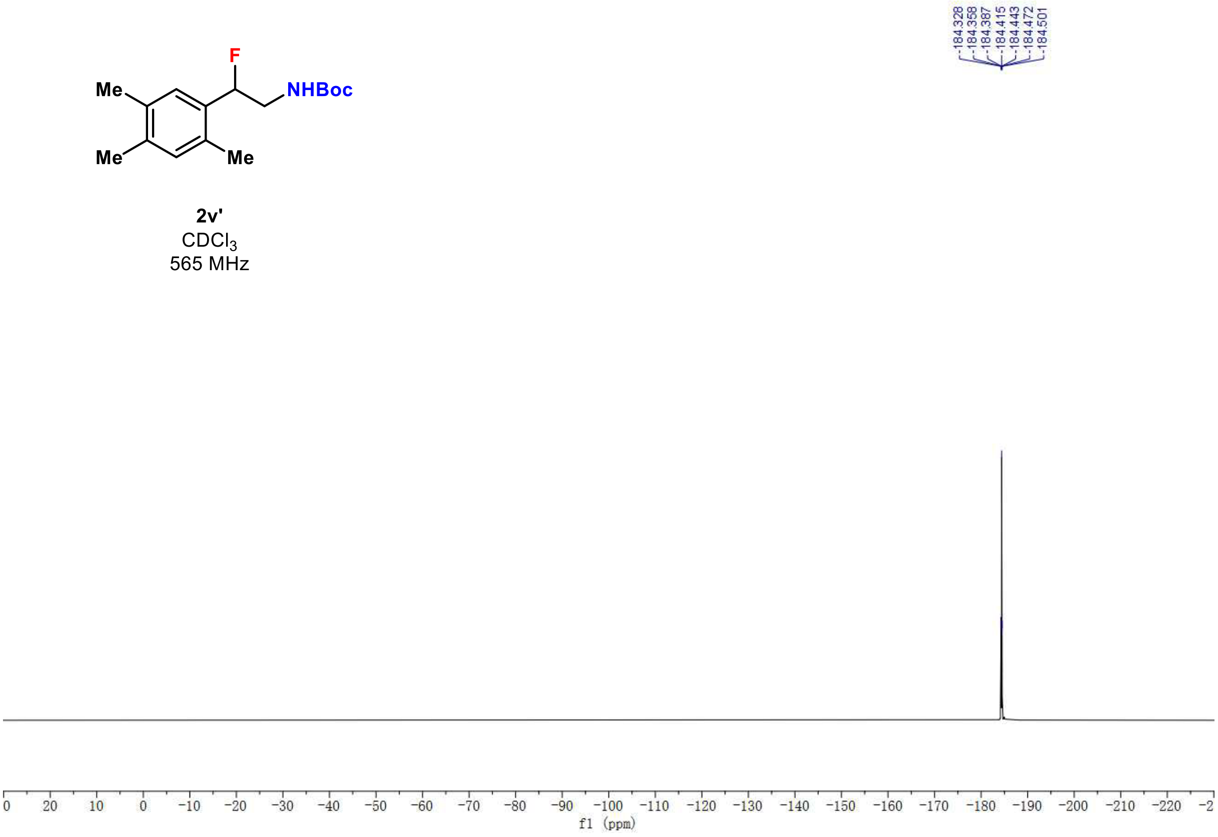

Supplementary Fig. 102. ^{19}F NMR Spectra of **2v'**

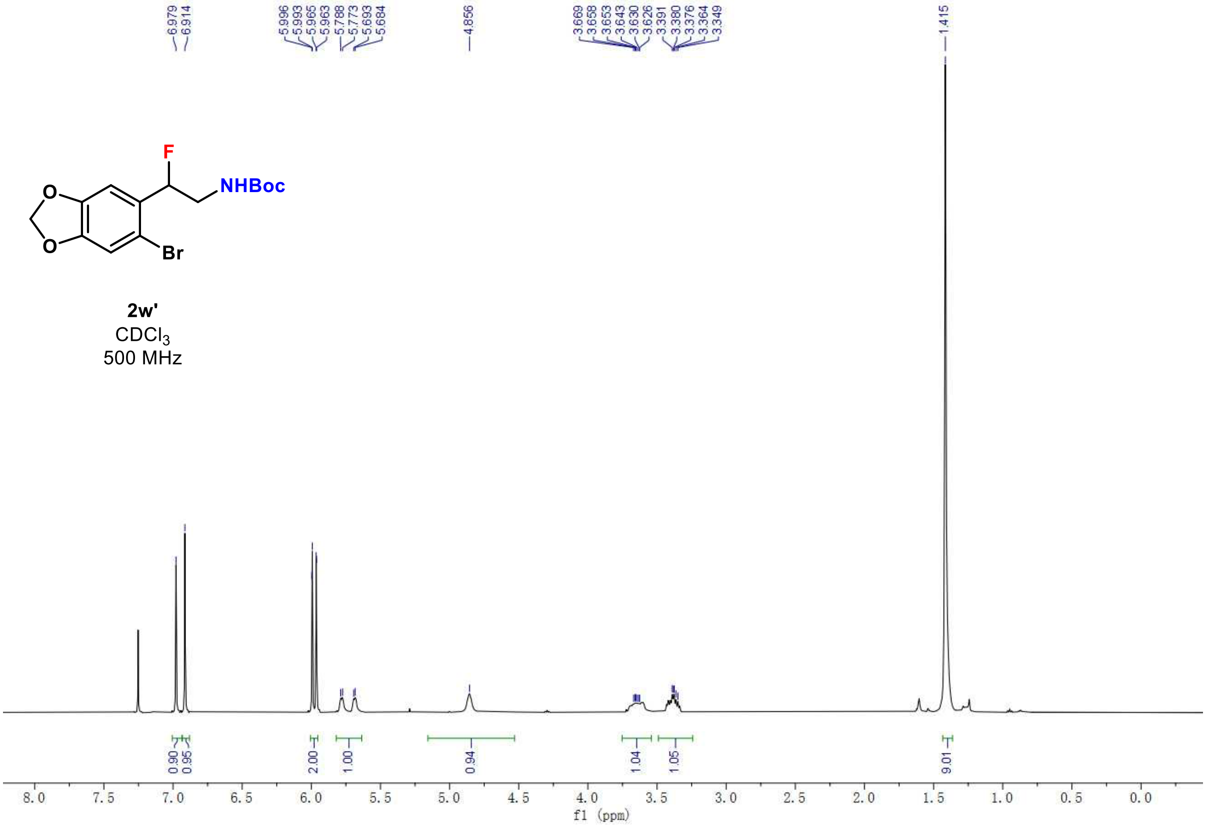

Supplementary Fig. 103. **^{1}H NMR** Spectra of **2w'**

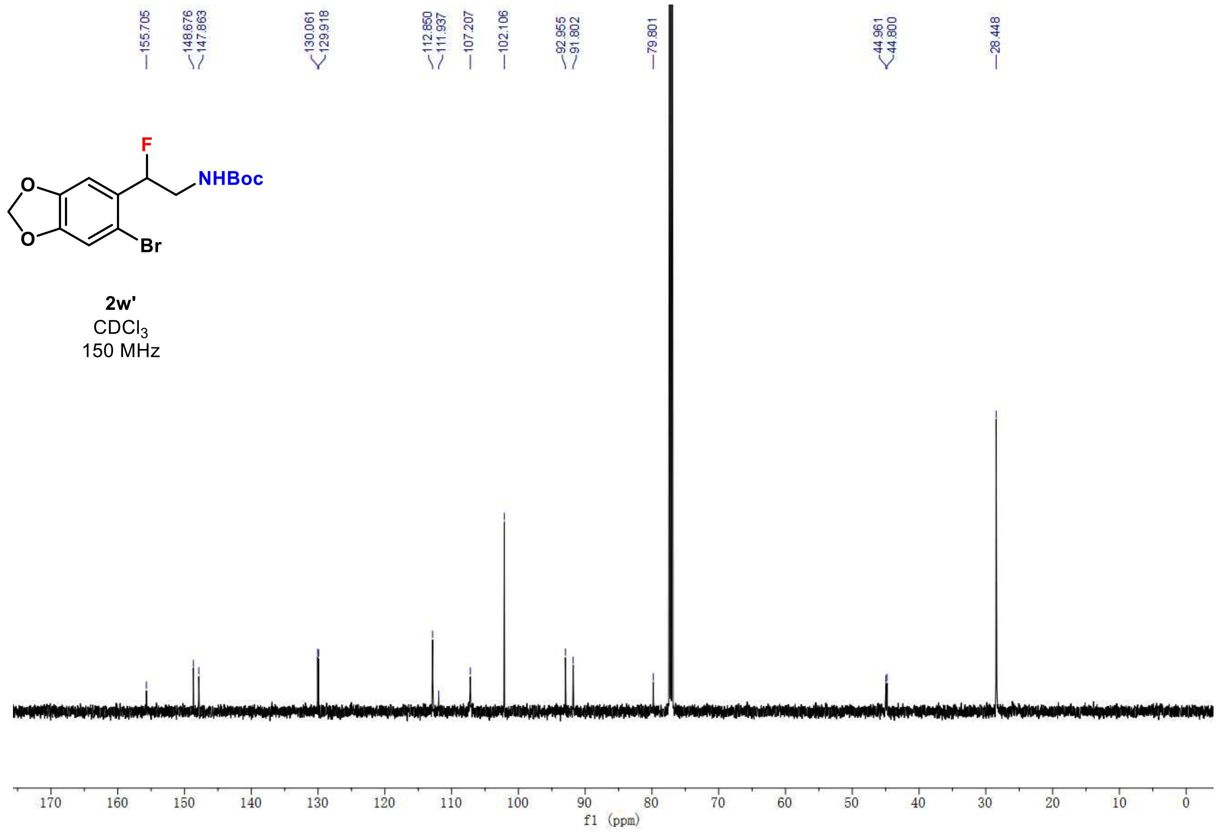

Supplementary Fig. 104. **^{13}C NMR** Spectra of **2w'**

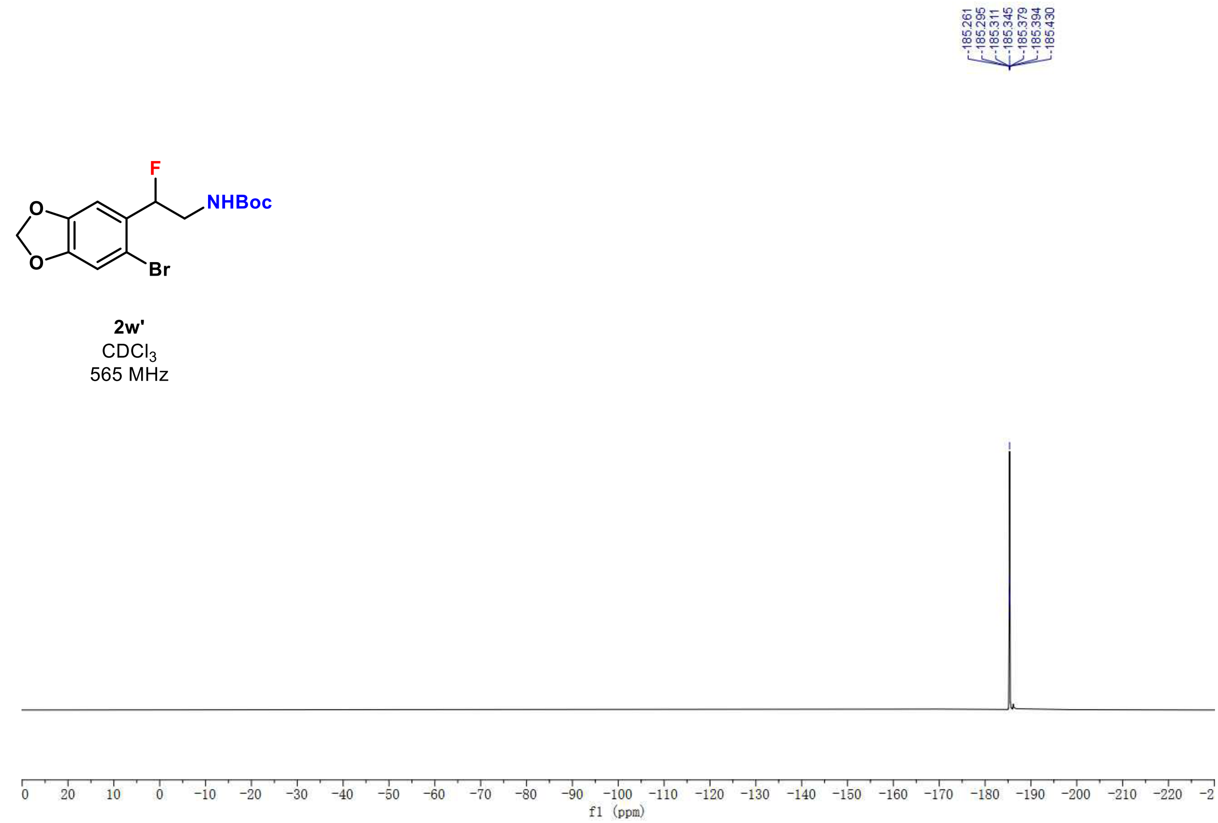

Supplementary Fig. 105. **^{19}F NMR** Spectra of **2w'**

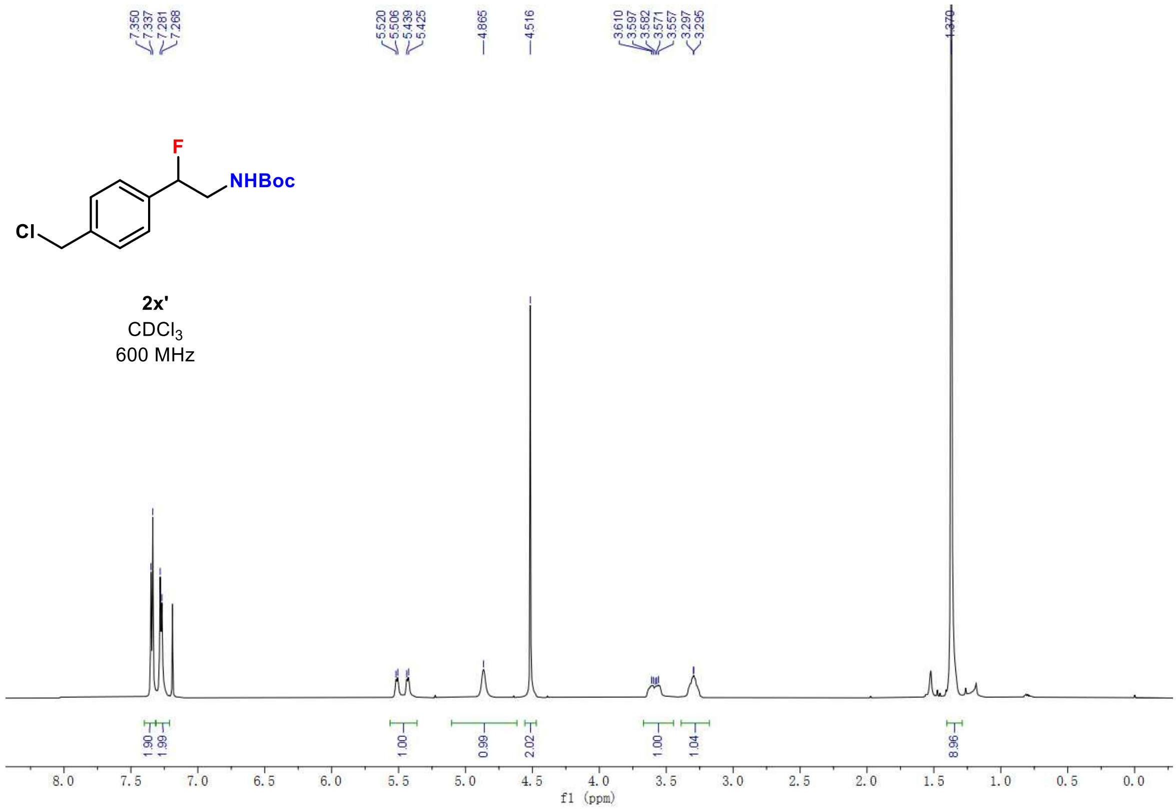

Supplementary Fig. 106. **^{1}H NMR** Spectra of **2x'**

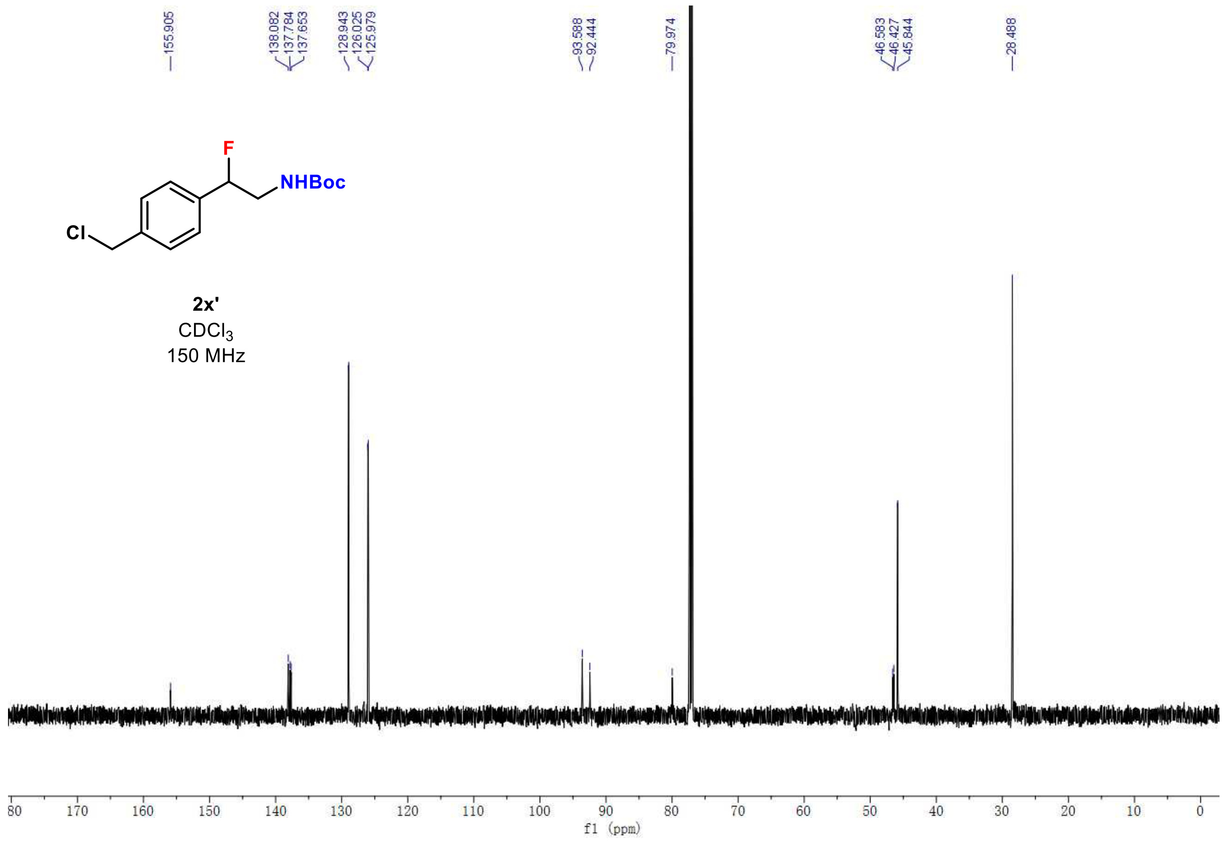

Supplementary Fig. 107. ^{13}**C NMR** Spectra of **2x'**

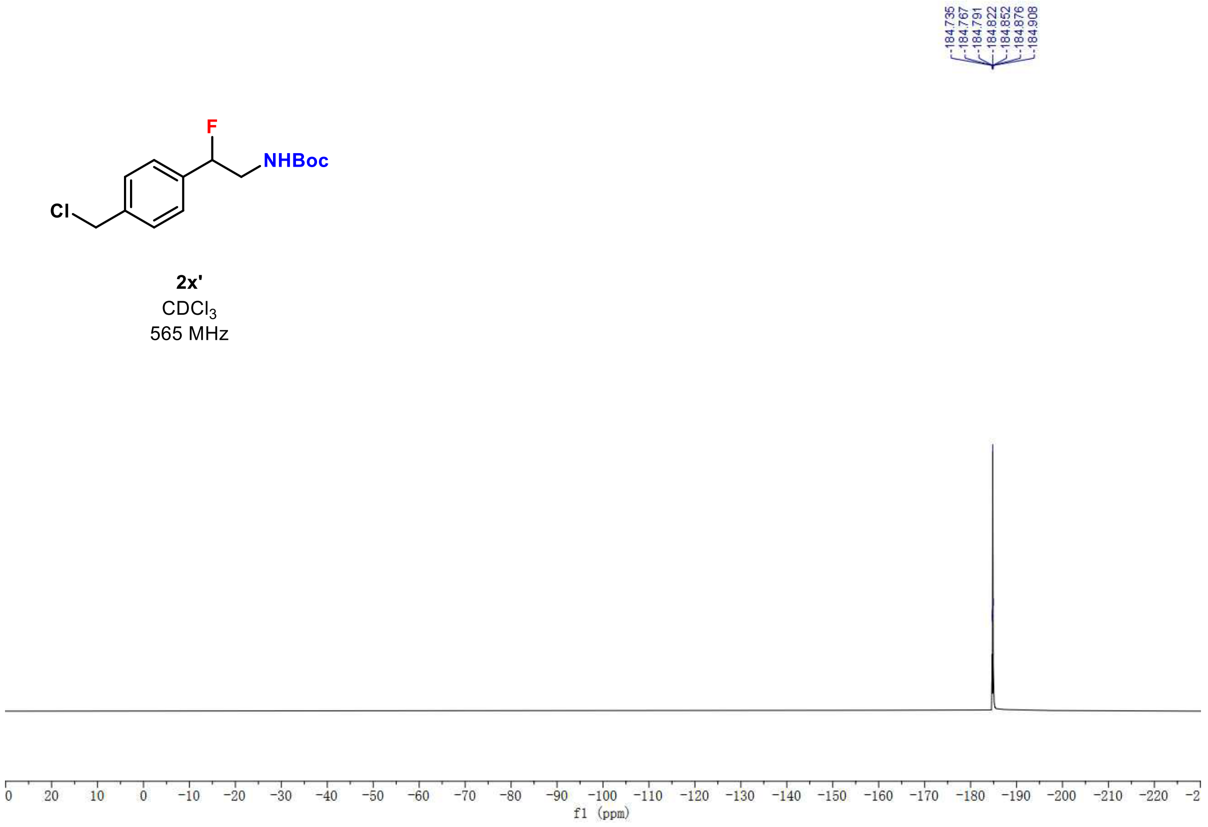

Supplementary Fig. 108. ^{19}**F NMR** Spectra of **2x'**

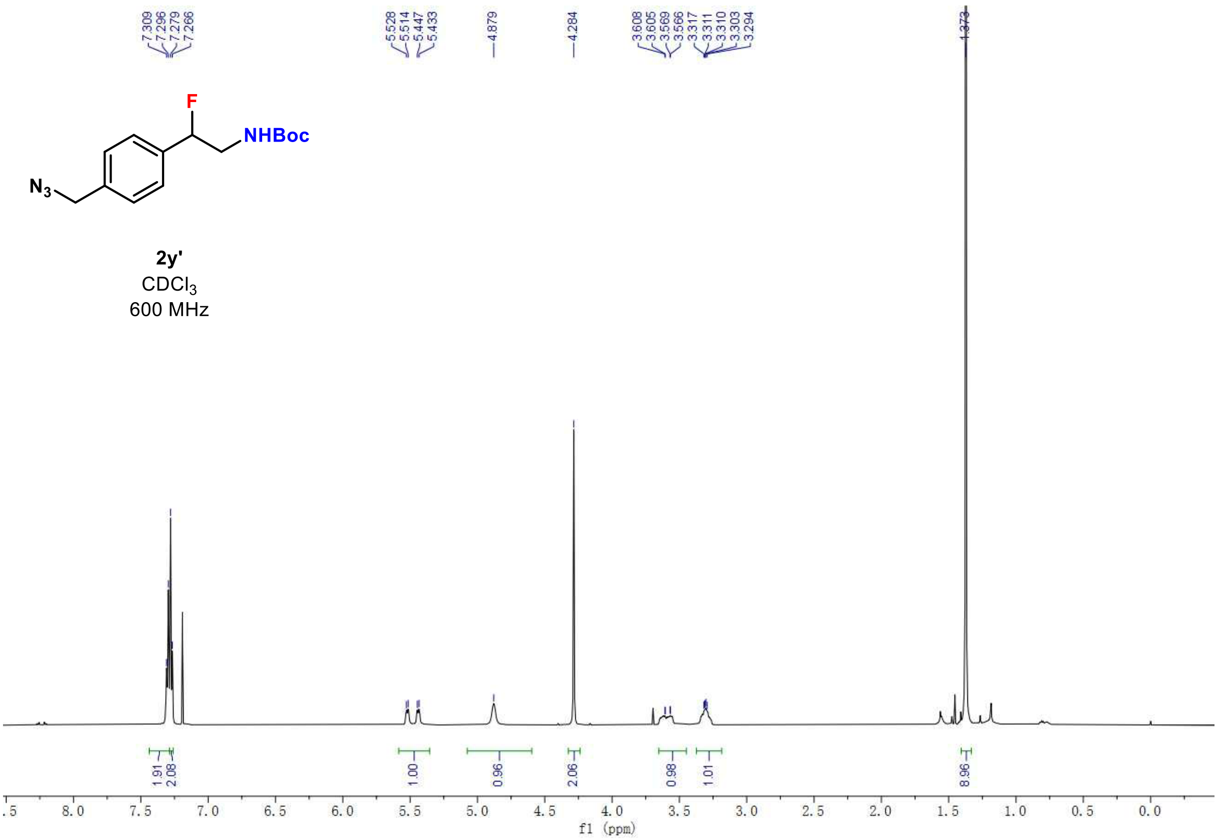

Supplementary Fig. 109. ^{1}**H NMR** Spectra of **2y'**

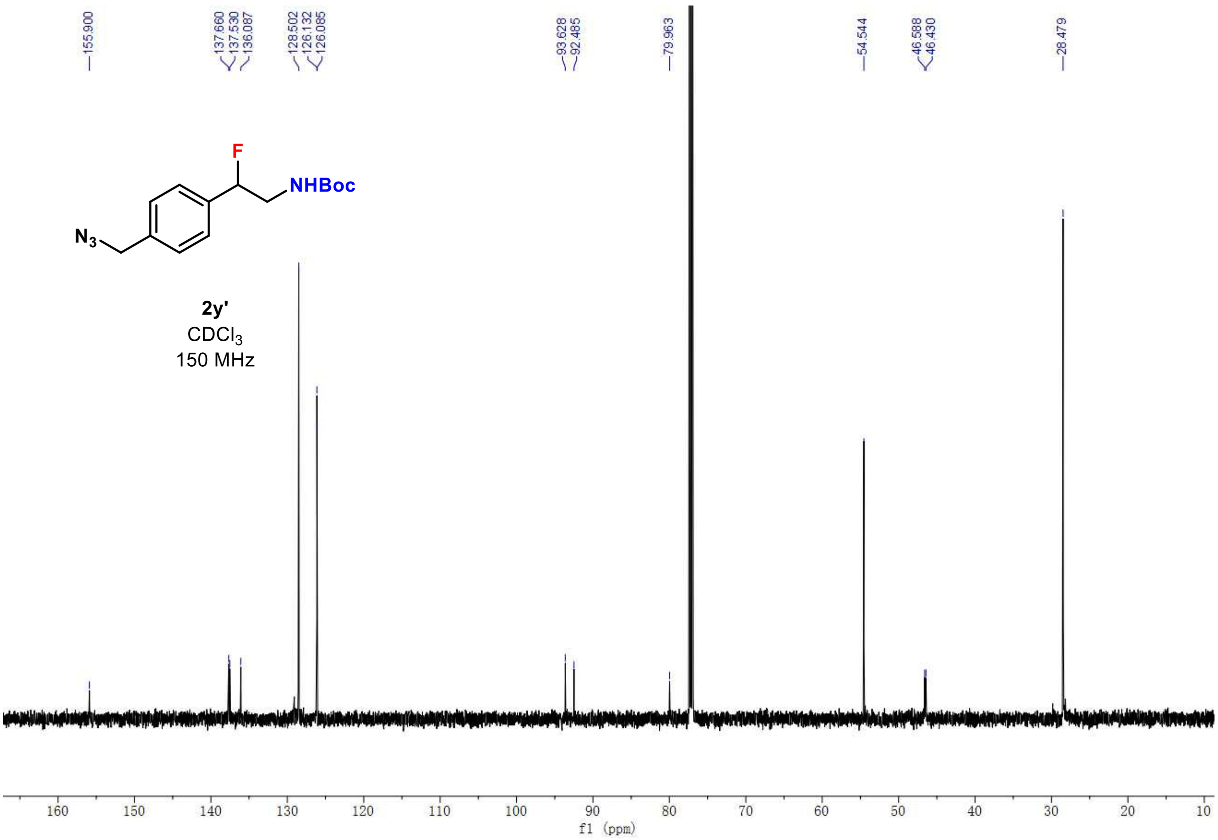

Supplementary Fig. 110. ^{13}**C NMR** Spectra of **2y'**

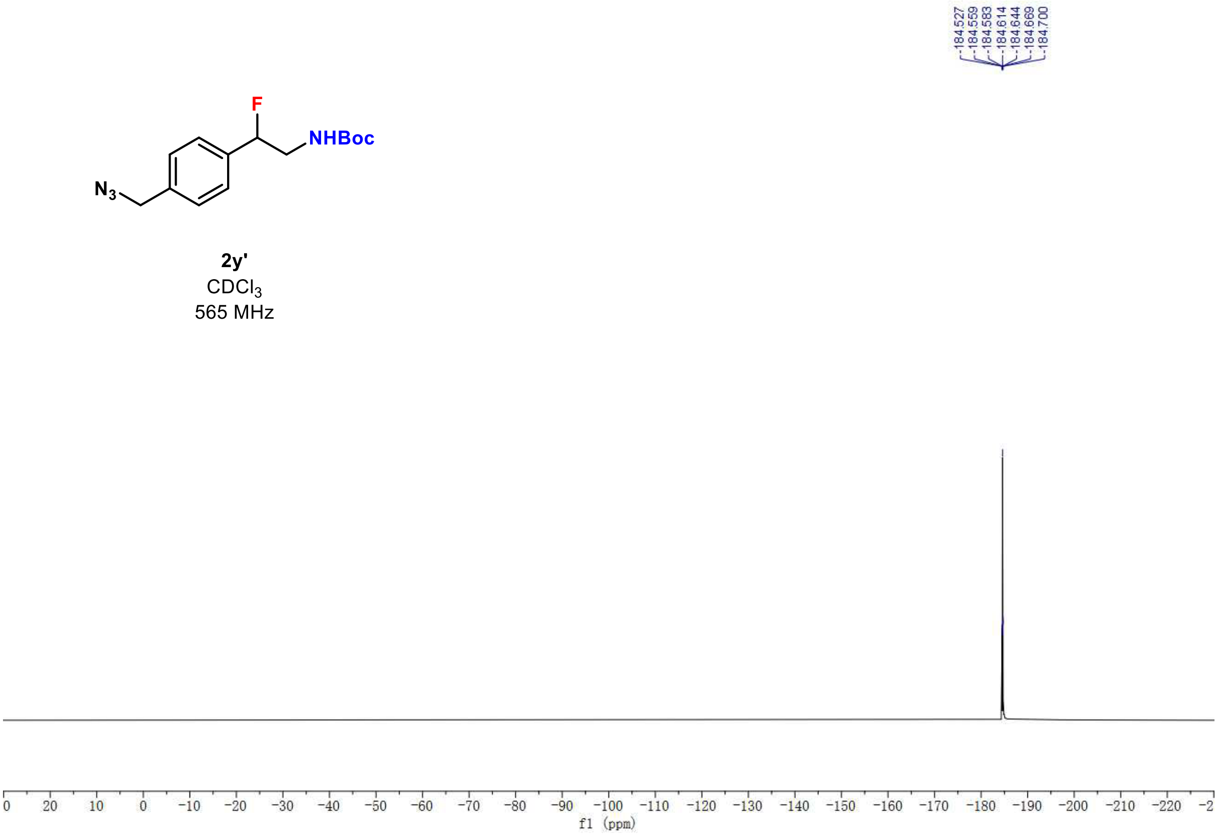

Supplementary Fig. 111. ^{19}F NMR Spectra of **2y'**

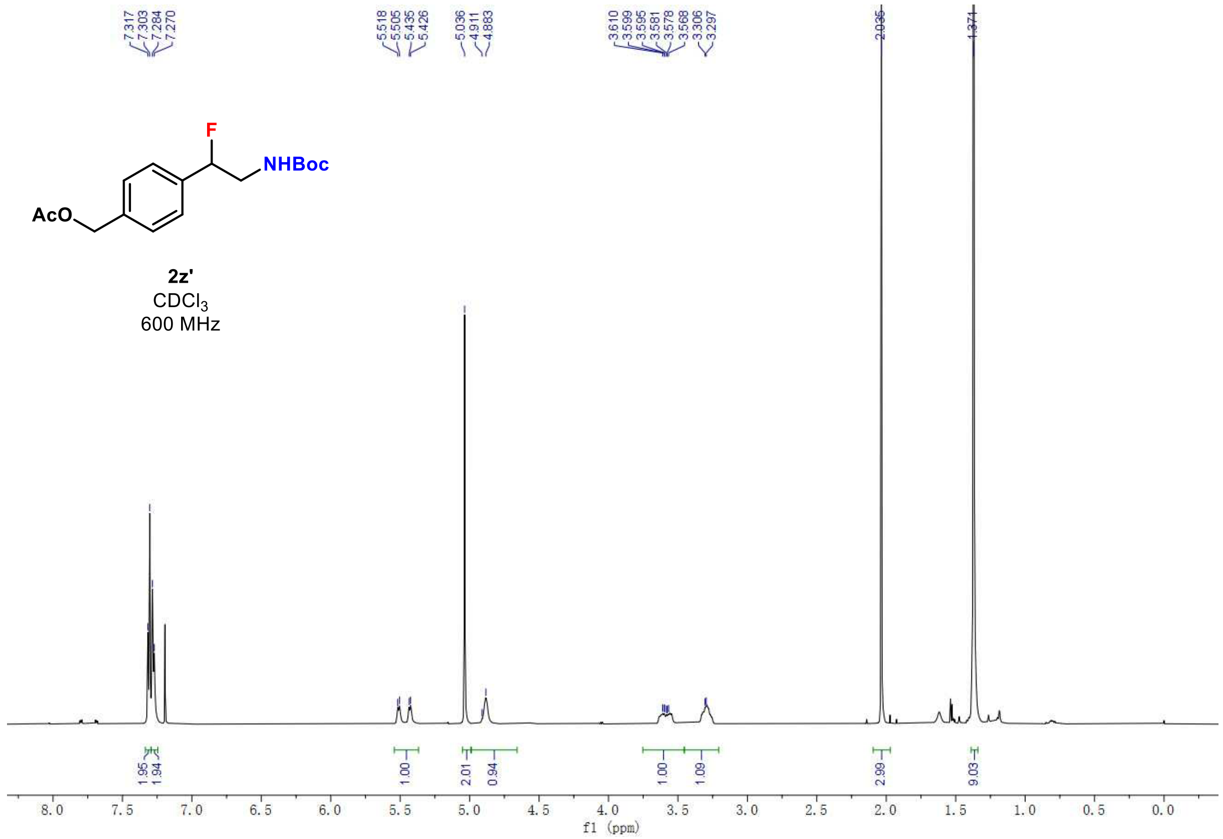

Supplementary Fig. 112. ^{1}H NMR Spectra of **2z'**

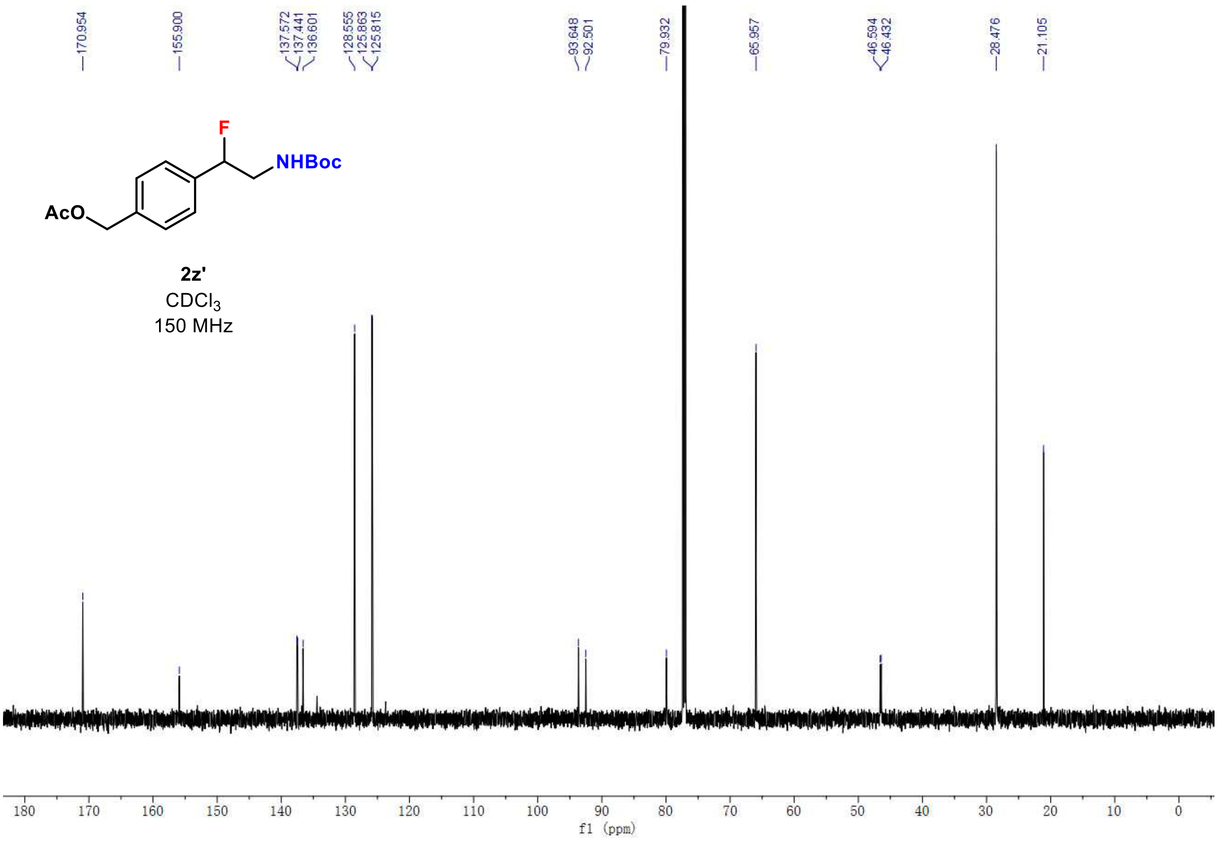

Supplementary Fig. 113. ^{13}**C NMR** Spectra of **2z'**

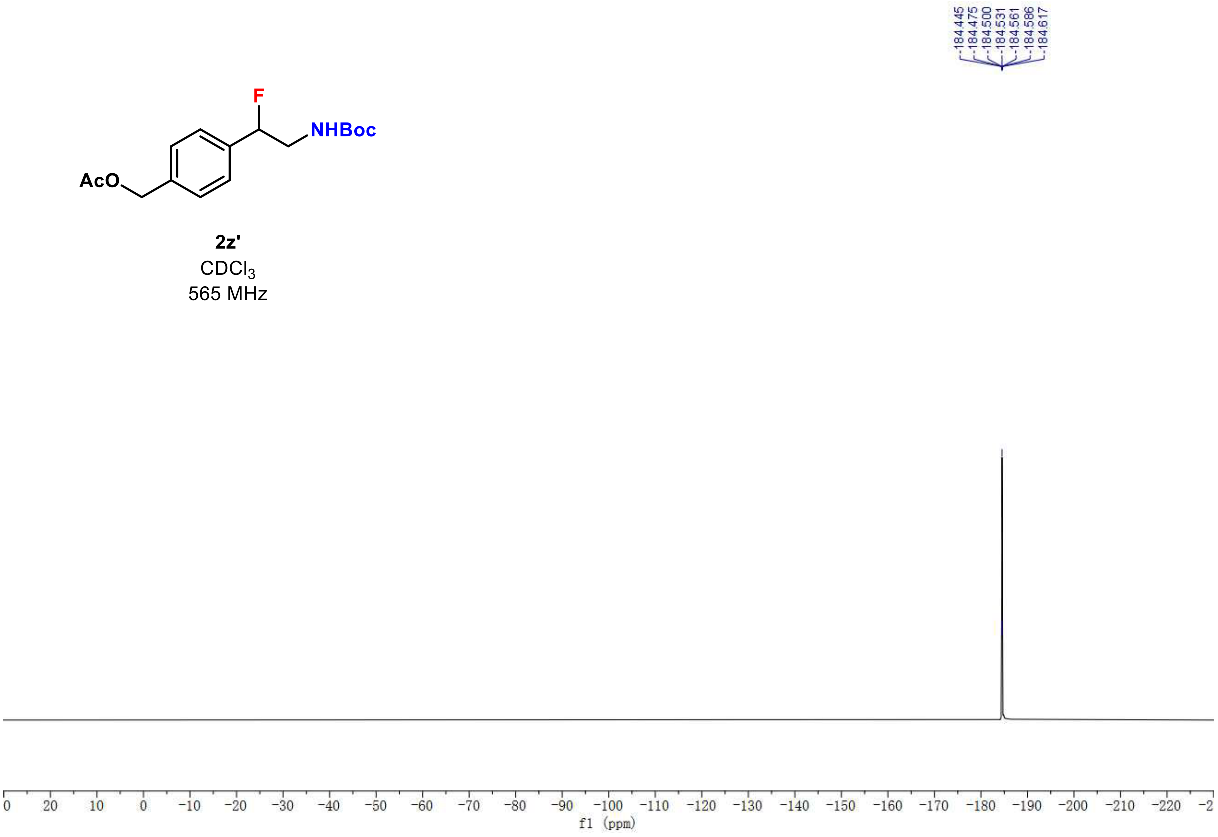

Supplementary Fig. 114. ^{19}**F NMR** Spectra of **2z'**

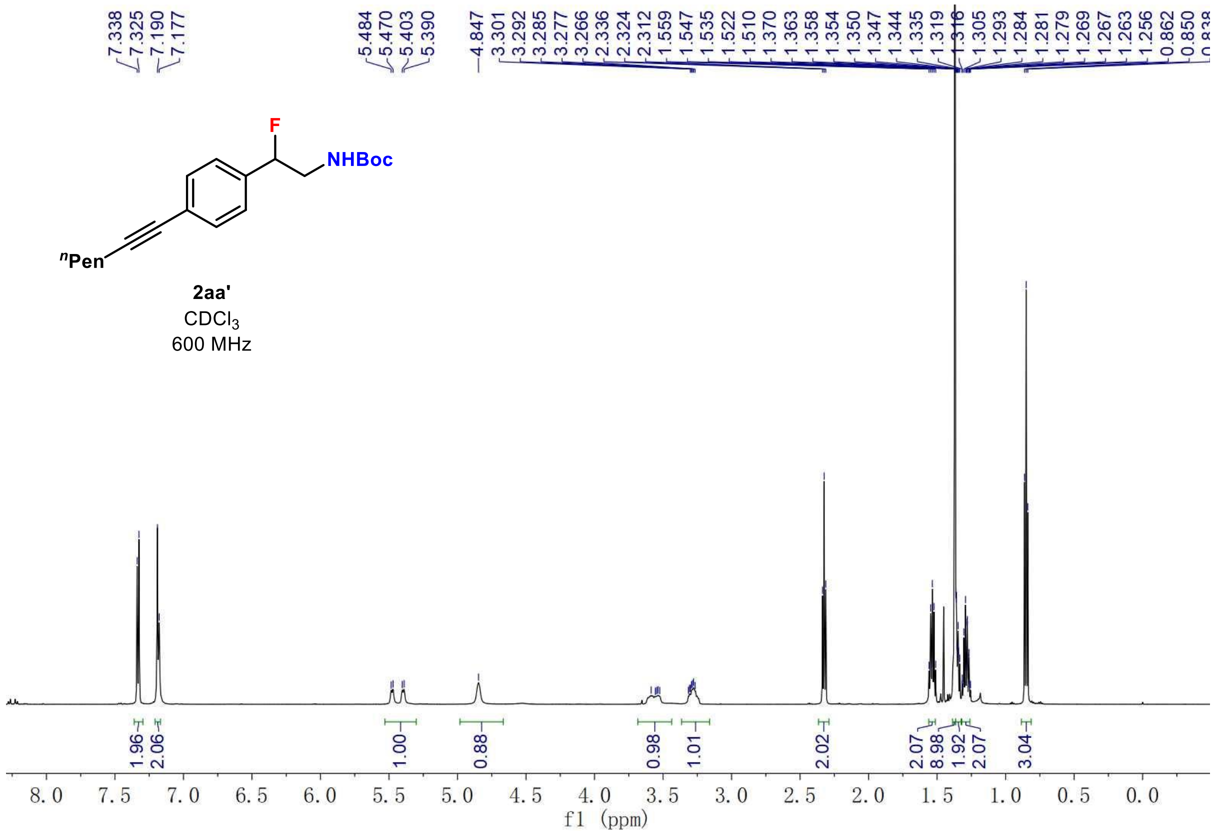

Supplementary Fig. 115. ^{1}**H NMR** Spectra of **2aa'**

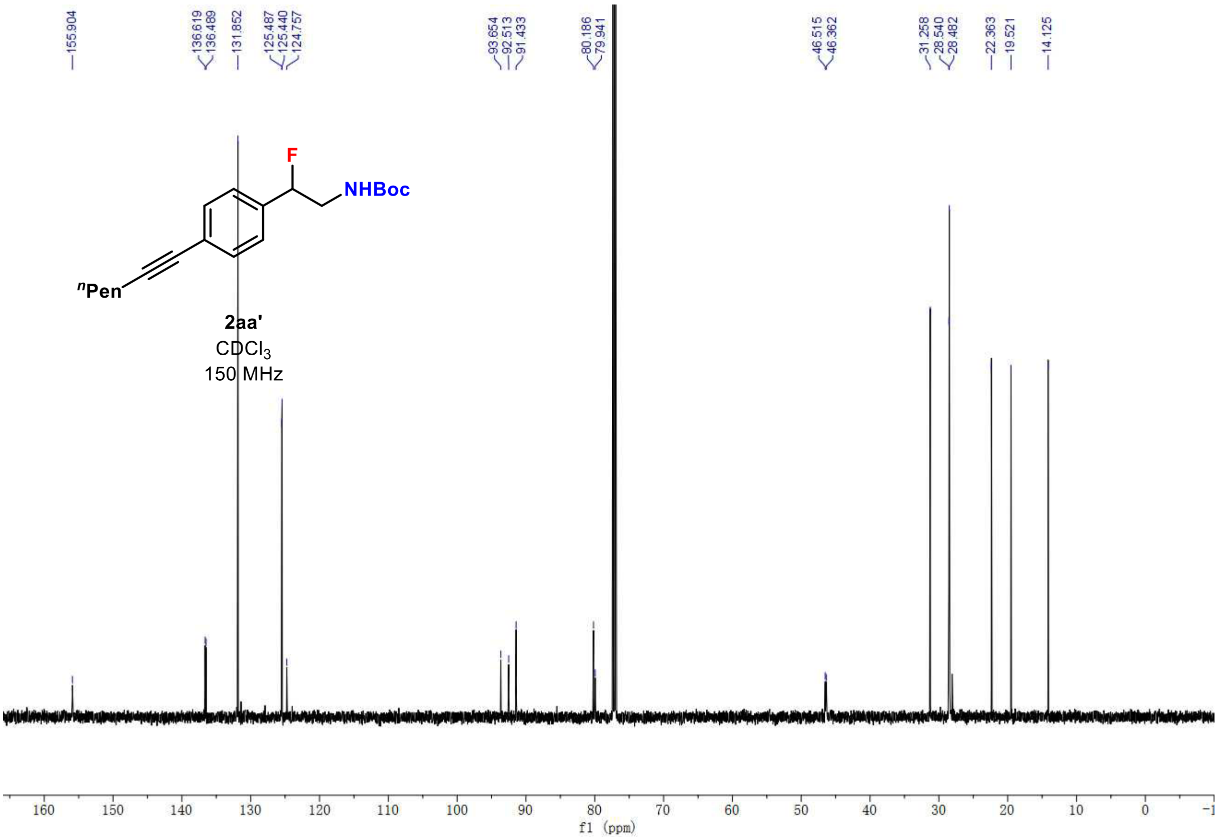

Supplementary Fig. 116. ^{13}**C NMR** Spectra of **2aa'**

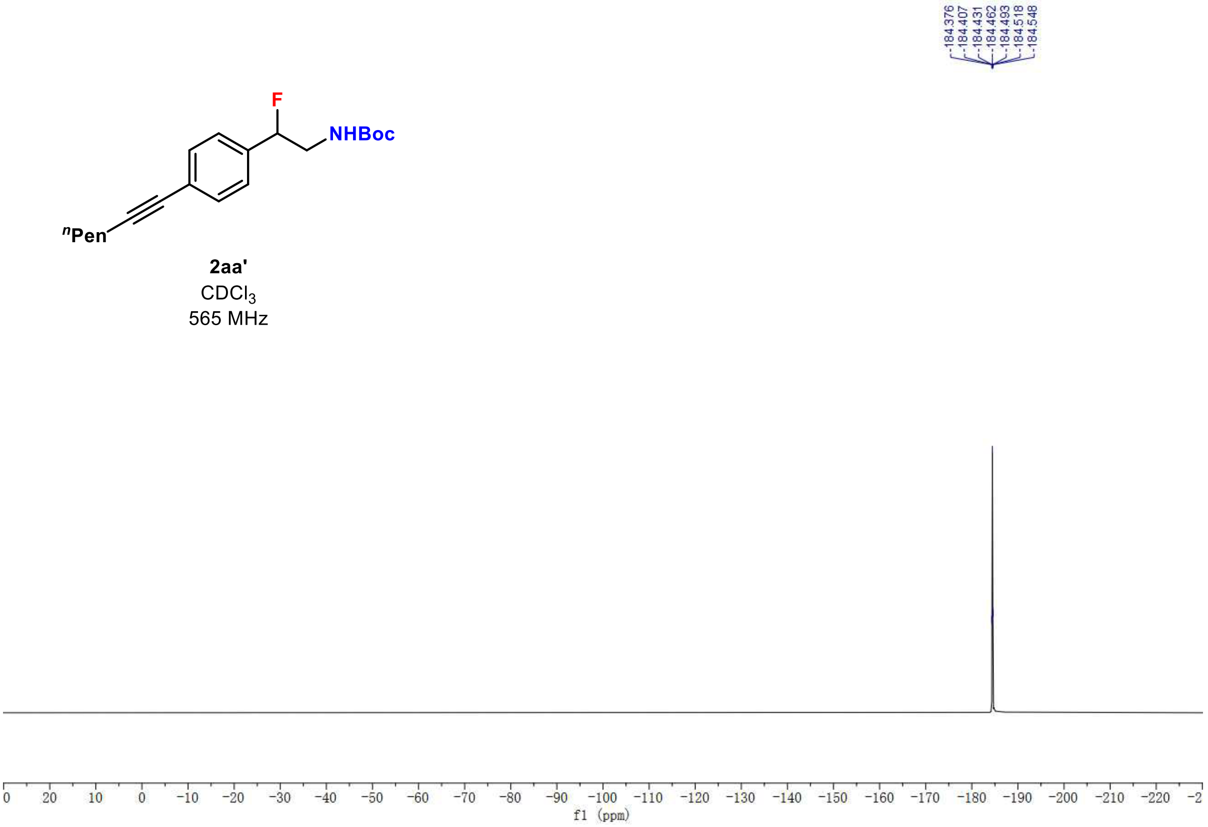

Supplementary Fig. 117. ^{19}F NMR Spectra of **2aa'**

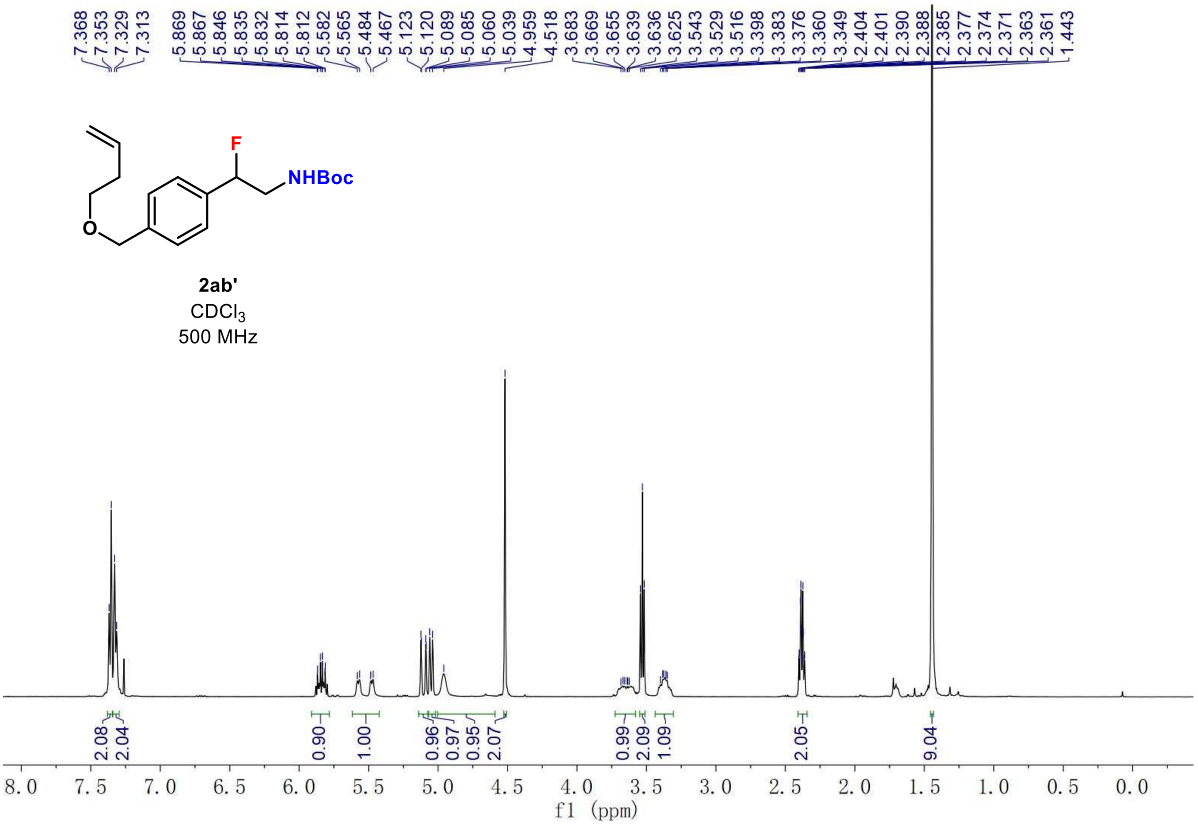

Supplementary Fig. 118. ^{1}H NMR Spectra of **2ab'**

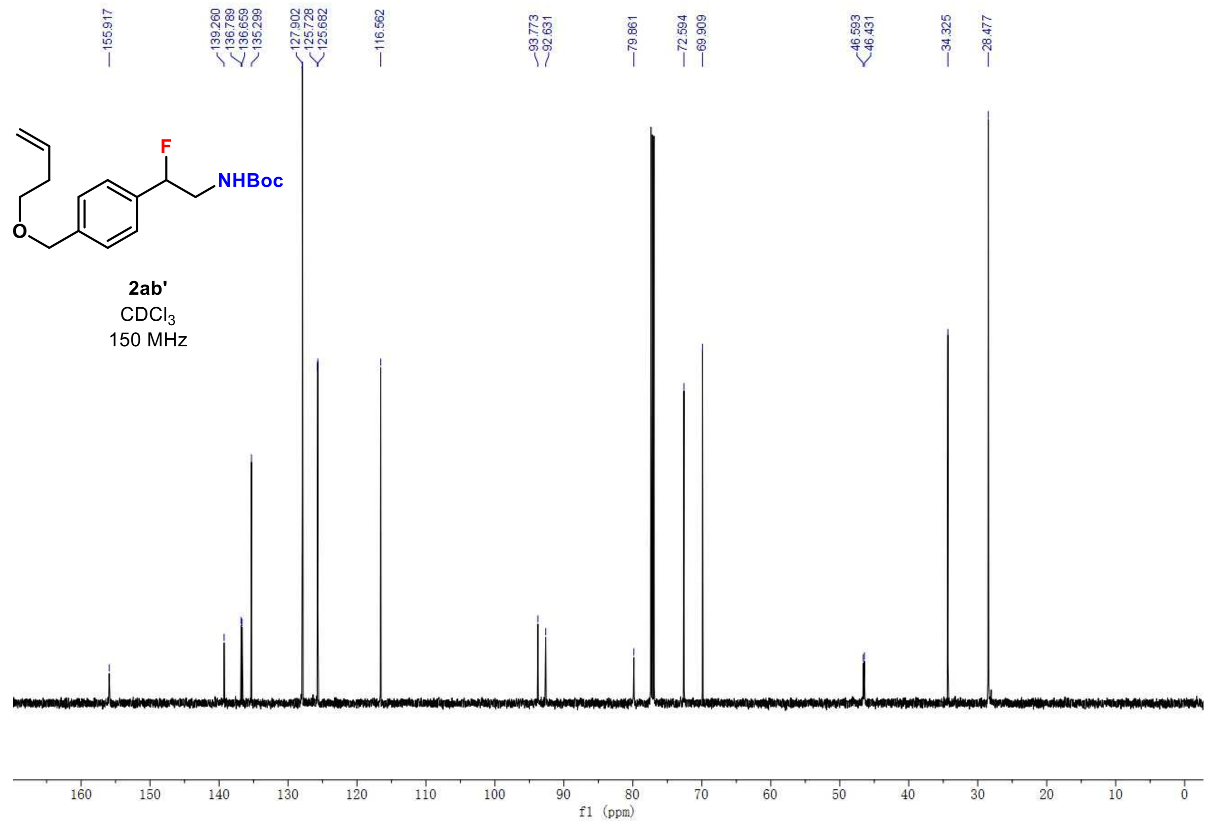

Supplementary Fig. 119. ^{13}**C NMR** Spectra of **2ab'**

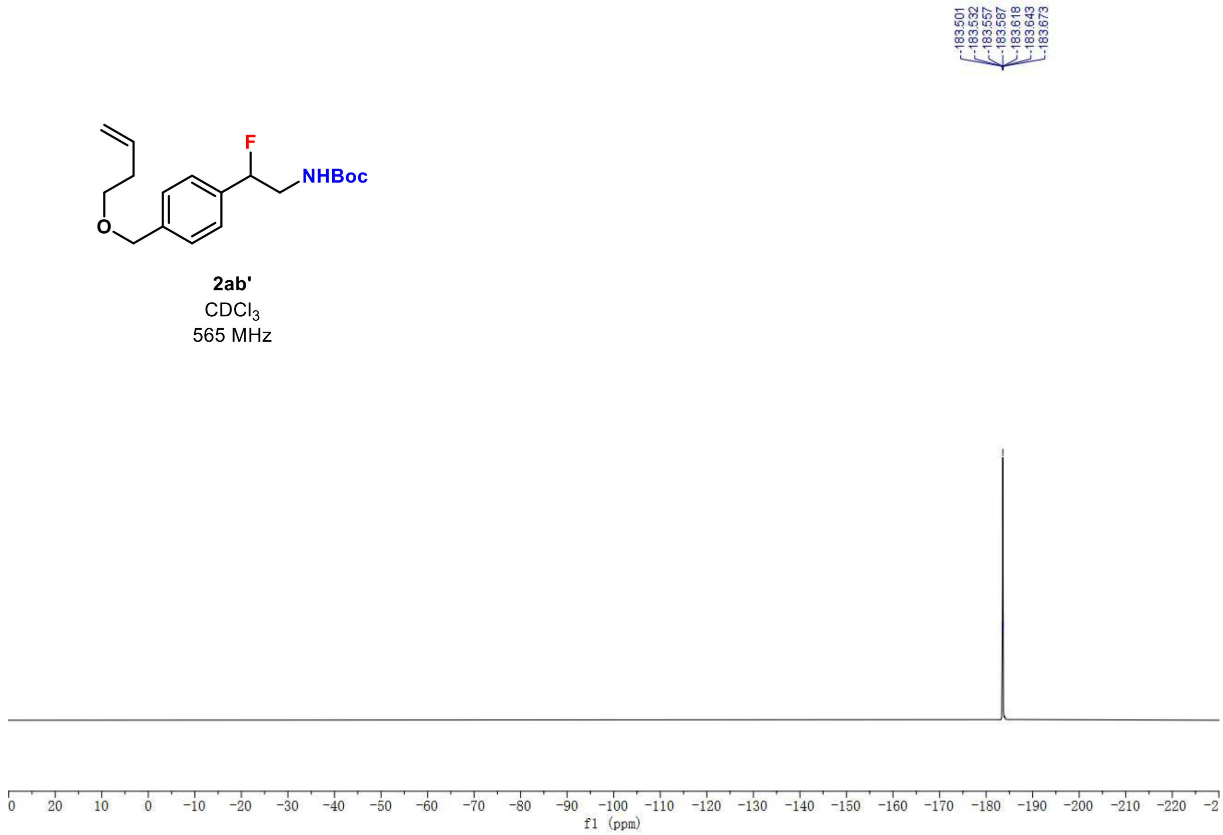

Supplementary Fig. 120. ^{19}**F NMR** Spectra of **2ab'**

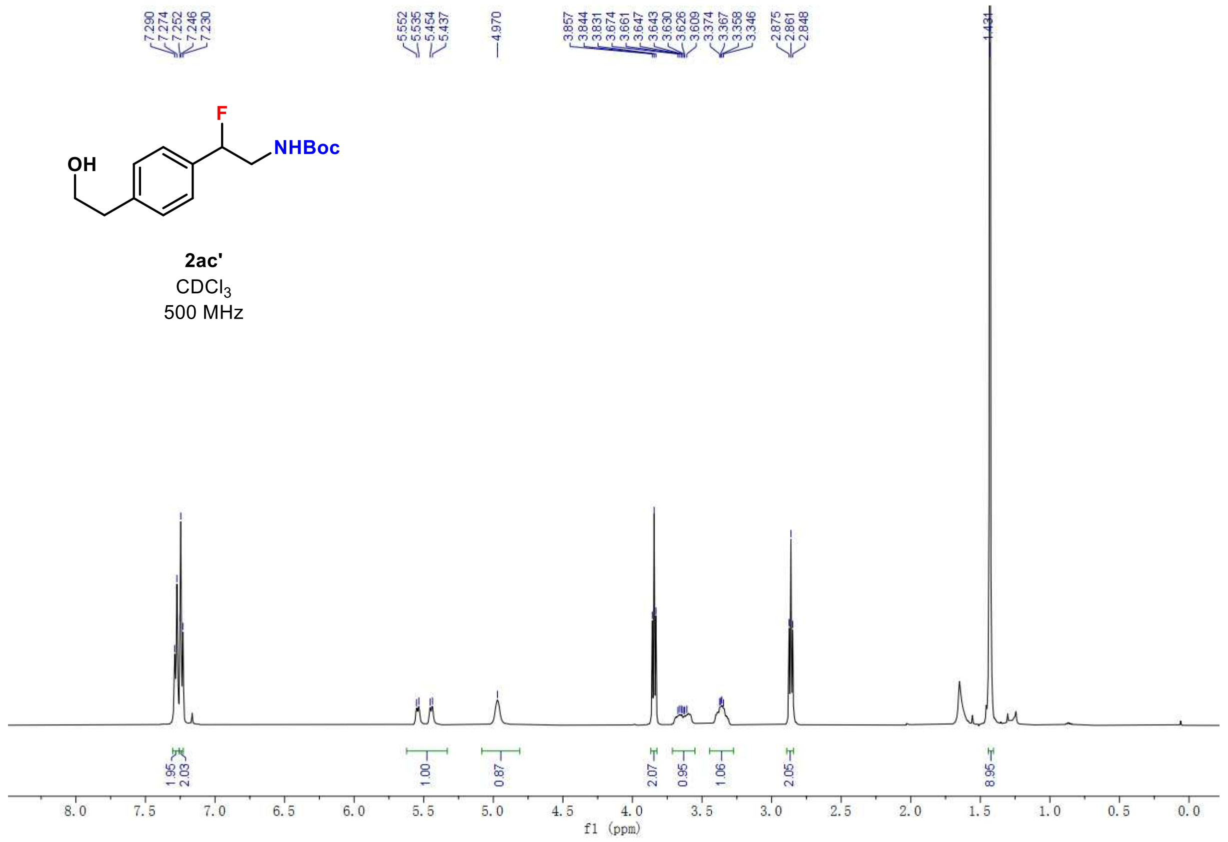

Supplementary Fig. 121. ^{1}**H NMR** Spectra of **2ac'**

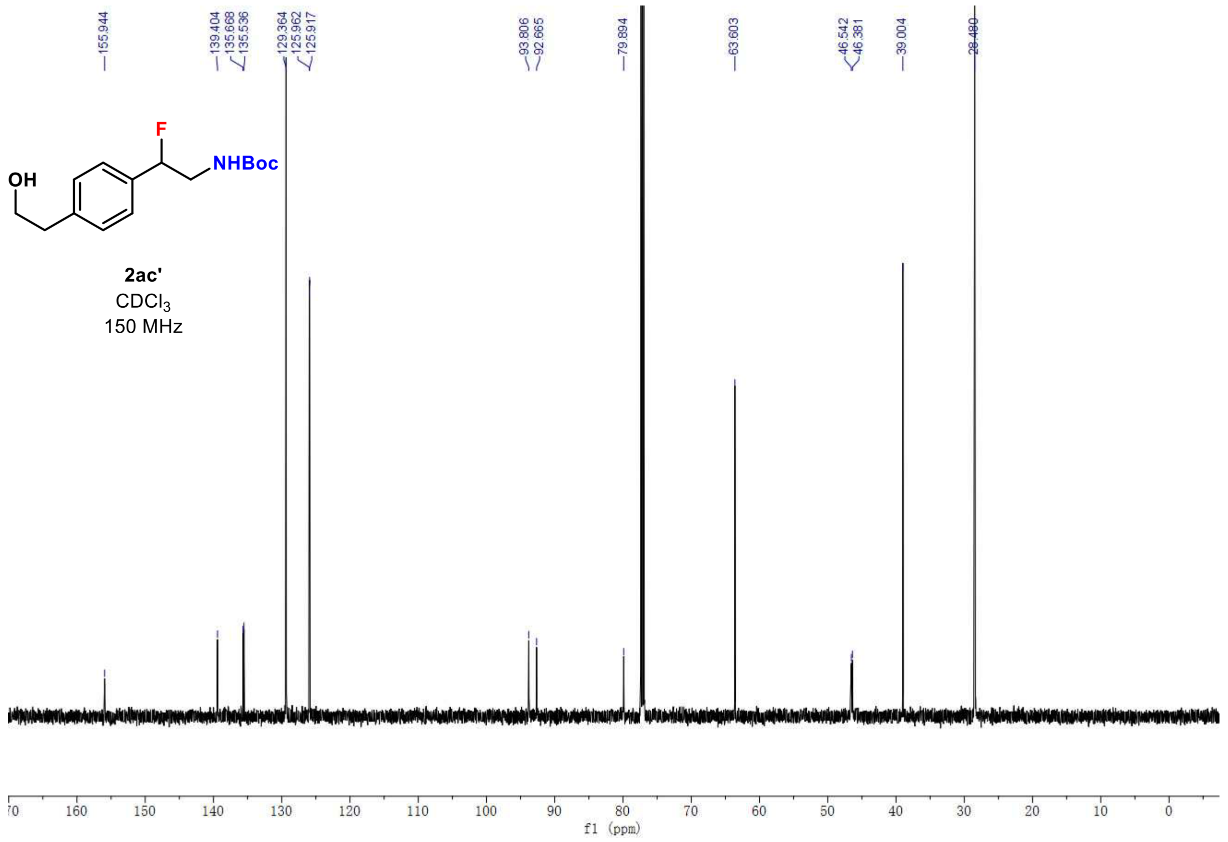

Supplementary Fig. 122. ^{13}**C NMR** Spectra of **2ac'**

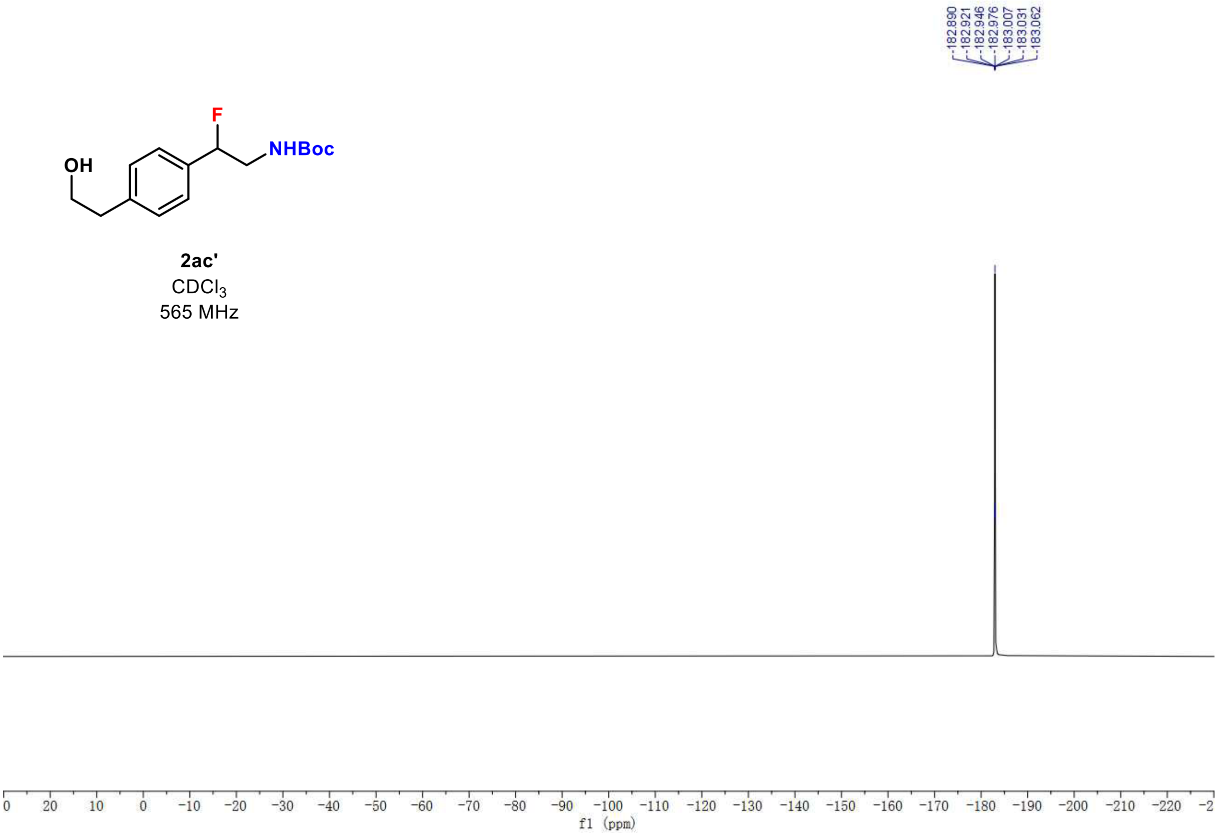

Supplementary Fig. 123. **^{19}F NMR** Spectra of **2ac'**

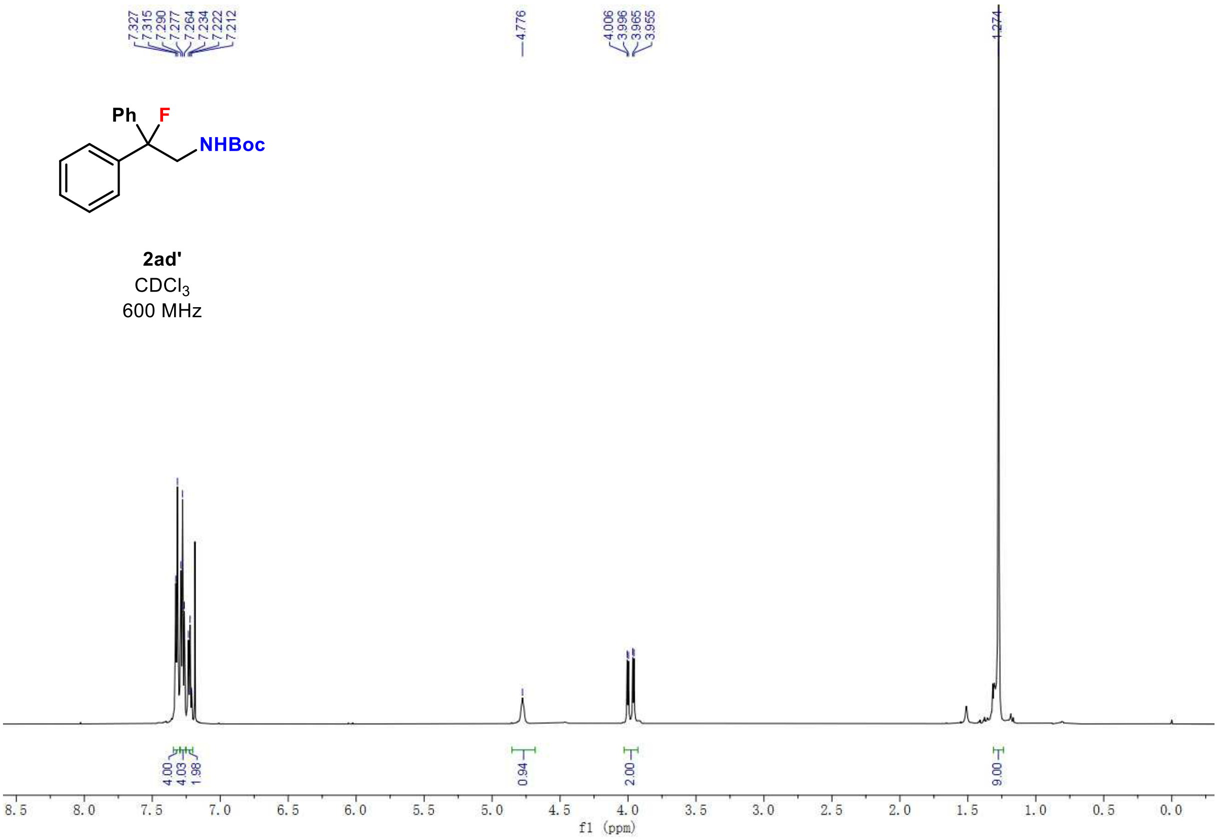

Supplementary Fig. 124. **^{1}H NMR** Spectra of **2ad'**

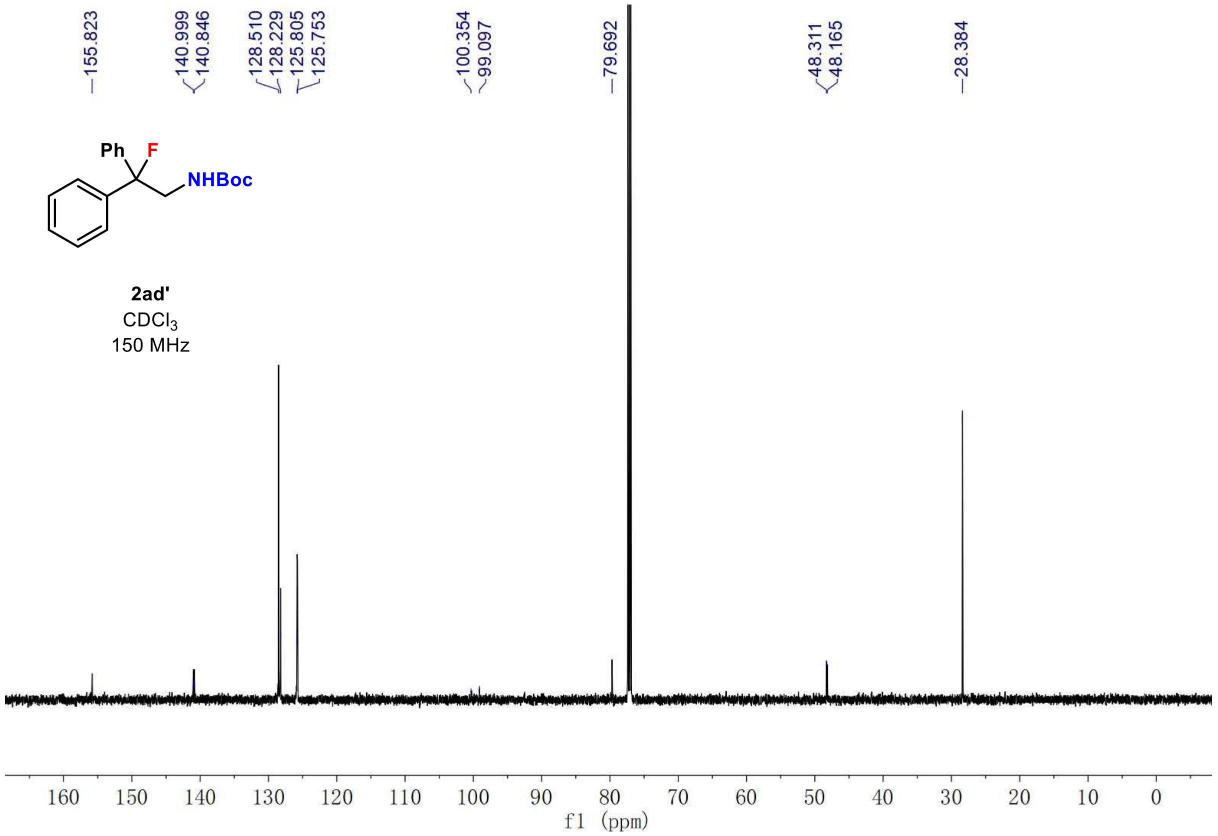

Supplementary Fig. 125. ^{13}**C NMR** Spectra of **2ad'**

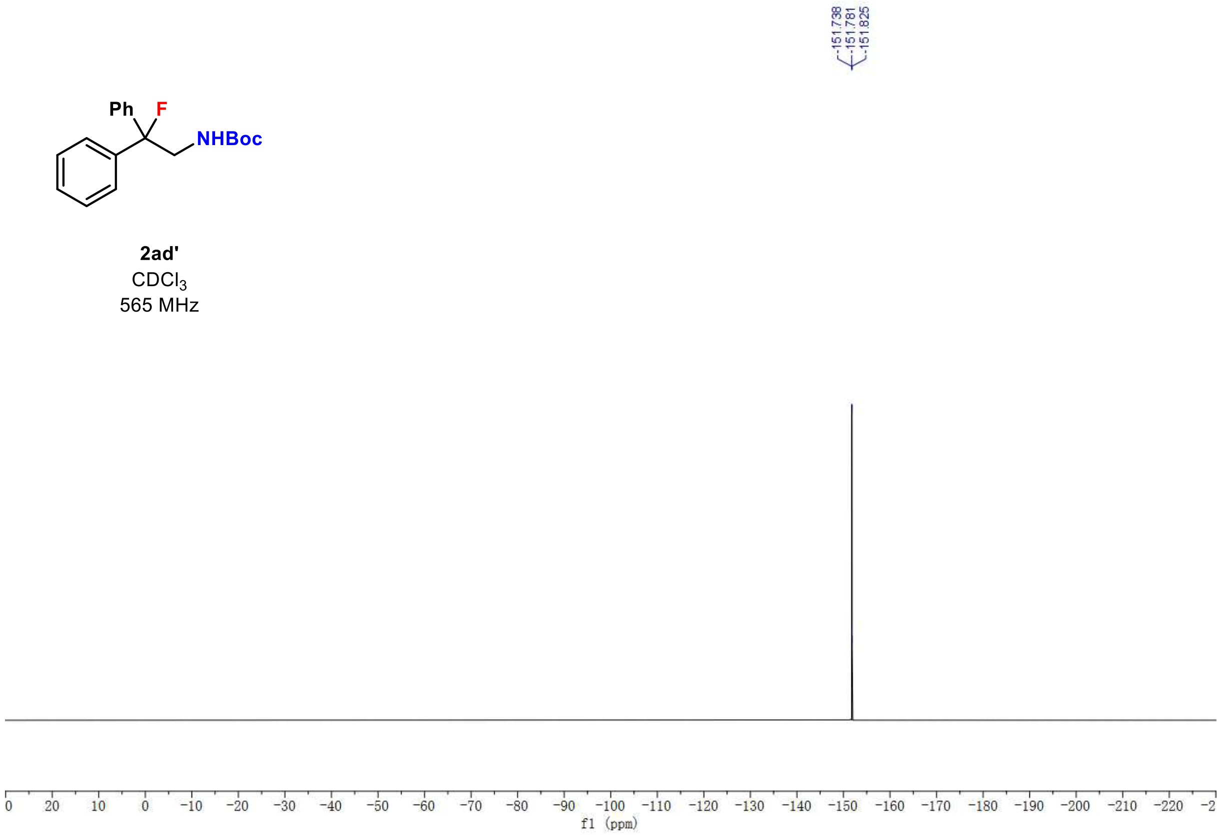

Supplementary Fig. 126. ^{19}**F NMR** Spectra of **2ad'**

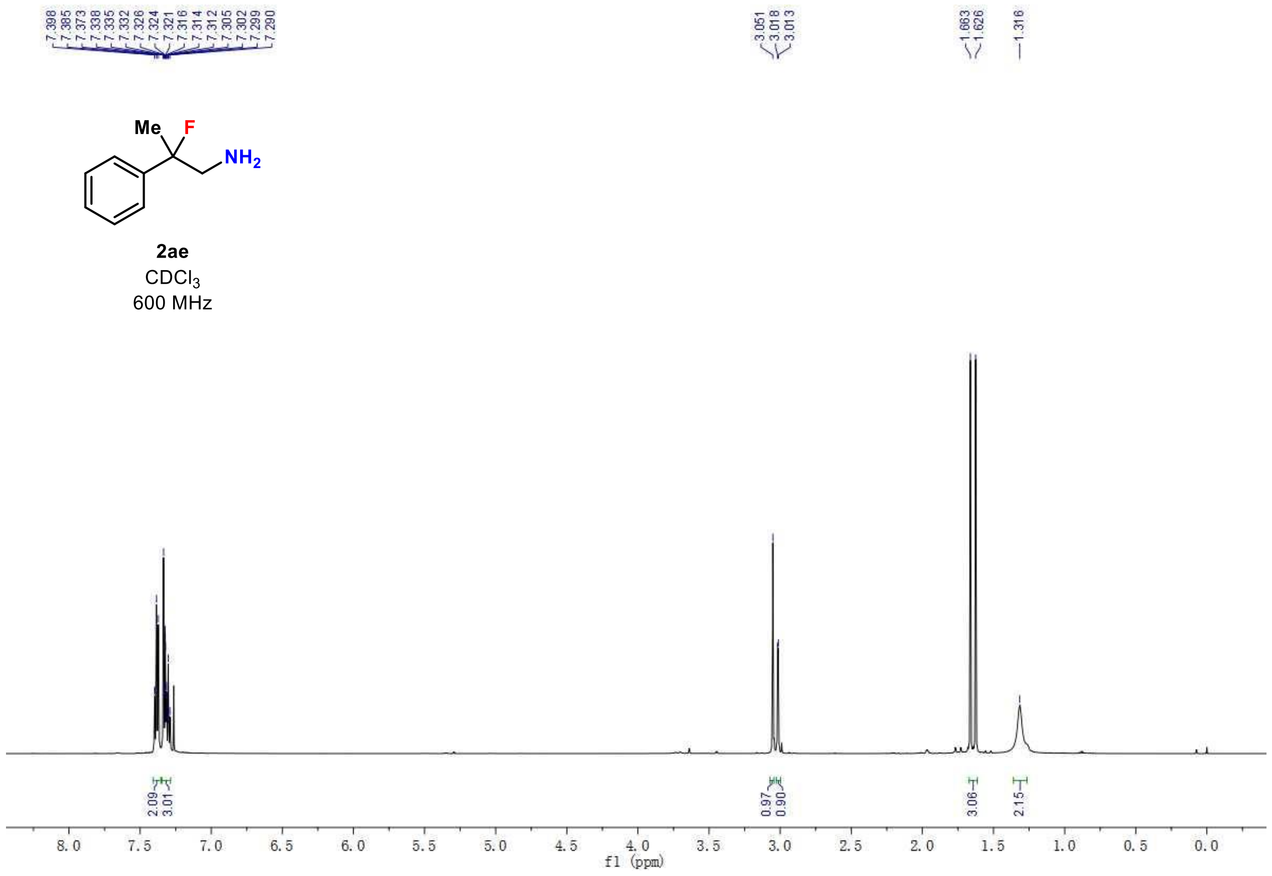

Supplementary Fig. 127. ^{1}H NMR Spectra of **2ae**

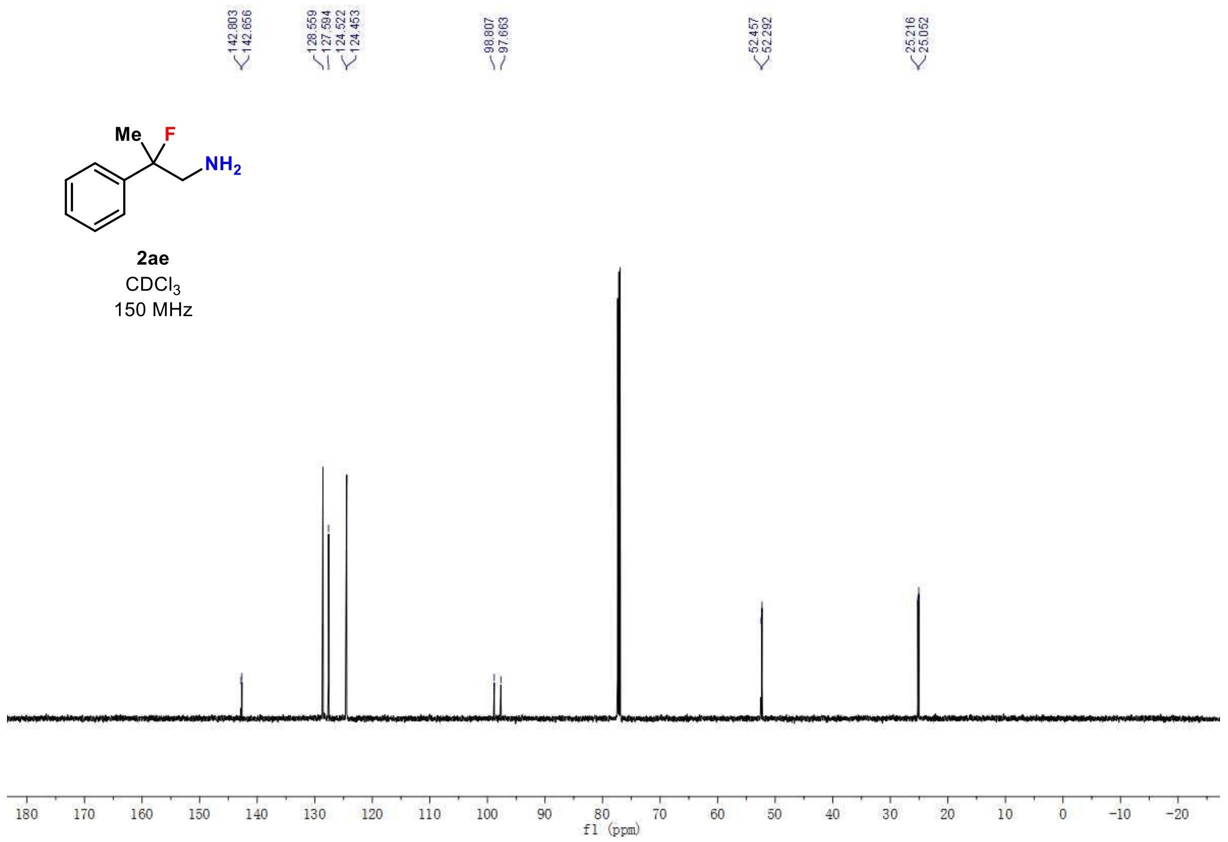

Supplementary Fig. 128. ^{13}C NMR Spectra of **2ae**

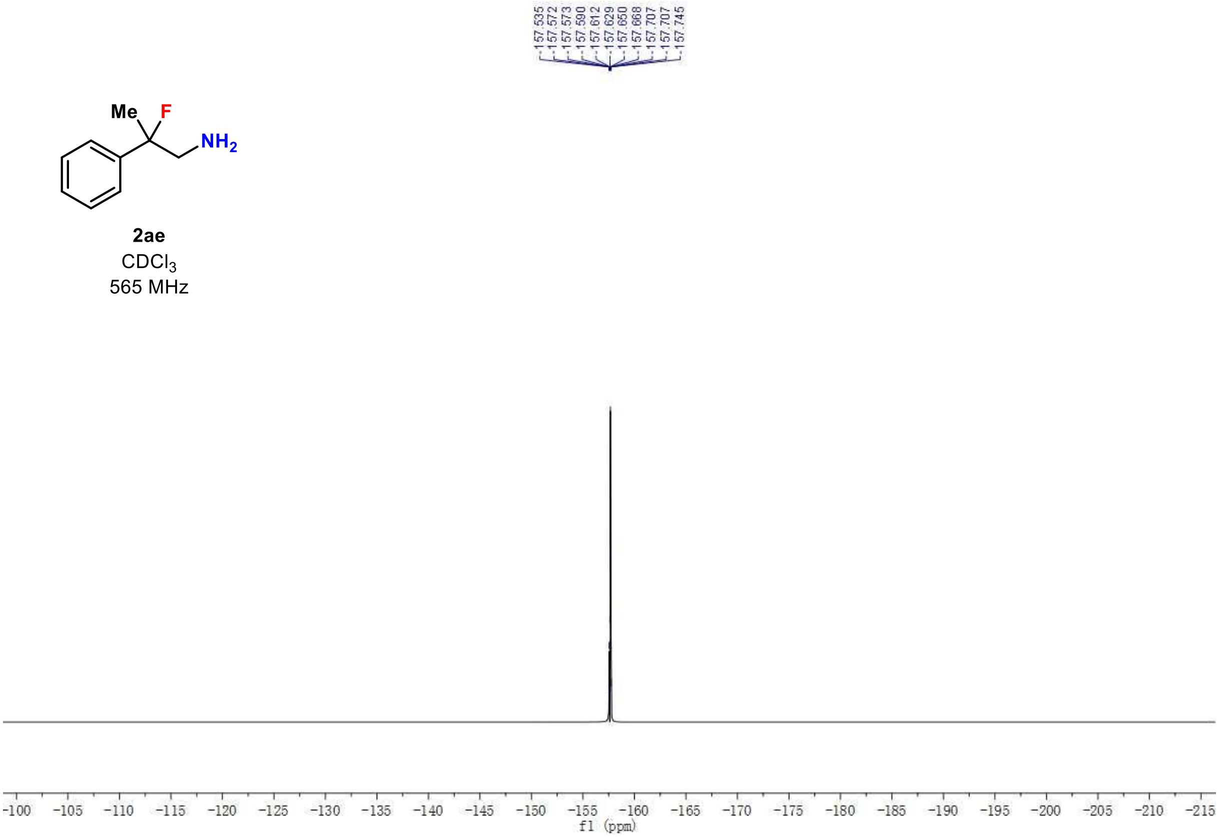

Supplementary Fig. 129. [19]**F NMR** Spectra of **2ae**

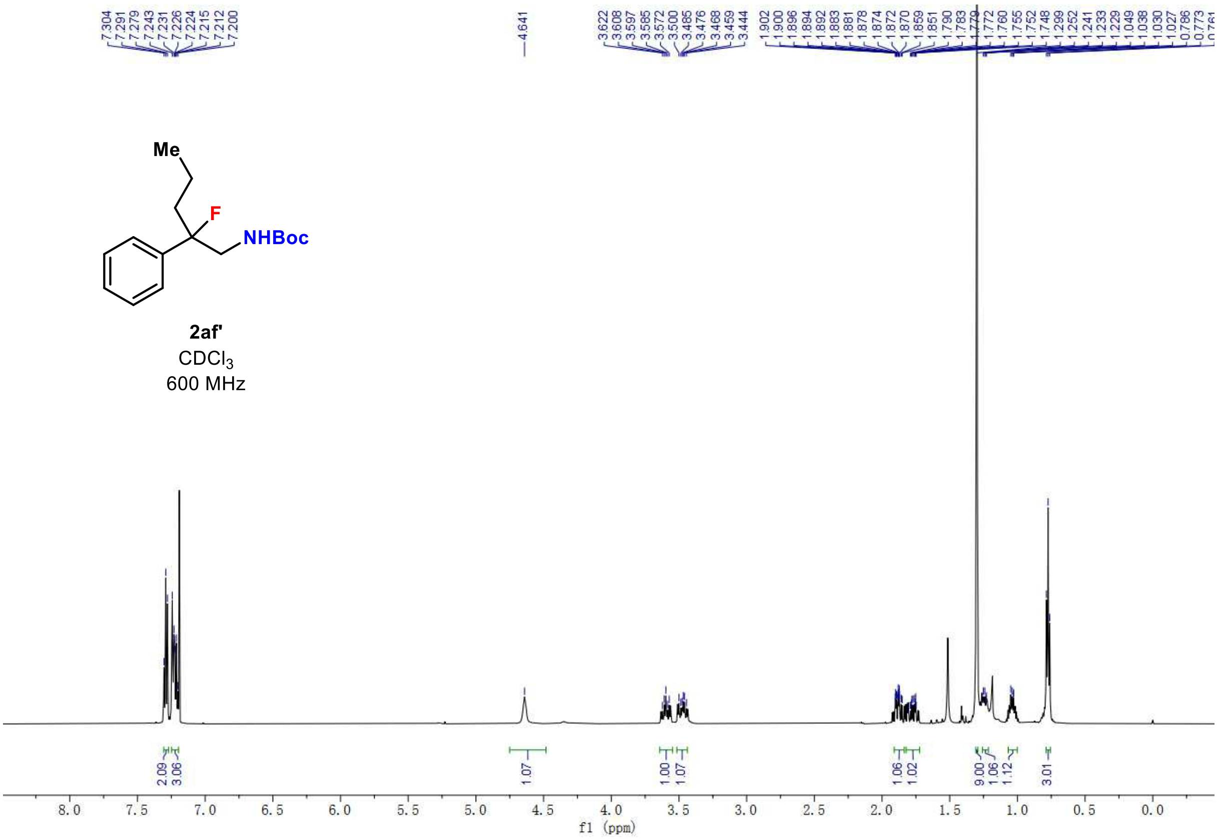

Supplementary Fig. 130. [1]**H NMR** Spectra of **2af'**

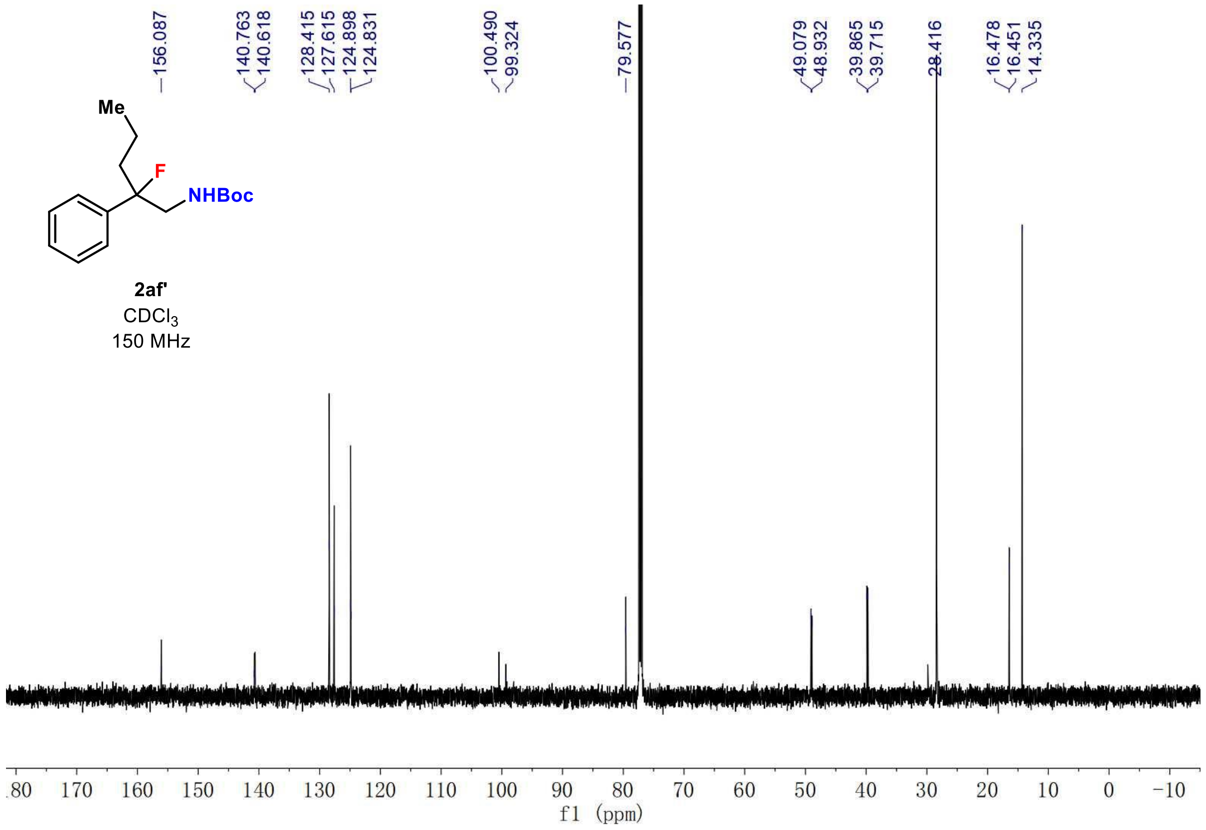

Supplementary Fig. 131. ^{13}**C NMR** Spectra of **2af'**

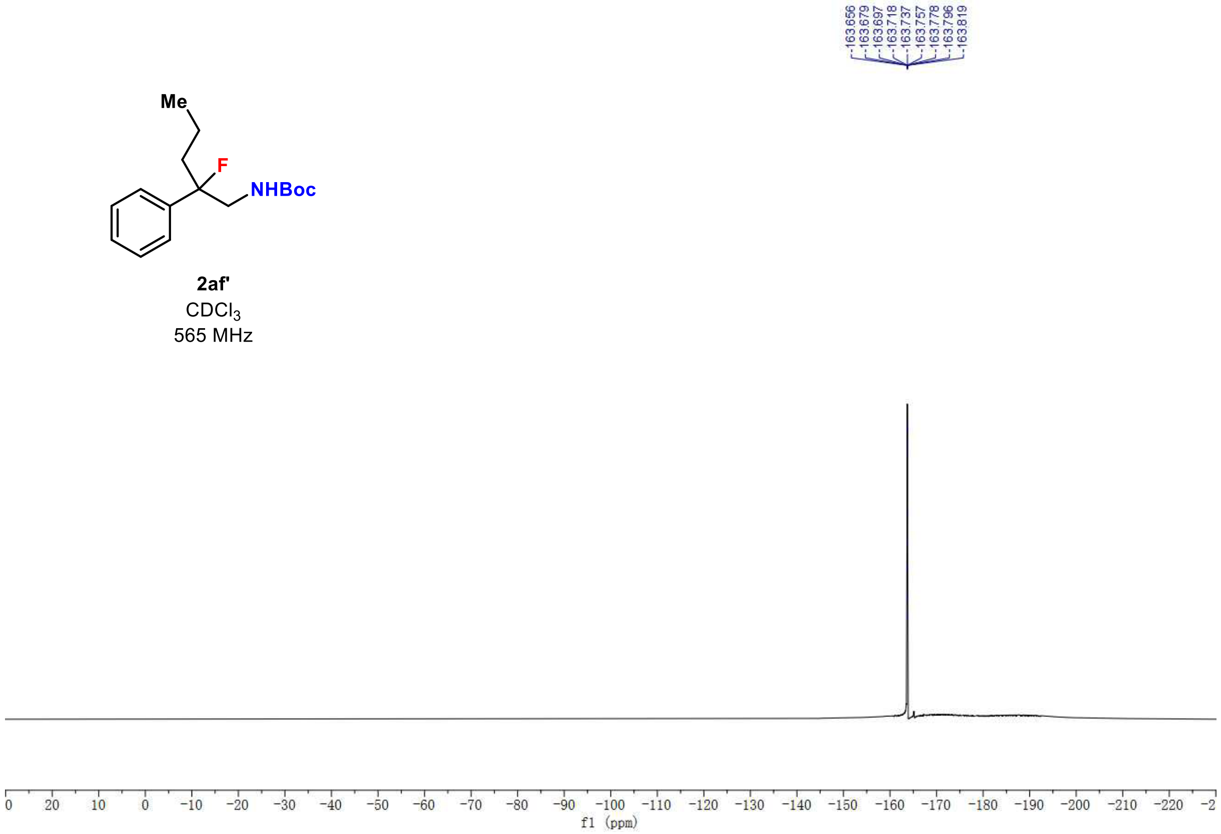

Supplementary Fig. 132. ^{19}**F NMR** Spectra of **2af'**

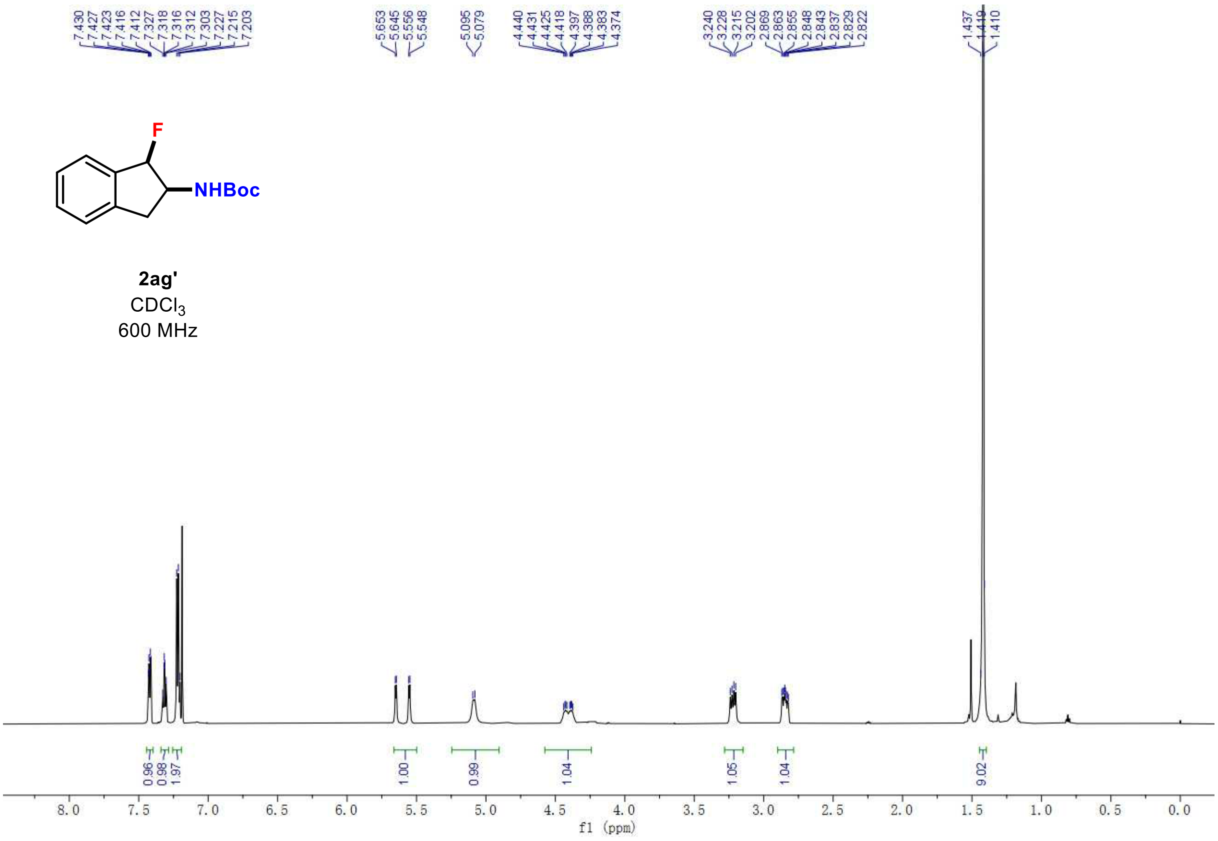

Supplementary Fig. 133. ^{1}**H NMR** Spectra of **2ag'**

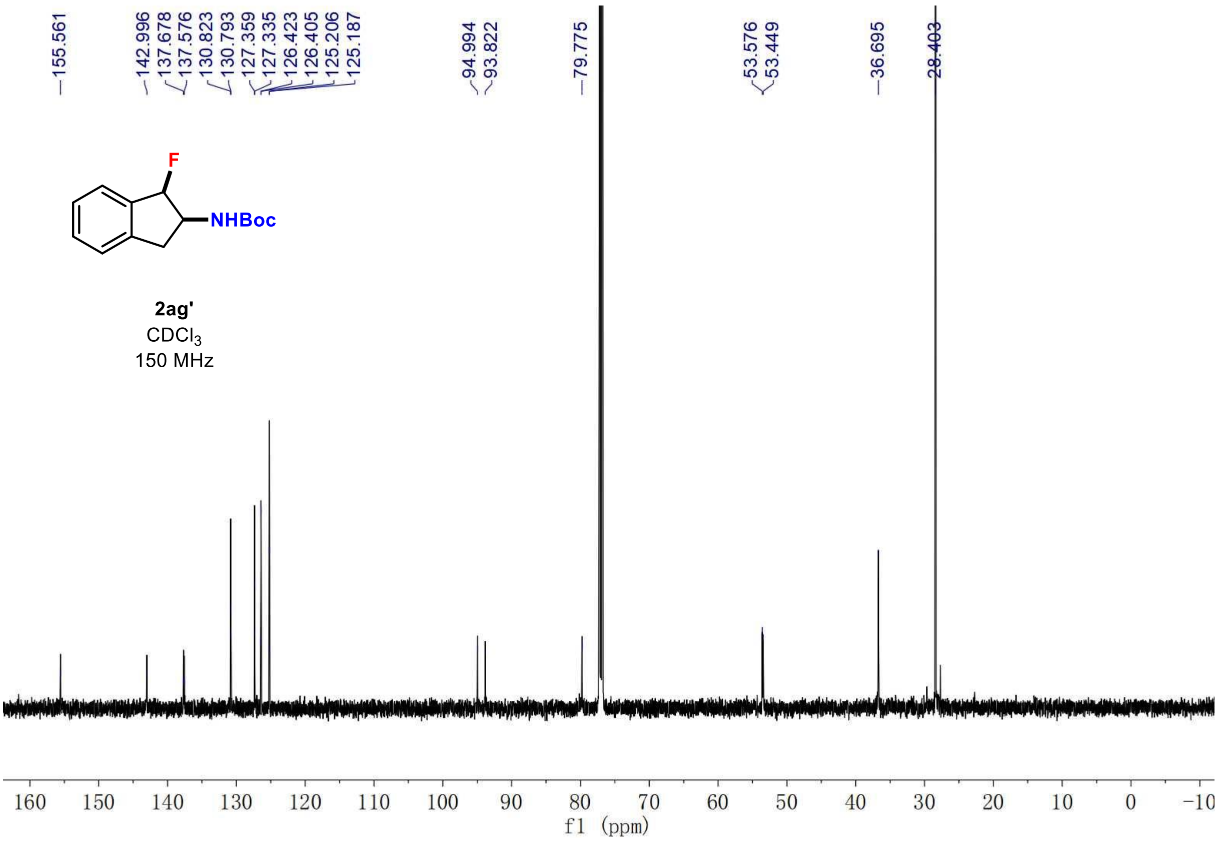

Supplementary Fig. 134. ^{13}**C NMR** Spectra of **2ag'**

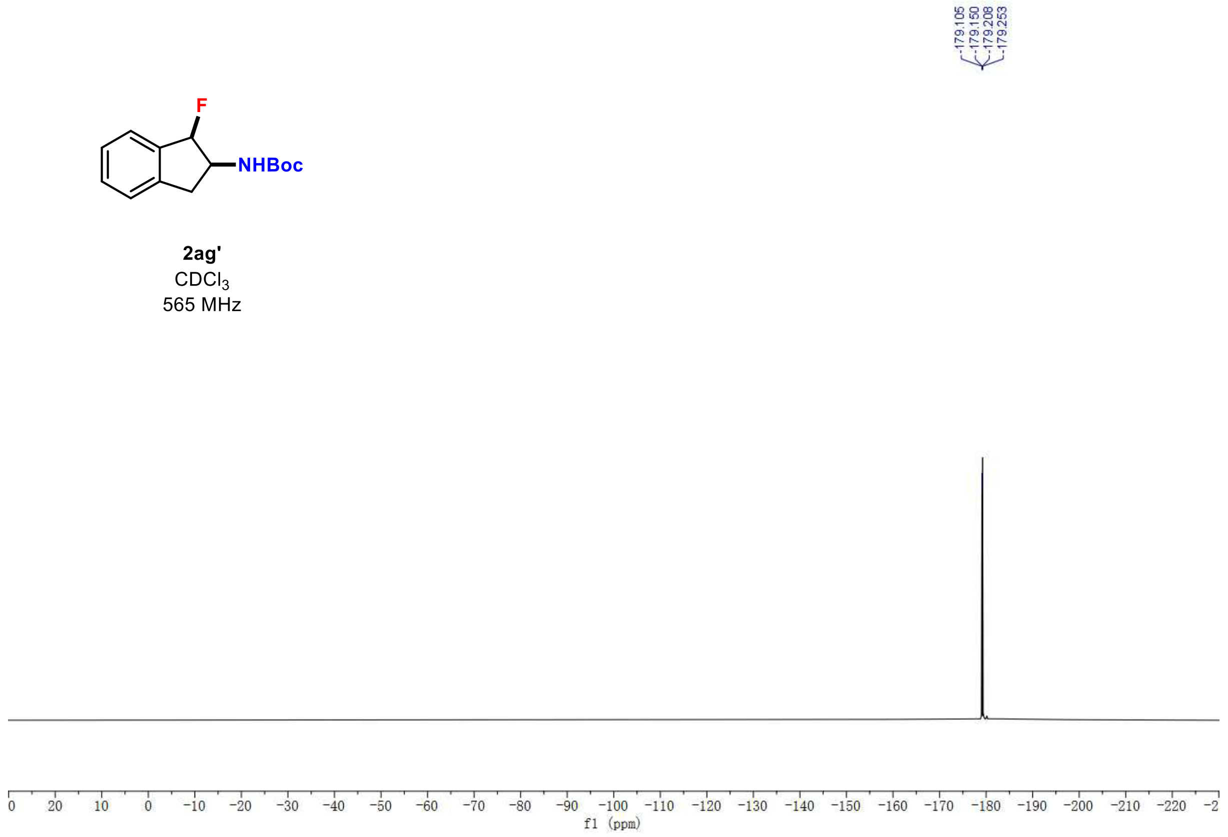

Supplementary Fig. 135. ^{19}**F NMR** Spectra of **2ag'**

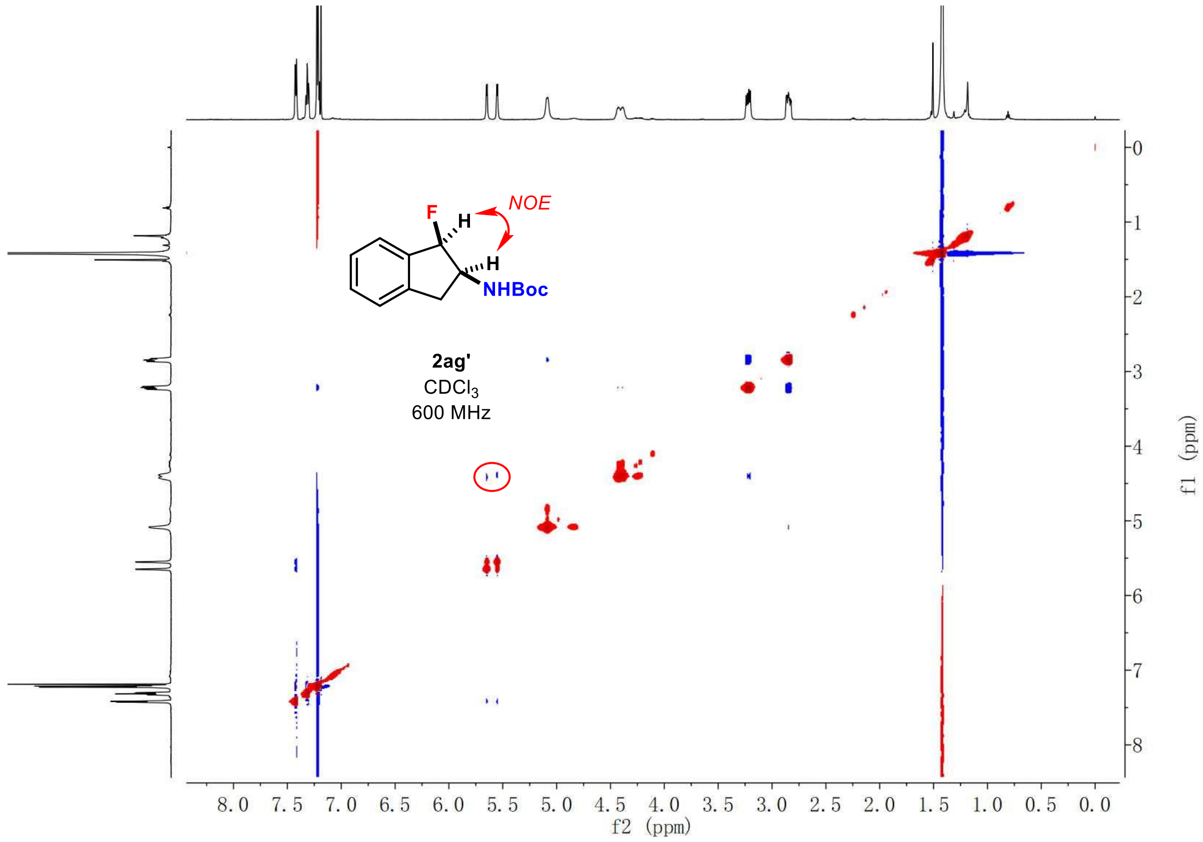

Supplementary Fig. 136. Noesy Spectra of **2ag'**

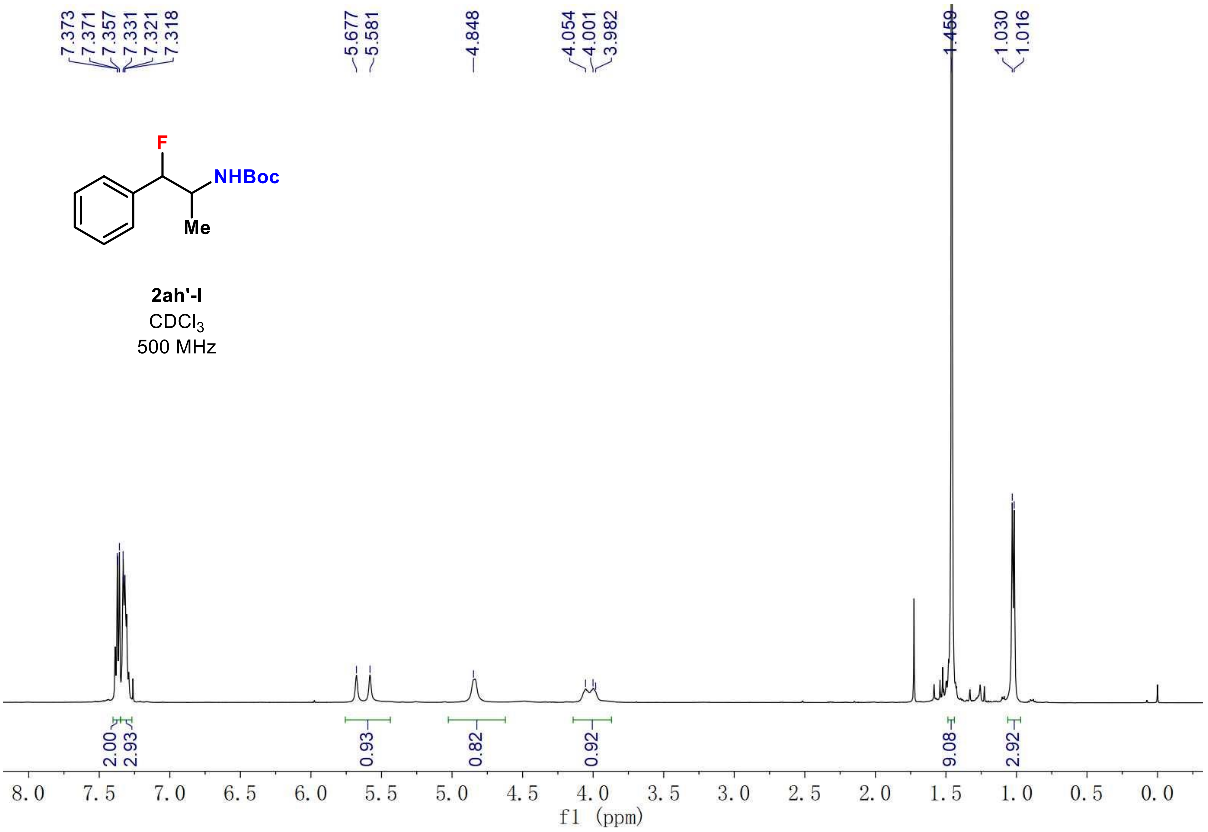

Supplementary Fig. 137. **^{1}H NMR** Spectra of **2ah'-I**

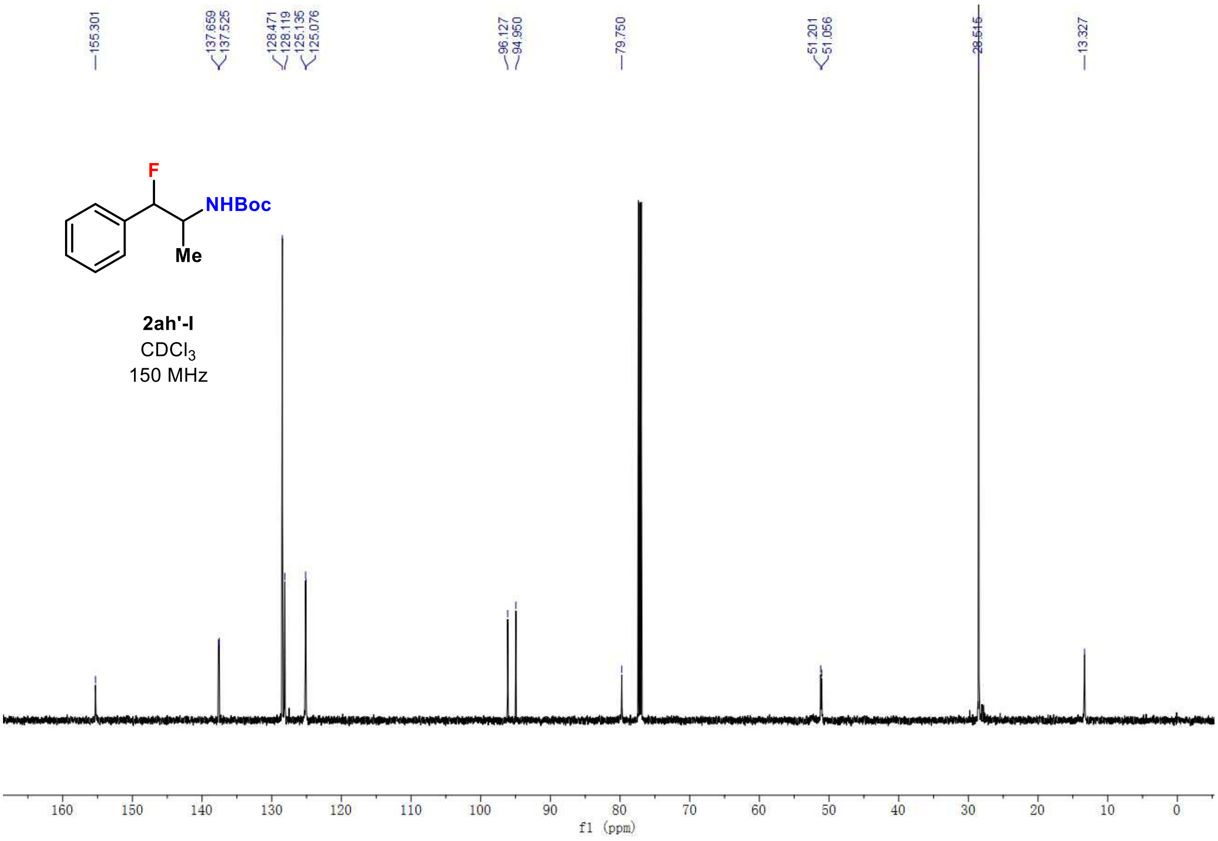

Supplementary Fig. 138. **^{13}C NMR** Spectra of **2ah'-I**

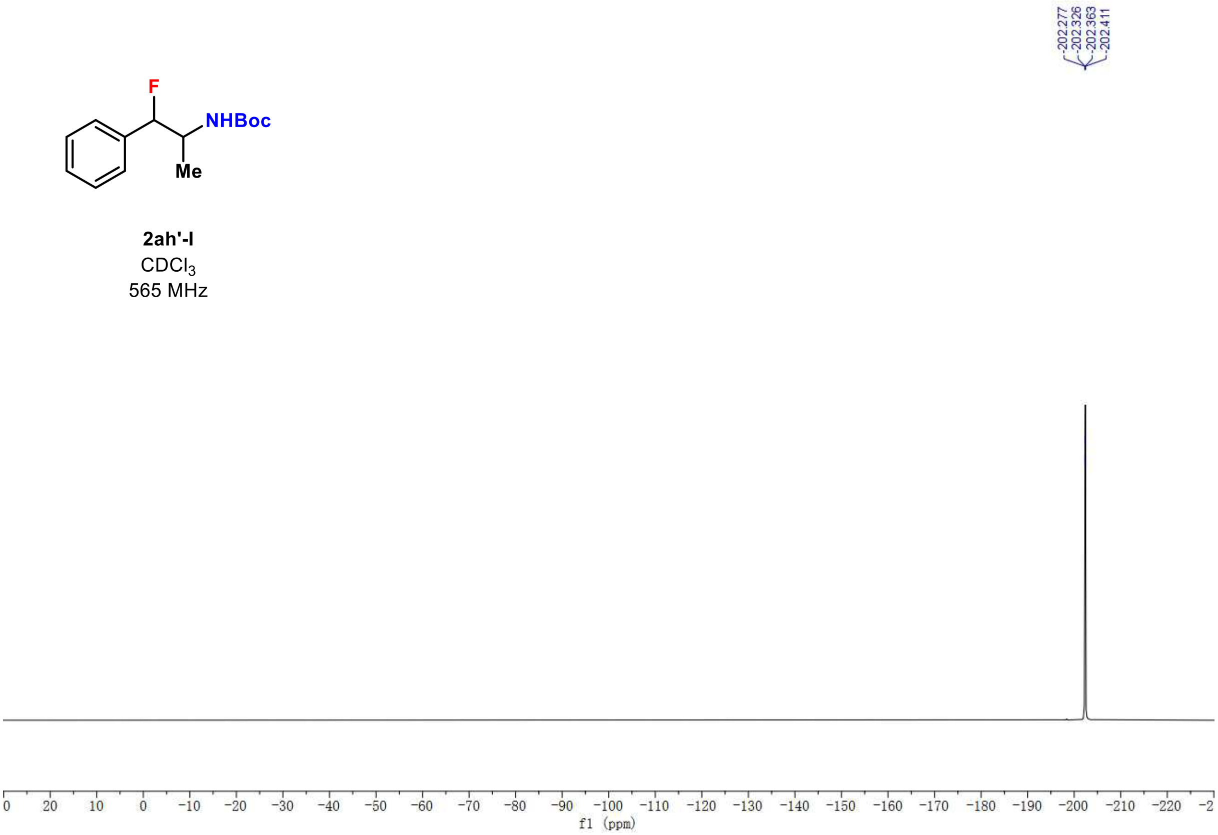

Supplementary Fig. 139. ^{19}**F NMR** Spectra of **2ah'-I**

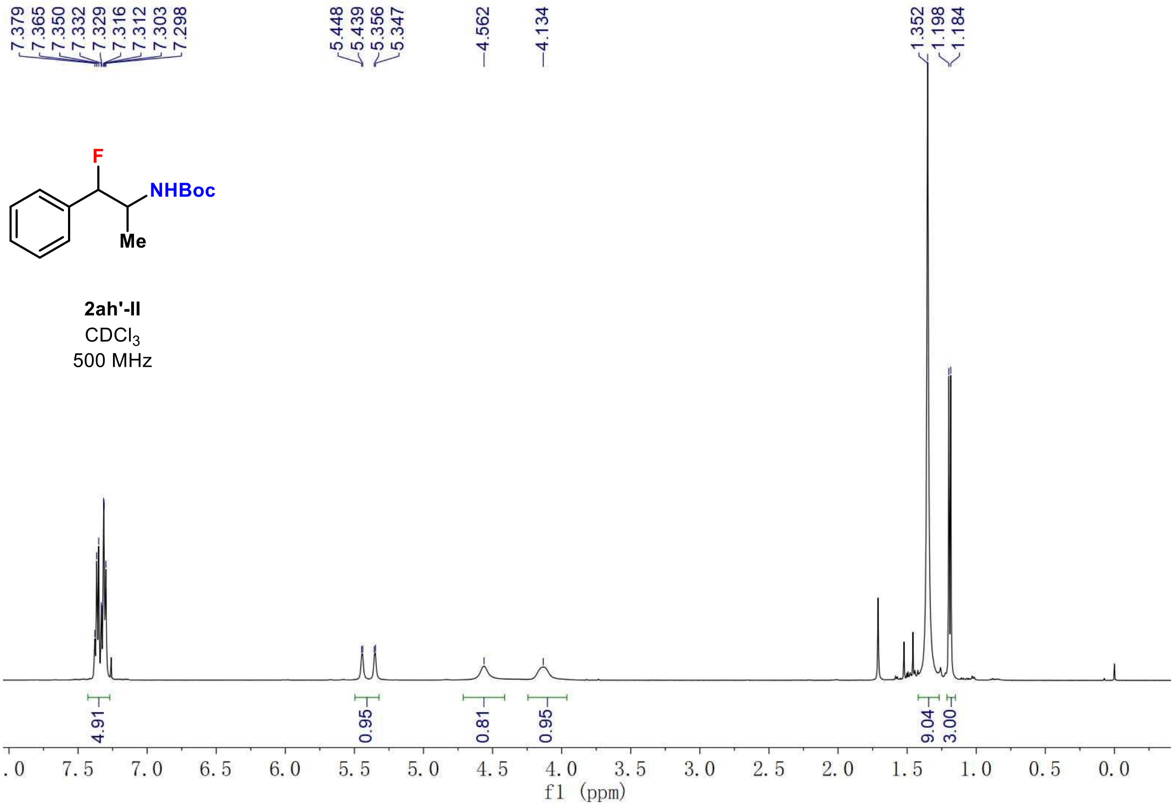

Supplementary Fig. 140. ^{1}**H NMR** Spectra of **2ah'-II**

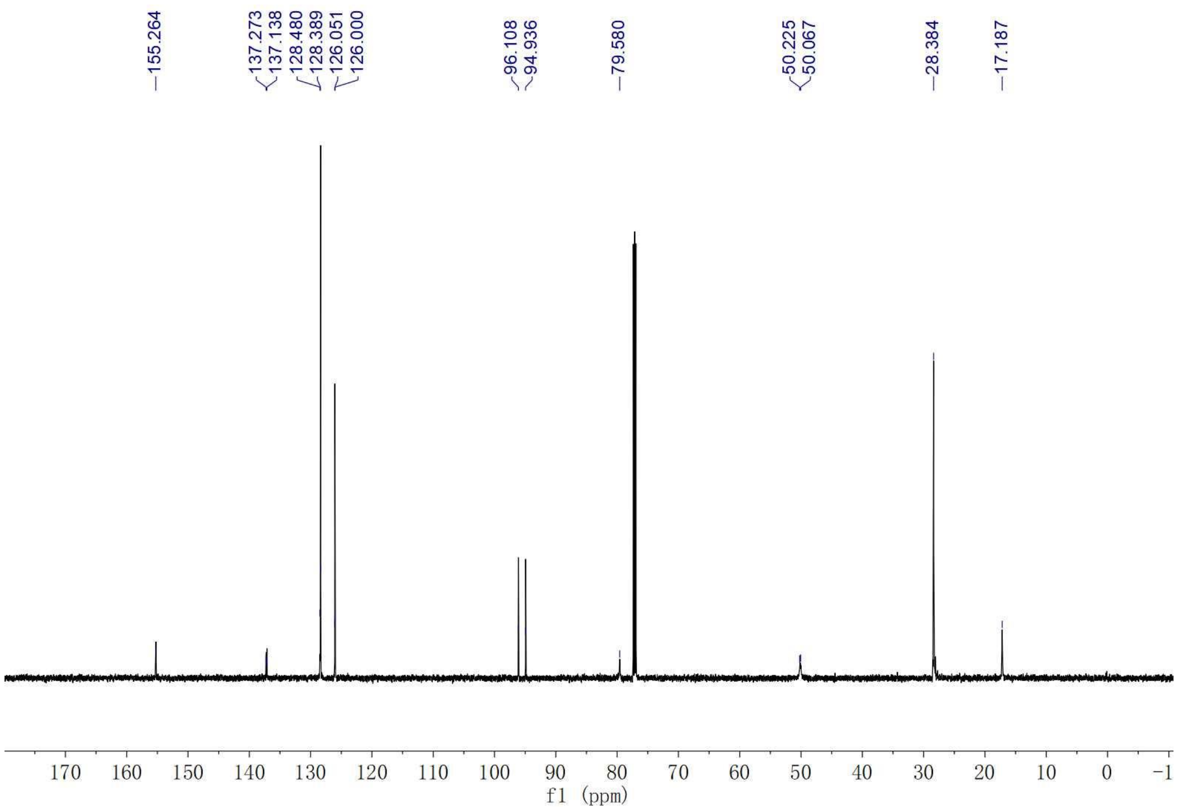

Supplementary Fig. 141. ^{13}**C NMR** Spectra of **2ah'-II**

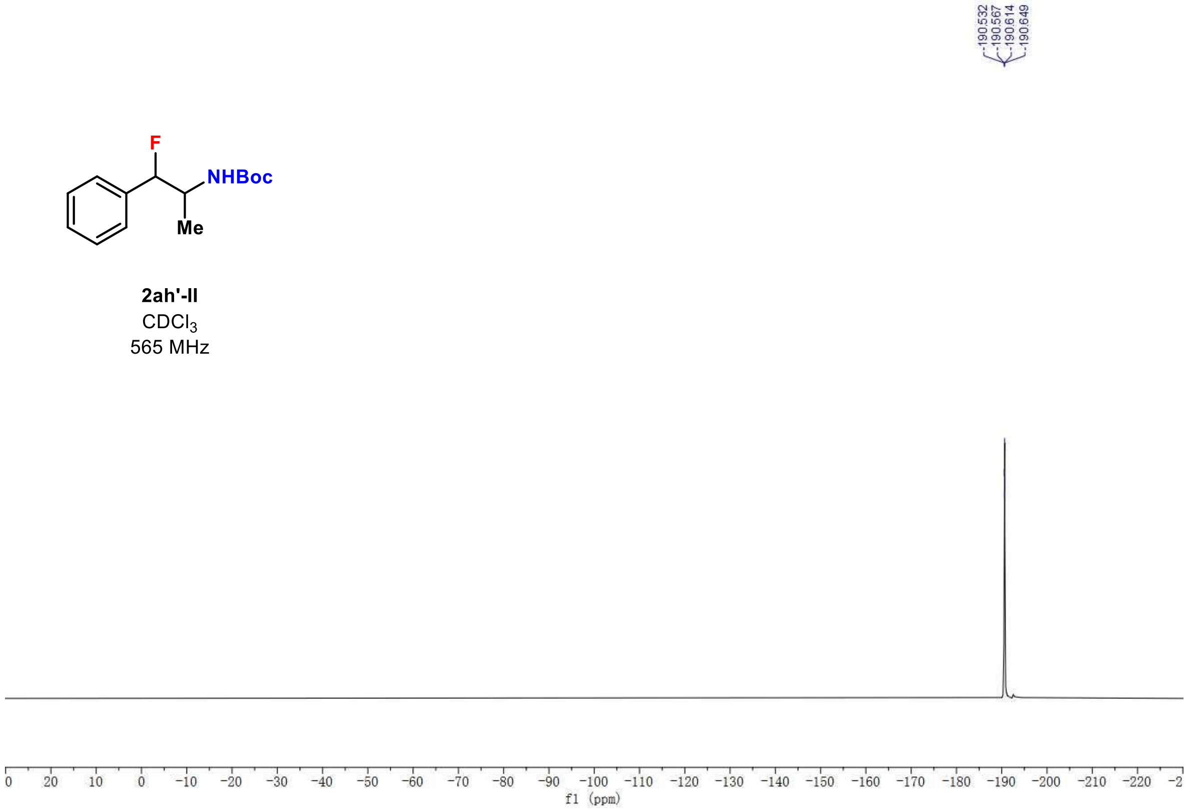

Supplementary Fig. 142. ^{19}**F NMR** Spectra of **2ah'-II**

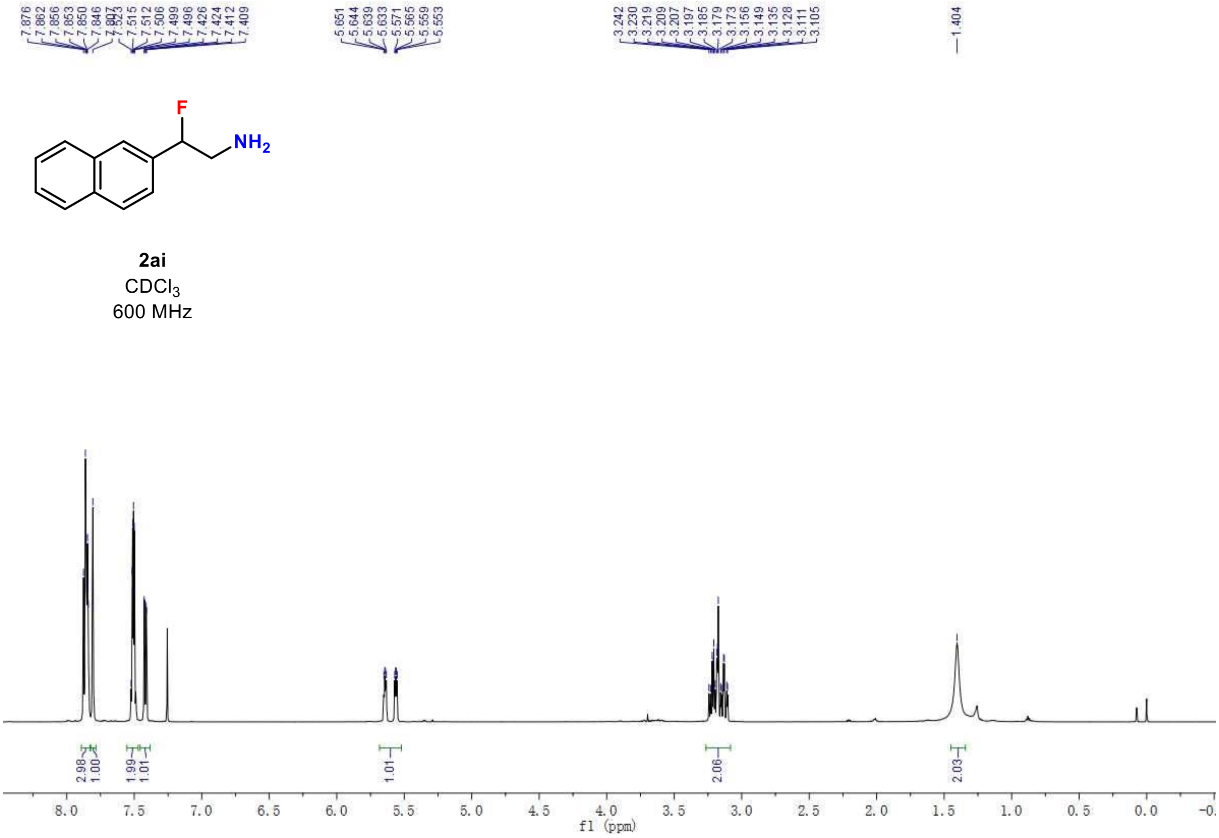

Supplementary Fig. 143. ^{1}H NMR Spectra of **2ai**

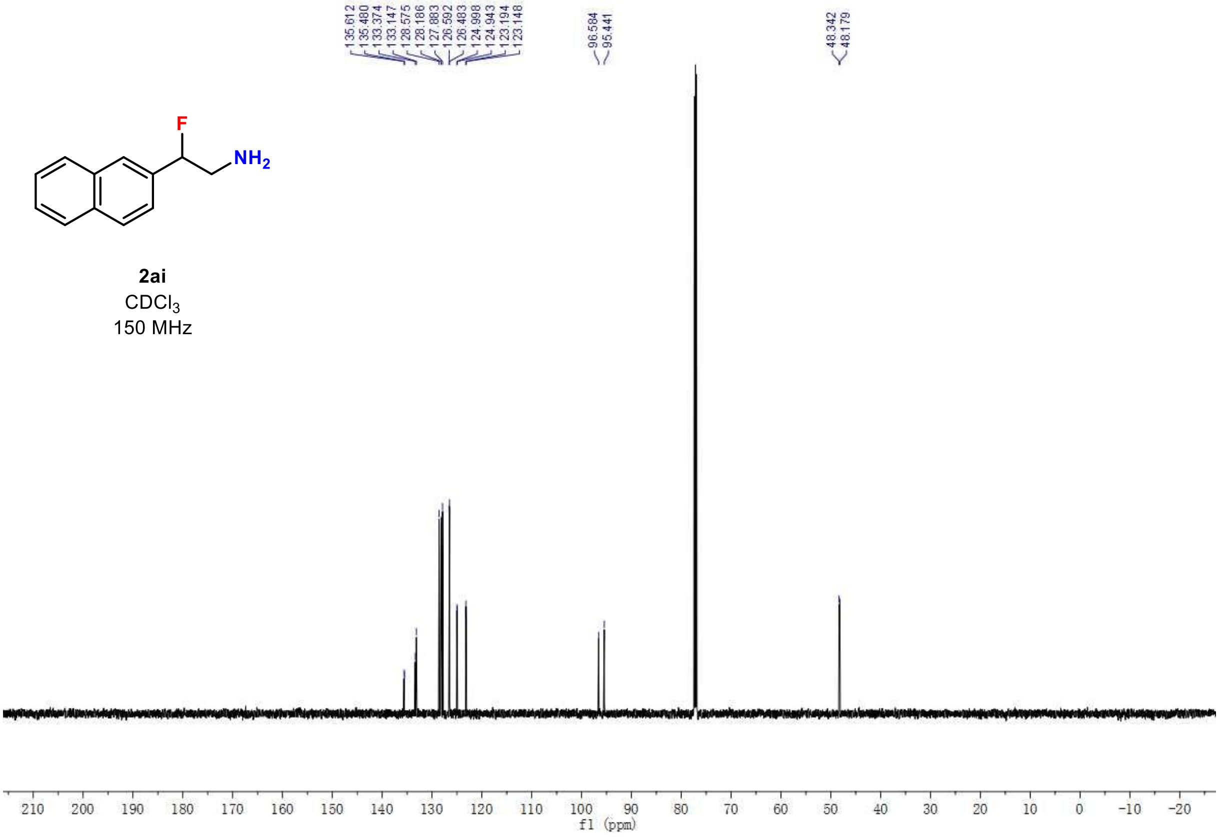

Supplementary Fig. 144. ^{13}C NMR Spectra of **2ai**

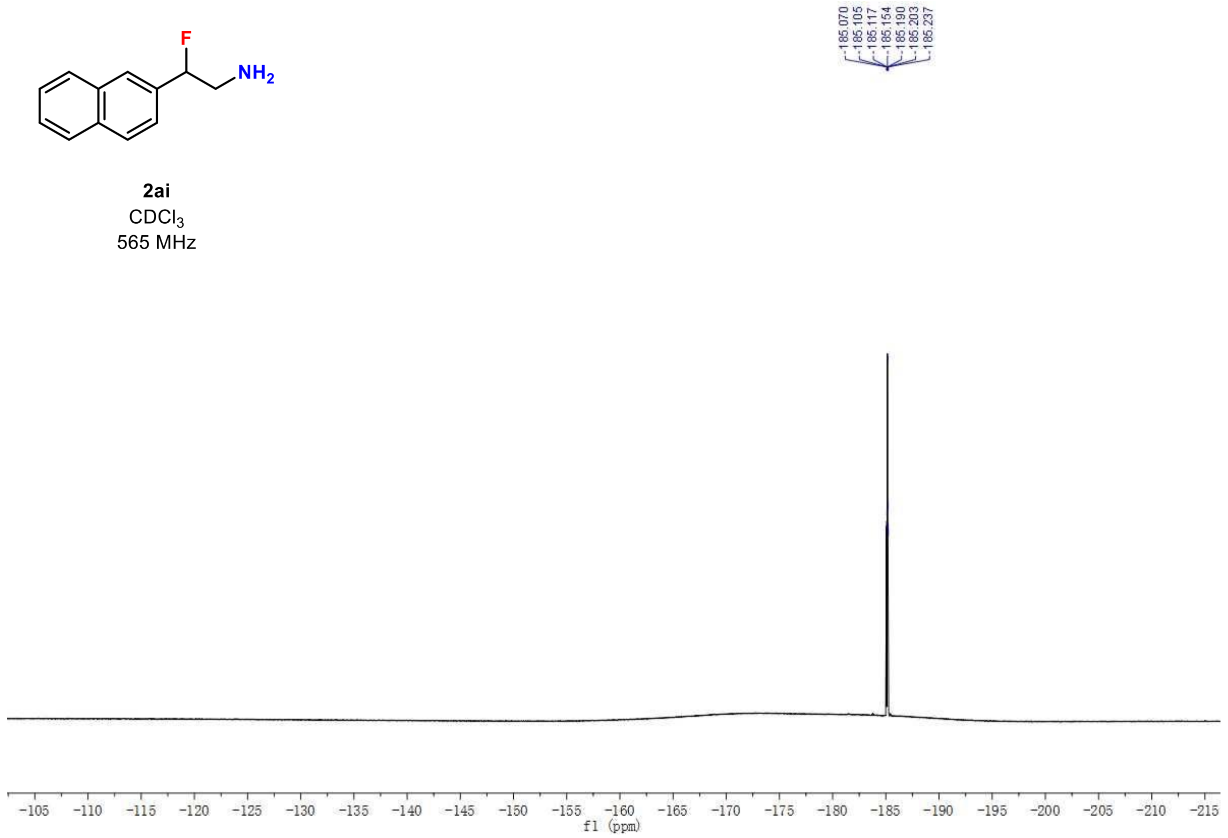

Supplementary Fig. 145. ^{19}F NMR Spectra of **2ai**

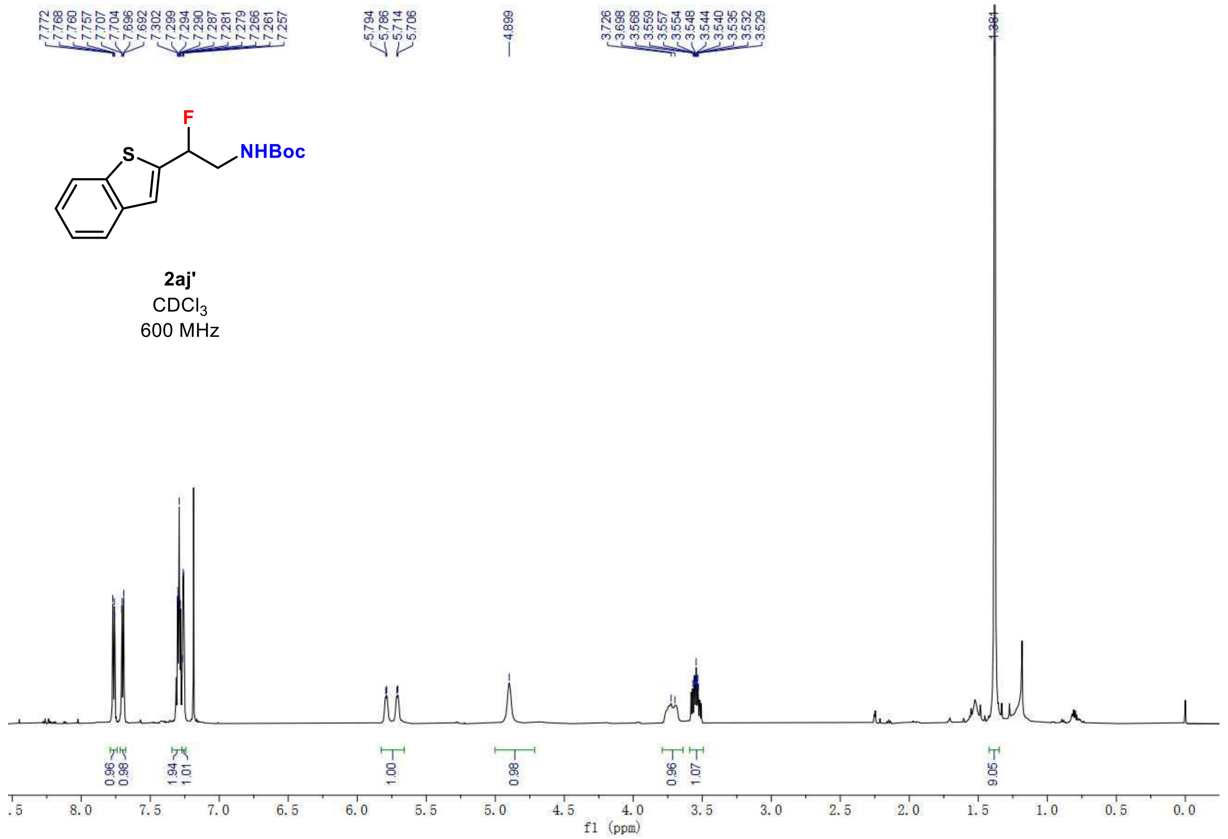

Supplementary Fig. 146. ^{1}H NMR Spectra of **2aj'**

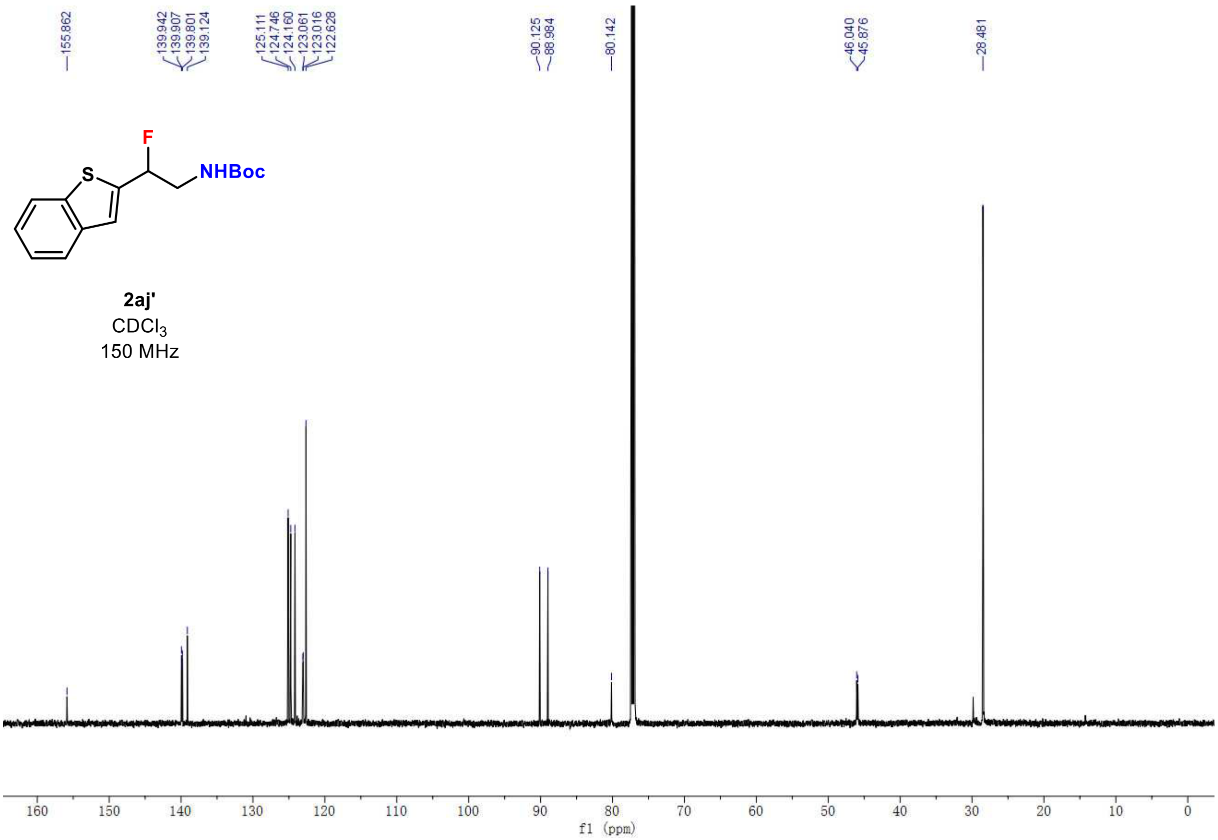

Supplementary Fig. 147. **^{13}C NMR** Spectra of **2aj'**

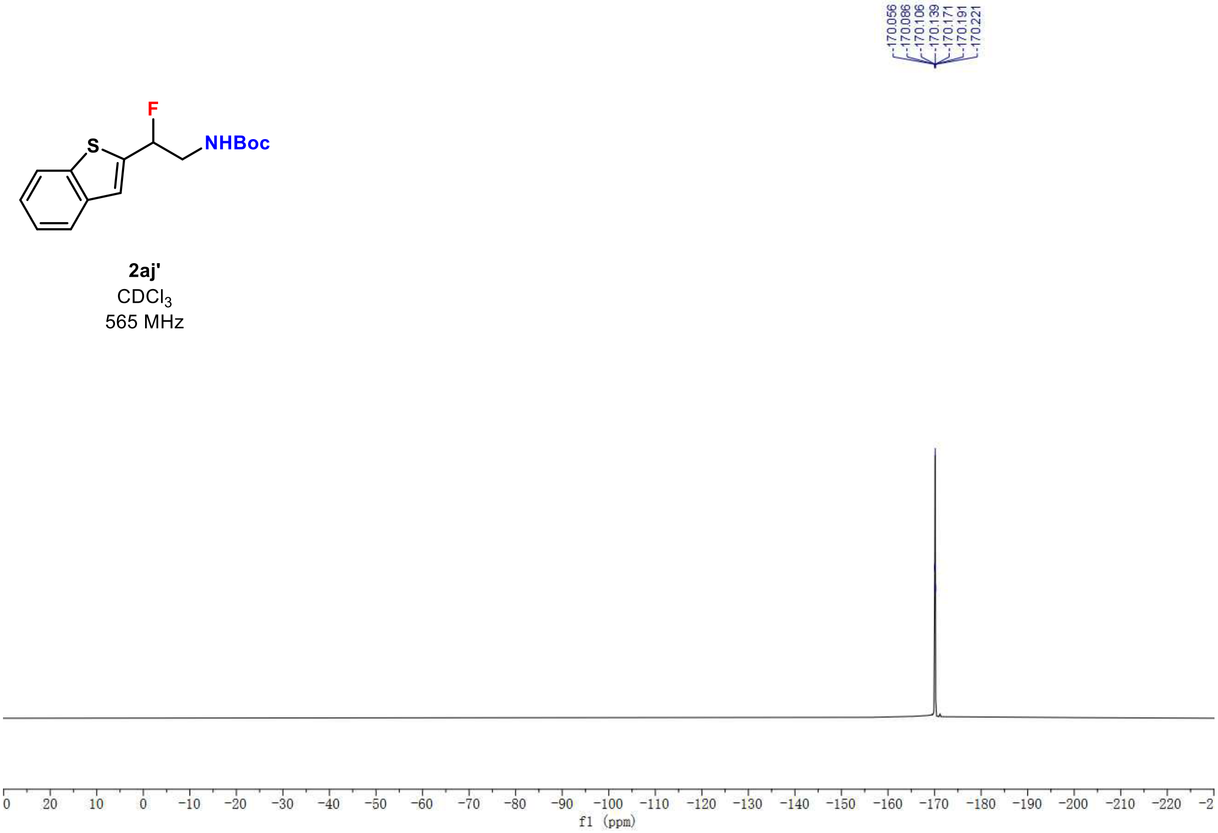

Supplementary Fig. 148. **^{19}F NMR** Spectra of **2aj'**

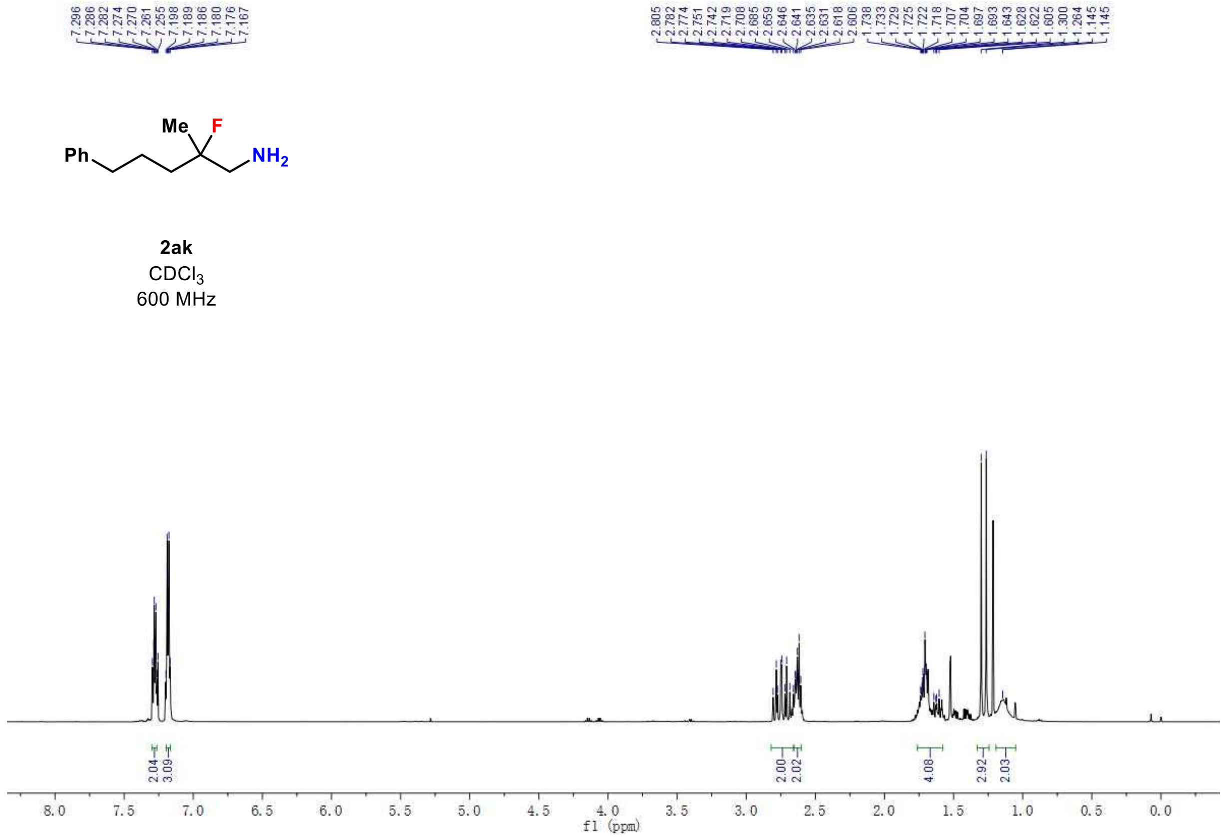

Supplementary Fig. 149. ^{1}H NMR Spectra of **2ak**

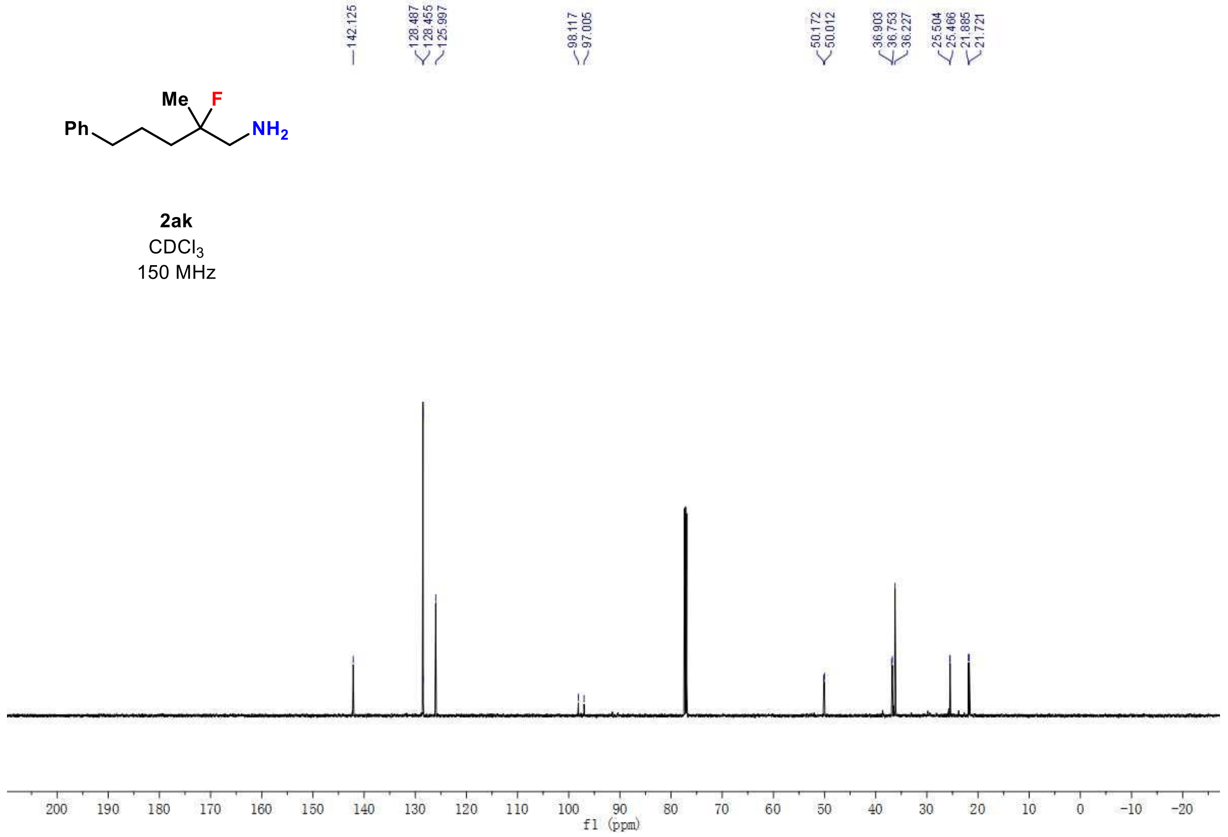

Supplementary Fig. 150. ^{13}C NMR Spectra of **2ak**

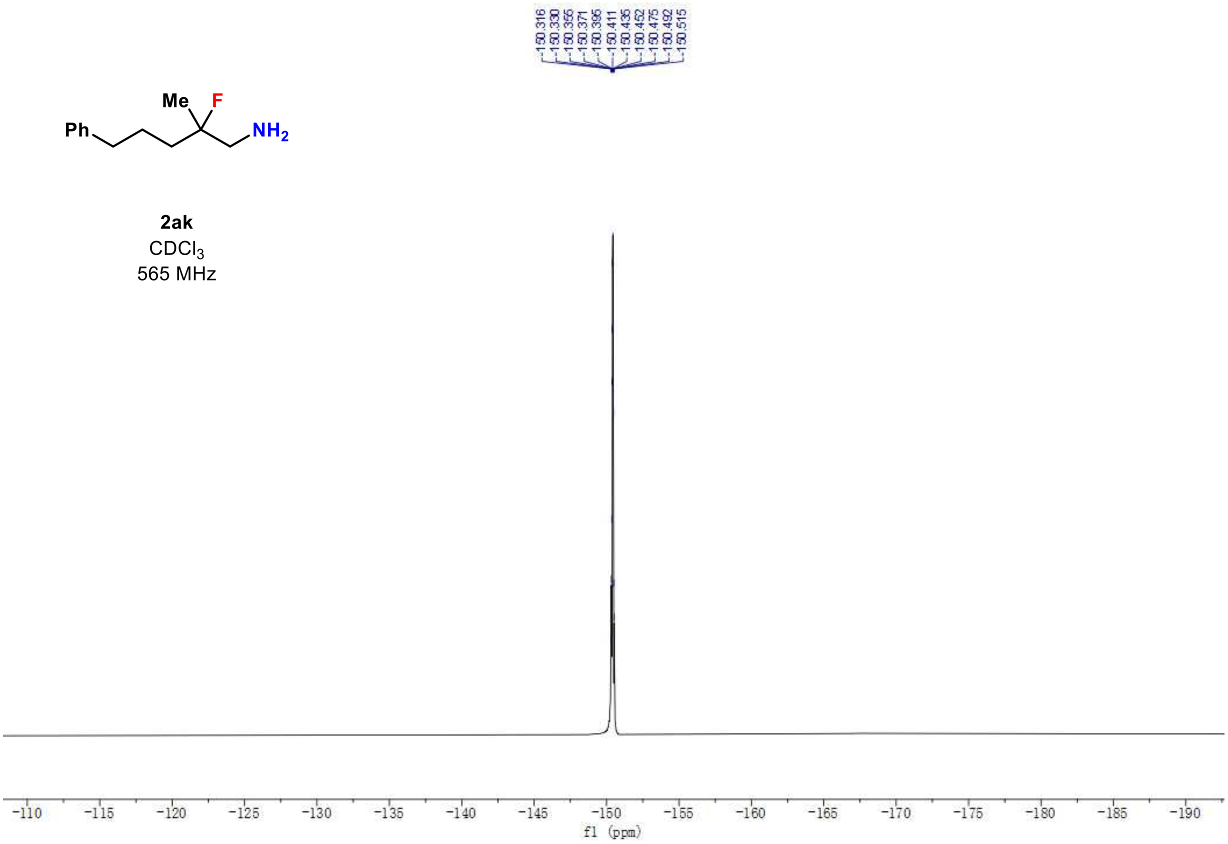

Supplementary Fig. 151. **^{19}F NMR** Spectra of **2ak**

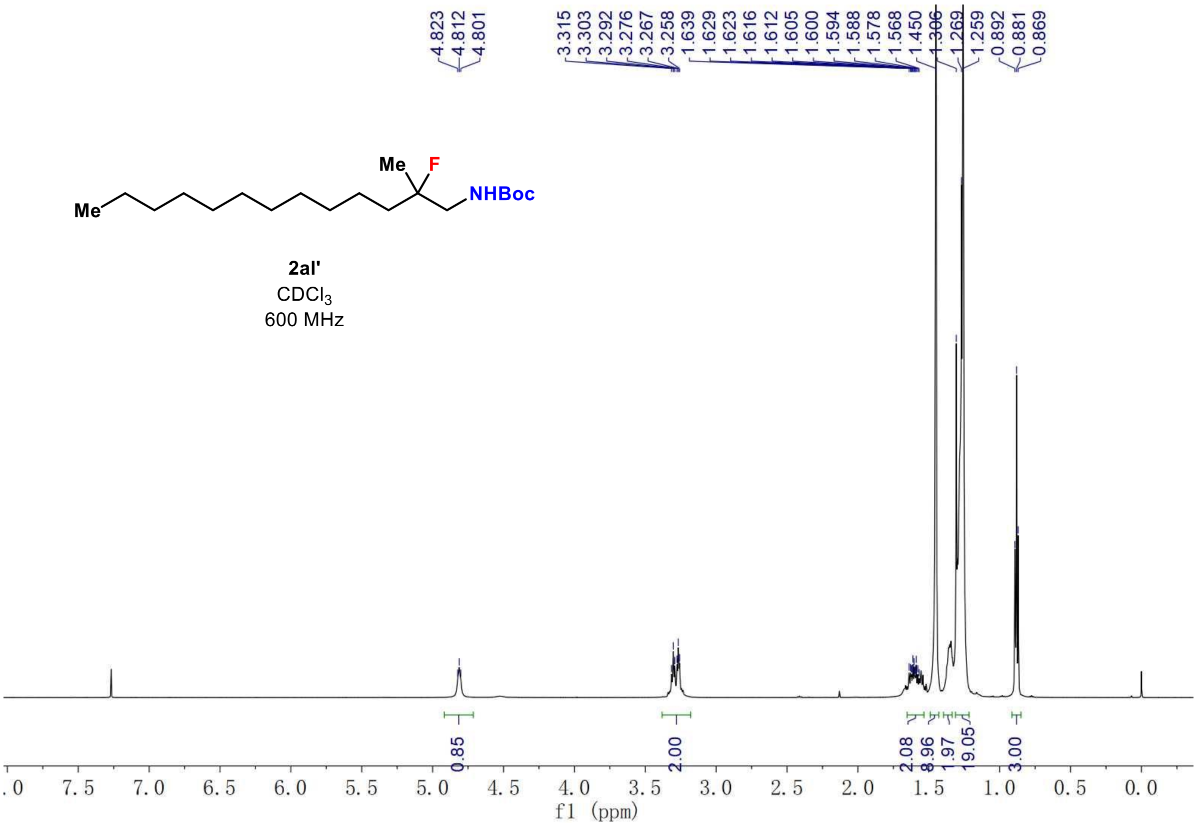

Supplementary Fig. 152. **^{1}H NMR** Spectra of **2al'**

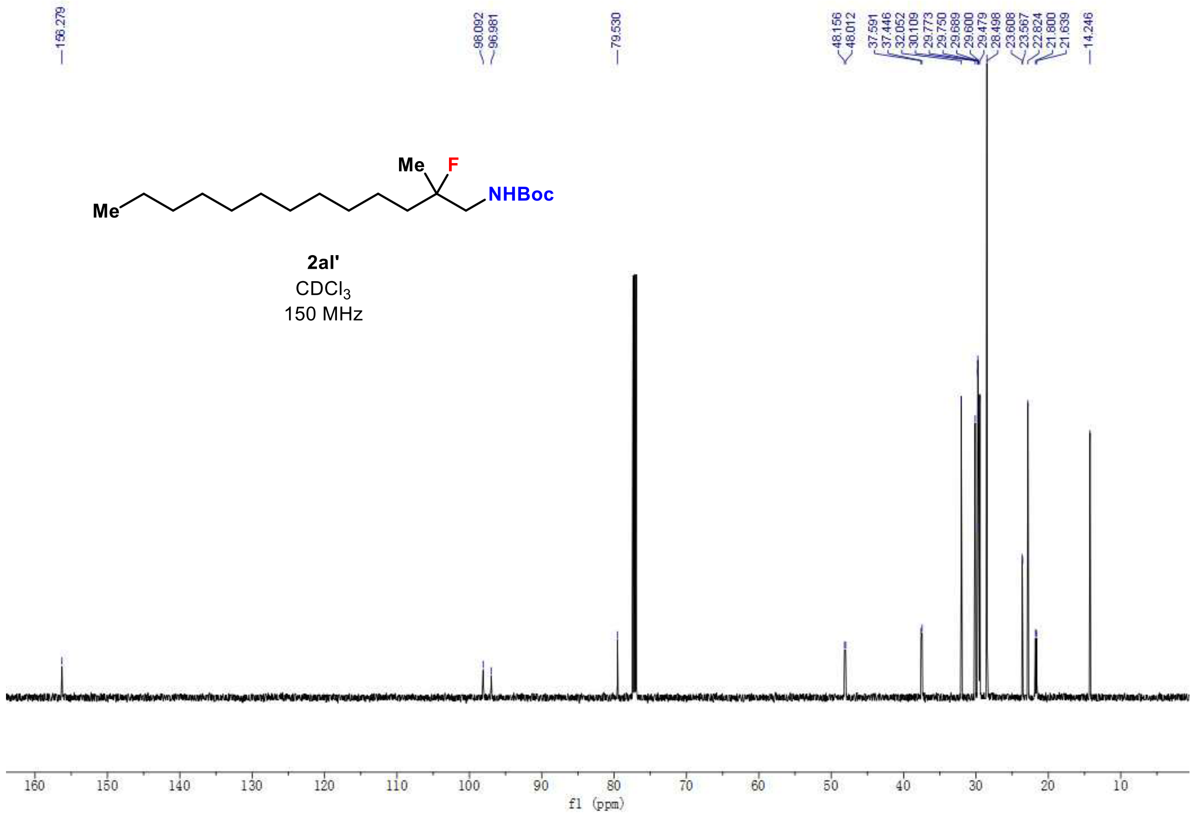

Supplementary Fig. 153. **^{13}C NMR** Spectra of **2al'**

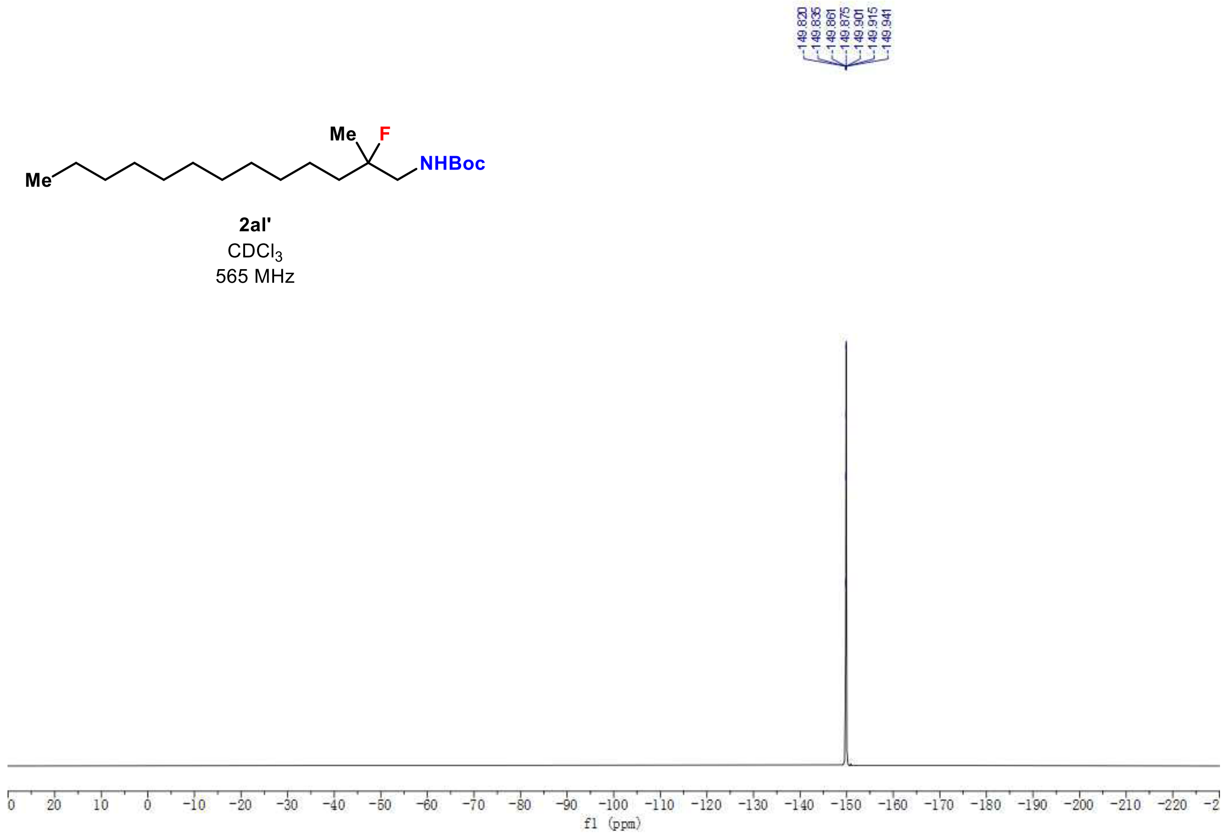

Supplementary Fig. 154. **^{19}F NMR** Spectra of **2al'**

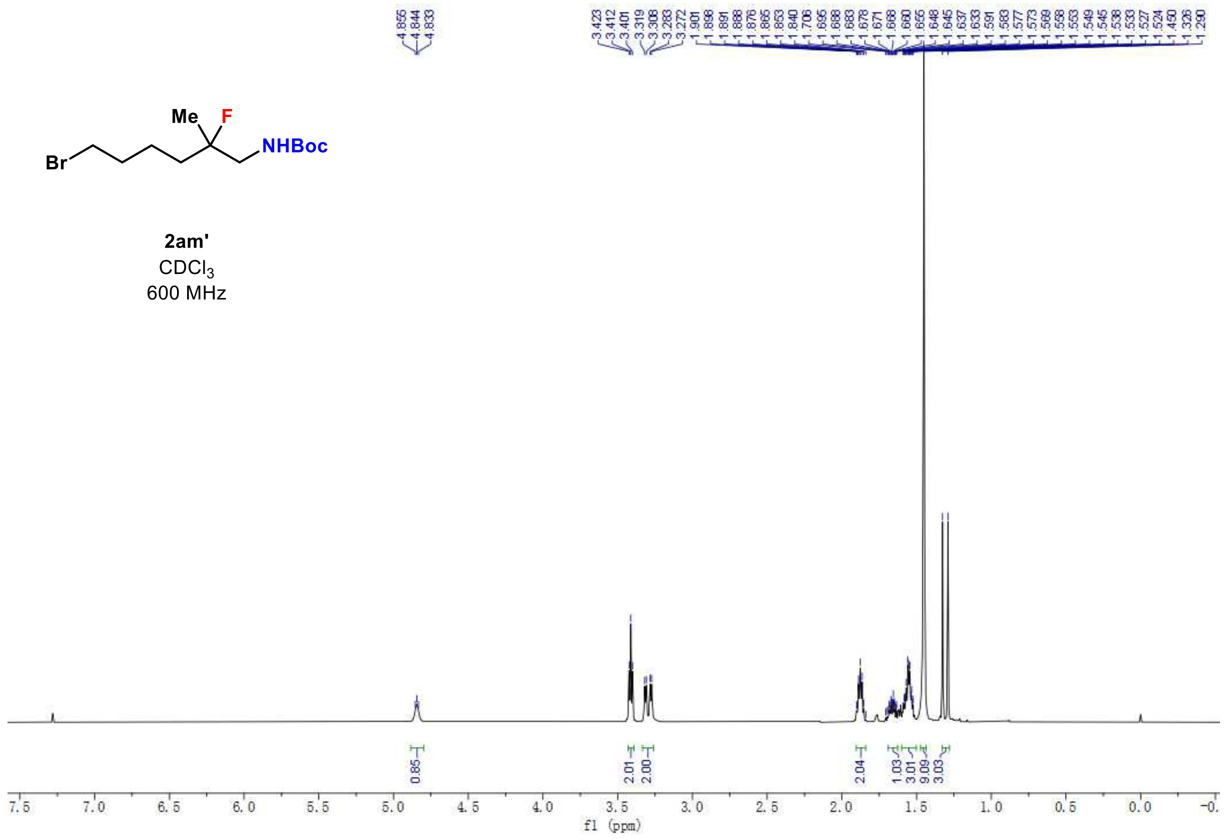

Supplementary Fig. 155. ^{1}H NMR Spectra of **2am'**

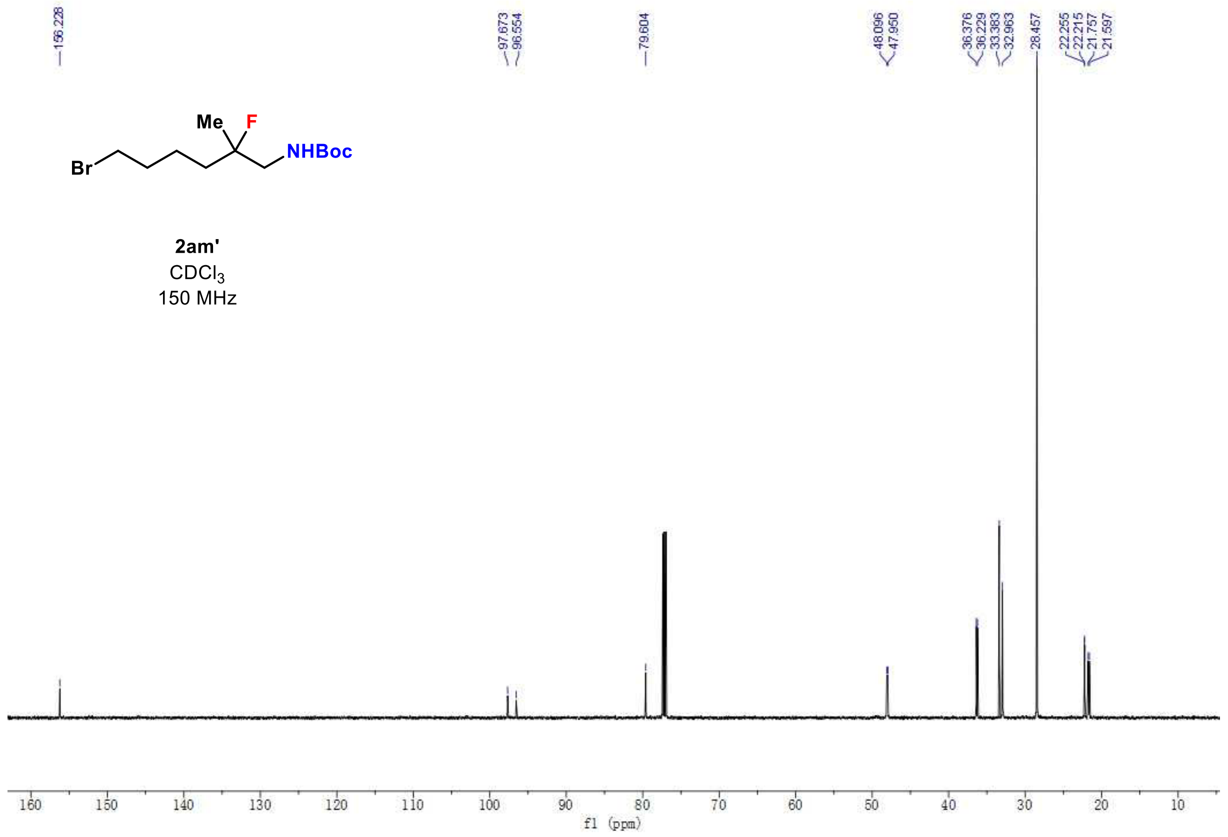

Supplementary Fig. 156. ^{13}C NMR Spectra of **2am'**

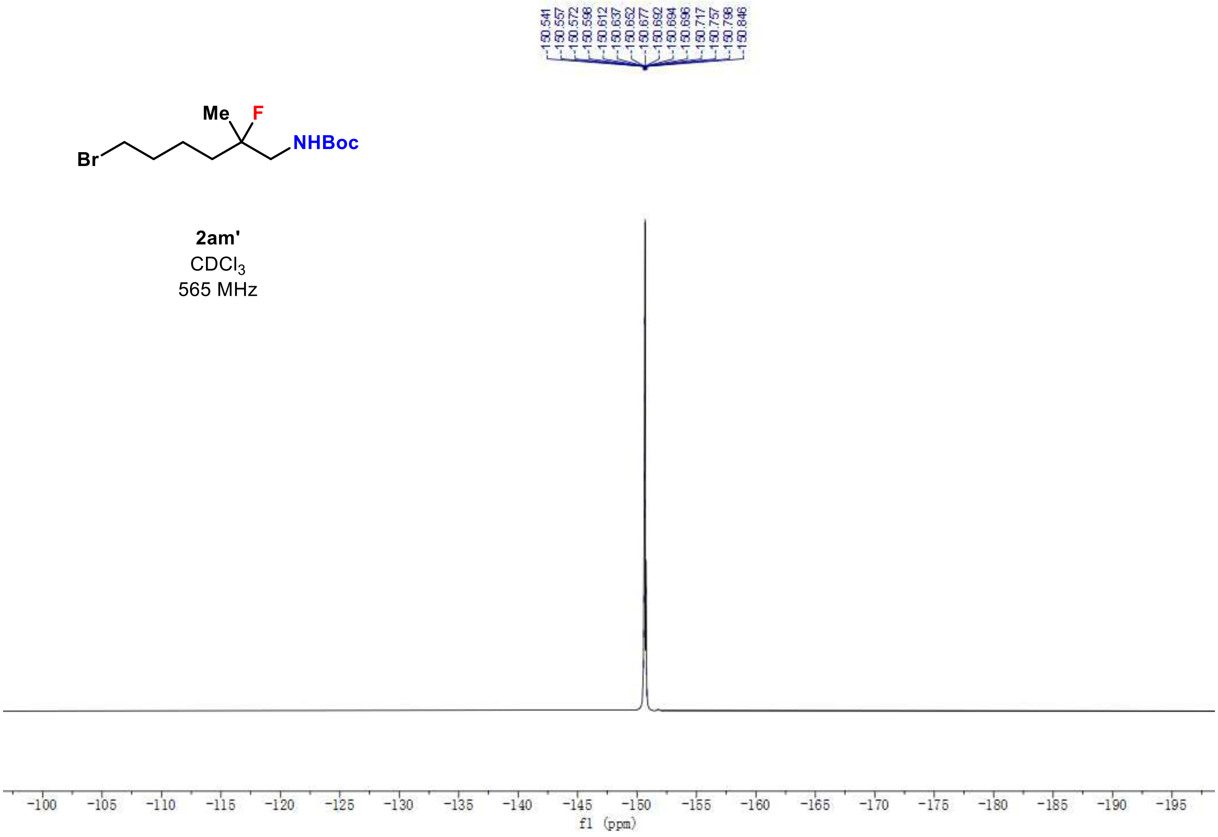

Supplementary Fig. 157. ^{19}**F NMR** Spectra of **2am'**

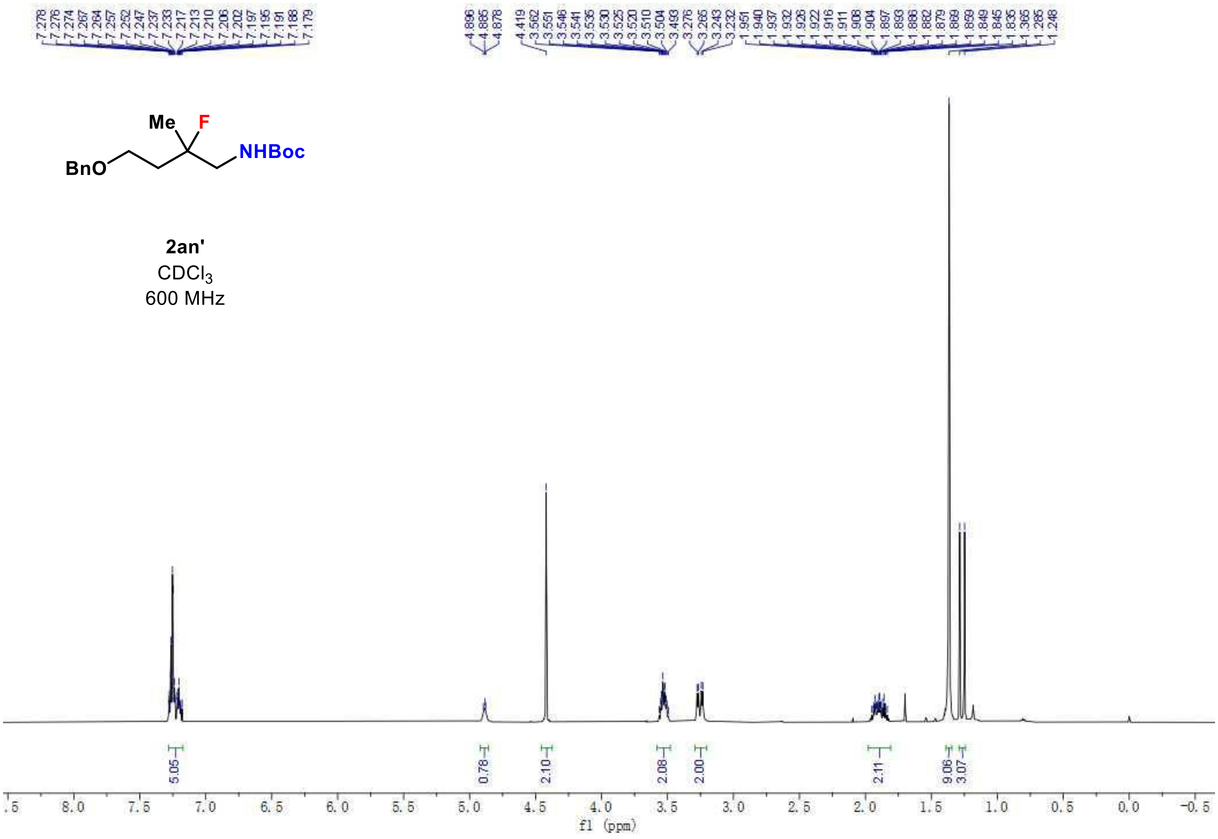

Supplementary Fig. 158. ^{1}**H NMR** Spectra of **2an'**

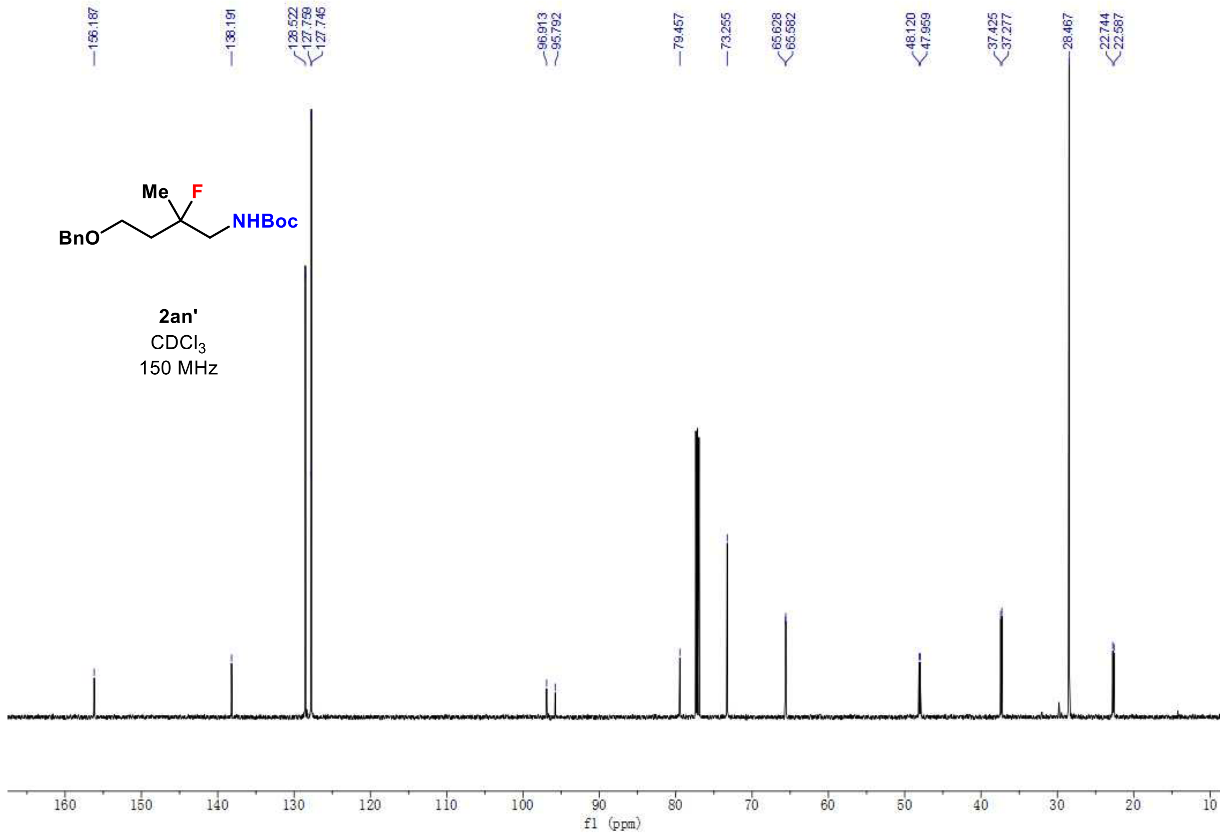

Supplementary Fig. 159. ^{13}**C NMR** Spectra of **2an'**

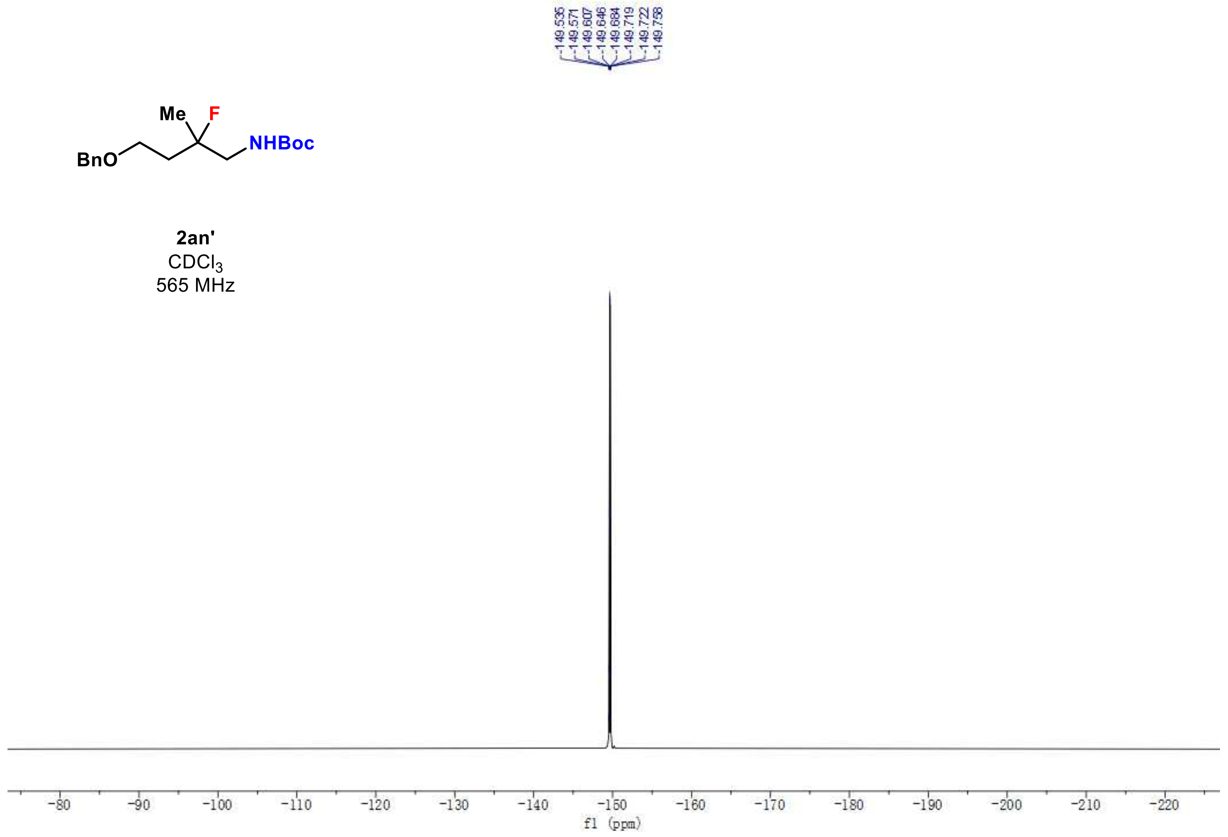

Supplementary Fig. 160. ^{19}**F NMR** Spectra of **2an'**

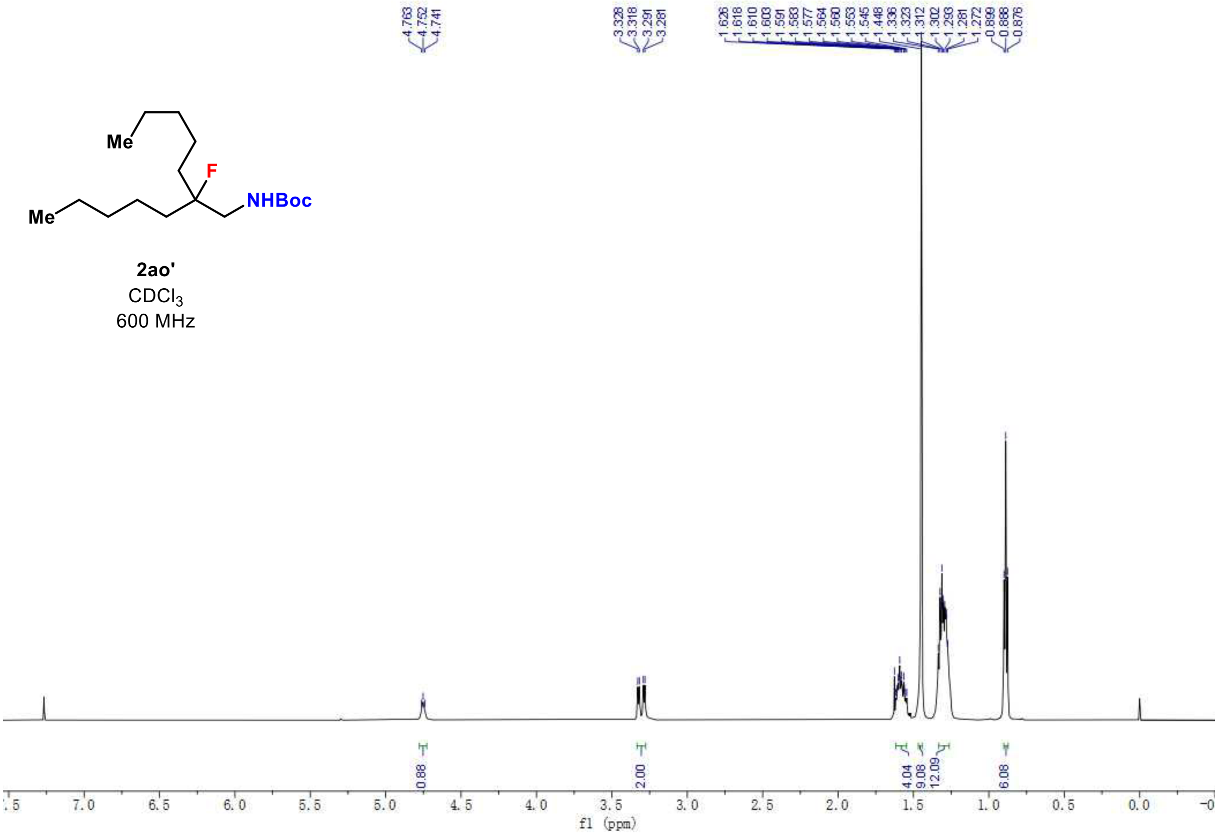

Supplementary Fig. 161. ^{1}**H NMR** Spectra of **2ao'**

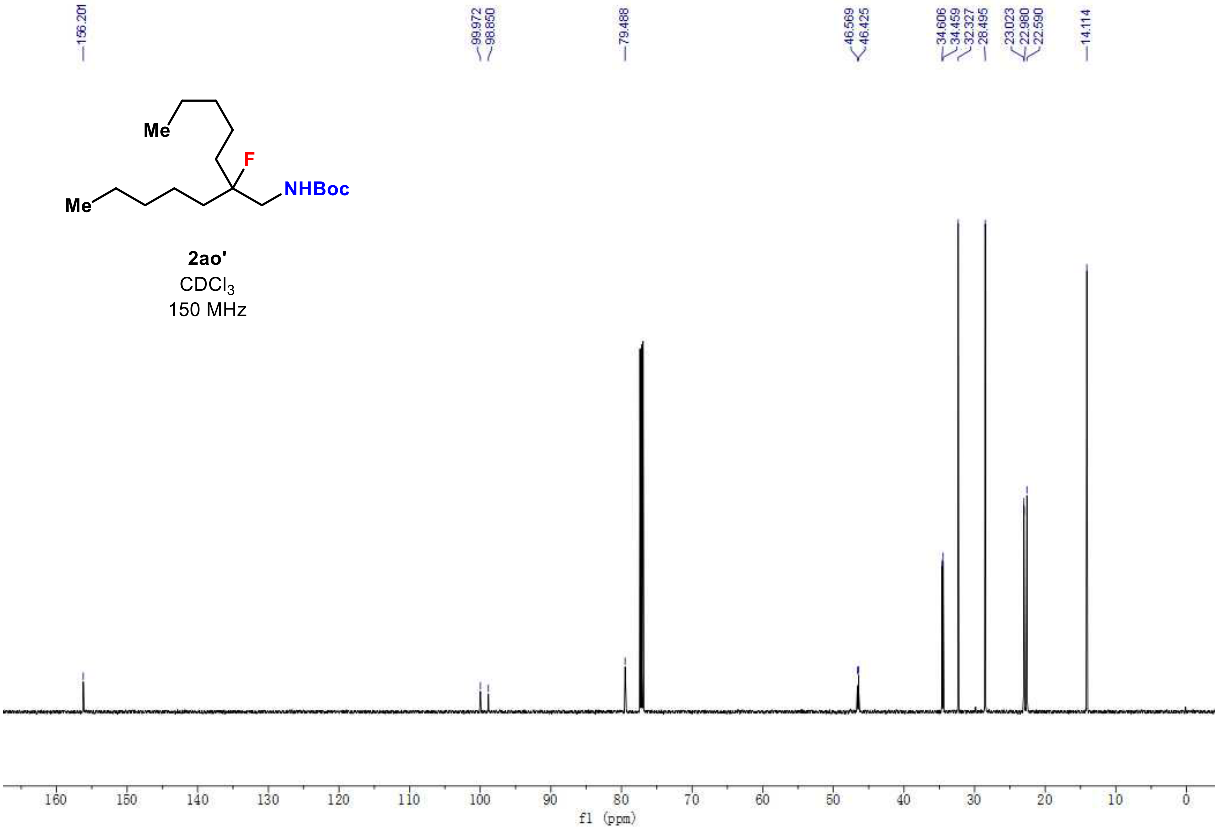

Supplementary Fig. 162. ^{13}**C NMR** Spectra of **2ao'**

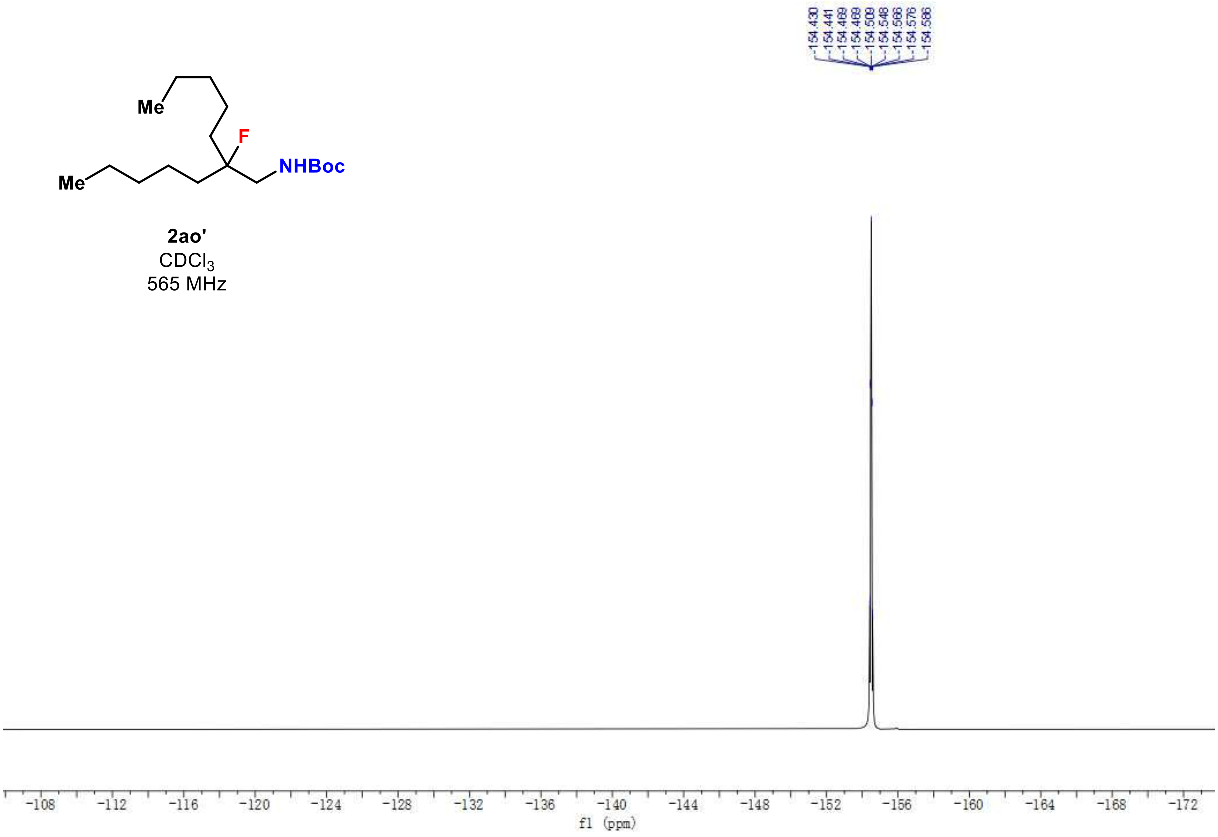

Supplementary Fig. 164. **^{19}F NMR** Spectra of **2ao'**

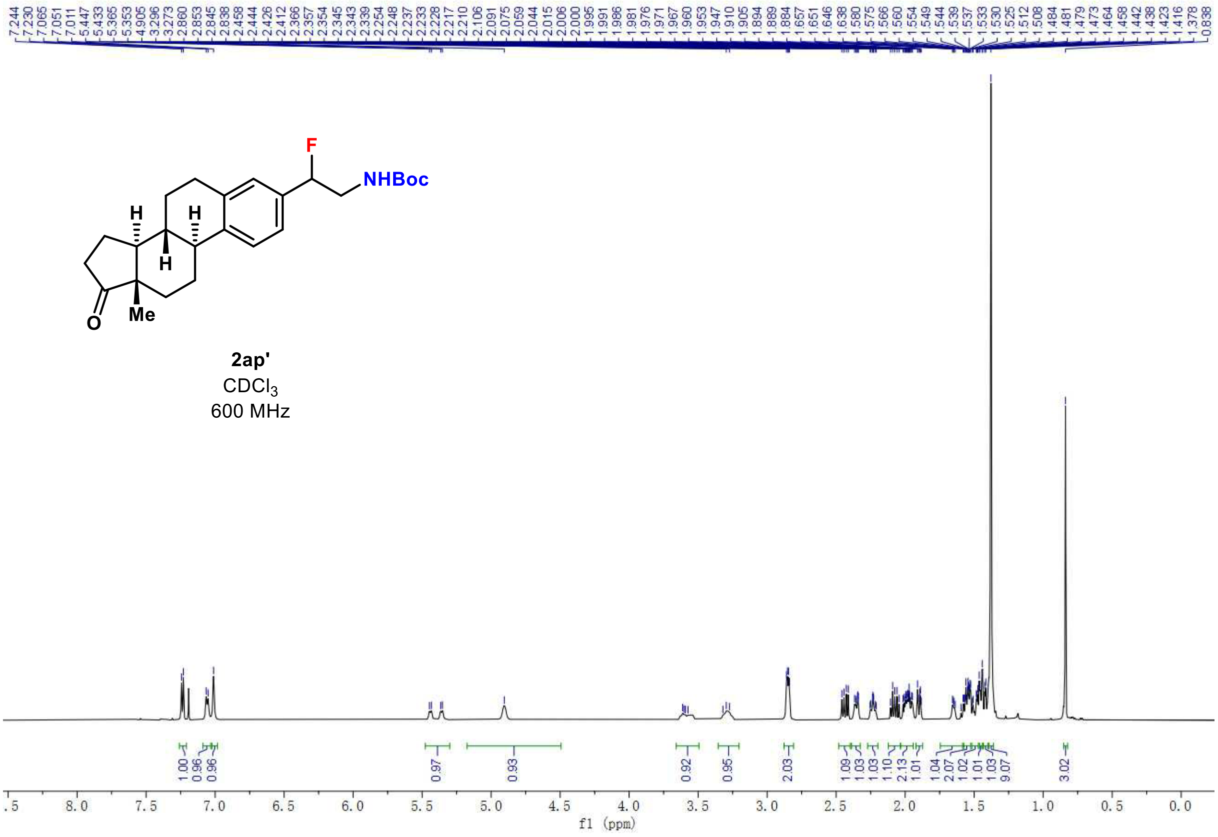

Supplementary Fig. 164. **^{1}H NMR** Spectra of **2ap'**

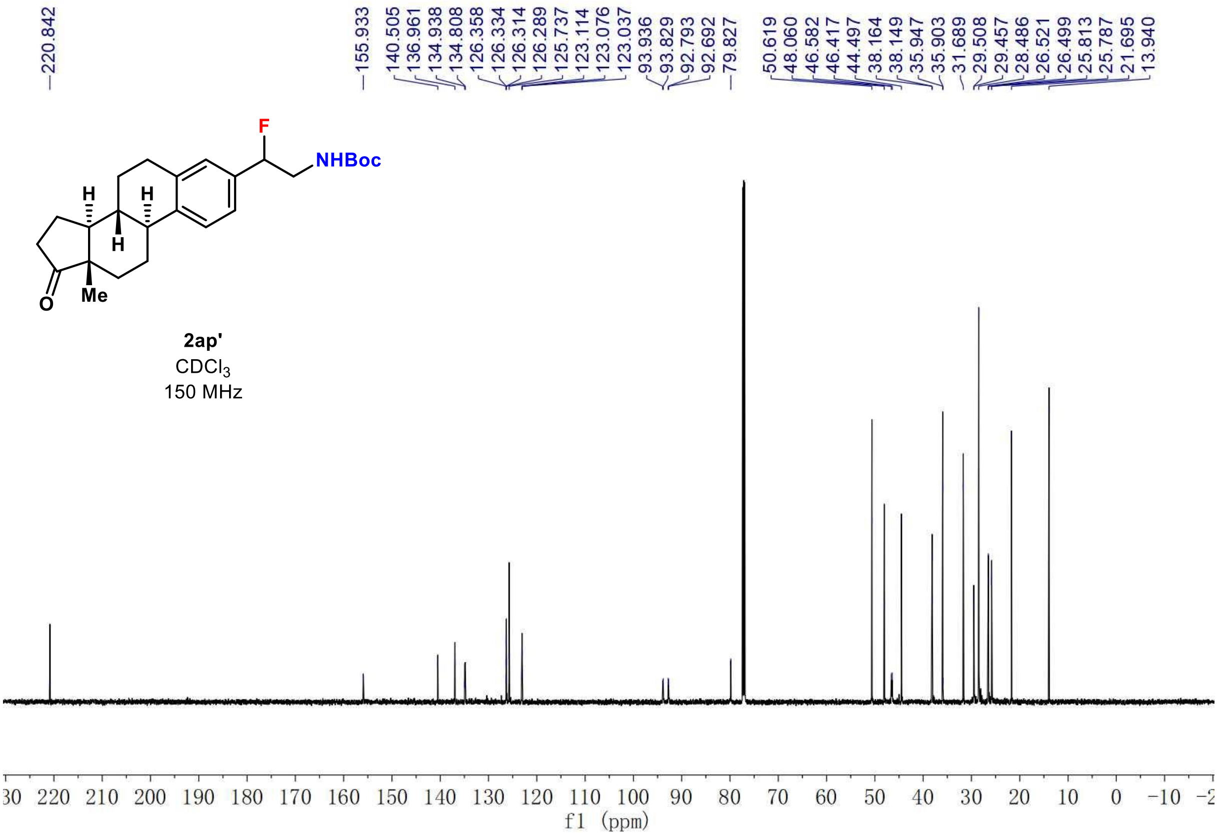

Supplementary Fig. 165. ^{13}**C NMR** Spectra of **2ap'**

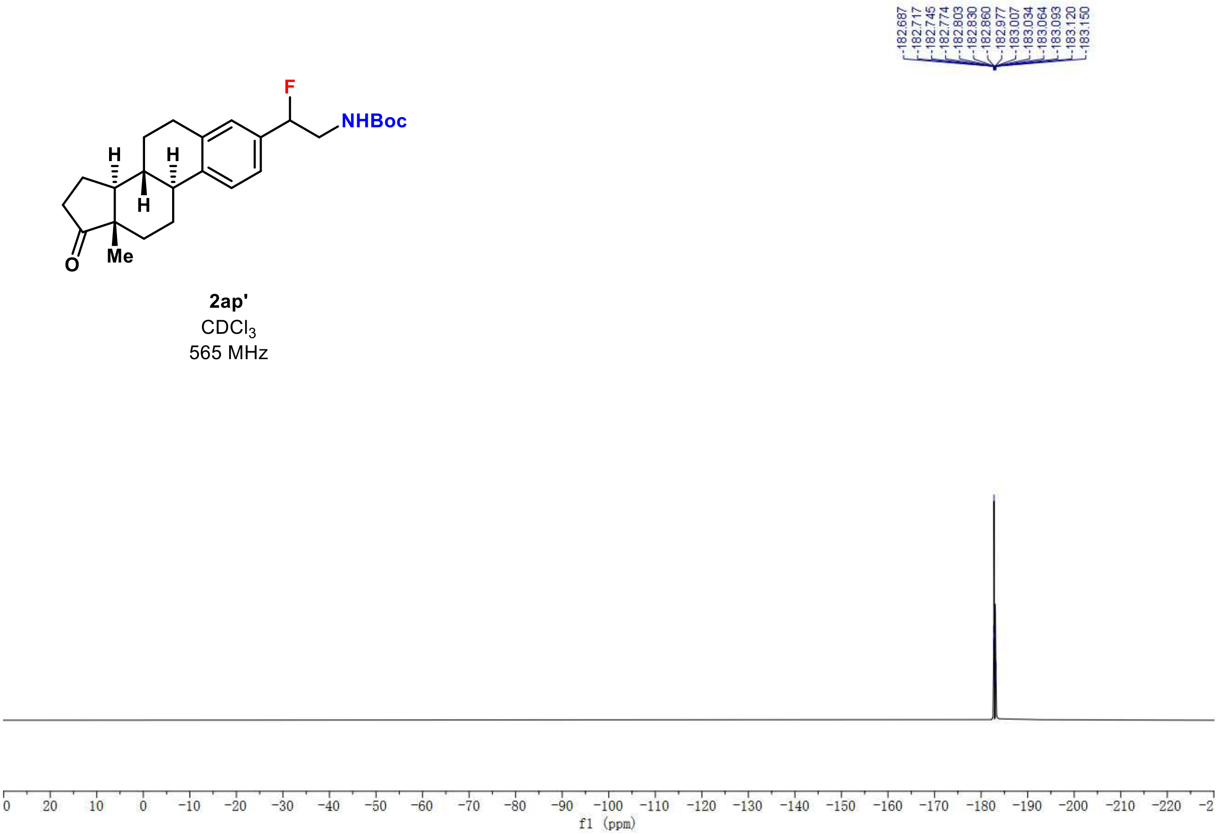

Supplementary Fig. 166. ^{19}**F NMR** Spectra of **2ap'**

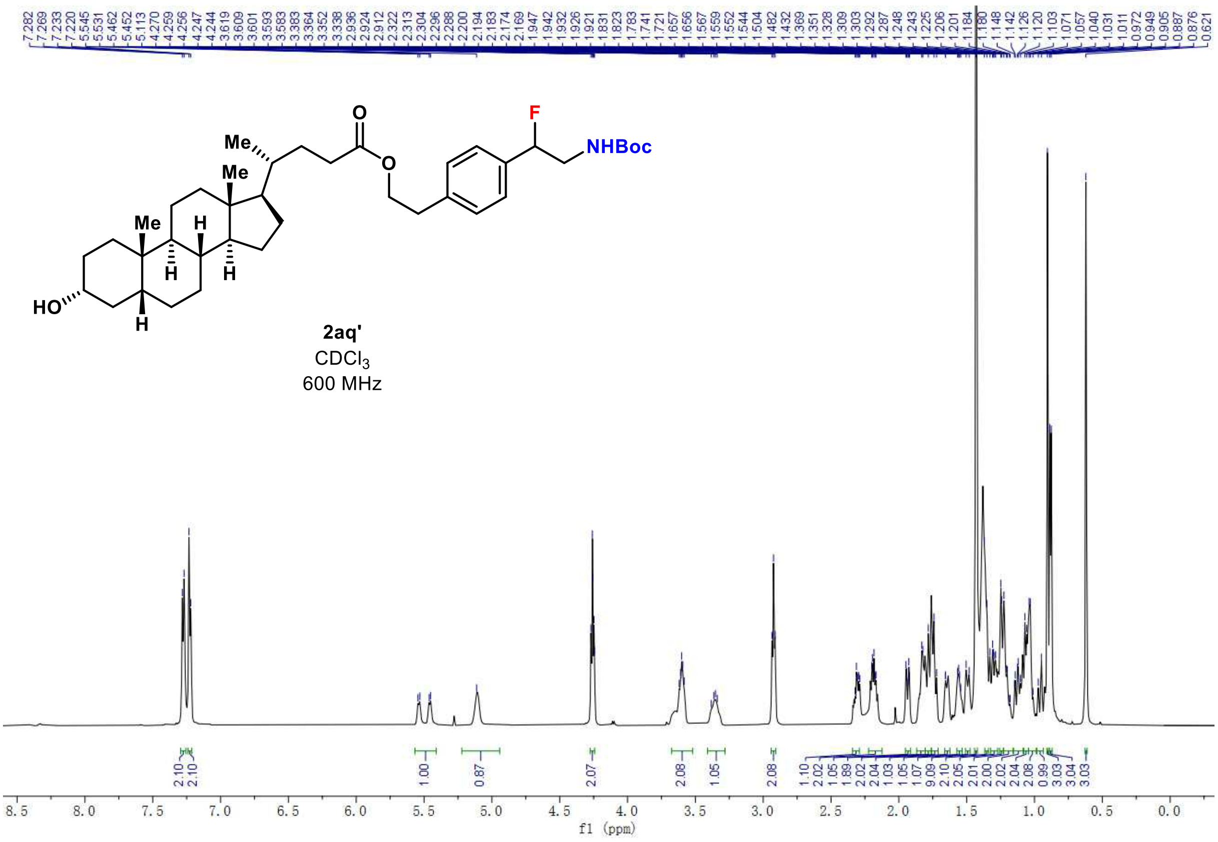

Supplementary Fig. 167. ^{1}**H NMR** Spectra of **2aq'**

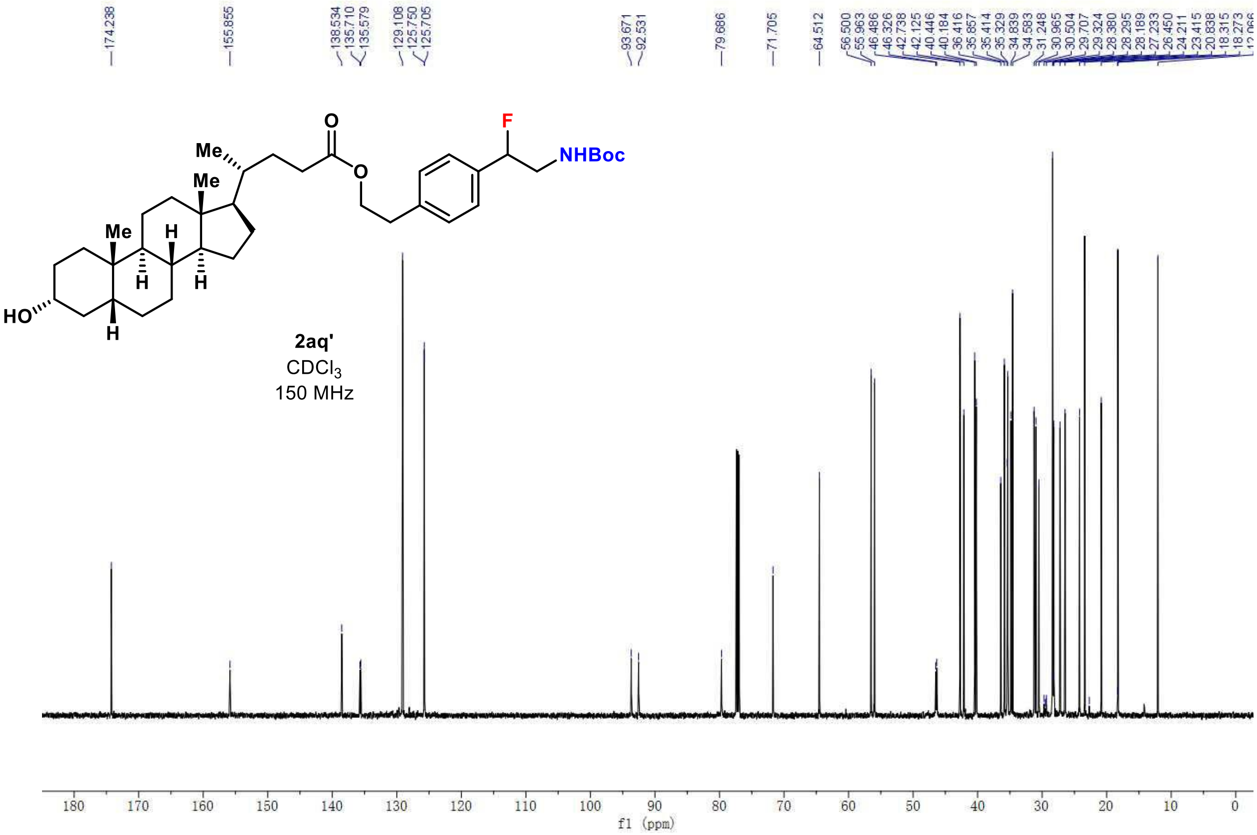

Supplementary Fig. 168. ^{13}**C NMR** Spectra of **2aq'**

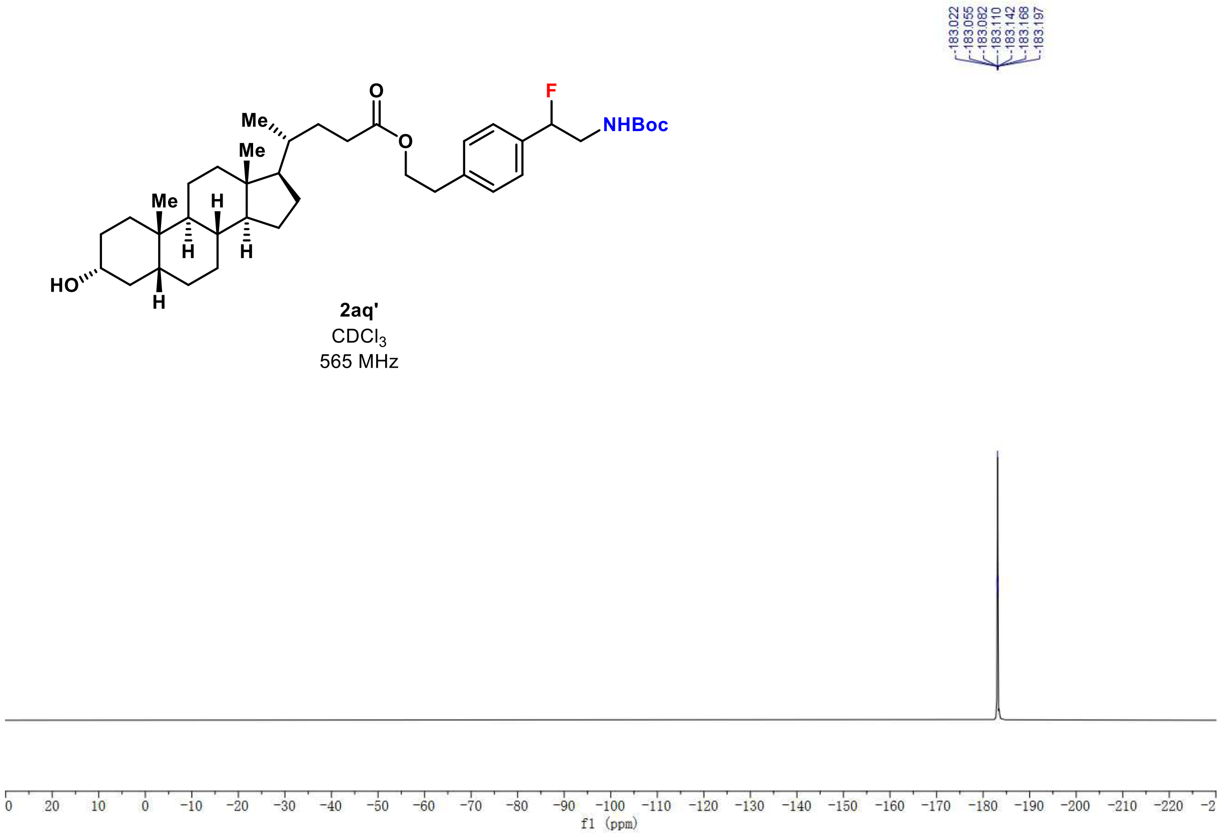

Supplementary Fig. 169. **^{19}F NMR** Spectra of **2aq'**

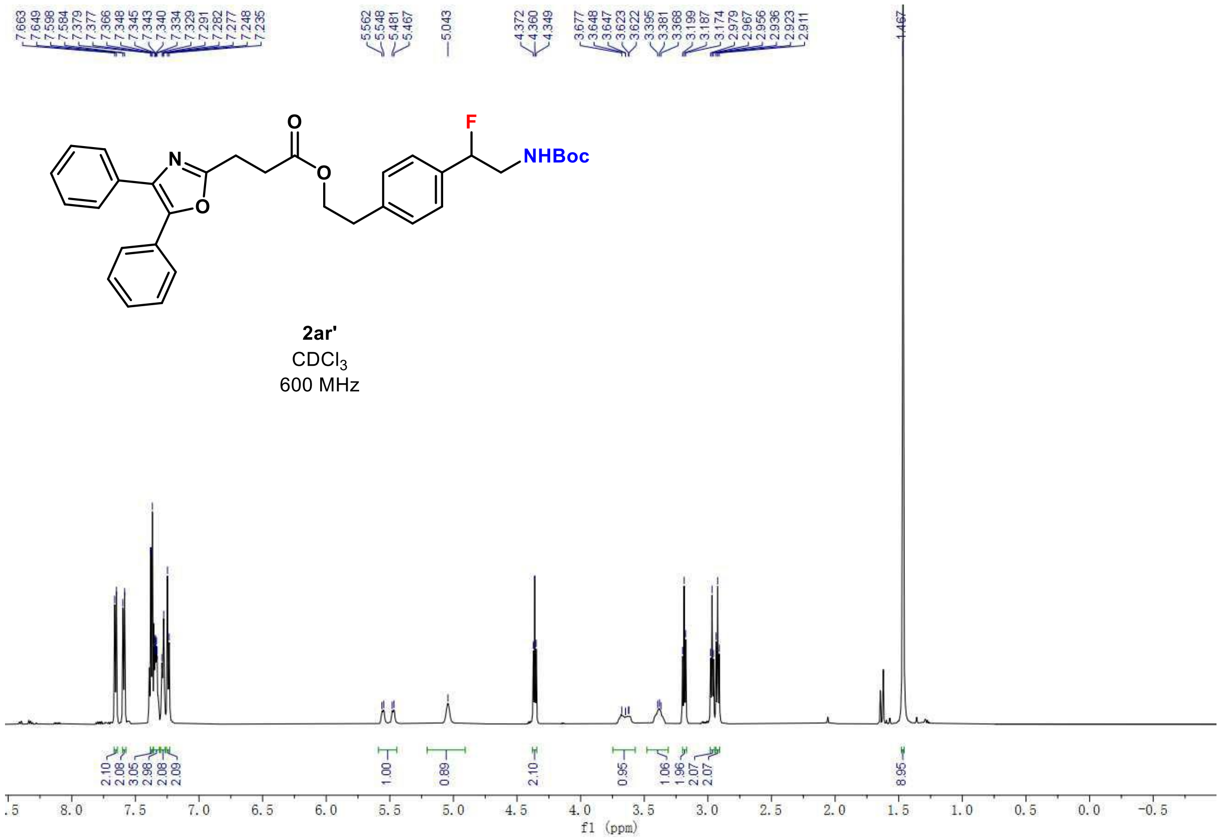

Supplementary Fig. 170. **^{1}H NMR** Spectra of **2ar'**

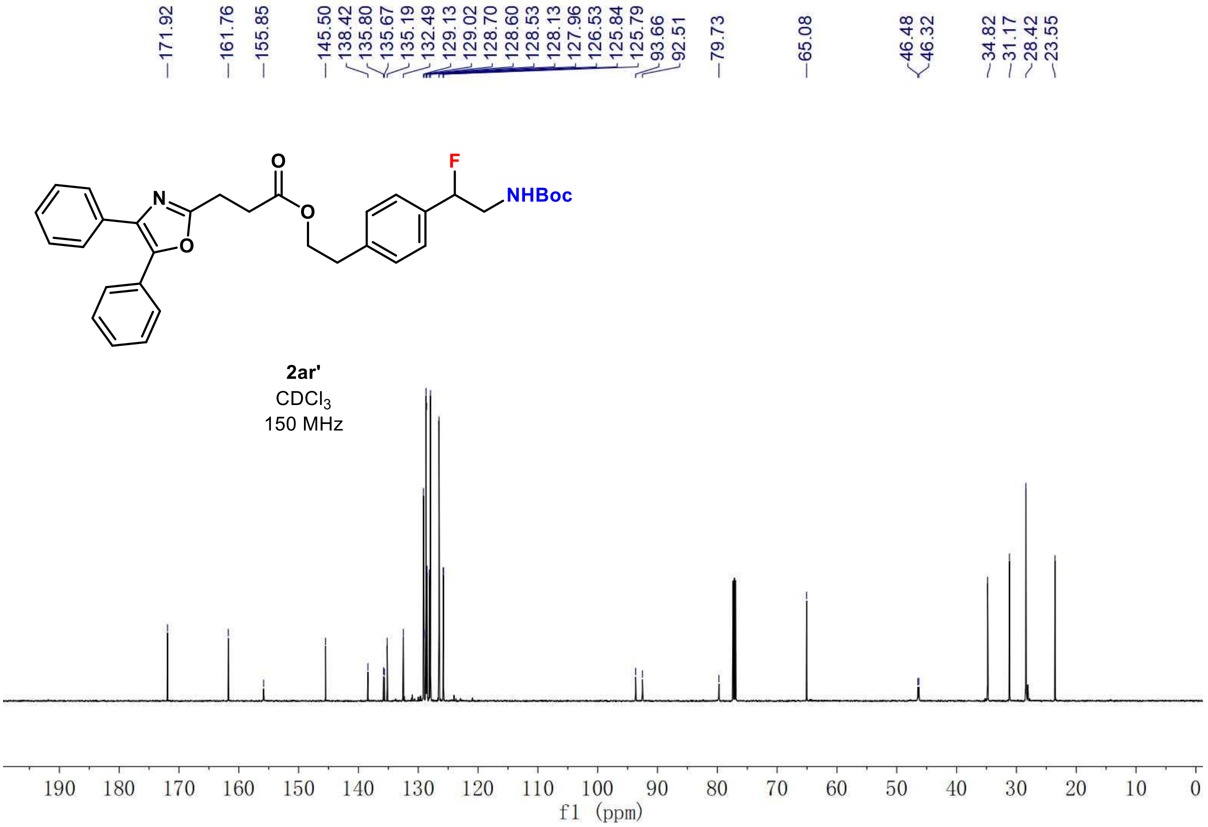

Supplementary Fig. 171. ^{13}**C NMR** Spectra of **2ar'**

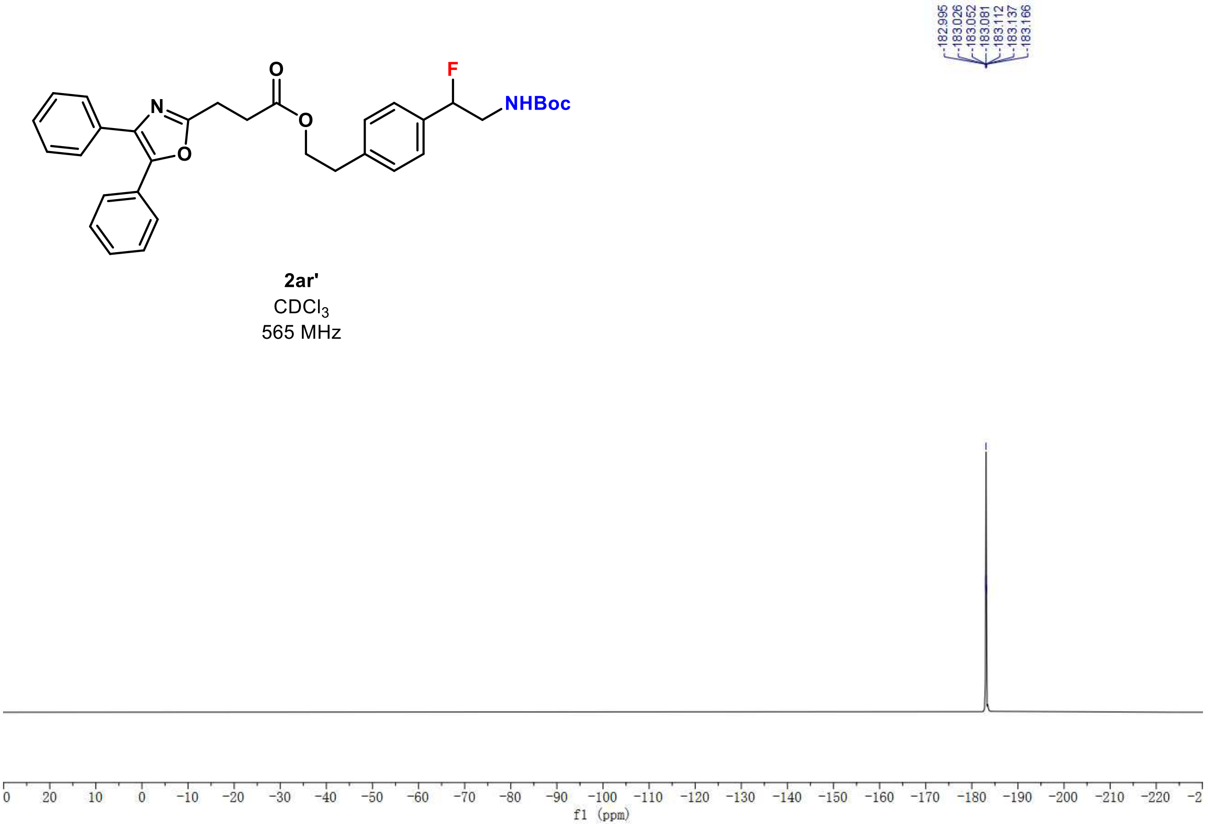

Supplementary Fig. 172. ^{19}**F NMR** Spectra of **2ar'**

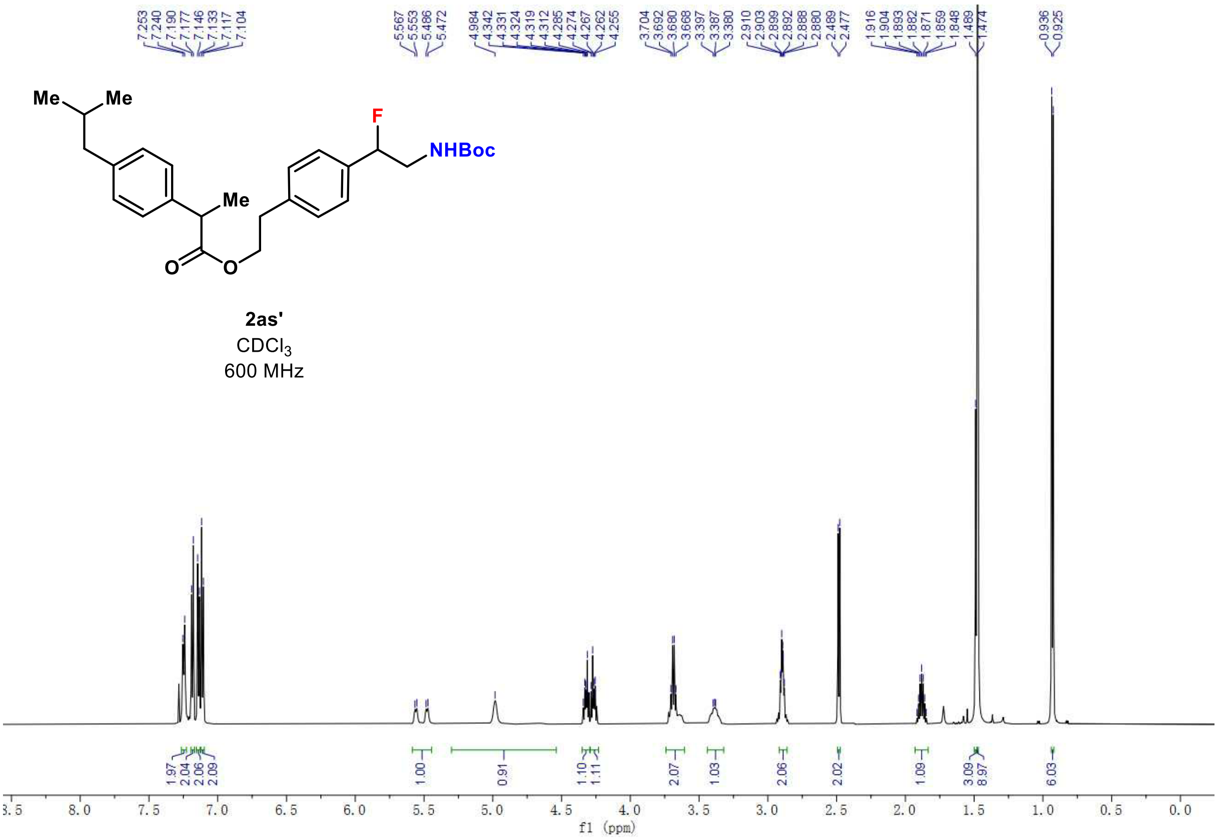

Supplementary Fig. 173. ^{1}**H NMR** Spectra of **2as'**

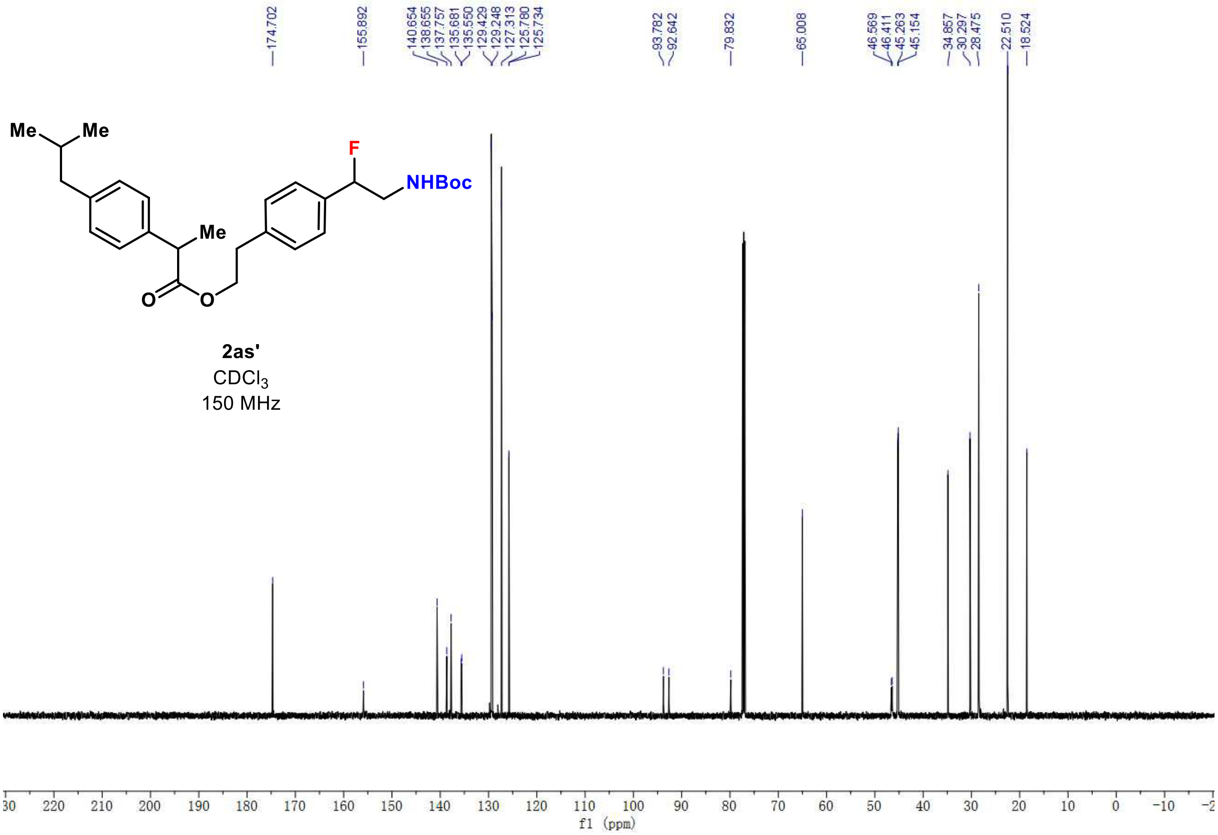

Supplementary Fig. 174. ^{13}**C NMR** Spectra of **2as'**

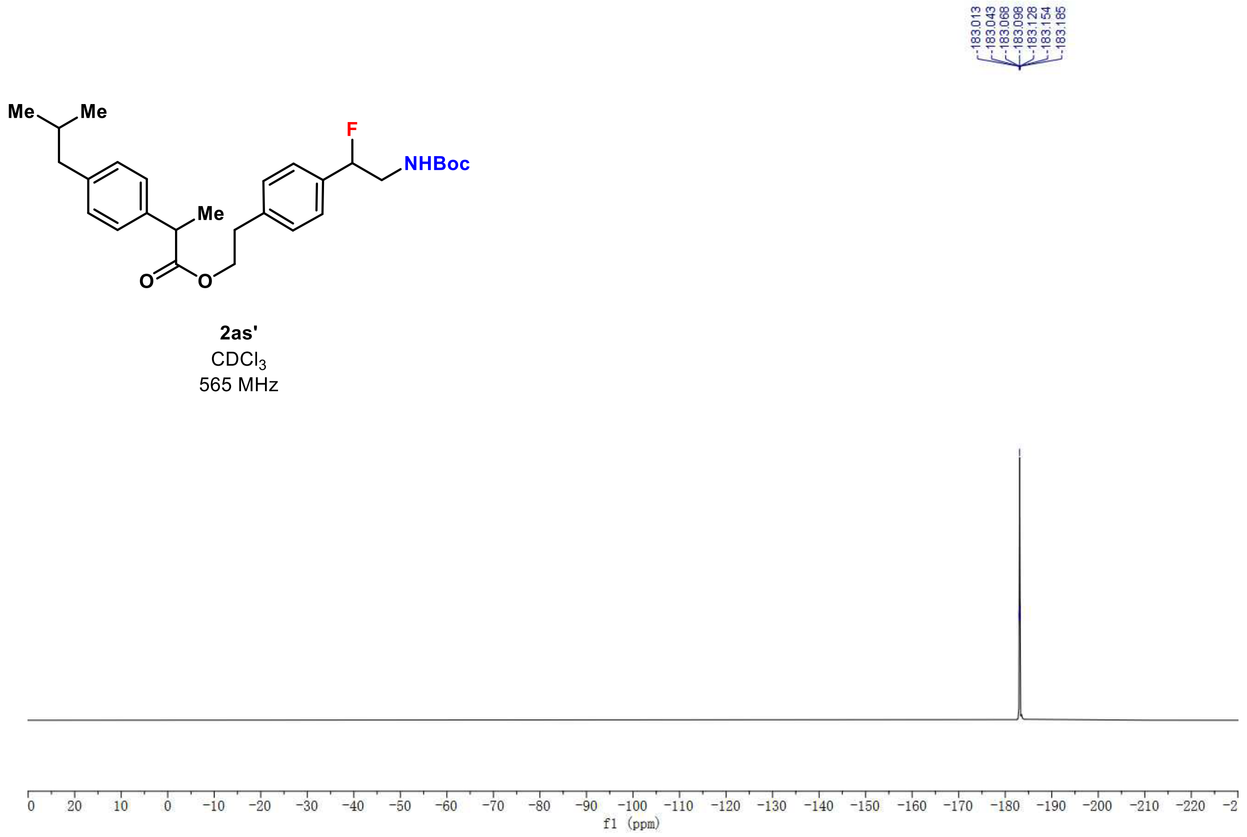

Supplementary Fig. 175. **^{19}F NMR** Spectra of **2as'**

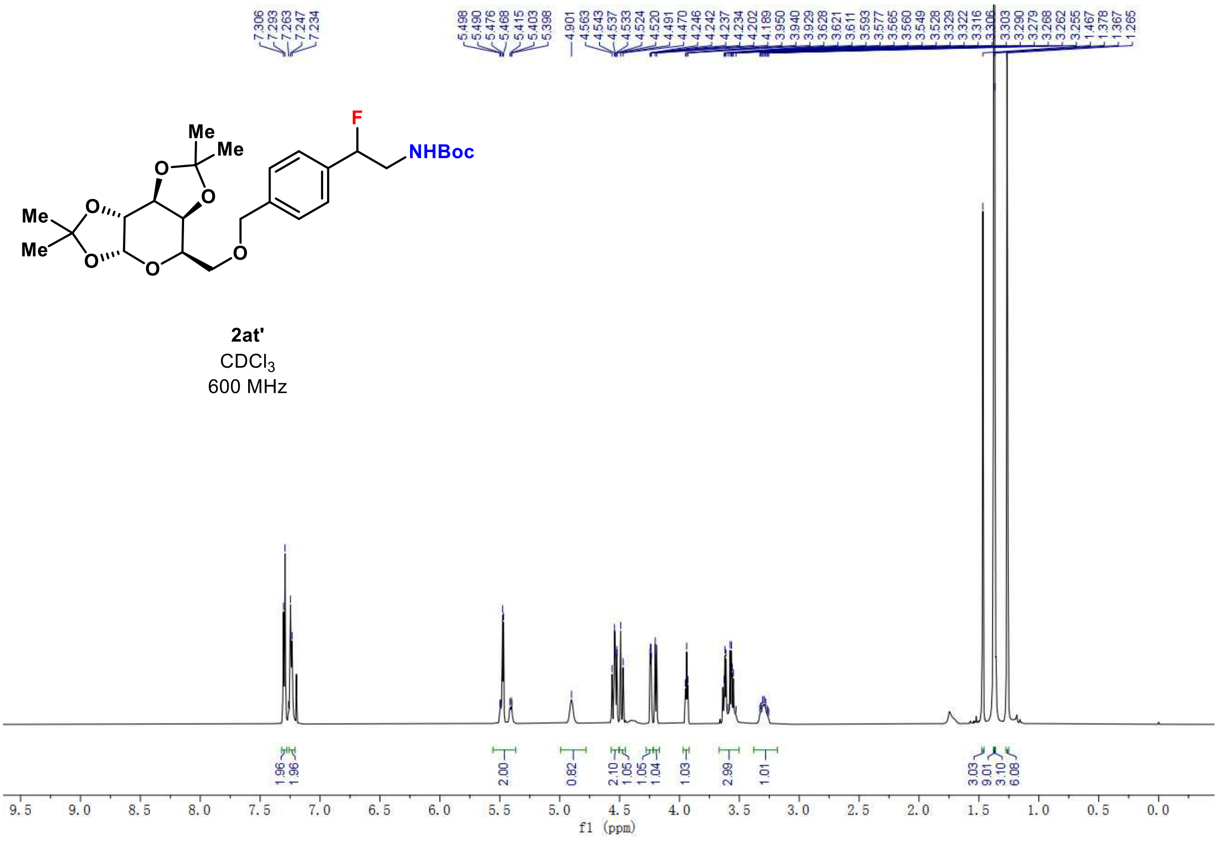

Supplementary Fig. 176. **^{1}H NMR** Spectra of **2at'**

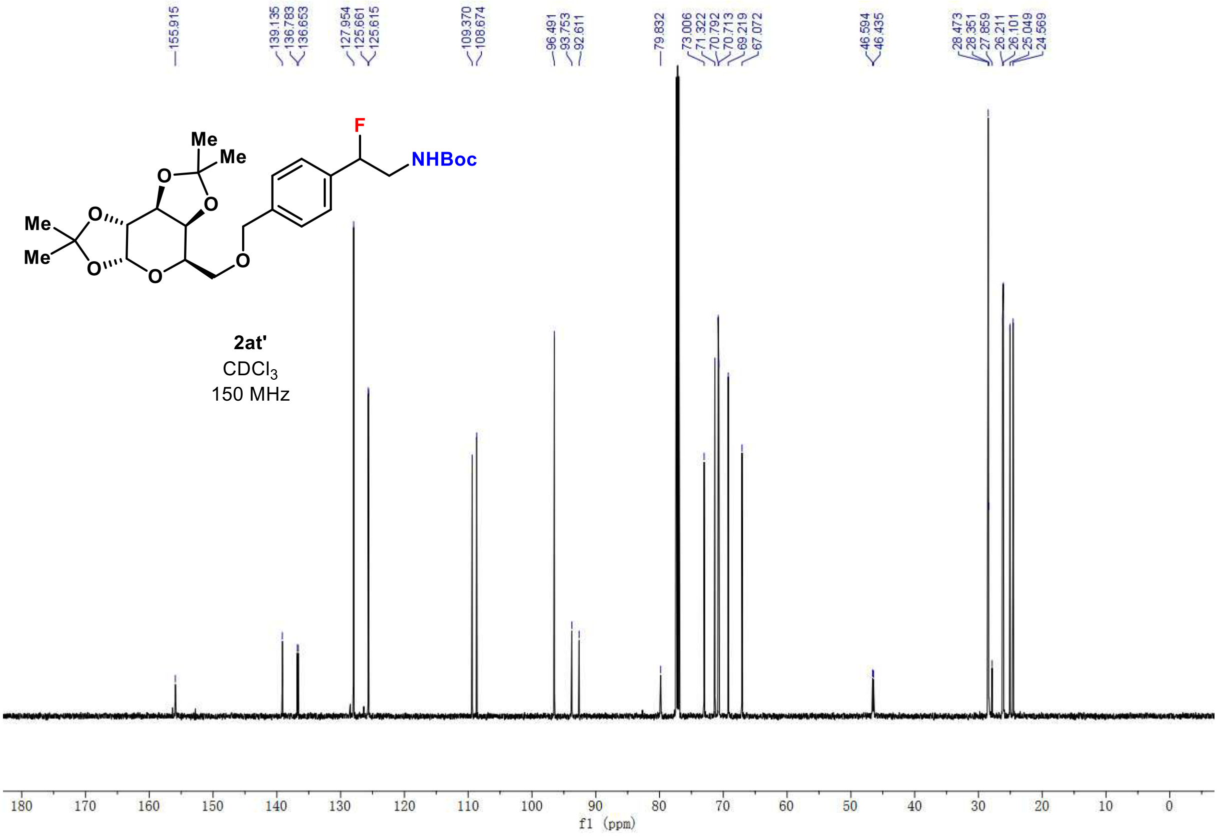

Supplementary Fig. 177. ^{13}**C NMR** Spectra of **2at'**

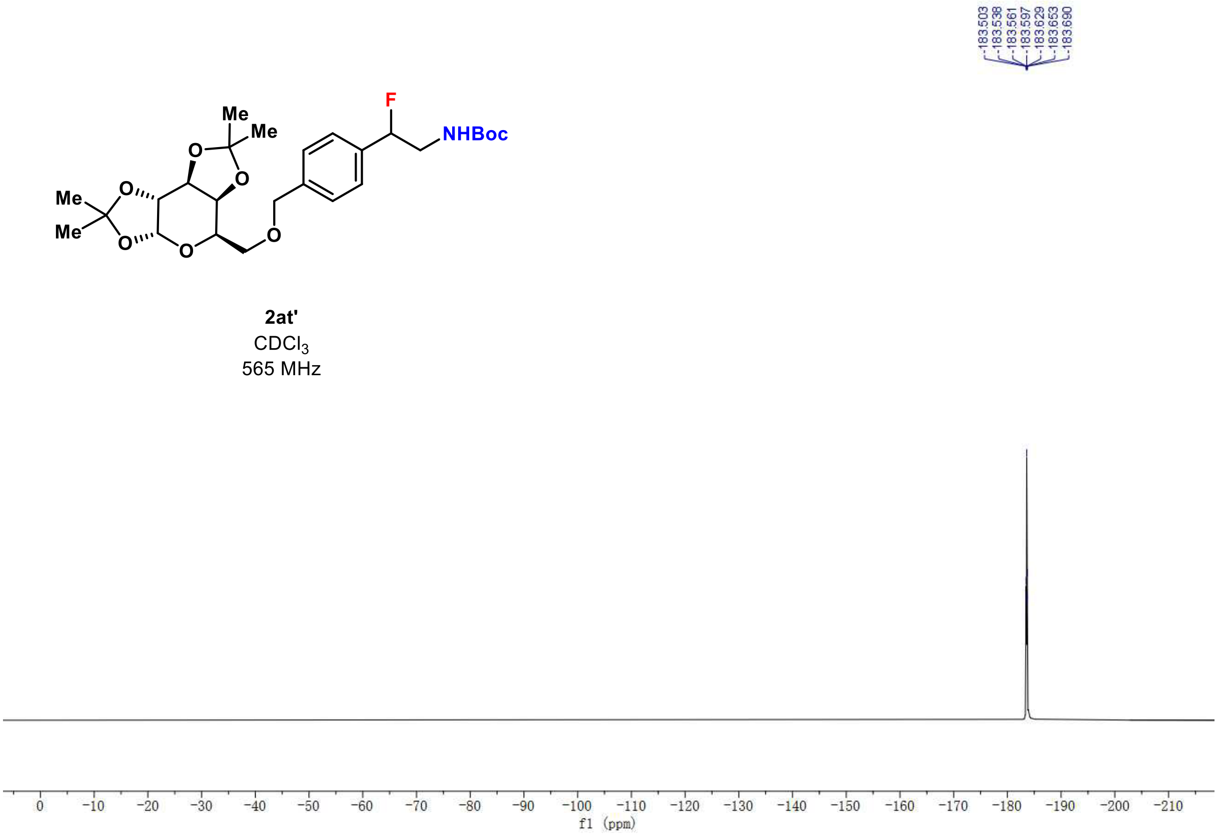

Supplementary Fig. 178. ^{19}**F NMR** Spectra of **2at'**

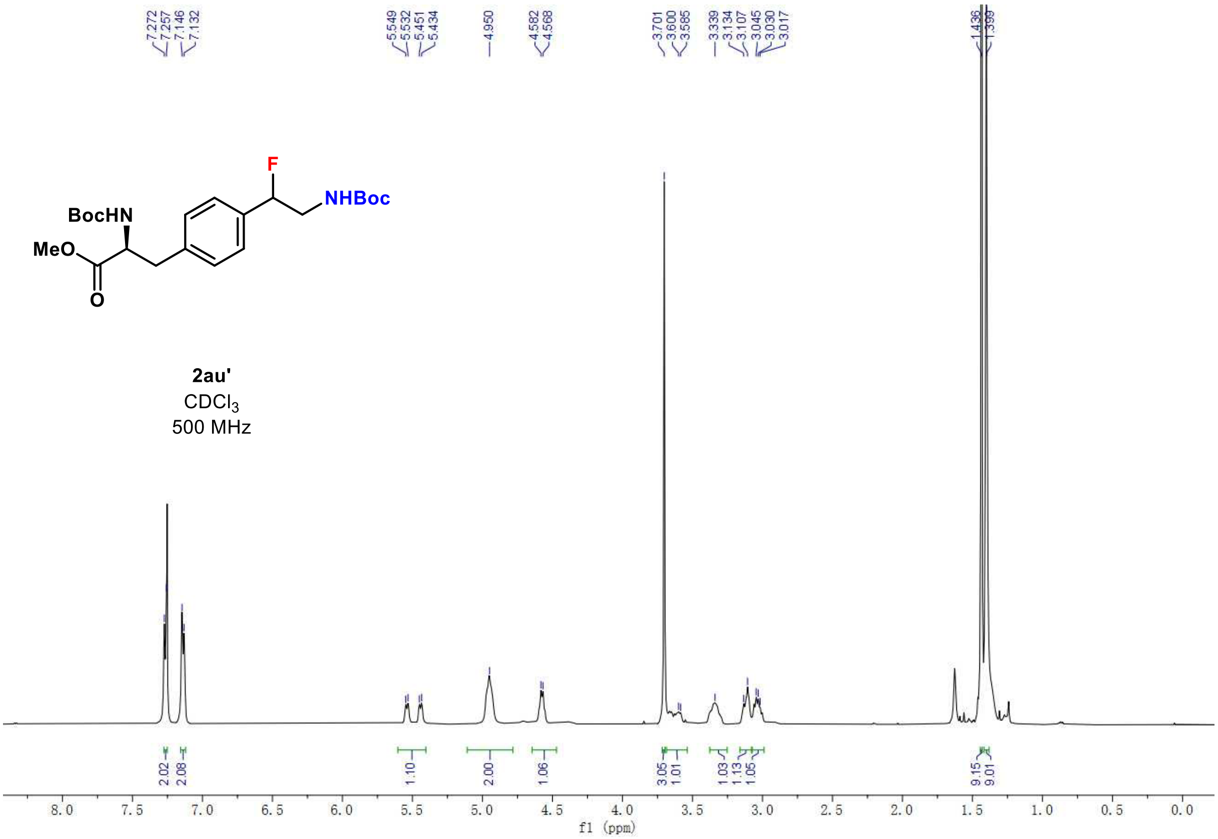

Supplementary Fig. 179. ^{1}H NMR Spectra of **2au'**

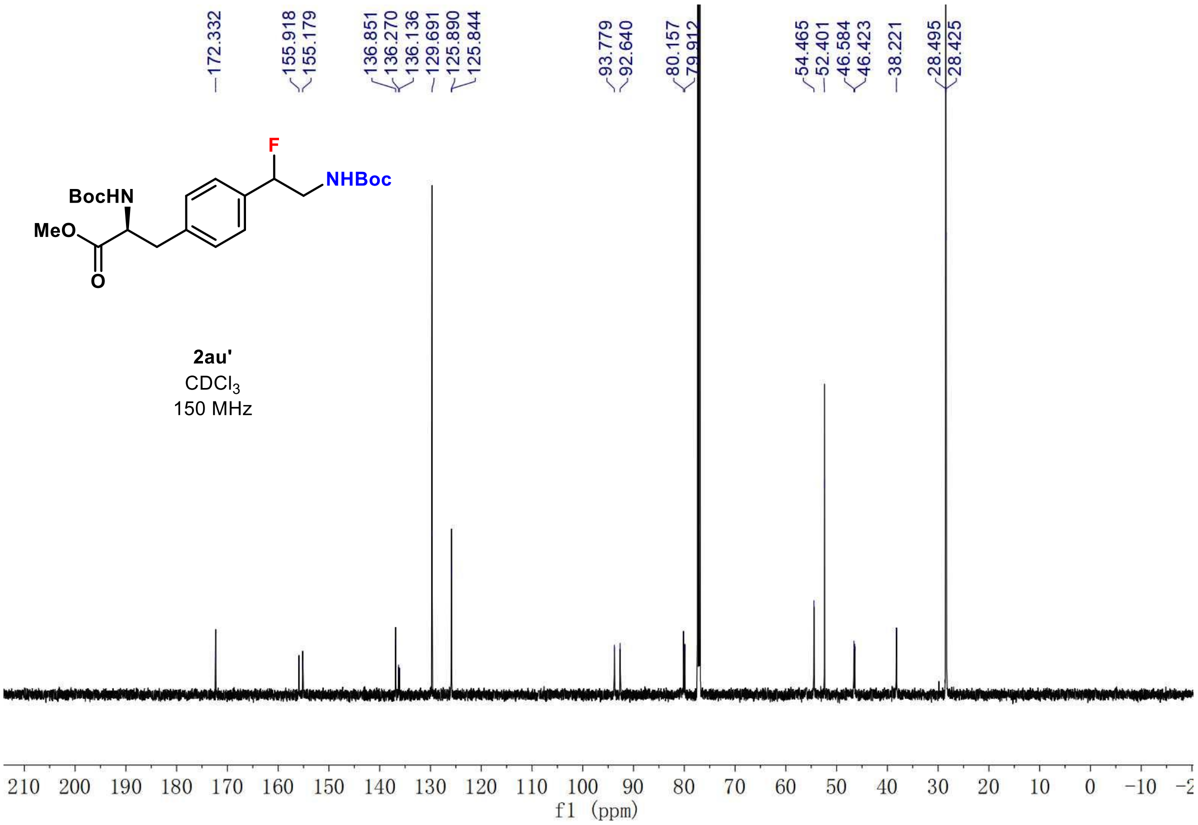

Supplementary Fig. 180. ^{13}C NMR Spectra of **2au'**

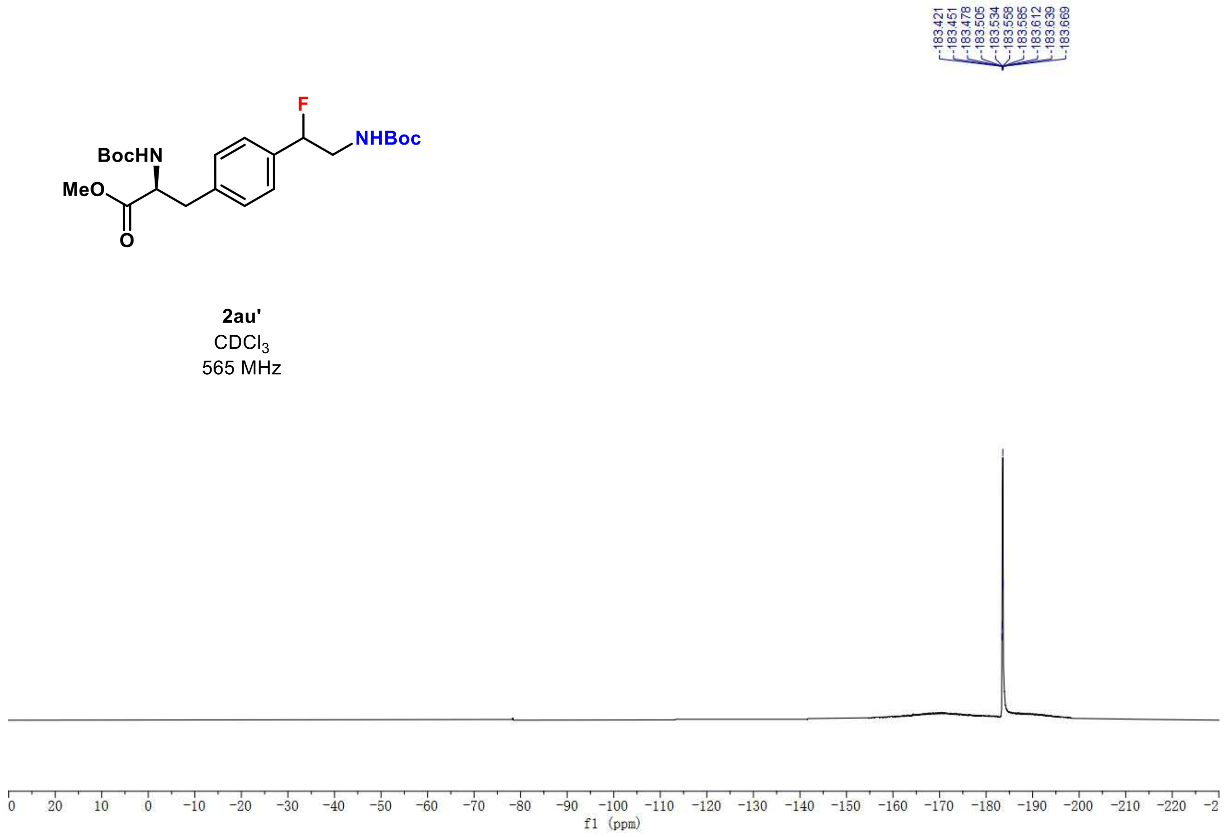

Supplementary Fig. 181. ^{19}**F NMR** Spectra of **2au'**

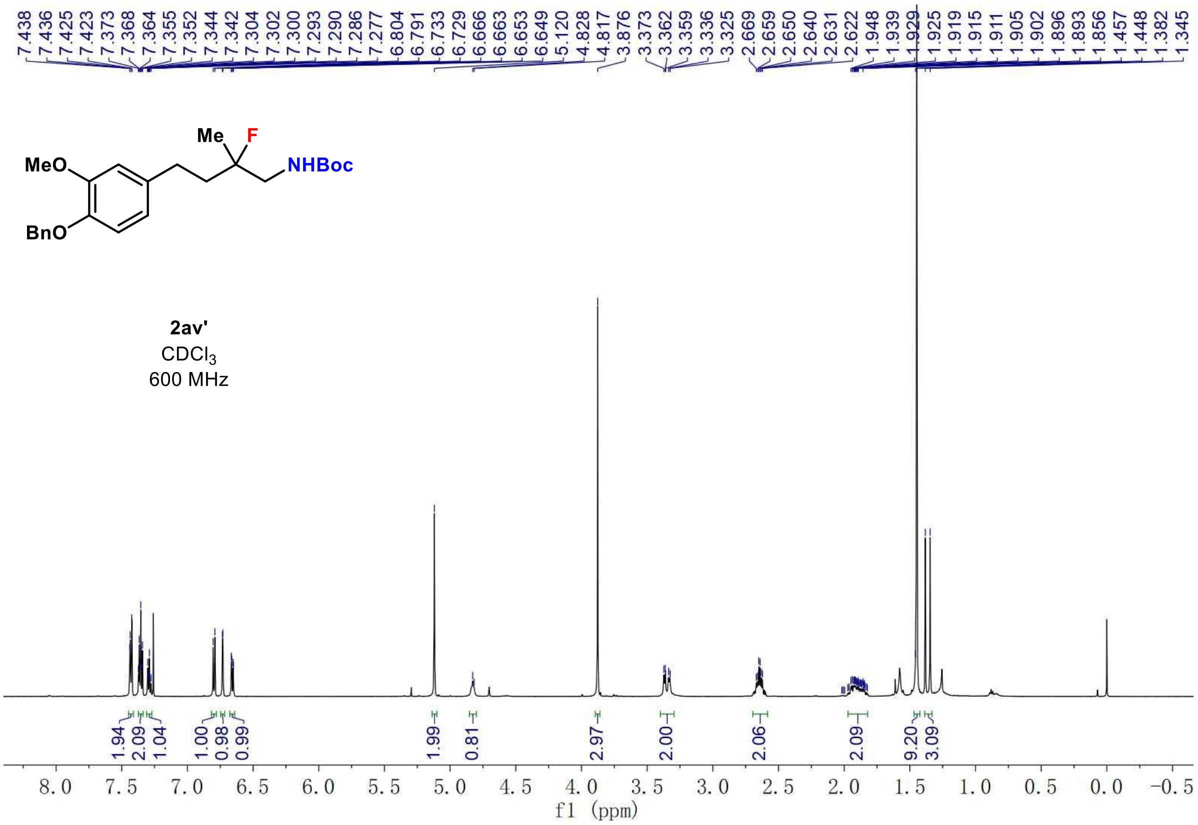

Supplementary Fig. 182. ^{1}**H NMR** Spectra of **2av'**

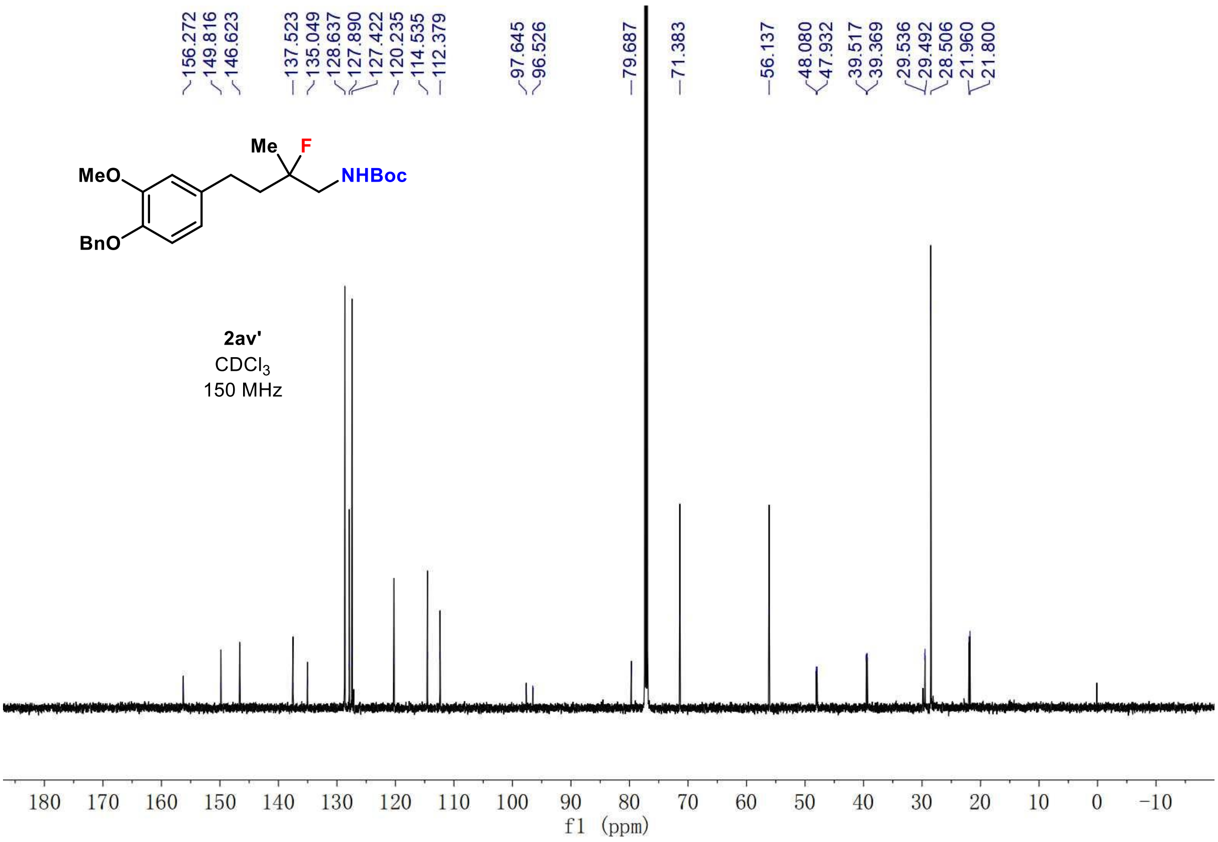

Supplementary Fig. 183. ^{13}**C NMR** Spectra of **2av'**

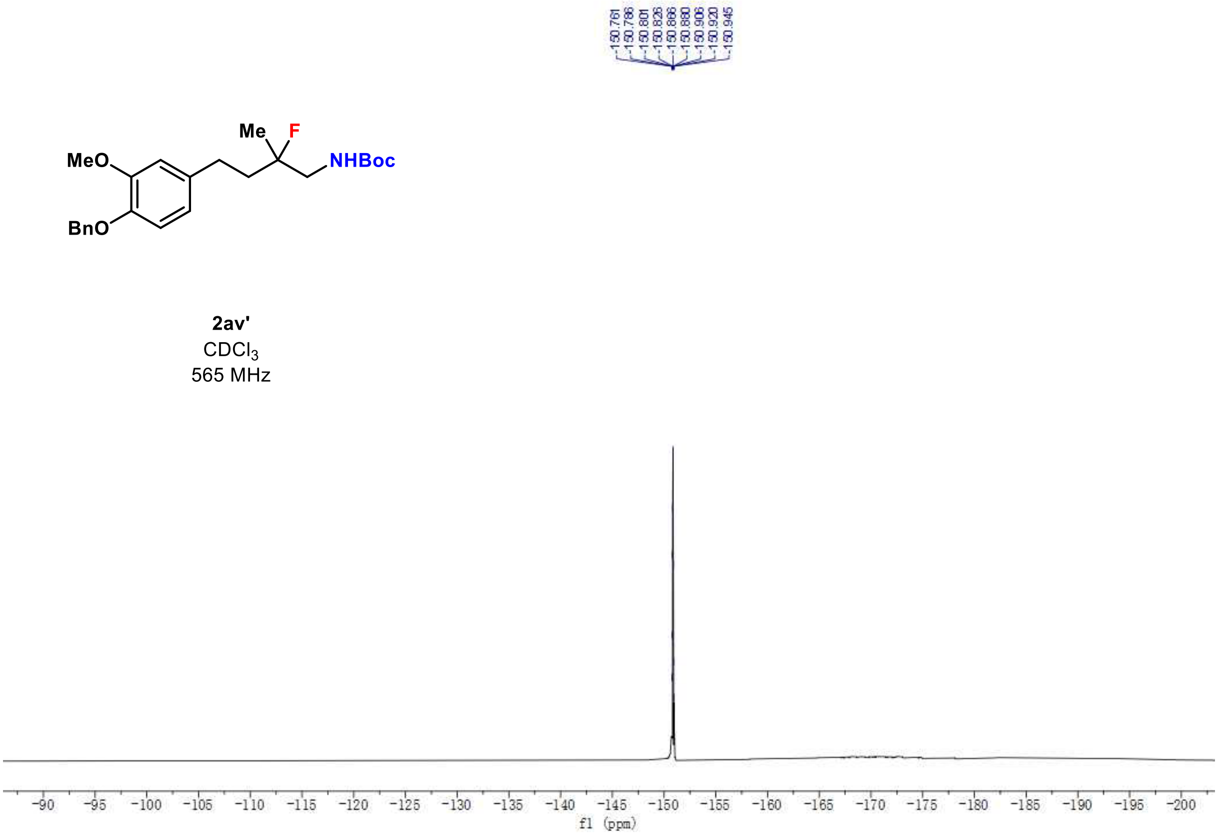

Supplementary Fig. 184. ^{19}**F NMR** Spectra of **2av'**

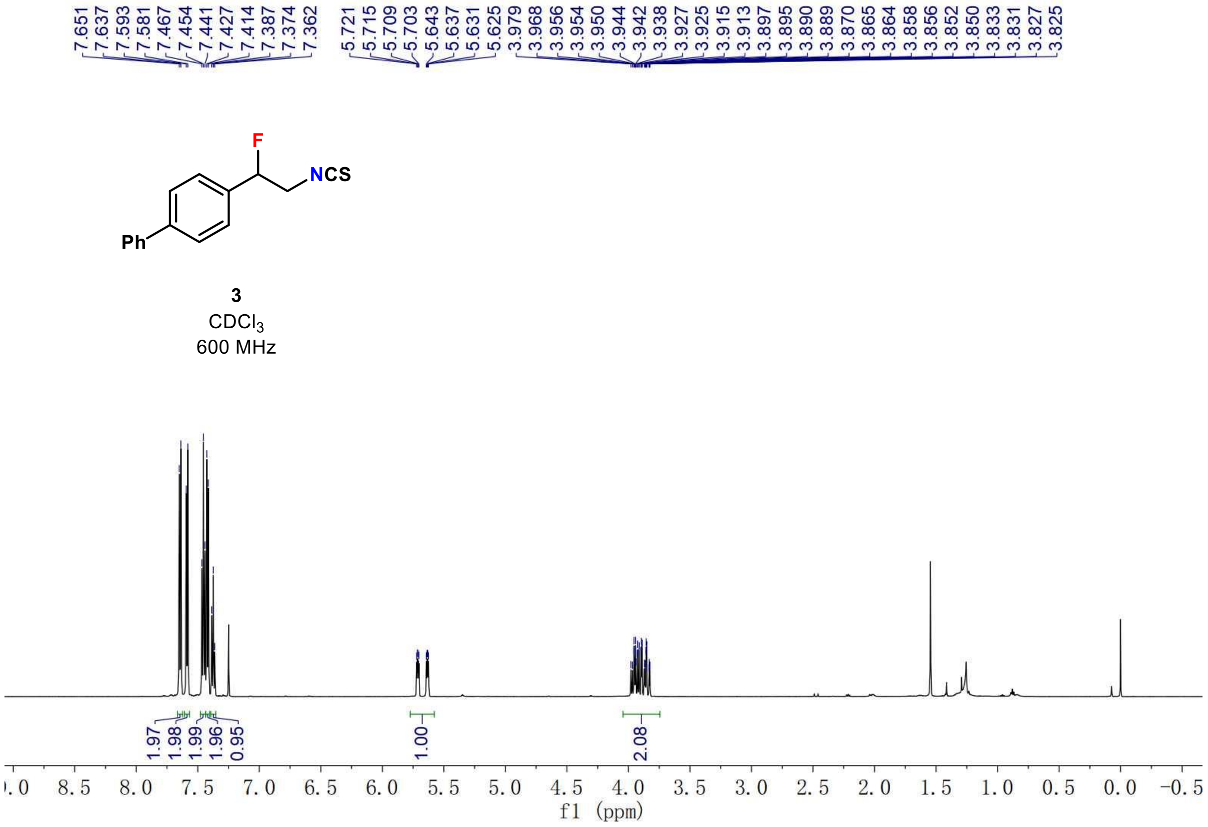

Supplementary Fig. 185. ^{1}H NMR Spectra of **3**

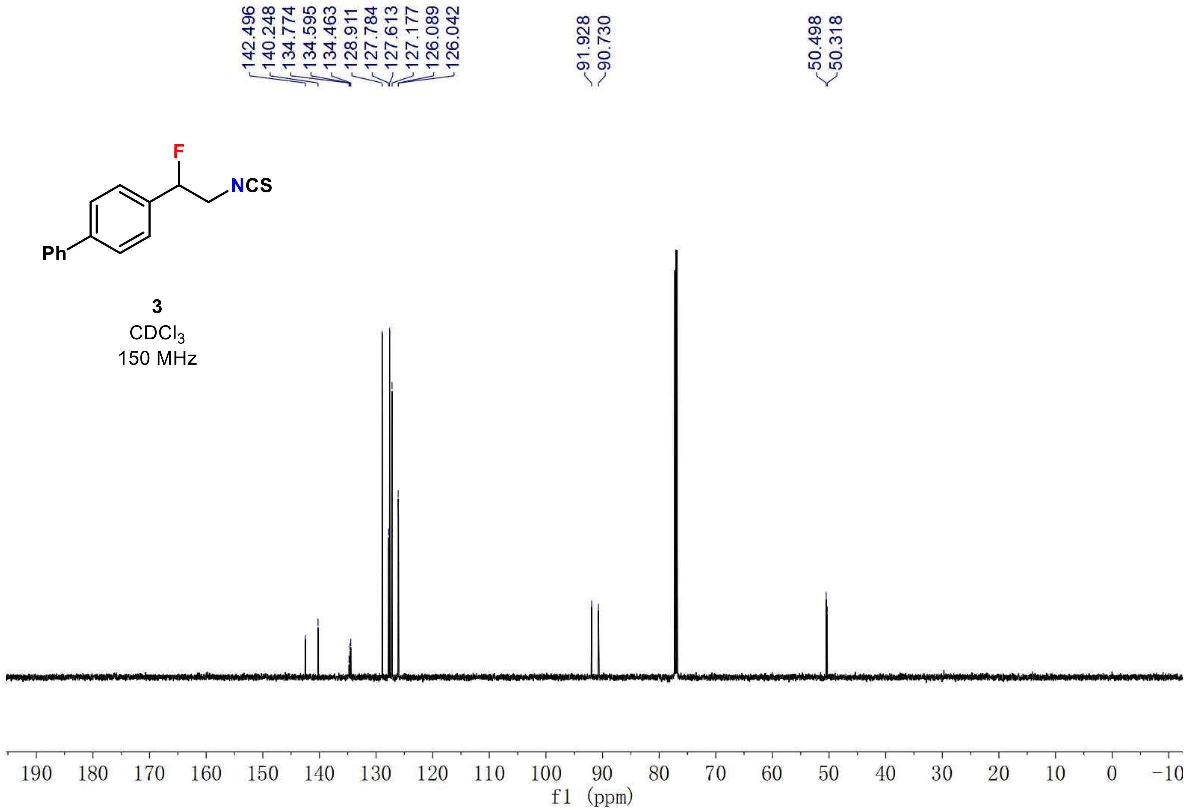

Supplementary Fig. 186. ^{13}C NMR Spectra of **3**

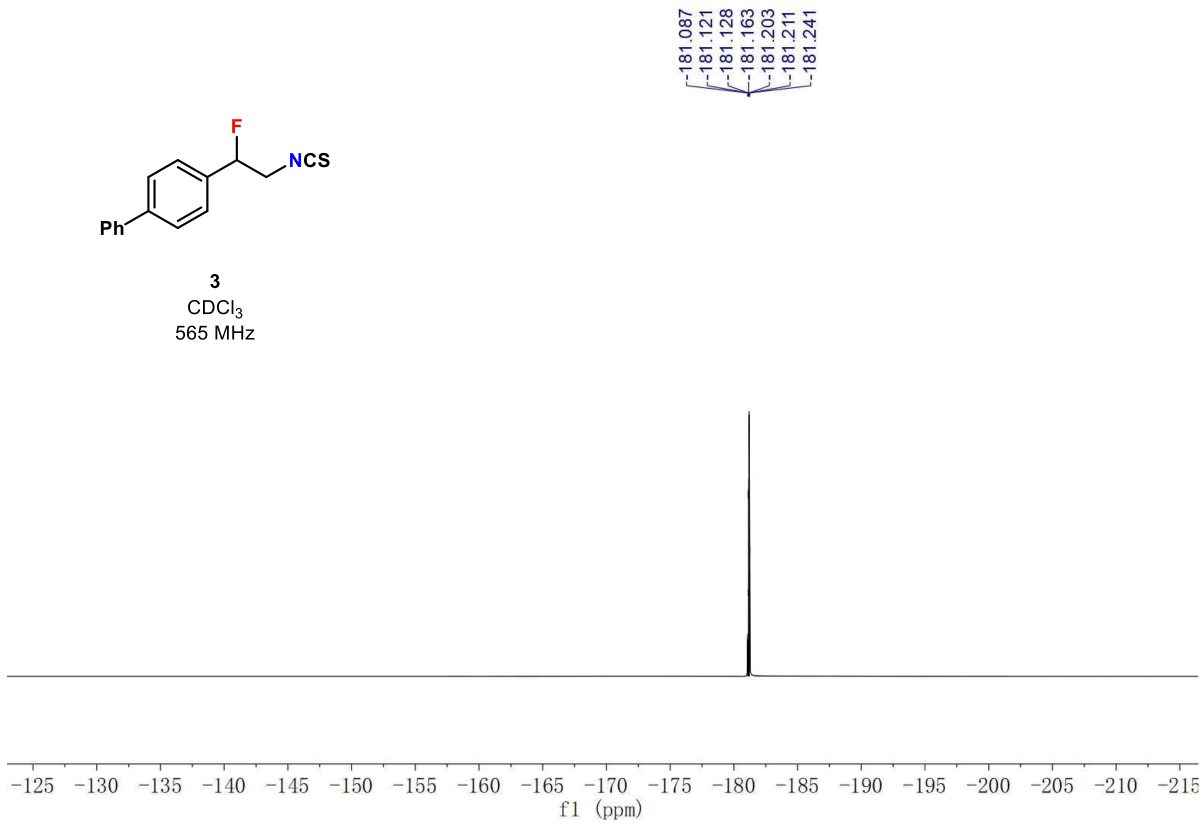

Supplementary Fig. 187. ^{19}**F NMR** Spectra of **3**

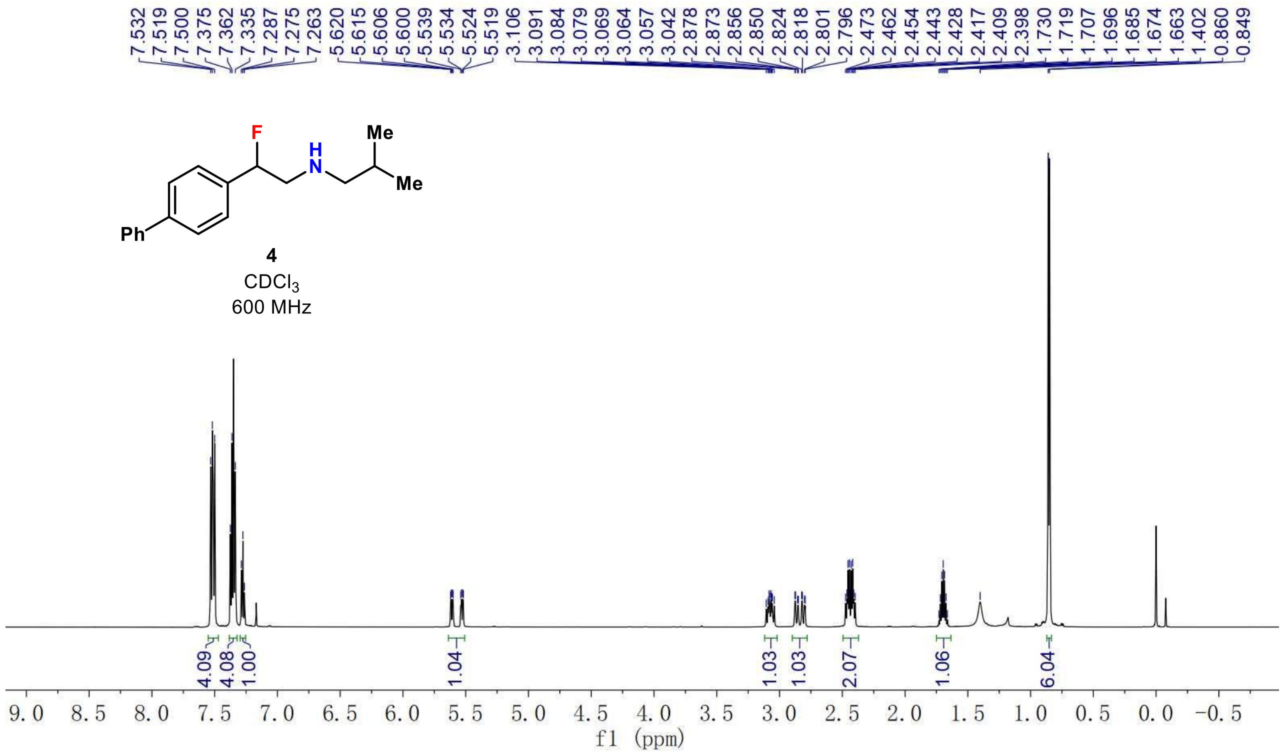

Supplementary Fig. 188. ^{1}**H NMR** Spectra of **4**

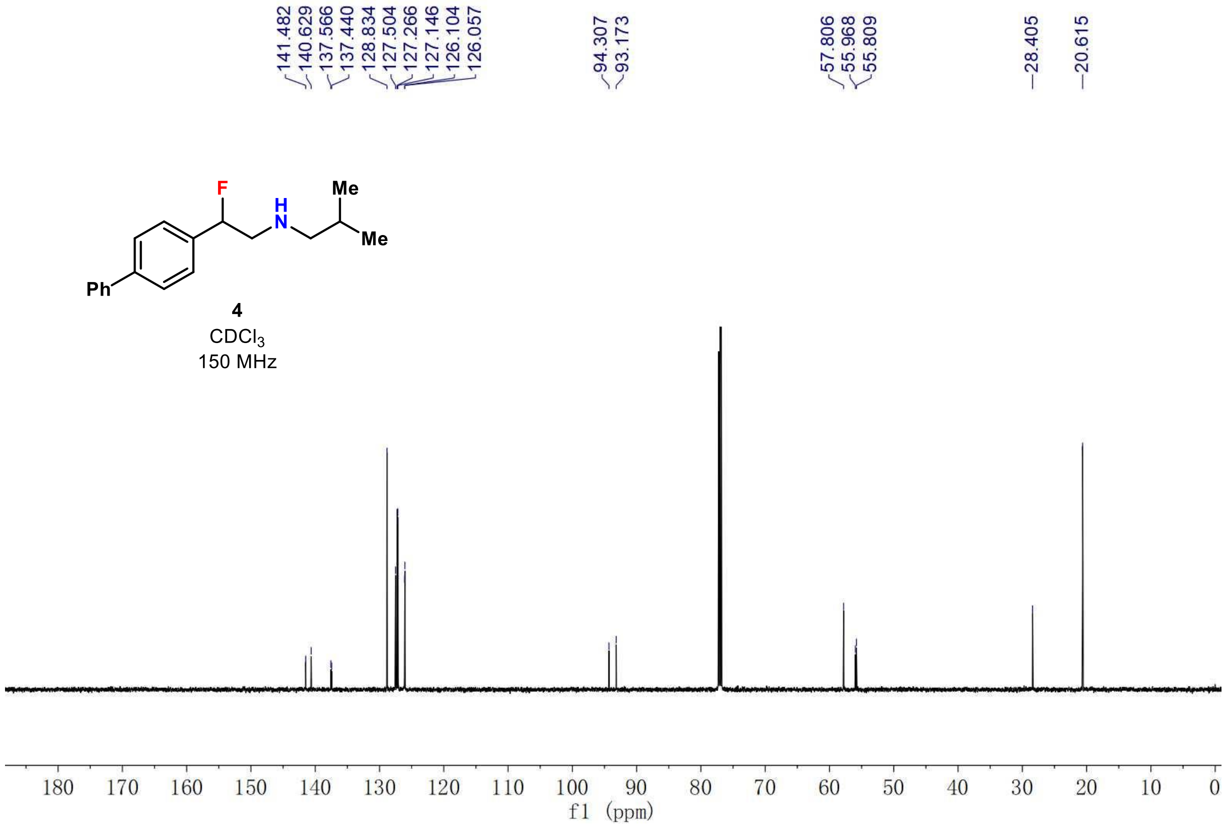

Supplementary Fig. 189. ^{13}**C NMR Spectra of 4**

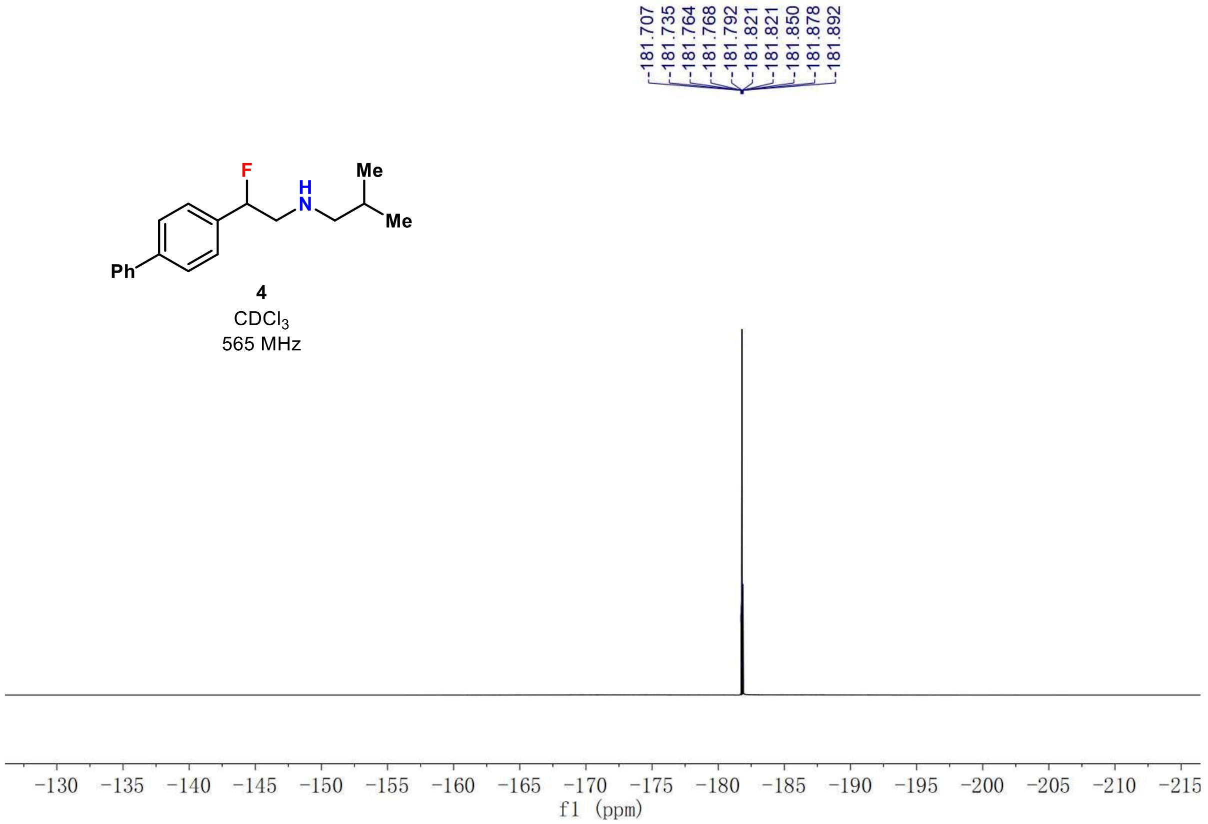

Supplementary Fig. 190. ^{19}**F NMR** Spectra of **4**

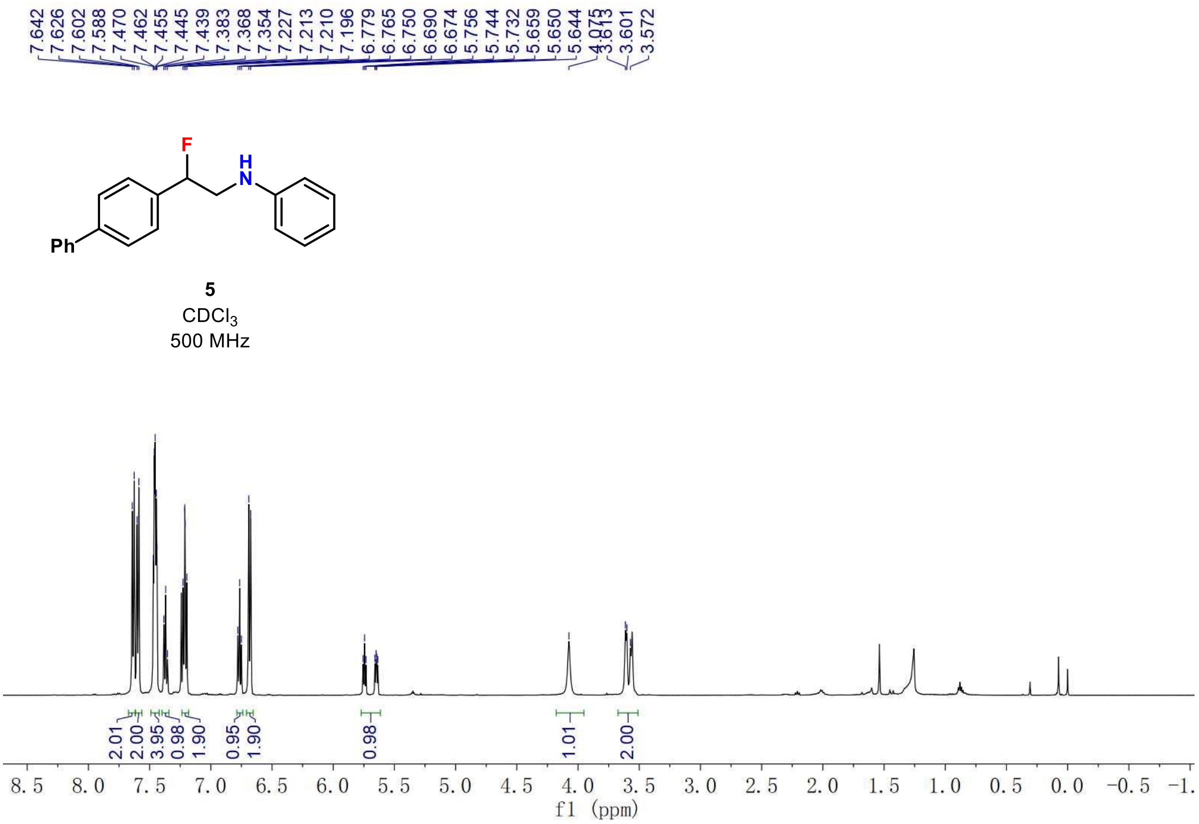

Supplementary Fig. 191. **^{1}H NMR** Spectra of **5**

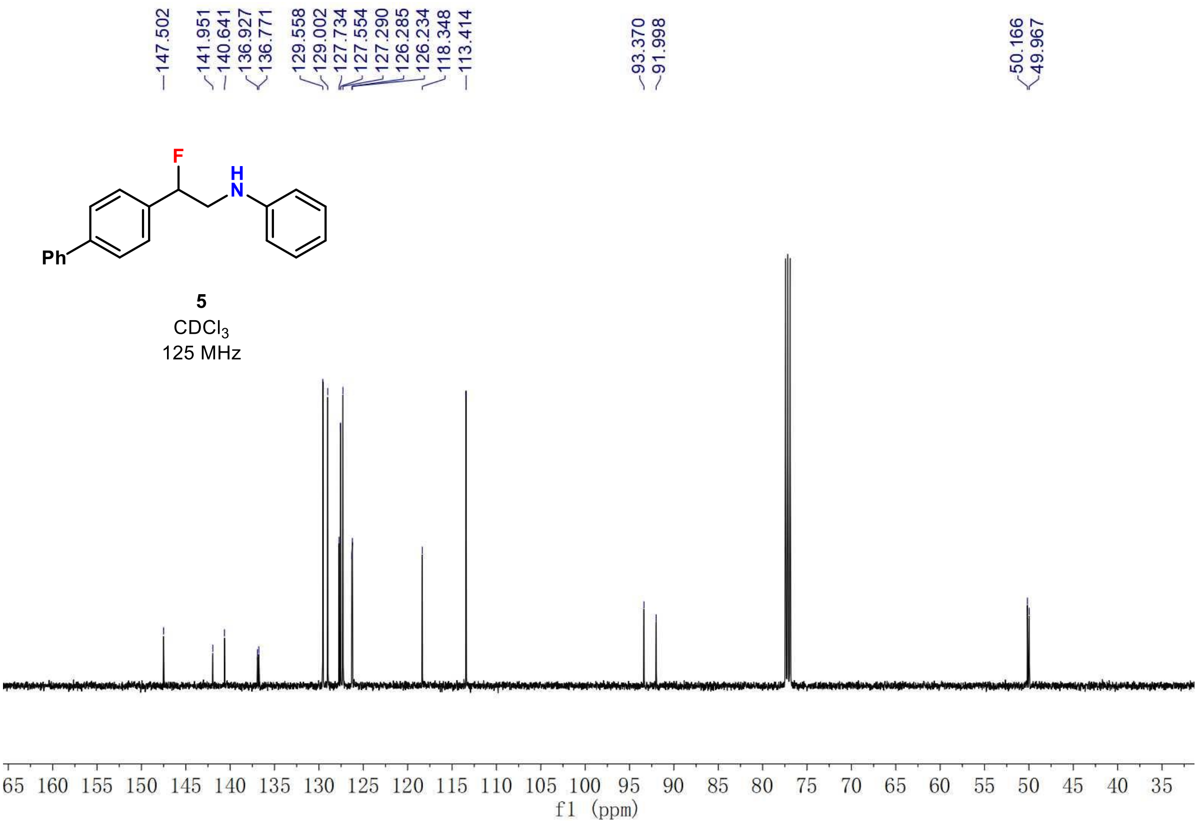

Supplementary Fig. 192. **^{13}C NMR** Spectra of **5**

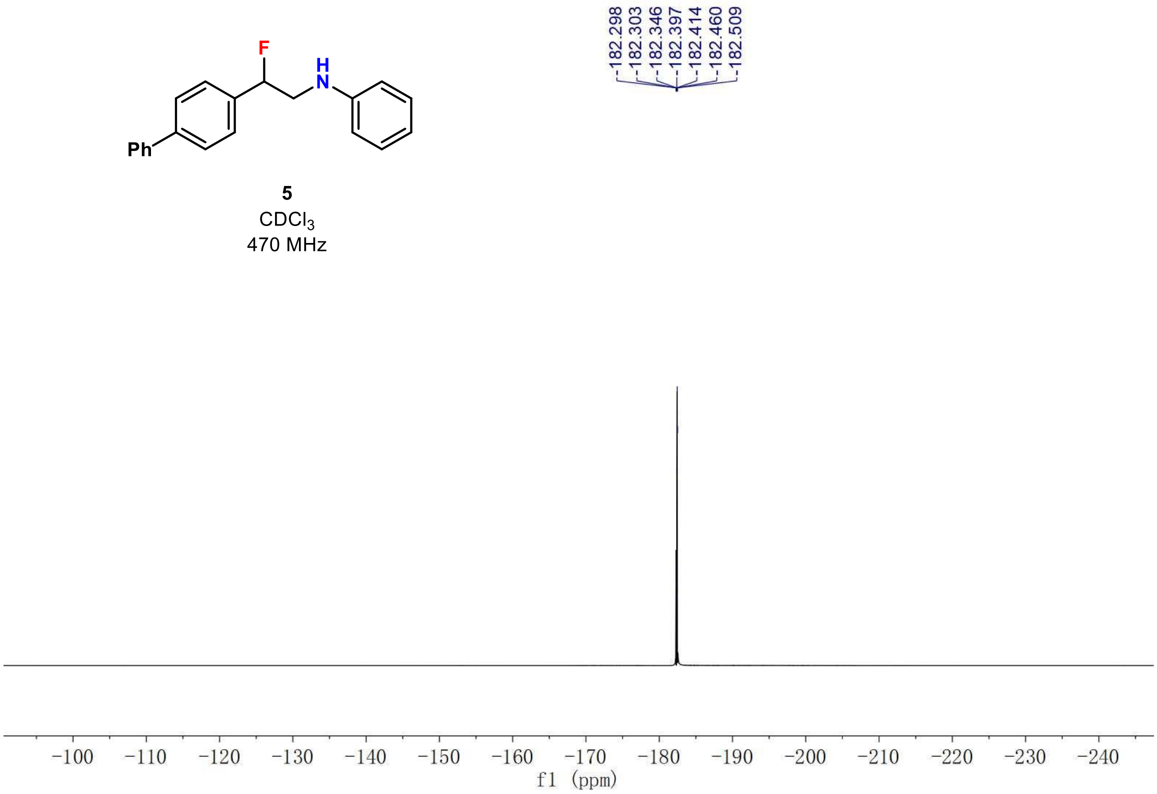

Supplementary Fig. 193. ^{19}**F NMR** Spectra of **5**

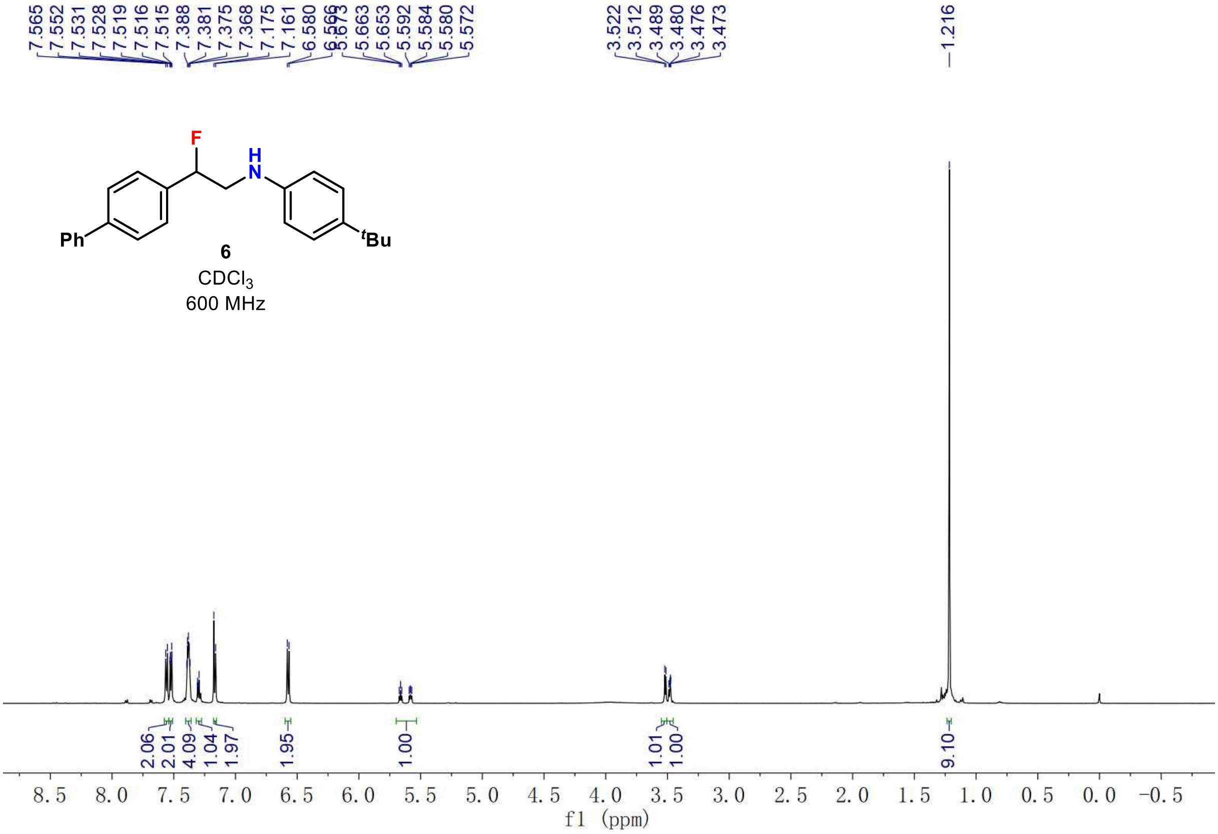

Supplementary Fig. 194. ^{1}**H NMR** Spectra of **6**

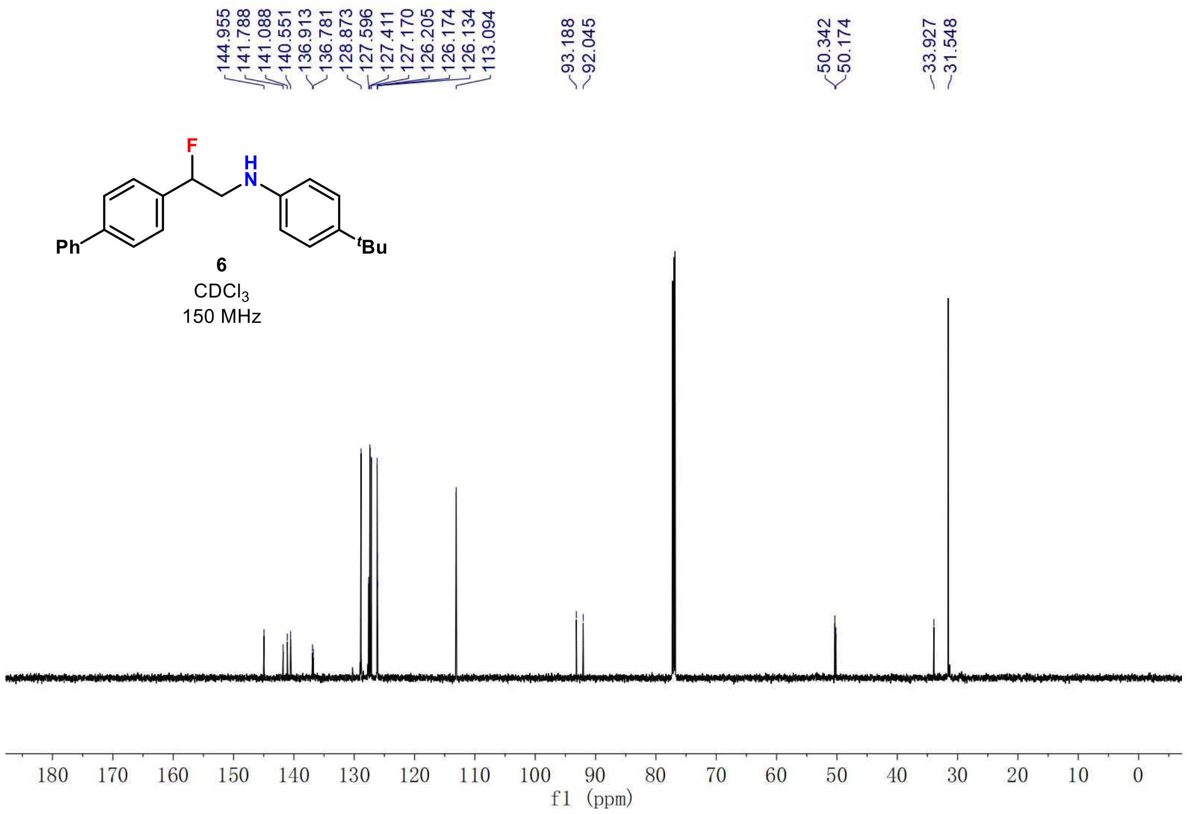

Supplementary Fig. 195. ^{13}**C NMR** Spectra of **6**

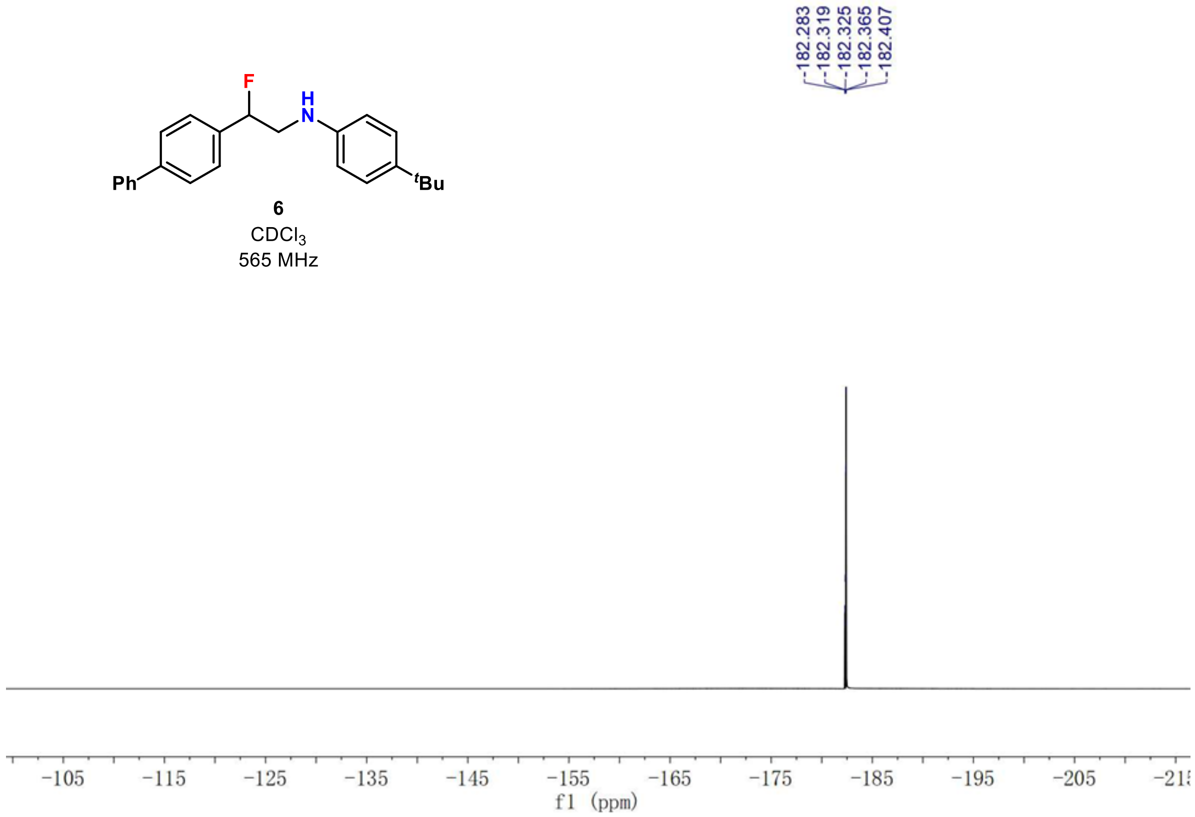

Supplementary Fig. 196. ^{19}**F NMR** Spectra of **6**

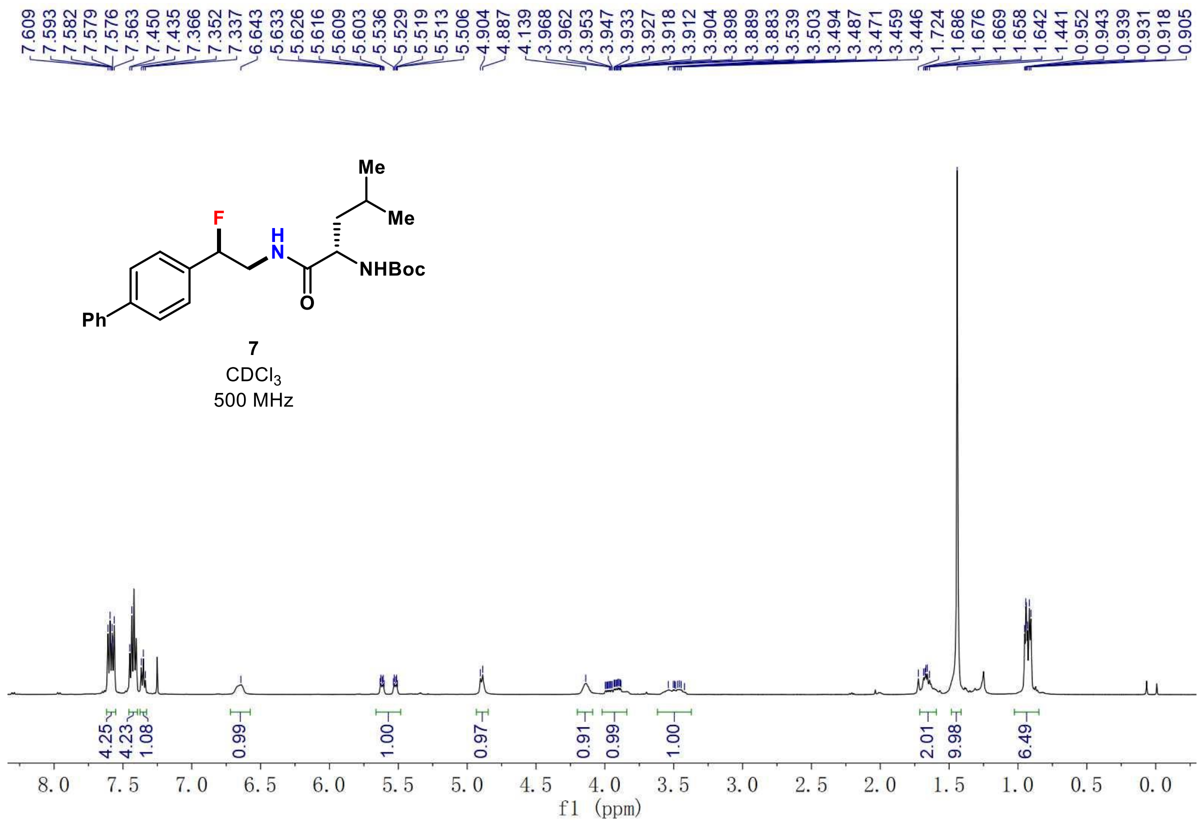

Supplementary Fig.197. ^{1}H NMR Spectra of **7**

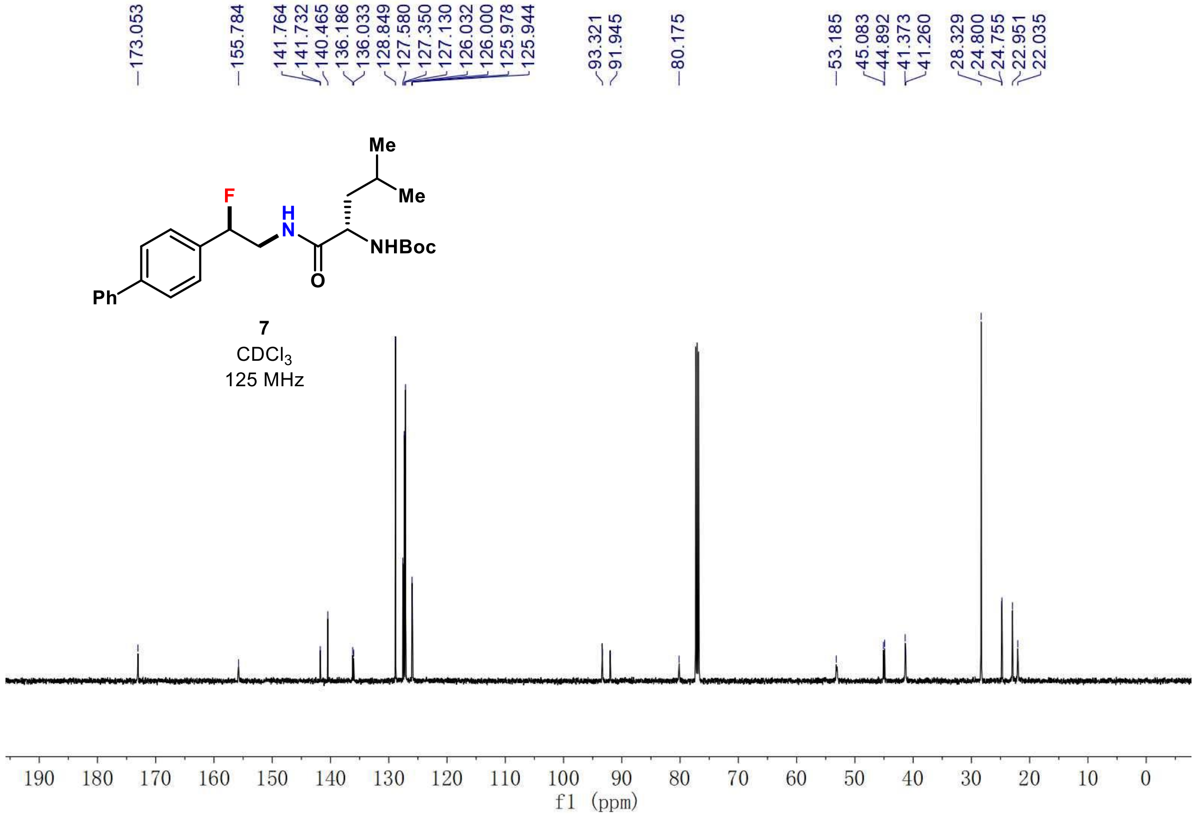

Supplementary Fig. 198. ^{13}C NMR Spectra of **7**

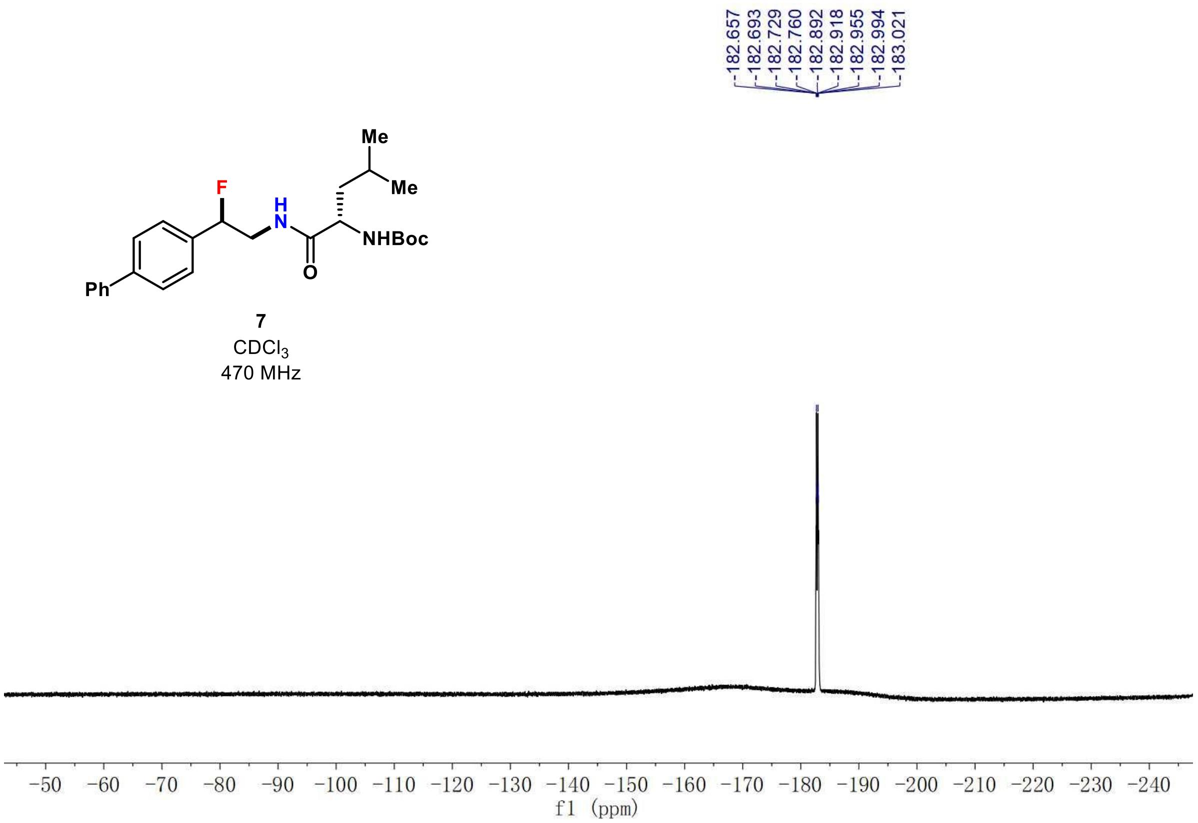

Supplementary Fig. 199. ^{19}**F NMR** Spectra of **7**

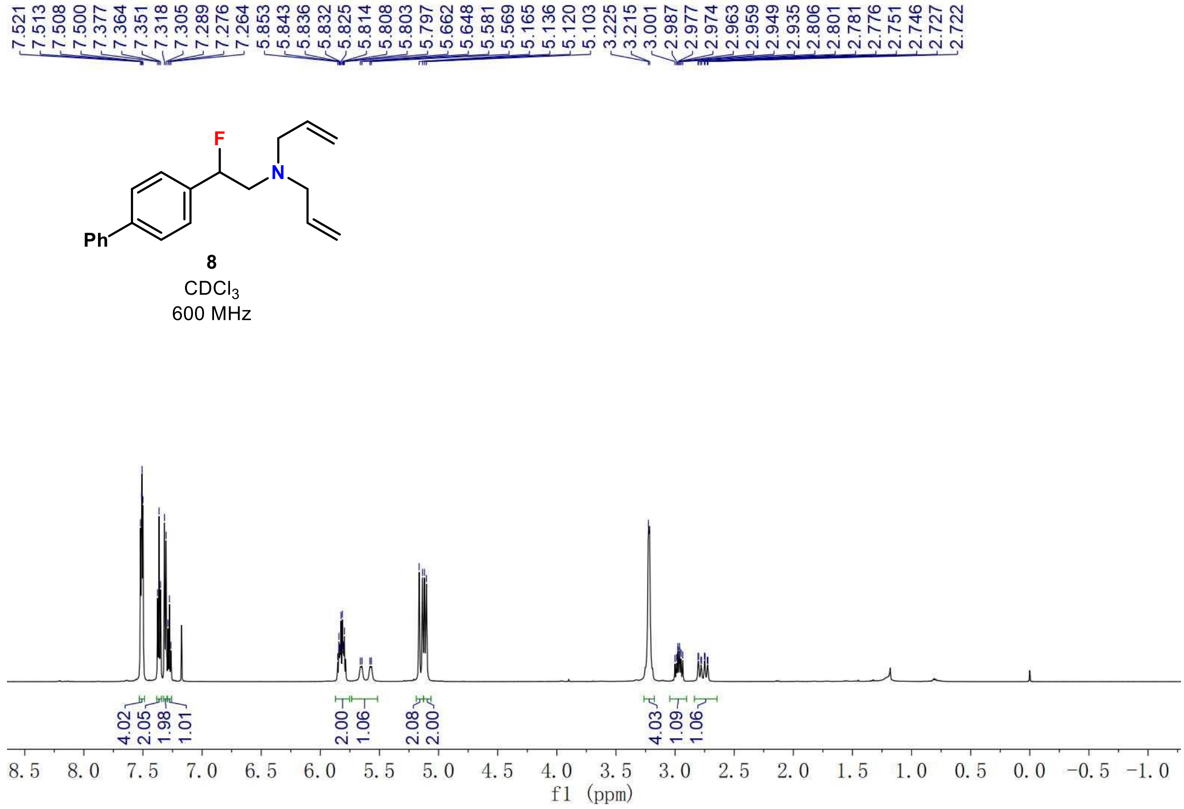

Supplementary Fig. 200. ^{1}**H NMR** Spectra of **8**

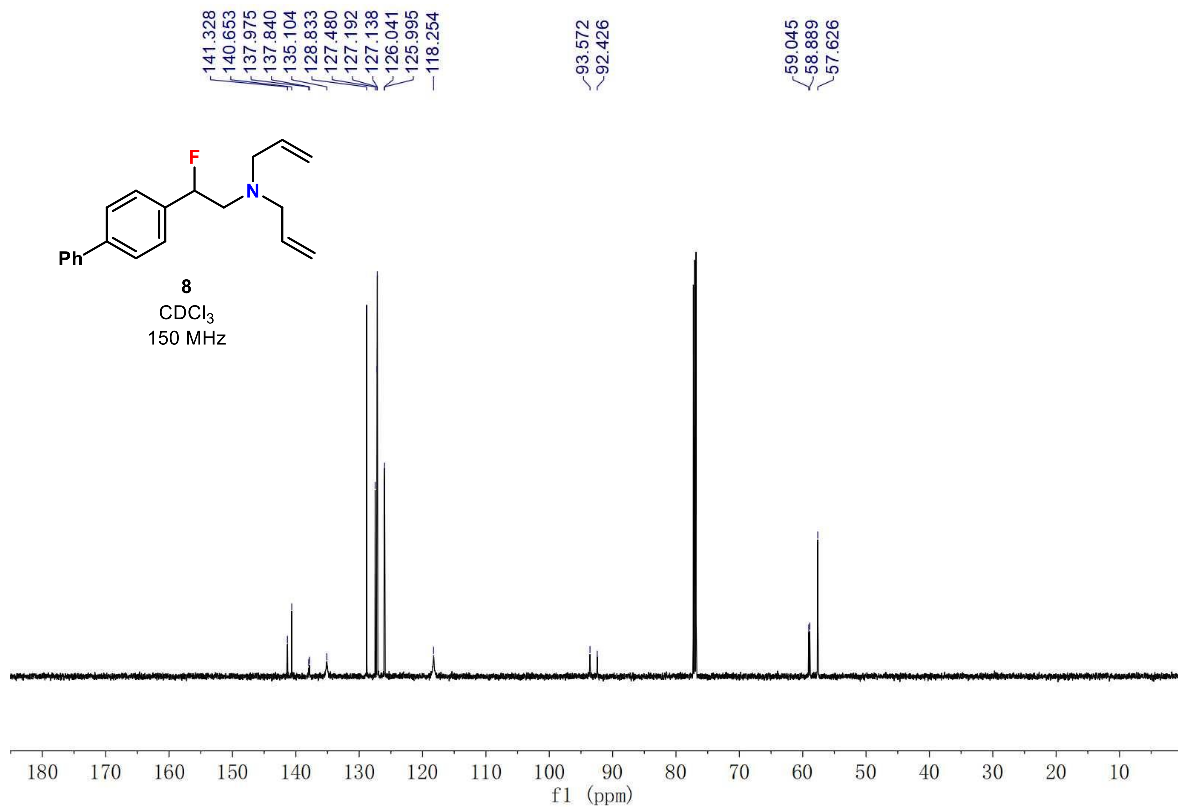

Supplementary Fig. 201. ^{13}**C NMR** Spectra of **8**

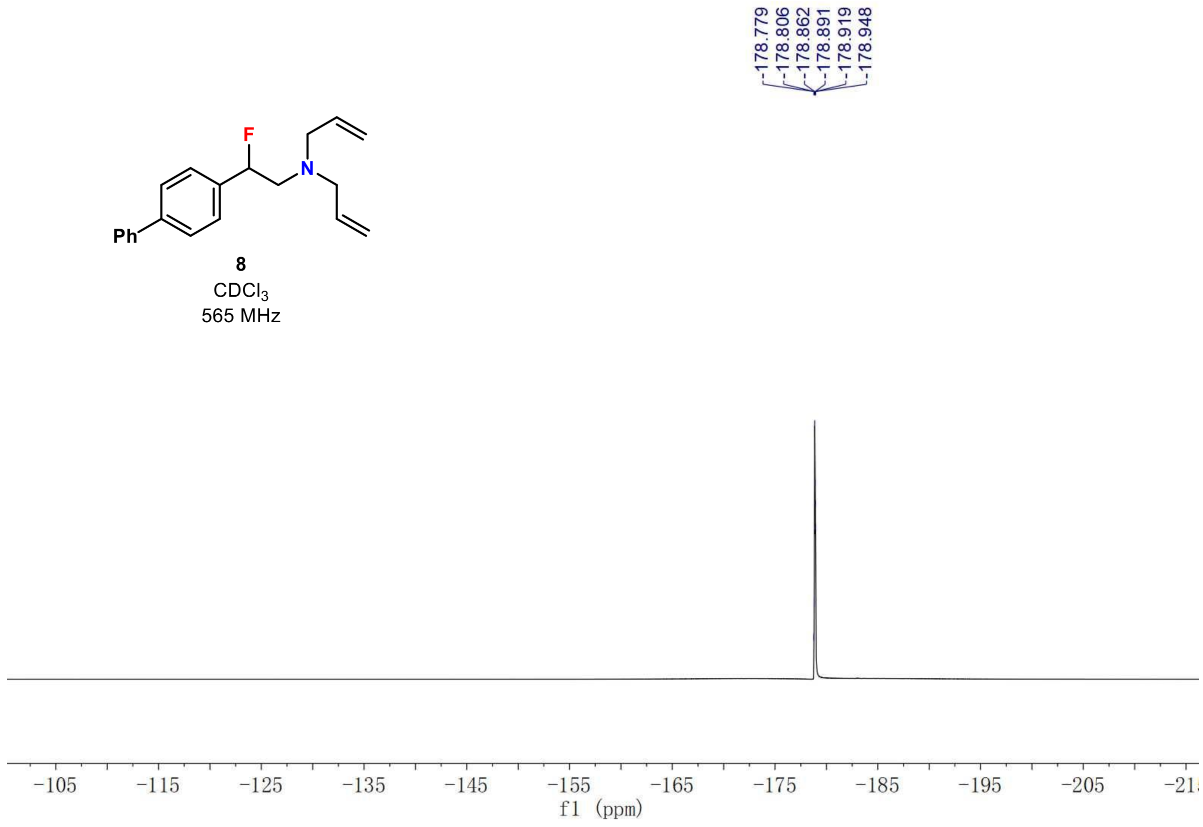

Supplementary Fig. 202. ^{19}**F NMR** Spectra of **8**

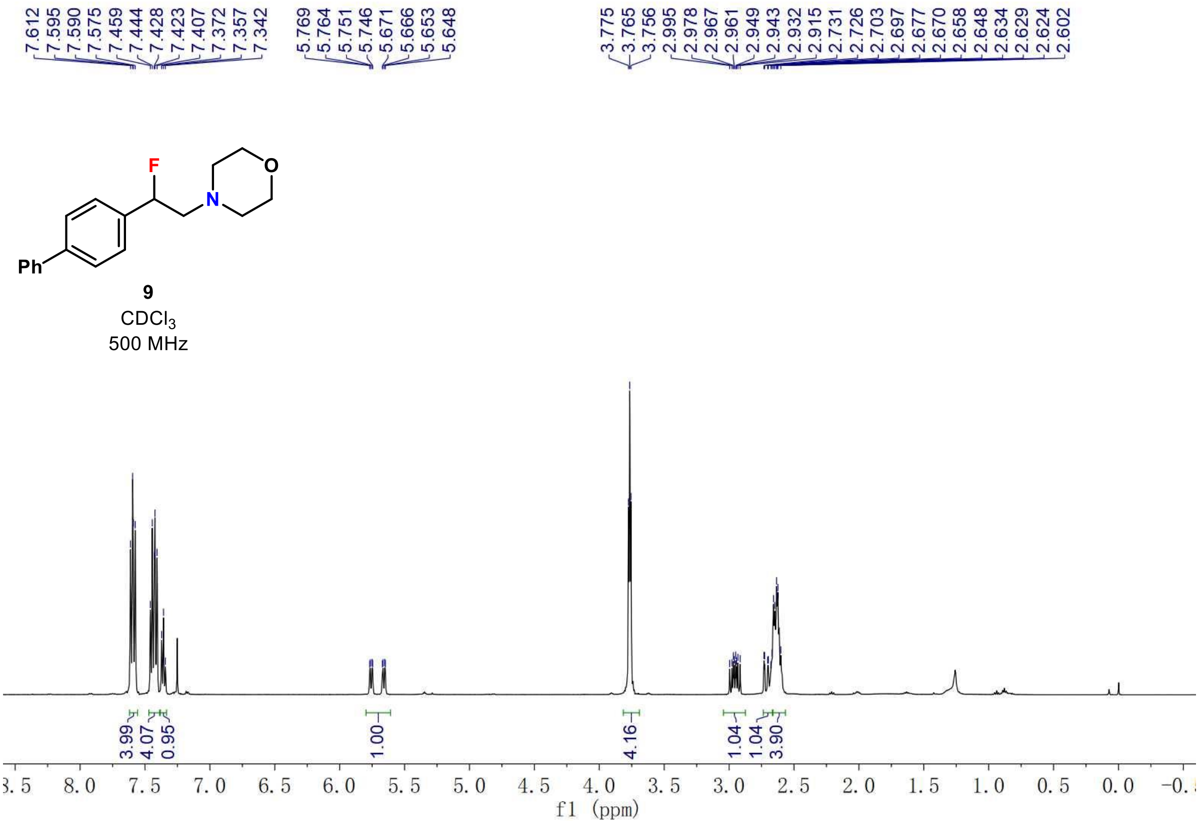

Supplementary Fig. 203. ^{1}**H NMR** Spectra of **9**

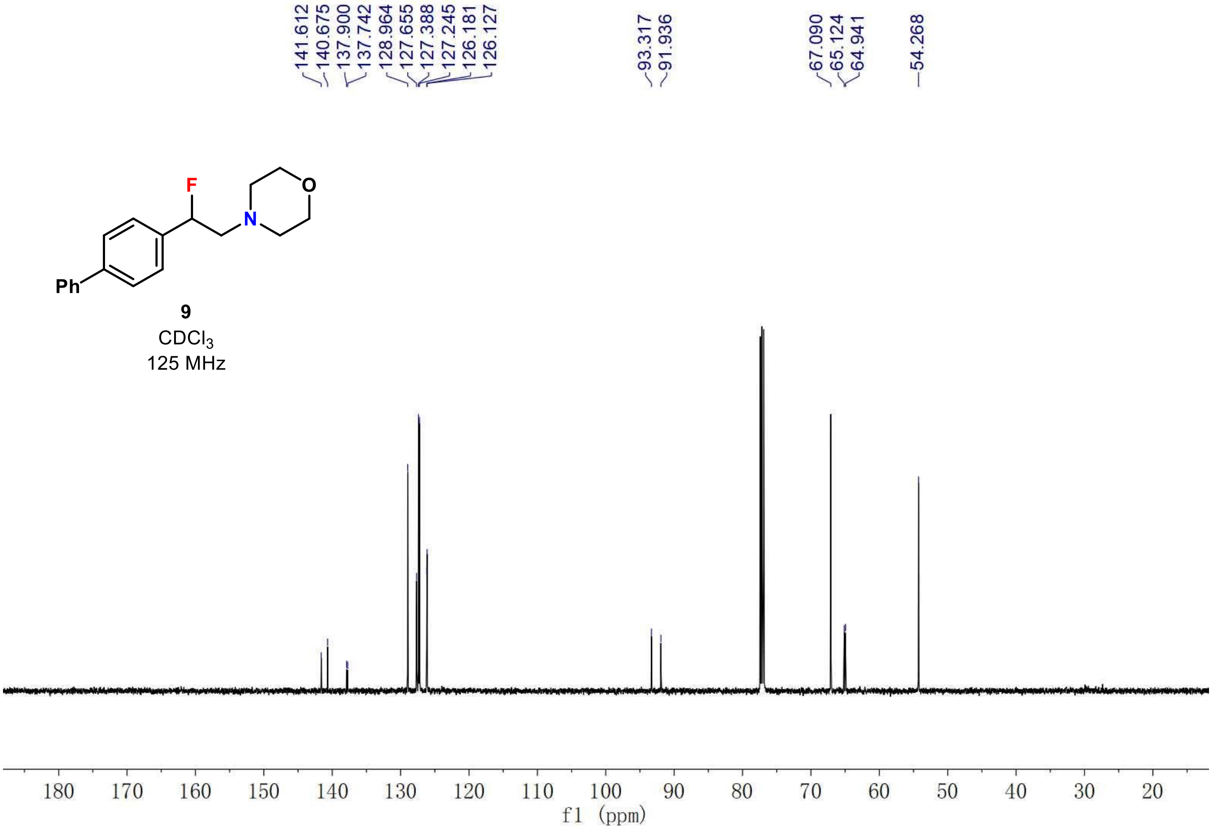

Supplementary Fig. 204. ^{13}**C NMR** Spectra of **9**

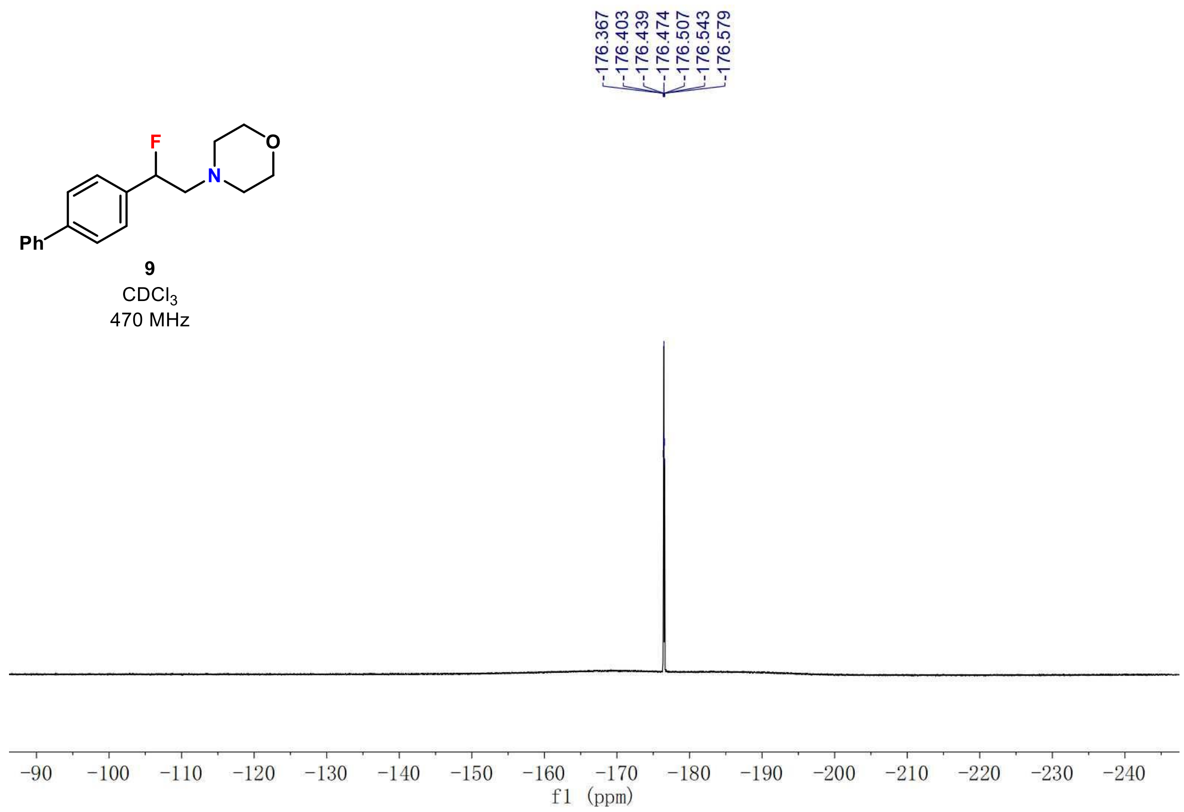

Supplementary Fig. 205. ^{19}**F NMR** Spectra of **9**

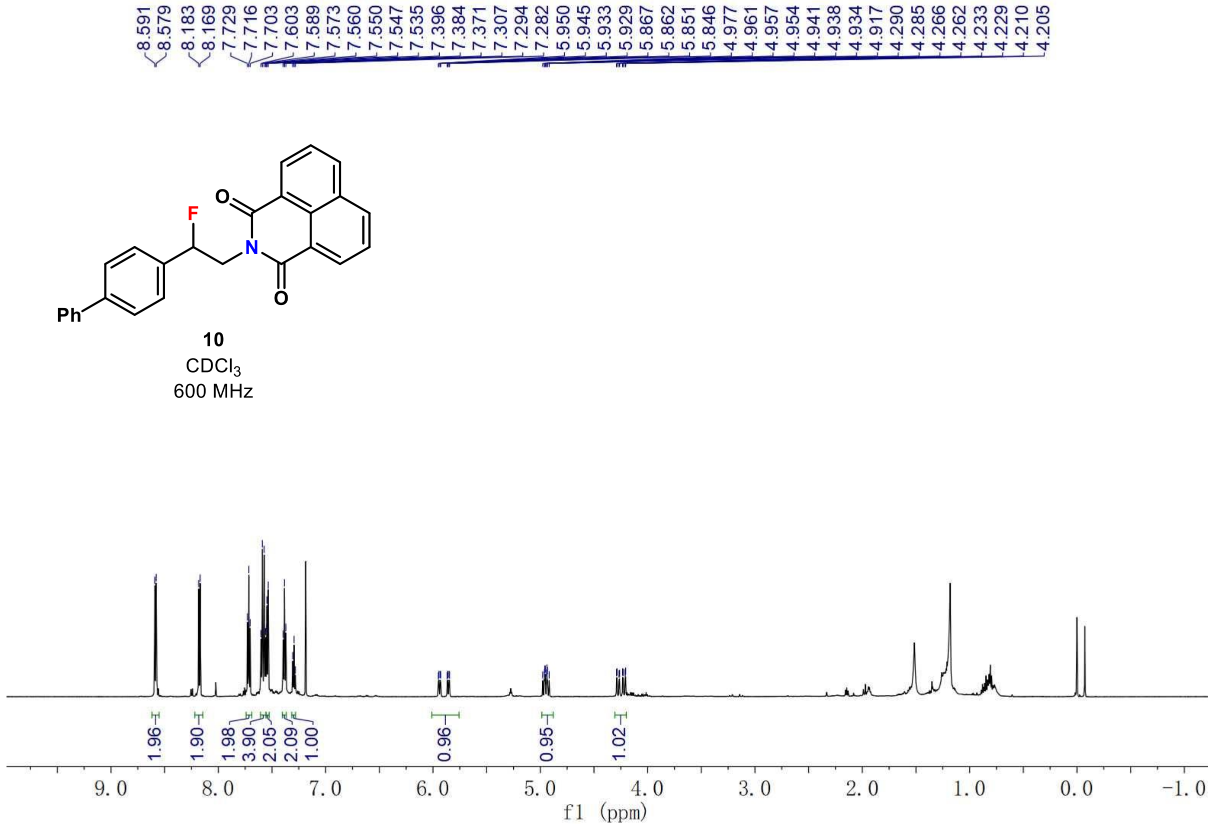

Supplementary Fig. 206. ^{1}**H NMR** Spectra of **10**

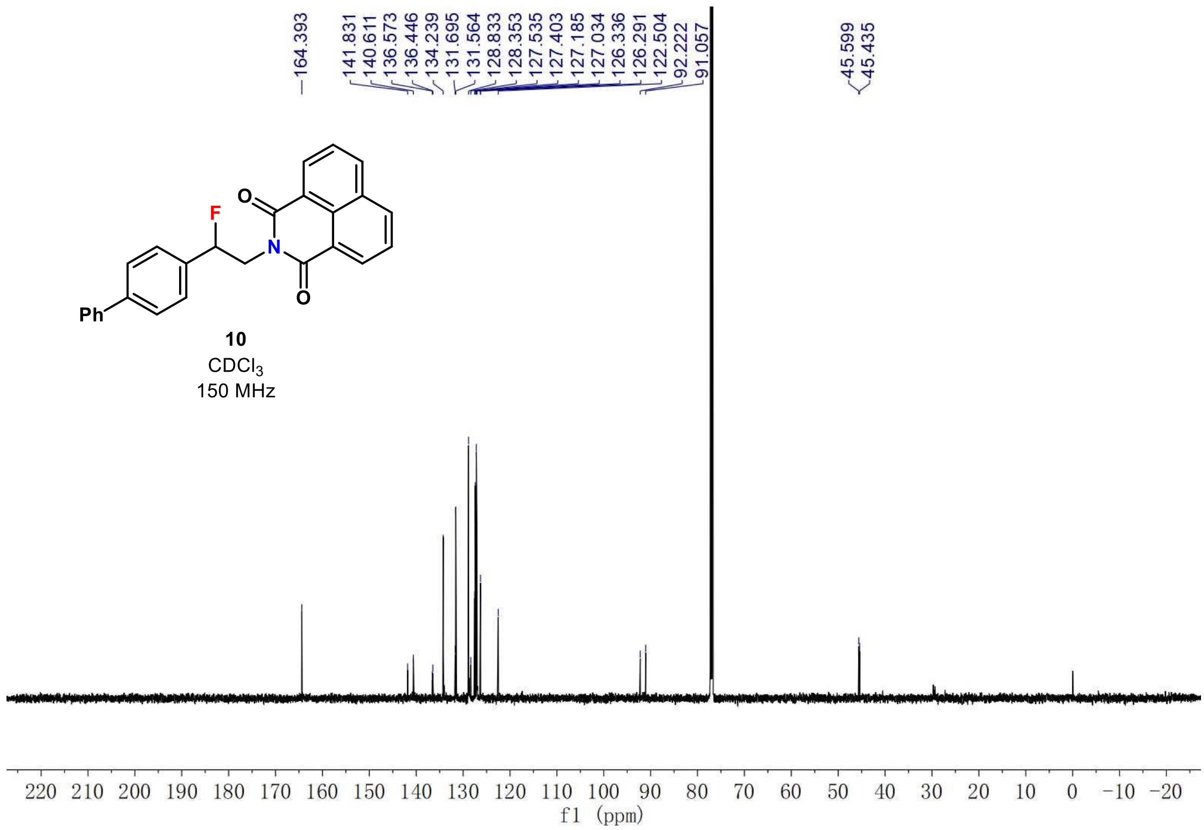

Supplementary Fig. 207. ^{13}**C NMR** Spectra of **10**

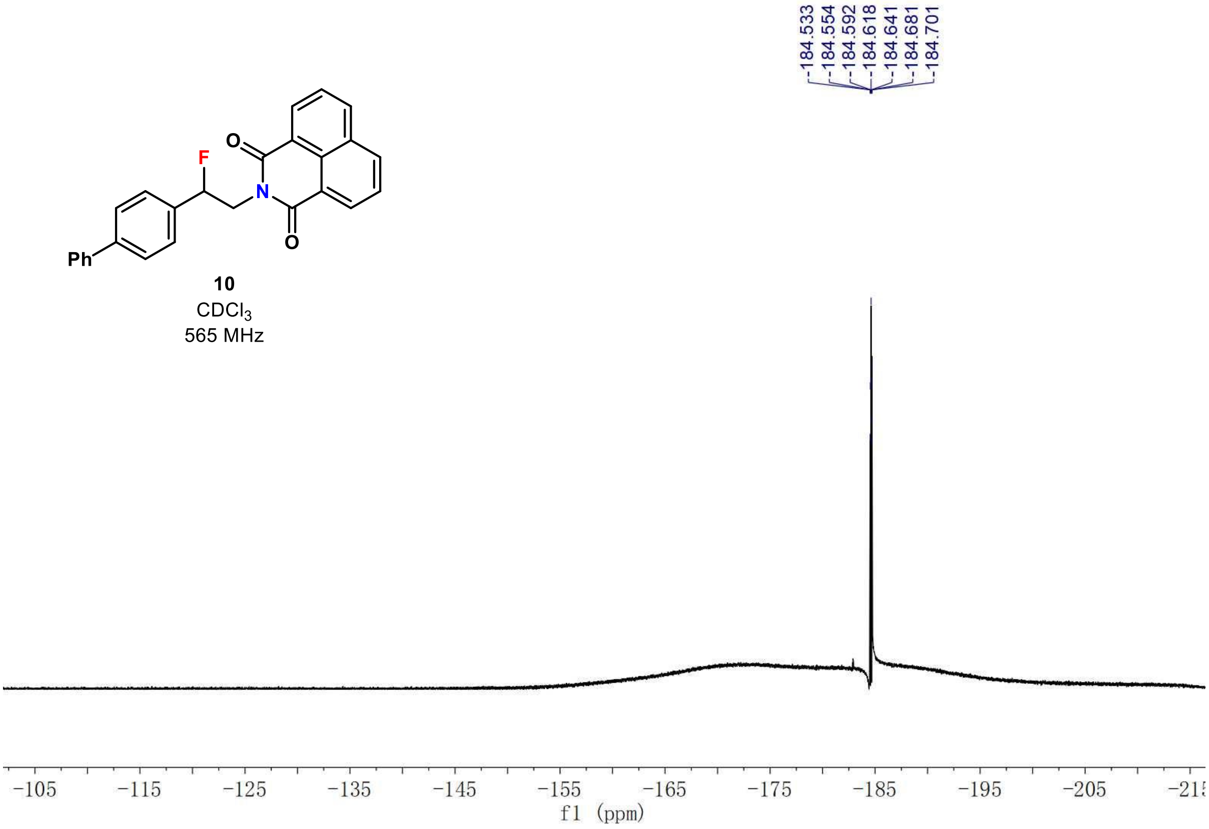

Supplementary Fig. 208. ^{19}**F NMR** Spectra of **10**

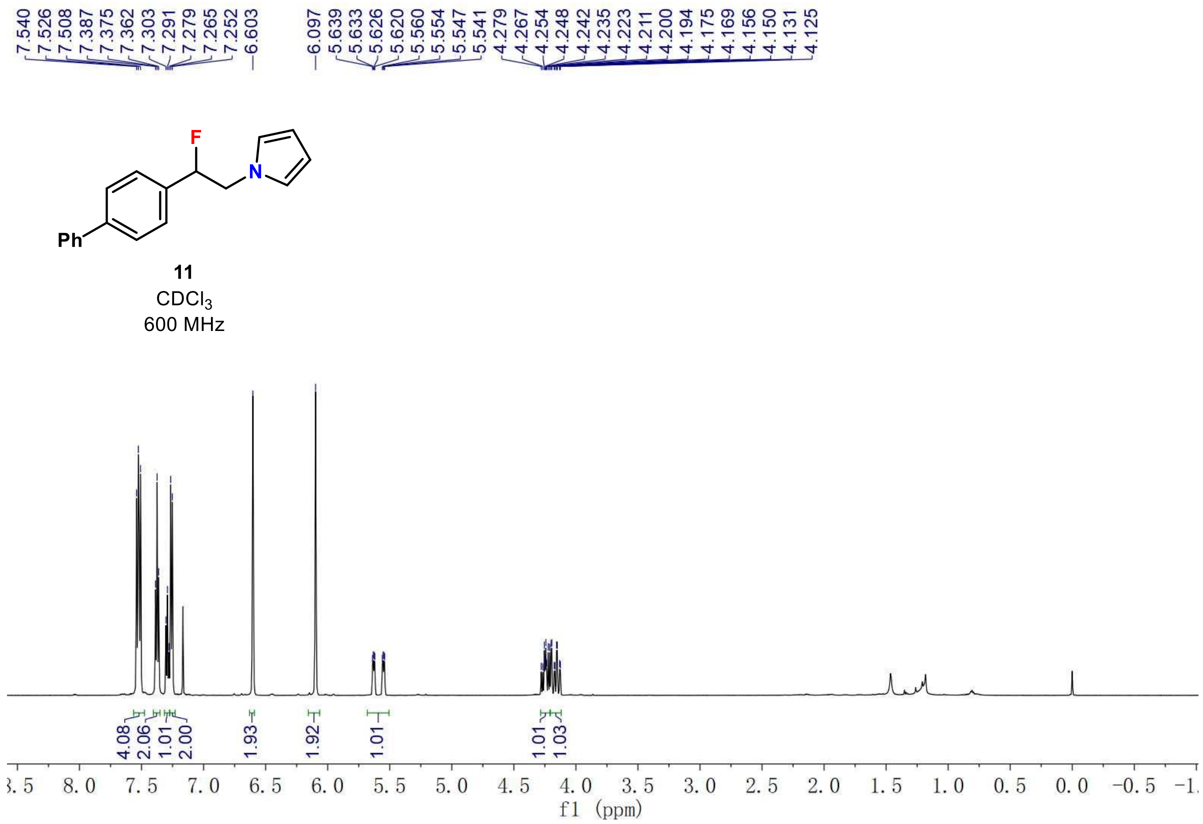

Supplementary Fig. 209. **^{1}H NMR** Spectra of **11**

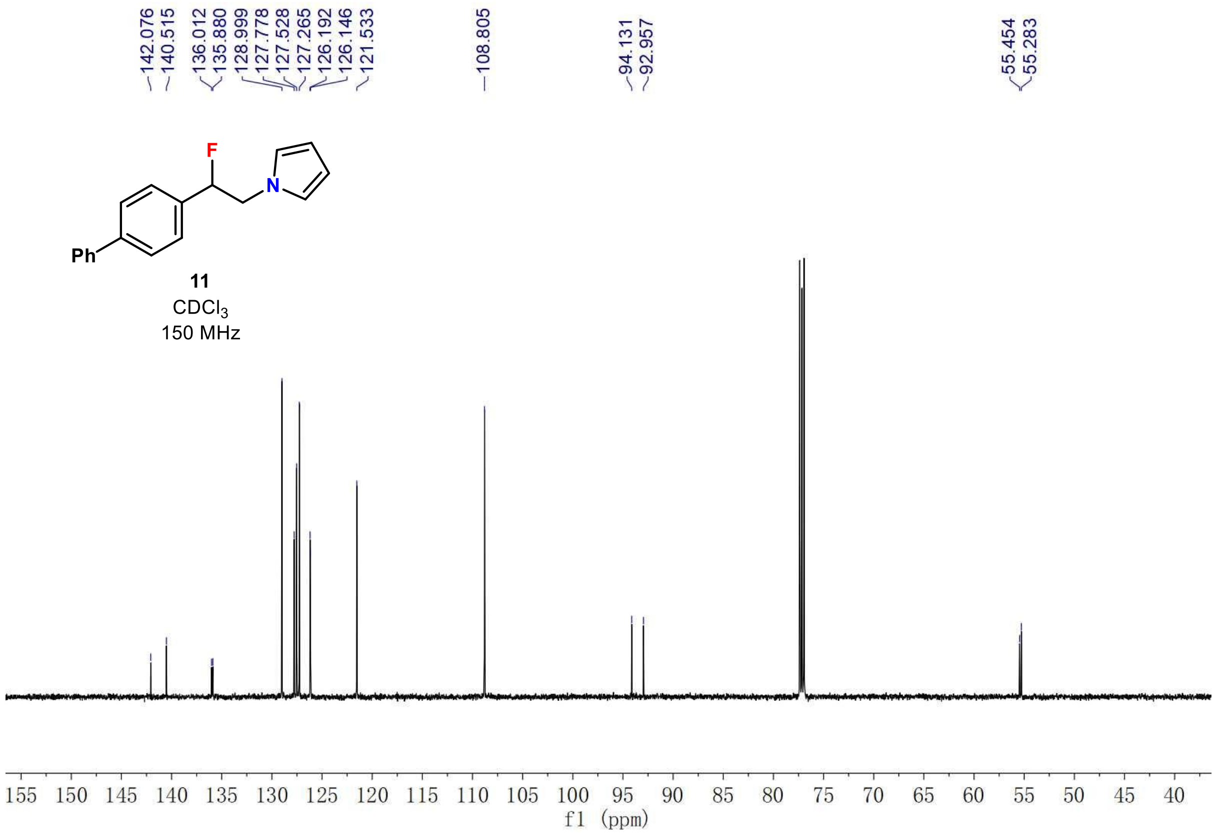

Supplementary Fig. 210. **^{13}C NMR** Spectra of **11**

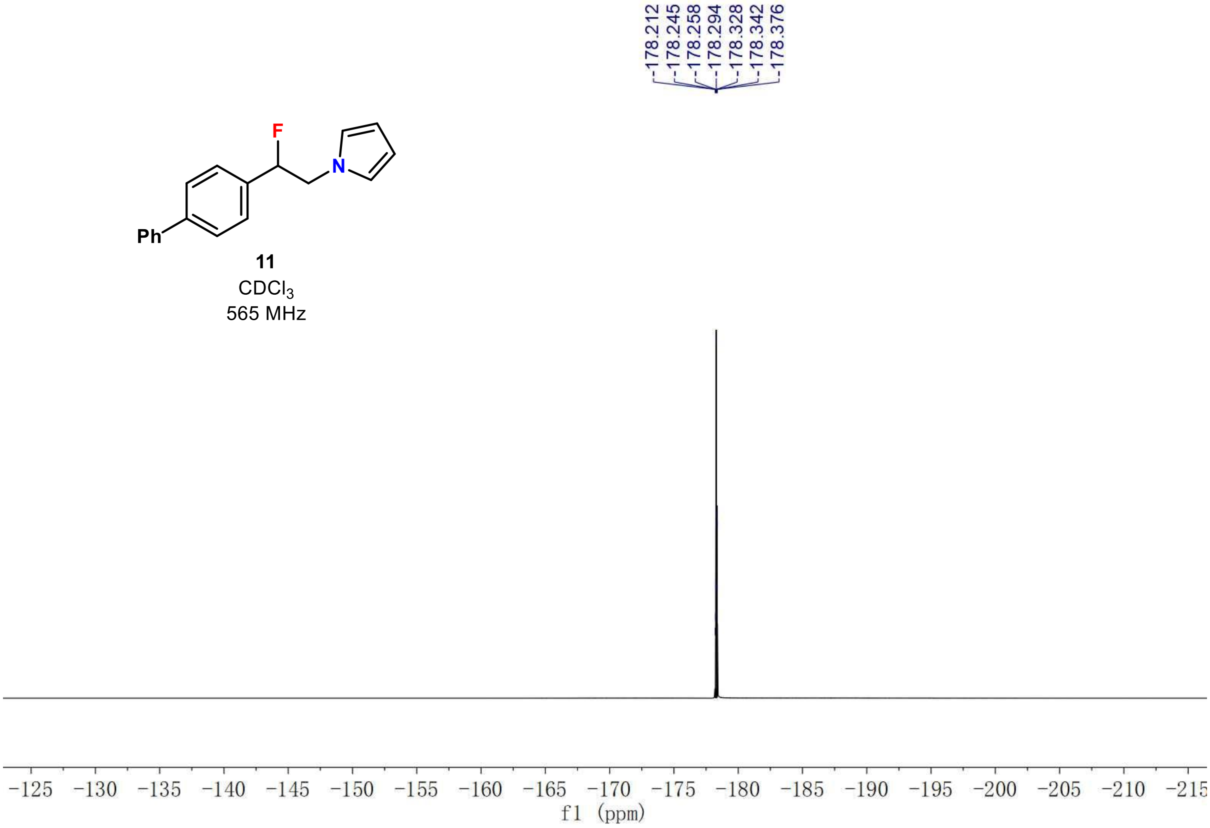

Supplementary Fig. 211. ^{19}**F NMR** Spectra of **11**

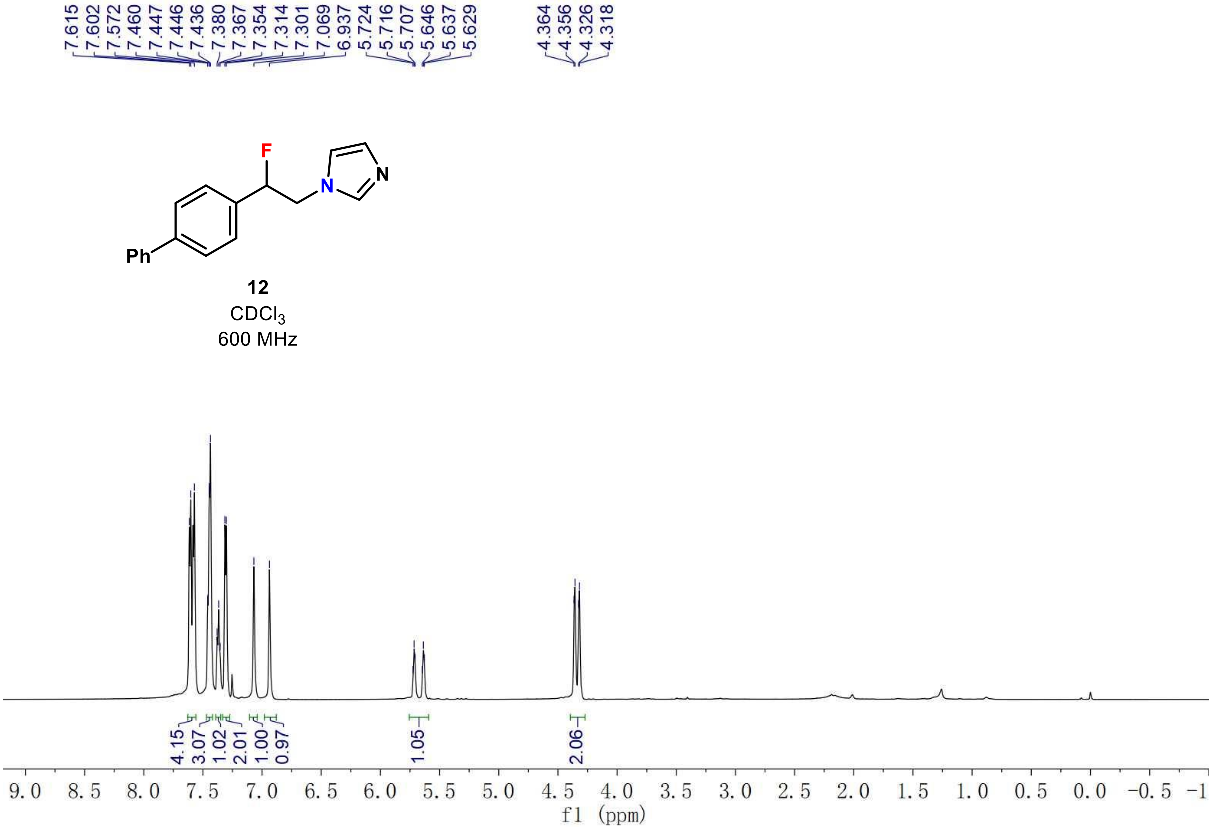

Supplementary Fig. 212. ^{1}**H NMR** Spectra of **12**

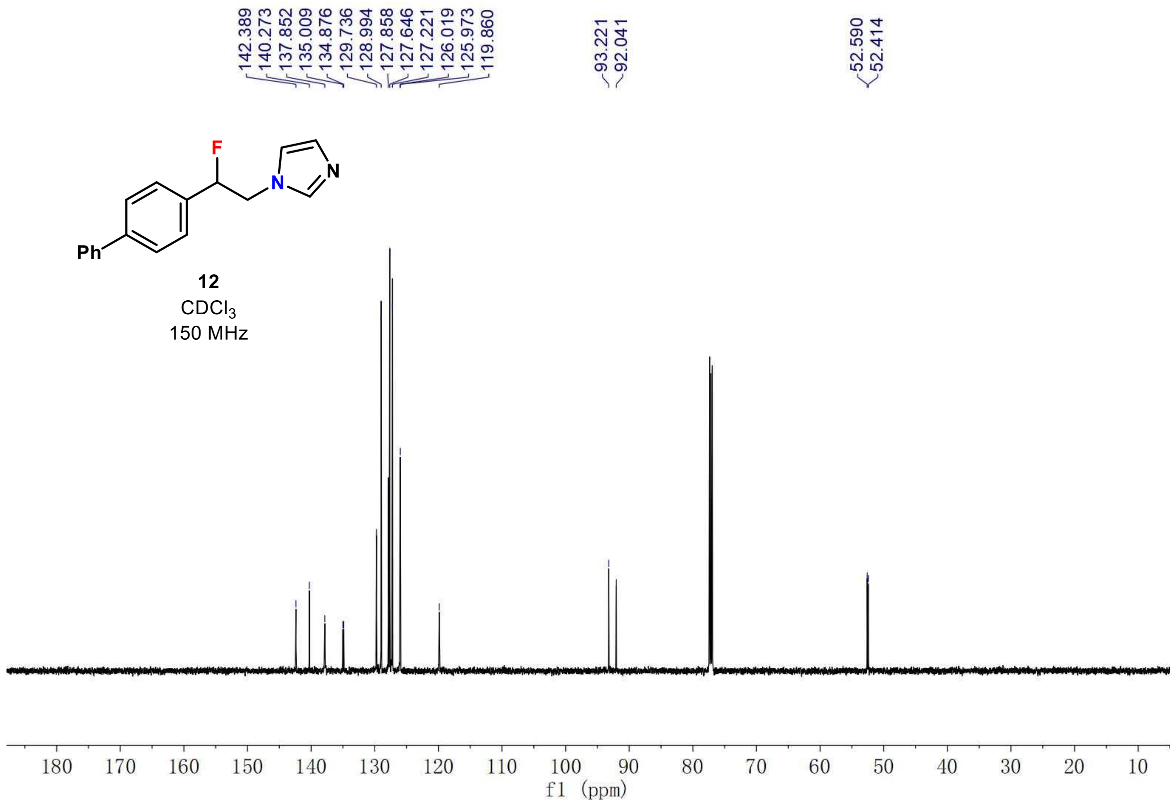

Supplementary Fig. 213. ^{13}**C NMR** Spectra of **12**

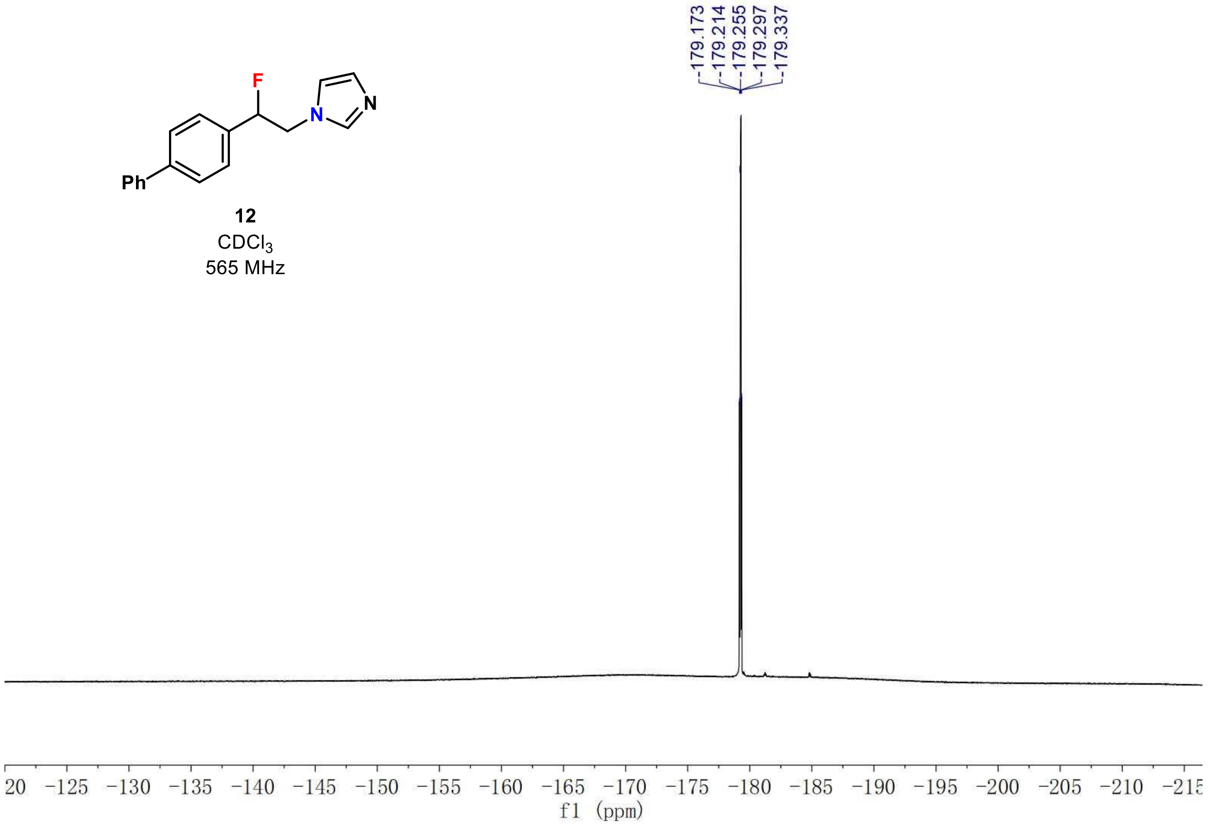

Supplementary Fig. 214. ^{19}**F NMR** Spectra of **12**

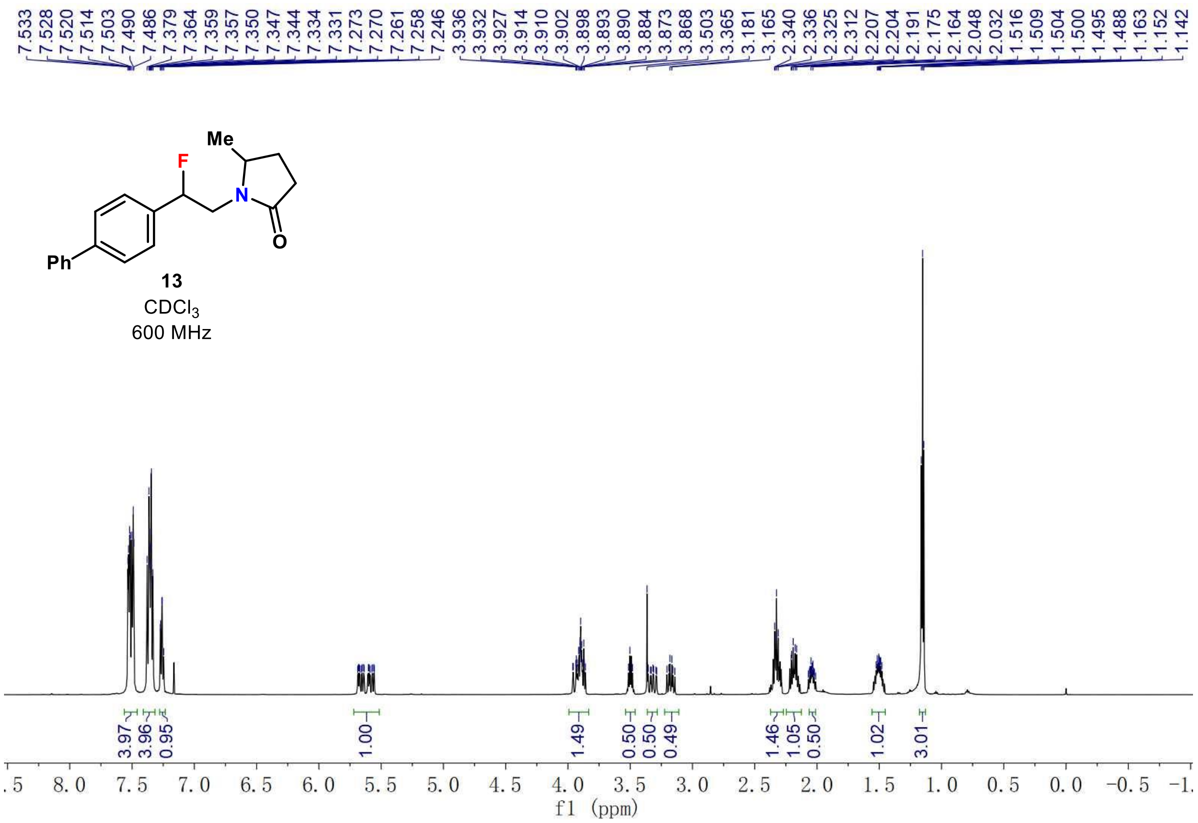

Supplementary Fig. 215. **^{1}H NMR** Spectra of **13**

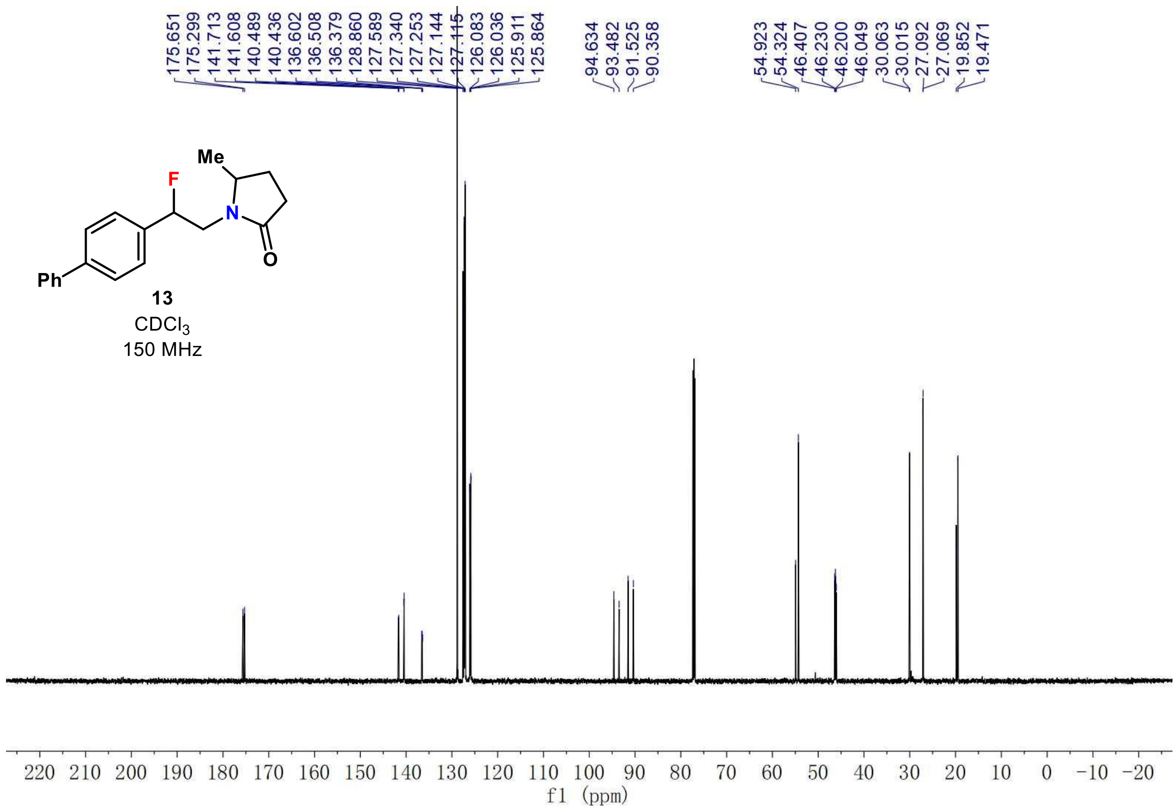

Supplementary Fig. 216. **^{13}C NMR** Spectra of **13**

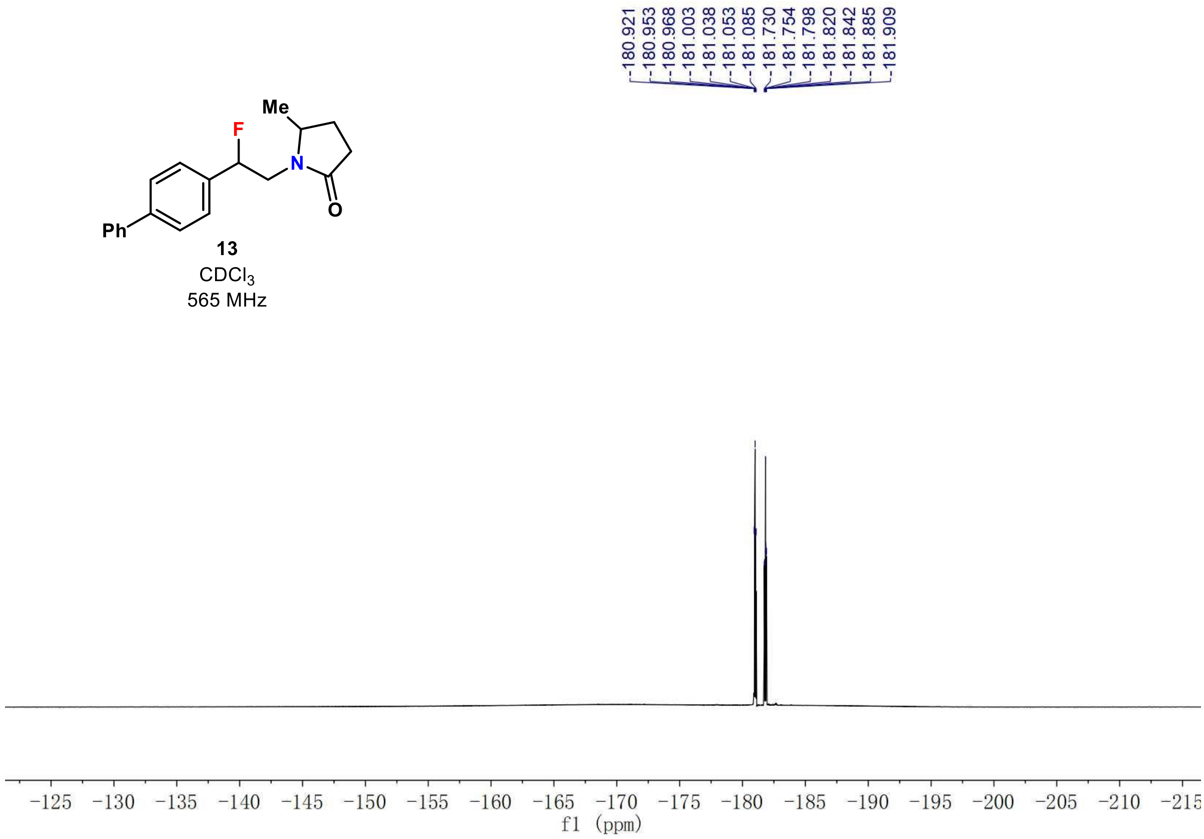

Supplementary Fig. 217. ^{19}**F NMR** Spectra of **13**

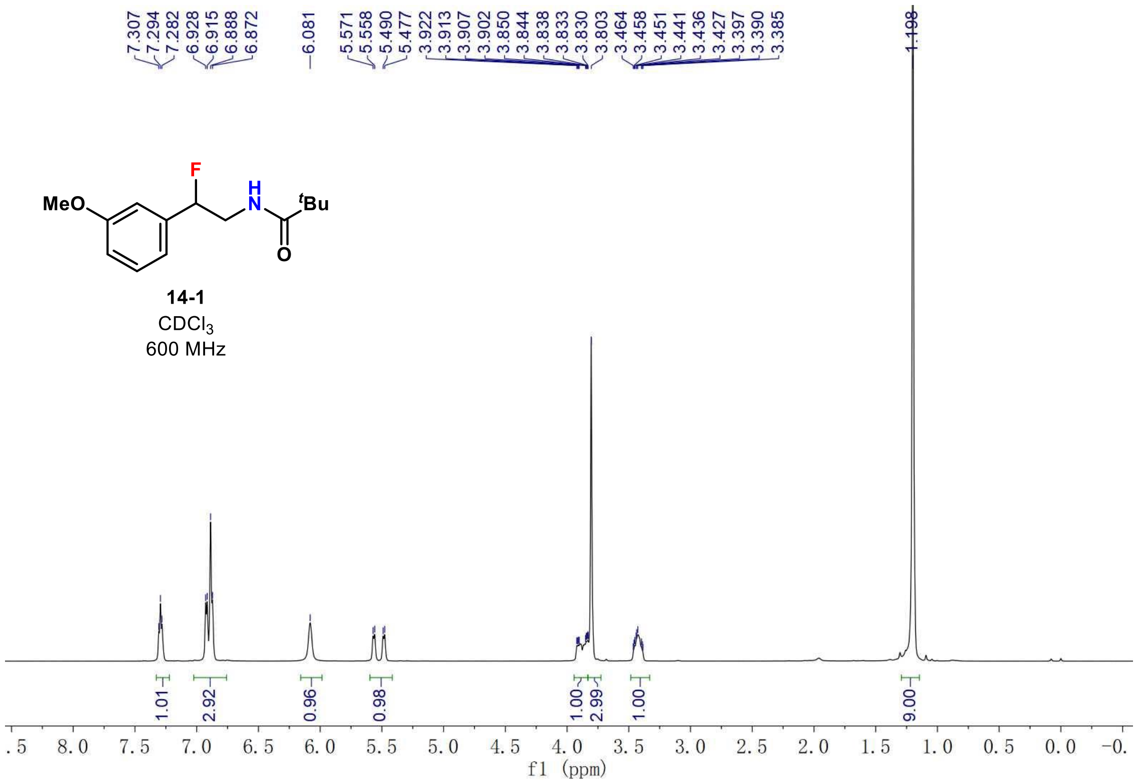

Supplementary Fig. 218. ^{1}**H NMR** Spectra of **14-1**

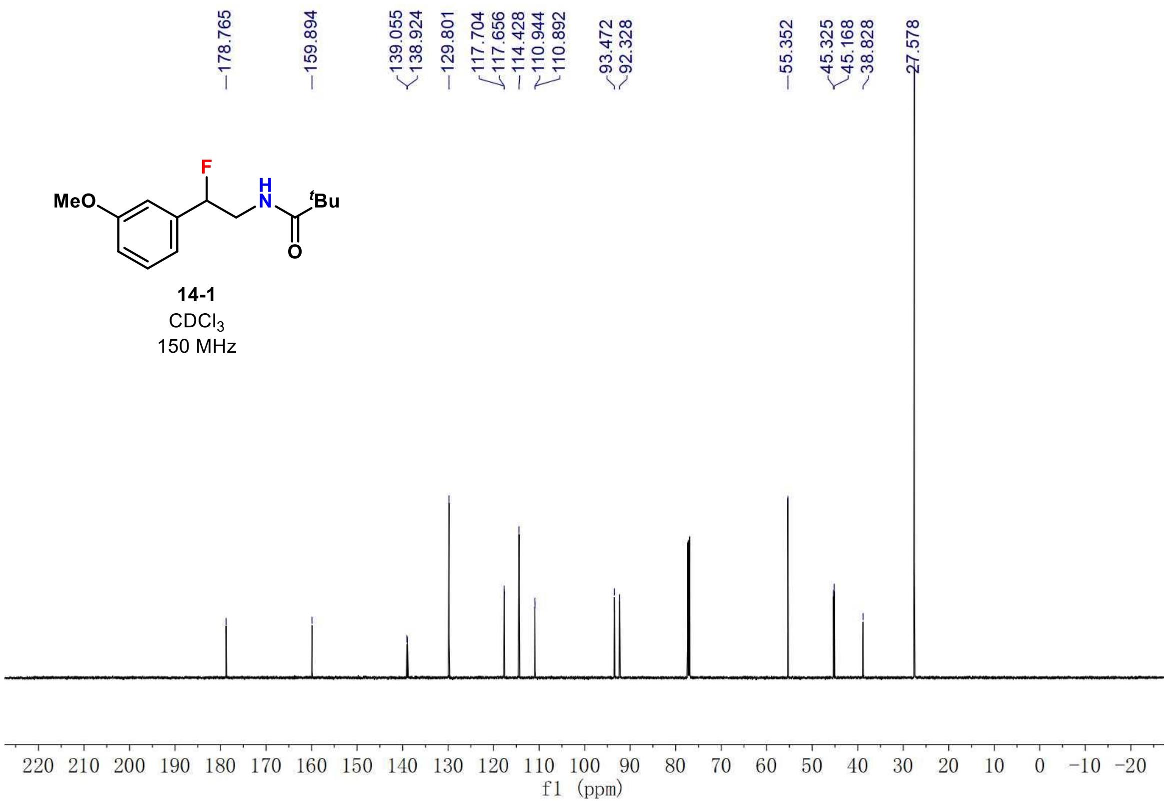

Supplementary Fig. 219. ^{13}**C NMR** Spectra of **14-1**

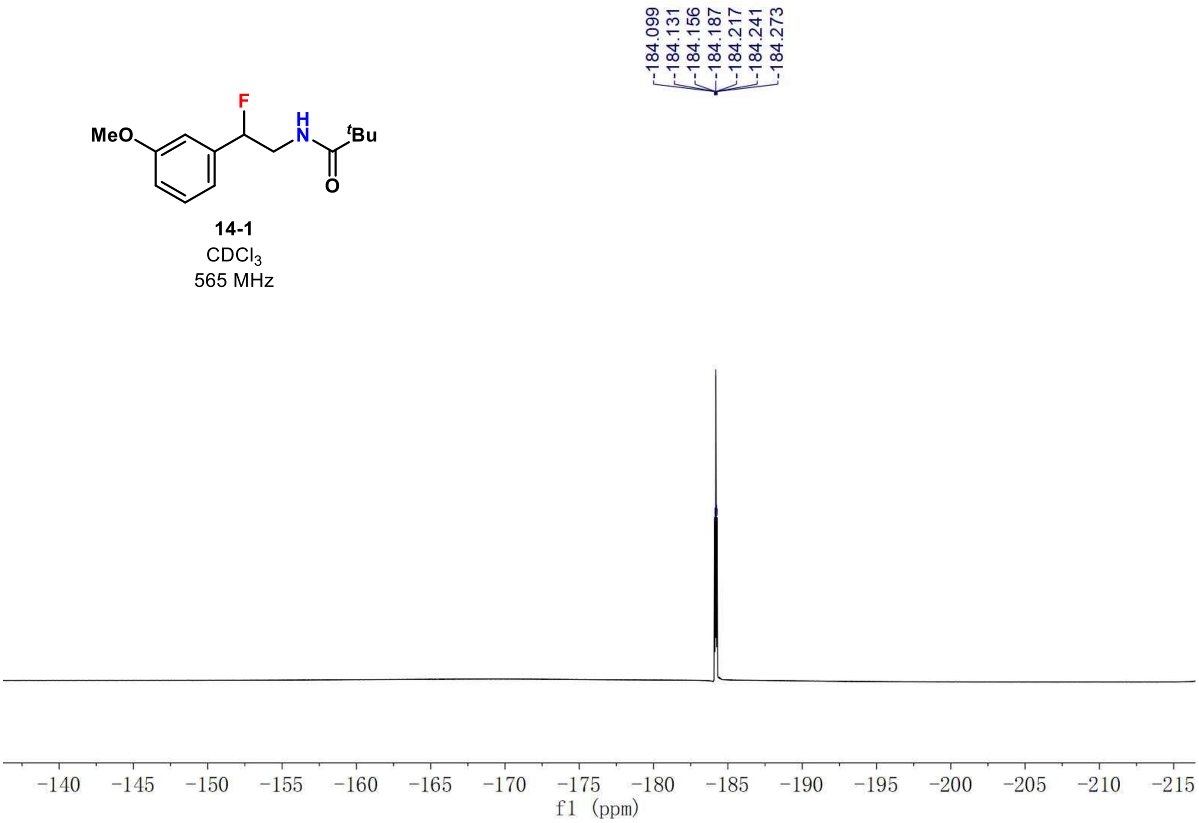

Supplementary Fig. 220. ^{19}**F NMR** Spectra of **14-1**

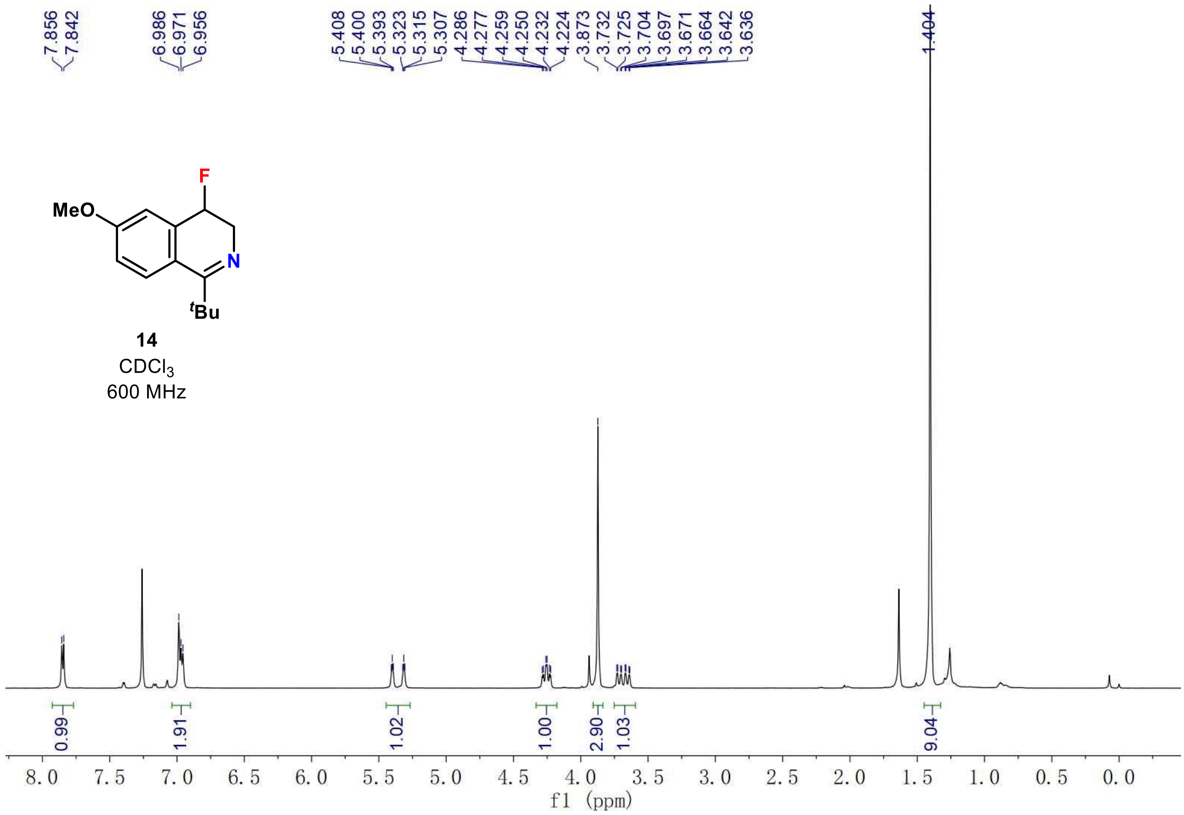

Supplementary Fig. 221. **^{1}H NMR** Spectra of **14**

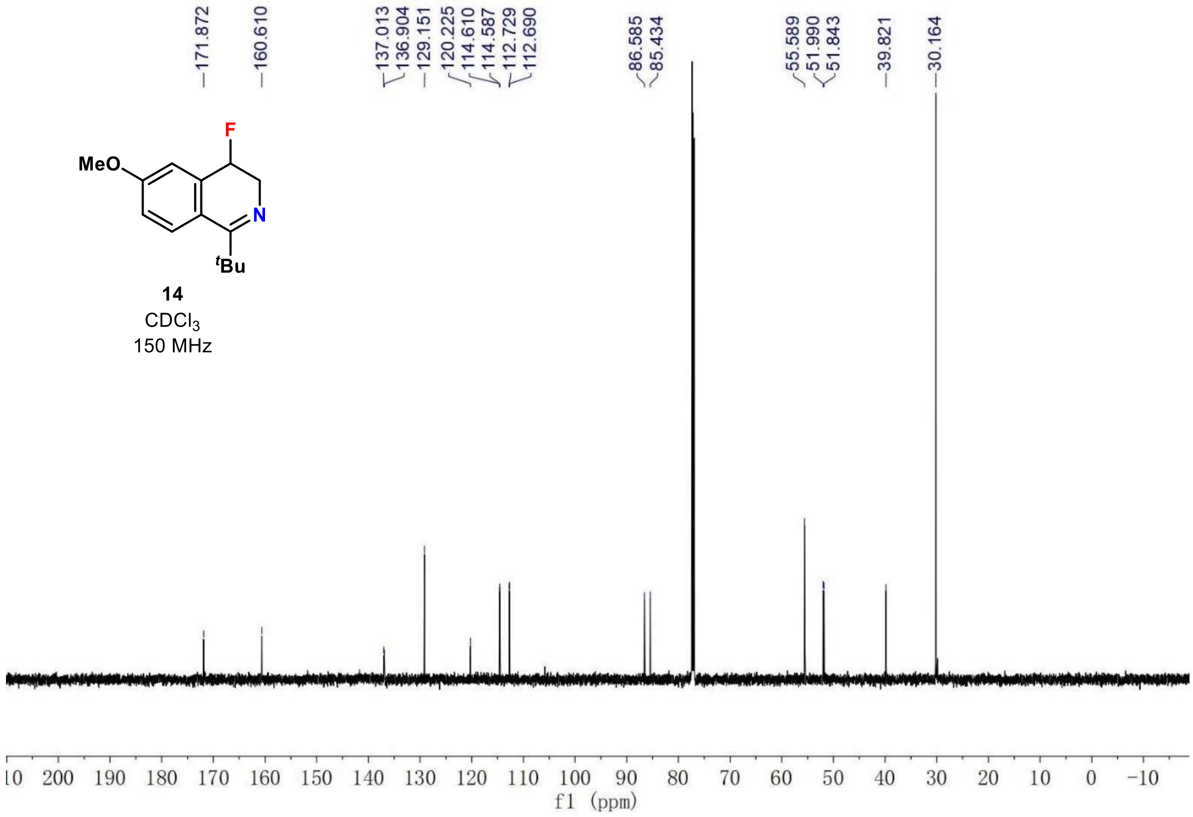

Supplementary Fig. 222. **^{13}C NMR** Spectra of **14**

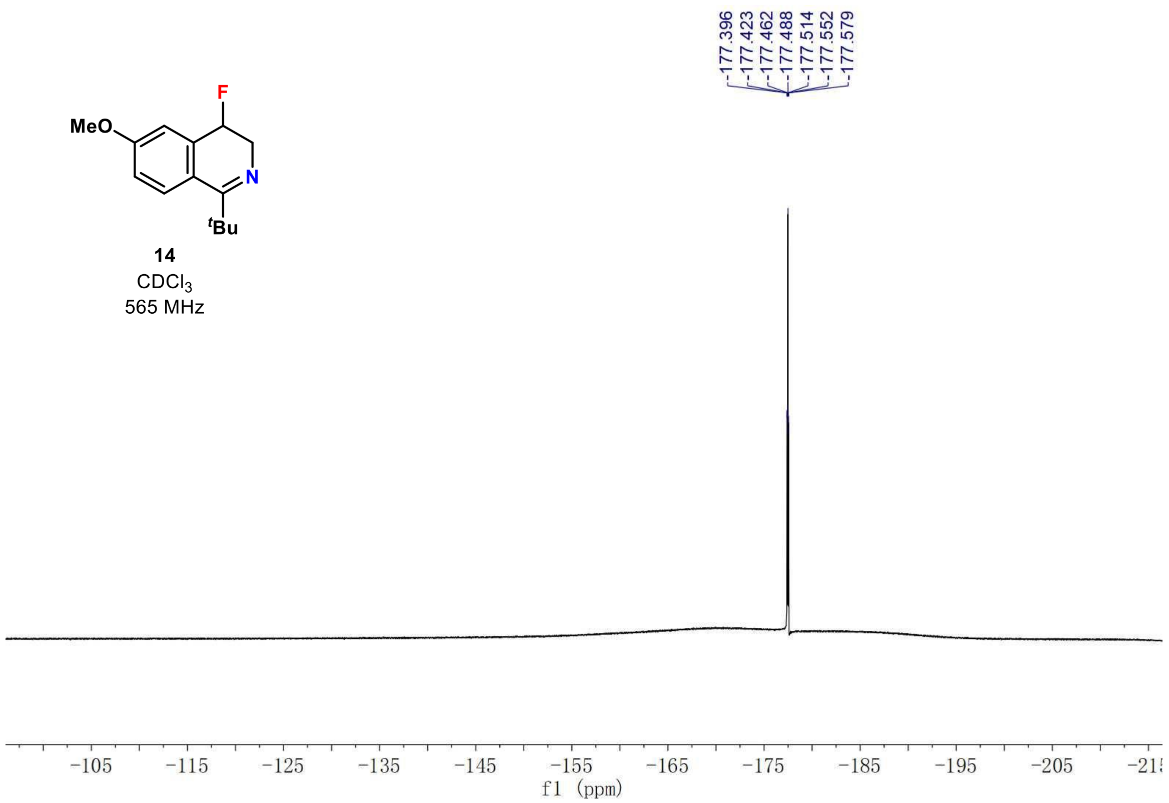

Supplementary Fig. 223. ^{19}**F NMR** Spectra of **14**

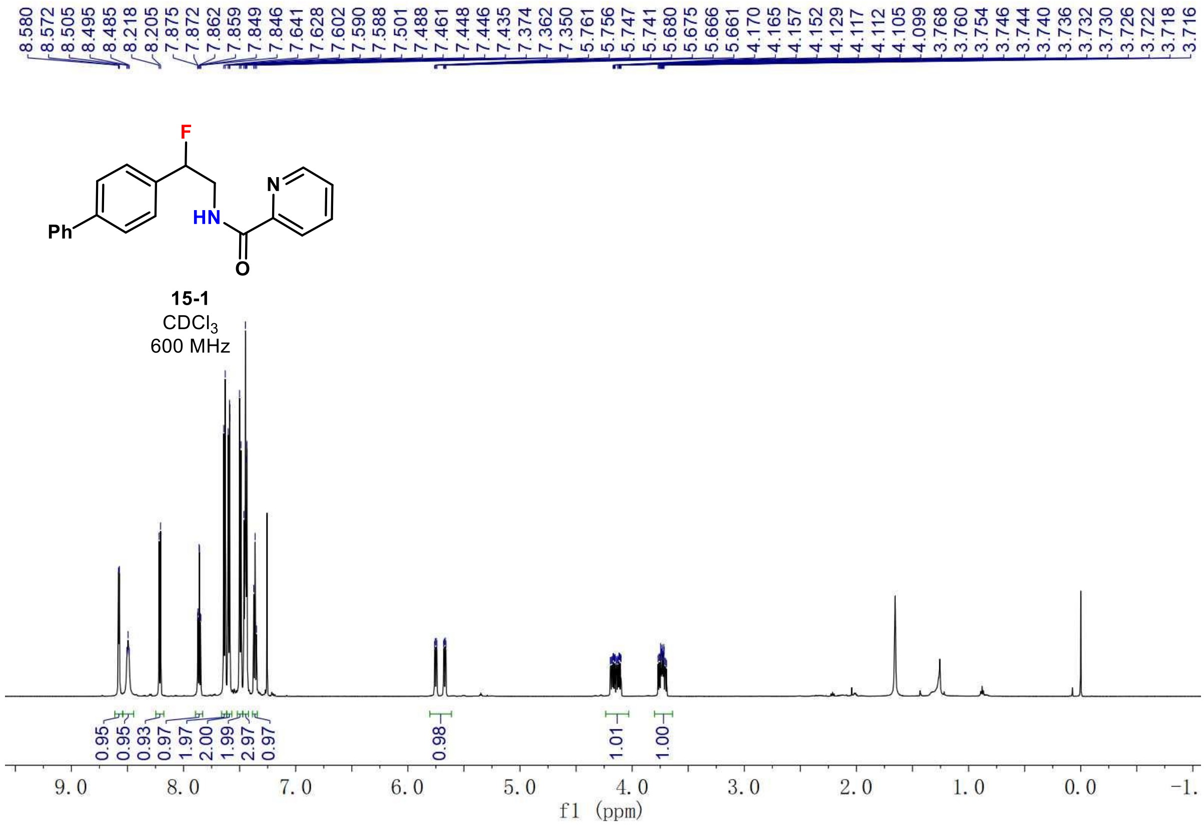

Supplementary Fig. 224. ^{1}**H NMR** Spectra of **15-1**

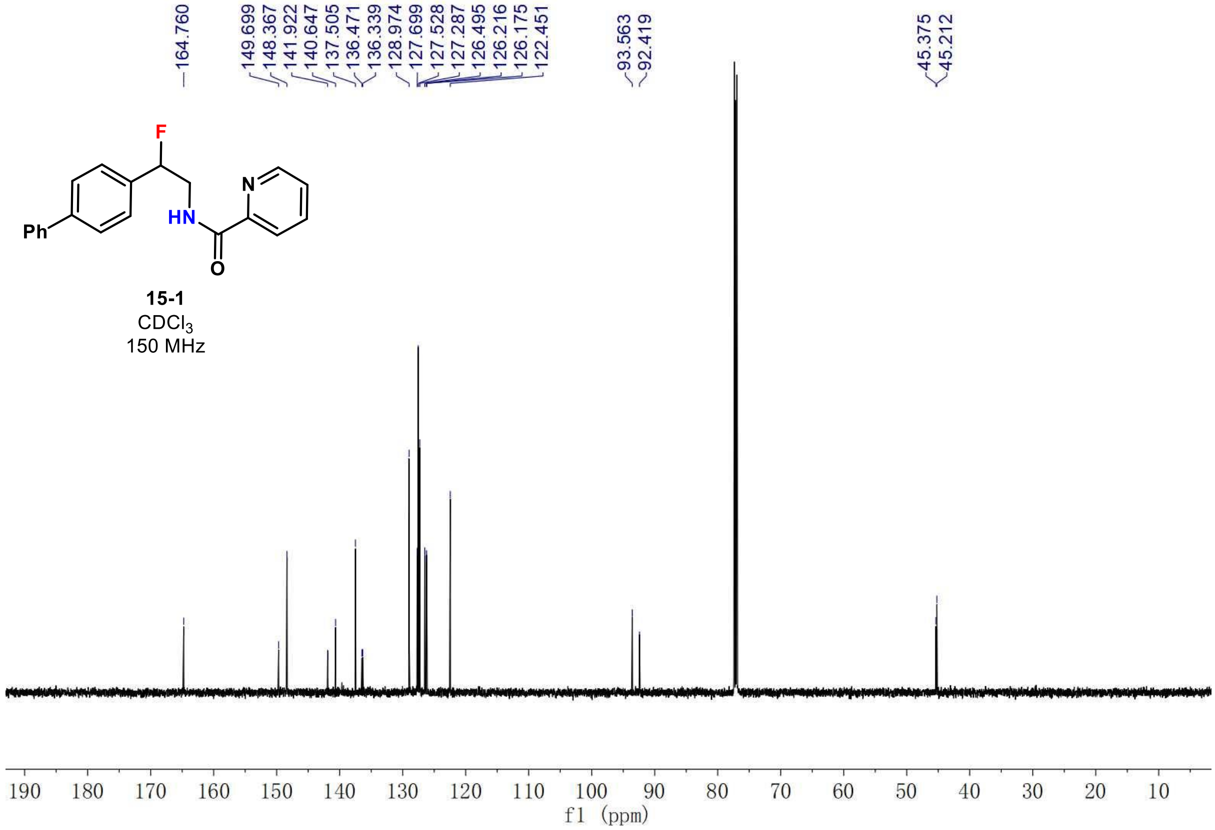

Supplementary Fig. 225. ^{13}**C NMR** Spectra of **15-1**

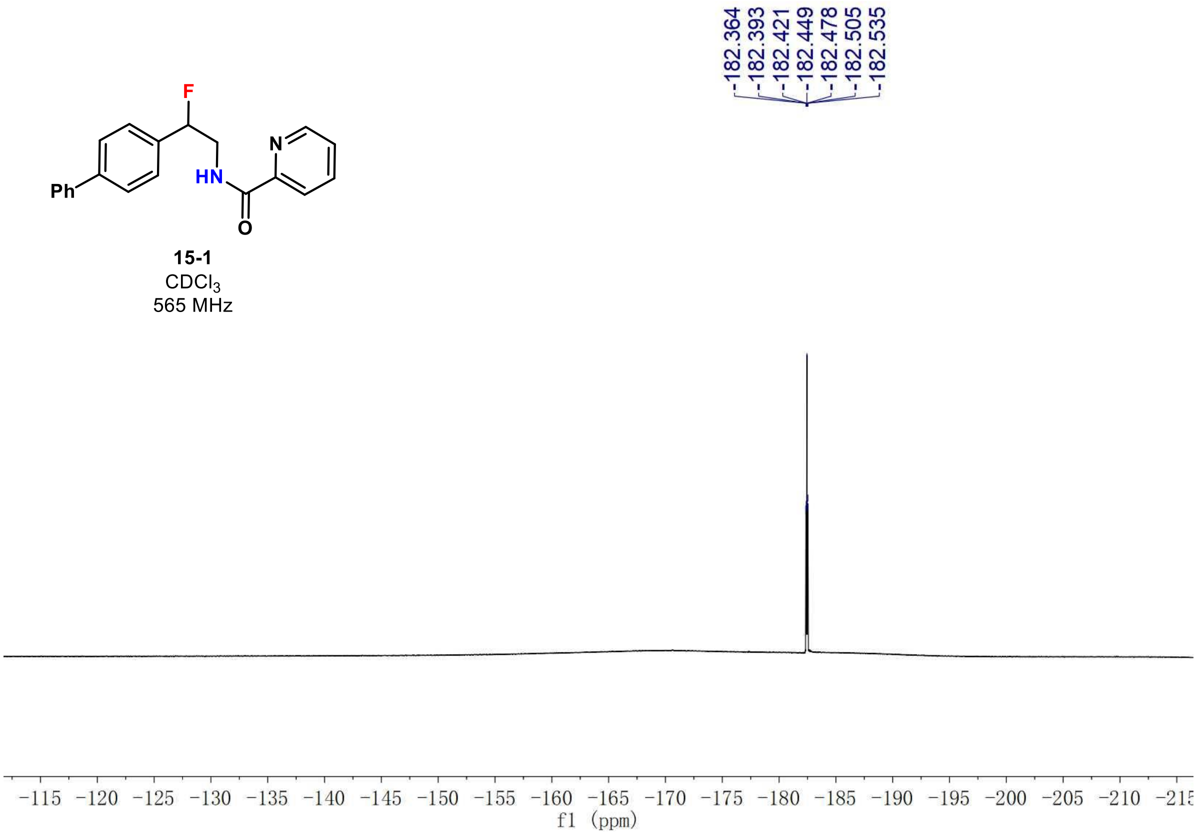

Supplementary Fig. 226. ^{19}**F NMR** Spectra of **15-1**

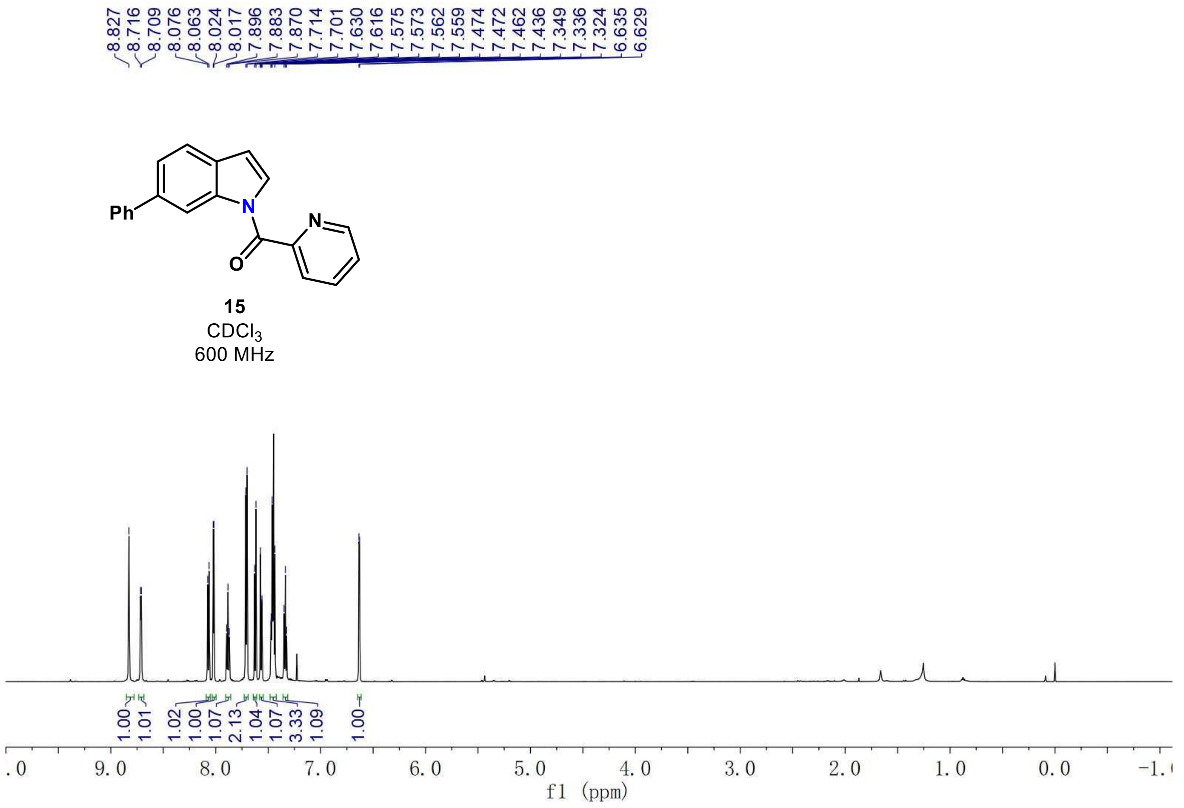

Supplementary Fig. 227. ^{1}H NMR Spectra of **15**

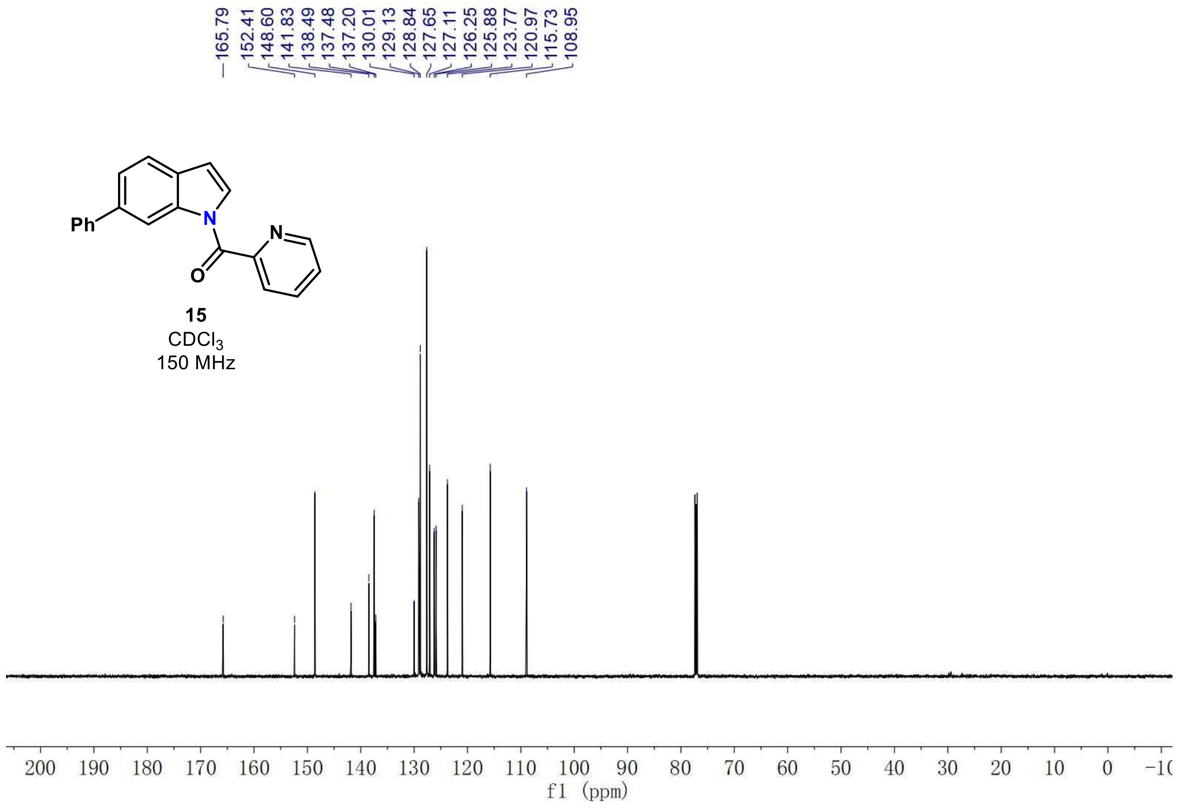

Supplementary Fig. 228. ^{13}C NMR Spectra of **15**

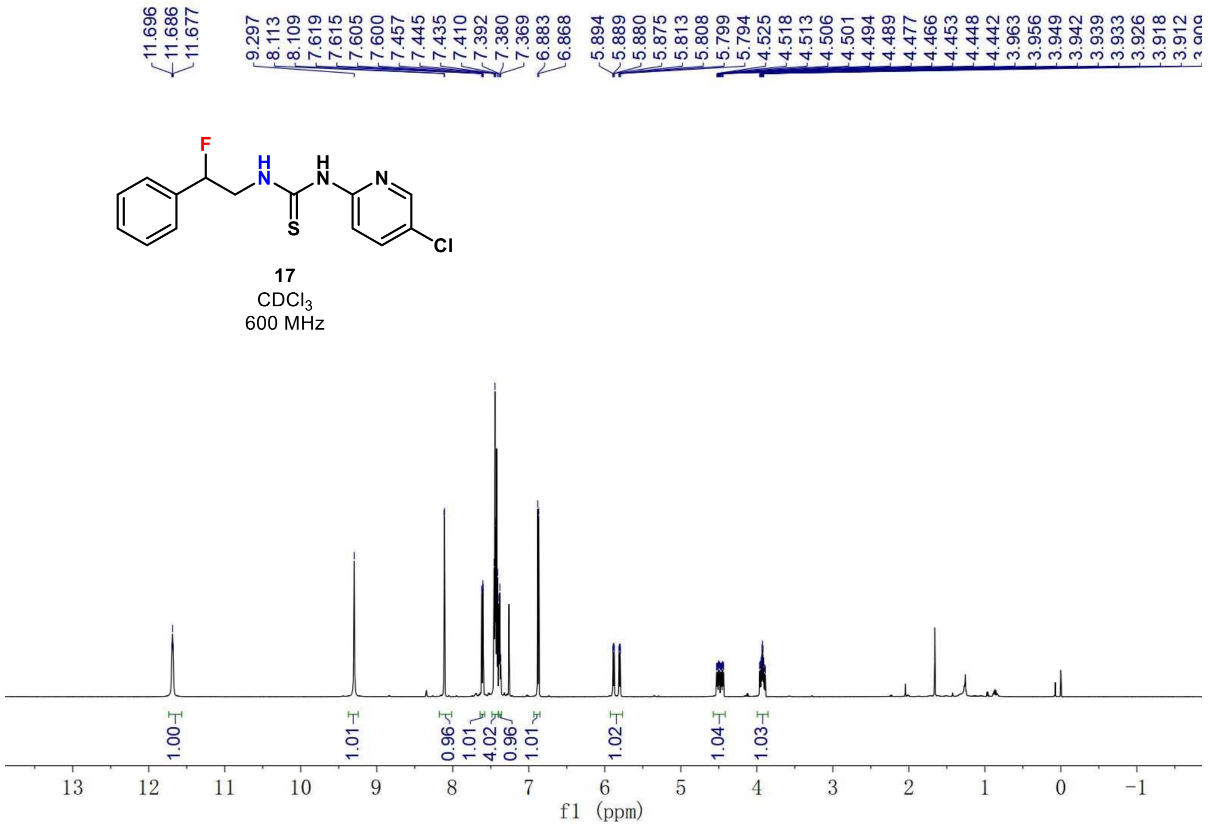

Supplementary Fig. 229. ^{1}H NMR Spectra of **17**

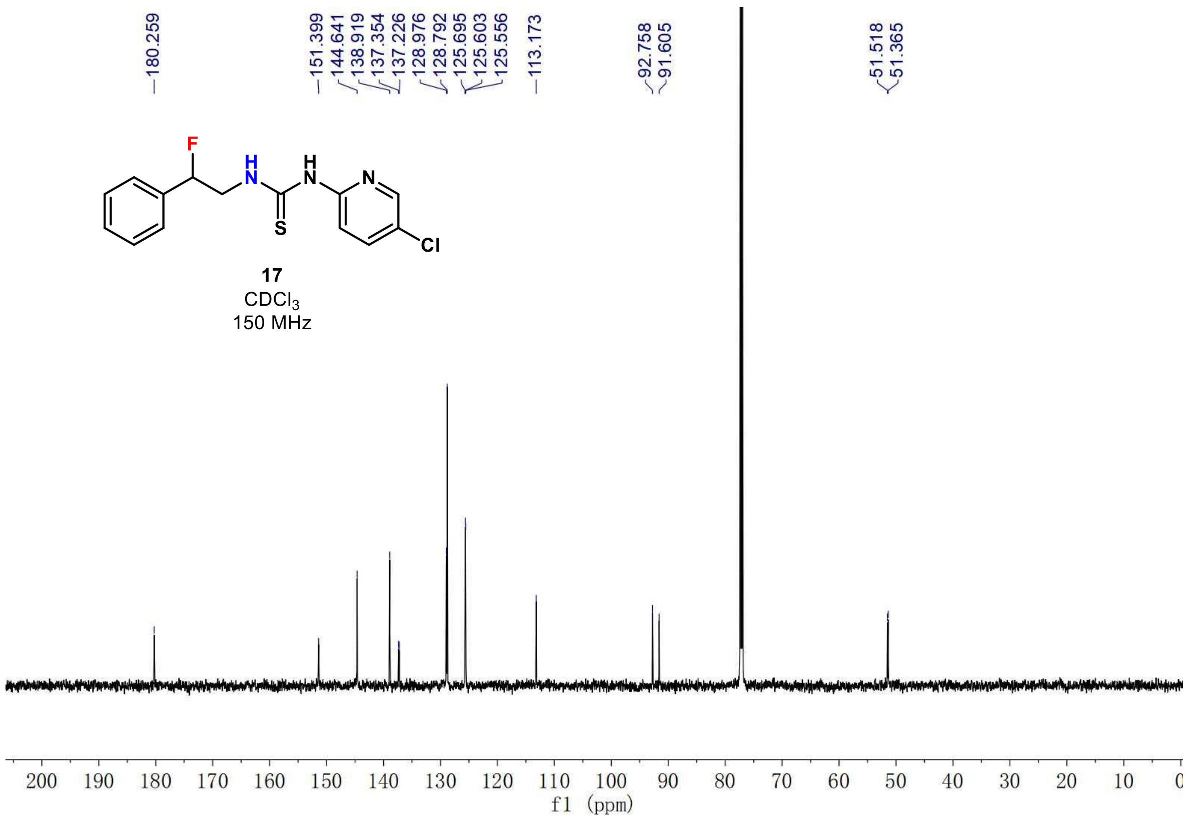

Supplementary Fig. 230. ^{13}**C NMR** Spectra of **17**

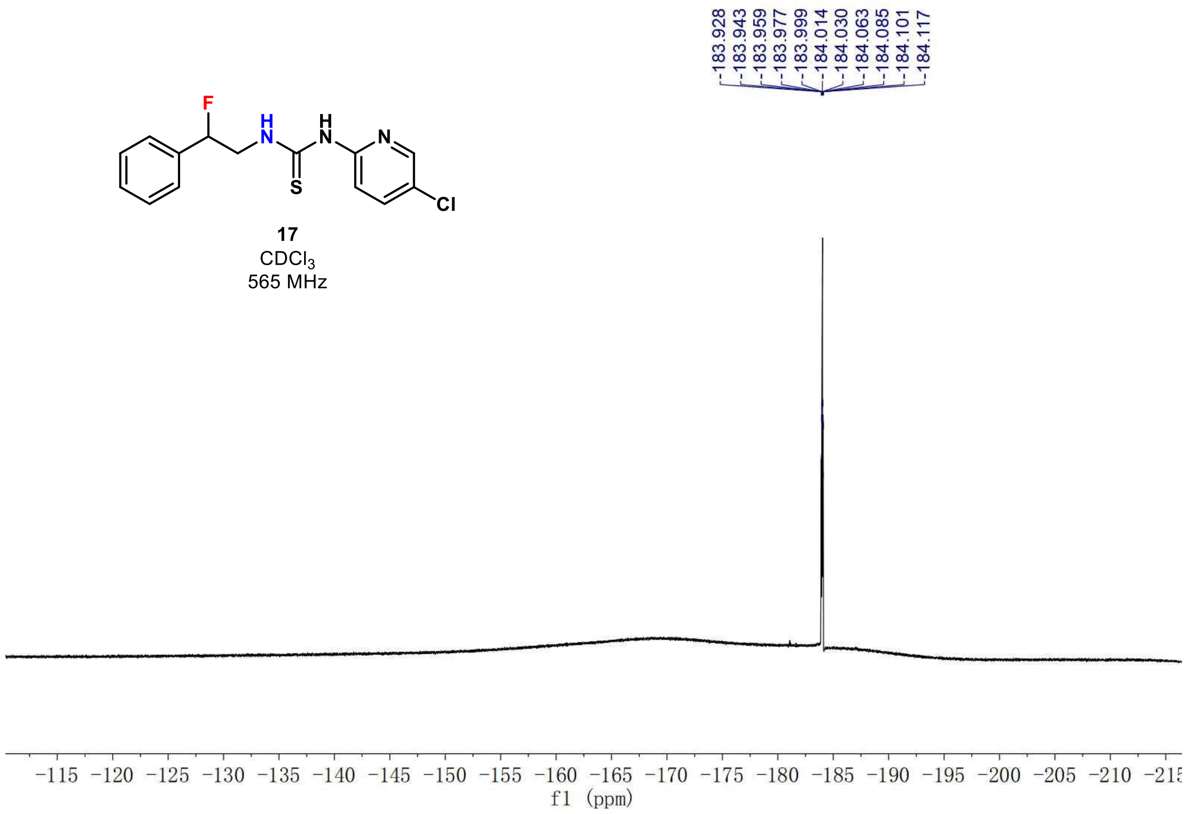

Supplementary Fig. 231. ^{19}F NMR Spectra of **17**

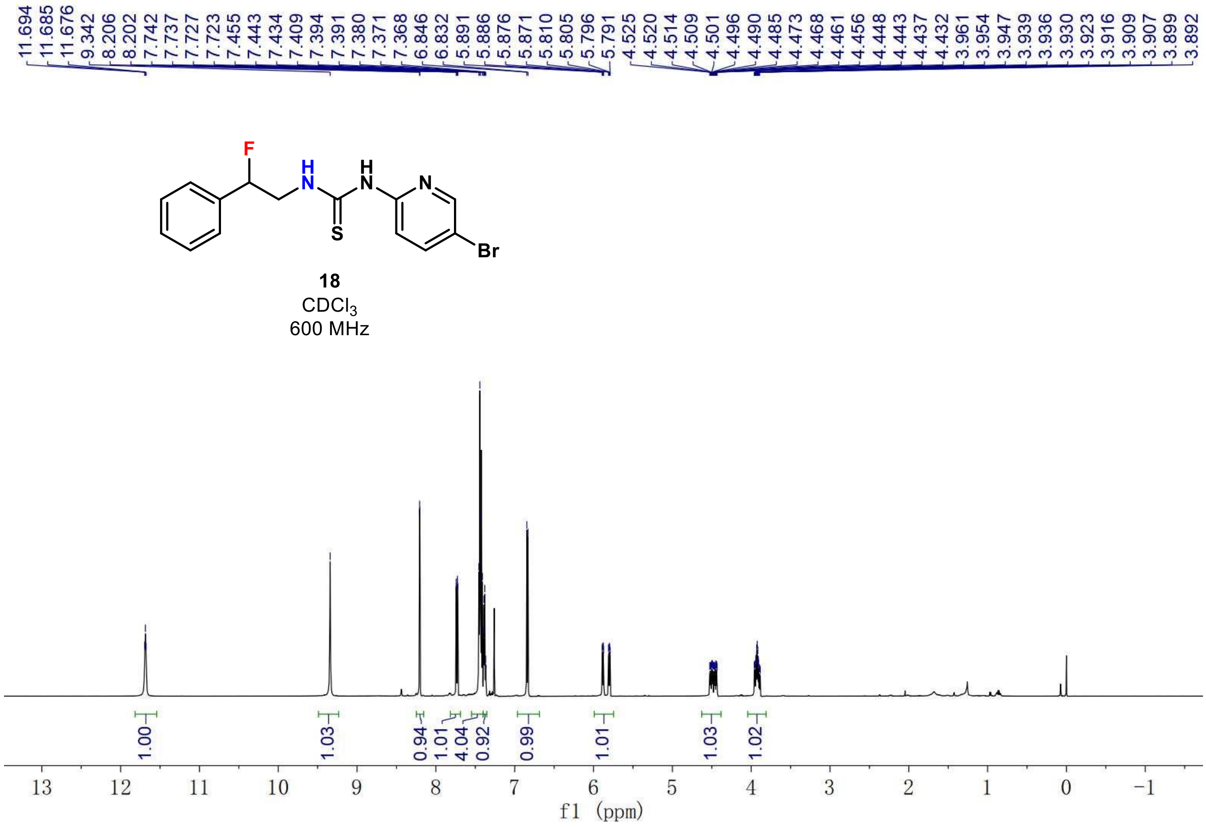

Supplementary Fig. 232. ^{1}H NMR Spectra of **18**

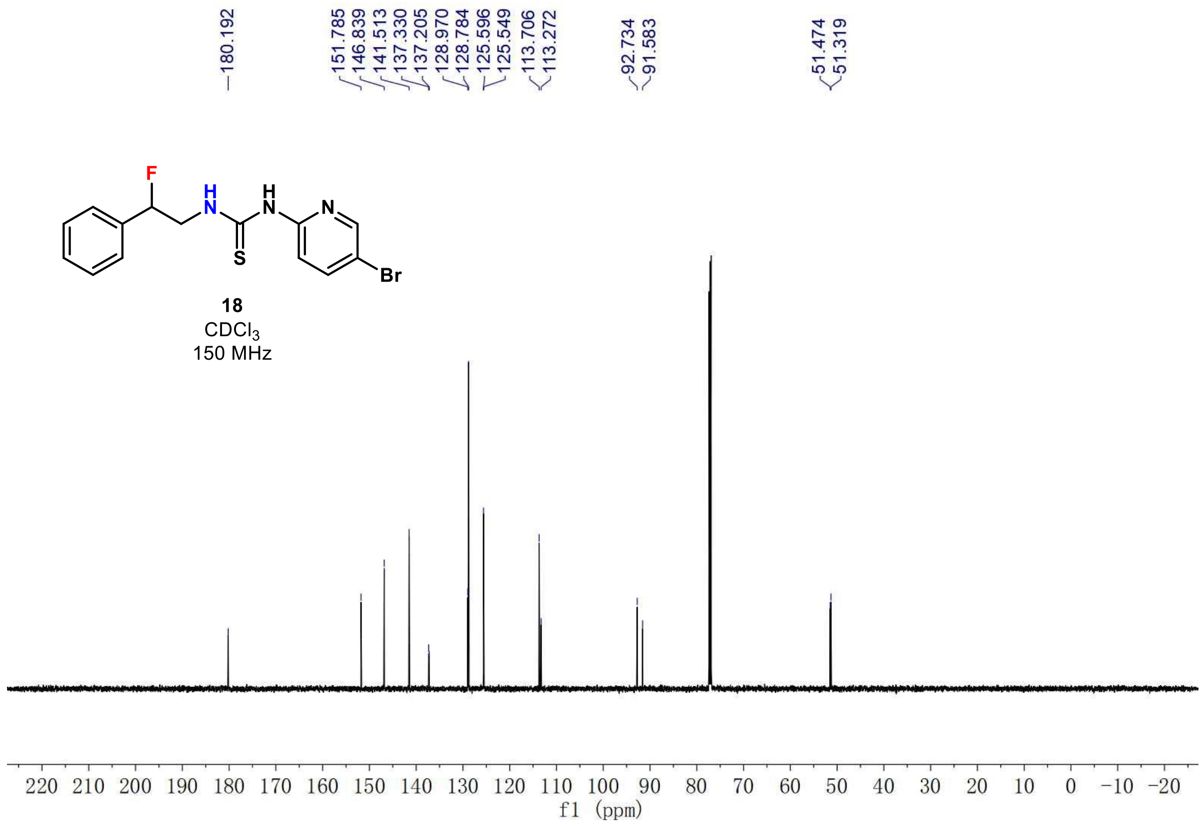

Supplementary Fig. 233. ^{13}**C NMR** Spectra of **18**

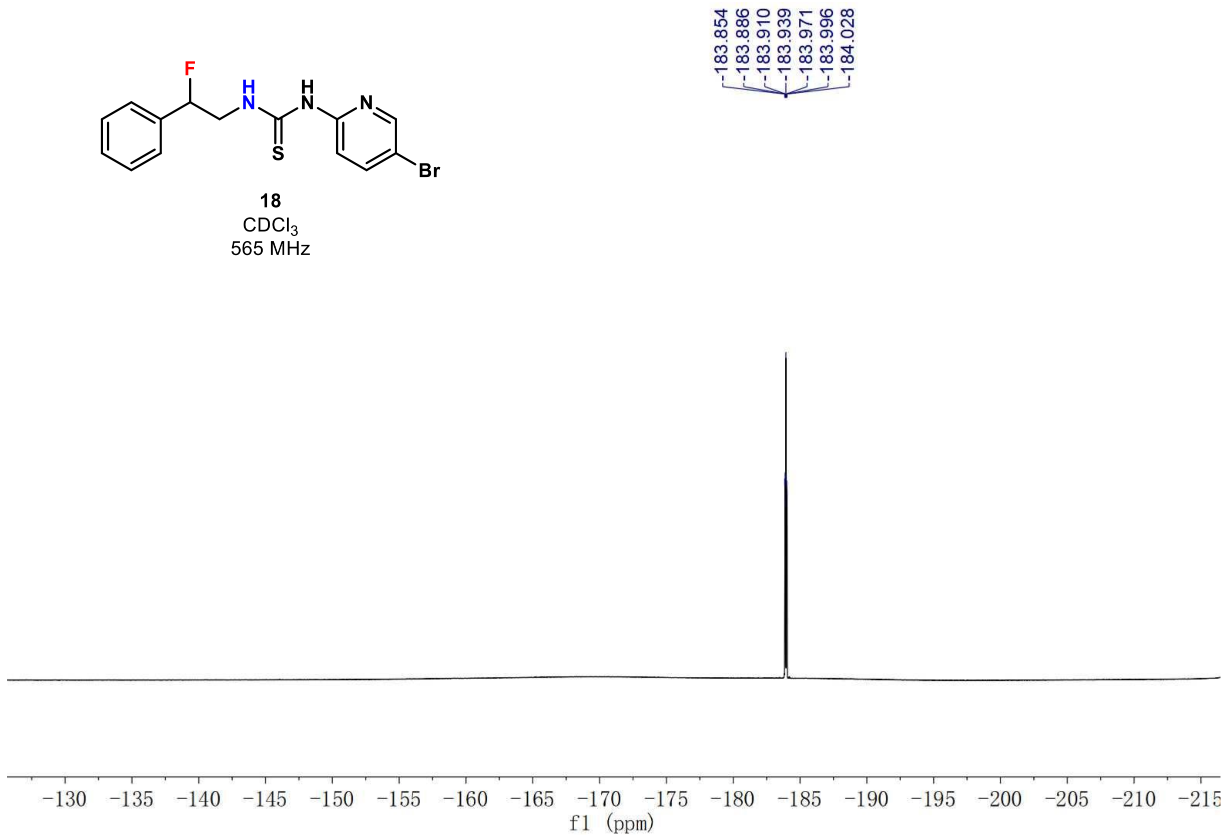

Supplementary Fig. 234. ^{19}**F NMR** Spectra of **18**

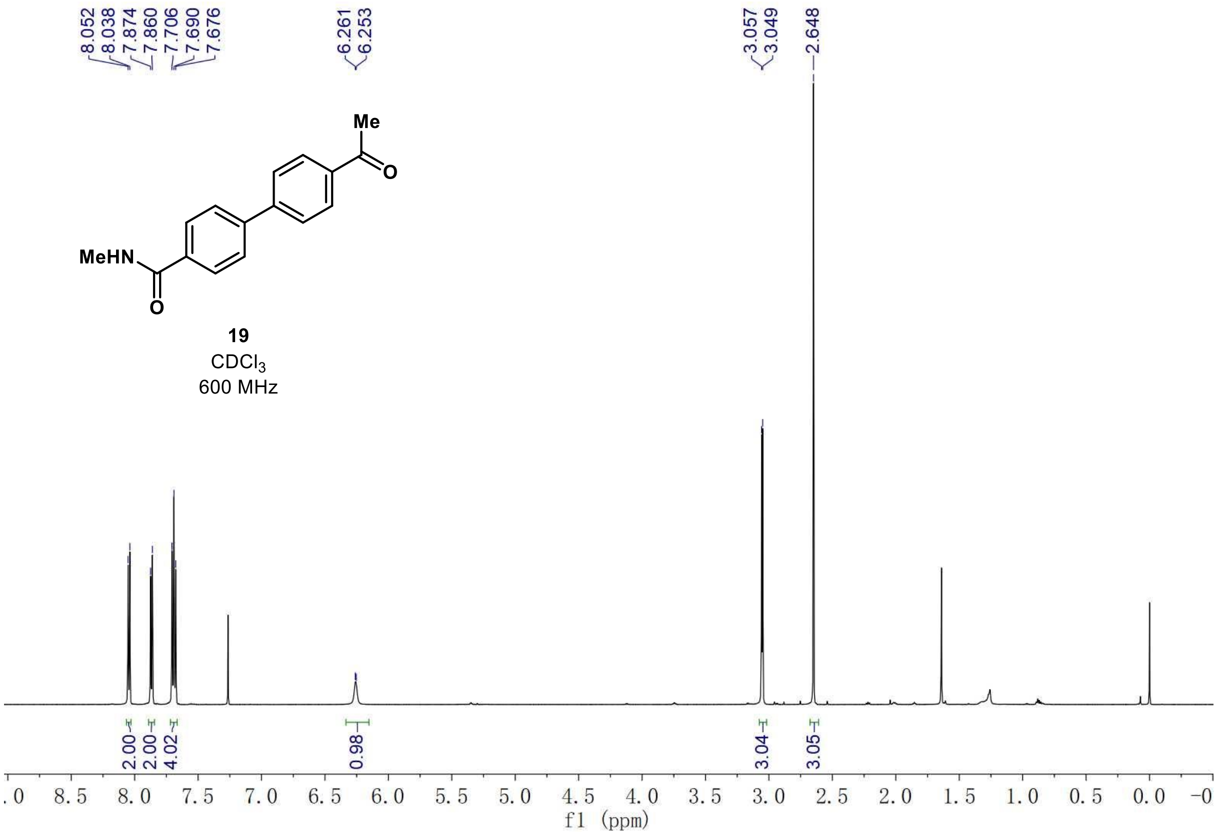

Supplementary Fig. 235. ^{13}C NMR Spectra of **19**

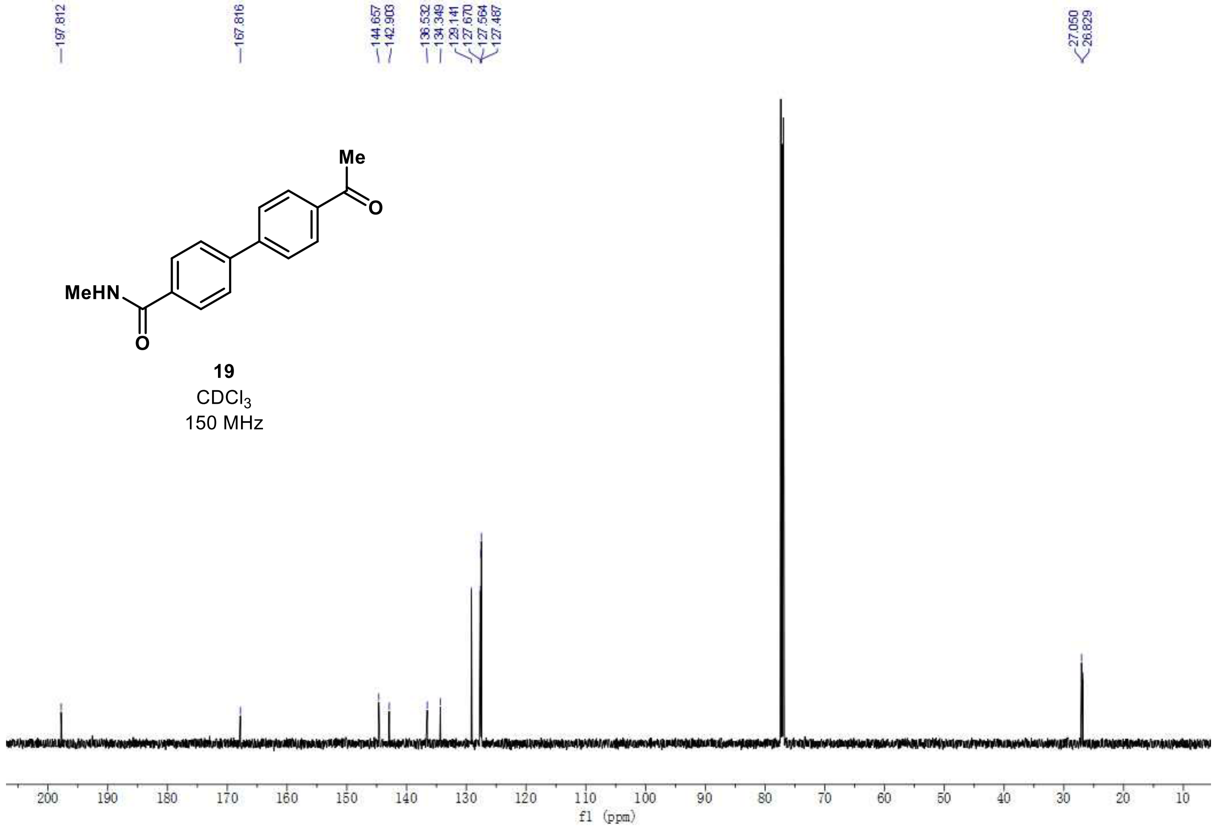

Supplementary Fig. 236. ^{13}C NMR Spectra of **19**

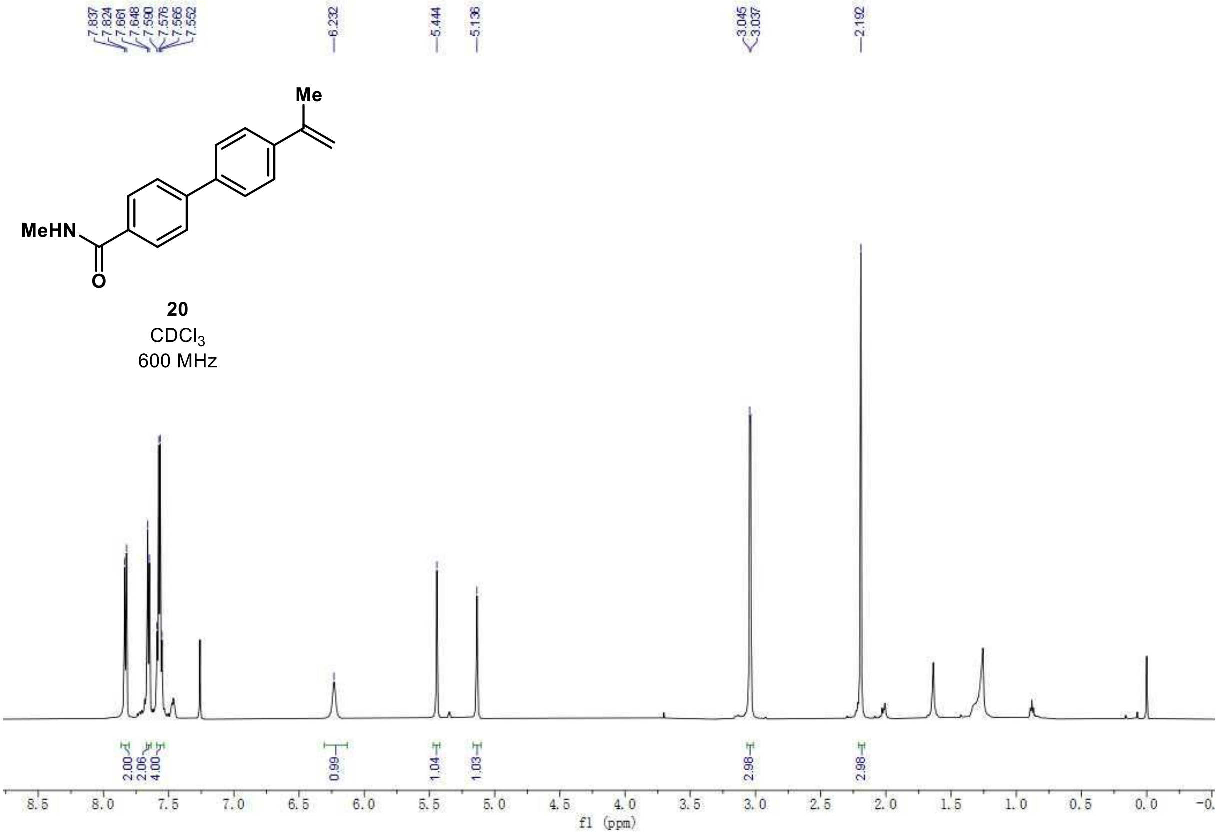

Supplementary Fig. 237. ^{1}**H NMR** Spectra of **20**

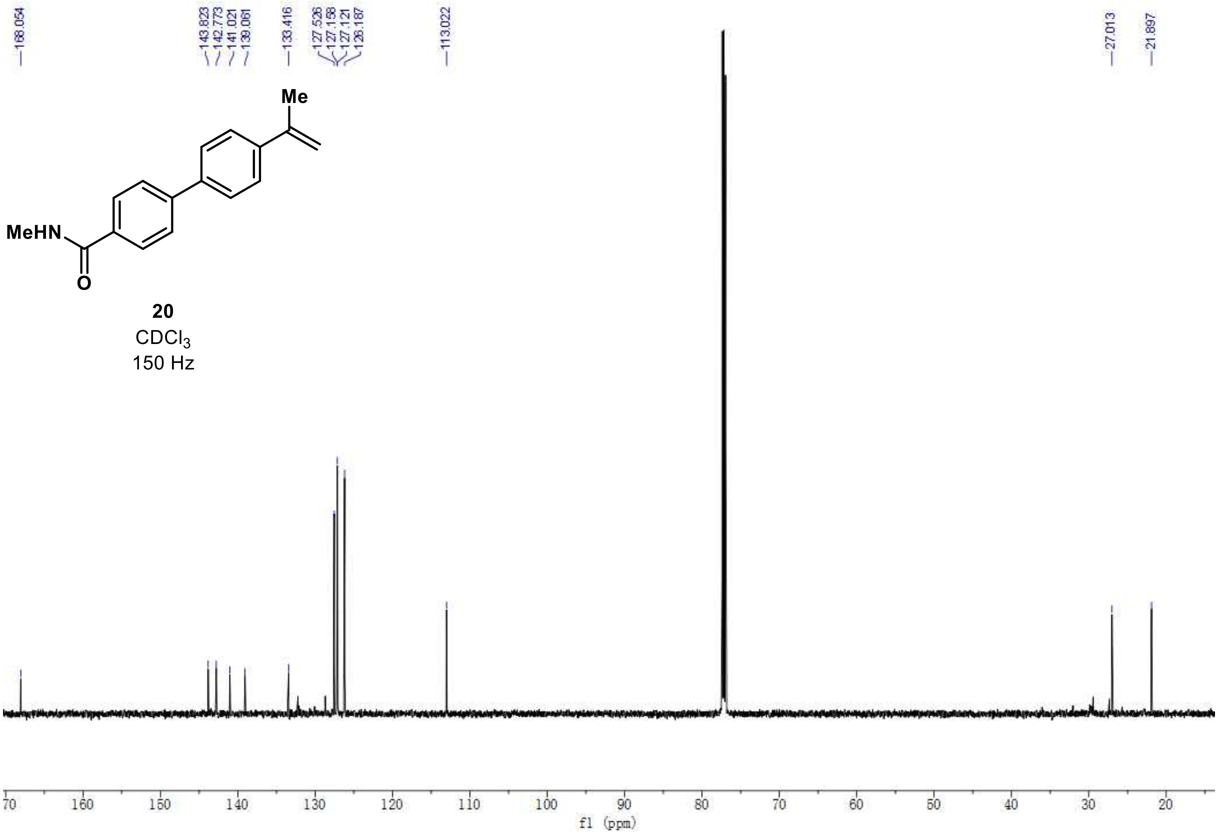

Supplementary Fig. 238. ^{13}**C NMR** Spectra of **20**

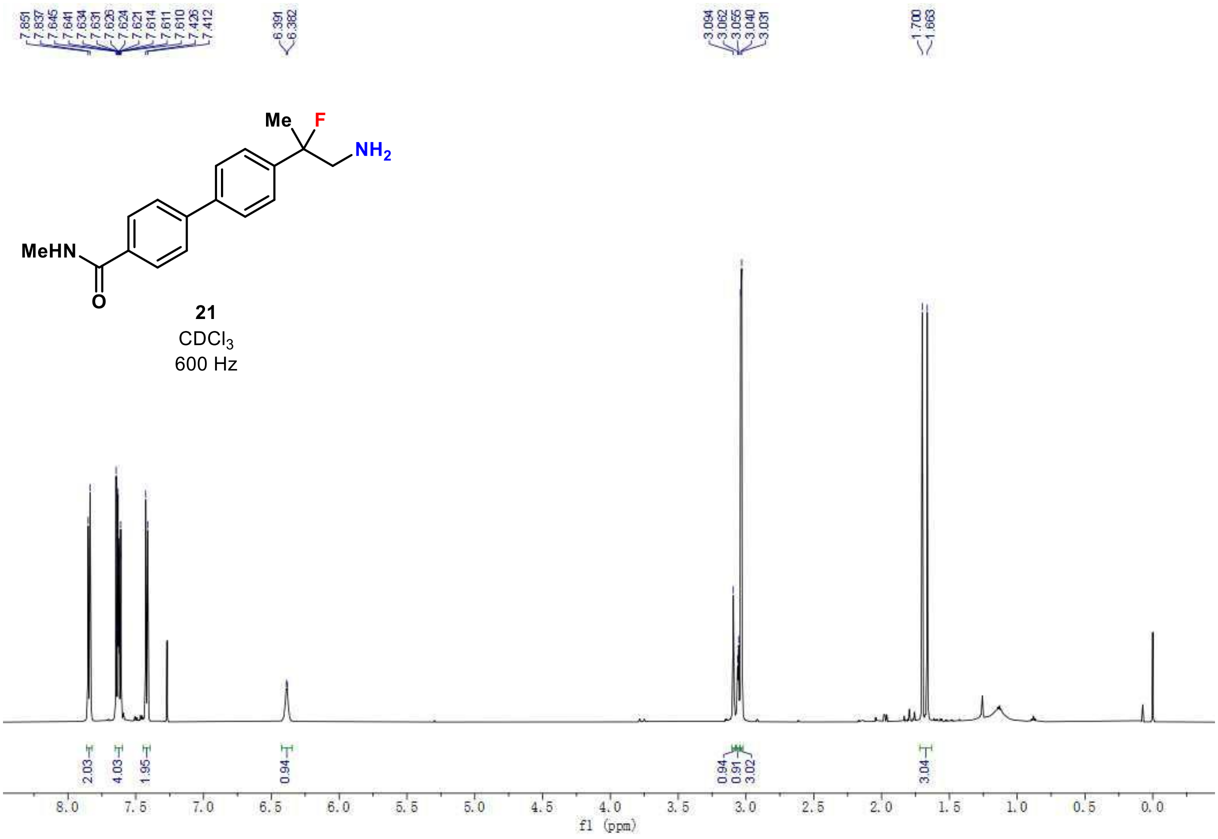

Supplementary Fig. 239. ^{1}H NMR Spectra of **21**

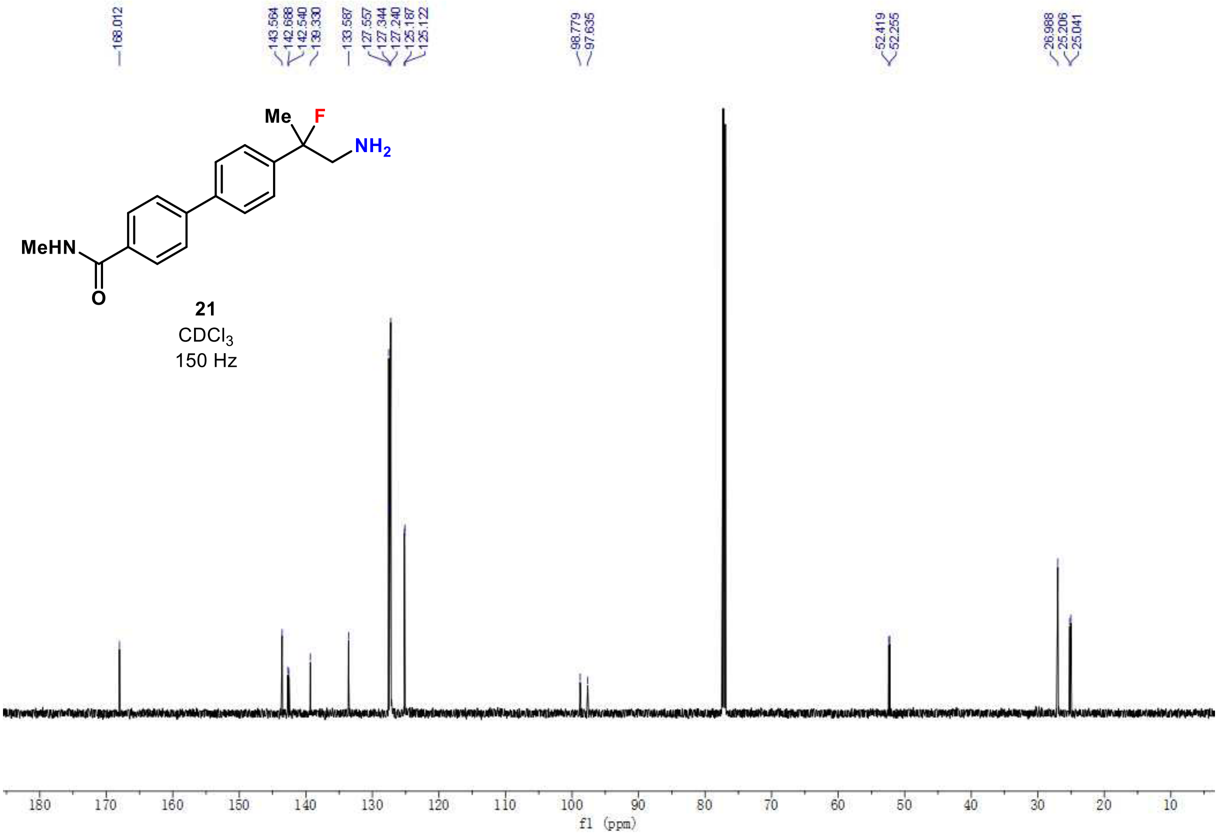

Supplementary Fig. 240. ^{13}C NMR Spectra of **21**

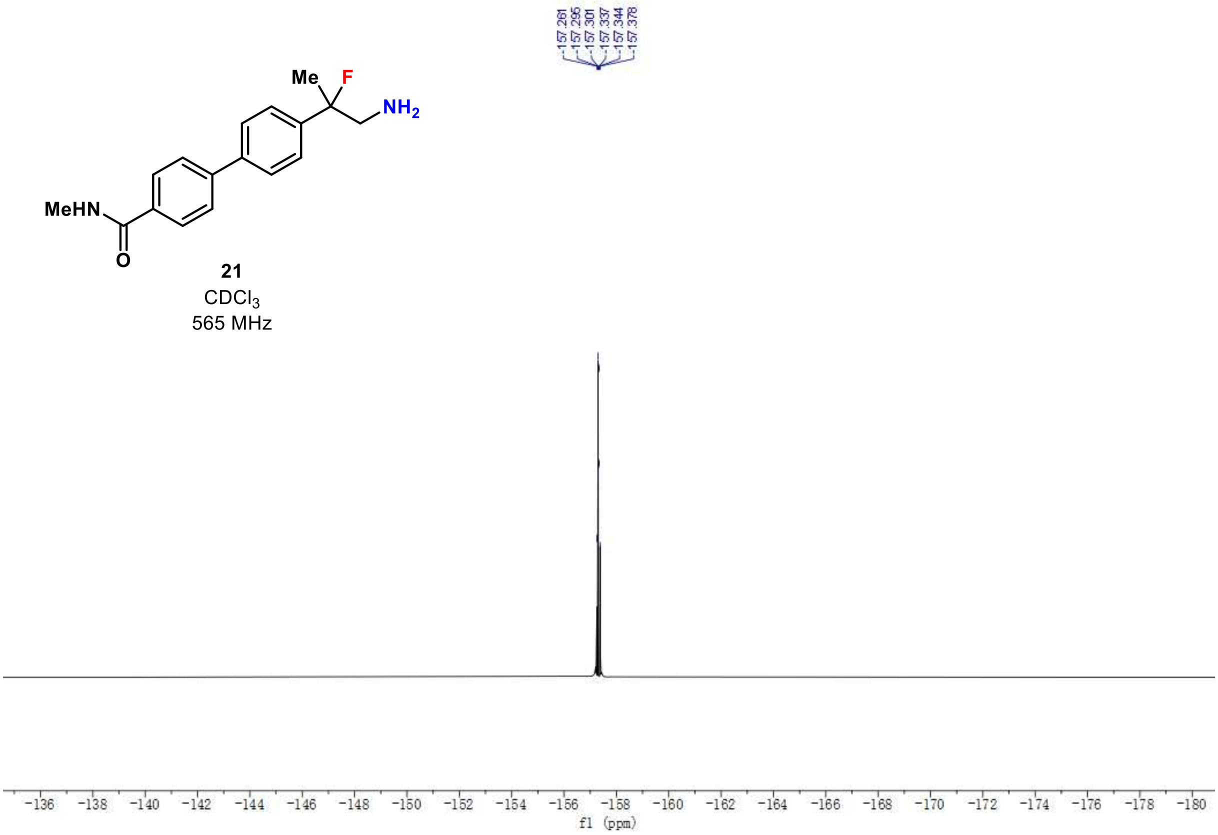

Supplementary Fig. 241. **^{19}F NMR** Spectra of **21**

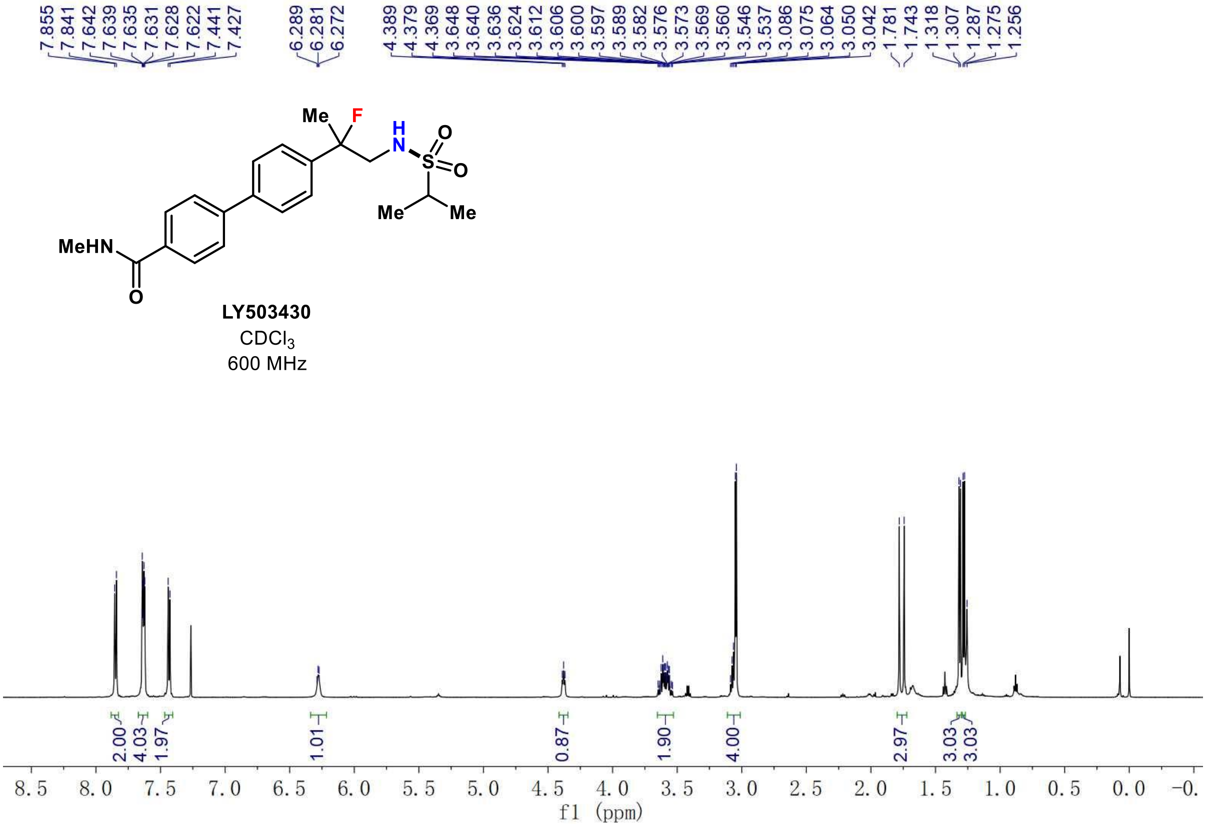

Supplementary Fig. 242. **^{1}H NMR** Spectra of **LY503430**

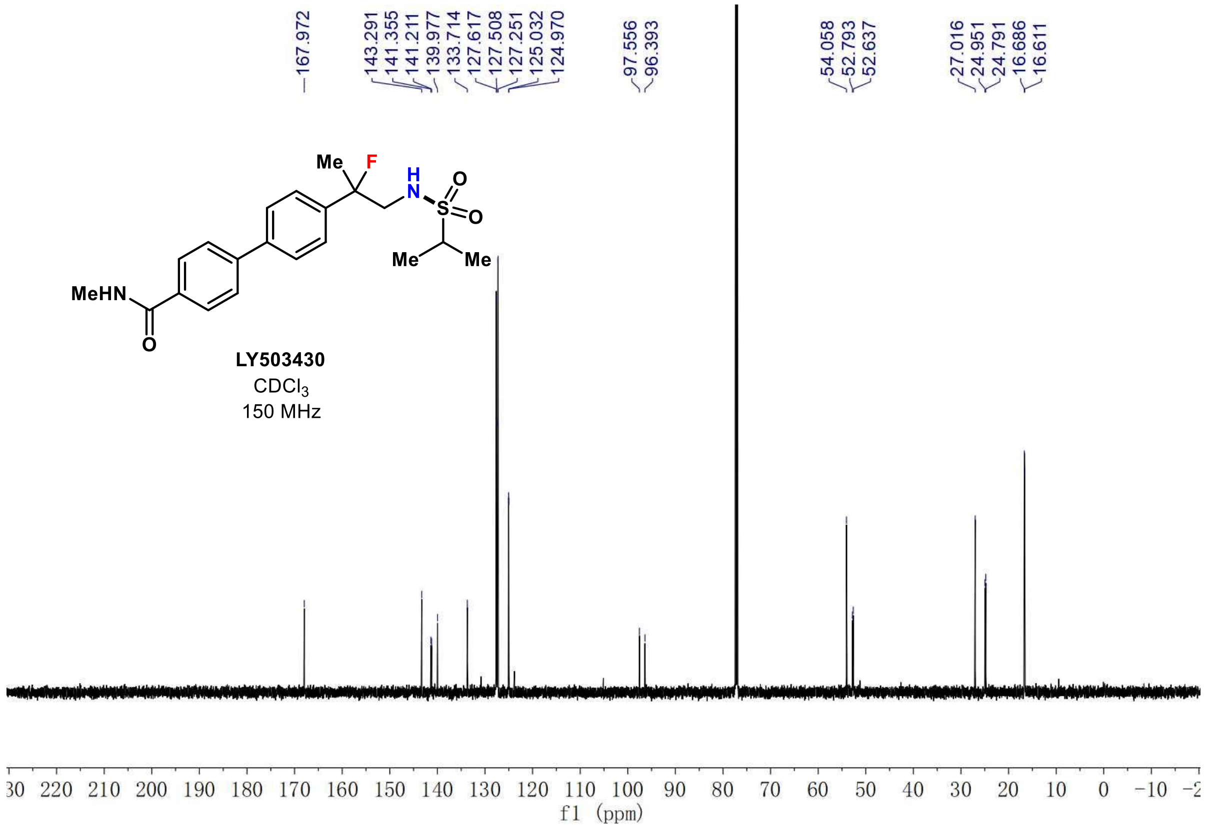

Supplementary Fig. 243. ^{13}**C NMR** Spectra of **LY503430**

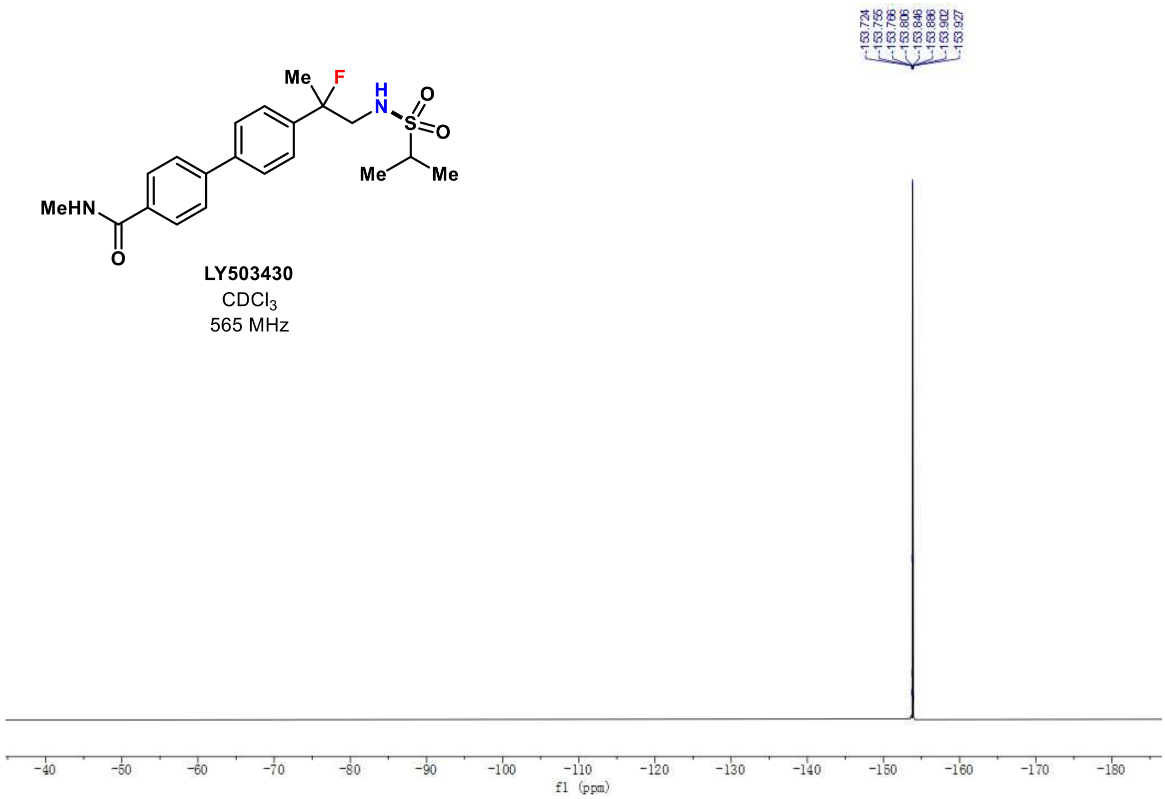

Supplementary Fig. 244. ^{19}**F NMR** Spectra of **LY503430**

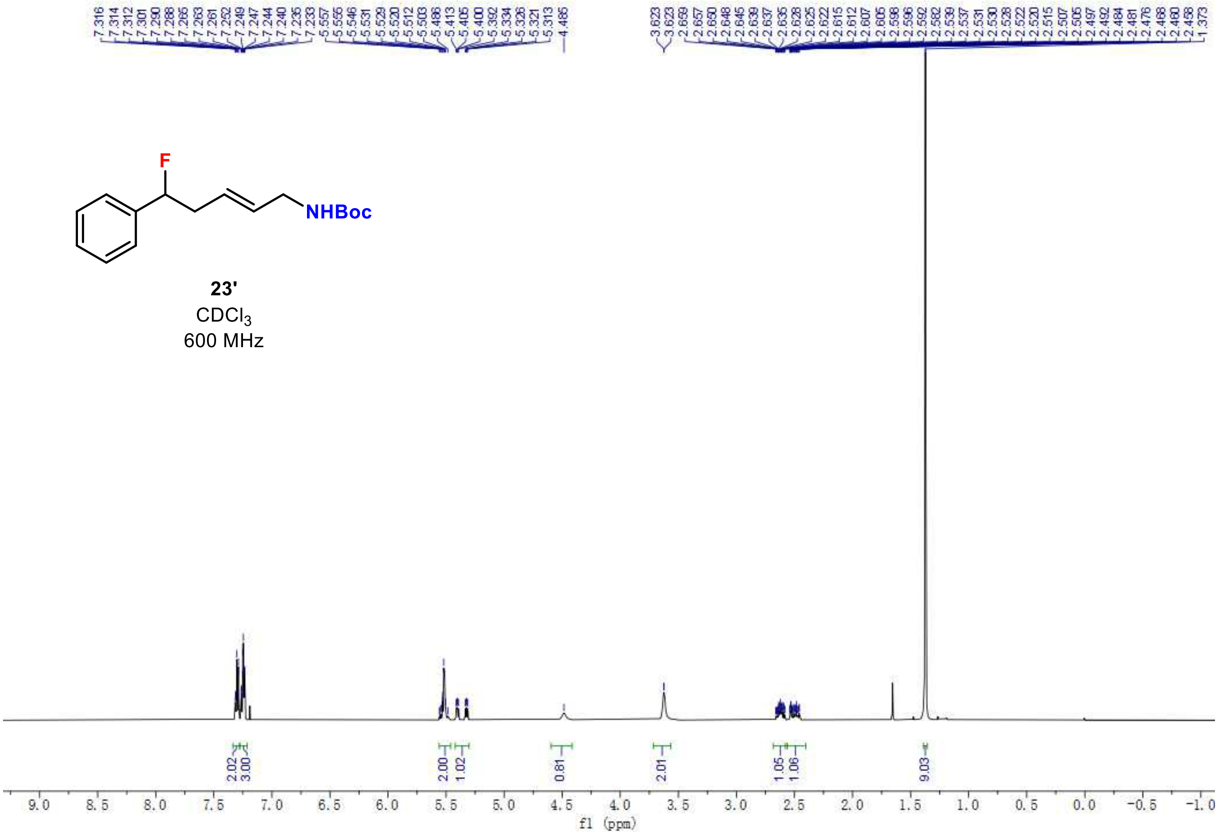

Supplementary Fig. 245. **^{1}H NMR** Spectra of **23'**

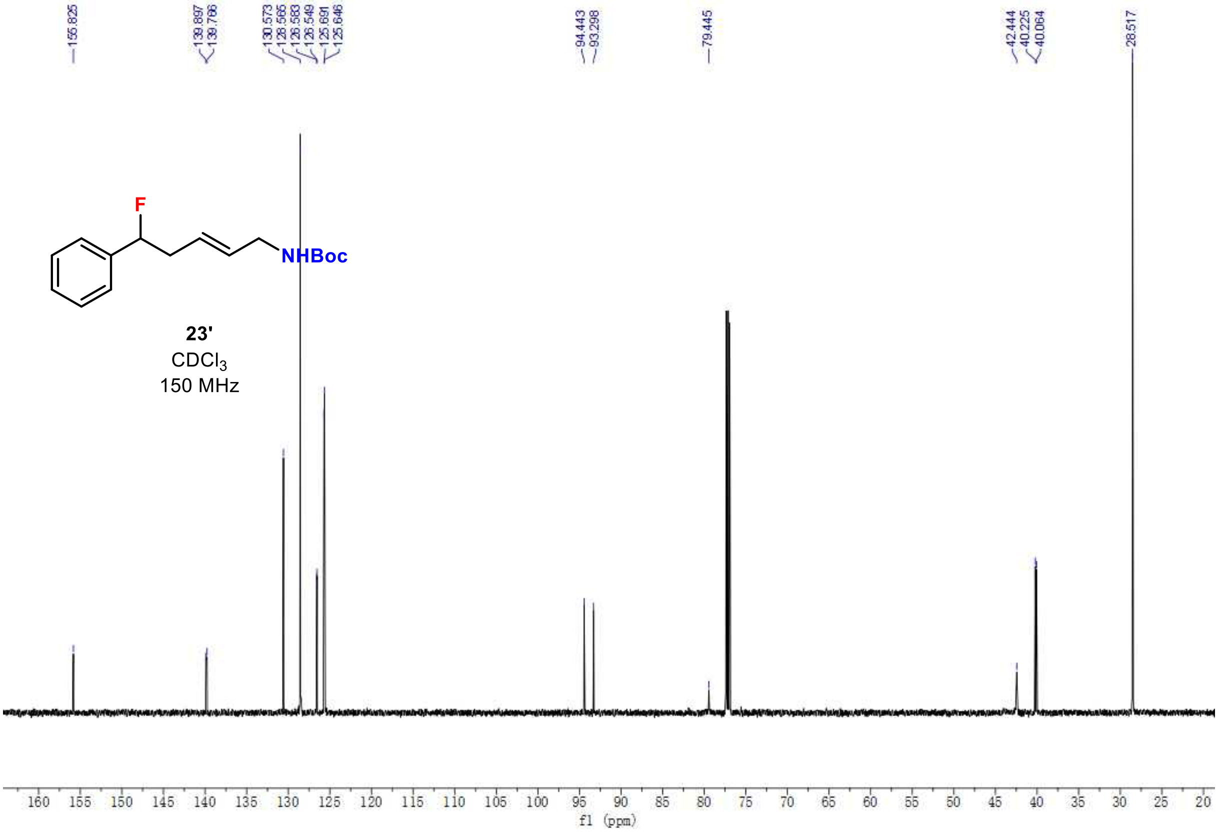

Supplementary Fig. 246. **^{13}C NMR** Spectra of **23'**

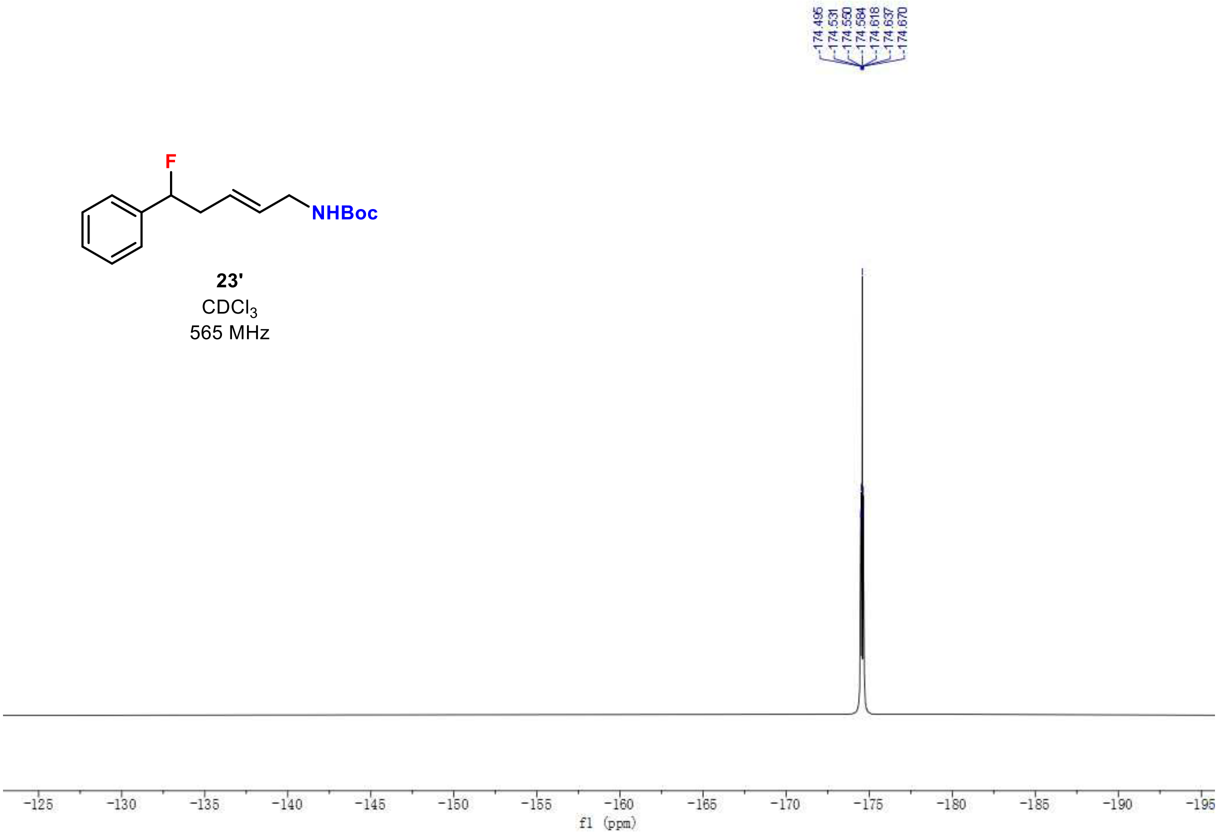

Supplementary Fig. 247. ^{19}**F NMR** Spectra of **23'**

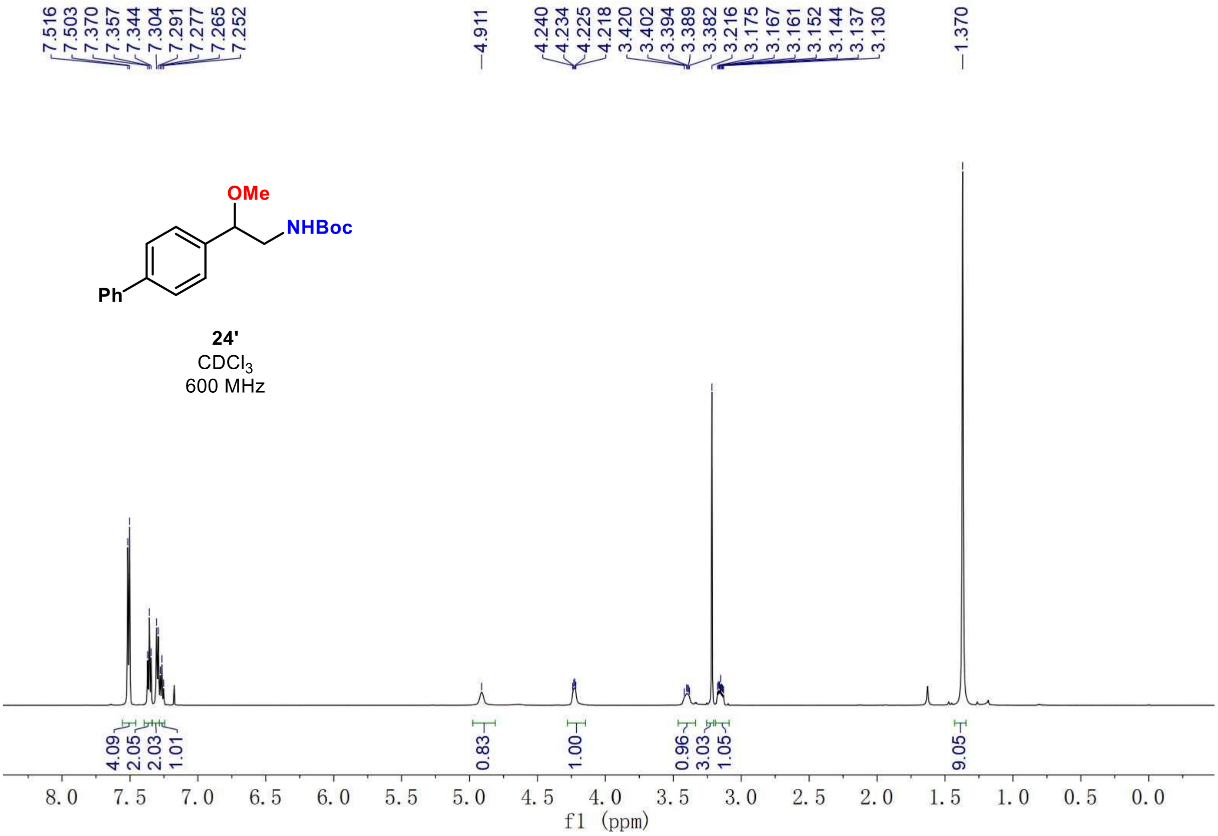

Supplementary Fig. 248. ^{1}**H NMR** Spectra of **24'**

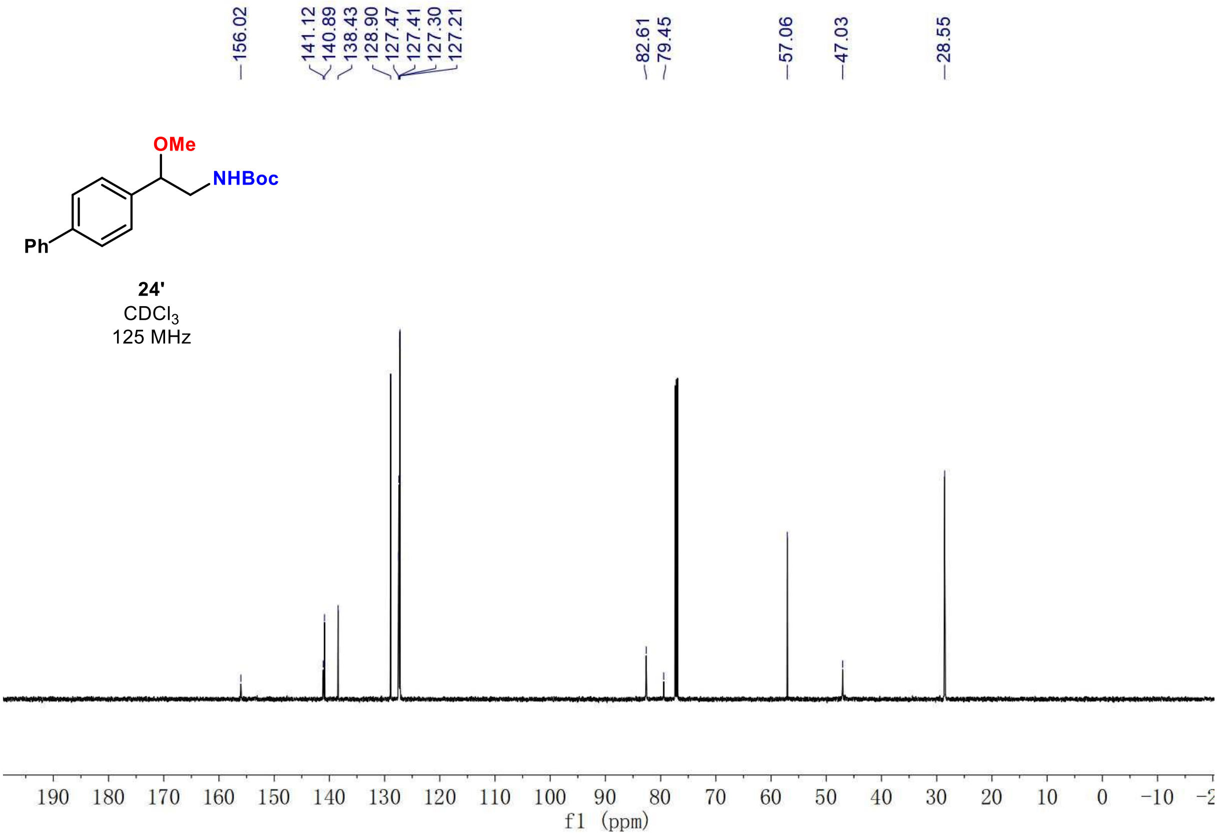

Supplementary Fig. 249. ^{13}**C NMR** Spectra of **24'**

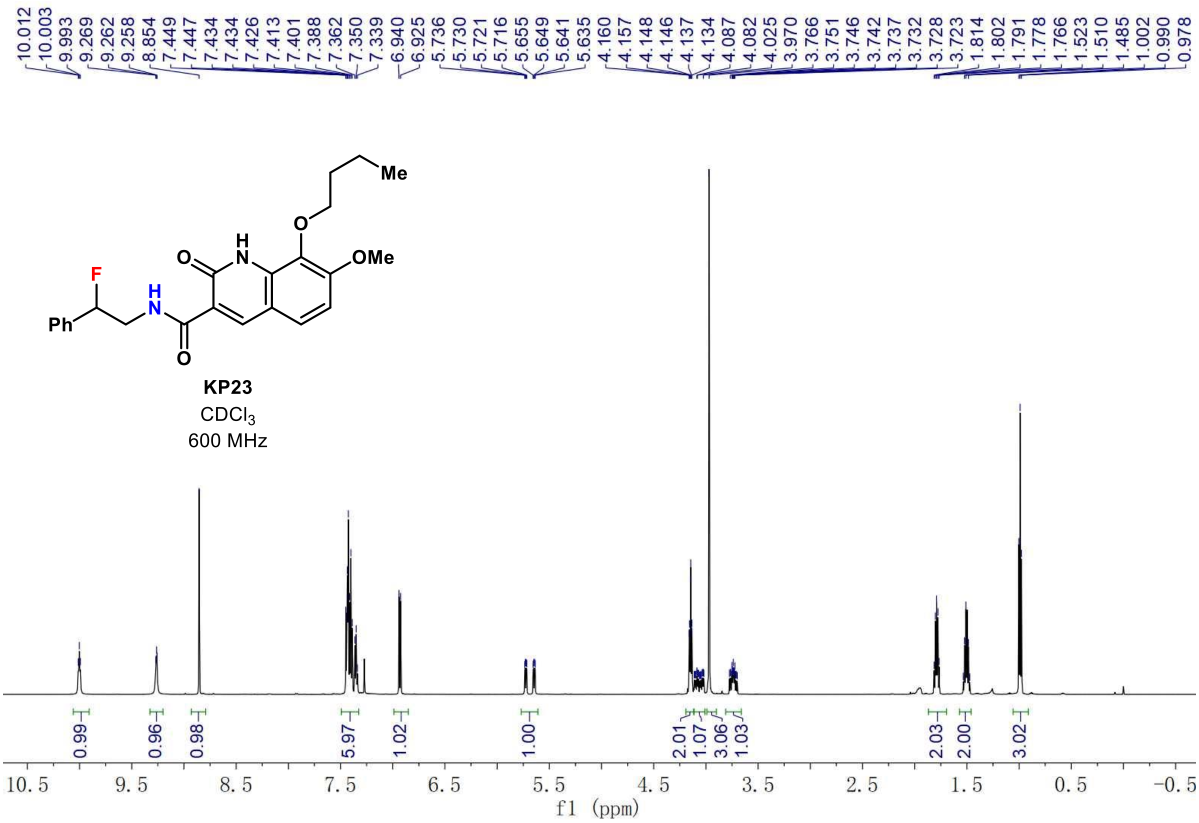

Supplementary Fig. 250. ^{1}**H NMR** Spectra of **KP23**

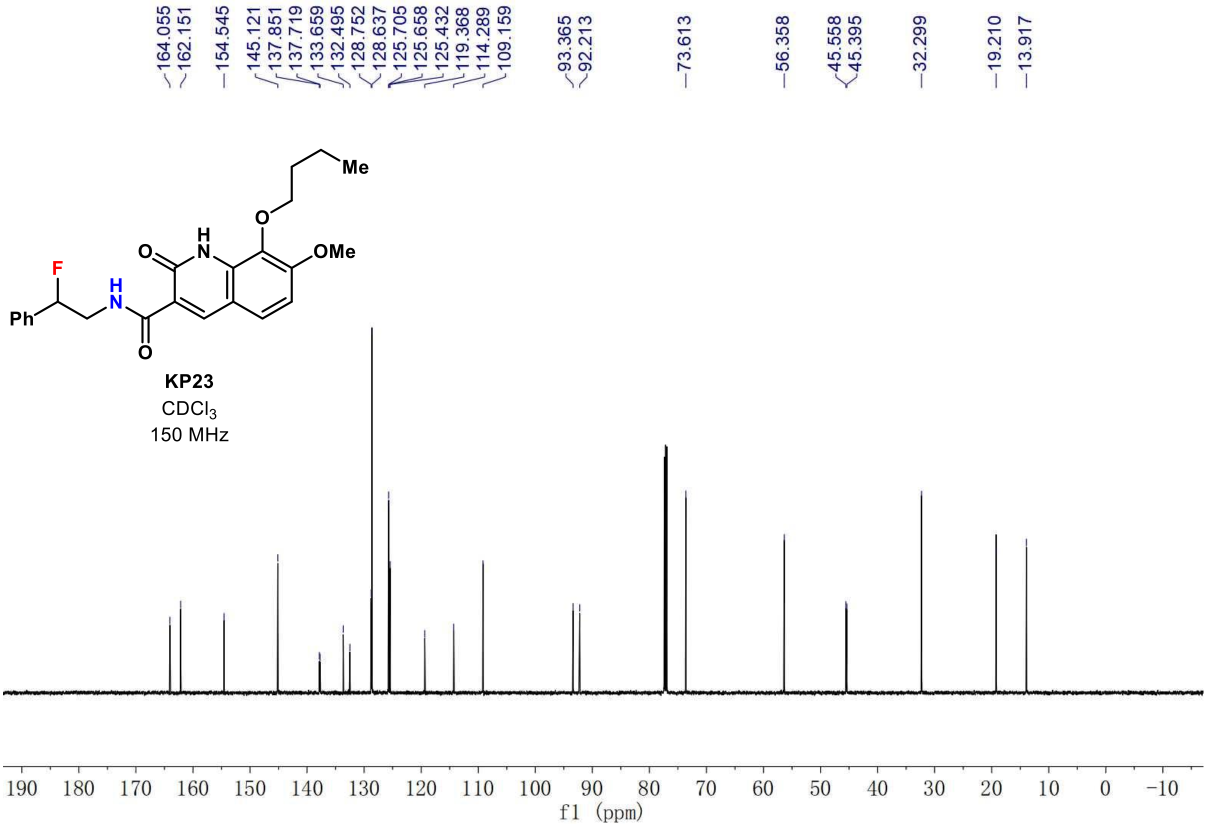

Supplementary Fig. 251. ^{13}**C NMR** Spectra of **KP23**

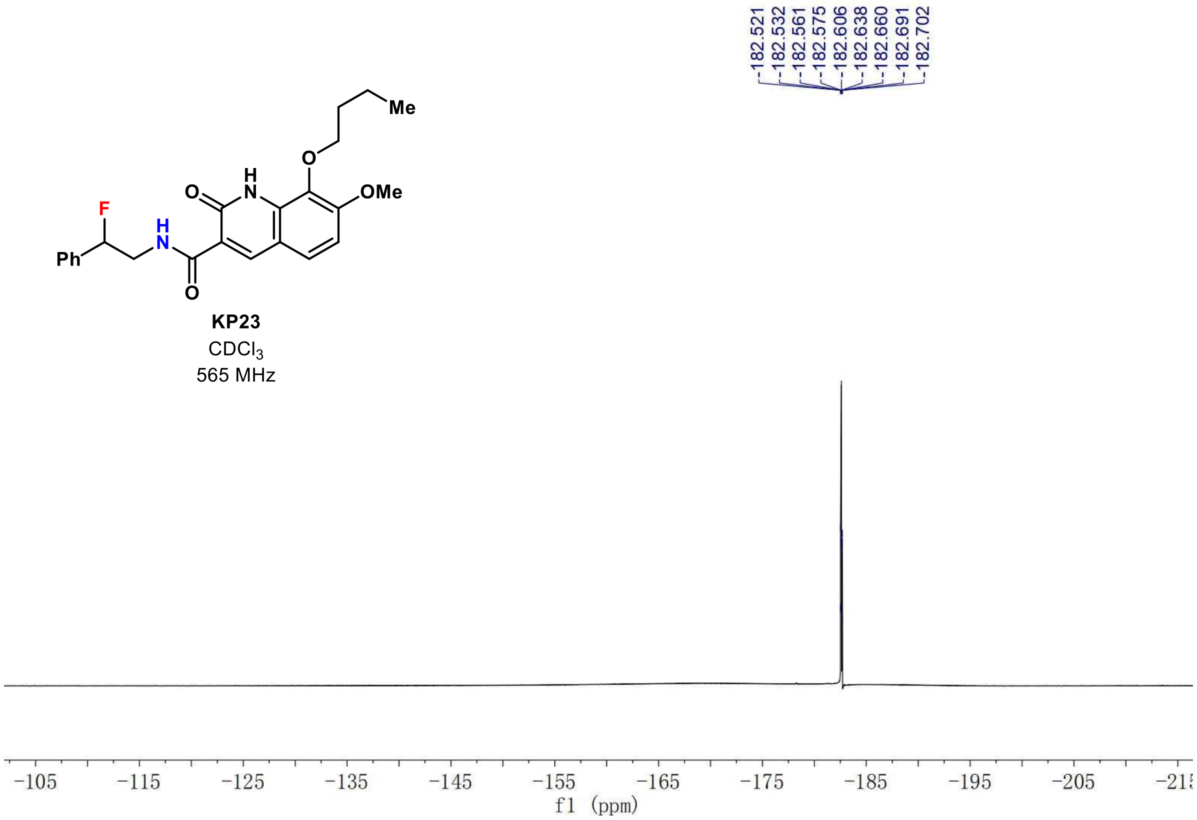

Supplementary Fig. 252. ^{19}**F NMR** Spectra of **KP23**

Part 9: References

[S1] Frisch, M. J.; Trucks, G. W.; Schlegel, H. B.; Scuseria, G. E.; Robb, M. A.; Cheeseman, J. R.; Scalmani, G.; Barone, V.; Mennucci, B.; Petersson, G. A.; Nakatsuji, H.; Caricato, M.; Li, X.; Hratchian, H. P.; Izmaylov, A. F.; Bloino, J.; Zheng, G.; Sonnenberg, J. L.; Hada, M.; Ehara, M.; Toyota, K.; Fukuda, R.; Hasegawa, J.; Ishida, M.; Nakajima, T.; Honda, Y.; Kitao, O.; Nakai, H.; Vreven, T.; Montgomery, J. A. J.; Peralta, J. E.; Ogliaro, F.; Bearpark, M.; Heyd, J. J.; Brothers, E.; Kudin, K. N.; Staroverov, V. N.; Kobayashi, R.; Normand, J.; Raghavachari, K.; Rendell, A.; Burant, J. C.; Iyengar, S. S.; Tomasi, J.; Cossi, M.; Rega, N.; Millam, M. J.; Klene, M.; Knox, J. E.; Cross, J. B.; Bakken, V.; Adamo, C.; Jaramillo, J.; Gomperts, R.; Stratmann, R. E.; Yazyev, O.; Austin, A. J.; Cammi, R.; Pomelli, C.; Ochterski, J. W.; Martin, R. L.; Morokuma, K.; Zakrzewski, V. G.; Voth, G. A.; Salvador, P.; Dannenberg, J. J.; Dapprich, S.; Daniels, A. D.; Farkas, Ö.; Foresman, J. B.; Ortiz, J. V.; Cioslowski, J.; Fox, D. J. *Gaussian 09, Revision D.01;* **2009**, Gaussian, Inc.: Wallingford, CT, .

[S2] Becke, A. D. Density-functional exchange-energy approximation with correct asymptotic behavior. *Phys. Rev. A* **1988**, *38*, 3098-3100.

[S3] Becke, A. D. Density-functional thermochemistry. III. The role of exact exchange. *J. Chem. Phys.* **1993**, *98*, 5648-5652.

[S4] Stephens, P. J.; Devlin, F. J.; Chabalowski, C. F.; Frisch, M. J. Ab Initio Calculation of Vibrational Absorption and Circular Dichroism Spectra Using Density Functional Force Fields. *J. Phys. Chem.* **1994**, *98*, 11623-11627.

[S5] Chen, H.; Ikeda-Saito, M.; Shaik, S. Nature of the Fe−O_2 Bonding in Oxy-Myoglobin: Effect of the Protein. *J. Am. Chem. Soc.* **2008**, *130*, 14778-14790.

[S6] Mallick, D.; Shaik, S. Kinetic Isotope Effect Probes the Reactive Spin State, As Well As the Geometric Feature and Constitution of the Transition State during H-Abstraction by Heme Compound II Complexes. *J. Am. Chem. Soc.* **2017**, *139*, 11451-11459.

[S7] Srnec, M.; Wong, S. D.; England, J.; Que, L.; Solomon, E. I. π-Frontier molecular orbitals in S=2 ferryl species and elucidation of their contributions to reactivity. *Proc. Natl. Acad. Sci. U.S.A.* **2012**, *109*, 14326-14331.

[S8] Pandey, B.; Jaccobab, M.; Rajaraman, G. Mechanistic insights into intramolecular ortho-amination/hydroxylation by nonheme Fe IV QNTs/Fe IV QO species: the r vs. the p channels1. *Chem. Commun.* **2017**, *53*, 3193-3196.

[S9] Li, Y.; Chen, J.-Y.; Miao, Q.; Yu, X.; Feng, L.; Liao, R.-Z.; Ye, S.; Tung, C.-H.; Wang, W. A Parent Iron Amido Complex in Catalysis of Ammonia Oxidation. *J. Am. Chem. Soc.* **2022**, *144*, 4365-4375.

[S10] Weigend, F.; Ahlrichs, R. Balanced basis sets of split valence, triple zeta valence and quadruple zeta valence quality for H to Rn: Design and assessment of accuracy. *Phys. Chem. Chem. Phys.* **2005**, *7*, 3297-3305.

[S11] Marenich, A. V.; Cramer, C. J.; Truhlar, D. G. Universal Solvation Model Based on Solute Electron Density and on a Continuum Model of the Solvent Defined by the Bulk Dielectric Constant and Atomic Surface Tensions. *J. Phys. Chem. B* **2009**, *113*, 6378-6396.

[S12] Lu, D. F.; Zhu, C. L.; Sears, J. D; Xu. H. Iron(II)-Catalyzed Intermolecular Aminofluorination of

Unfunctionalized Olefins Using Fluoride Ion. *J. Am. Chem. Soc.* **2016**, *138*, 11360-11367.
[S13] Lv, D. Q.; Sun, H. Z.; Ge, L.; Qu, Y.; Li, T.; Ma, X.; Li, Y.; Bao, H. Iron-Catalyzed Radical Asymmetric Aminoazidation and Diazidation of Styrenes. *Angew. Chem. Int. Ed.* **2021**, *60*, 12455-12460.
[S14] Yu, D.; Shin, K.-P.; Liu, Y.; Liu H.; Che, C.-M. Ruthenium porphyrin catalysed intermolecular amino-oxyarylation of alkenes to give primary amines via a ruthenium nitrido intermediate. *Chem. Commun.* **2020**, *56*, 137-140.
[S15] Li, Y.; Bao, J.; Zhang, Y.; Peng, X.; Yu, W.; Wang, T.; Yang, D.; Liu, Q.; Zhang, Q.; Fu, J. Three-Component Aminofluorination of Alkenes with Electronically Rich Amino Sources. *Chem* **2022**, *8*, 1147-1163.
[S16] Qin, J.-H.; Luo, M.-J.; An, D.-L.; Li, J.-H. Electrochemical 1,2-Diarylation of Alkenes Enabled by Direct Dual C–H Functionalizations of Electron-Rich Aromatic Hydrocarbons. *Angew. Chem. Int. Ed.* **2021**, *60*, 1861-1868.
[S17] Patra, S.; Mosiagin, I.; Giri, R.; Nauser, T.; Katayev, D. Electron-Driven Nitration of Unsaturated Hydrocarbons. *Angew. Chem.Int. Ed.* **2023**, *62*, e2023005.
[S18] Moon, J.; Jang, M.; Lee, S. Palladium-Catalyzed Decarboxylative Coupling of Alkynyl Carboxylic Acids and Aryl Halides. *J. Org. Chem.* **2009**, *74*, 1403-1406.
[S19] Li, C.-J.; Meng, Y.; Yi, X.-H.; Ma, J.; Chan, T.-H. Manganese-Mediated Carbon-Carbon Bond Formation in Aqueous Media: Chemoselective Allylation and Pinacol Coupling of Aryl Aldehydes. *J. Org. Chem.* **1998**, *63*, 7498-7504.
[S20] Yang, B.; Xu, X.-H.; Qing, F.-L. Copper-Mediated Radical 1,2-Bis(trifluoromethylation) of Alkenes with Sodium Trifluoromethanesulfinate. *Org. Lett.* **2015**, *17*, 1906-1909.
[S21] Movahhed, S.; Westphal, J.; Dindarog,ˋ1. M.; Falk, A.; Schmalz, H.-G. Low-Pressure Cobalt-Catalyzed Enantioselective Hydrovinylation of Vinylarenes. *Chem. Eur. J.* **2016**, *22*, 7381-7384.
[S22] Youn, S. W.; Pastine, S. J.; Sames, D. Ru(III)-Catalyzed Cyclization of Arene-Alkene Substrates via Intramolecular Electrophilic Hydroarylation. *Org. Lett.* **2004**, *6*, 581-584.
[S23] Huang, Z.; Guan, R.; Shanmugam, M.; Bennett, E. L.; Robertson, C. M.; Brookfield, A. E.; McInnes, J. L.; Xiao, J. Oxidative Cleavage of Alkenes by O_2 with a Non-Heme ManganeseCatalyst. *J. Am. Chem. Soc.* **2021**, *143*, 10005-10013.
[S24] Ghosh, A. K.; Tomaine, A. J.; Cantwell, K. E. Lewis Acid Mediated Cyclizations: Diastereoselective Synthesis of Six- to Eight-Membered Substituted Cyclic Ethers. *Synthesis* **2017**, *49*, 4229-246.
[S25] Connolly, T.; Wang, Z.; Walker, M. A., McDonald, I. M.; Peese. K. M. Tandem Ring-Closing Metathesis /Transfer Hydrogenation: PracticalChemoselective Hydrogenation of Alkenes. *Org. Lett.* **2014**, *16*, 4444-4447.
[S26] Matsubara, S.; Mizuno, T.; Otake, T.; Kobata, M.; Utimoto, K.; Takai, K. Alkylidenation of carbonyl compounds with gem-dizincioalkanes mediated with titanium dichloride. *Synlett* **1998**, *12*, 1369-1371.
[S27] Wu, Q.; Zhao, Y.-H.; Chai, L.-L.; Li, H.-Y.; Li, H.-X. Metal-free photocleavage of the C(non-acyl)–S bond of thioesters for regioselective pyridylthioesterification of styrenes. *Org. Chem. Front.* **2022**, *9*, 2977-2985.
[S28] Reichle, A.; Koch, M.; Sterzel, H.; Großkopf, L.-J.; Floss, J.; Rehbein, J.; Reiser, O. Copper(I) Photocatalyzed Bromonitroalkylation of Olefins: Evidence for Highly Efficient Inner-Sphere Pathways. *Angew. Chem.Int. Ed.* **2023**,

62, e202219086.

[S29] Chen, B.; Cao, P.; Yin, X.; Liao, Y.; Jiang, L.; Ye, J.; Wang, M.; Liao, J. Modular Synthesis of Enantioenriched 1,1,2-Triarylethanes by an Enantioselective Arylboration and Cross-Coupling Sequence. *ACS Catal.* **2017**, *7*, 2425 - 2429.

[S30] Turkman, N.; Shavrin, A.; Ivanov, R. A.; Rabinovich, B.; Volgin,A.; Gelovani, J. G.; Alauddin, M. M. Fluorinated cannabinoid CB_2 receptor ligands: synthesis and in vitro binding characteristics of 2-oxoquinoline derivatives. *Bioorg. Med. Chem.* **2011**, *19*, 5698-5707.

[S31] Morales, C. M.; See, Y. Y.; Lee, S. J.; Scott, P. J. H.; Bland, D. C.; Sanford, M. S. Tetramethylammonium Fluoride Alcohol Adducts for SNAr Fluorination. *Org. Lett.* **2021**, *23*, 4493-4498.

[S32] Herth, M. M., et al. On the consensus nomenclature rules for radiopharmaceutical chemistry – Reconsideration of radiochemical conversion. *Nucl. Med. Biol.* **2021**, *93*, 19-21.
