# Supplementary material for: Iron-catalyzed three-component amino(radio)fluorination of alkenes to unprotected β-(radio)fluoroamines
Source: Nat Commun. 2025 Dec 5;16:10917. doi: 10.1038/s41467-025-65880-z (PMC12680689; doi:10.1038/s41467-025-65880-z)
Supplement: Supplementary file 2 — Description of Additional Supplementary Files [file 41467_2025_65880_MOESM2_ESM.pdf]

Description of Additional Supplementary Files

File Name: Supplementary Data 1

Description: Cartesian coordinates of optimized structures.
